# Supplementary material for: CETN3 deficiency induces microcephaly by disrupting neural stem/progenitor cell fate through impaired centrosome assembly and RNA splicing
Source: EMBO Mol Med. 2025 Sep 8;17(10):2735–61. doi: 10.1038/s44321-025-00302-7 (PMC12514221; doi:10.1038/s44321-025-00302-7)
Supplement: Supplementary file 3 — Appendix [file 44321_2025_302_MOESM3_ESM.pdf]

APPENDIX

CETN3 Deficiency Induces Microcephaly by Disrupting Neural Stem/Progenitor Cell Fate through Impaired Centrosome Assembly and RNA Splicing

Table of contents:

Appendix	Description	Page
Appendix Figure S1	Conservation of CETN3 in evolution.	2
Appendix Figure S2	The generation of *CETN3*-KO cell lines based on H9.	3
Appendix Figure S3	Deficiency of CETN3 interferes with differentiation and proliferation of NS/PCs in hCOs derived from H9.	4
Appendix Figure S4	Introduction of biallelic *CETN3* mutations into WT iPSCs.	5
Appendix Figure S5	The role of CETN3 in RNA splicing.	6
Appendix Table S1	Primers used in the study.	7
Appendix Table S2	Primary antibodies used in the study.	9

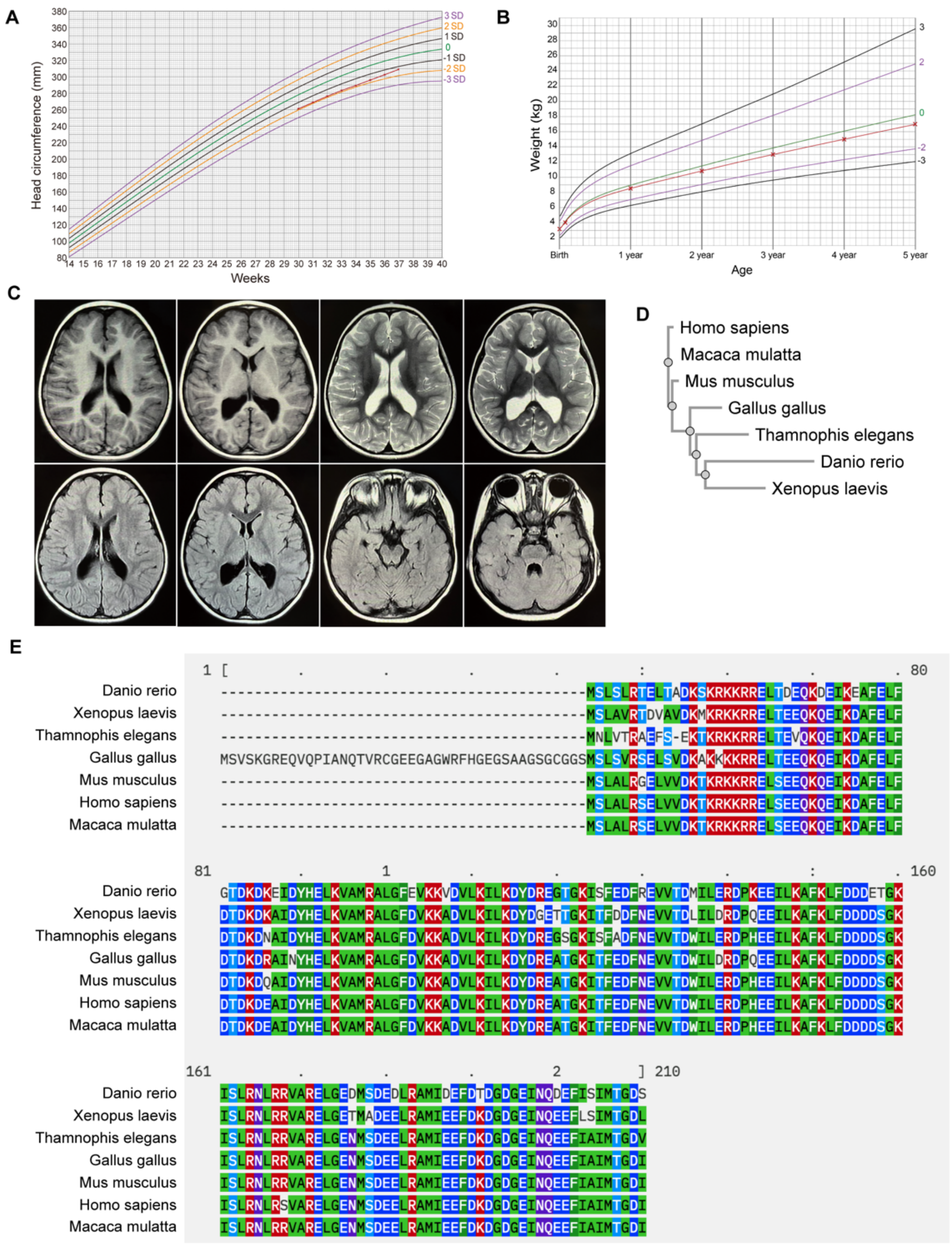

Appendix Figure S1. Conservation of CETN3 in evolution.

(**A, B**) Head circumference during the embryonic period (**A**) and weight of the patient (**B**). Red lines with forks represent data of the patient, and the numbers on other lines indicate SD from the average. (**C**) MRI of the patient's head at 3 years old. Upper left: T1-weighted images; upper right: T2-weighted images; lower: FLAIR images. (**D**) Cladogram of CETN3. (**E**) Multiple sequence alignment of CETN3 protein in different species using MUSCLE 3.8.

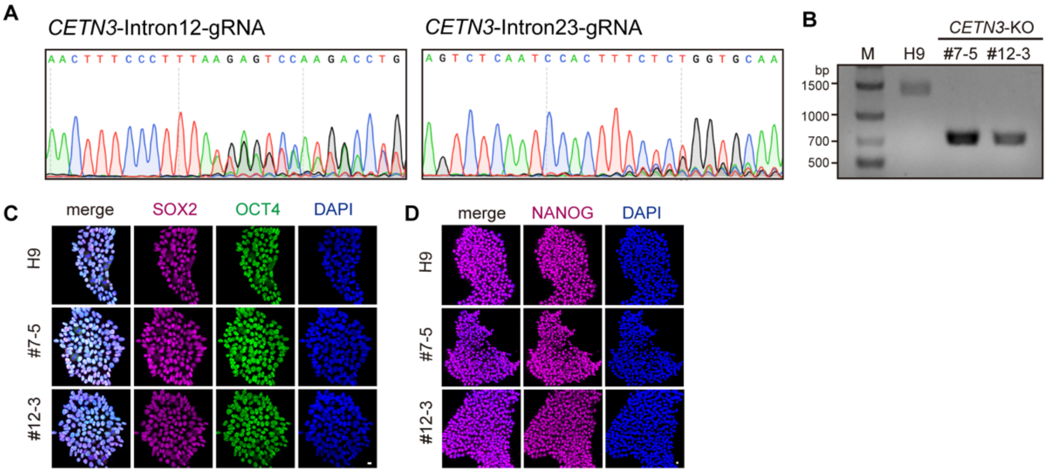

Appendix Figure S2. The generation of *CETN3*-KO cell lines based on H9.

(**A**) Evaluation of the editing efficiency of two sgRNAs targeting *CETN3* in HEK 293T cells. (**B**) Genotyping results for *CETN3*-KO cell lines. Clones #7-5 and #12-3 exhibited successful deletion of exon 2 (136 bp) from the *CETN3* locus. (**C, D**) Immunofluorescence staining for pluripotency markers SOX2, OCT4, and NANOG in *CETN3*-KO cell lines to assess maintenance of pluripotency. Scale bar: 10μm.

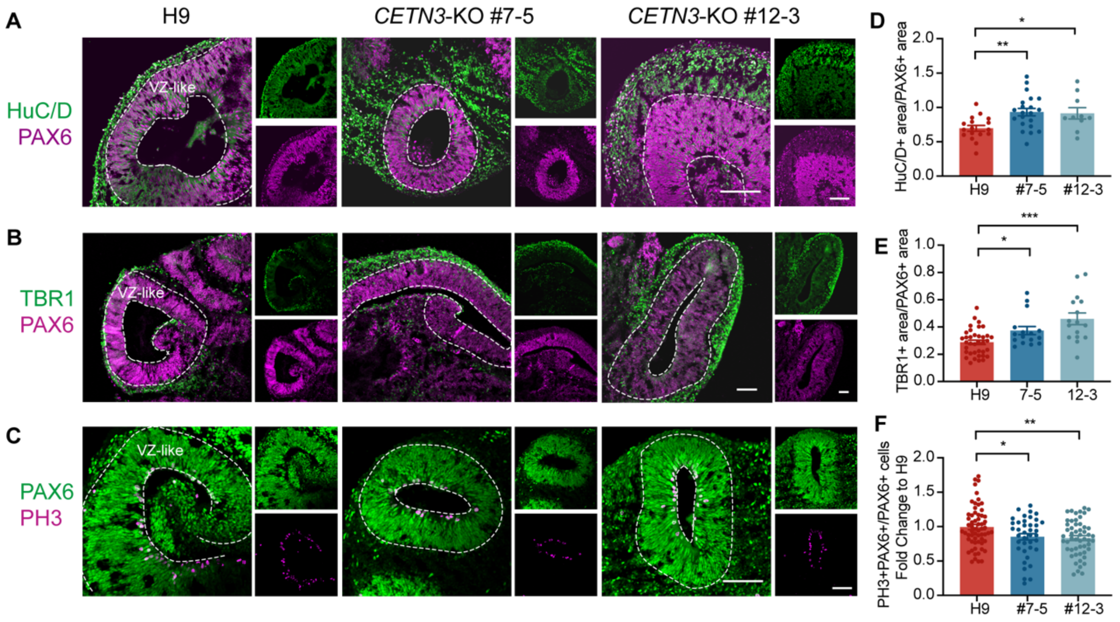

Appendix Figure S3. Deficiency of CETN3 interferes with differentiation and proliferation of NS/PCs in hCOs derived from H9.

(**A-C**) Immunofluorescence staining of cryosections from day 35 hCOs. NS/PCs were labeled with PAX6, neurons were identified using HuC/D or TBR1, and cell proliferation was evaluated by PH3 staining. Scale bar: 100μm. VZ: ventricular zone (**D-F**) Quantification of cell number or staining area for each marker. Each dot represented an individual rosette or several adjacent rosettes (D: H9, n = 17, #7-5, n = 21, #12-3, n = 10; E: H9, n = 35, #7-5, n = 16, #12-3, n = 14; F: H9, n = 61, #7-5, n = 39, #12-3, n = 53). Data were collected from organoids across three independent experiments, with results presented as mean ± SEM. D: *P* = 0.0051 (H9 vs. #7-5), *P* = 0.0372 (H9 vs. #12-3); E: *P* = 0.0329 (H9 vs. #7-5), *P* = 0.0002 (H9 vs. #12-3); F: *P* = 0.0315 (H9 vs. #7-5), *P* = 0.0063 (H9 vs. #12-3). Differential analysis was performed using one-way ANOVA. $^*P < 0.05$; $^{**}P < 0.01$; $^{***}P < 0.001$.

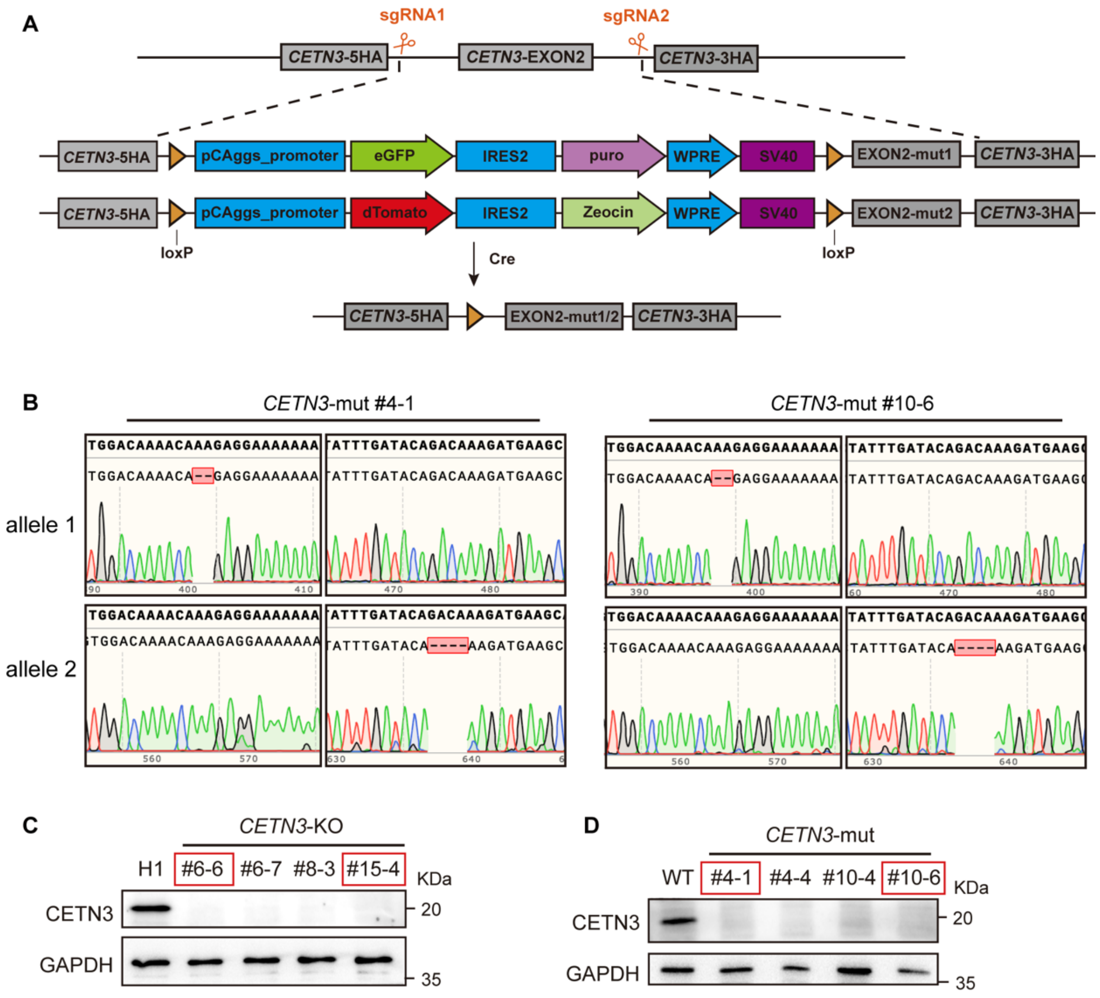

Appendix Figure S4. Introduction of biallelic *CETN3* mutations into WT iPSCs.

(**A**) The strategy for generating *CETN3*-mut cell lines based on WT iPSCs using CRISPR-Cas9 technique involved two sgRNAs targeting intron 1-2 and intron 2-3 to cleave the genomic DNA. WT exon 2 of *CETN3* was replaced with mutant exon 2, plus an exogenous DNA fragment containing selection elements flanked by loxP sites. Clones exhibiting both red and green fluorescence were selected. Cre recombinase was subsequently used to delete the exogenous sequence, thereby replacing exon 2 of *CETN3*. (**B**) Sanger sequencing to confirm the simultaneous introduction of two mutations in *CETN3*. Mutation sites were highlighted by red rectangles. (**C, D**) Western blot validating the loss of CETN3 protein in selected clones. Clones chosen for subsequent experiments were marked with red rectangles.

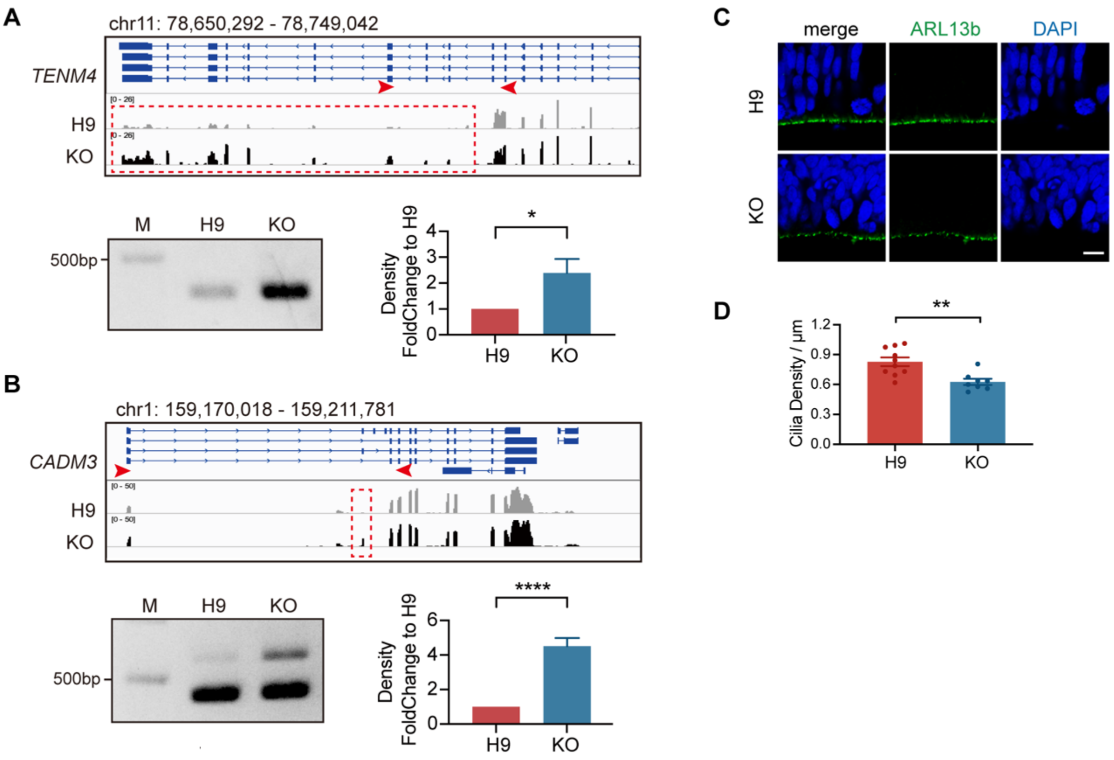

Appendix Figure S5. The role of CETN3 in RNA splicing.

(**A, B**) Splicing information for 2 representative neurodevelopment-related genes, *TENM4* and *CADM3*, in H9 and *CETN3*-KO NS/PCs. IGV tracks depicted the selected regions of target genes, with alternatively spliced exons highlighted by red dashed frame (upper). Alternative splicing was validated by RT-PCR, with primers indicated by red arrows in the upper panel (lower left). Quantification of PCR results was shown as mean ± SEM, collected from 3 batches and normalized to H9 (lower right). A: $P = 0.0417$ (H9 vs. KO); B: $P < 0.0001$ (H9 vs. KO). Unpaired t-test was used for differential analysis. *$P < 0.05$; ****$P < 0.0001$. M: marker. (**C**) Immunofluorescence of H9 and *CETN3*-KO organoids at day 45. Marker for cilia, ARL13b, was stained. Regions of interest of 400 × 400 pixels on apical-like surface were randomly selected for visualization and statistical analysis. Scale bar: 10µm. (**D**) Cilia density analysis of panel K. Each dot indicated a rosette (H9, n = 10; KO, n = 8). 6 organoids from 2 independent experiment were used for counting. Results was shown as mean ± SEM. $P = 0.0024$ (H9 vs. KO). Unpaired t-test was used for differential analysis. **$P < 0.01$.

Appendix Table S1. Primers used in the study.

name	sequence
cell line	
CETN3-intron1,2-gRNA-F	CACCGACAGGTCATTGACTCATAAA
CETN3-intron1,2-gRNA-R	AAACTTTATGAGTCAATGACCTGTC
CETN3-intron1,2-gRNA-geno-F	TTGCCAAACTGTTAAAAACGTG
CETN3-intron1,2-gRNA-geno-R	GGAAAGAAAAAGCCCTTAGTCC
CETN3-intron2,3-gRNA-F	CACCGTTGTTTGCACCAGAGAAAG
CETN3-intron2,3-gRNA-R	AAACCTTTCTCTGGTGCAAACAAC
CETN3-intron2,3-gRNA-geno-F	AAGCAATAGATTATCATGAATTAAAGGTAATGGTATACTT
CETN3-intron2,3-gRNA-geno-R	ACACTTACATTCACCTATCCATTAAGGCC
CETN3-intron1,2-5HA-F	GCGACGGTATCGATACCGGTTGGGCAGCTCTTAAGGAAAA
CETN3-intron1,2-5HA-R	GCTATACGAAGTTATGCGGCCGCTGAGTCAATGACCTGTTTATTCCA
CETN3-intron2,3-3HA-F	GTTATCTTAAGGGTTTAAACTGGTGCAAACAACTTTAGTGC
CETN3-intron2,3-3HA-R	GGTGGCGGCCTAGGATTTAAATTGAAATAAGGTCCCAAACCAA
CRISPR-insert-genotyping-5'-F	CGTTATTCCAGTGTTGCGGC
CRISPR-insert-genotyping-5'-R	CCGTAAGTTATGTAACGCG
CRISPR-insert-genotyping-3'-F	CACTGCATTCTAGTTGTGGTTTG
CRISPR-insert-genotyping-3'-R	GTGGTAGTGTACAAGGTGAACTAG
CRISPR-insert-genotyping-WT-F	AAGCAATAGATTATCATGAATTAAAGGTAATGGTATACTT
CRISPR-insert-genotyping-WT-R	ACACTTACATTCACCTATCCATTAAGGCC
CRISPR-Cre-genotyping-F	TTGCCAAACTGTTAAAAACGTG
CRISPR-Cre-genotyping-R	ACACTTACATTCACCTATCCATTAAGGCC
qPCR	
hACTIN-F	GGACTTCGAGCAAGAGATGG
hACTIN-R	AGCACTGTGTTGGCGTACAG
hNANOG-F	CAGTCTGGACACTGGCTGAA
hNANOG-R	CTCGCTGATTAGGCTCCAAC
hOCT4-F	TGTACTCCTCGGTCCCTTTC
hOCT4-R	TCCAGGTTTTCTTTCCCTAGC
hSOX2-F	GCTAGTCTCCAAGCGACGAA
hSOX2-F	GCAAGAAGCCTCTCCTTGAA
hDNMT3b-F	ATAAGTCGAAGGTGCGTCGT
hDNMT3b-R	GGCAACATCTGAAGCCATTT
hTERT-F	TGTGCACCAACATCTACAAG

hTERT-R	GCGTTCTTGGCTTTCAGGAT
hREX1-F	TGGACACGTCTGTGCTCTTC
hREX1-R	GTCTTGGCGTCTTCTCGAAC
CoIP plasmid	
myc-CETN3-F	GGGCGGCGGCGGCAGCACCGGTATGAGTTTAGCTCTGAGAAGTGAGCT
myc-CETN3-R	ATTTACGTAGCGGCCGCTTAAATGTCACCAGTCATAATAGCAATGAACTCC
USP44-flag-F	ATCATTTTGGCAAAGAATTCCTCGAGCGCCACCATGCTAGCAATGGATACGTGCAAAC
USP44-flag-R	GTCGCTGCCGCCGCCGCCACCGGTGCTAAGGATTTCATTAGACGAGGTATCAGC
USP49-flag-F	TGGCAAAGAATTCCTCGAGCGCCACCATGGATAGATGCAAACATGTAGGGC
USP49-flag-R	TGTAGTCGCTGCCGCCGCCGCCACCGGTGGAAAATGTCTGTGGTCTGCCT
splicing	
CADM3-F	TCGGTCAACATCGTAGTCCA
CADM3-R	TTCGCACAGGCATAGTGAAG
PCM1-F	TGAGAGCCATGAAAAAGGAGA
PCM1-R	TCTCCACCACACCCTACTCA
CEP250-F	TGGCAGAAATCCTGGGATAA
CEP250-R	ATTCTCTGCCATCTGCTGCT
TENM4-F	AACAACCCCATCAGCAACTC
TENM4-R	TCATTGGCCACACTGATGAT

Appendix Table S2. Primary antibodies used in the study.

antibody	species	dilution	company	Cat. No.	application
MAP2	chicken	1:1000	Abcam	AB5392	IF
PAX6	sheep	1:100	R&D	AF8150	IF
CETN3	rabbit	1:2000 for WB; 1:200 for IF	Abcam	AB228690	IF and WB
α-tubulin	mouse	1:1000	Abclonal	AC012	IF
γ-tubulin	mouse	1:1000	Proteintech	66320-1-Ig	IF
SOX2	rabbit	1:200	Abcam	AB97959	IF
Oct3/4 (C-10)	mouse-IgG2b	1:200	Santa Cruz	sc-5279	IF
NANOG	rabbit	1:200	Protrintech	14295-1-AP	IF
HuC/D	mouse-IgG2b	1:200	Invitrogen	A21271	IF
TBR1	rabbit	1:200	Abcam	AB31940	IF
CTIP2	rat	1:500	Abcam	AB18465	IF
TBR2	sheep	1:200	R&D	AF6166	IF
PH3 (Ser10)	rabbit	1:2000	Millipore	06-570	IF
Ki67	mouse	1:100	BD Pharmingen	550609	IF
Caspase-3	rabbit	1:200	CST	#9664	IF
p-Vimentin	mouse	1:100	Abcam	AB22651	IF
PCNT	rabbit	1:500	Abclonal	A20161	IF
SOX1	goat	1:100	R&D	AF3369	IF
TUJ1	rabbit	1:1000	Abcam	AB18207	IF
ARL13b	rabbit	1:200	Abclonal	A5200	IF
GAPDH	rabbit	1:5000	CST	#2118	WB
β-tubulin	mouse	1:5000	Proteintech	66240-1-Ig	WB
Flag	rabbit	1:5000	CST	#14793	WB
Myc	mouse	1:5000	CST	#2276	WB
IgG	rabbit	1:50	CST	#2729	IP
Flag	mouse	1:50	Sigma	F1804-200UG	IP
Myc	goat	1:50	Abcam	AB9132	IP
