# Supplementary figures and images for: CETN3 deficiency induces microcephaly by disrupting neural stem/progenitor cell fate through impaired centrosome assembly and RNA splicing (part 1 of 5)
Source: EMBO Mol Med. 2025 Sep 8;17(10):2735–61. doi: 10.1038/s44321-025-00302-7 (PMC12514221; doi:10.1038/s44321-025-00302-7)

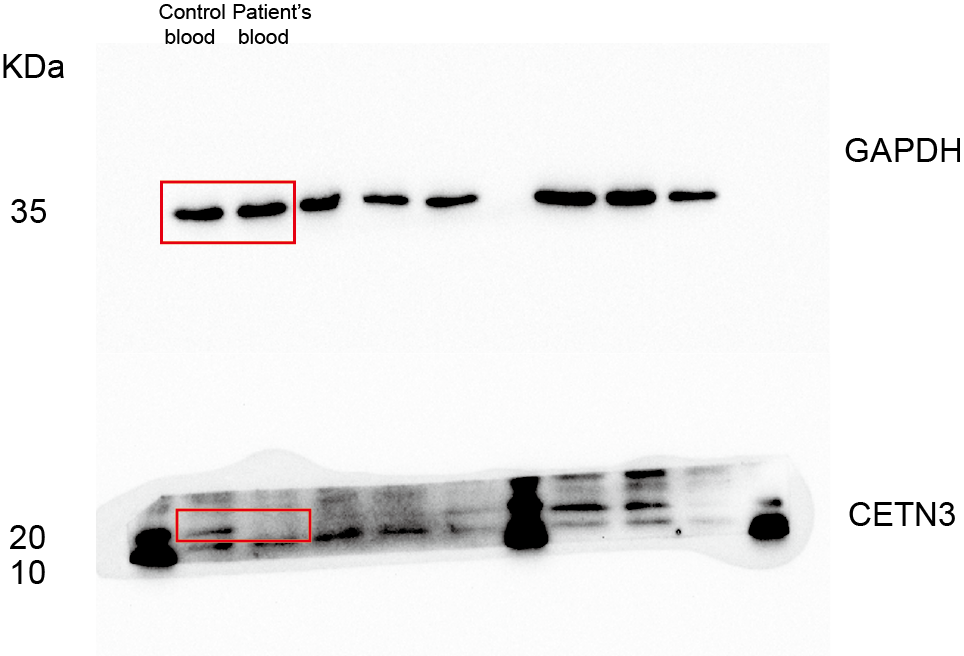

Supplement: Supplementary file 4 — Source data Fig. 1 [file 44321_2025_302_MOESM4_ESM.zip › Figure 1/1E/western blot.png]

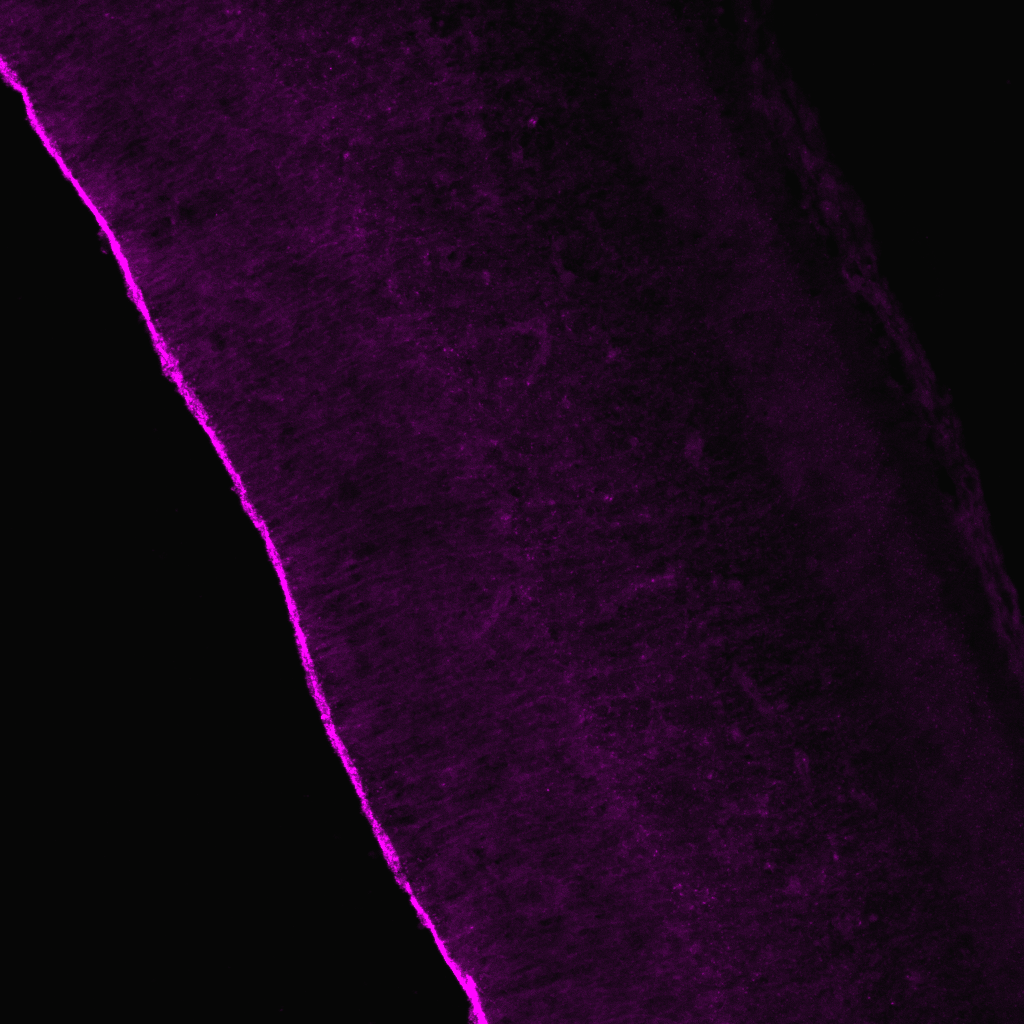

Supplement: Supplementary file 4 — Source data Fig. 1 [file 44321_2025_302_MOESM4_ESM.zip › Figure 1/1I/IF-human-CETN3.tif]

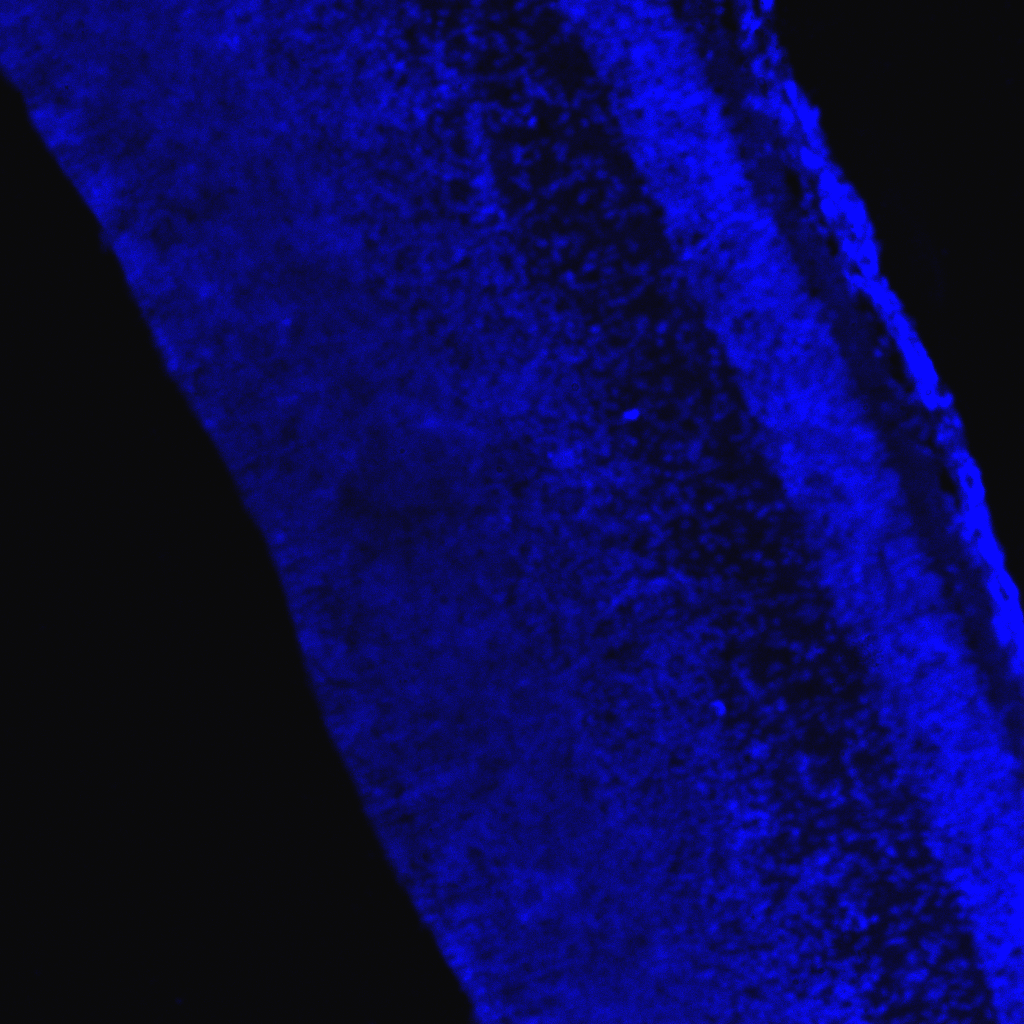

Supplement: Supplementary file 4 — Source data Fig. 1 [file 44321_2025_302_MOESM4_ESM.zip › Figure 1/1I/IF-human-DAPI.tif]

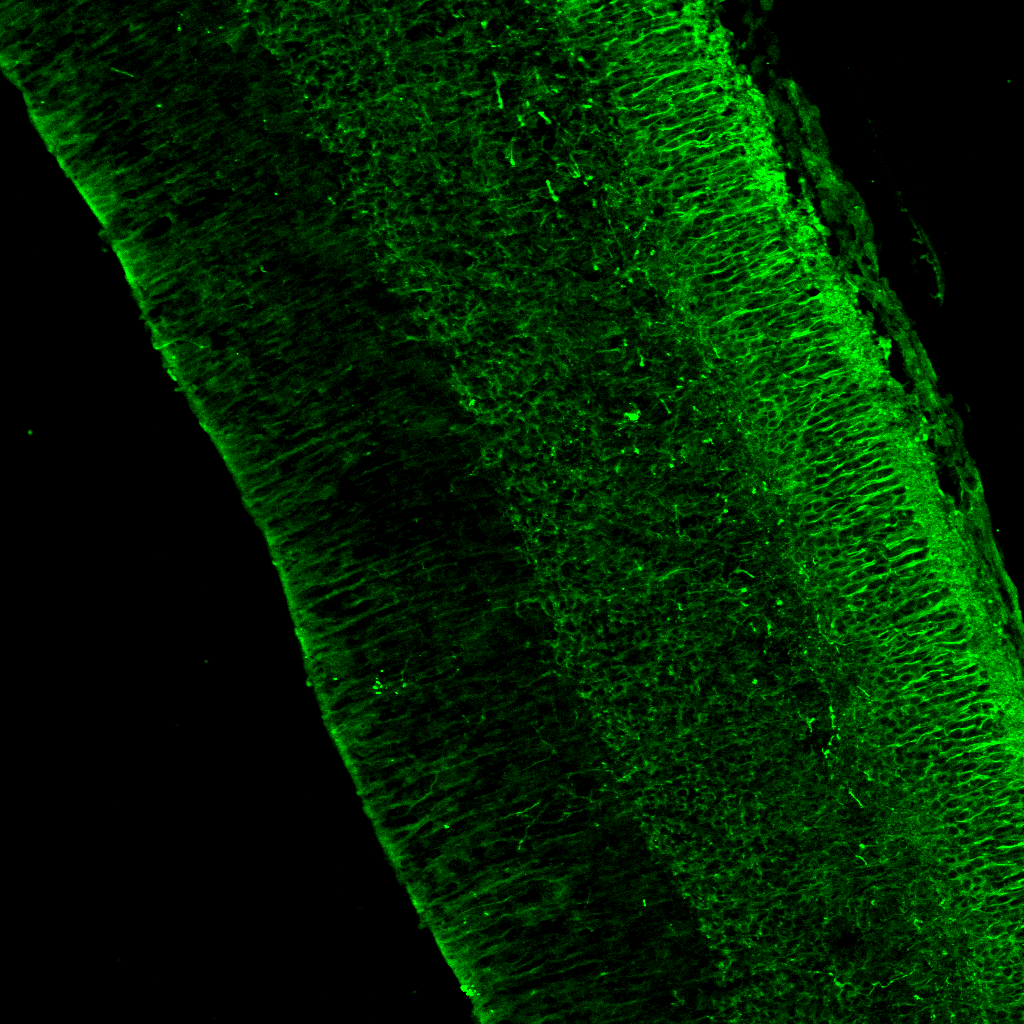

Supplement: Supplementary file 4 — Source data Fig. 1 [file 44321_2025_302_MOESM4_ESM.zip › Figure 1/1I/IF-human-MAP2.tif]

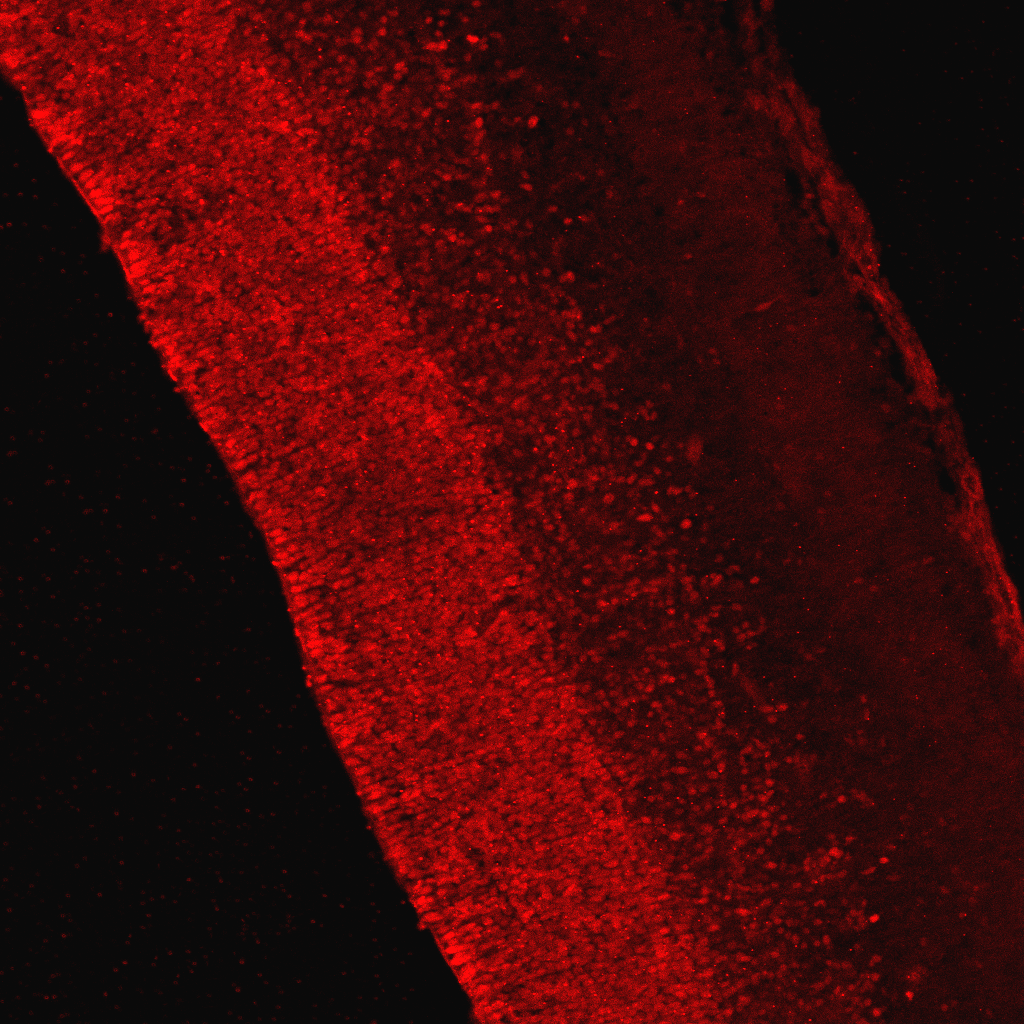

Supplement: Supplementary file 4 — Source data Fig. 1 [file 44321_2025_302_MOESM4_ESM.zip › Figure 1/1I/IF-human-PAX6.tif]

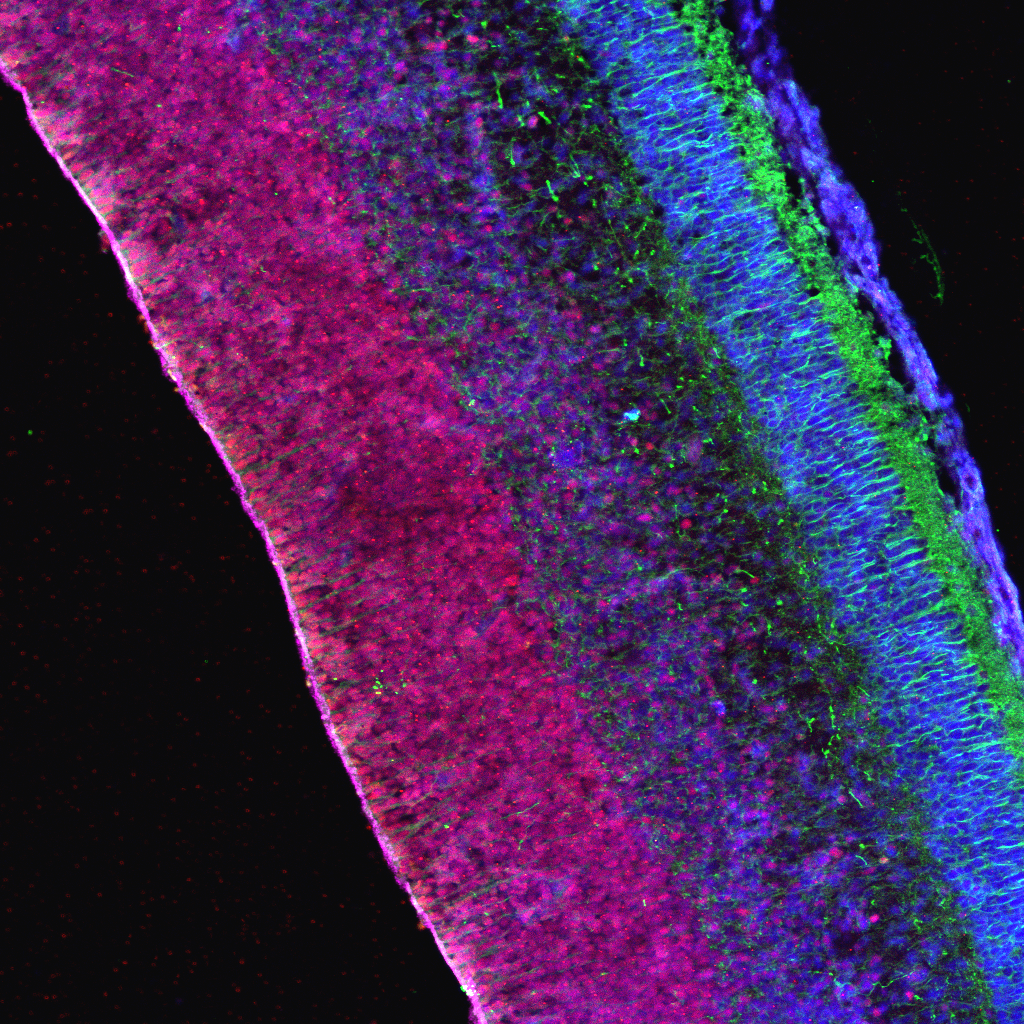

Supplement: Supplementary file 4 — Source data Fig. 1 [file 44321_2025_302_MOESM4_ESM.zip › Figure 1/1I/IF-human-merge.tif]

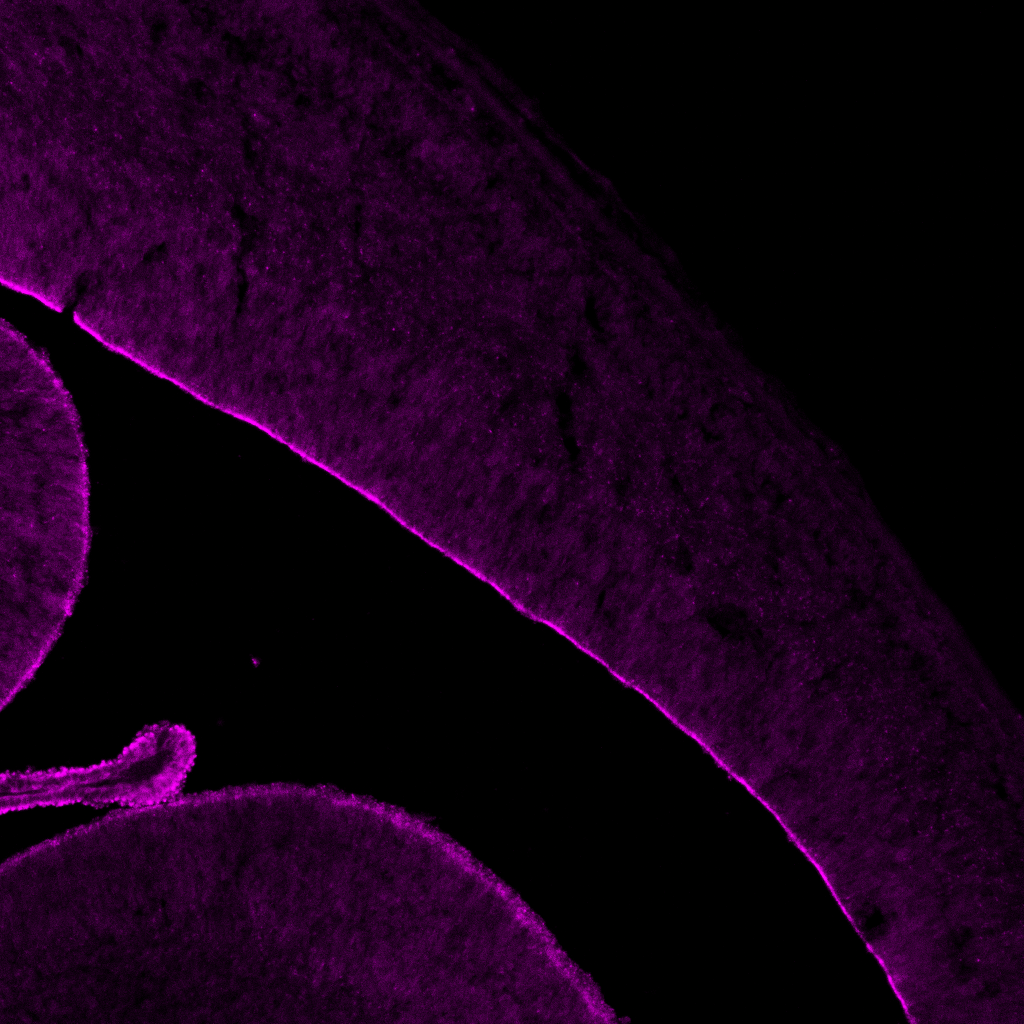

Supplement: Supplementary file 4 — Source data Fig. 1 [file 44321_2025_302_MOESM4_ESM.zip › Figure 1/1I/IF-mouse-CETN3.tif]

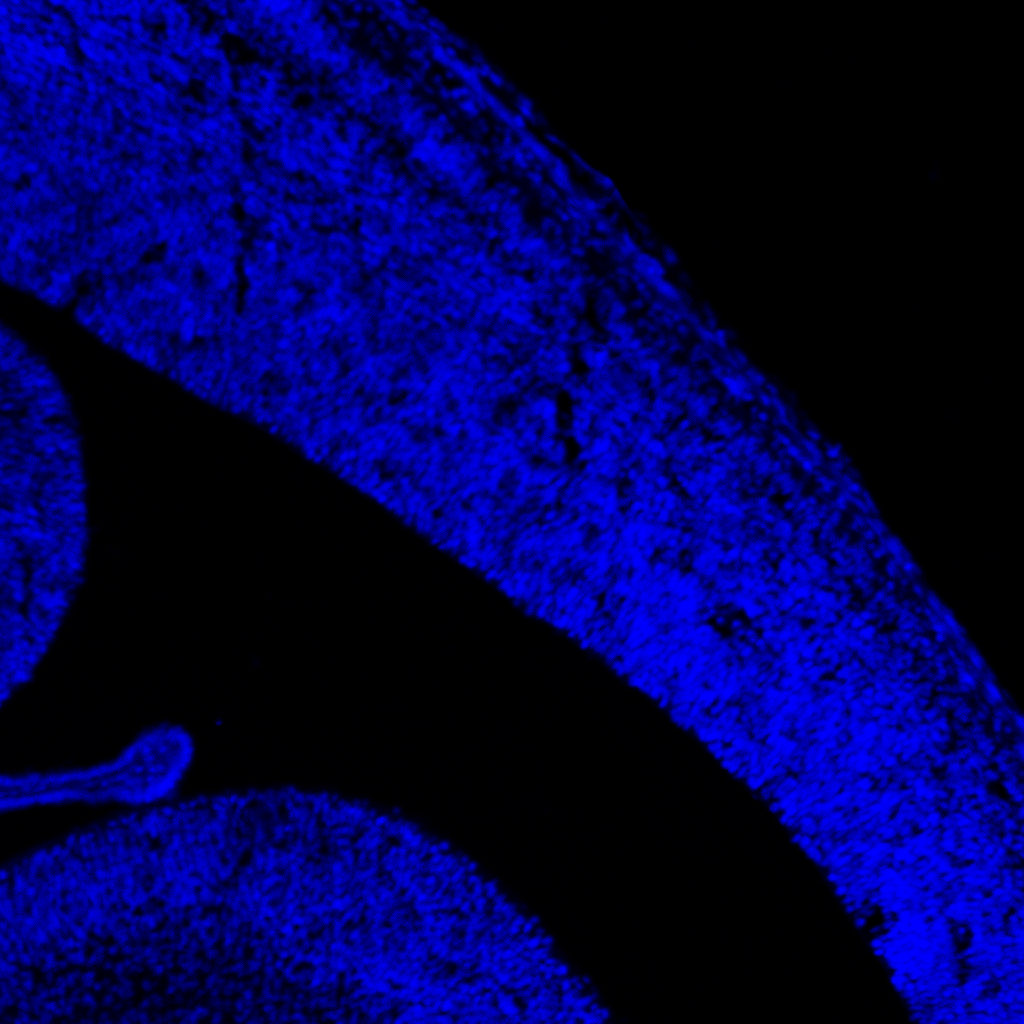

Supplement: Supplementary file 4 — Source data Fig. 1 [file 44321_2025_302_MOESM4_ESM.zip › Figure 1/1I/IF-mouse-DAPI.tif]

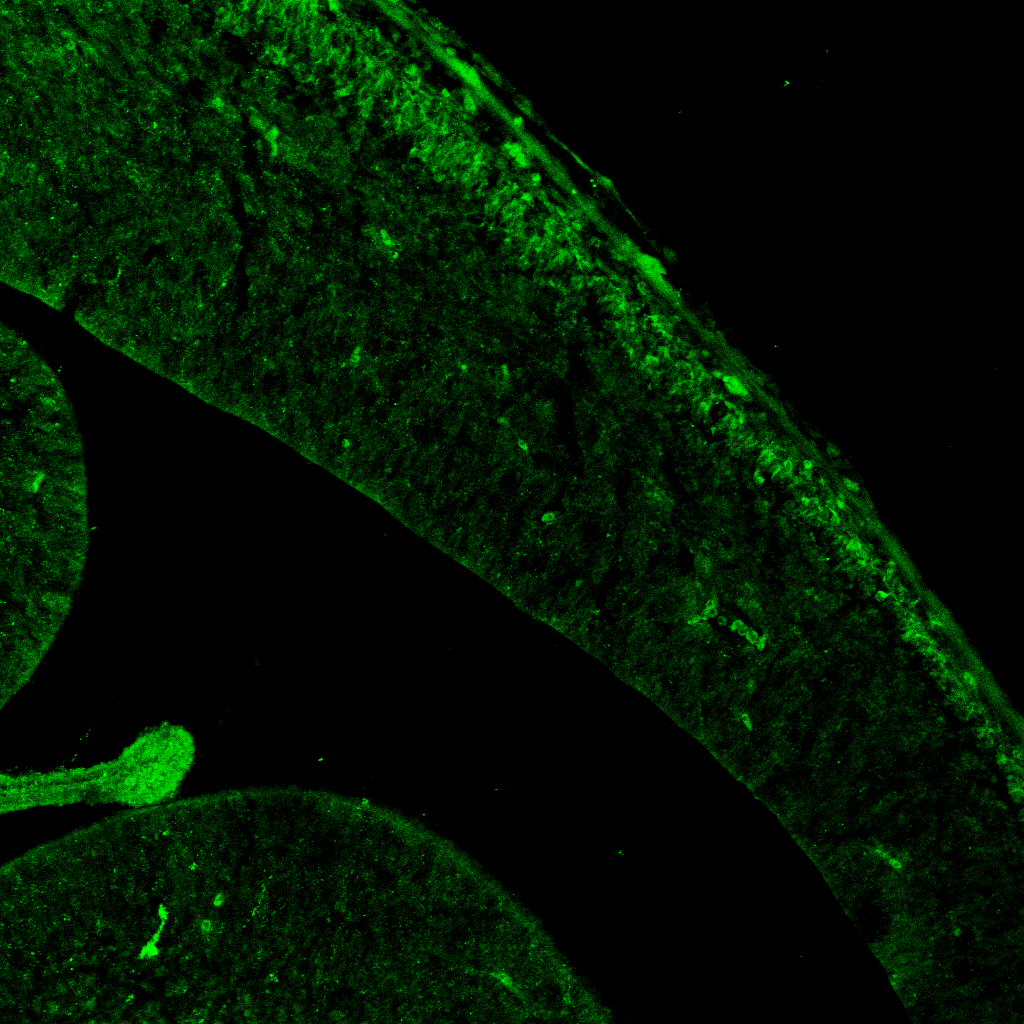

Supplement: Supplementary file 4 — Source data Fig. 1 [file 44321_2025_302_MOESM4_ESM.zip › Figure 1/1I/IF-mouse-MAP2.tif]

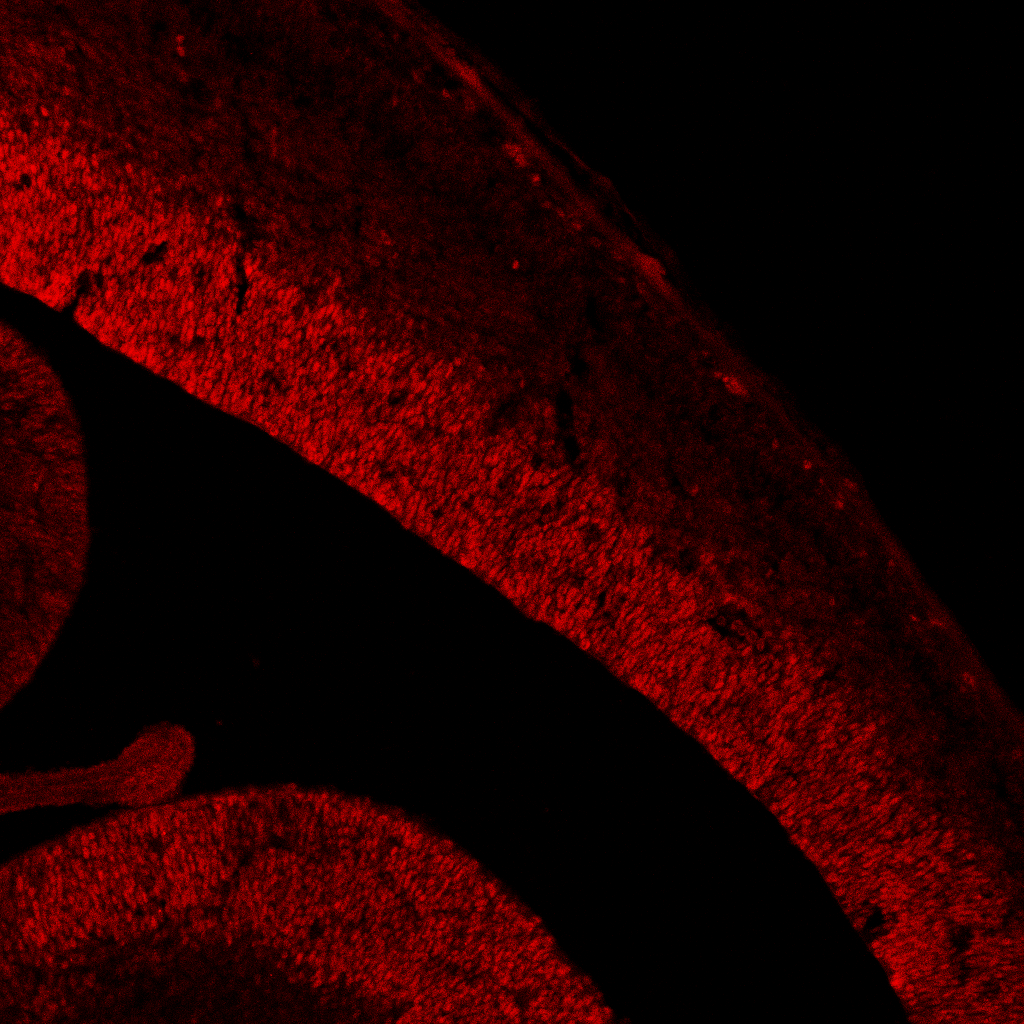

Supplement: Supplementary file 4 — Source data Fig. 1 [file 44321_2025_302_MOESM4_ESM.zip › Figure 1/1I/IF-mouse-PAX6.tif]

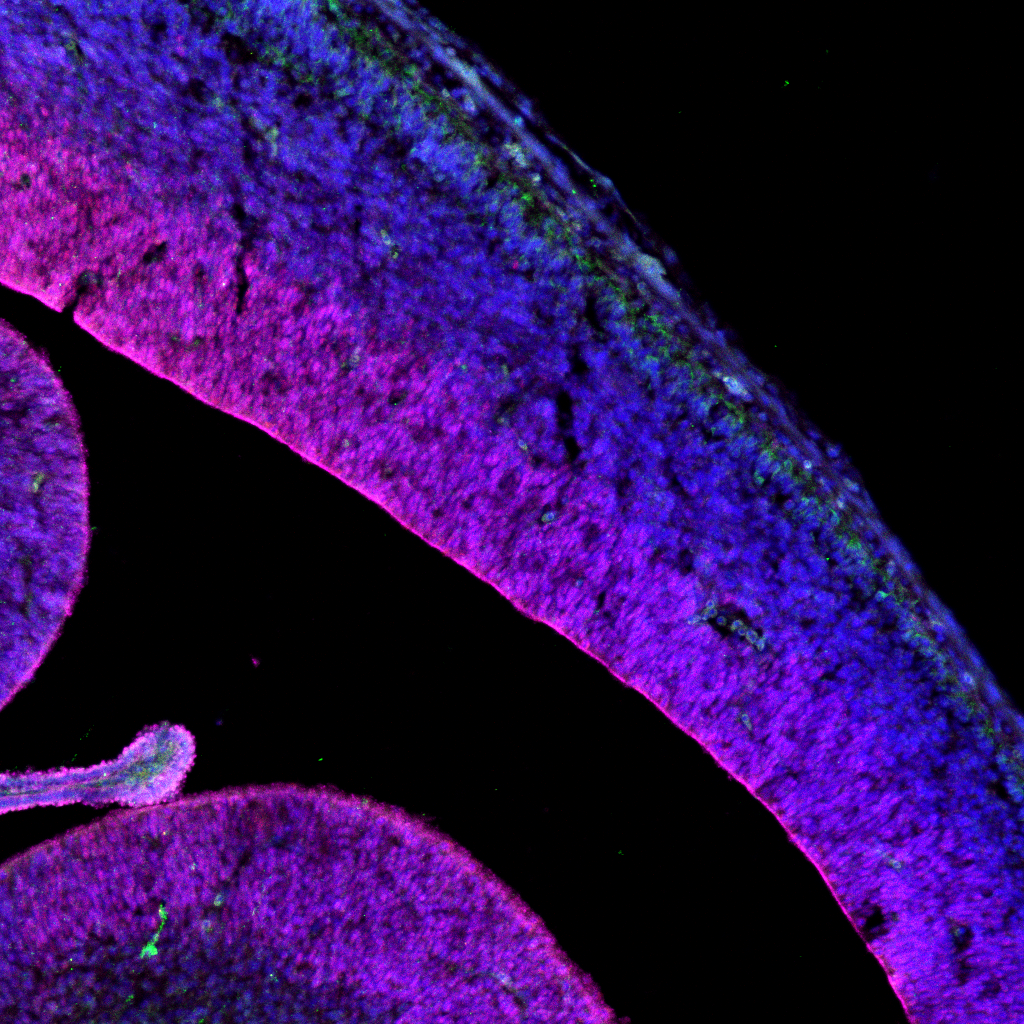

Supplement: Supplementary file 4 — Source data Fig. 1 [file 44321_2025_302_MOESM4_ESM.zip › Figure 1/1I/IF-mouse-merge.tif]

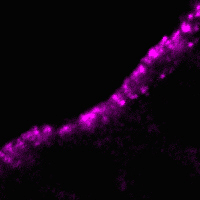

Supplement: Supplementary file 4 — Source data Fig. 1 [file 44321_2025_302_MOESM4_ESM.zip › Figure 1/1J/IF-CETN3-lower.tif]

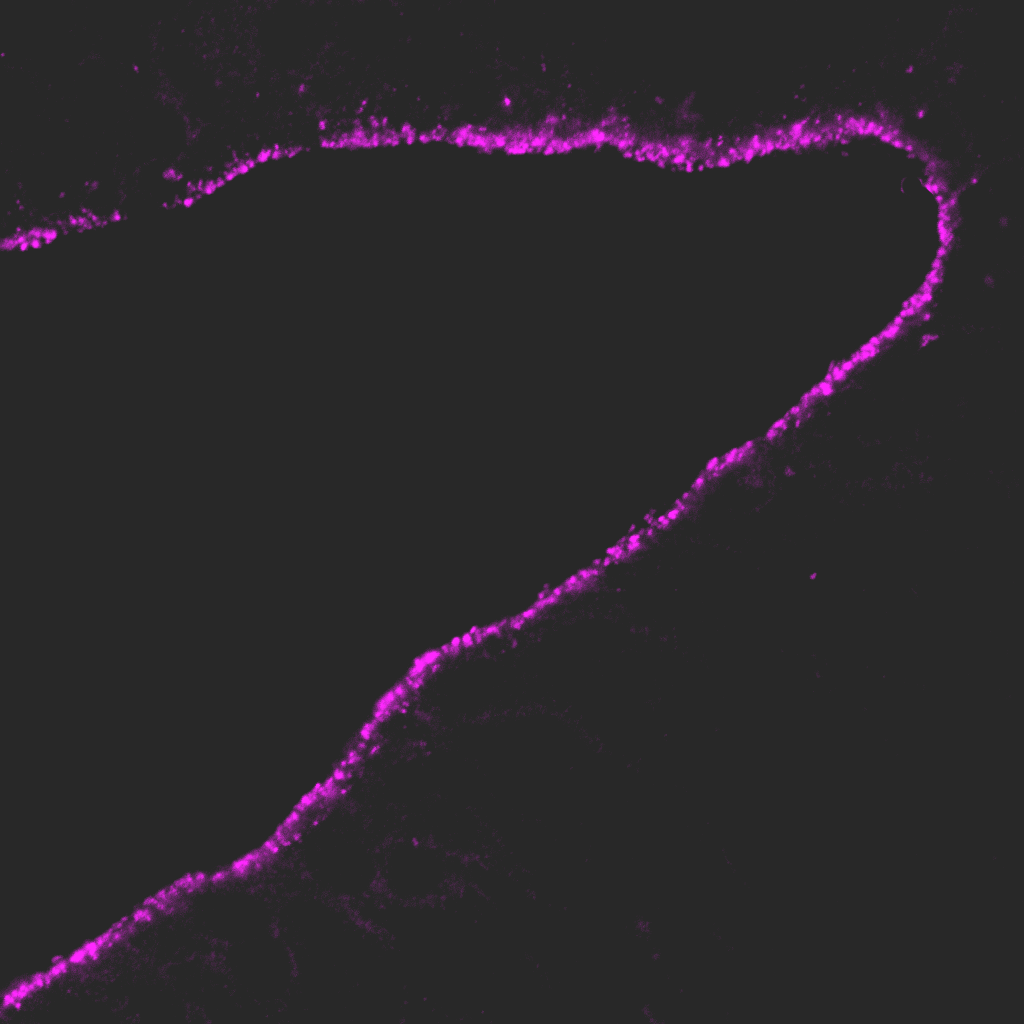

Supplement: Supplementary file 4 — Source data Fig. 1 [file 44321_2025_302_MOESM4_ESM.zip › Figure 1/1J/IF-CETN3-upper.tif]

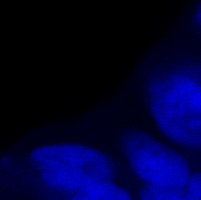

Supplement: Supplementary file 4 — Source data Fig. 1 [file 44321_2025_302_MOESM4_ESM.zip › Figure 1/1J/IF-DAPI-lower.jpg]

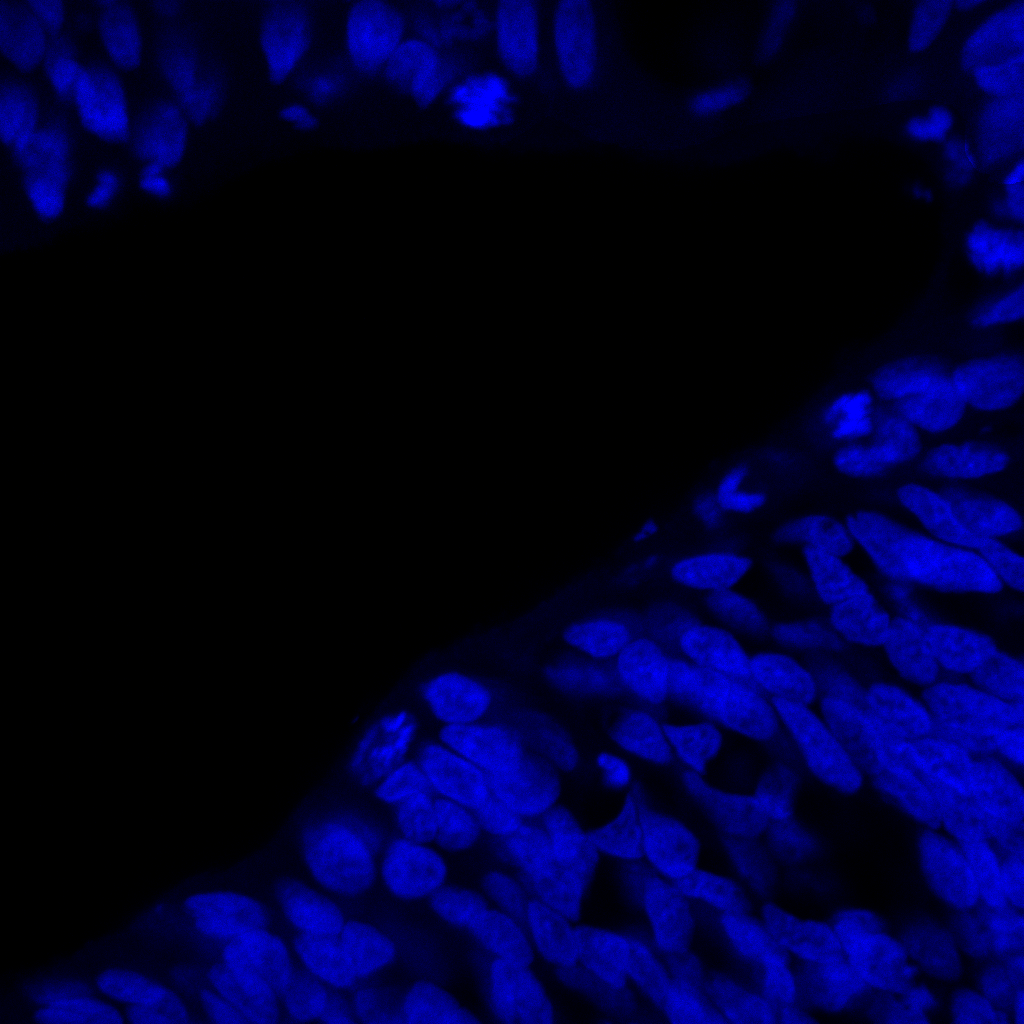

Supplement: Supplementary file 4 — Source data Fig. 1 [file 44321_2025_302_MOESM4_ESM.zip › Figure 1/1J/IF-DAPI-upper.tif]

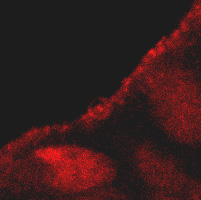

Supplement: Supplementary file 4 — Source data Fig. 1 [file 44321_2025_302_MOESM4_ESM.zip › Figure 1/1J/IF-PAX6-lower.jpg]

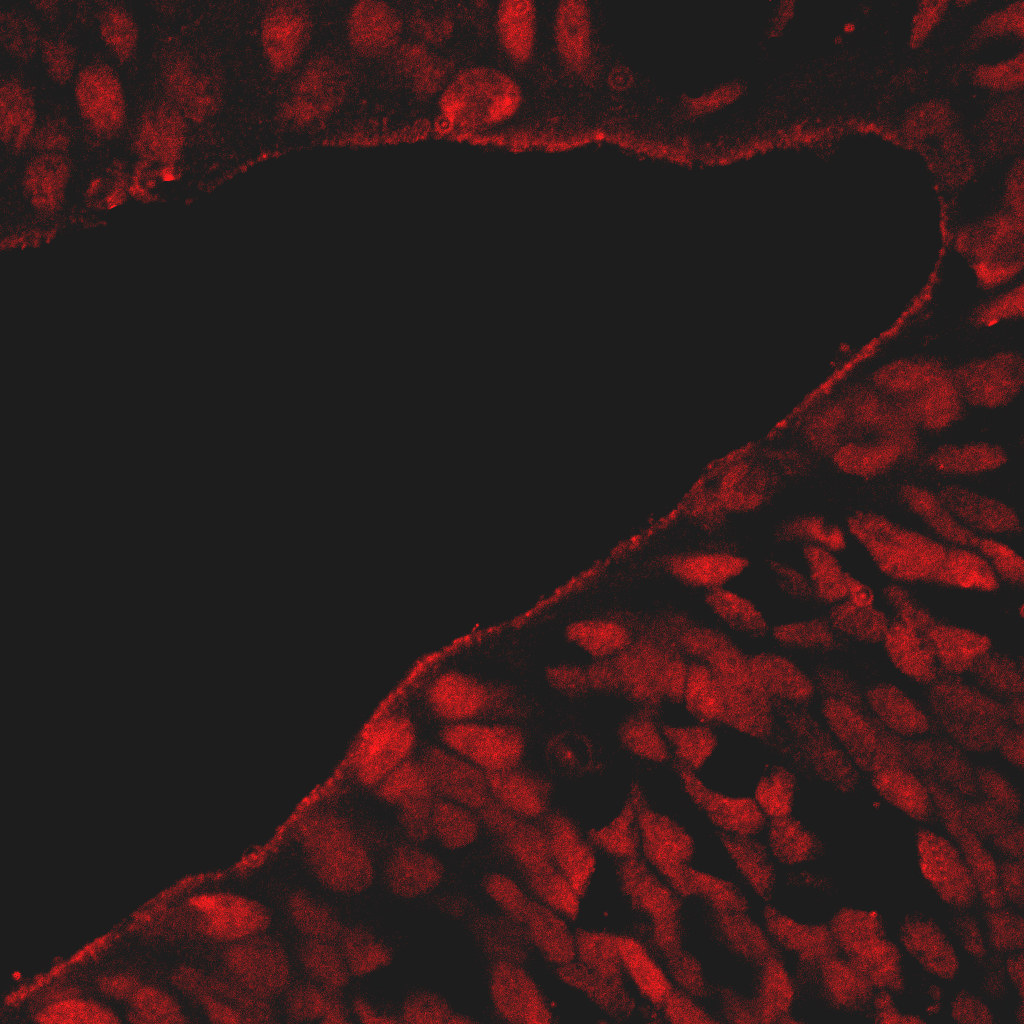

Supplement: Supplementary file 4 — Source data Fig. 1 [file 44321_2025_302_MOESM4_ESM.zip › Figure 1/1J/IF-PAX6-upper.tif]

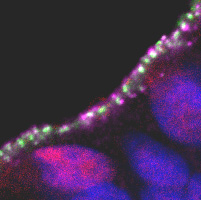

Supplement: Supplementary file 4 — Source data Fig. 1 [file 44321_2025_302_MOESM4_ESM.zip › Figure 1/1J/IF-merge-lower.jpg]

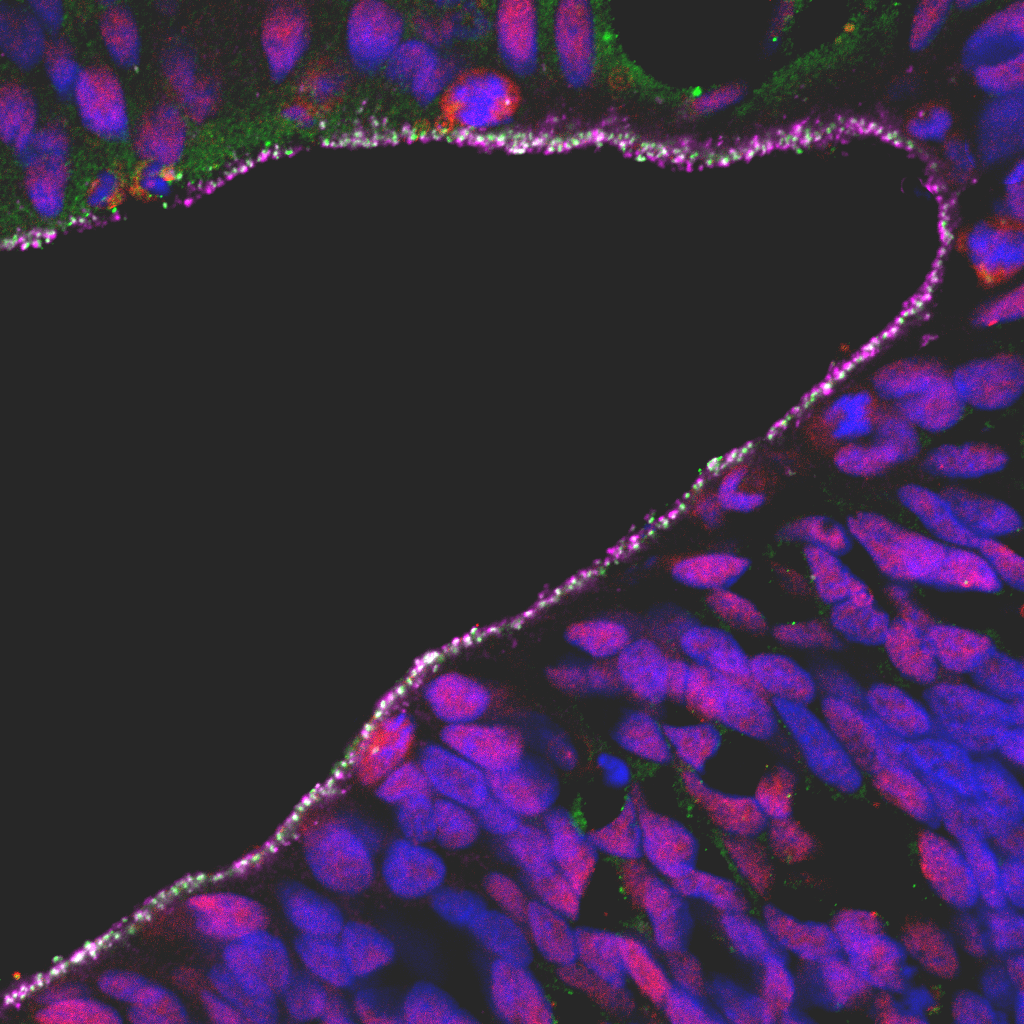

Supplement: Supplementary file 4 — Source data Fig. 1 [file 44321_2025_302_MOESM4_ESM.zip › Figure 1/1J/IF-merge-upper.tif]

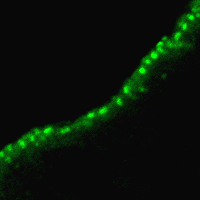

Supplement: Supplementary file 4 — Source data Fig. 1 [file 44321_2025_302_MOESM4_ESM.zip › Figure 1/1J/IF-r-TUBULIN-lower.tif]

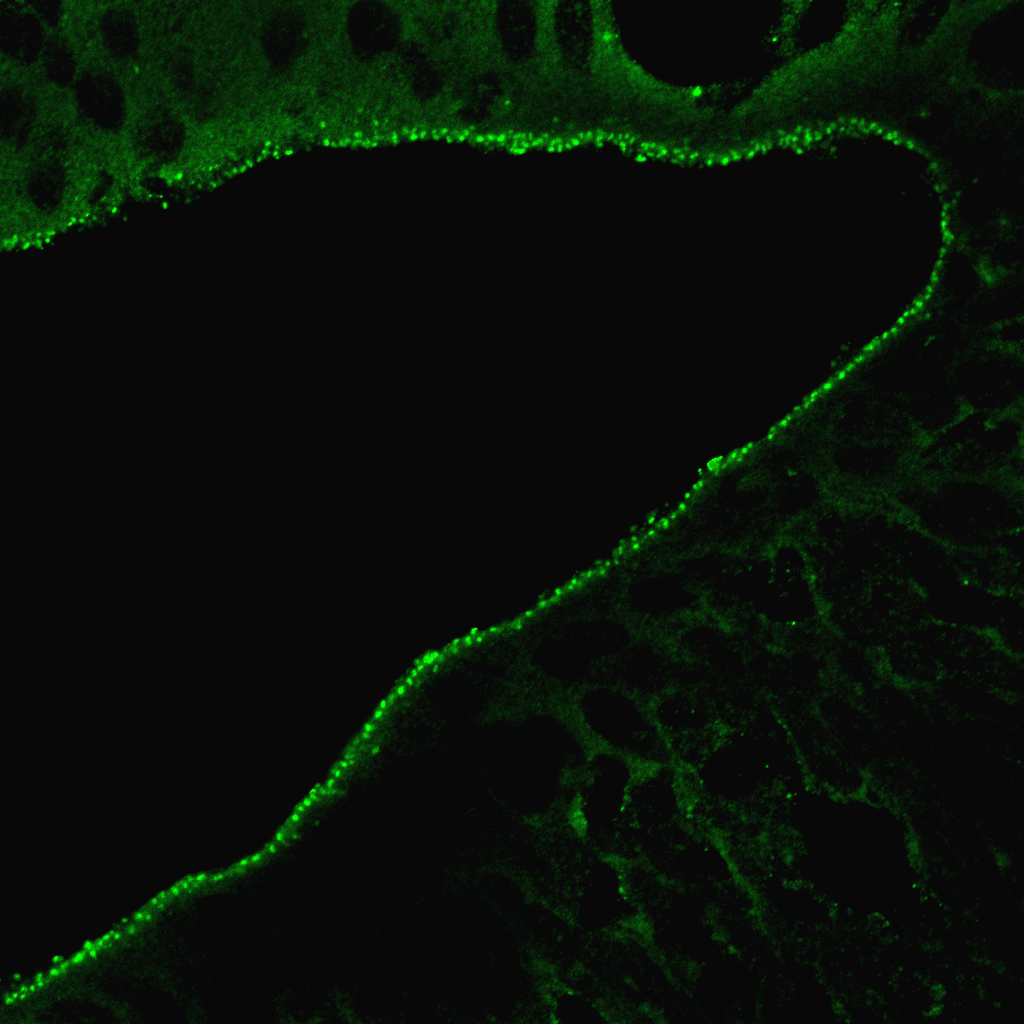

Supplement: Supplementary file 4 — Source data Fig. 1 [file 44321_2025_302_MOESM4_ESM.zip › Figure 1/1J/IF-r-TUBULIN-upper.tif]

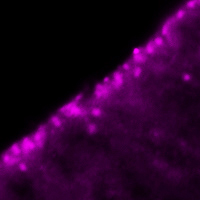

Supplement: Supplementary file 4 — Source data Fig. 1 [file 44321_2025_302_MOESM4_ESM.zip › Figure 1/1K/IF-CETN3-down.jpg]

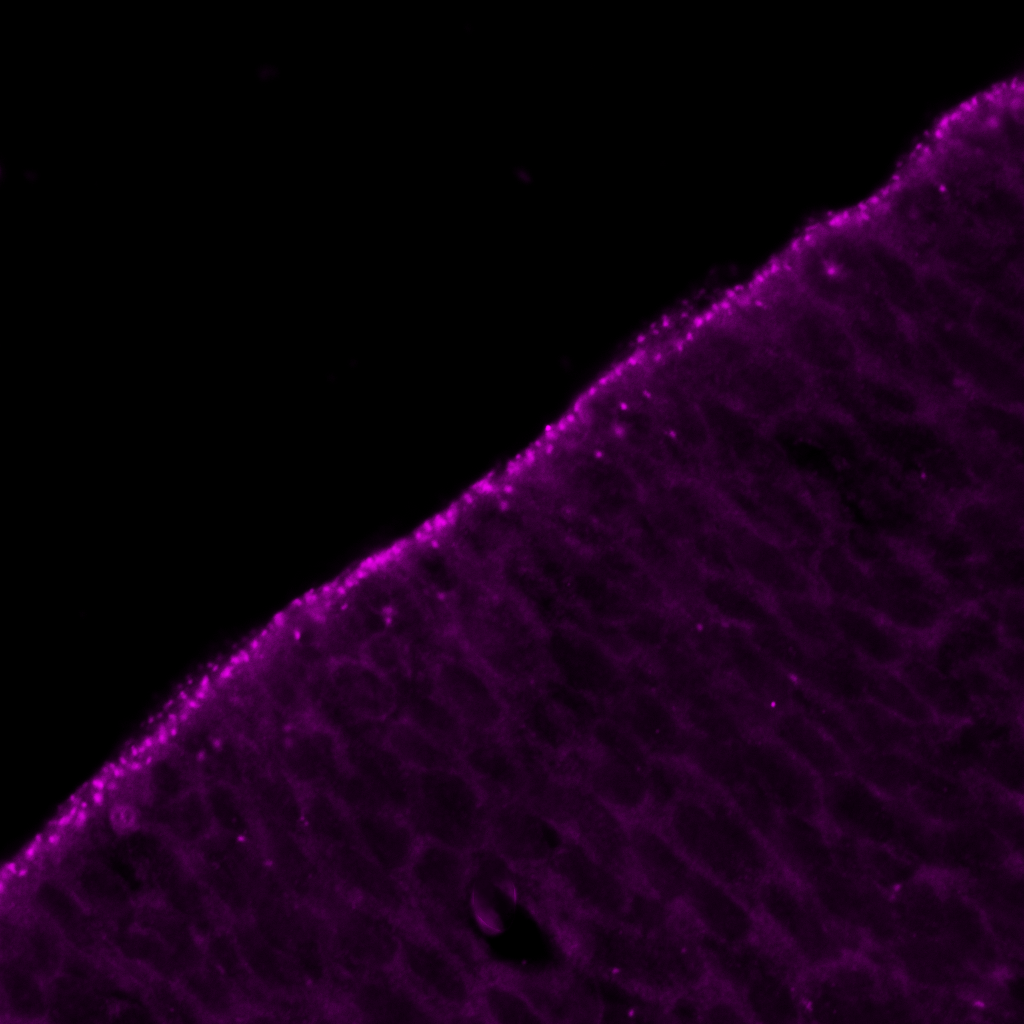

Supplement: Supplementary file 4 — Source data Fig. 1 [file 44321_2025_302_MOESM4_ESM.zip › Figure 1/1K/IF-CETN3-up.tif]

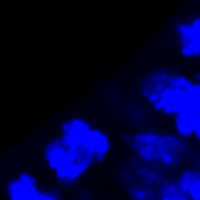

Supplement: Supplementary file 4 — Source data Fig. 1 [file 44321_2025_302_MOESM4_ESM.zip › Figure 1/1K/IF-DAPI-down.jpg]

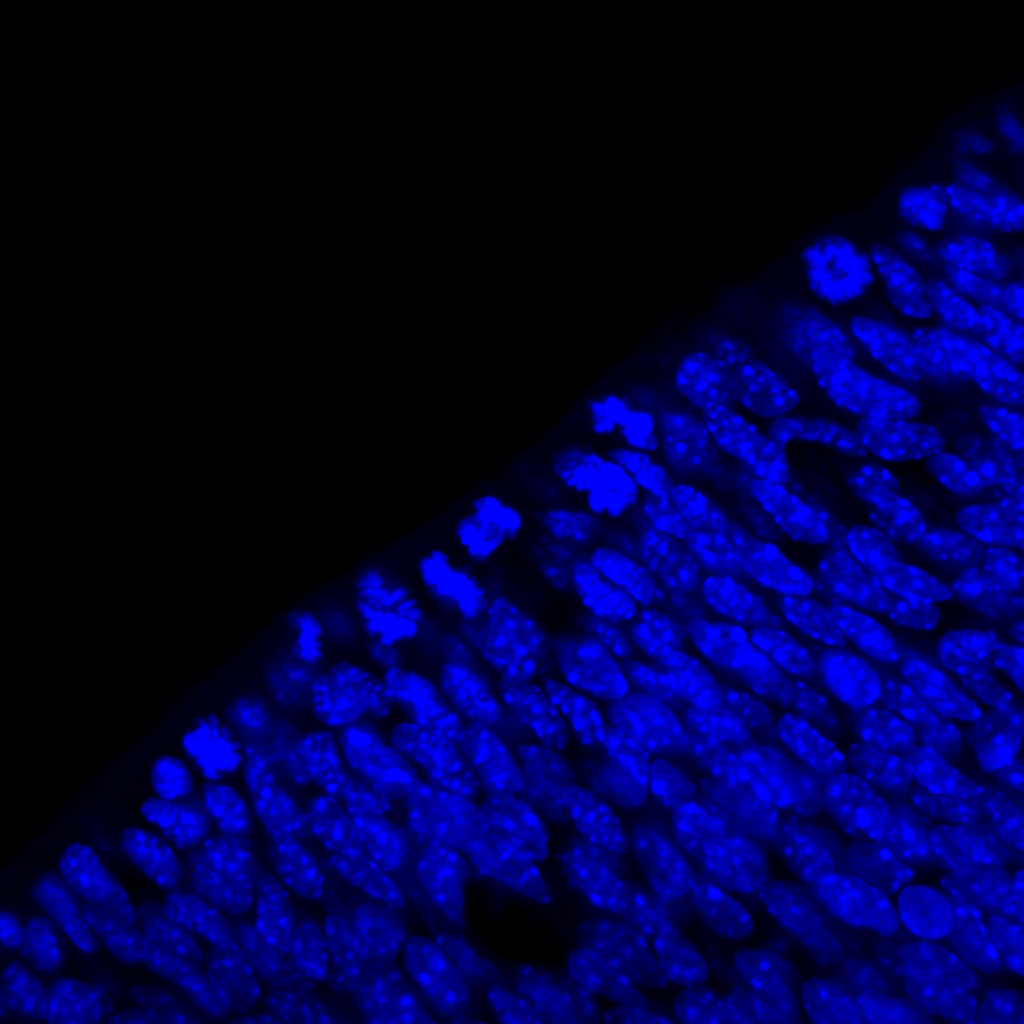

Supplement: Supplementary file 4 — Source data Fig. 1 [file 44321_2025_302_MOESM4_ESM.zip › Figure 1/1K/IF-DAPI-up.tif]

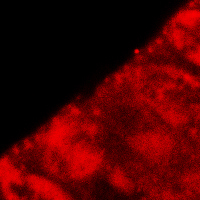

Supplement: Supplementary file 4 — Source data Fig. 1 [file 44321_2025_302_MOESM4_ESM.zip › Figure 1/1K/IF-PAX6-down.jpg]

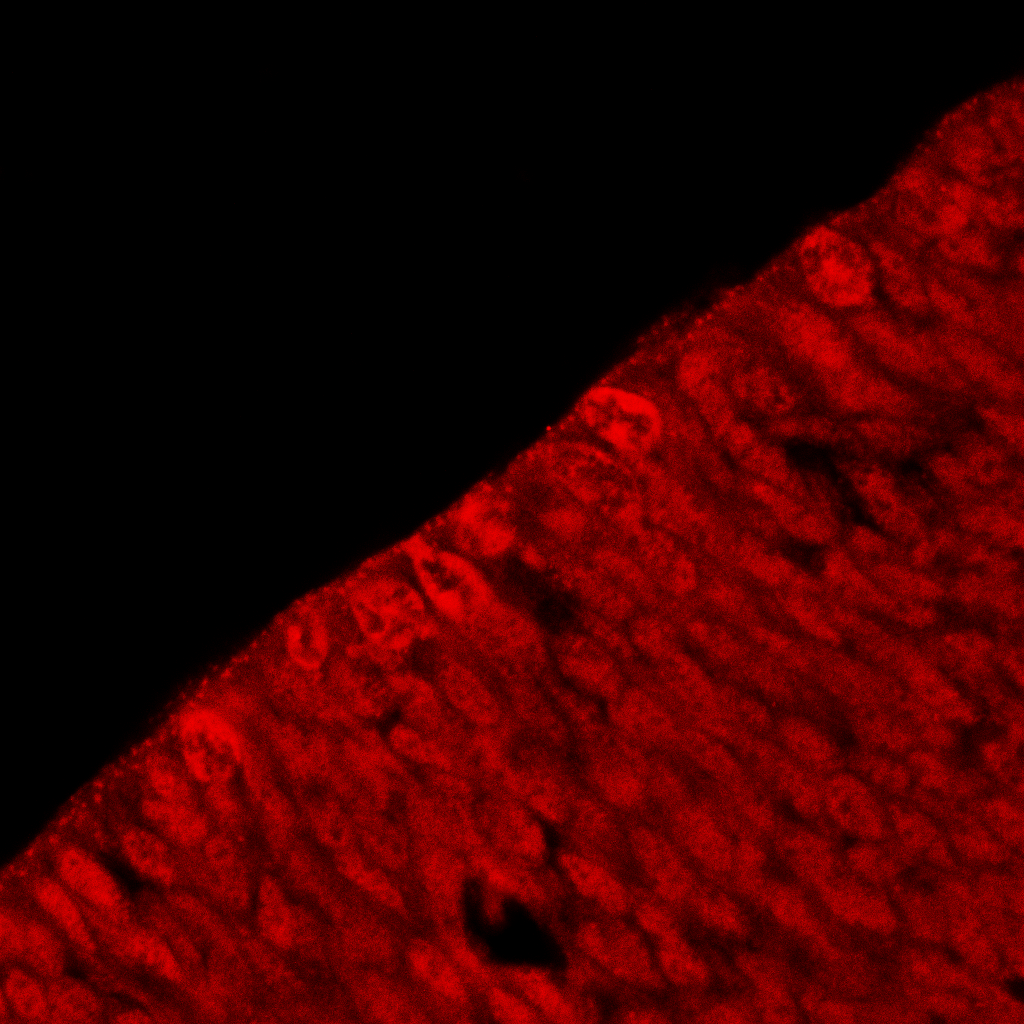

Supplement: Supplementary file 4 — Source data Fig. 1 [file 44321_2025_302_MOESM4_ESM.zip › Figure 1/1K/IF-PAX6-up.tif]

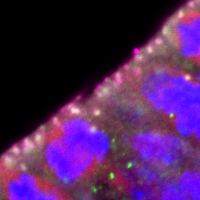

Supplement: Supplementary file 4 — Source data Fig. 1 [file 44321_2025_302_MOESM4_ESM.zip › Figure 1/1K/IF-merge-down.jpg]

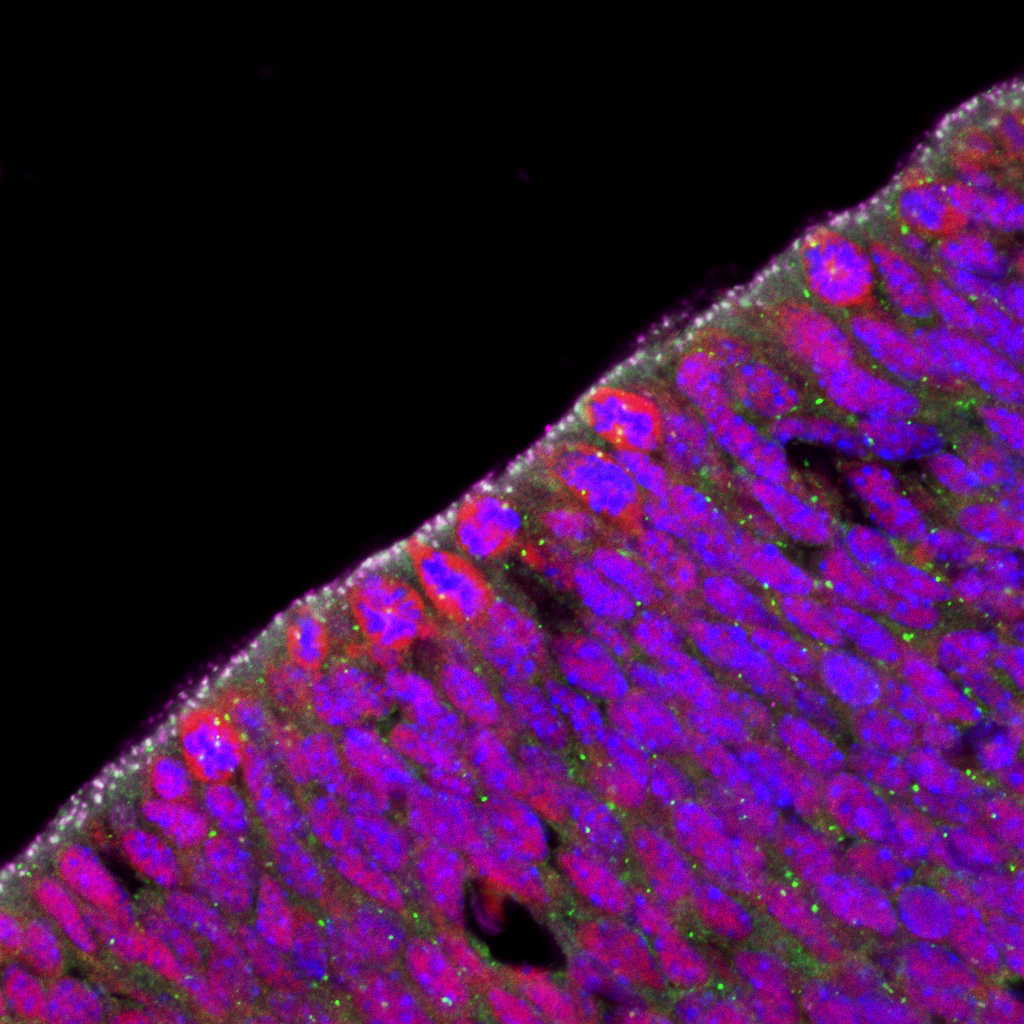

Supplement: Supplementary file 4 — Source data Fig. 1 [file 44321_2025_302_MOESM4_ESM.zip › Figure 1/1K/IF-merge-up.tif]

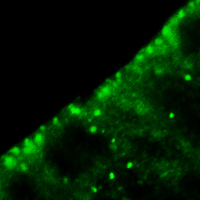

Supplement: Supplementary file 4 — Source data Fig. 1 [file 44321_2025_302_MOESM4_ESM.zip › Figure 1/1K/IF-r-TUBULIN-down.jpg]

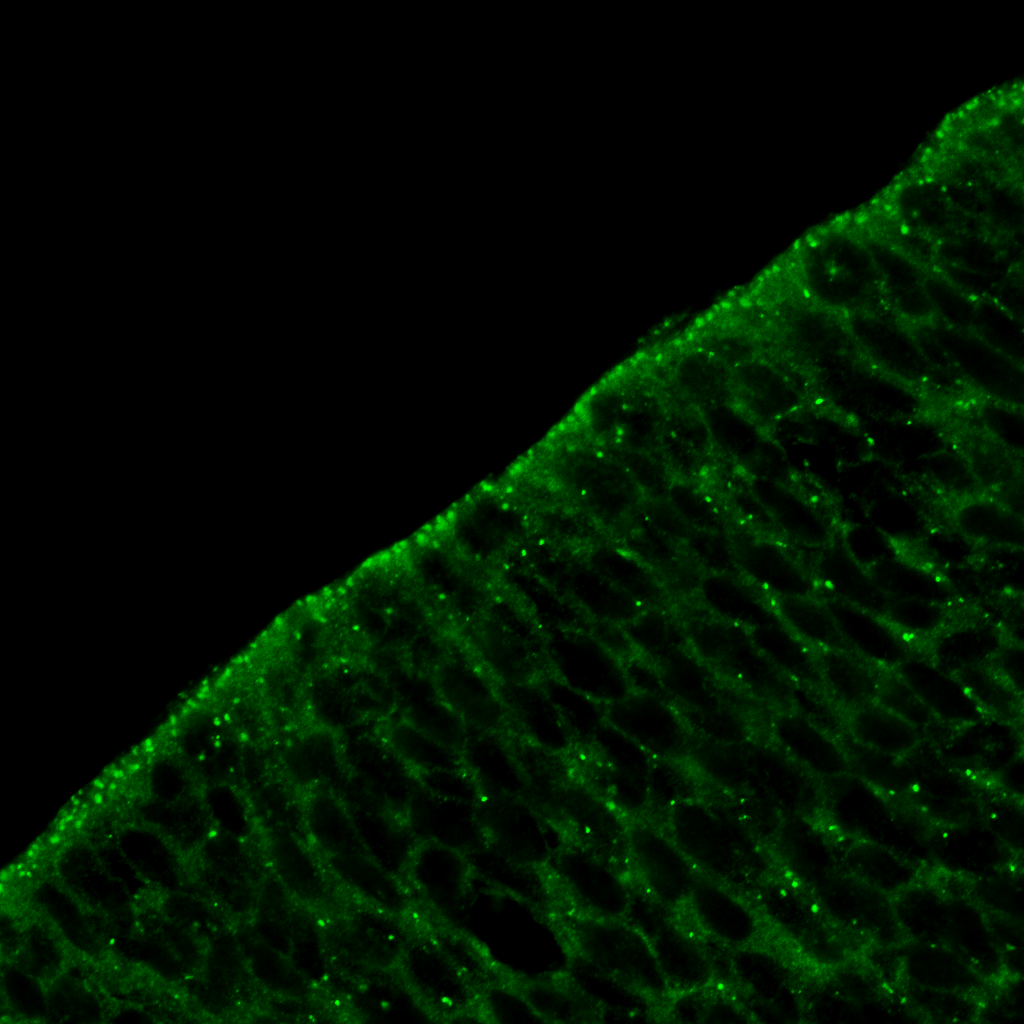

Supplement: Supplementary file 4 — Source data Fig. 1 [file 44321_2025_302_MOESM4_ESM.zip › Figure 1/1K/IF-r-TUBULIN-up.tif]

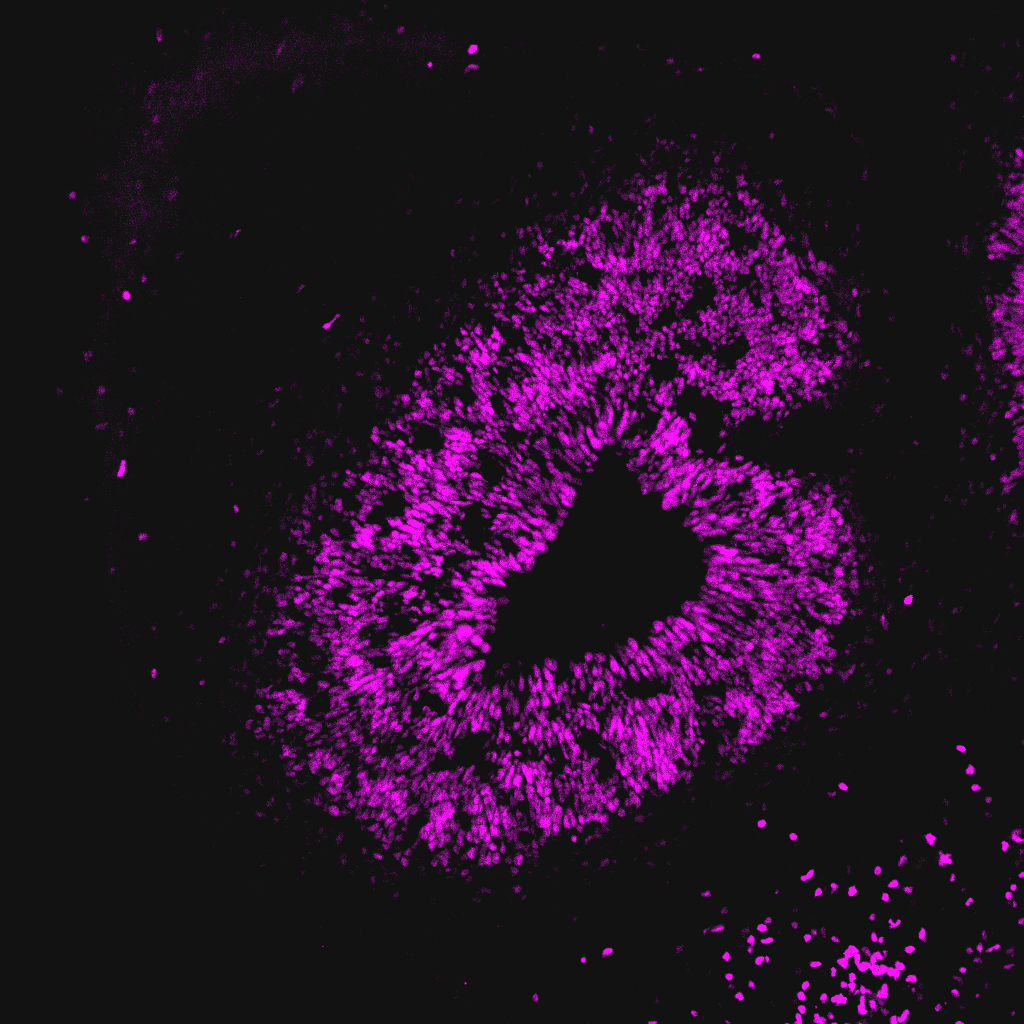

Supplement: Supplementary file 5 — Source data Fig. 4 [file 44321_2025_302_MOESM5_ESM.zip › Figure 4/4A/#12-3-PAX6.tif]

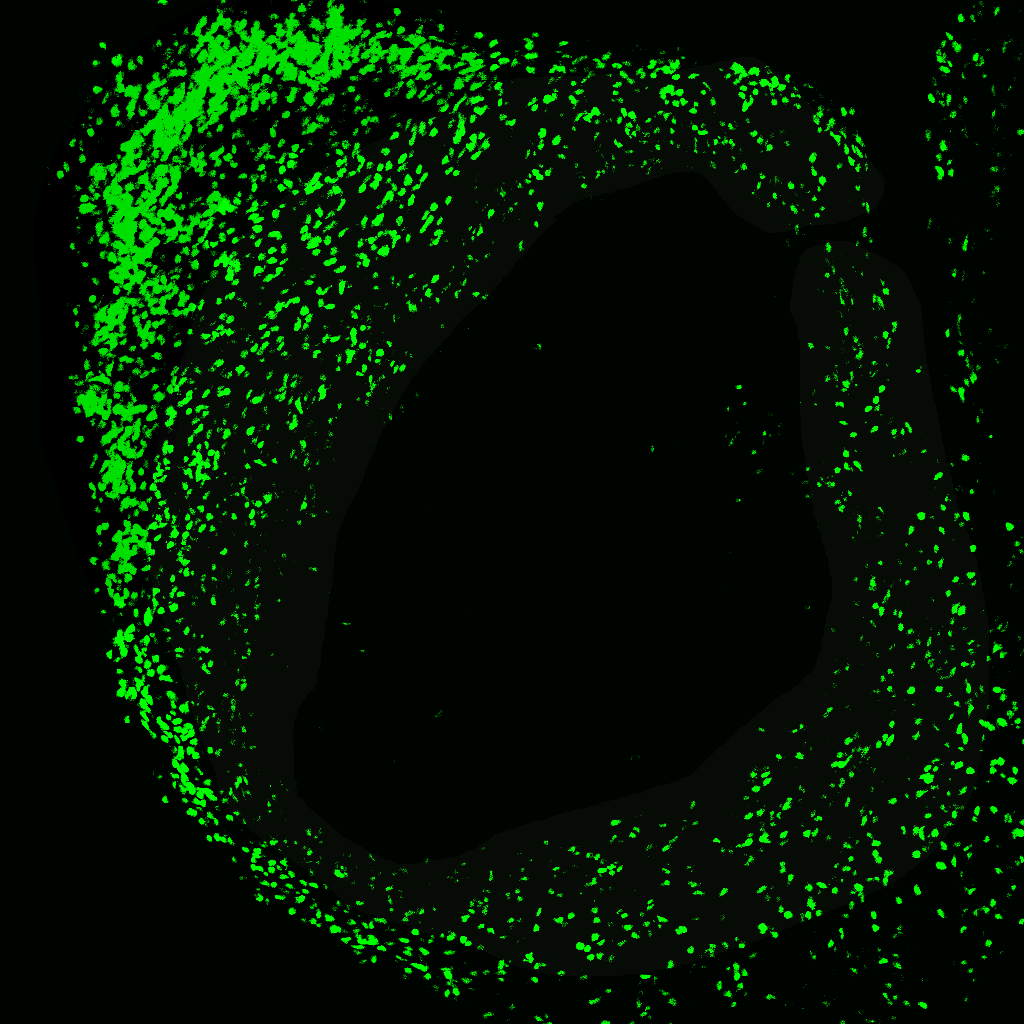

Supplement: Supplementary file 5 — Source data Fig. 4 [file 44321_2025_302_MOESM5_ESM.zip › Figure 4/4A/#12-3-TBR1.tif]

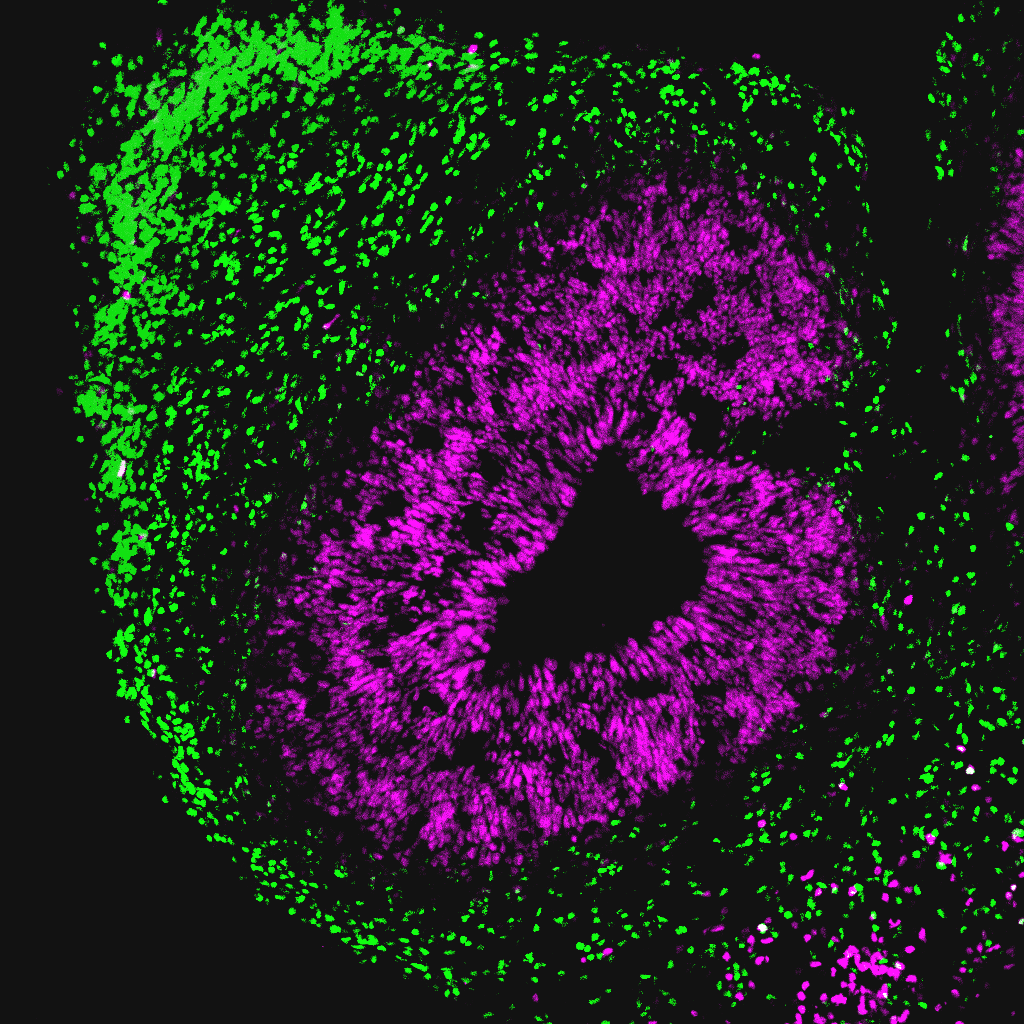

Supplement: Supplementary file 5 — Source data Fig. 4 [file 44321_2025_302_MOESM5_ESM.zip › Figure 4/4A/#12-3-merge.tif]

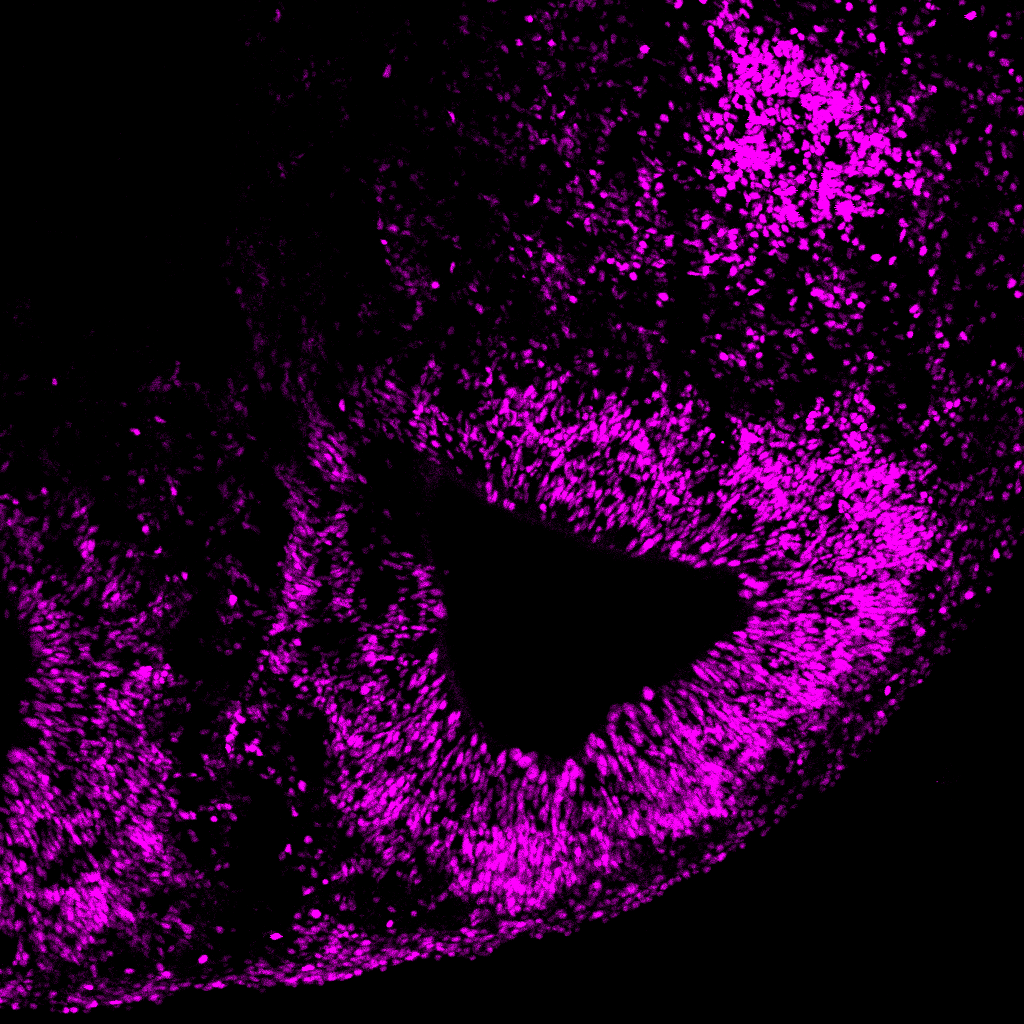

Supplement: Supplementary file 5 — Source data Fig. 4 [file 44321_2025_302_MOESM5_ESM.zip › Figure 4/4A/#7-5-PAX6.tif]

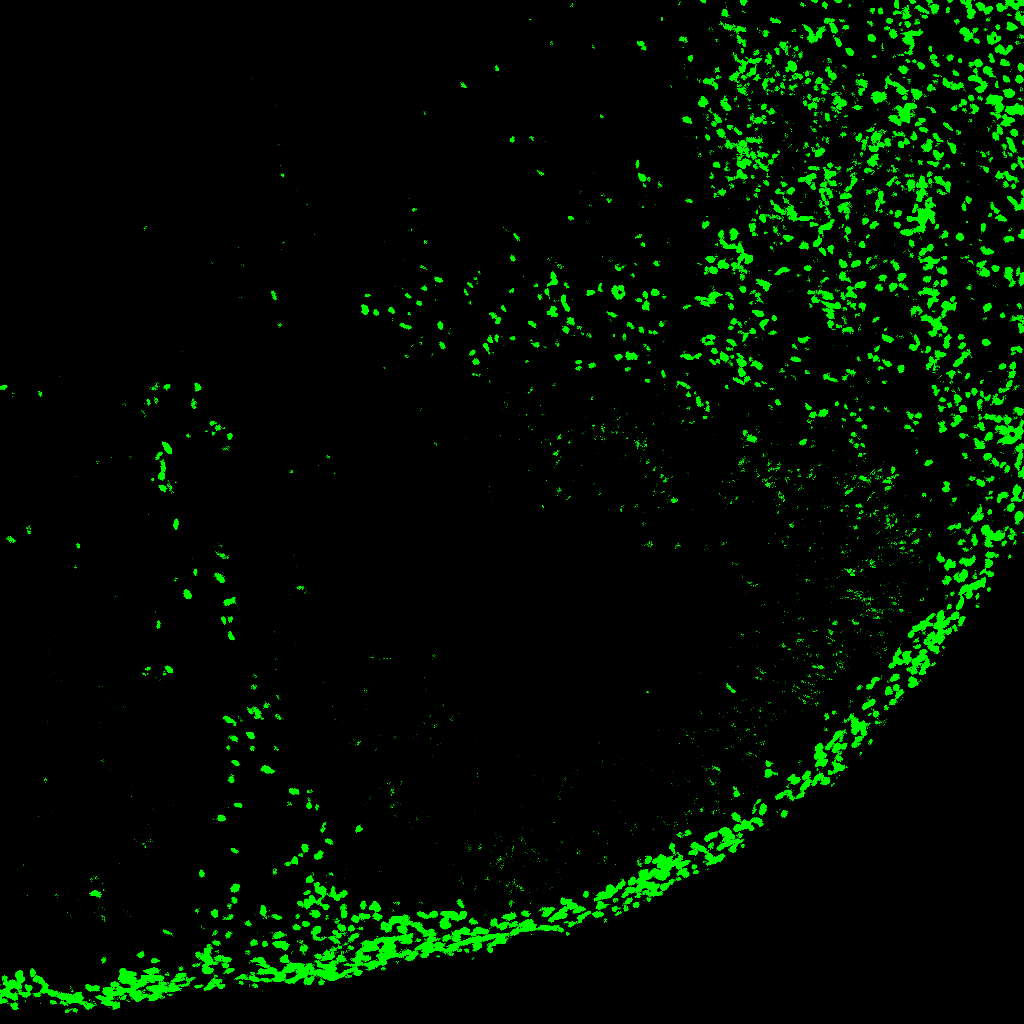

Supplement: Supplementary file 5 — Source data Fig. 4 [file 44321_2025_302_MOESM5_ESM.zip › Figure 4/4A/#7-5-TBR1.tif]

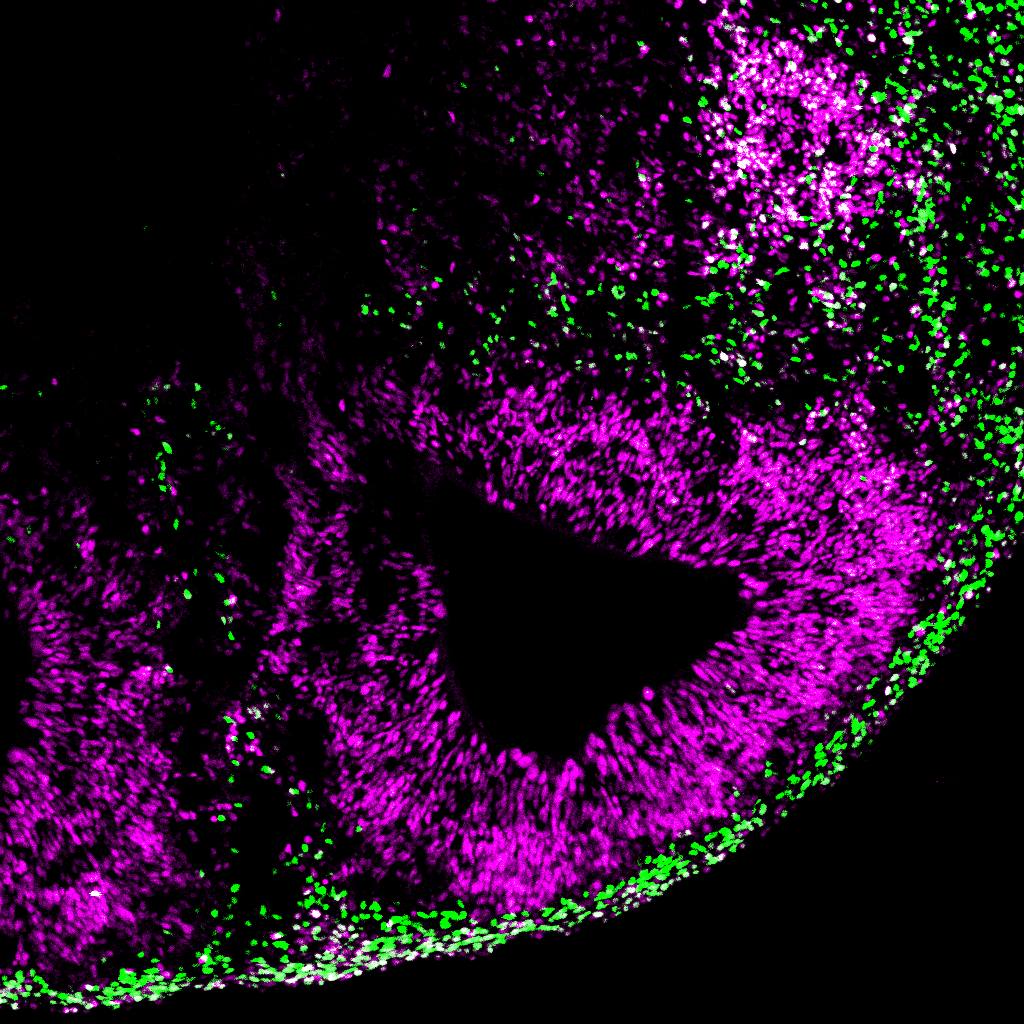

Supplement: Supplementary file 5 — Source data Fig. 4 [file 44321_2025_302_MOESM5_ESM.zip › Figure 4/4A/#7-5-merge.tif]

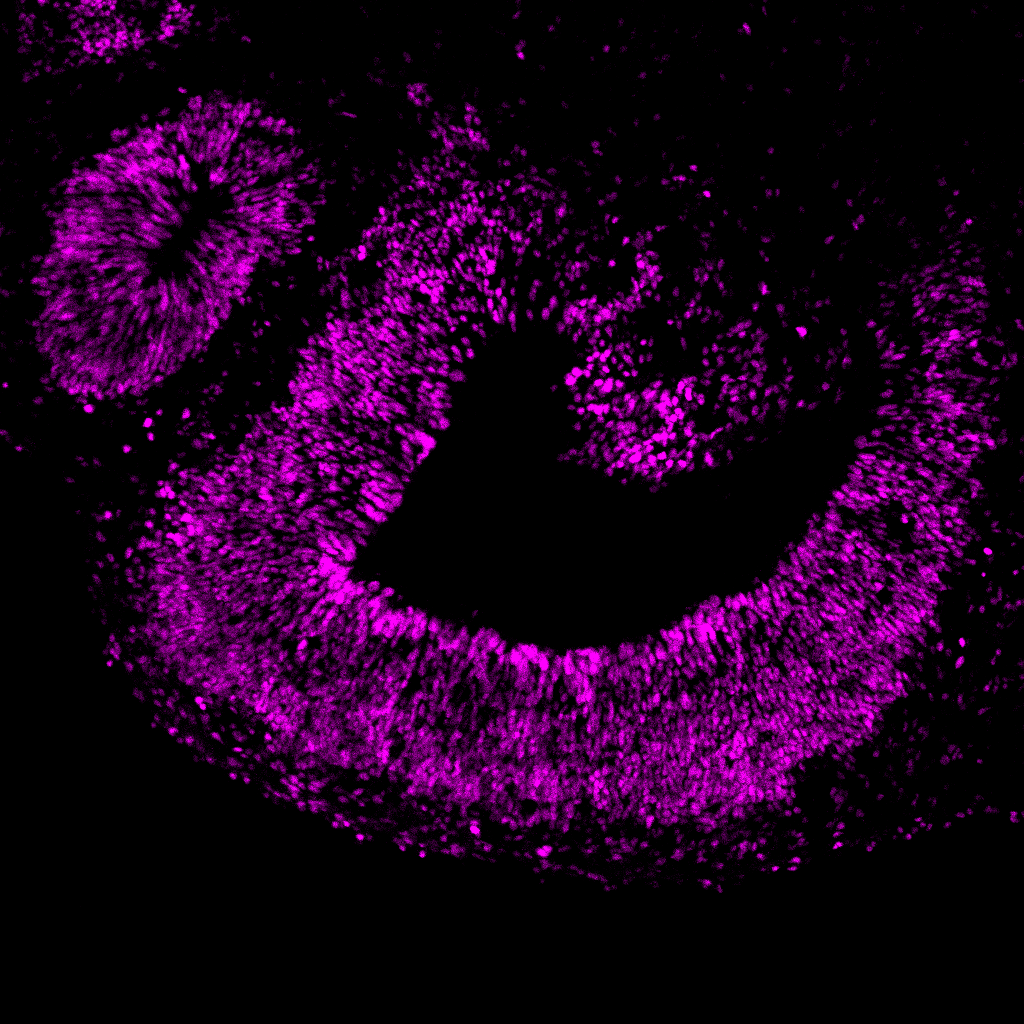

Supplement: Supplementary file 5 — Source data Fig. 4 [file 44321_2025_302_MOESM5_ESM.zip › Figure 4/4A/H9-PAX6.tif]

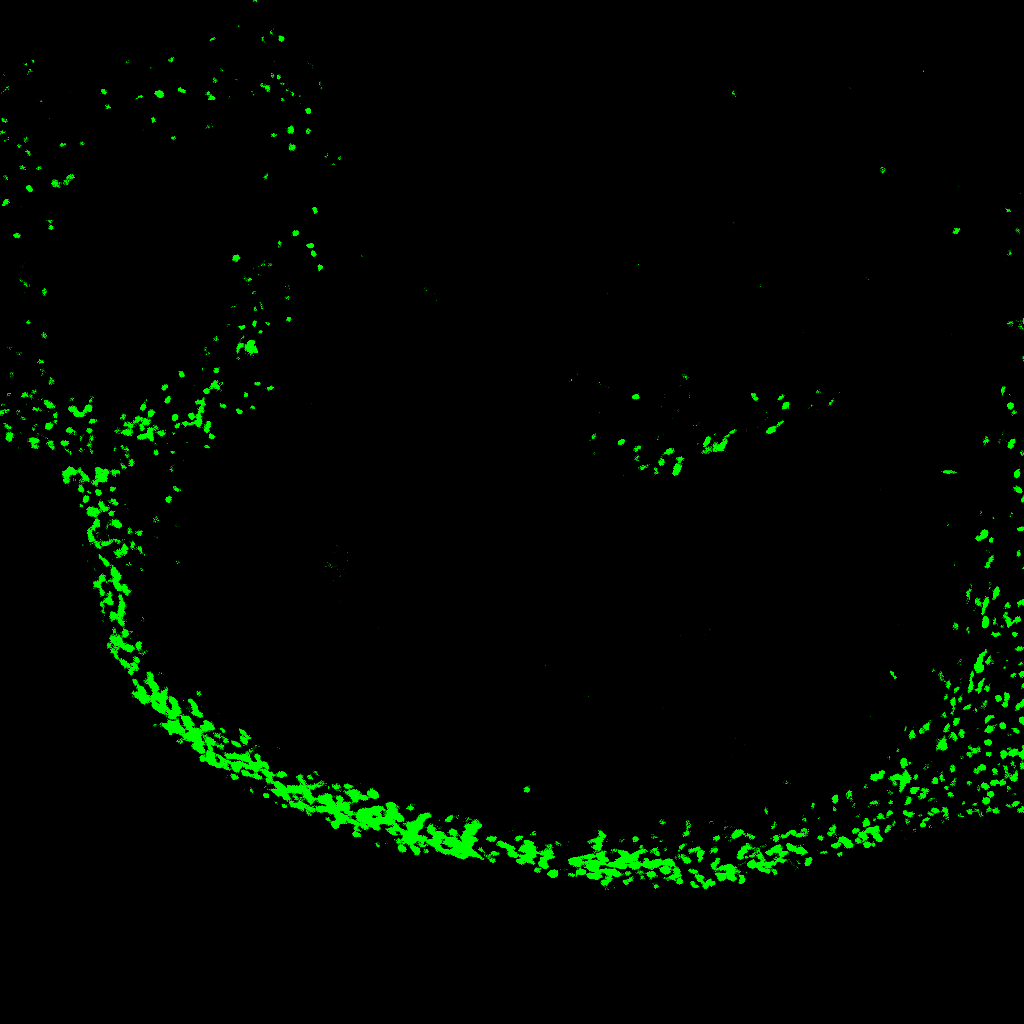

Supplement: Supplementary file 5 — Source data Fig. 4 [file 44321_2025_302_MOESM5_ESM.zip › Figure 4/4A/H9-TBR1.tif]

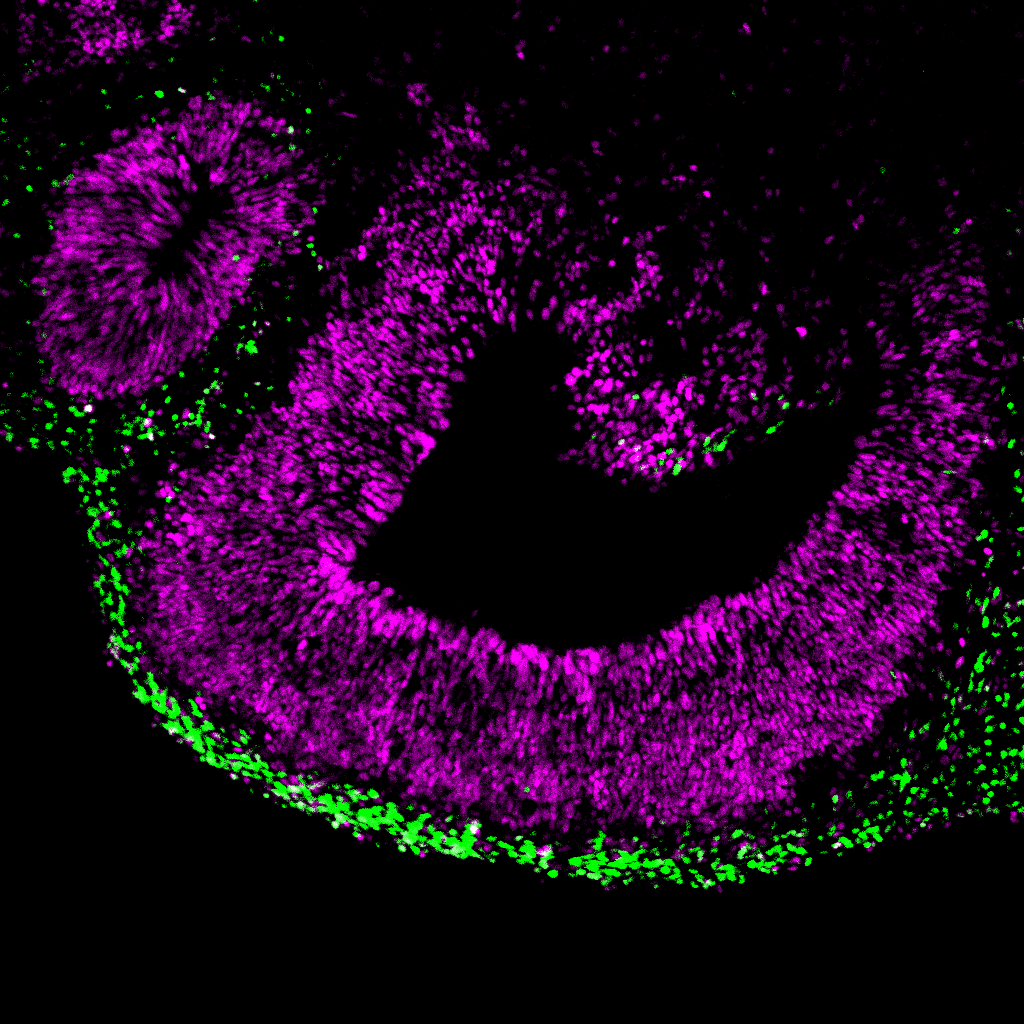

Supplement: Supplementary file 5 — Source data Fig. 4 [file 44321_2025_302_MOESM5_ESM.zip › Figure 4/4A/H9-merge.tif]

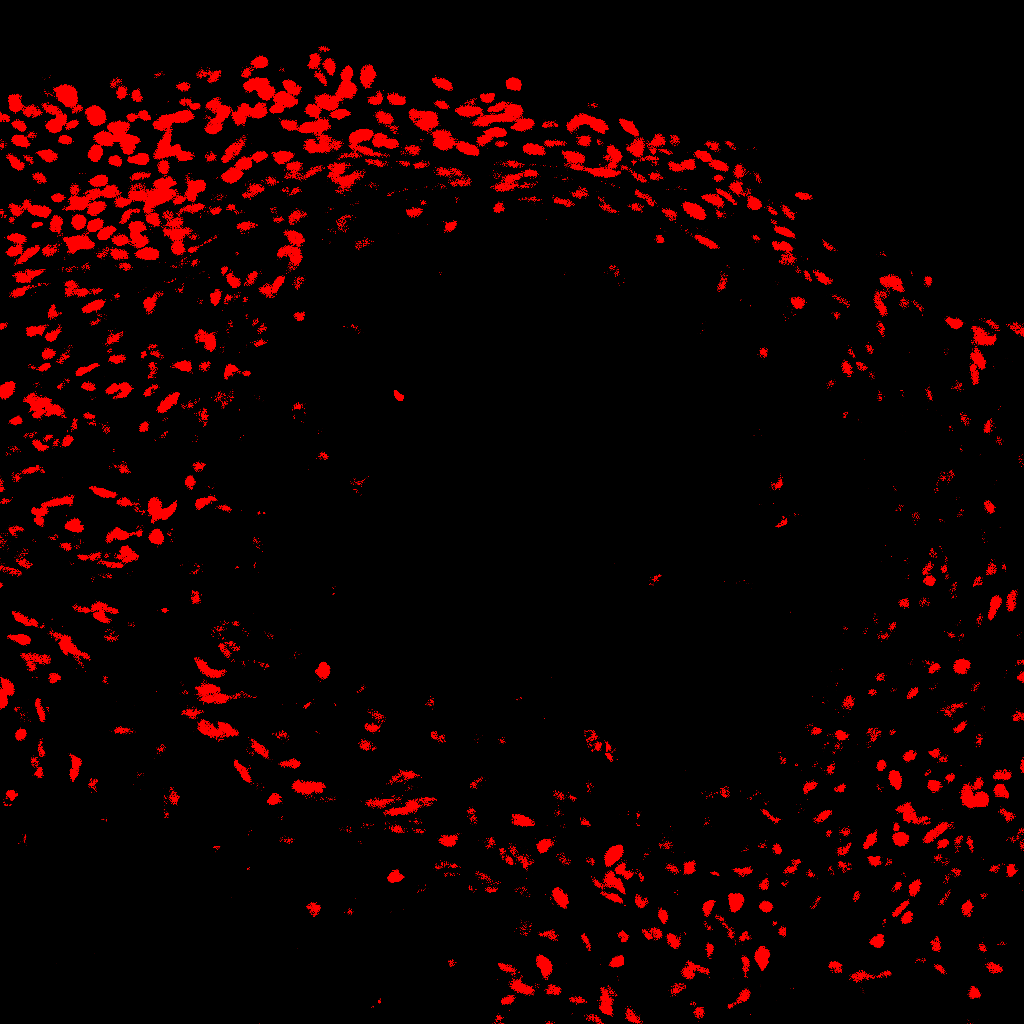

Supplement: Supplementary file 5 — Source data Fig. 4 [file 44321_2025_302_MOESM5_ESM.zip › Figure 4/4B/#12-3-CTIP2.tif]

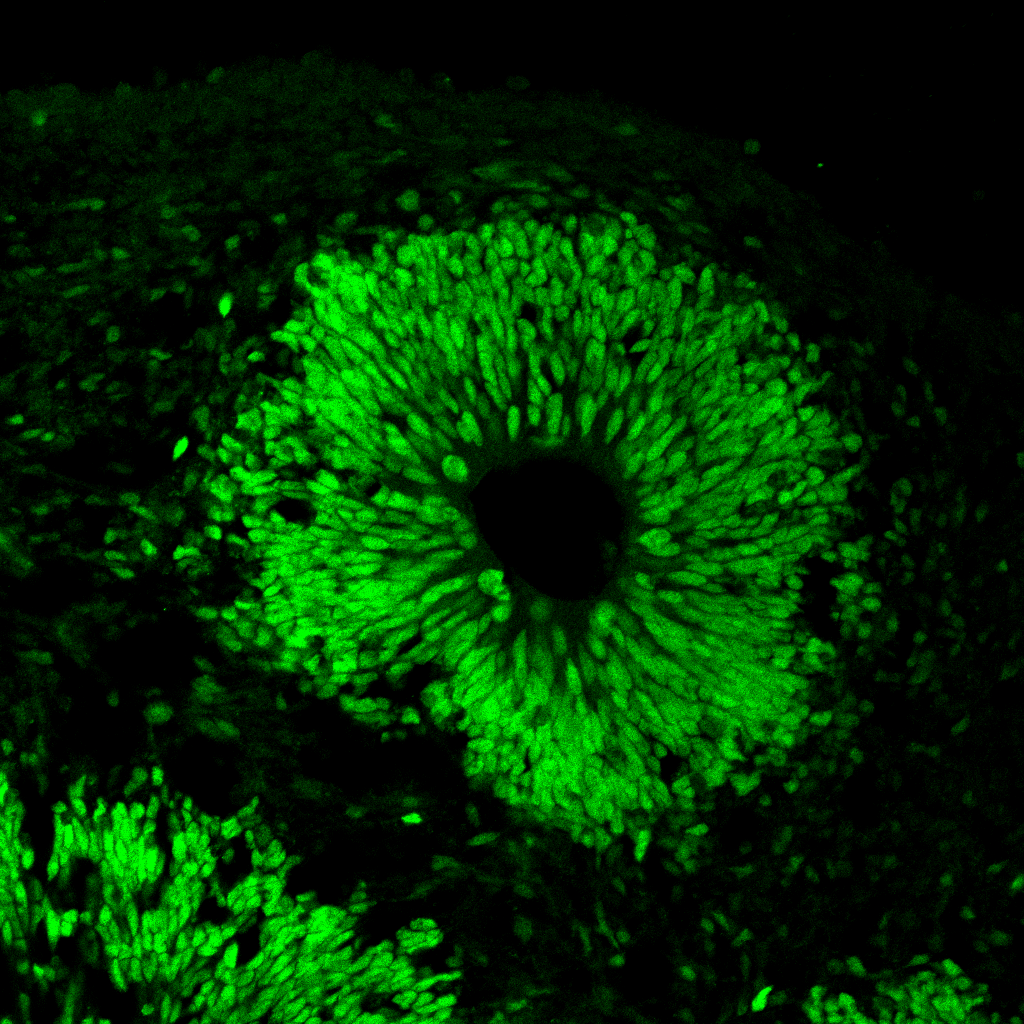

Supplement: Supplementary file 5 — Source data Fig. 4 [file 44321_2025_302_MOESM5_ESM.zip › Figure 4/4B/#12-3-PAX6.tif]

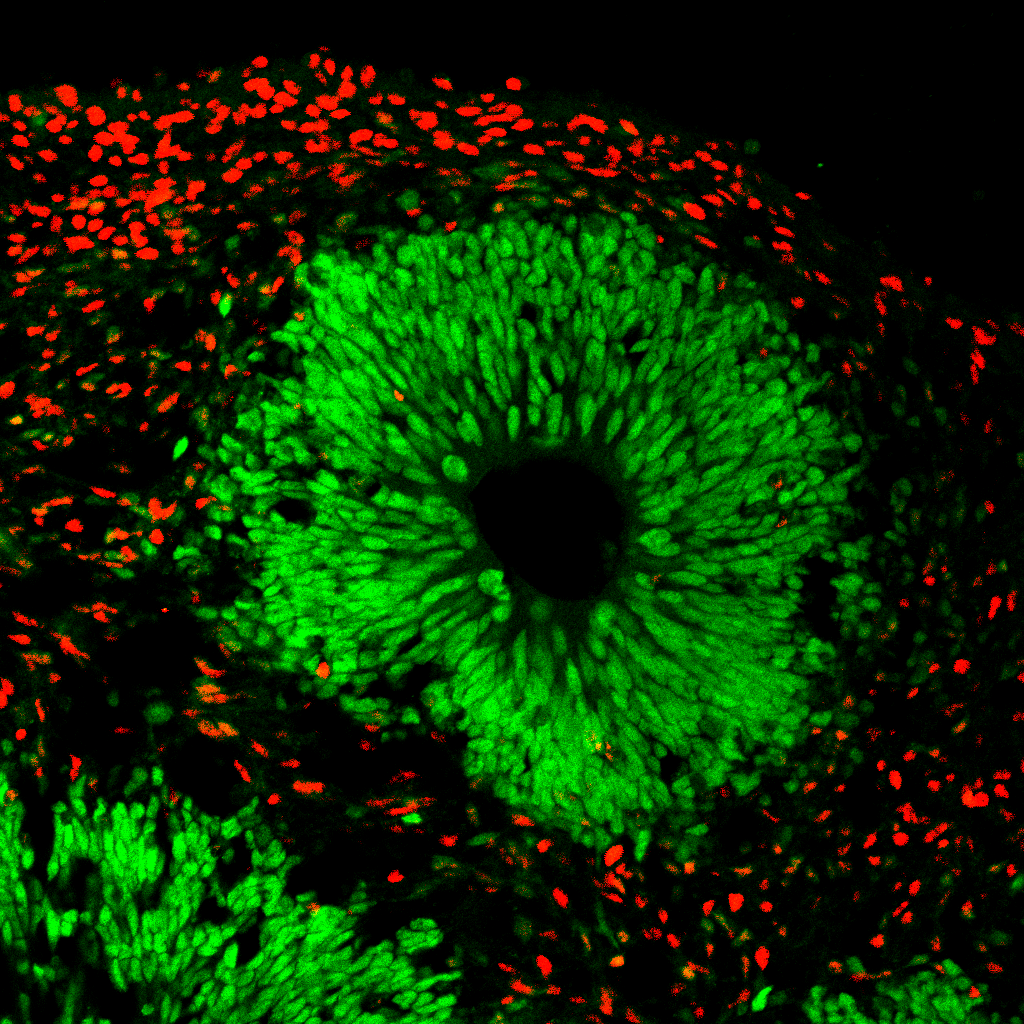

Supplement: Supplementary file 5 — Source data Fig. 4 [file 44321_2025_302_MOESM5_ESM.zip › Figure 4/4B/#12-3-merge.tif]

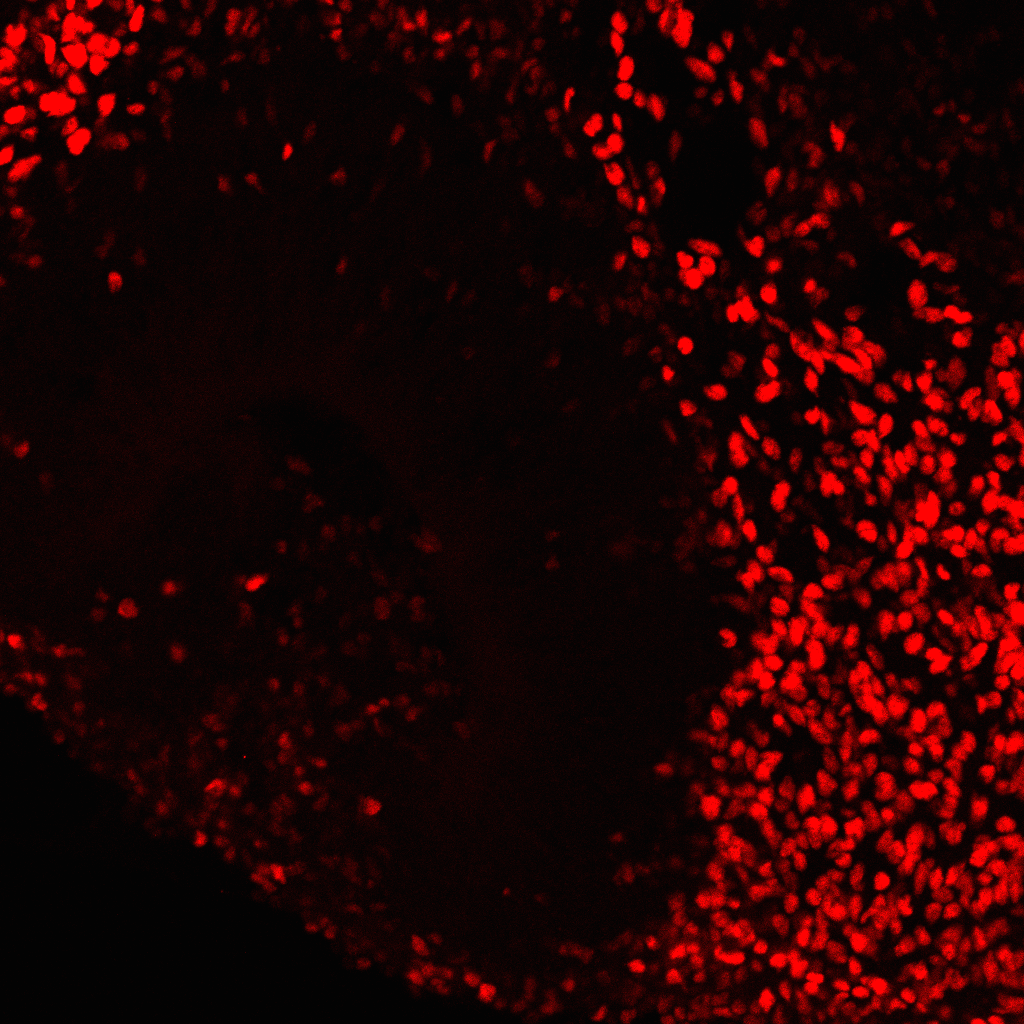

Supplement: Supplementary file 5 — Source data Fig. 4 [file 44321_2025_302_MOESM5_ESM.zip › Figure 4/4B/#7-5-CTIP2.tif]

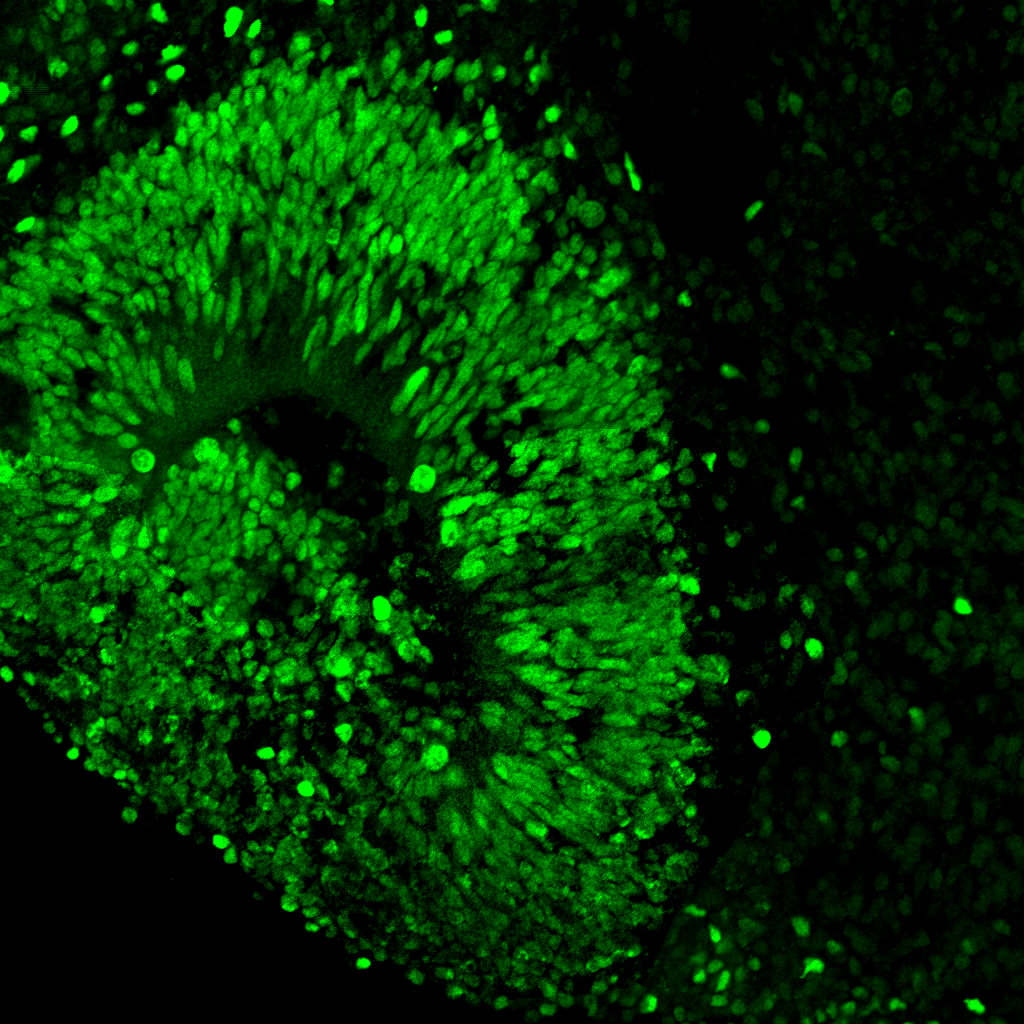

Supplement: Supplementary file 5 — Source data Fig. 4 [file 44321_2025_302_MOESM5_ESM.zip › Figure 4/4B/#7-5-PAX6.tif]

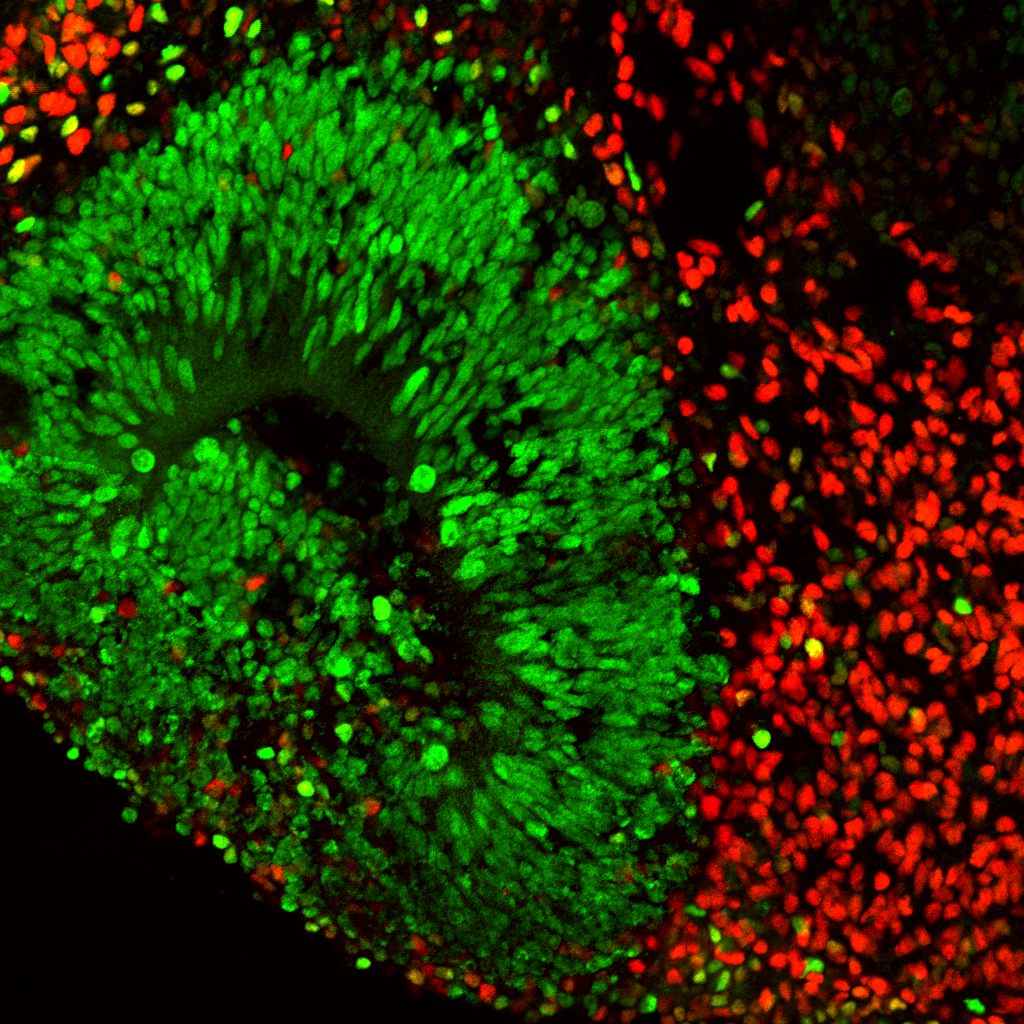

Supplement: Supplementary file 5 — Source data Fig. 4 [file 44321_2025_302_MOESM5_ESM.zip › Figure 4/4B/#7-5-merge.tif]

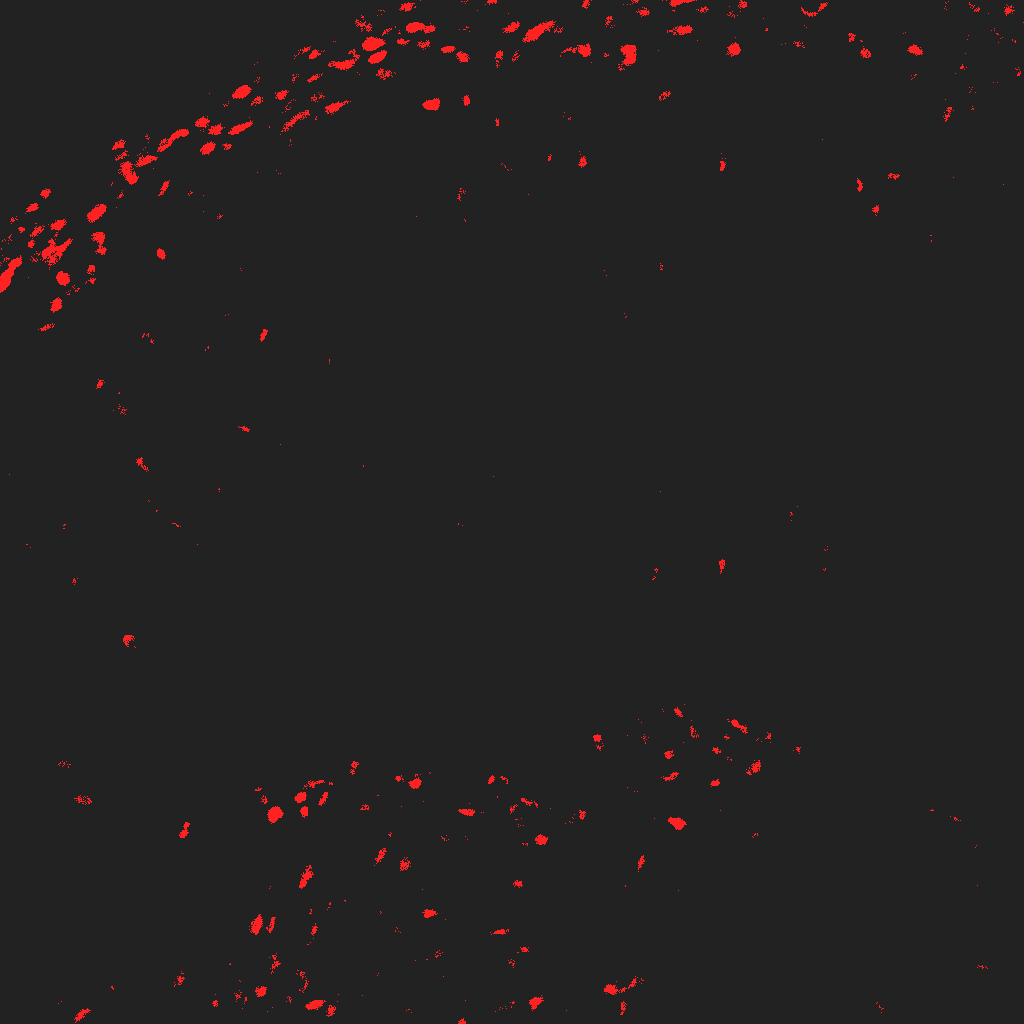

Supplement: Supplementary file 5 — Source data Fig. 4 [file 44321_2025_302_MOESM5_ESM.zip › Figure 4/4B/H9-CTIP2.tif]

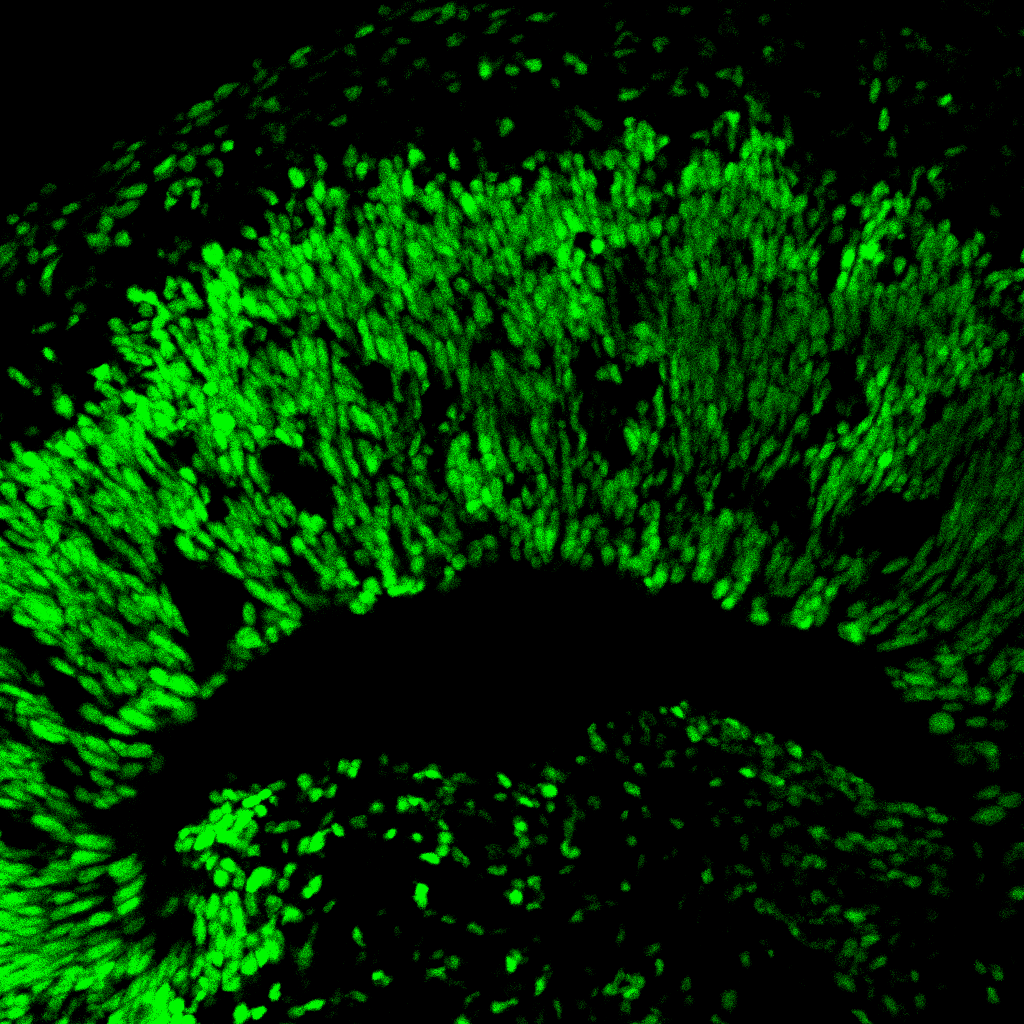

Supplement: Supplementary file 5 — Source data Fig. 4 [file 44321_2025_302_MOESM5_ESM.zip › Figure 4/4B/H9-PAX6.tif]

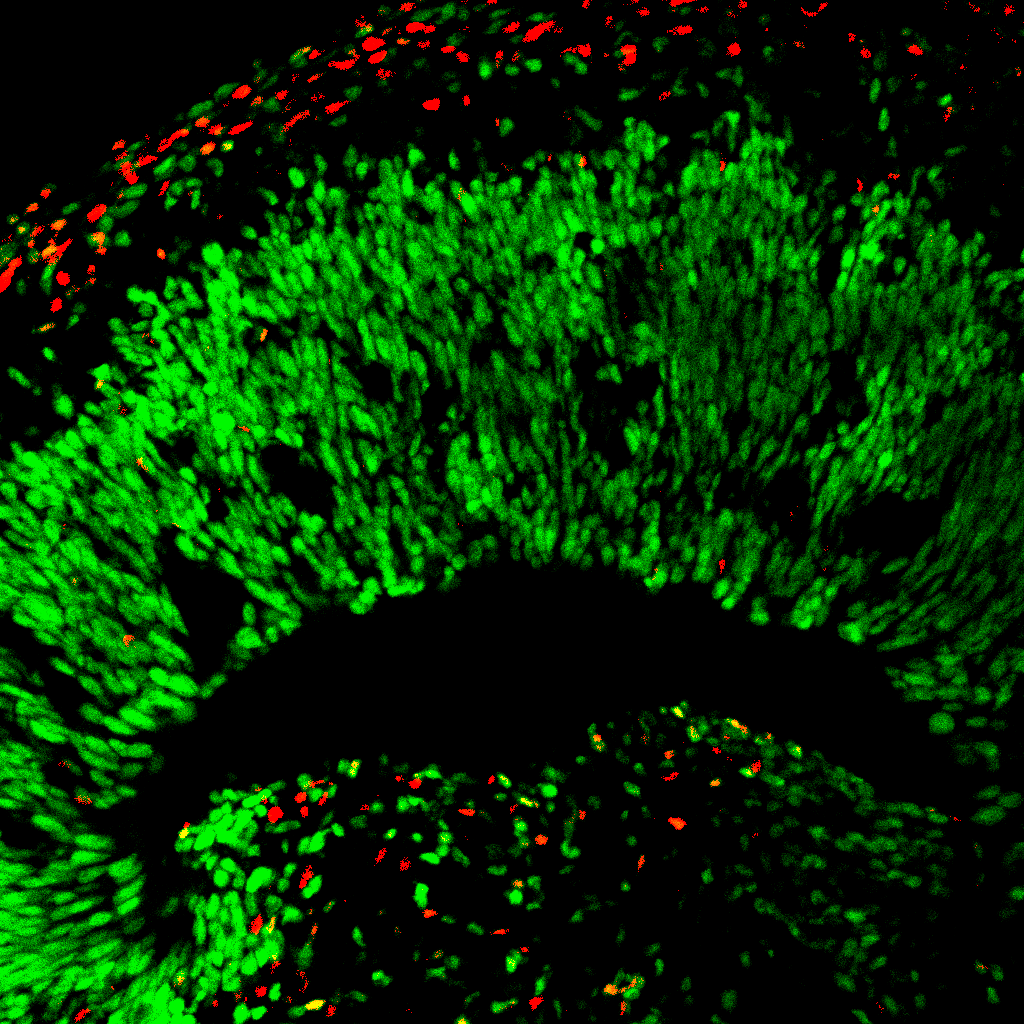

Supplement: Supplementary file 5 — Source data Fig. 4 [file 44321_2025_302_MOESM5_ESM.zip › Figure 4/4B/H9-merge.tif]

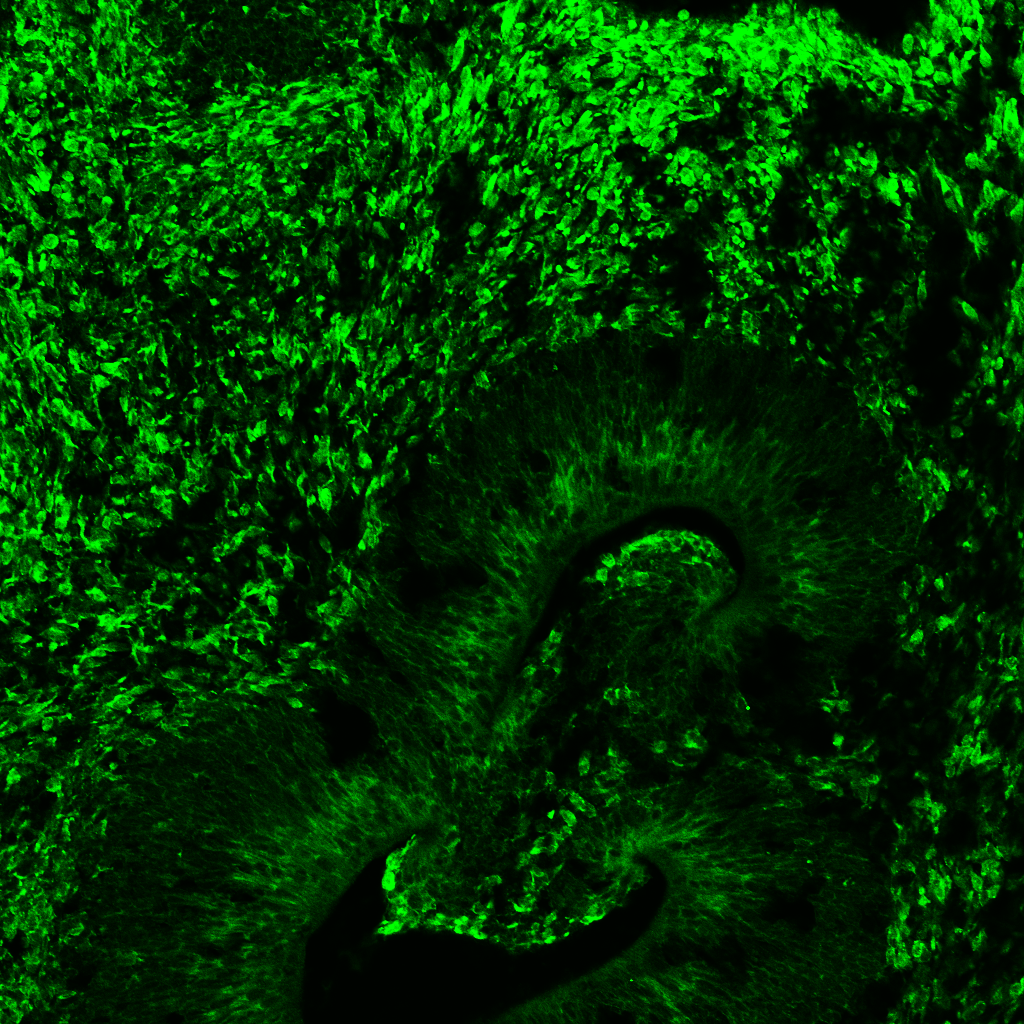

Supplement: Supplementary file 5 — Source data Fig. 4 [file 44321_2025_302_MOESM5_ESM.zip › Figure 4/4C/#12-3-HuCD.tif]

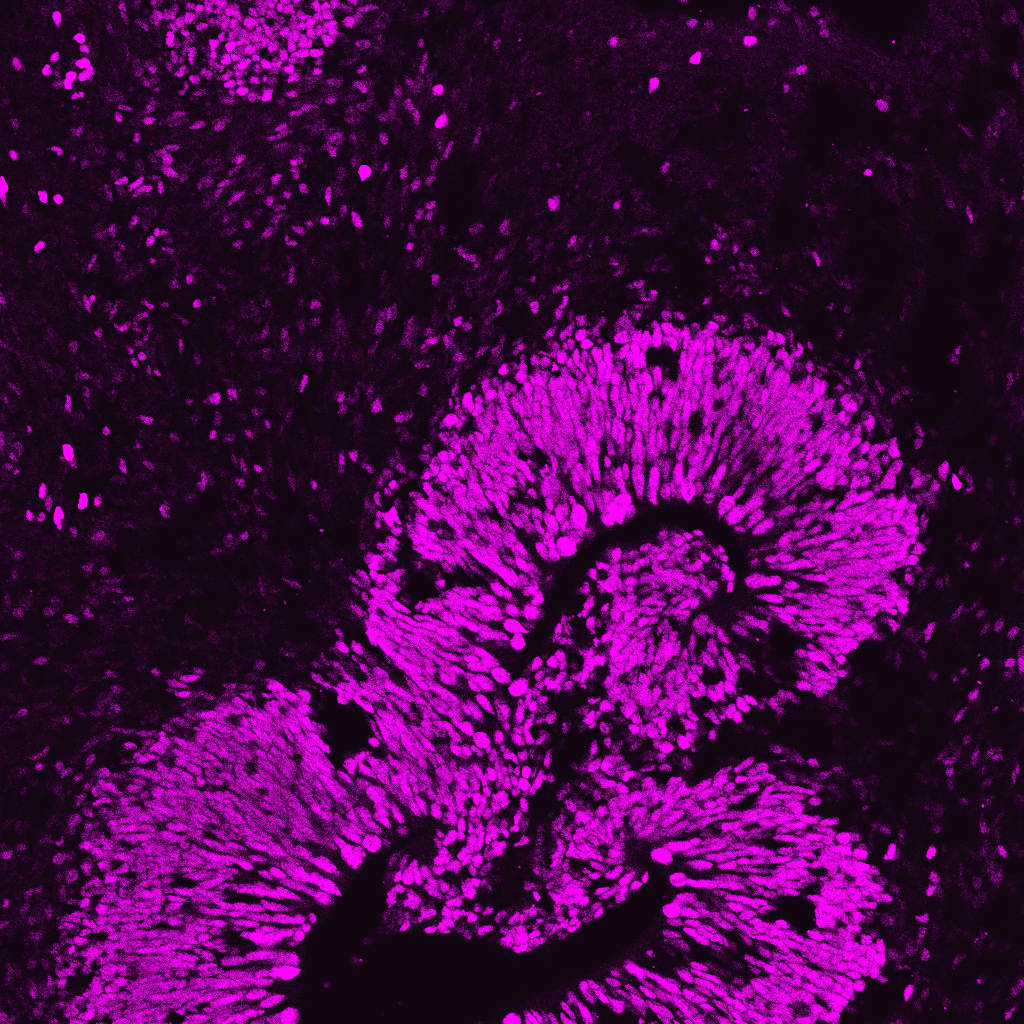

Supplement: Supplementary file 5 — Source data Fig. 4 [file 44321_2025_302_MOESM5_ESM.zip › Figure 4/4C/#12-3-PAX6.tif]

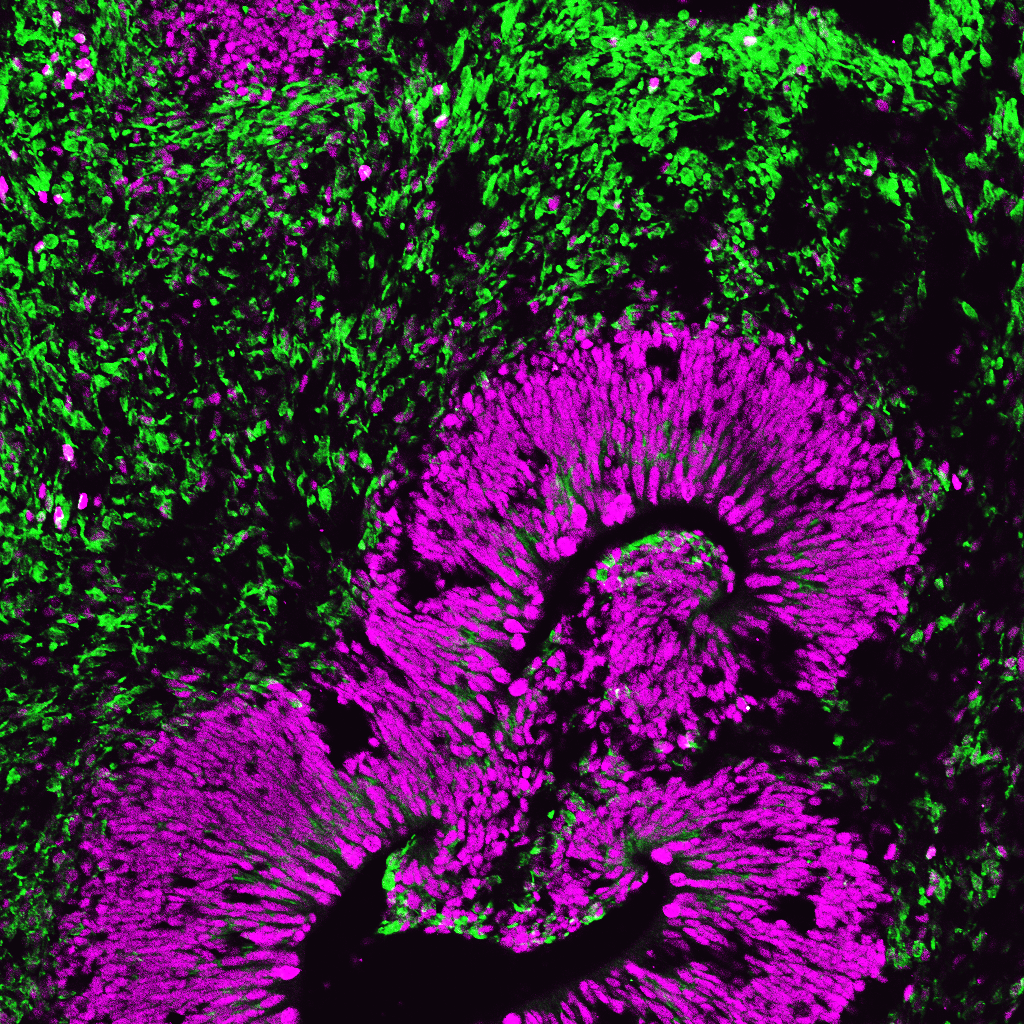

Supplement: Supplementary file 5 — Source data Fig. 4 [file 44321_2025_302_MOESM5_ESM.zip › Figure 4/4C/#12-3-merge.tif]

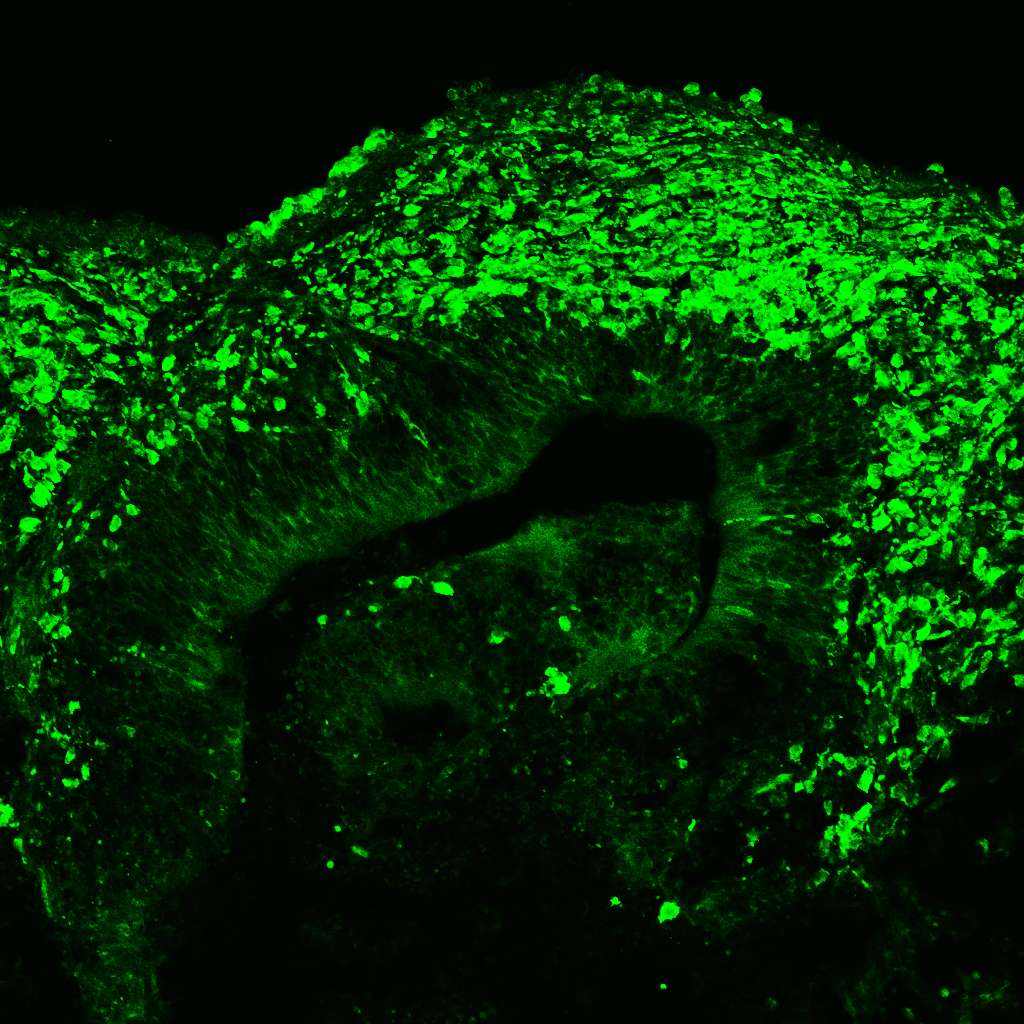

Supplement: Supplementary file 5 — Source data Fig. 4 [file 44321_2025_302_MOESM5_ESM.zip › Figure 4/4C/#7-5-HuCD.tif]

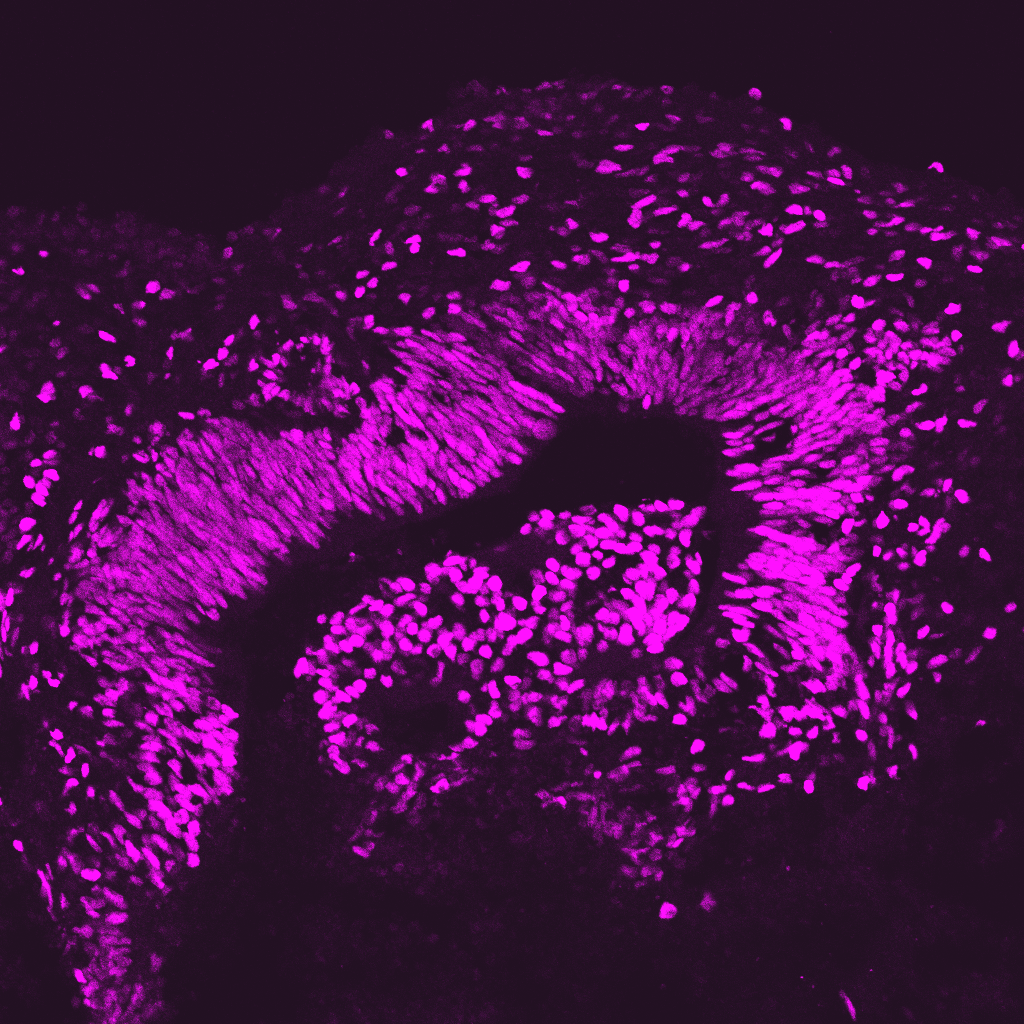

Supplement: Supplementary file 5 — Source data Fig. 4 [file 44321_2025_302_MOESM5_ESM.zip › Figure 4/4C/#7-5-PAX6.tif]

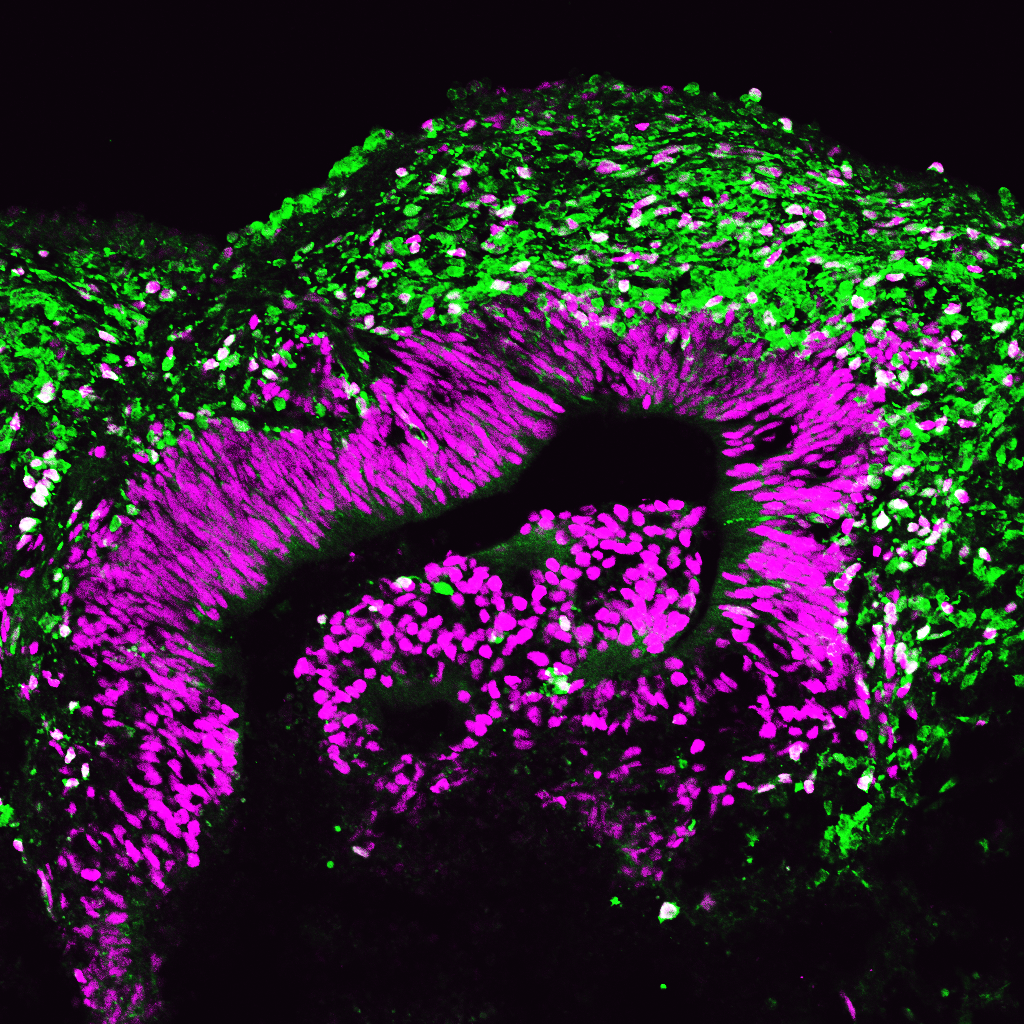

Supplement: Supplementary file 5 — Source data Fig. 4 [file 44321_2025_302_MOESM5_ESM.zip › Figure 4/4C/#7-5-merge.tif]

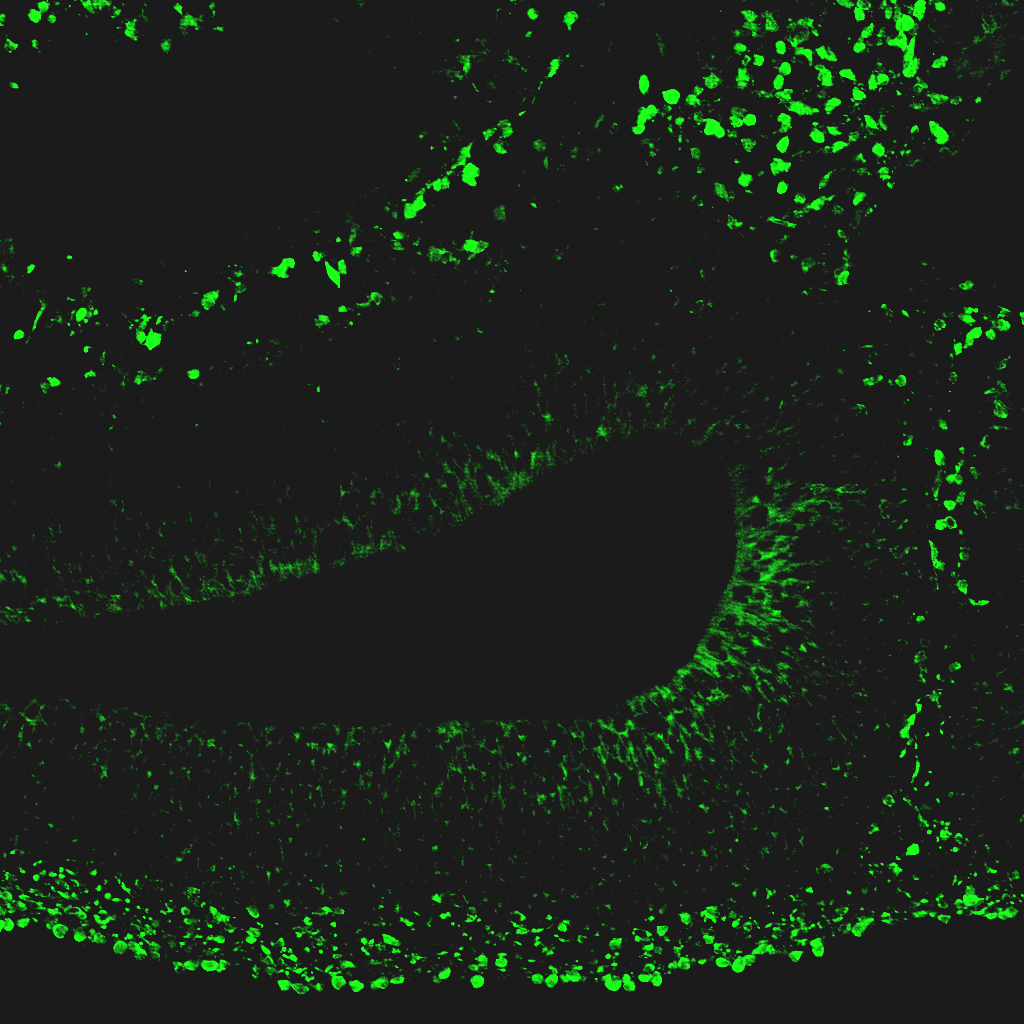

Supplement: Supplementary file 5 — Source data Fig. 4 [file 44321_2025_302_MOESM5_ESM.zip › Figure 4/4C/H9-HuCD.tif]

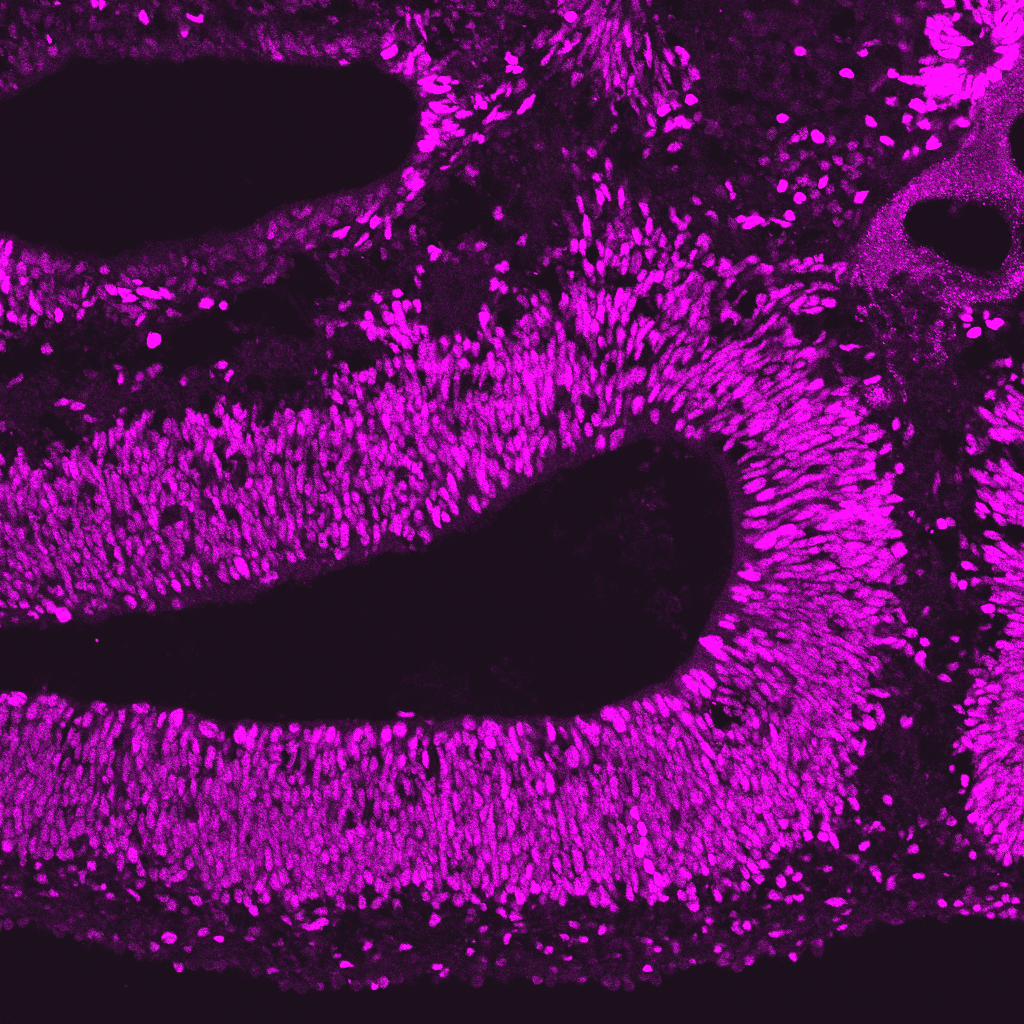

Supplement: Supplementary file 5 — Source data Fig. 4 [file 44321_2025_302_MOESM5_ESM.zip › Figure 4/4C/H9-PAX6.tif]

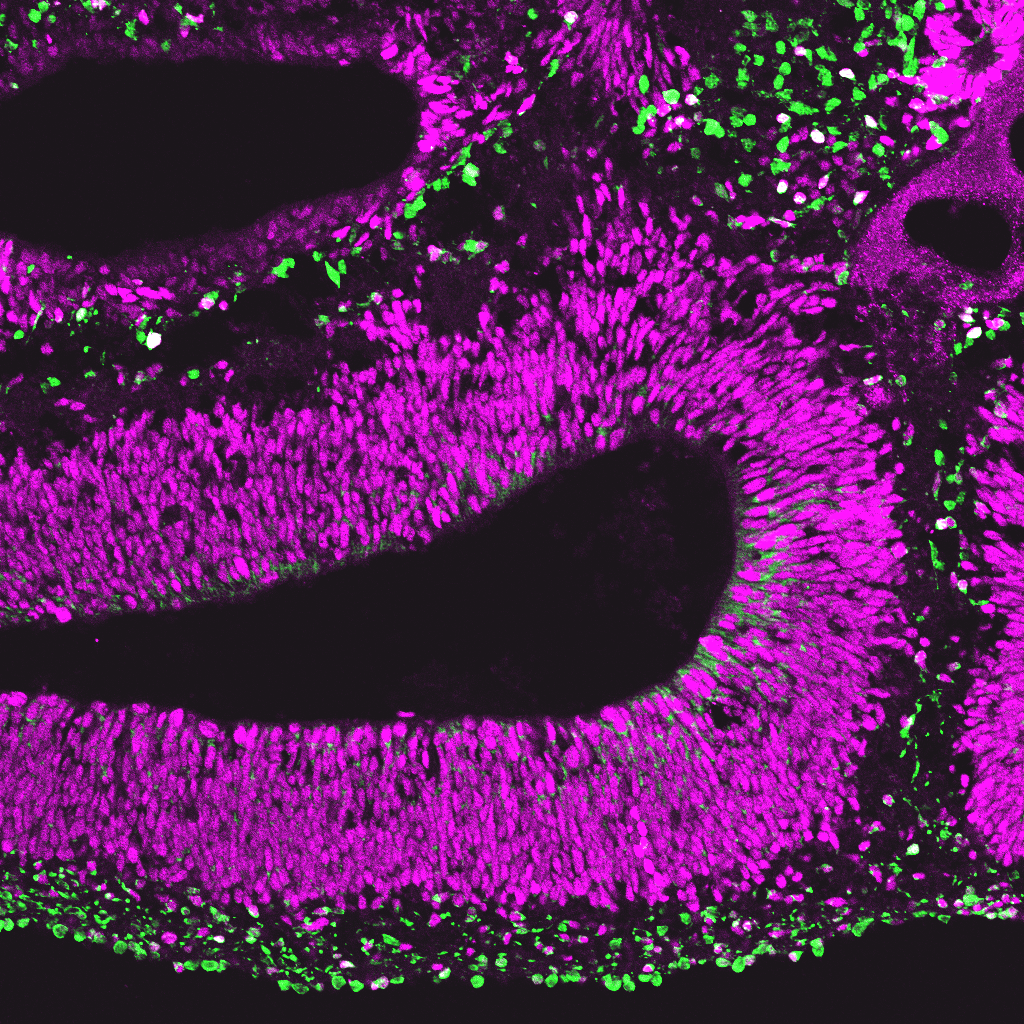

Supplement: Supplementary file 5 — Source data Fig. 4 [file 44321_2025_302_MOESM5_ESM.zip › Figure 4/4C/H9-merge.tif]

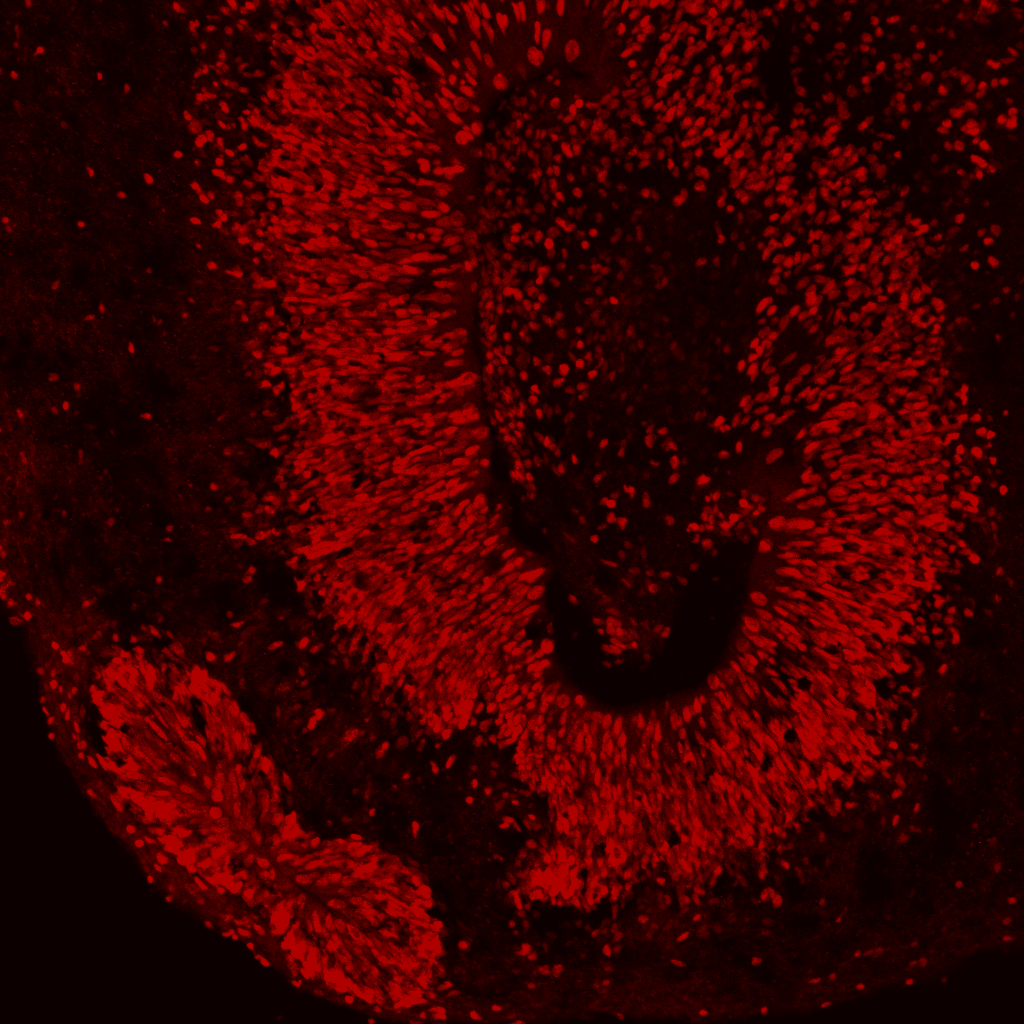

Supplement: Supplementary file 5 — Source data Fig. 4 [file 44321_2025_302_MOESM5_ESM.zip › Figure 4/4D/#12-3-SOX2.tif]

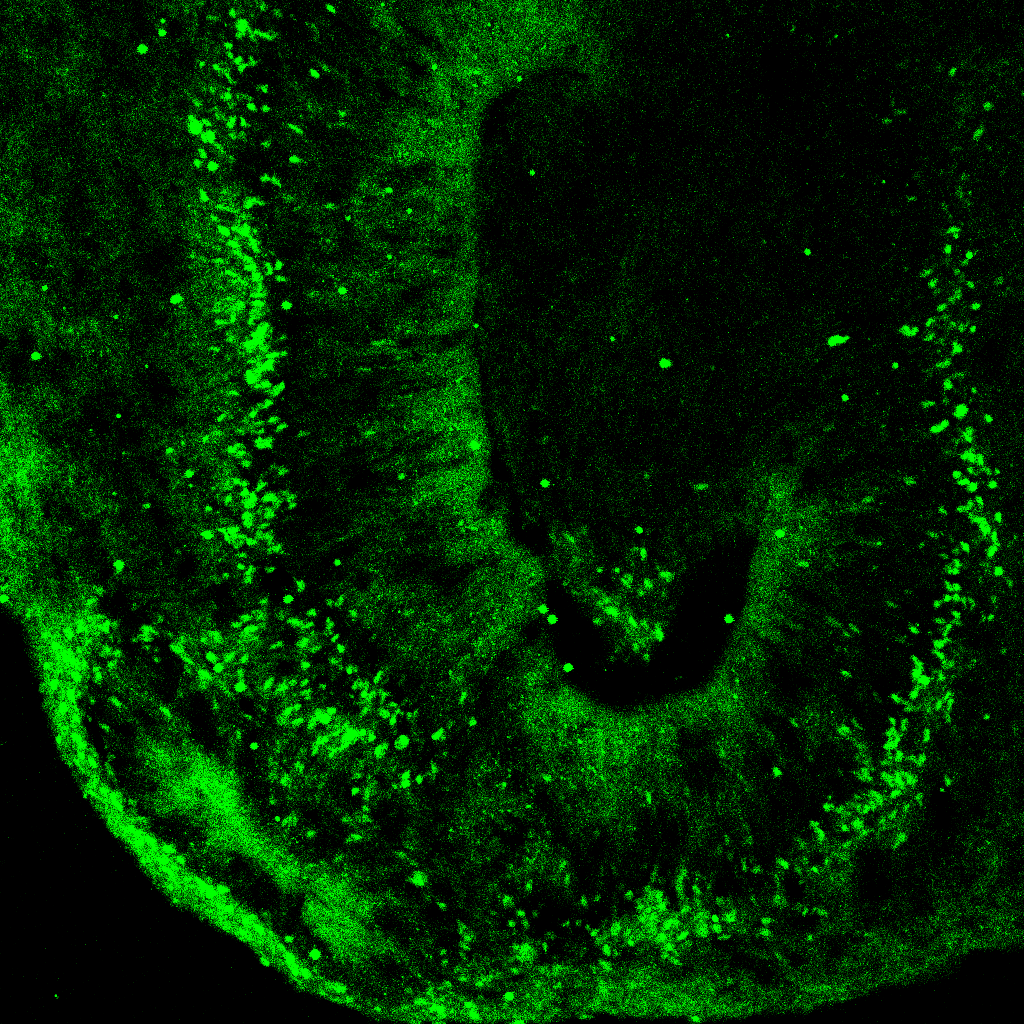

Supplement: Supplementary file 5 — Source data Fig. 4 [file 44321_2025_302_MOESM5_ESM.zip › Figure 4/4D/#12-3-TBR2.tif]

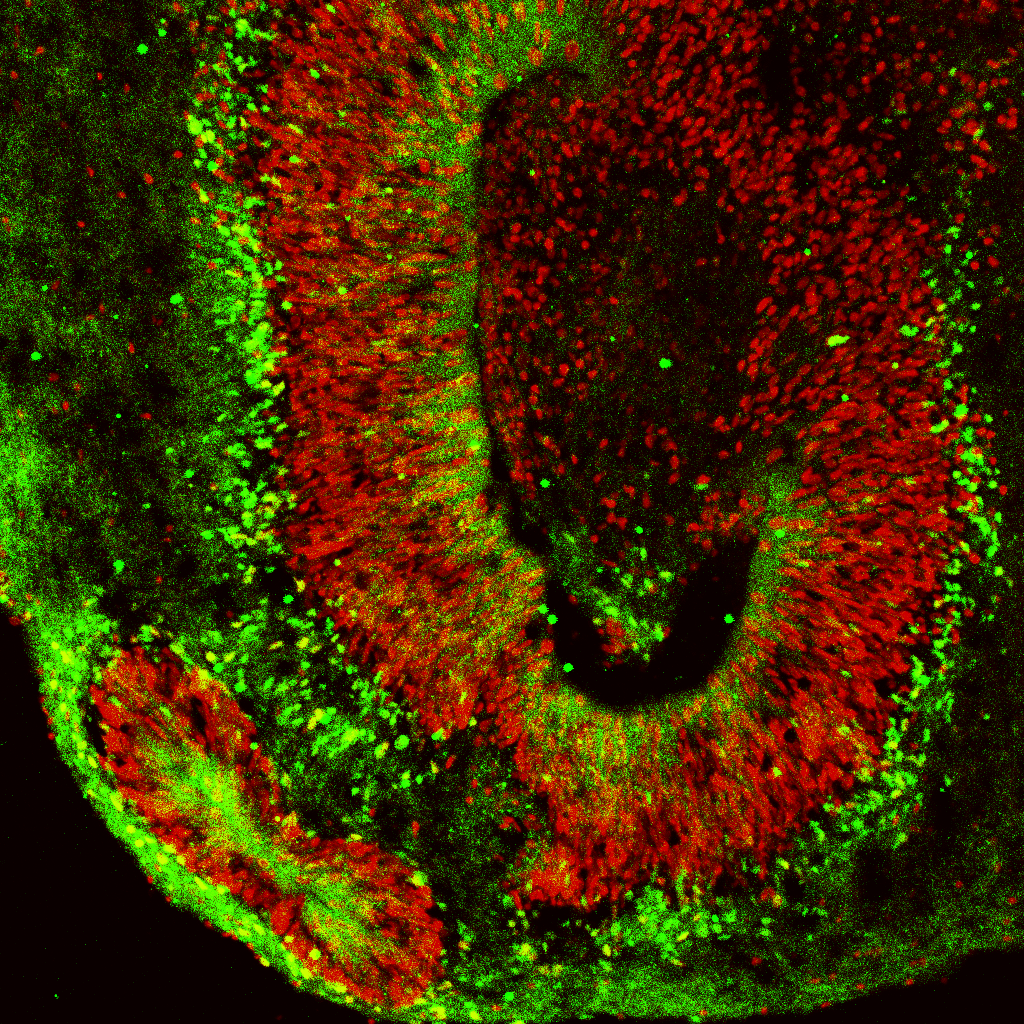

Supplement: Supplementary file 5 — Source data Fig. 4 [file 44321_2025_302_MOESM5_ESM.zip › Figure 4/4D/#12-3-merge.tif]

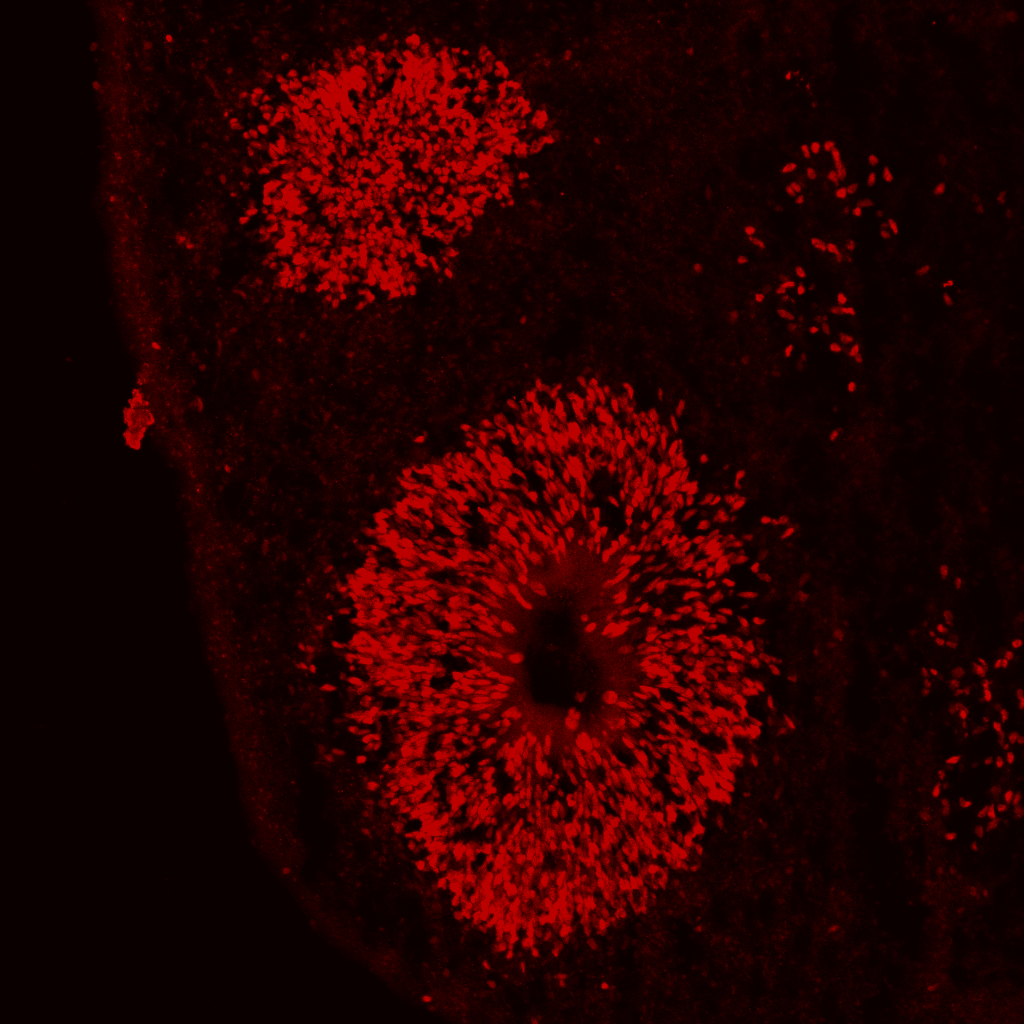

Supplement: Supplementary file 5 — Source data Fig. 4 [file 44321_2025_302_MOESM5_ESM.zip › Figure 4/4D/#7-5-SOX2.tif]

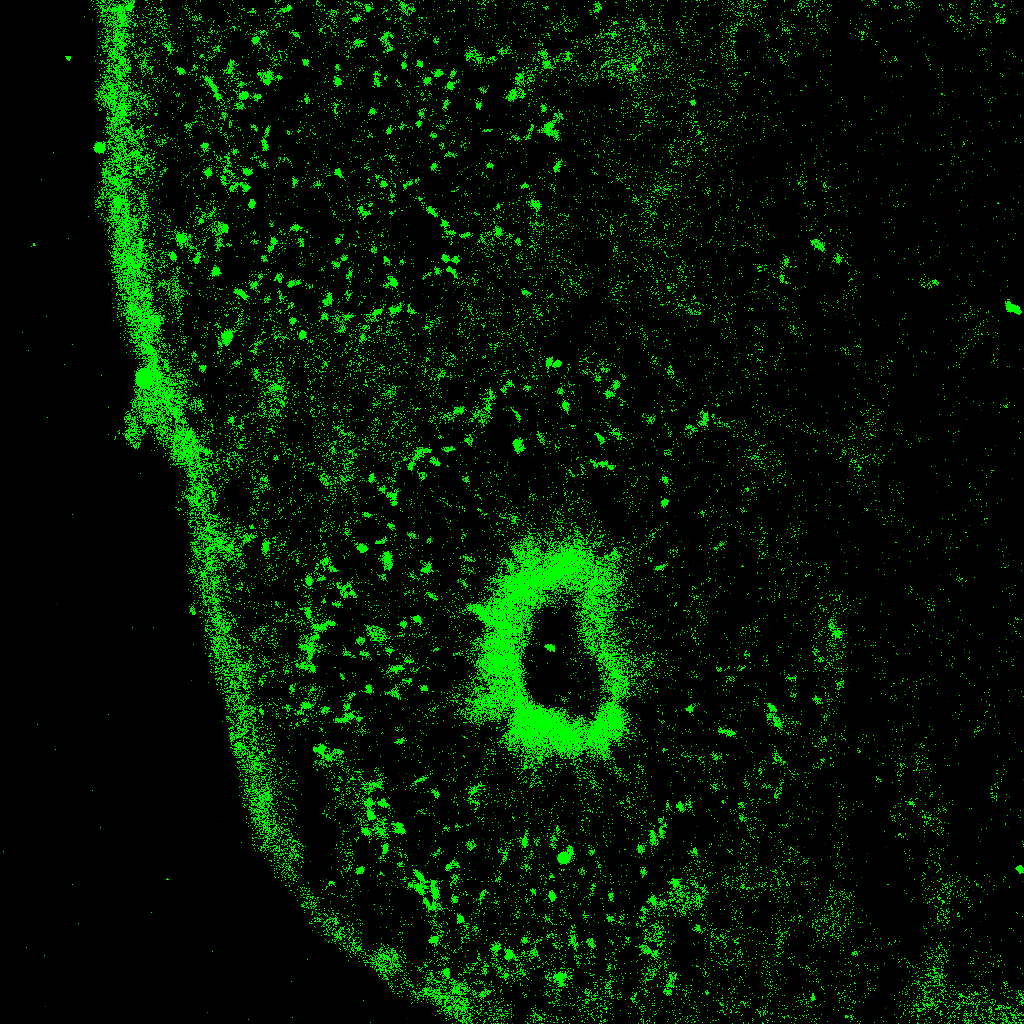

Supplement: Supplementary file 5 — Source data Fig. 4 [file 44321_2025_302_MOESM5_ESM.zip › Figure 4/4D/#7-5-TBR2.tif]

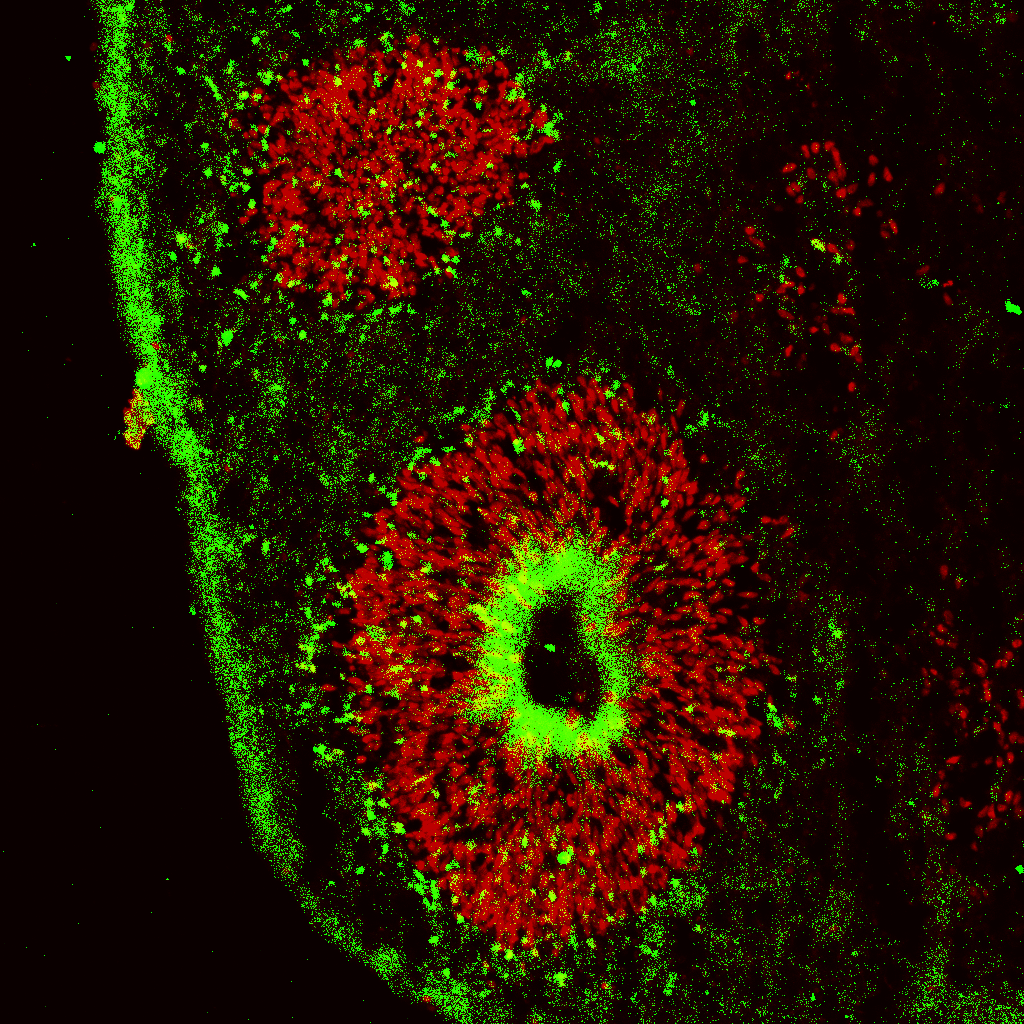

Supplement: Supplementary file 5 — Source data Fig. 4 [file 44321_2025_302_MOESM5_ESM.zip › Figure 4/4D/#7-5-merge.tif]

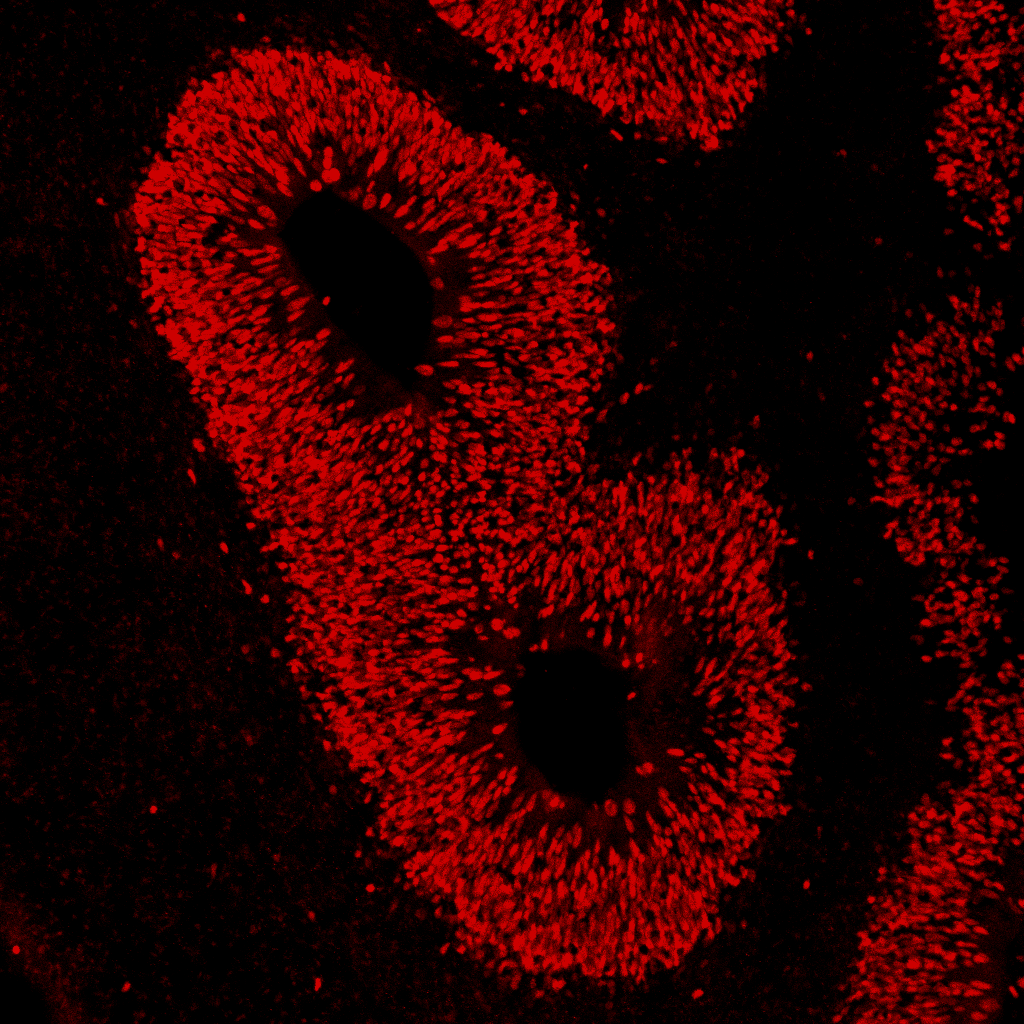

Supplement: Supplementary file 5 — Source data Fig. 4 [file 44321_2025_302_MOESM5_ESM.zip › Figure 4/4D/H9-SOX2.tif]

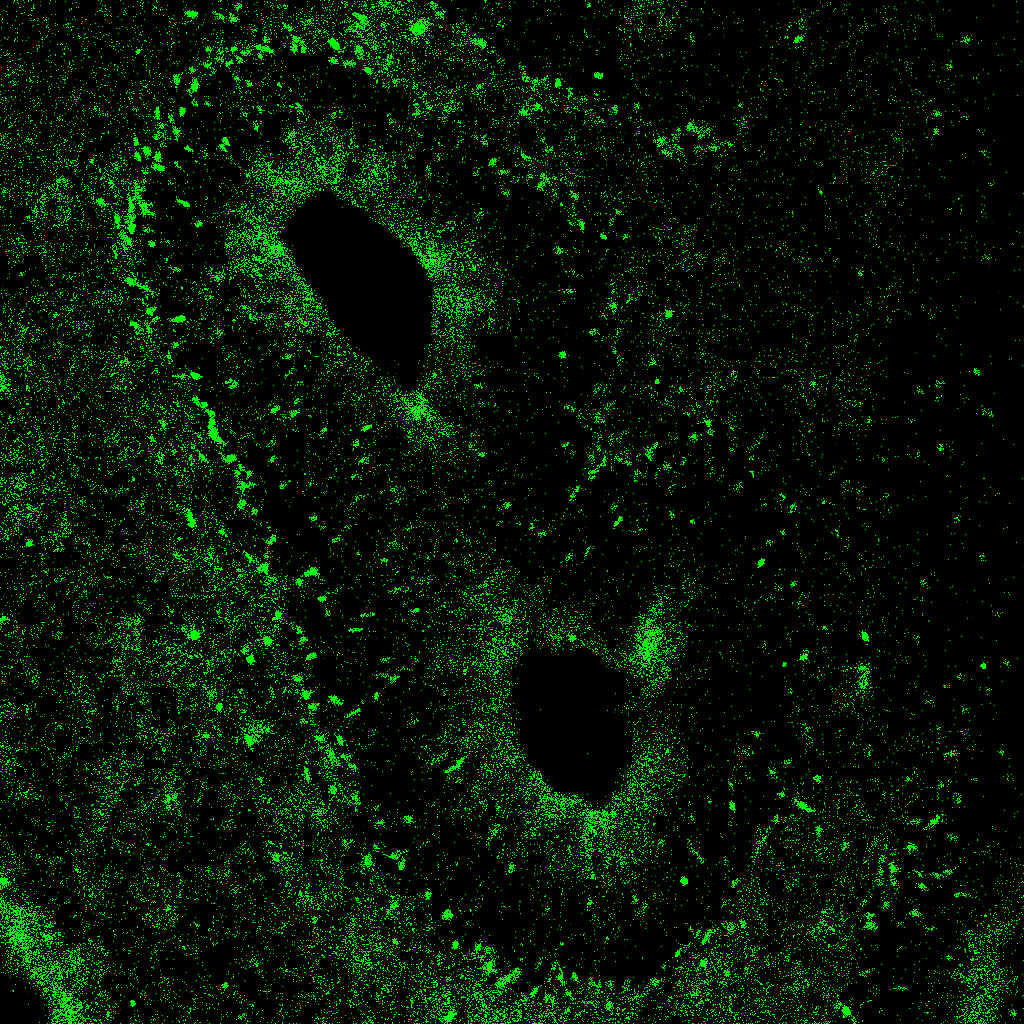

Supplement: Supplementary file 5 — Source data Fig. 4 [file 44321_2025_302_MOESM5_ESM.zip › Figure 4/4D/H9-TBR2.tif]

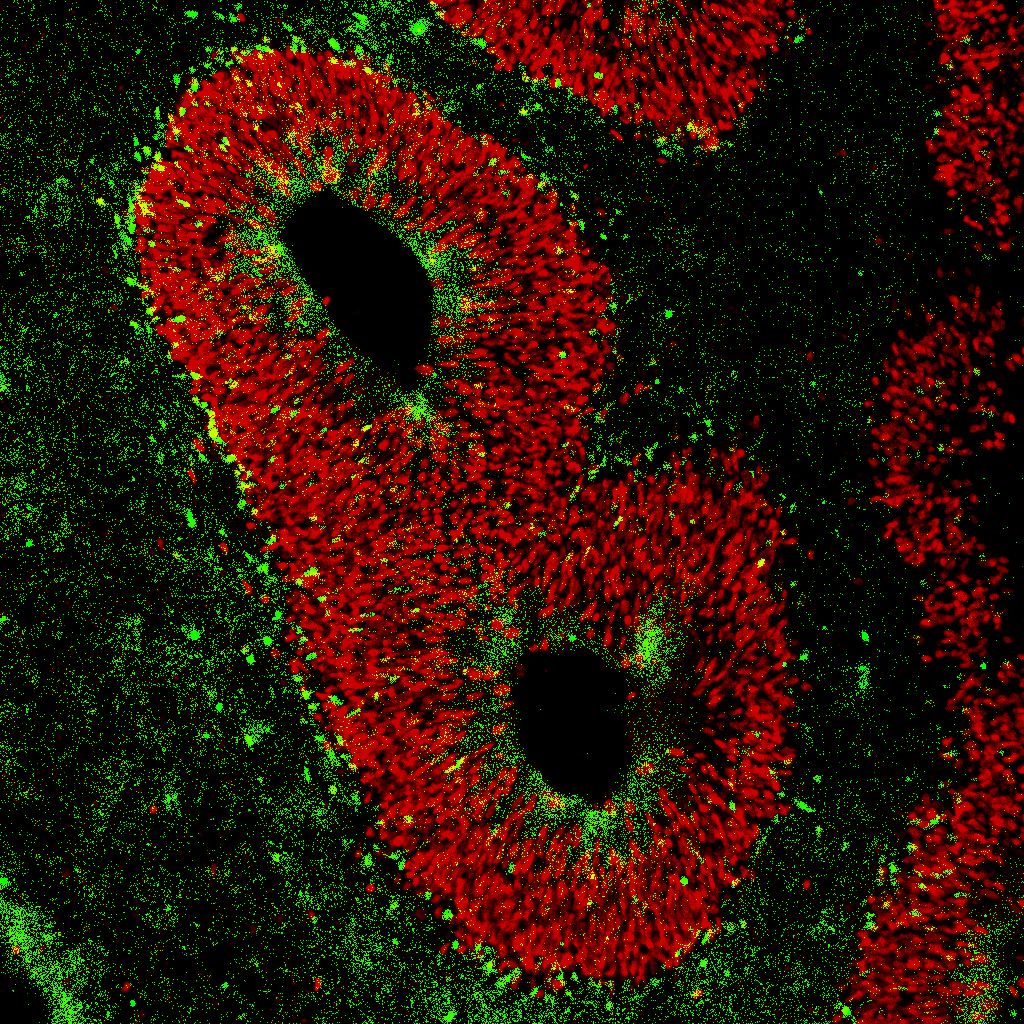

Supplement: Supplementary file 5 — Source data Fig. 4 [file 44321_2025_302_MOESM5_ESM.zip › Figure 4/4D/H9-merge.tif]

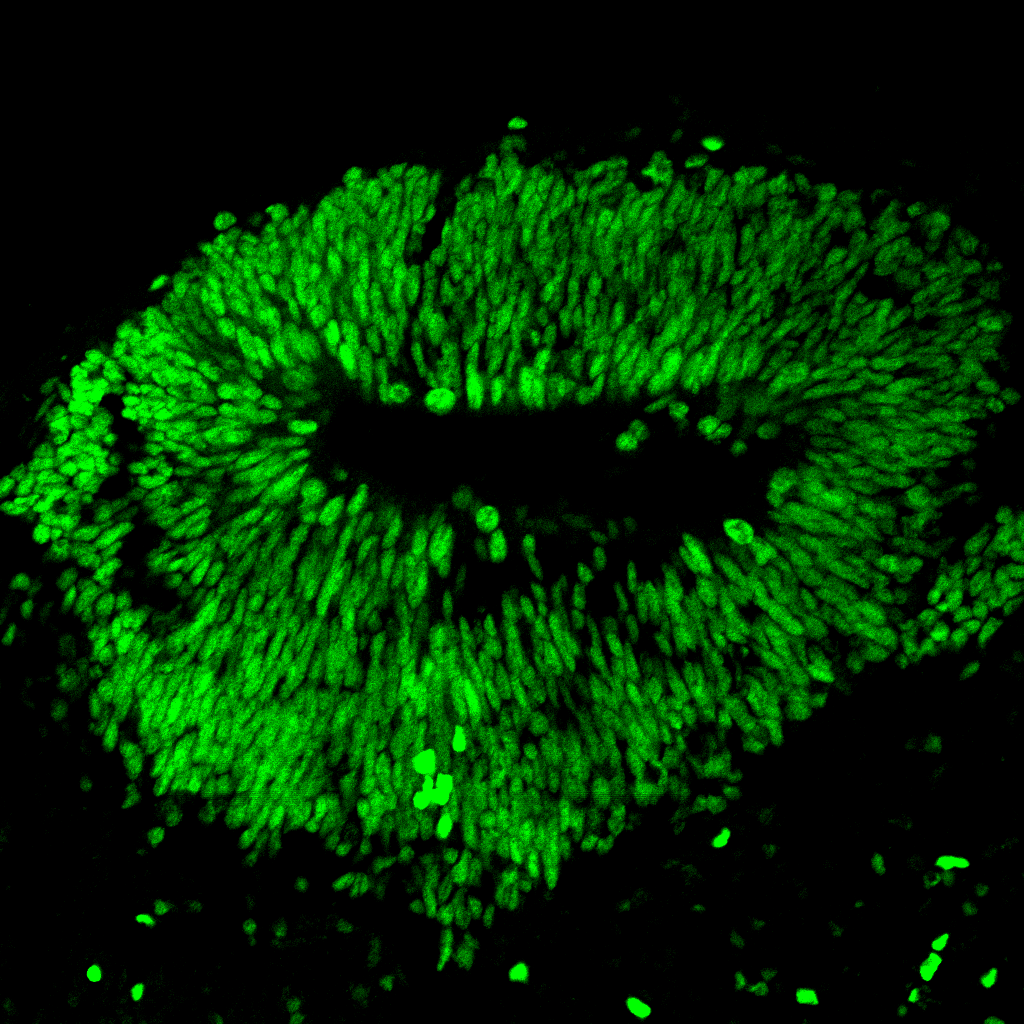

Supplement: Supplementary file 5 — Source data Fig. 4 [file 44321_2025_302_MOESM5_ESM.zip › Figure 4/4E/#12-3-PAX6.tif]

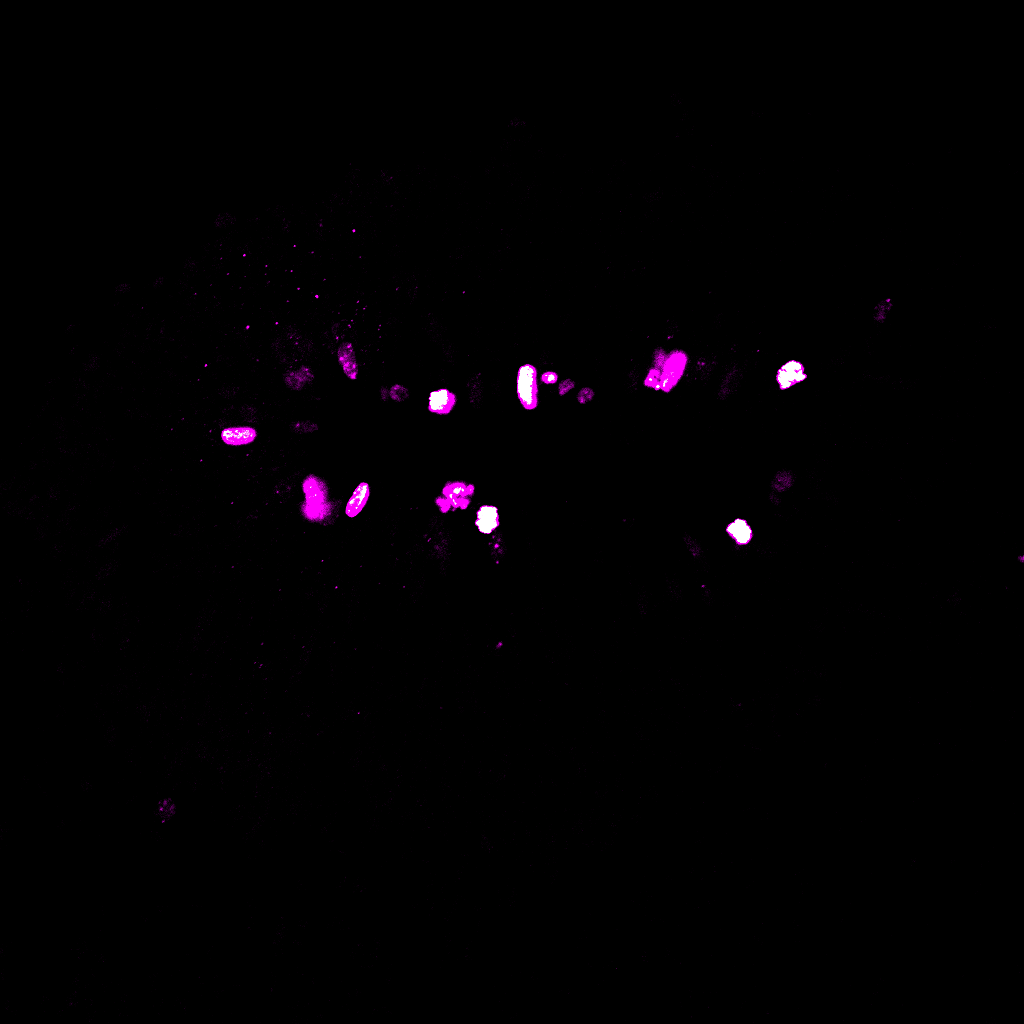

Supplement: Supplementary file 5 — Source data Fig. 4 [file 44321_2025_302_MOESM5_ESM.zip › Figure 4/4E/#12-3-PH3.tif]

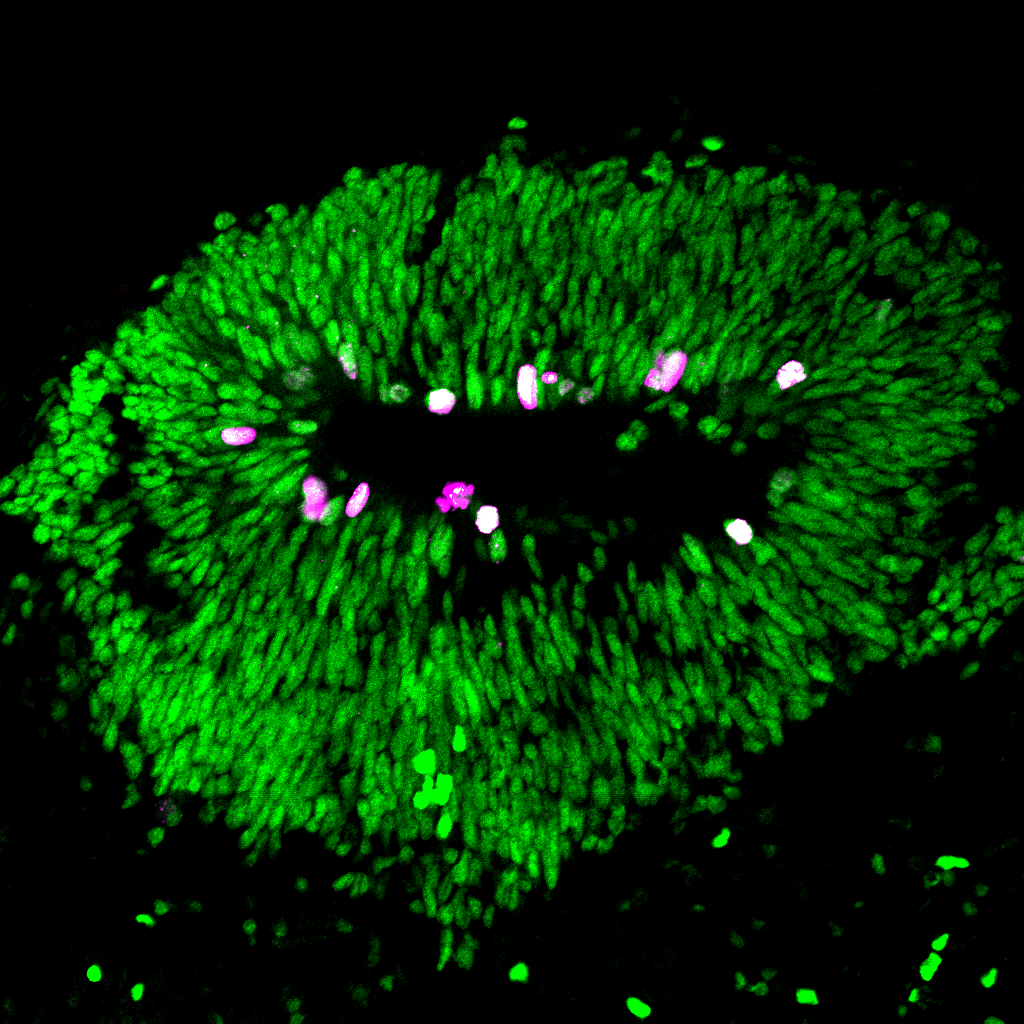

Supplement: Supplementary file 5 — Source data Fig. 4 [file 44321_2025_302_MOESM5_ESM.zip › Figure 4/4E/#12-3-merge.tif]

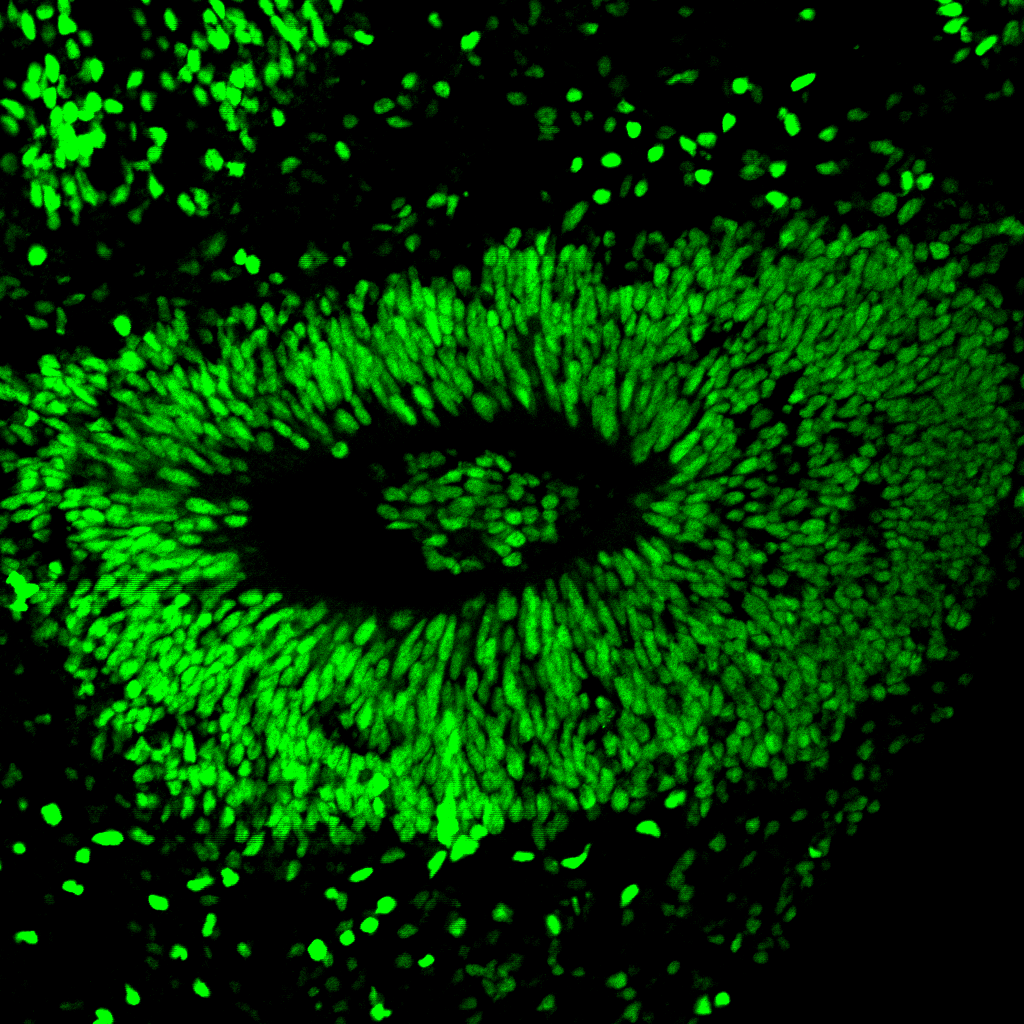

Supplement: Supplementary file 5 — Source data Fig. 4 [file 44321_2025_302_MOESM5_ESM.zip › Figure 4/4E/#7-5-PAX6.tif]

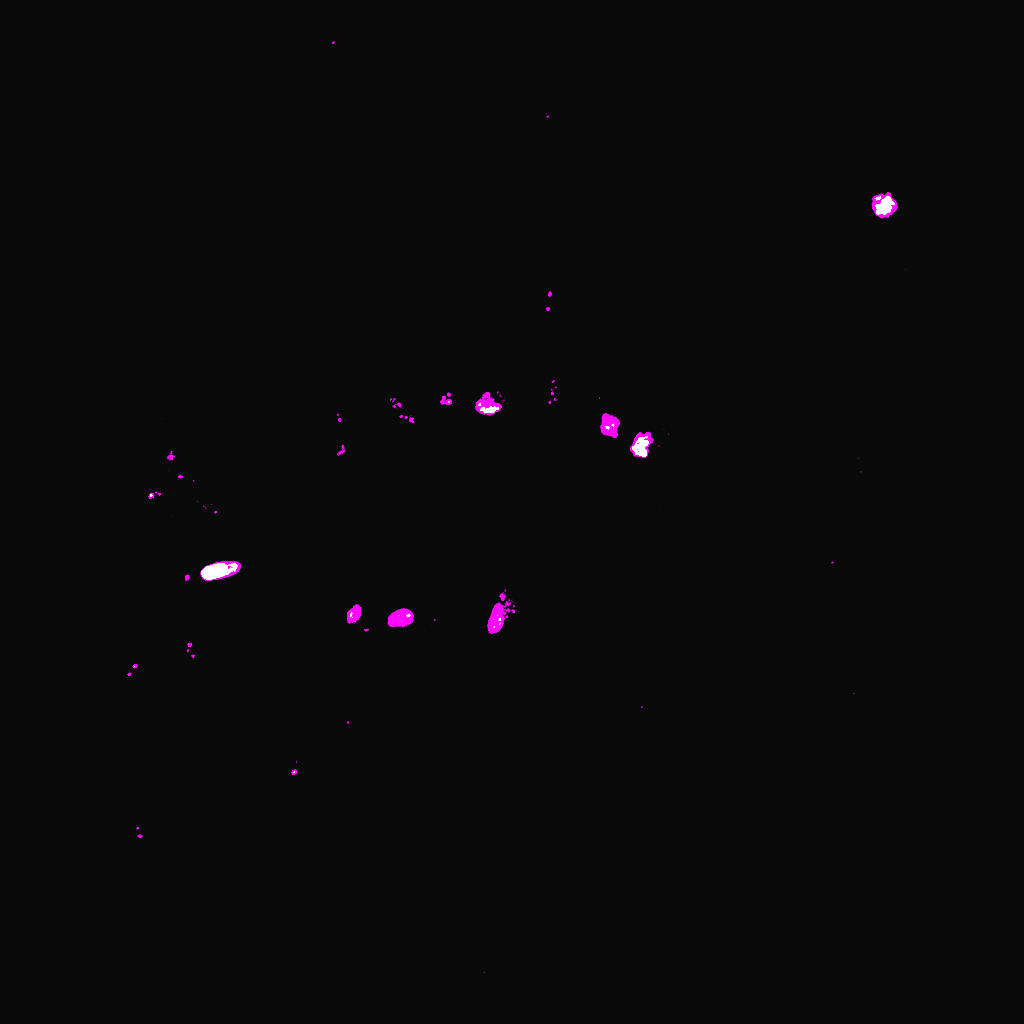

Supplement: Supplementary file 5 — Source data Fig. 4 [file 44321_2025_302_MOESM5_ESM.zip › Figure 4/4E/#7-5-PH3.tif]

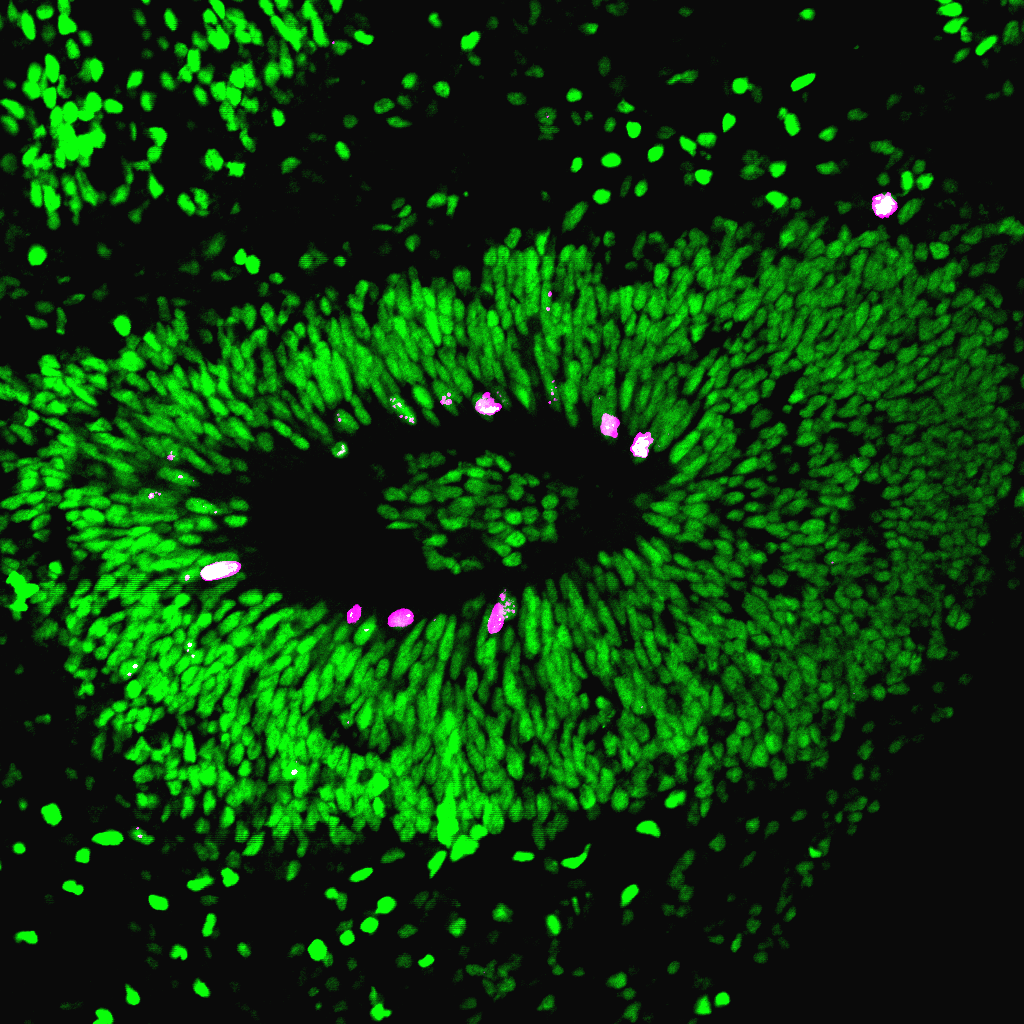

Supplement: Supplementary file 5 — Source data Fig. 4 [file 44321_2025_302_MOESM5_ESM.zip › Figure 4/4E/#7-5-merge.tif]

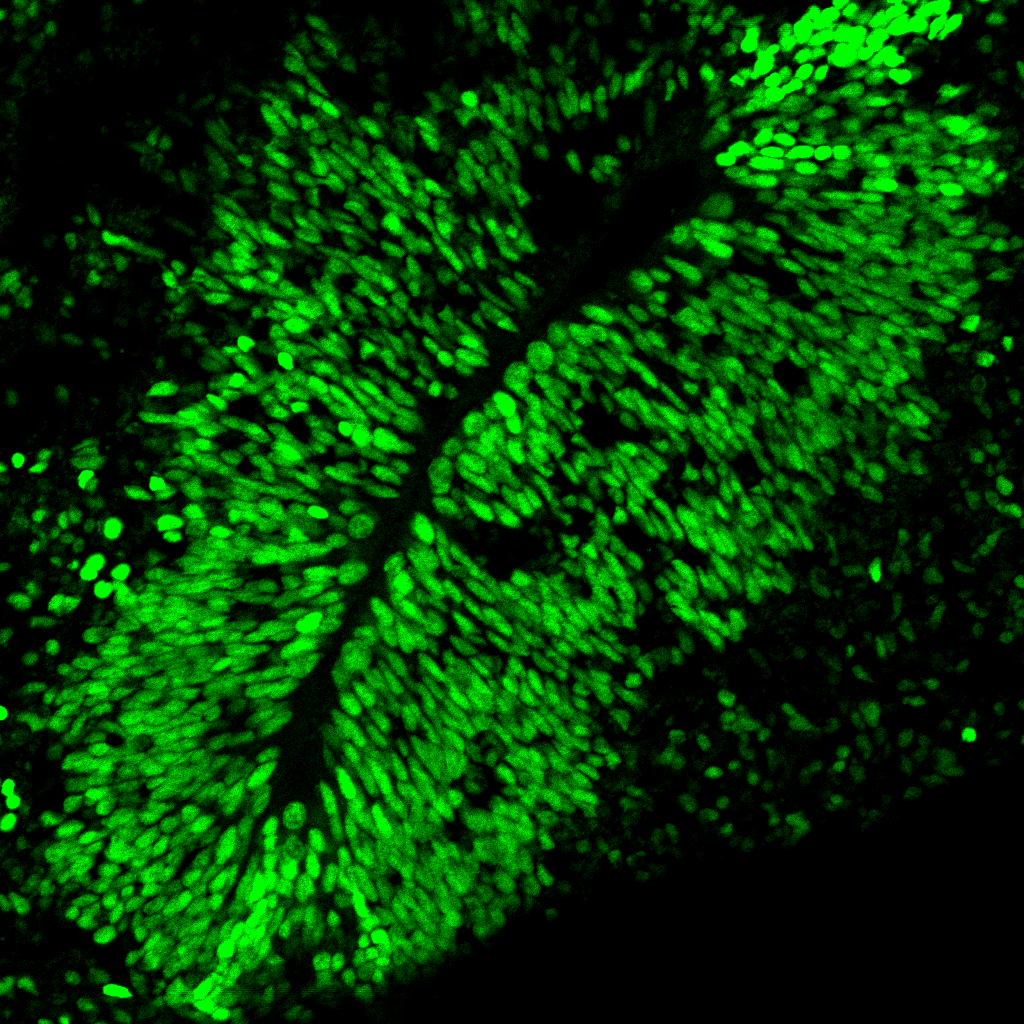

Supplement: Supplementary file 5 — Source data Fig. 4 [file 44321_2025_302_MOESM5_ESM.zip › Figure 4/4E/H9-PAX6.tif]

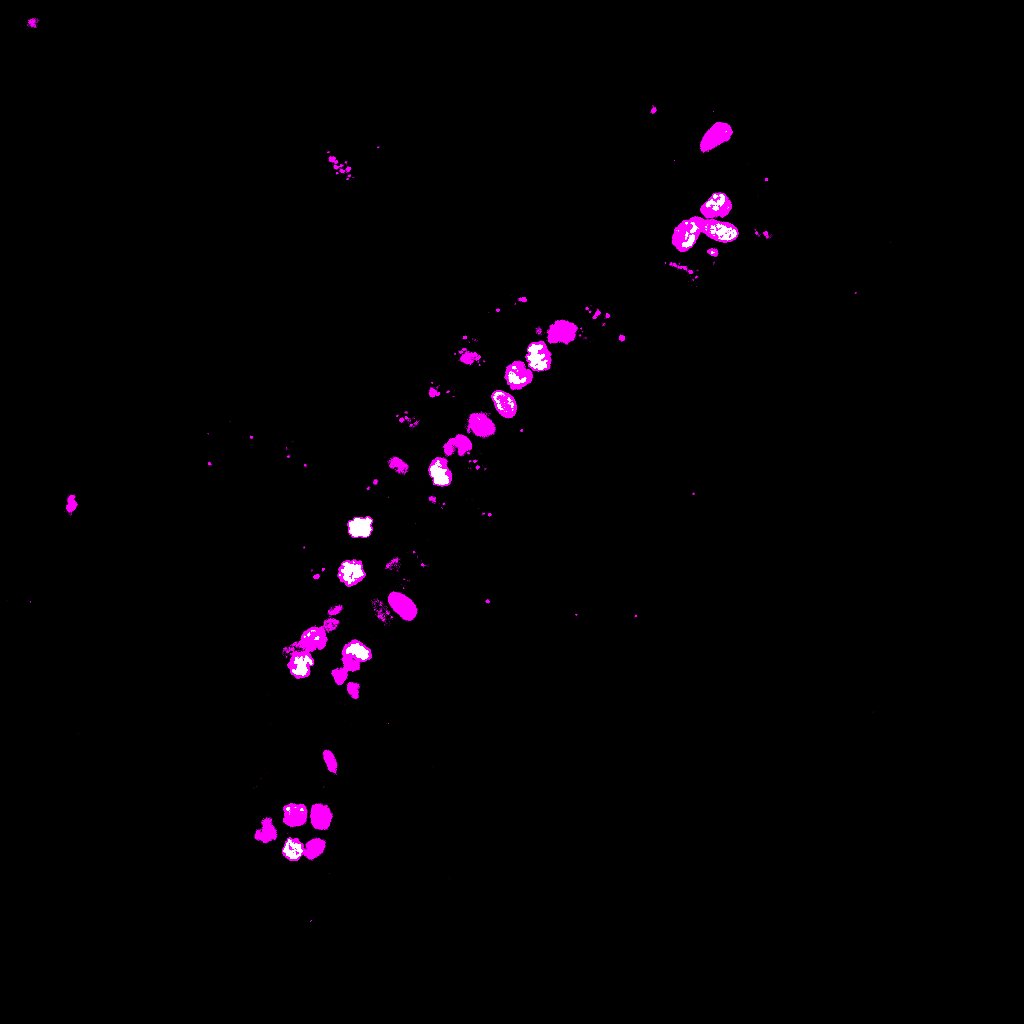

Supplement: Supplementary file 5 — Source data Fig. 4 [file 44321_2025_302_MOESM5_ESM.zip › Figure 4/4E/H9-PH3.tif]

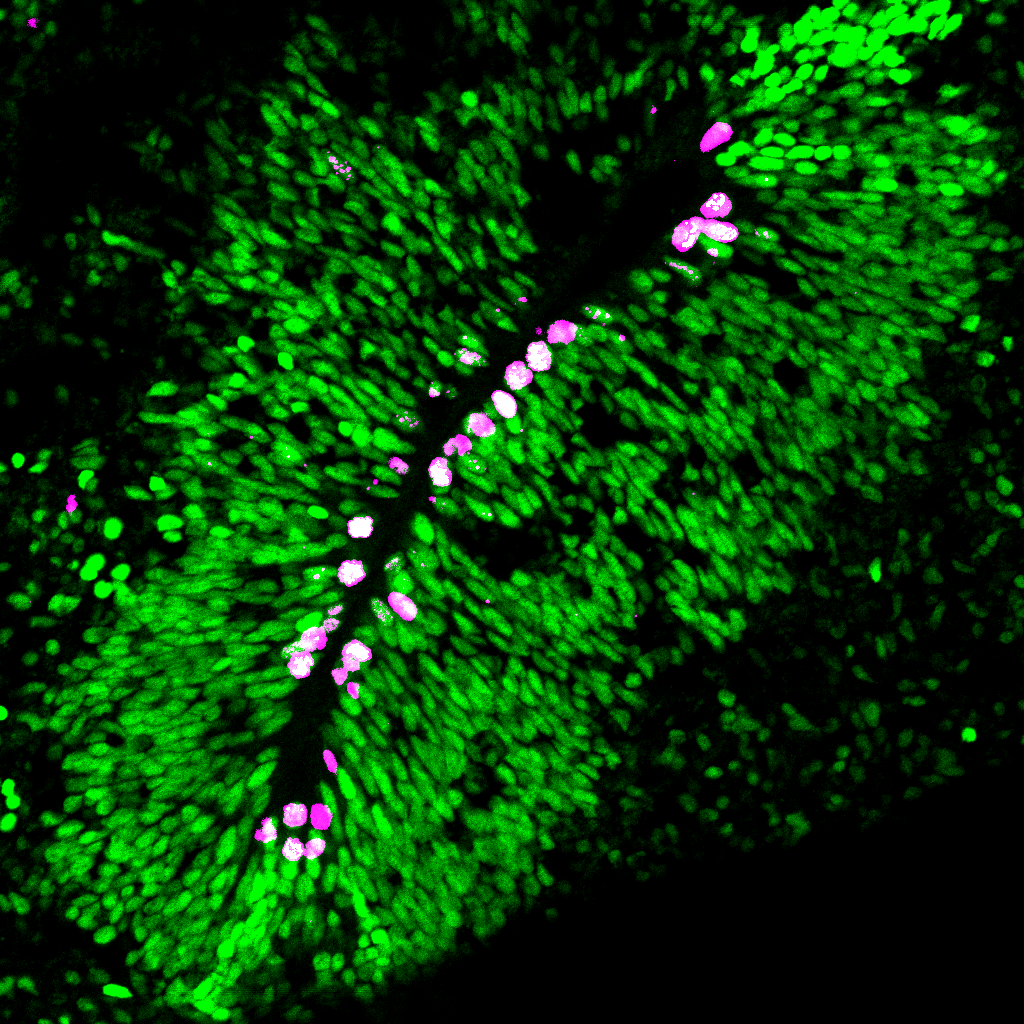

Supplement: Supplementary file 5 — Source data Fig. 4 [file 44321_2025_302_MOESM5_ESM.zip › Figure 4/4E/H9-merge.tif]

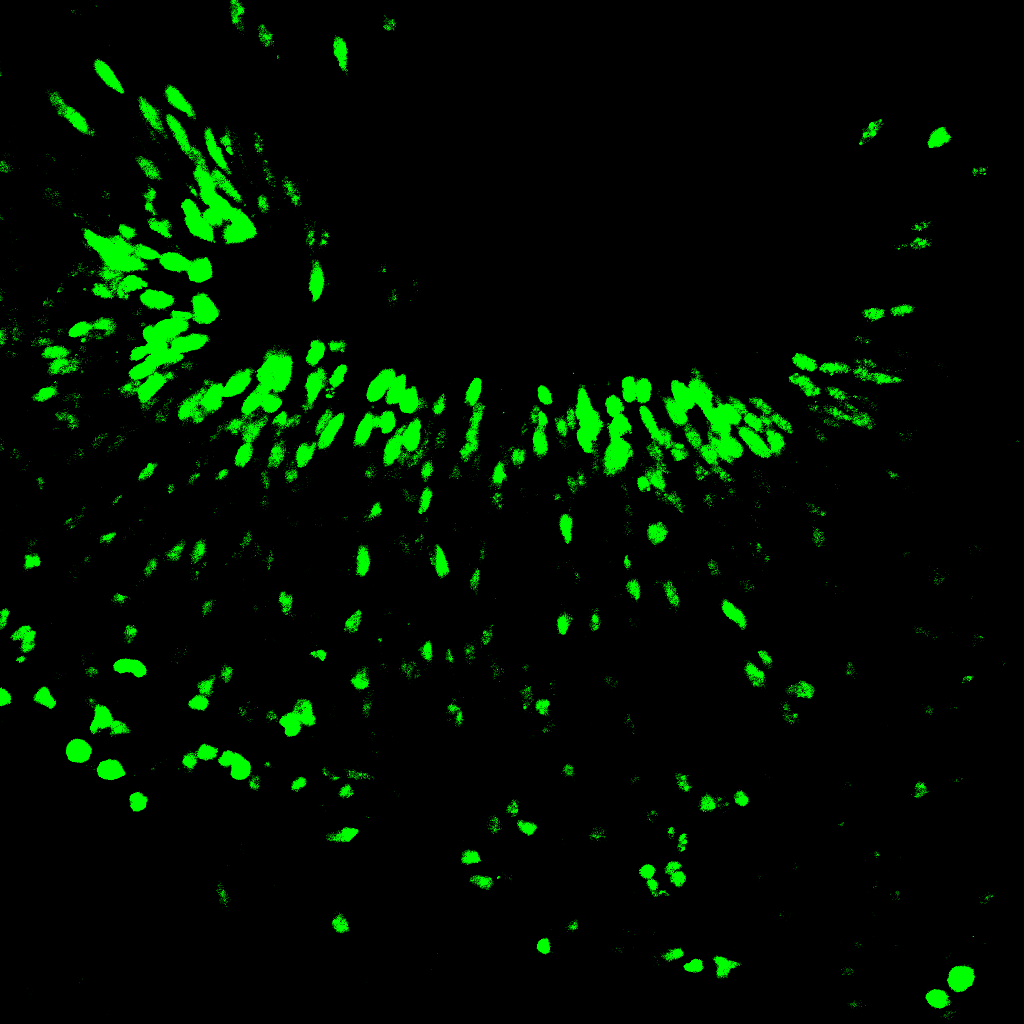

Supplement: Supplementary file 5 — Source data Fig. 4 [file 44321_2025_302_MOESM5_ESM.zip › Figure 4/4F/#12-3-Ki67.tif]

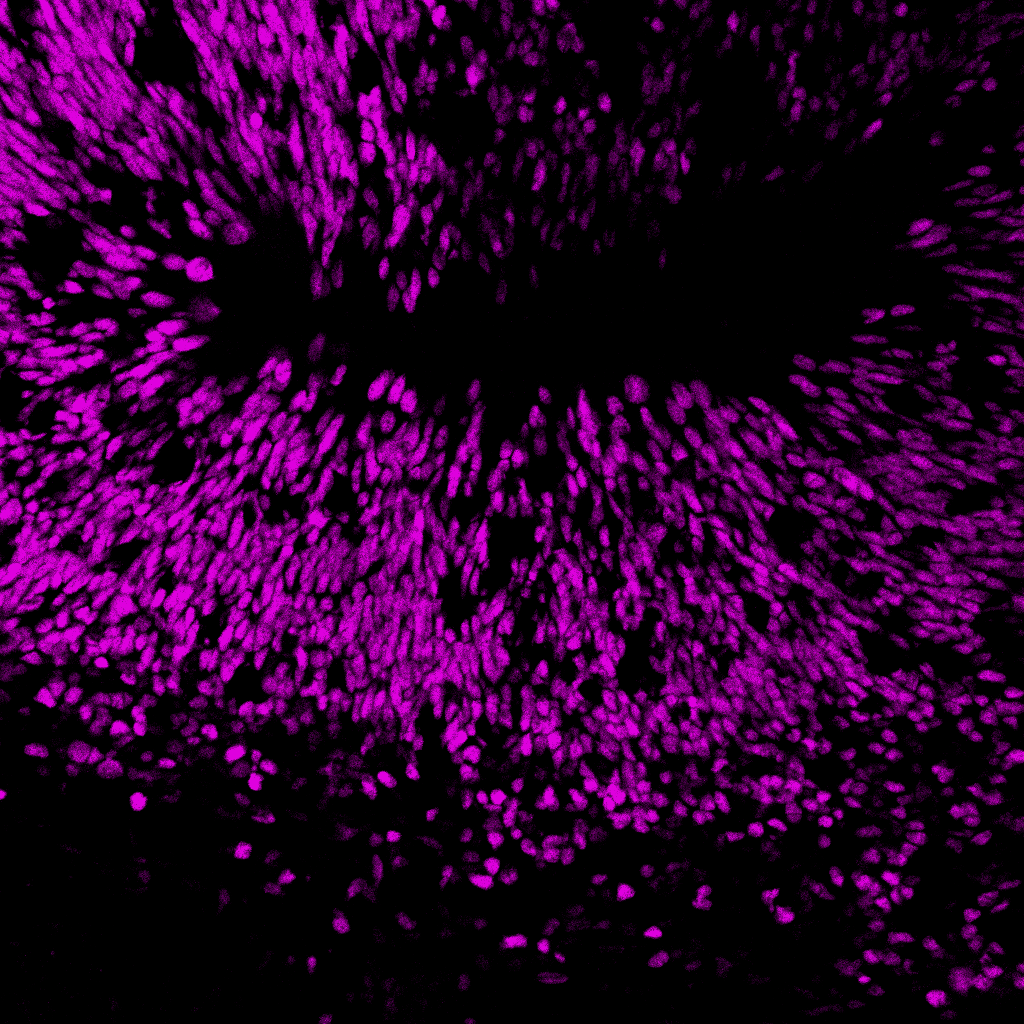

Supplement: Supplementary file 5 — Source data Fig. 4 [file 44321_2025_302_MOESM5_ESM.zip › Figure 4/4F/#12-3-PAX6.tif]

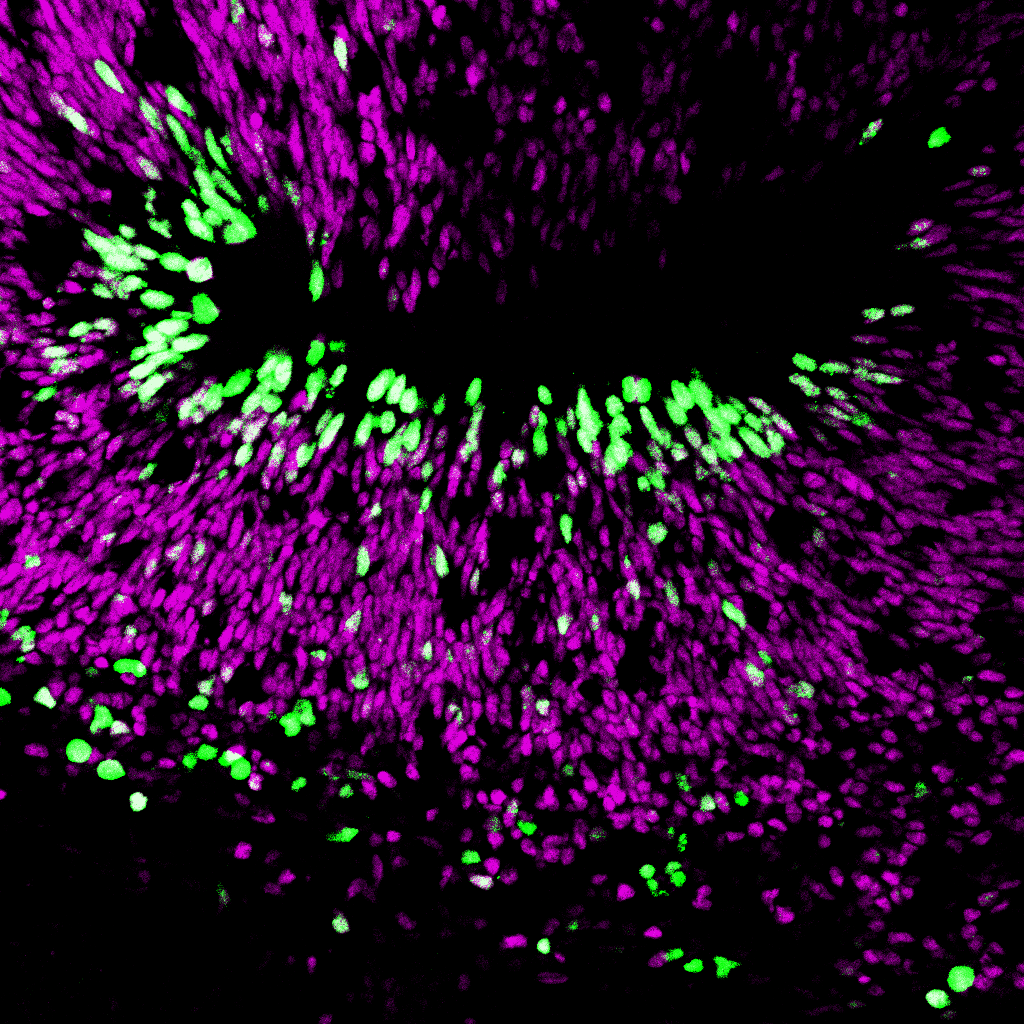

Supplement: Supplementary file 5 — Source data Fig. 4 [file 44321_2025_302_MOESM5_ESM.zip › Figure 4/4F/#12-3-merge.tif]

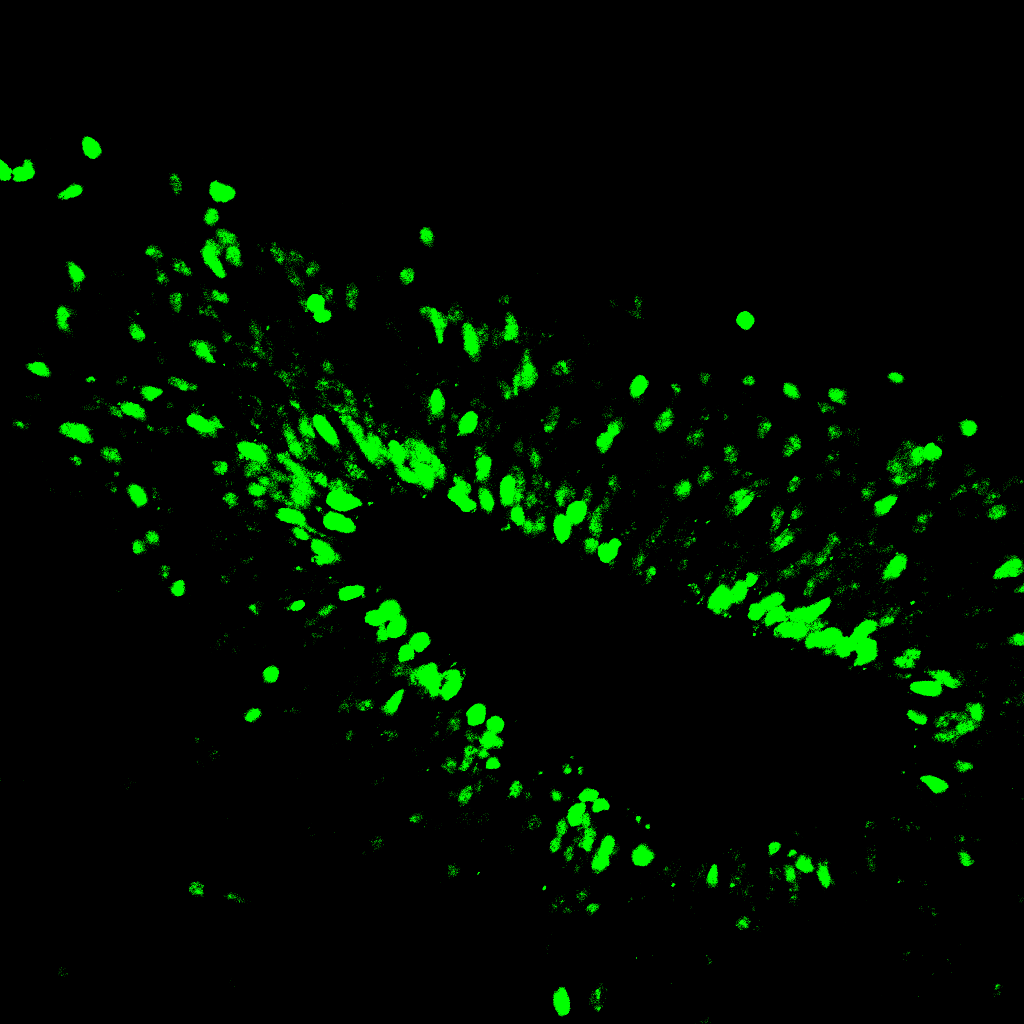

Supplement: Supplementary file 5 — Source data Fig. 4 [file 44321_2025_302_MOESM5_ESM.zip › Figure 4/4F/#7-5-Ki67.tif]

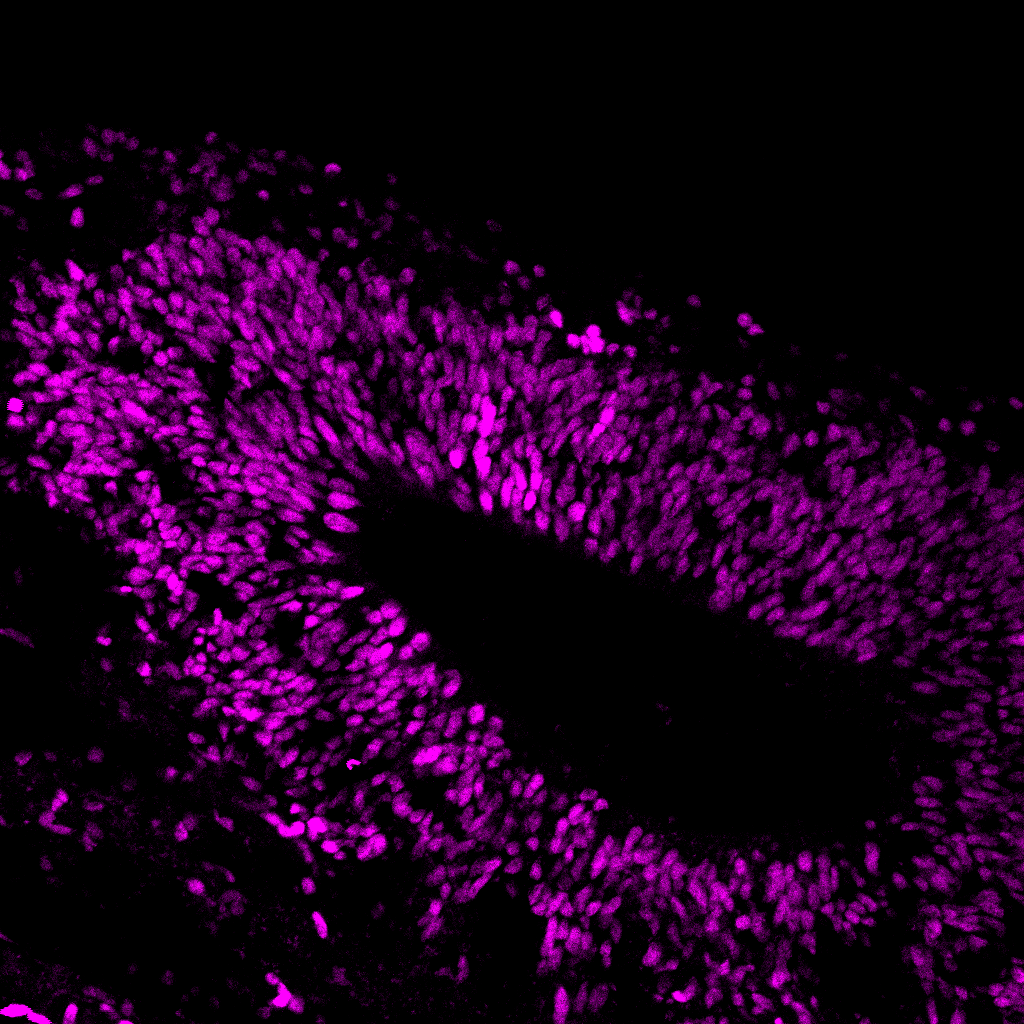

Supplement: Supplementary file 5 — Source data Fig. 4 [file 44321_2025_302_MOESM5_ESM.zip › Figure 4/4F/#7-5-PAX6.tif]

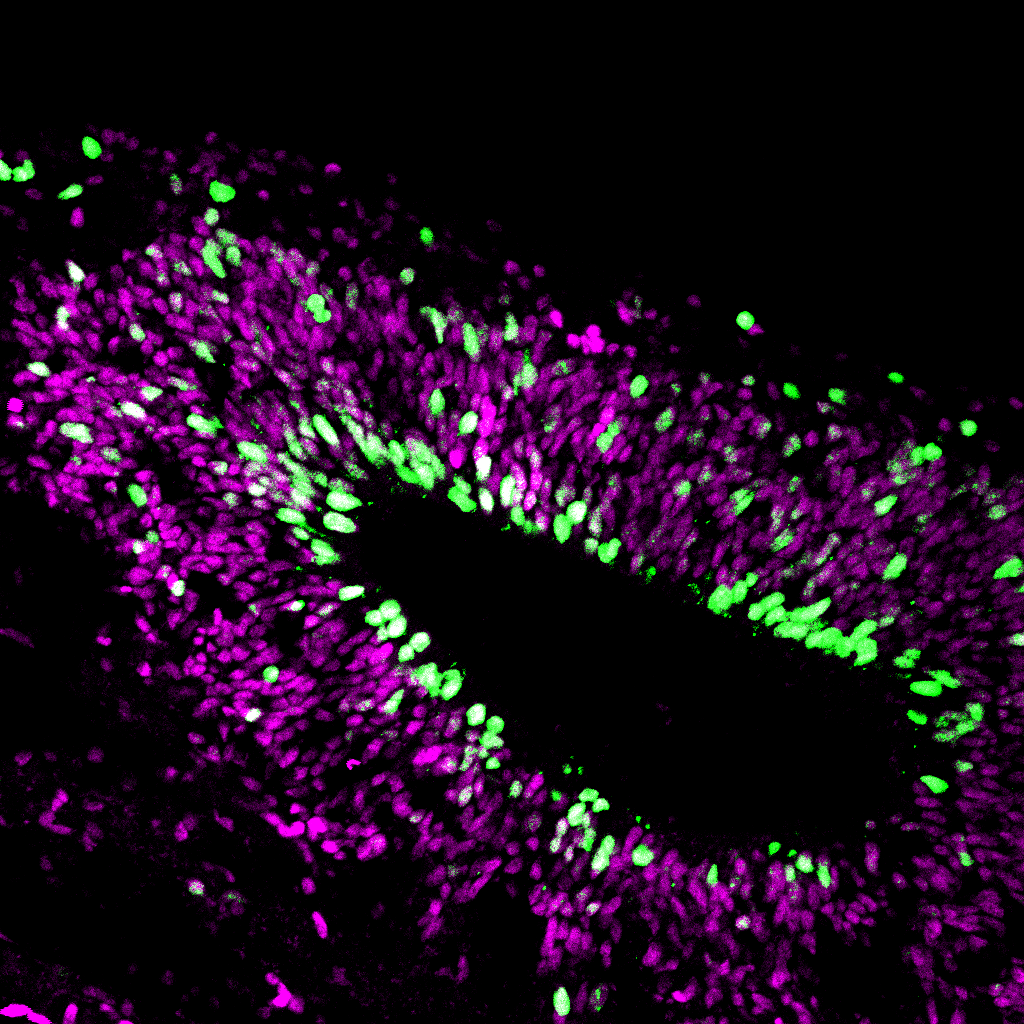

Supplement: Supplementary file 5 — Source data Fig. 4 [file 44321_2025_302_MOESM5_ESM.zip › Figure 4/4F/#7-5-merge.tif]

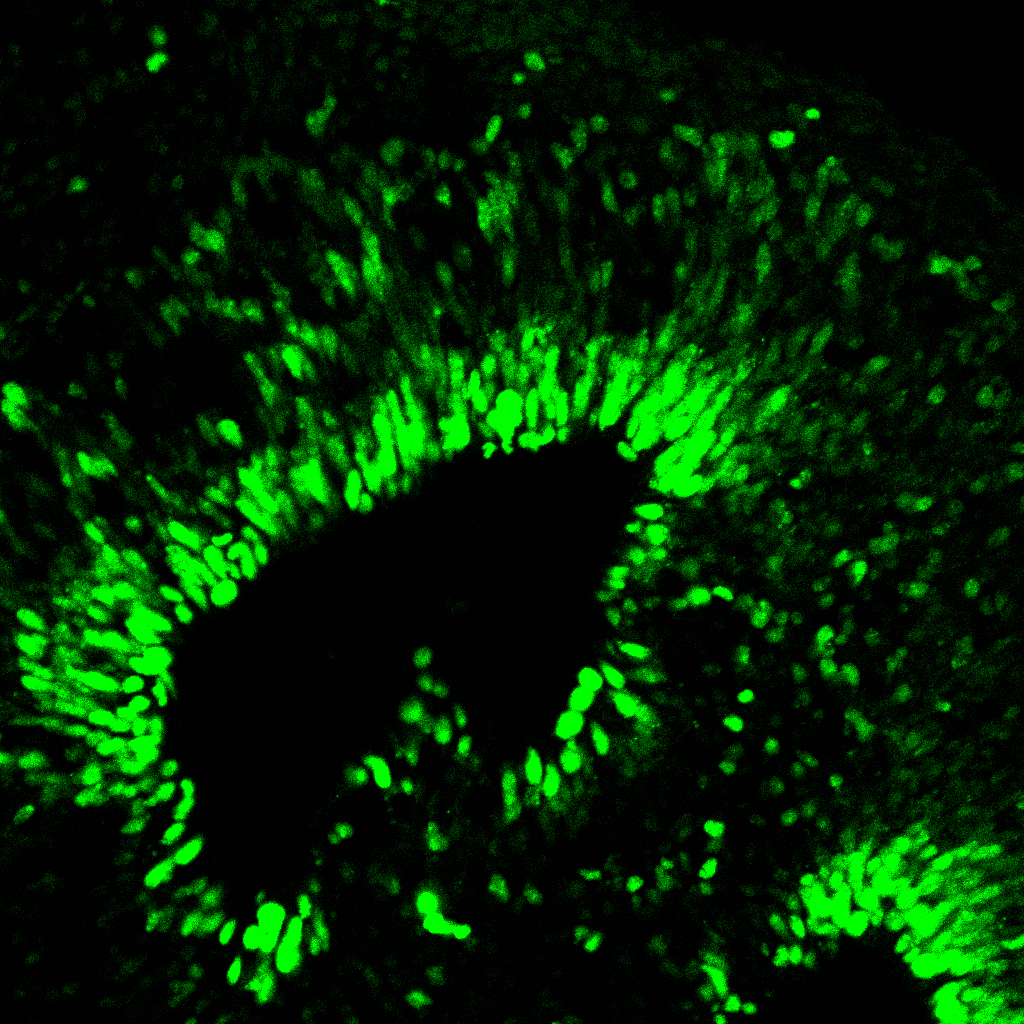

Supplement: Supplementary file 5 — Source data Fig. 4 [file 44321_2025_302_MOESM5_ESM.zip › Figure 4/4F/H9-Ki67.tif]

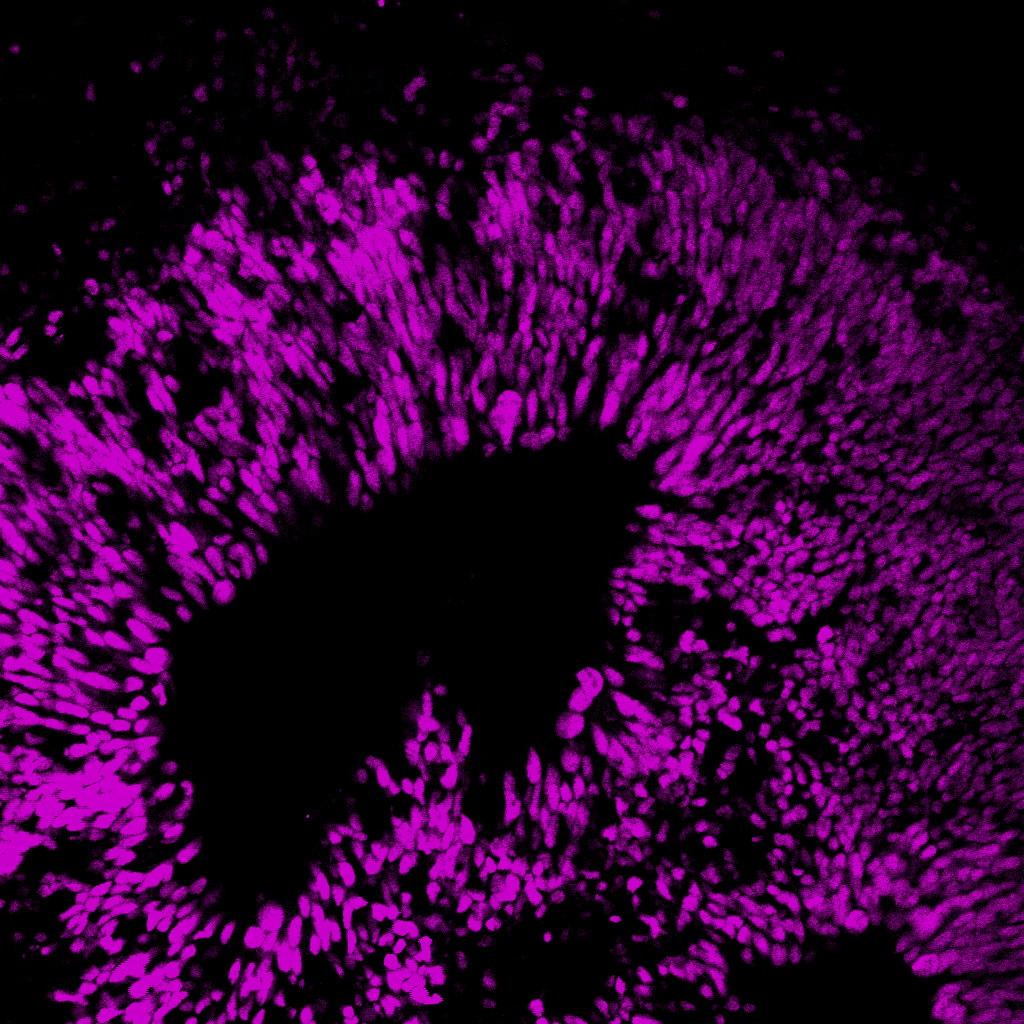

Supplement: Supplementary file 5 — Source data Fig. 4 [file 44321_2025_302_MOESM5_ESM.zip › Figure 4/4F/H9-PAX6.tif]

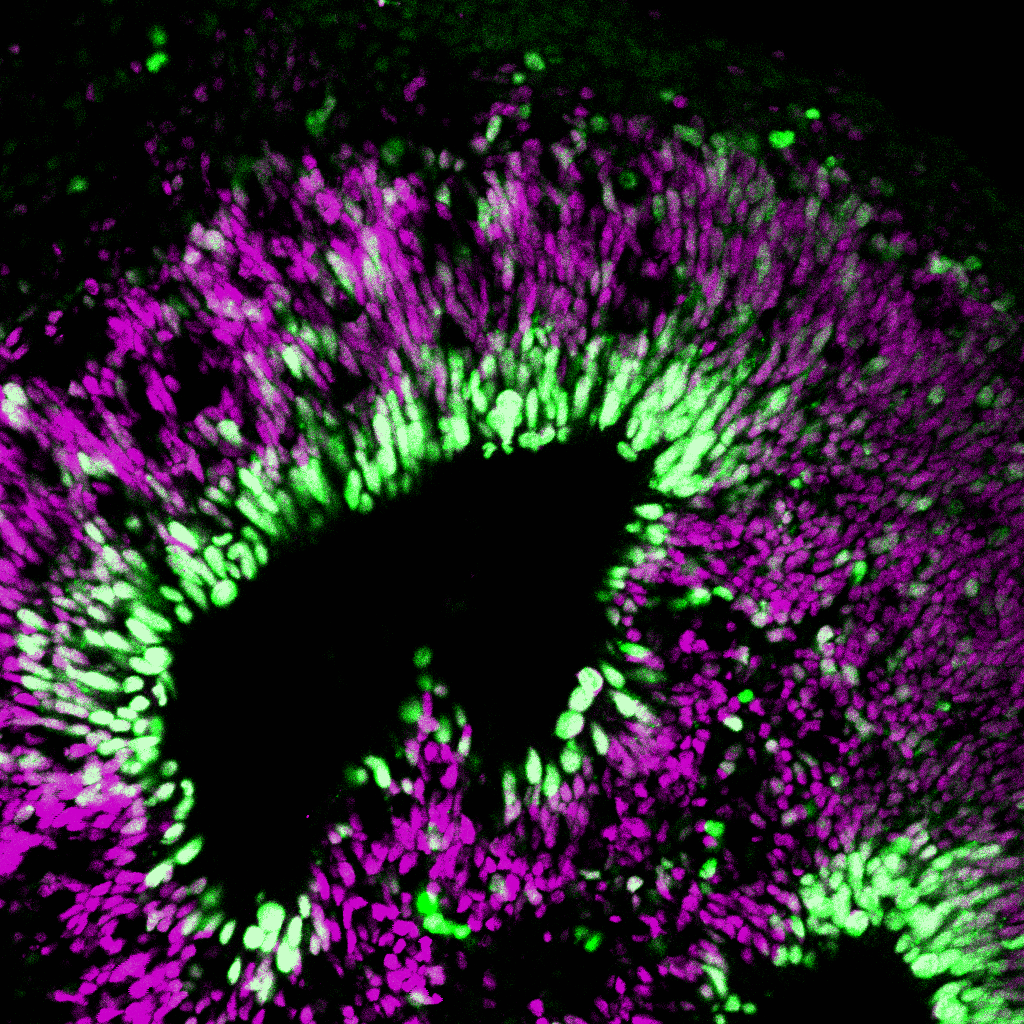

Supplement: Supplementary file 5 — Source data Fig. 4 [file 44321_2025_302_MOESM5_ESM.zip › Figure 4/4F/H9-merge.tif]

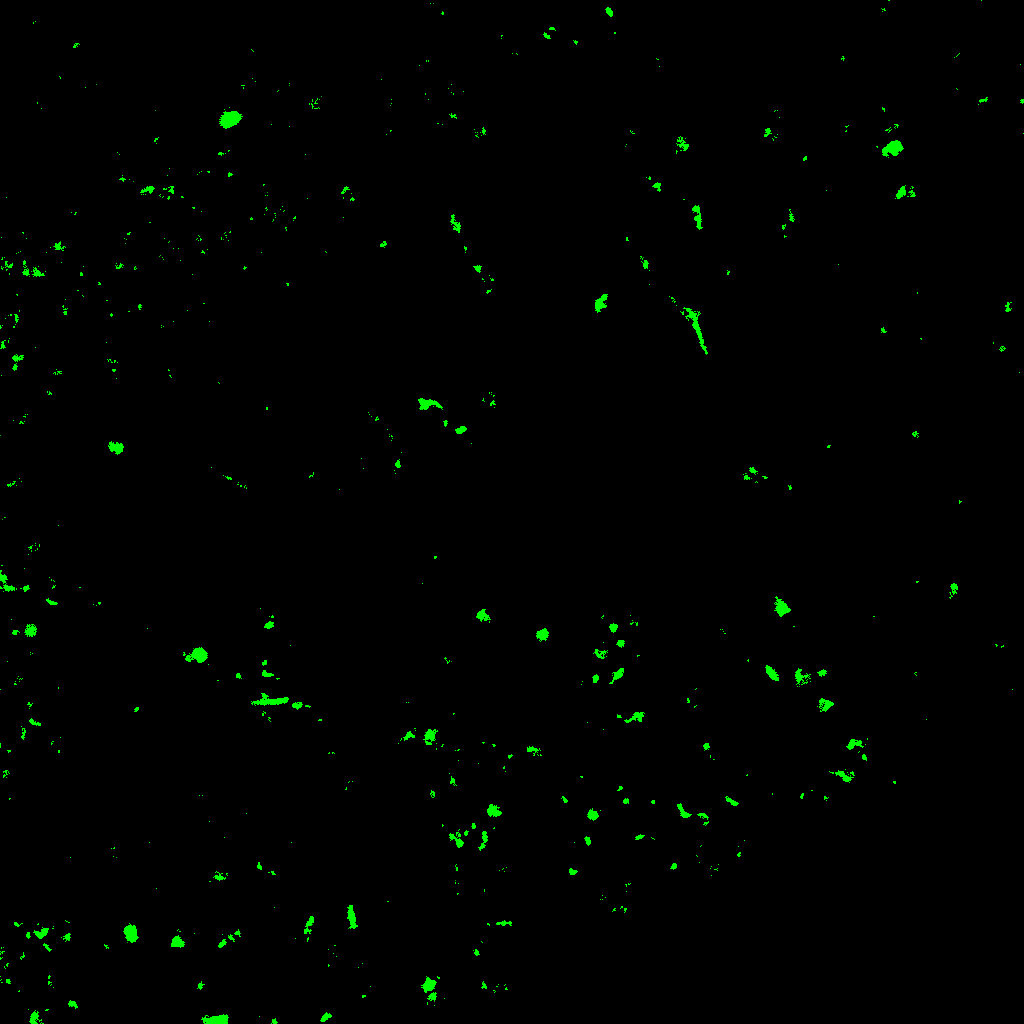

Supplement: Supplementary file 5 — Source data Fig. 4 [file 44321_2025_302_MOESM5_ESM.zip › Figure 4/4G/#12-3-Caspase3.tif]

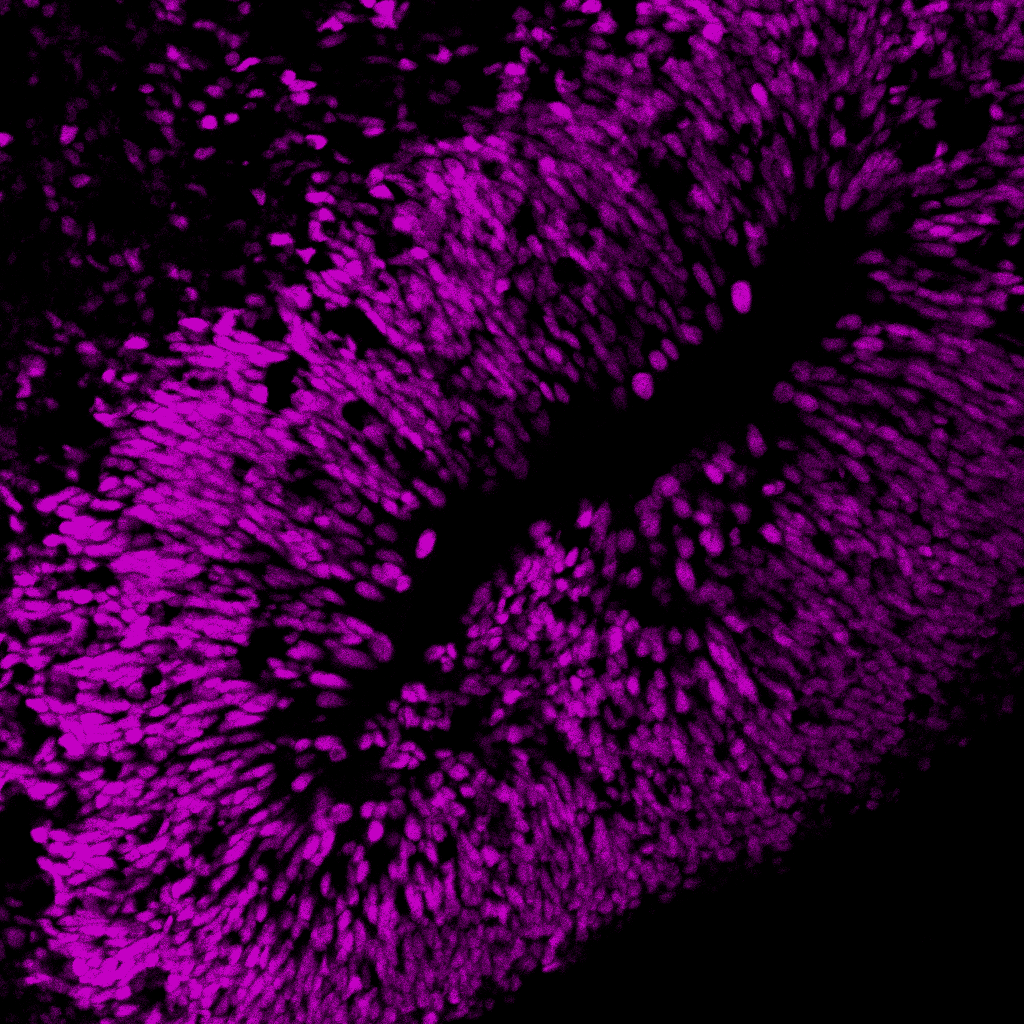

Supplement: Supplementary file 5 — Source data Fig. 4 [file 44321_2025_302_MOESM5_ESM.zip › Figure 4/4G/#12-3-PAX6.tif]

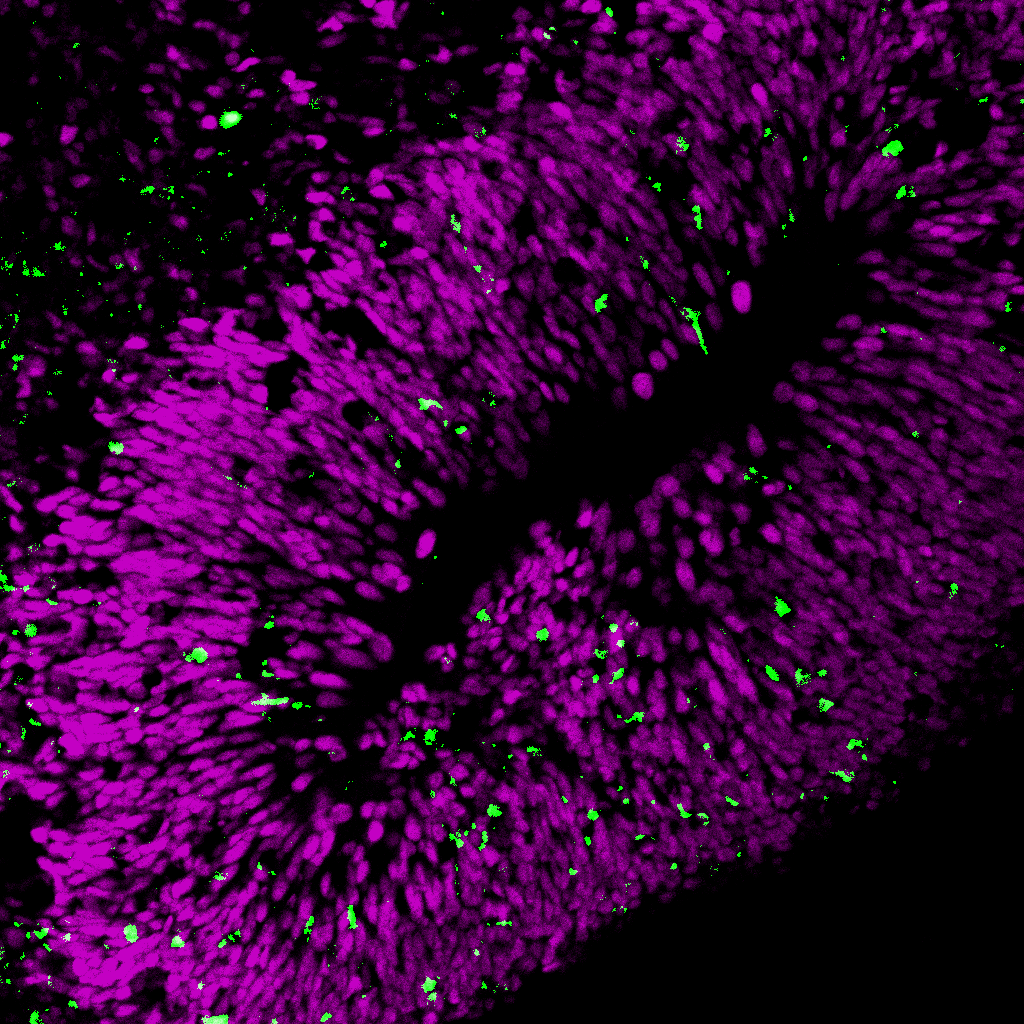

Supplement: Supplementary file 5 — Source data Fig. 4 [file 44321_2025_302_MOESM5_ESM.zip › Figure 4/4G/#12-3-merge.tif]

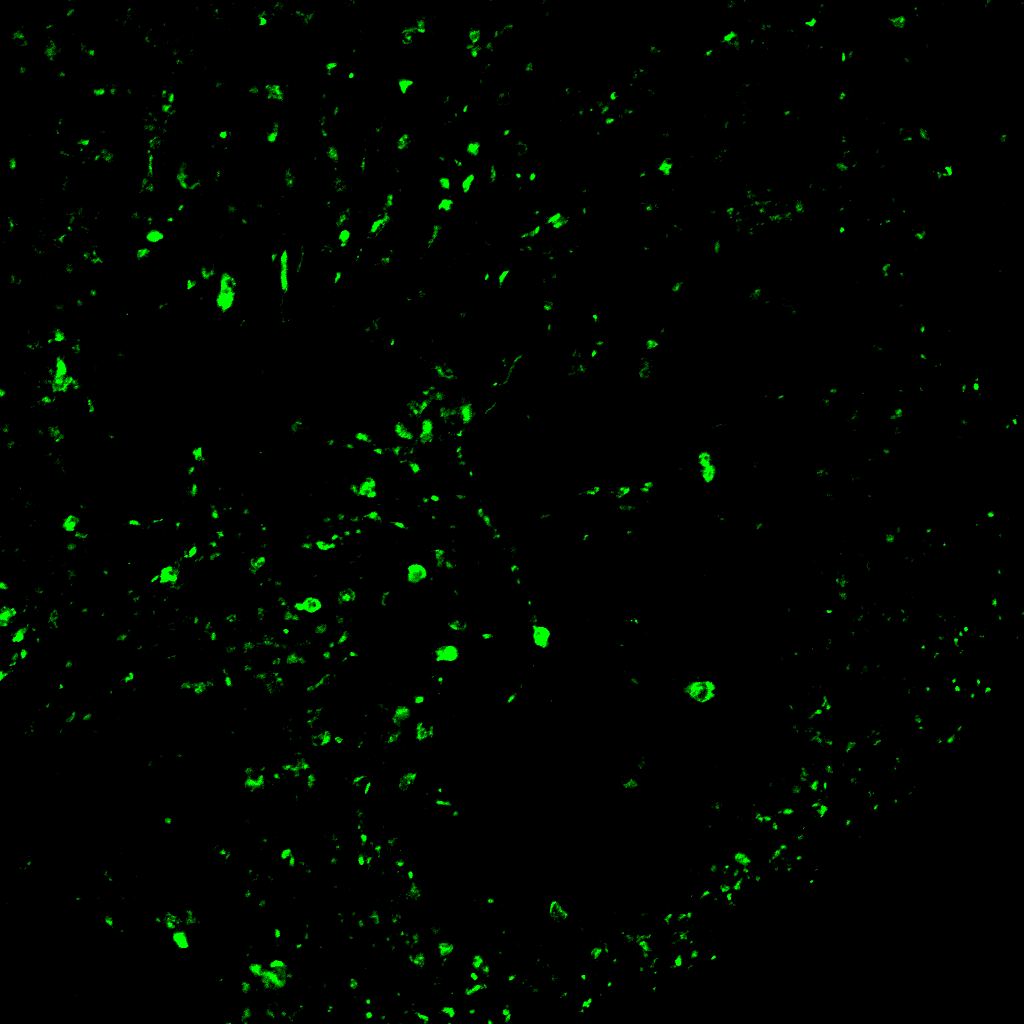

Supplement: Supplementary file 5 — Source data Fig. 4 [file 44321_2025_302_MOESM5_ESM.zip › Figure 4/4G/#7-5-Caspase3.tif]

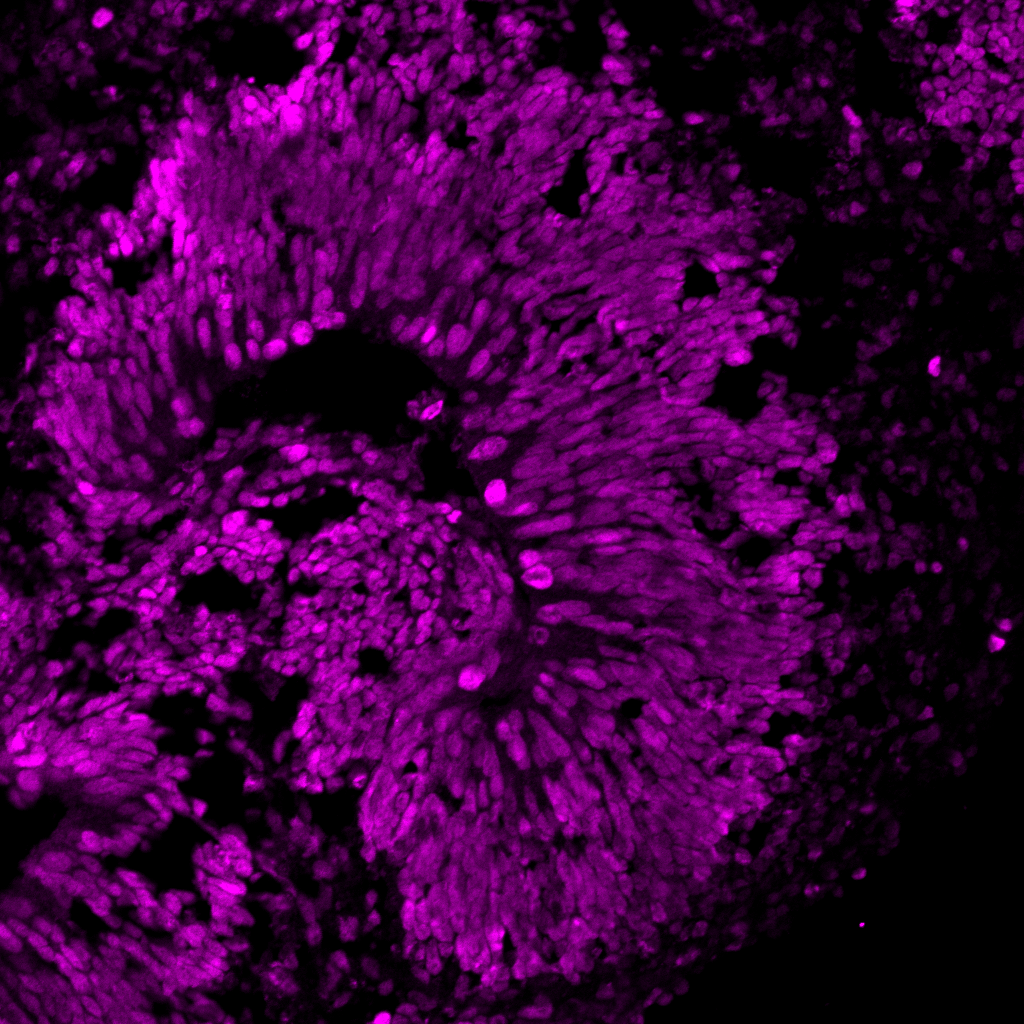

Supplement: Supplementary file 5 — Source data Fig. 4 [file 44321_2025_302_MOESM5_ESM.zip › Figure 4/4G/#7-5-PAX6.tif]

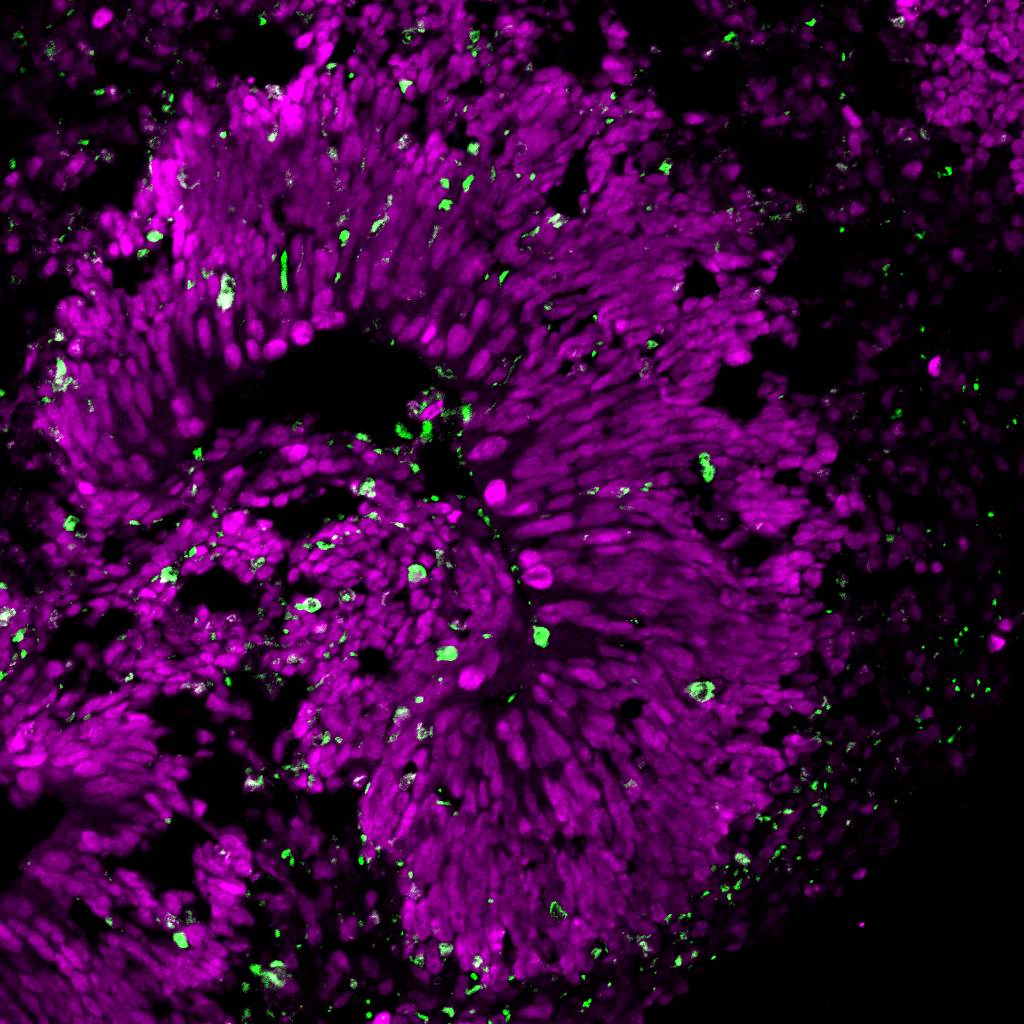

Supplement: Supplementary file 5 — Source data Fig. 4 [file 44321_2025_302_MOESM5_ESM.zip › Figure 4/4G/#7-5-merge.tif]

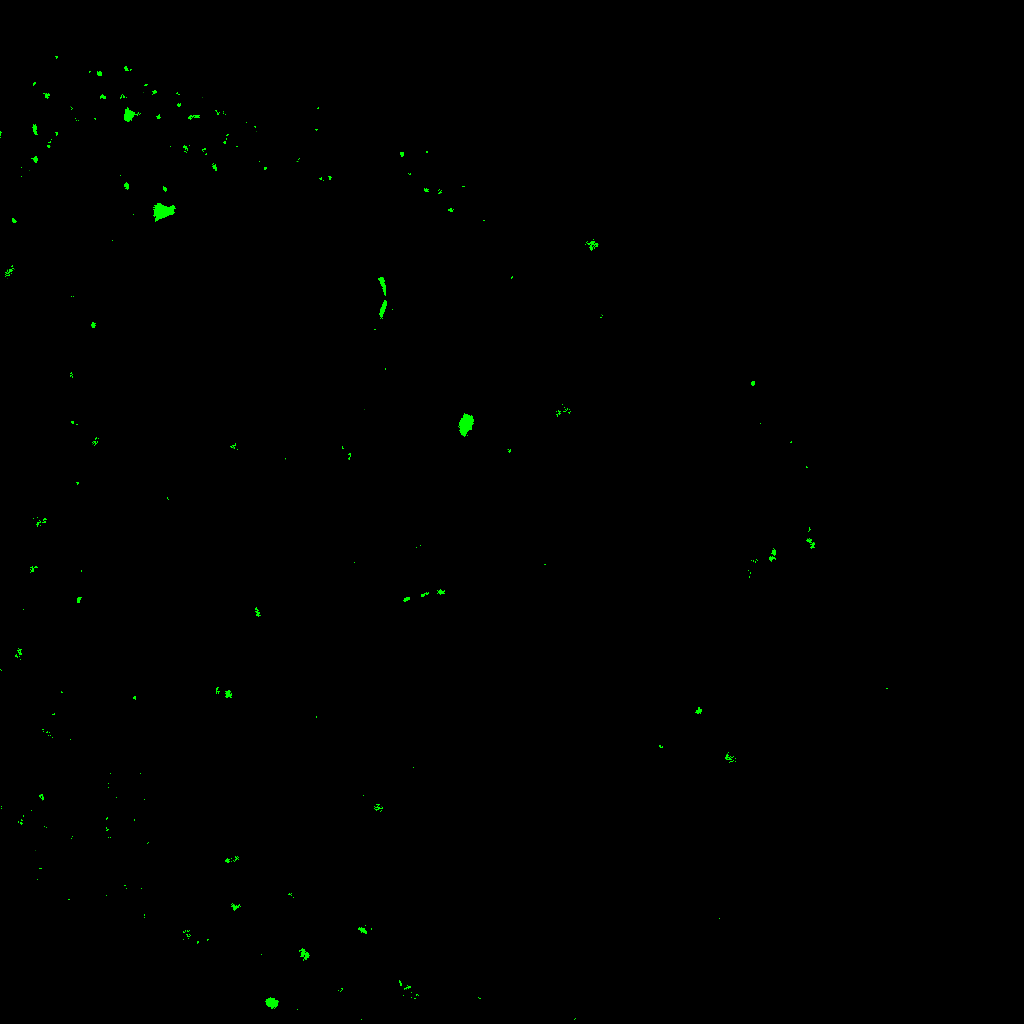

Supplement: Supplementary file 5 — Source data Fig. 4 [file 44321_2025_302_MOESM5_ESM.zip › Figure 4/4G/H9-Caspase3.tif]

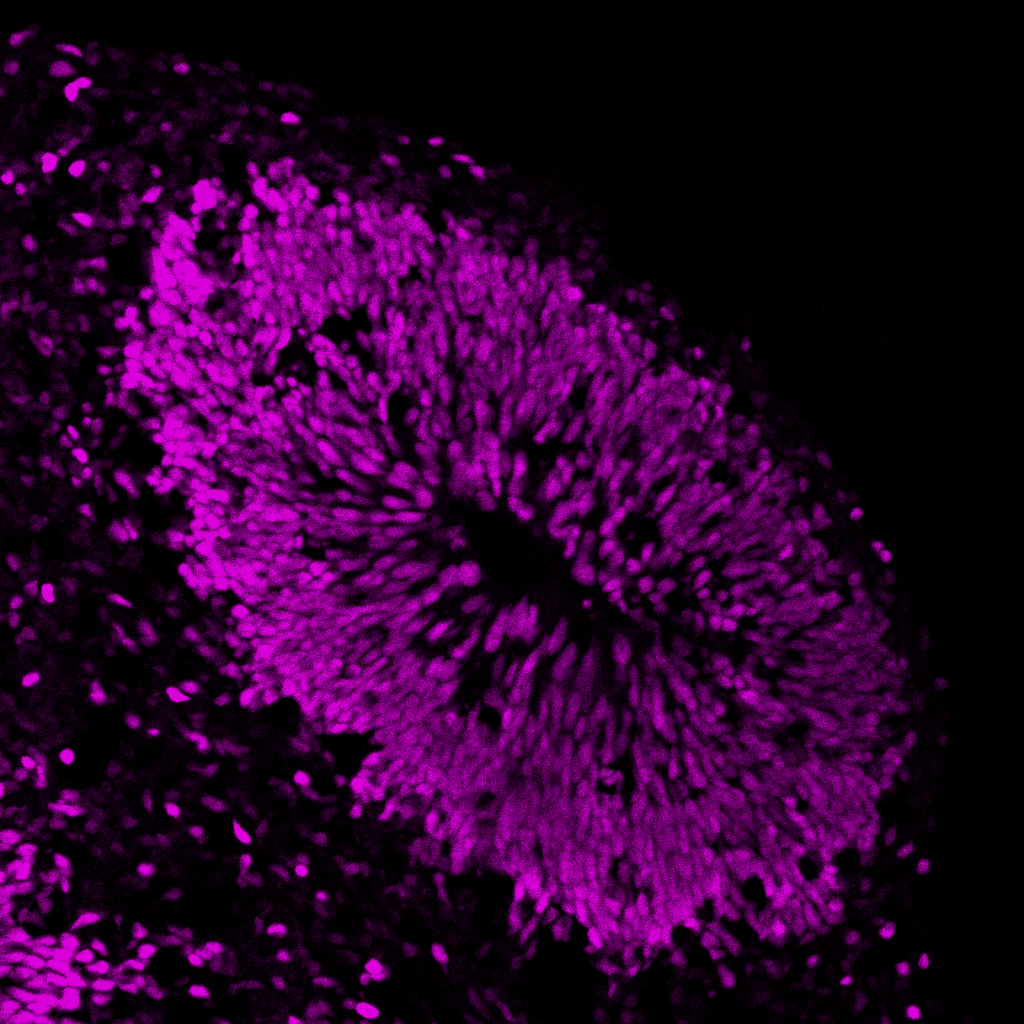

Supplement: Supplementary file 5 — Source data Fig. 4 [file 44321_2025_302_MOESM5_ESM.zip › Figure 4/4G/H9-PAX6.tif]

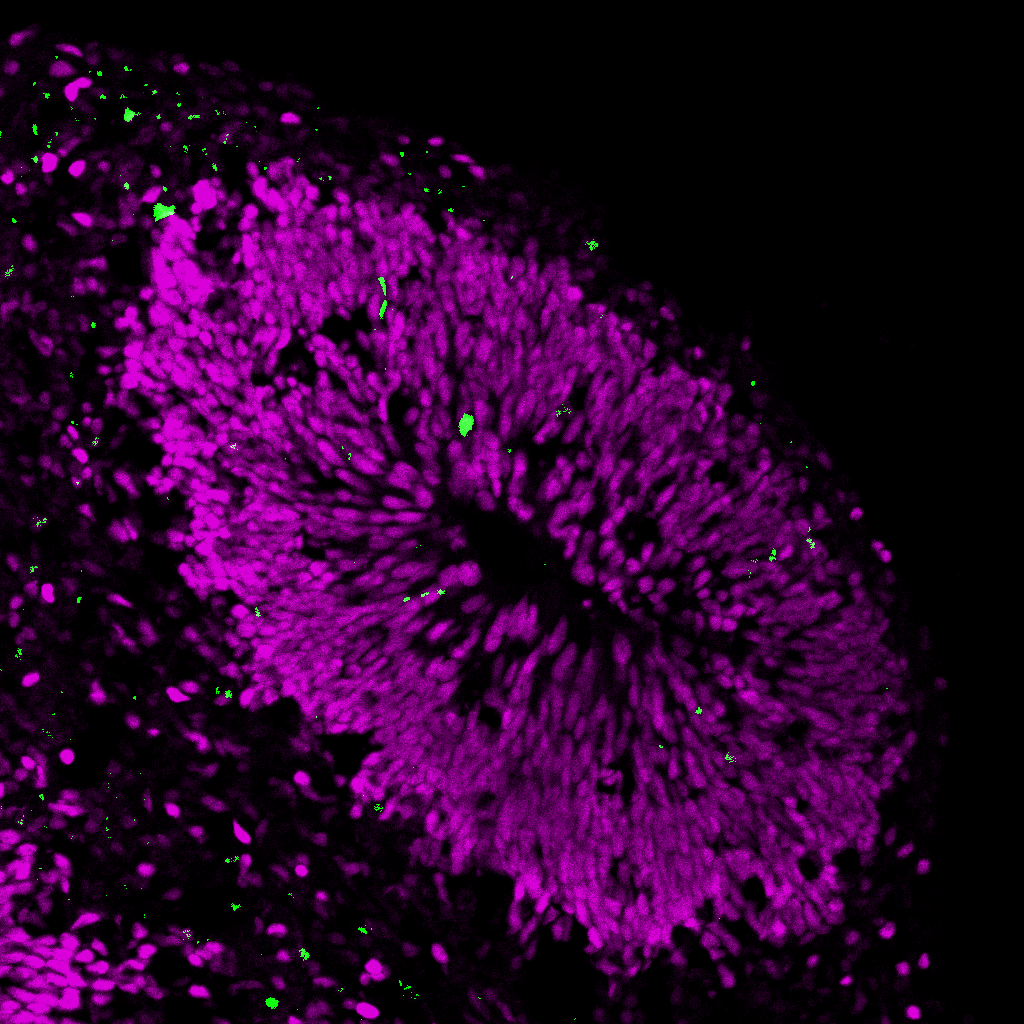

Supplement: Supplementary file 5 — Source data Fig. 4 [file 44321_2025_302_MOESM5_ESM.zip › Figure 4/4G/H9-merge.tif]

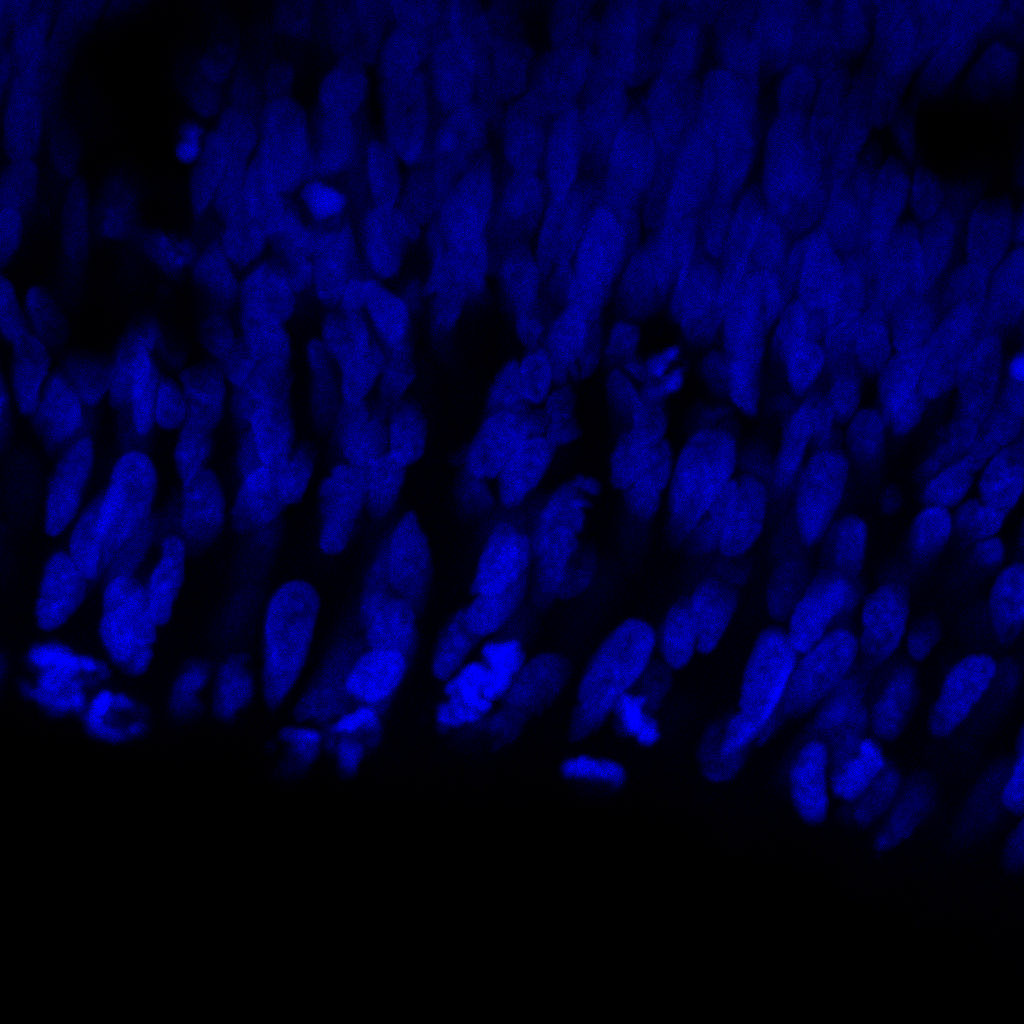

Supplement: Supplementary file 6 — Source data Fig. 5 [file 44321_2025_302_MOESM6_ESM.zip › Figure 5/5A/#12-3-DAPI.tif]

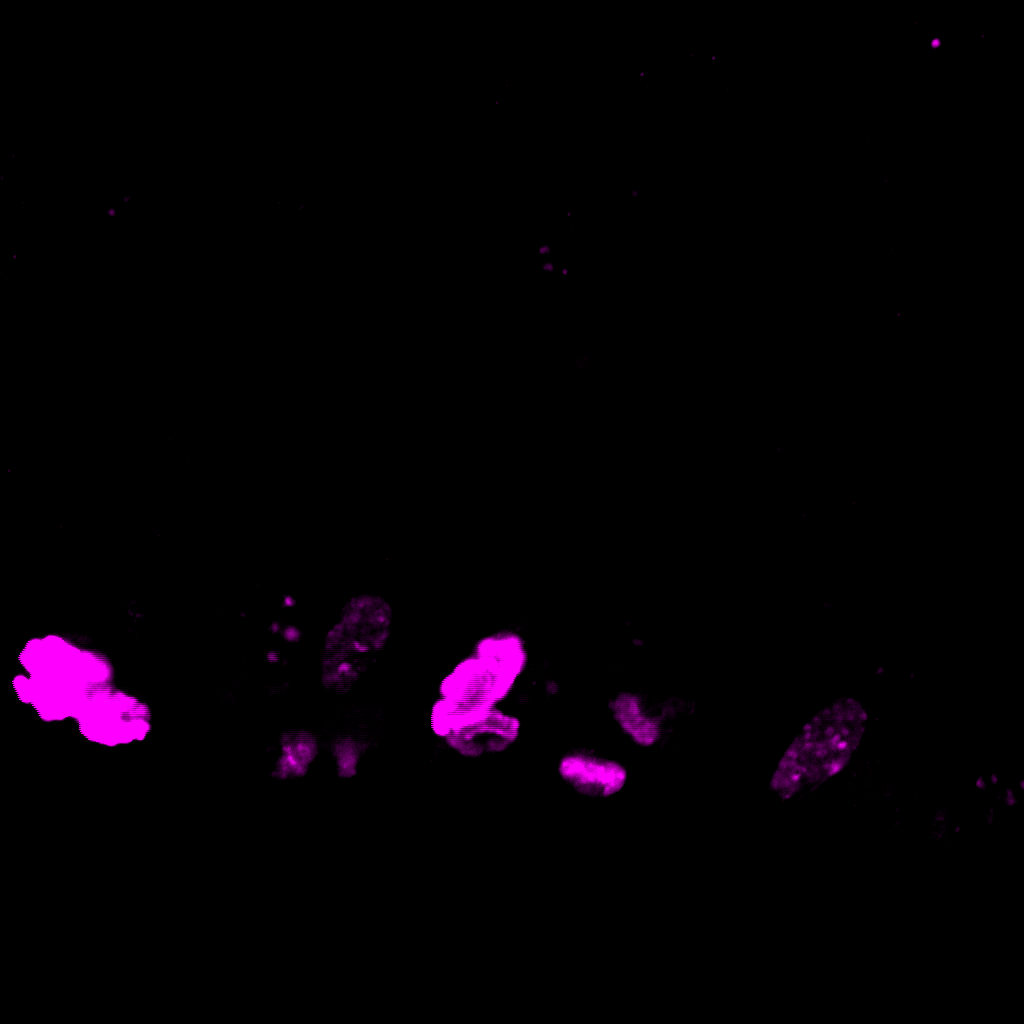

Supplement: Supplementary file 6 — Source data Fig. 5 [file 44321_2025_302_MOESM6_ESM.zip › Figure 5/5A/#12-3-PH3.tif]

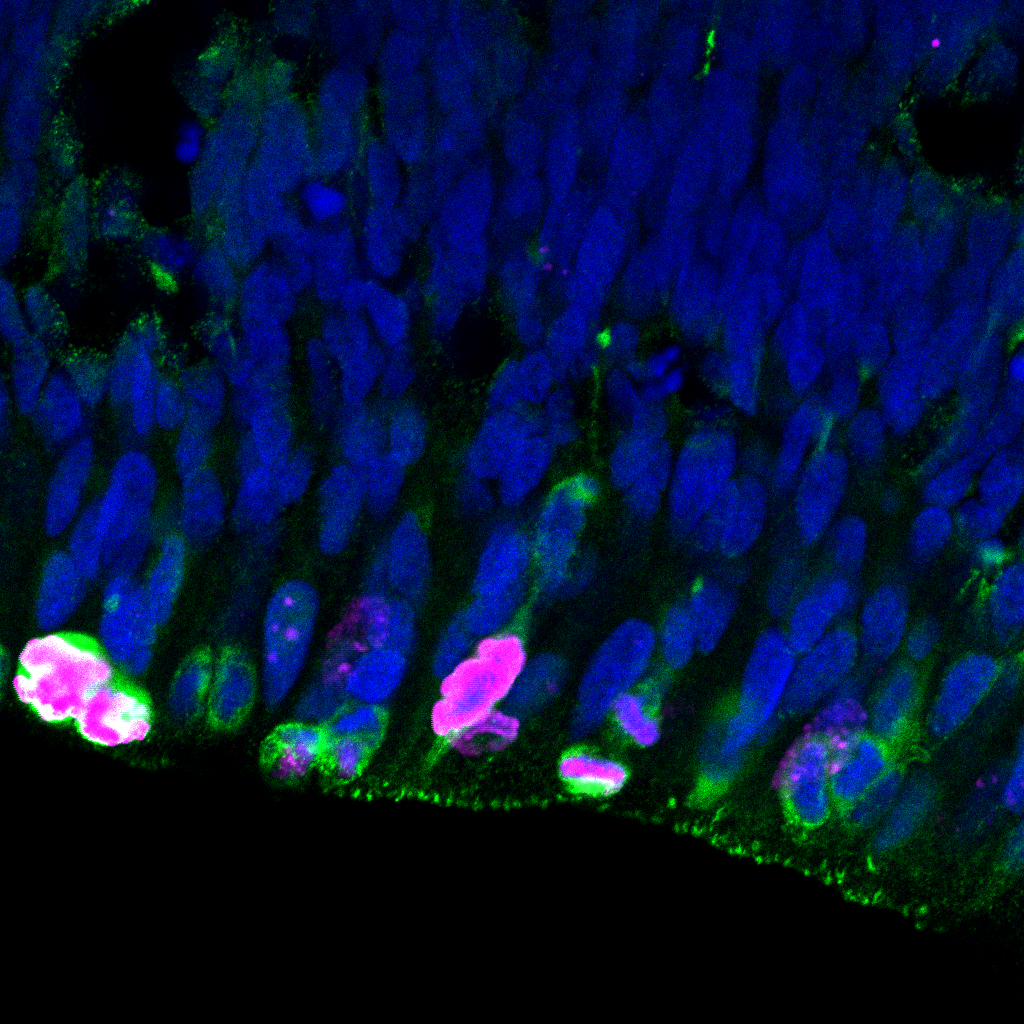

Supplement: Supplementary file 6 — Source data Fig. 5 [file 44321_2025_302_MOESM6_ESM.zip › Figure 5/5A/#12-3-merge.tif]

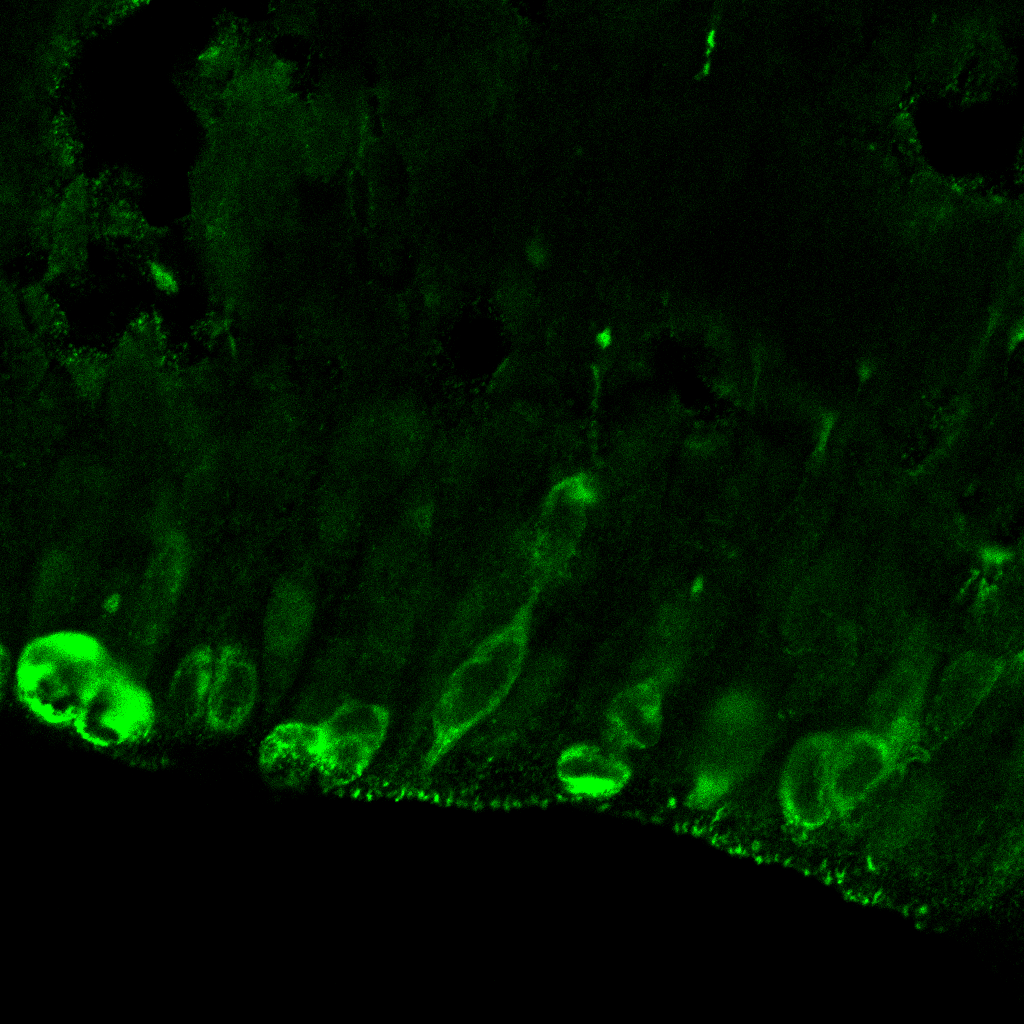

Supplement: Supplementary file 6 — Source data Fig. 5 [file 44321_2025_302_MOESM6_ESM.zip › Figure 5/5A/#12-3-pVimentin.tif]

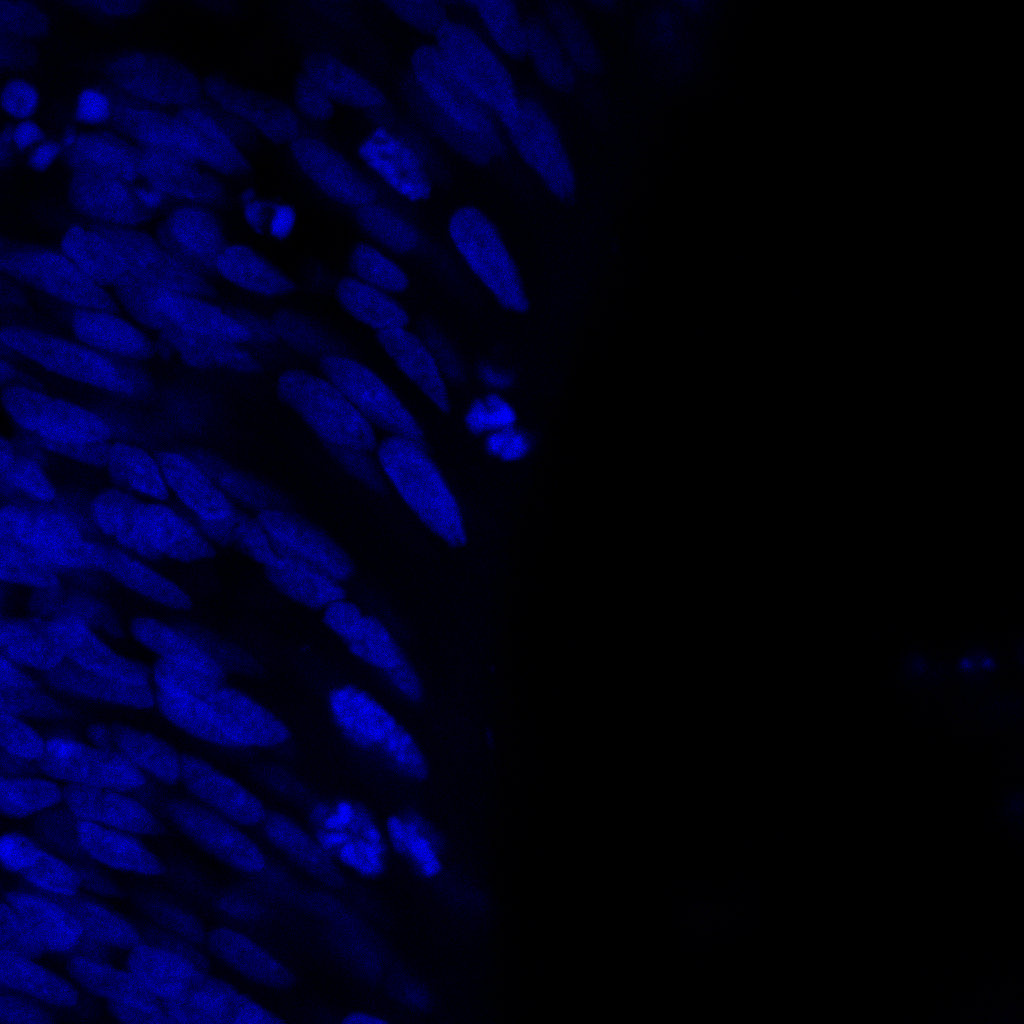

Supplement: Supplementary file 6 — Source data Fig. 5 [file 44321_2025_302_MOESM6_ESM.zip › Figure 5/5A/#7-5-DAPI.tif]

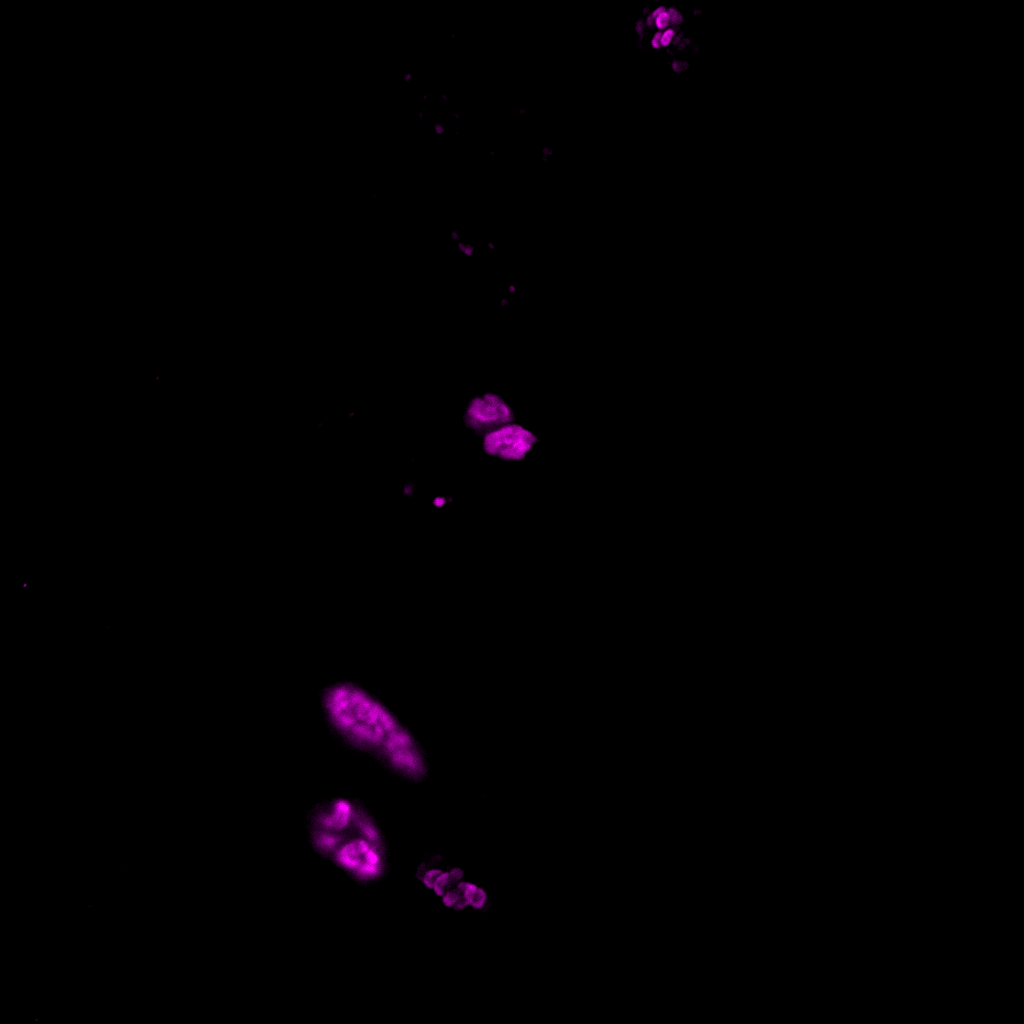

Supplement: Supplementary file 6 — Source data Fig. 5 [file 44321_2025_302_MOESM6_ESM.zip › Figure 5/5A/#7-5-PH3.tif]
