# Supplementary figures and images for: CETN3 deficiency induces microcephaly by disrupting neural stem/progenitor cell fate through impaired centrosome assembly and RNA splicing (part 2 of 5)
Source: EMBO Mol Med. 2025 Sep 8;17(10):2735–61. doi: 10.1038/s44321-025-00302-7 (PMC12514221; doi:10.1038/s44321-025-00302-7)

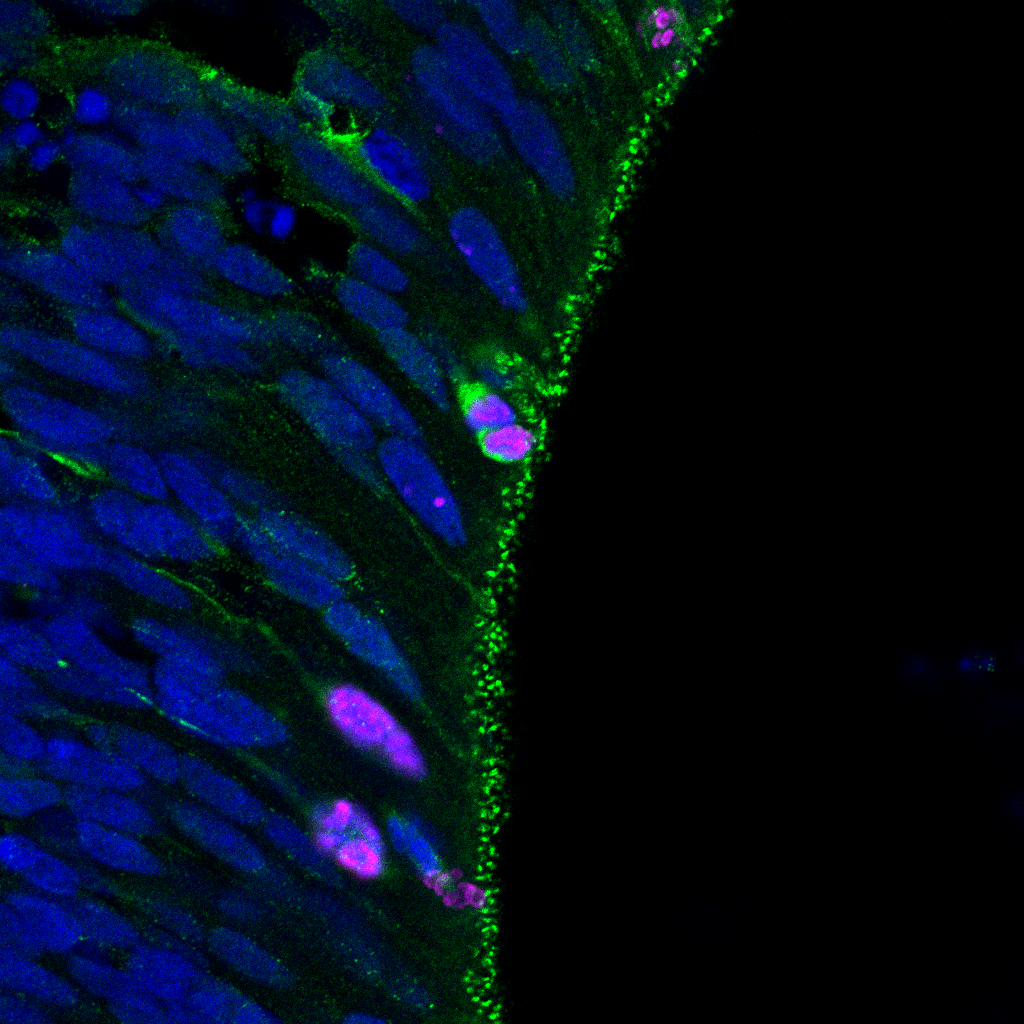

Supplement: Supplementary file 6 — Source data Fig. 5 [file 44321_2025_302_MOESM6_ESM.zip › Figure 5/5A/#7-5-merge.tif]

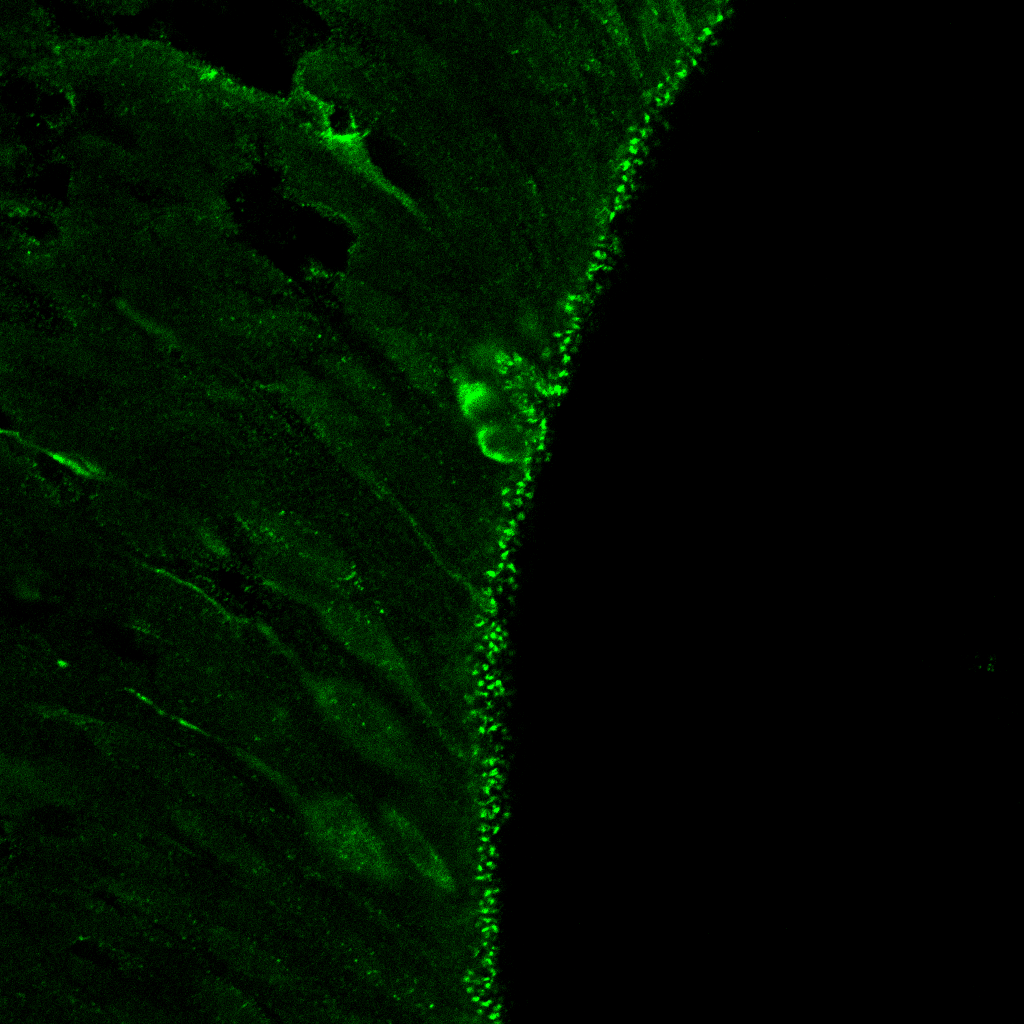

Supplement: Supplementary file 6 — Source data Fig. 5 [file 44321_2025_302_MOESM6_ESM.zip › Figure 5/5A/#7-5-pVimentin.tif]

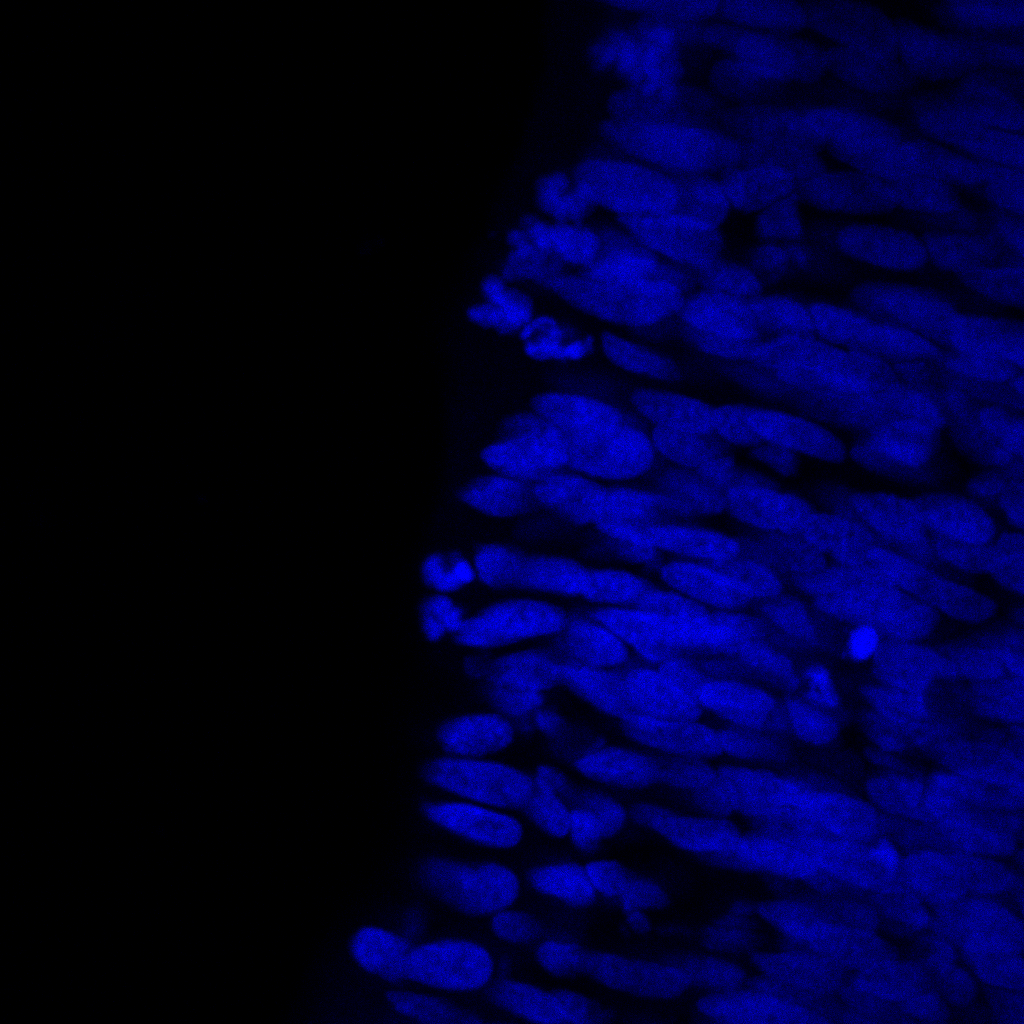

Supplement: Supplementary file 6 — Source data Fig. 5 [file 44321_2025_302_MOESM6_ESM.zip › Figure 5/5A/H9-DAPI.tif]

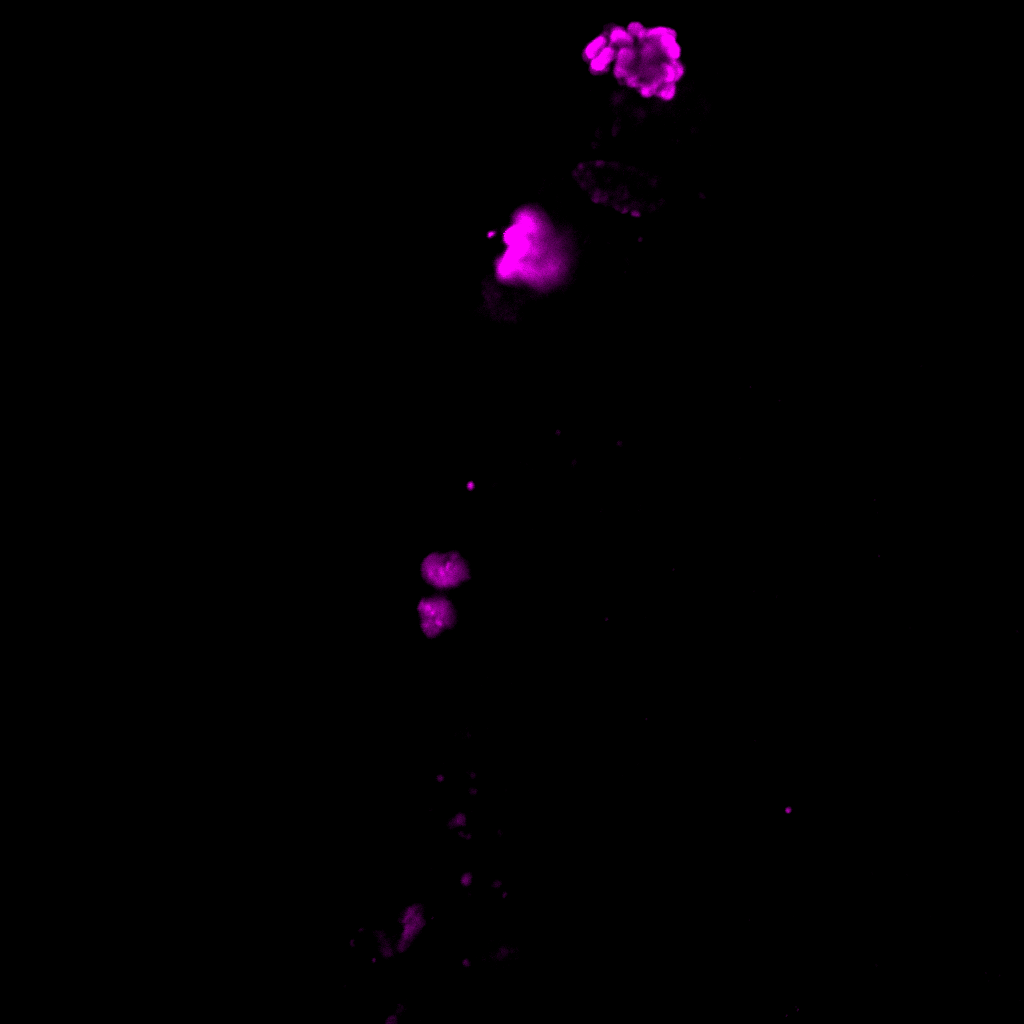

Supplement: Supplementary file 6 — Source data Fig. 5 [file 44321_2025_302_MOESM6_ESM.zip › Figure 5/5A/H9-PH3.tif]

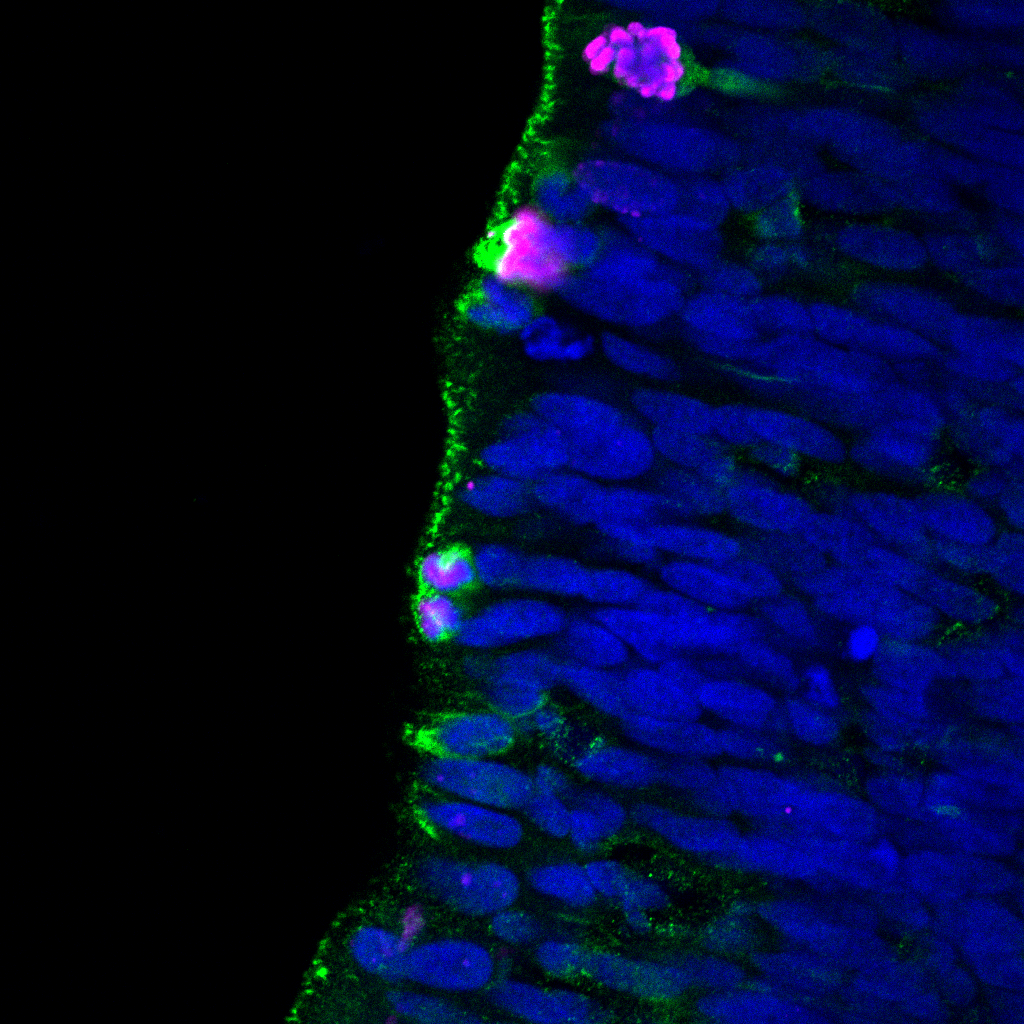

Supplement: Supplementary file 6 — Source data Fig. 5 [file 44321_2025_302_MOESM6_ESM.zip › Figure 5/5A/H9-merge.tif]

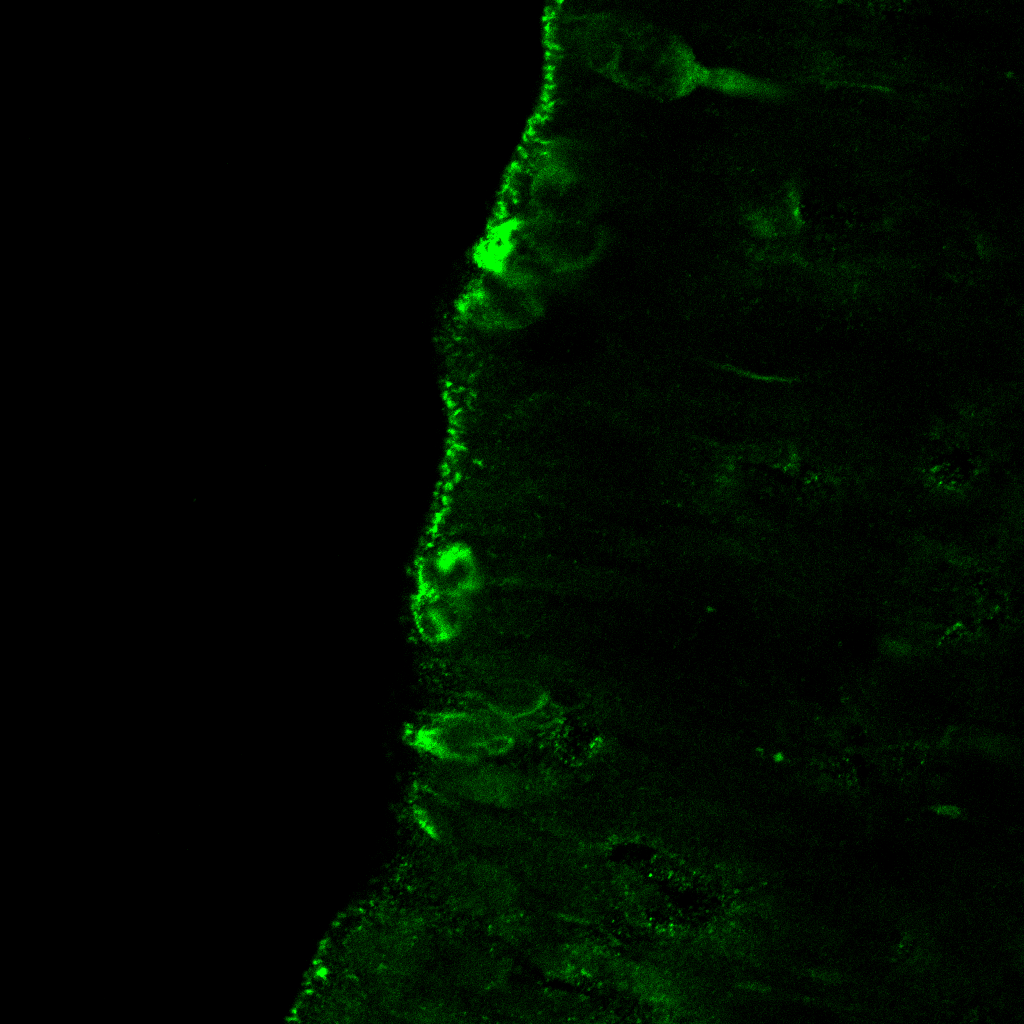

Supplement: Supplementary file 6 — Source data Fig. 5 [file 44321_2025_302_MOESM6_ESM.zip › Figure 5/5A/H9-pVimentin.tif]

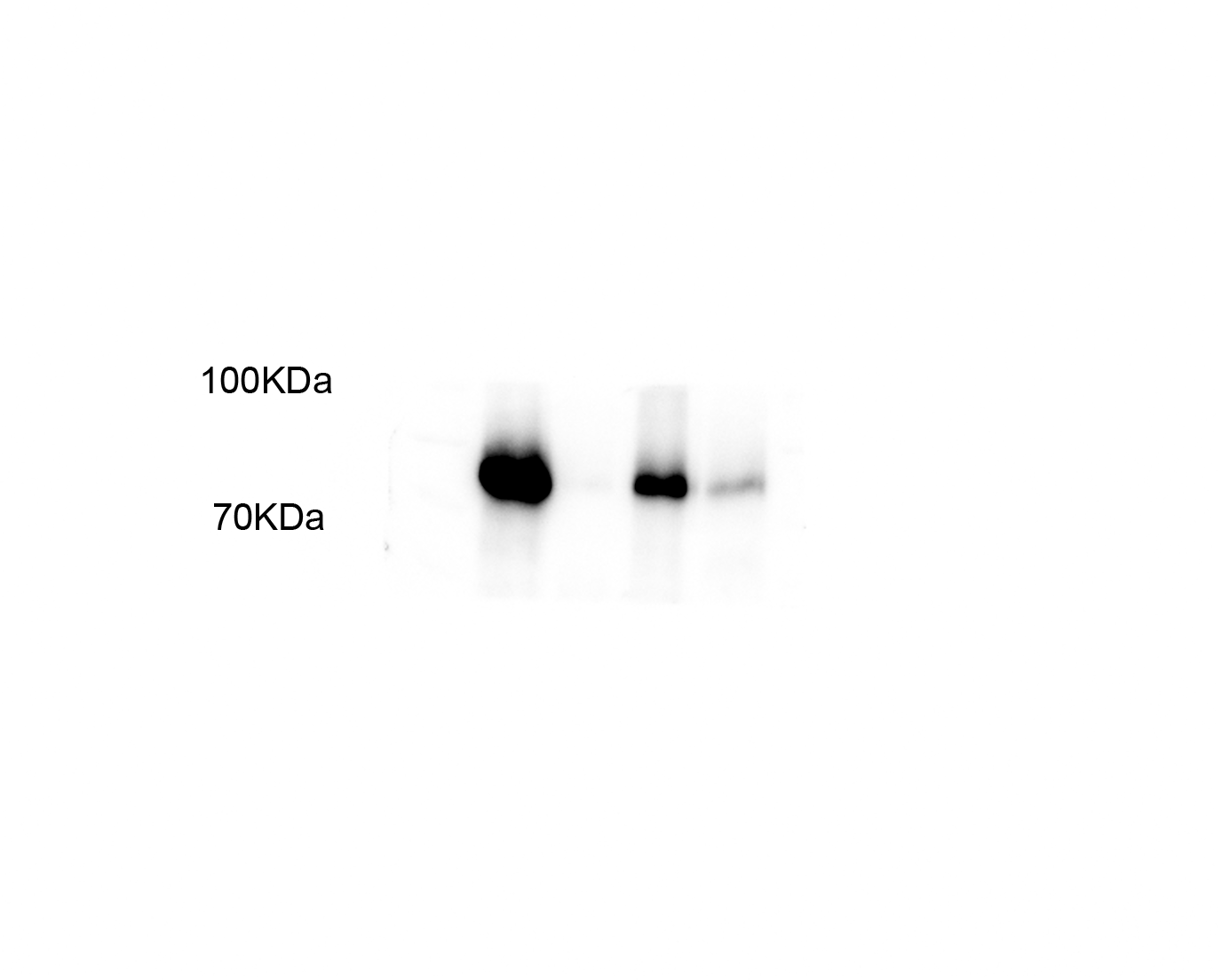

Supplement: Supplementary file 6 — Source data Fig. 5 [file 44321_2025_302_MOESM6_ESM.zip › Figure 5/5G/blot-flag.Tif]

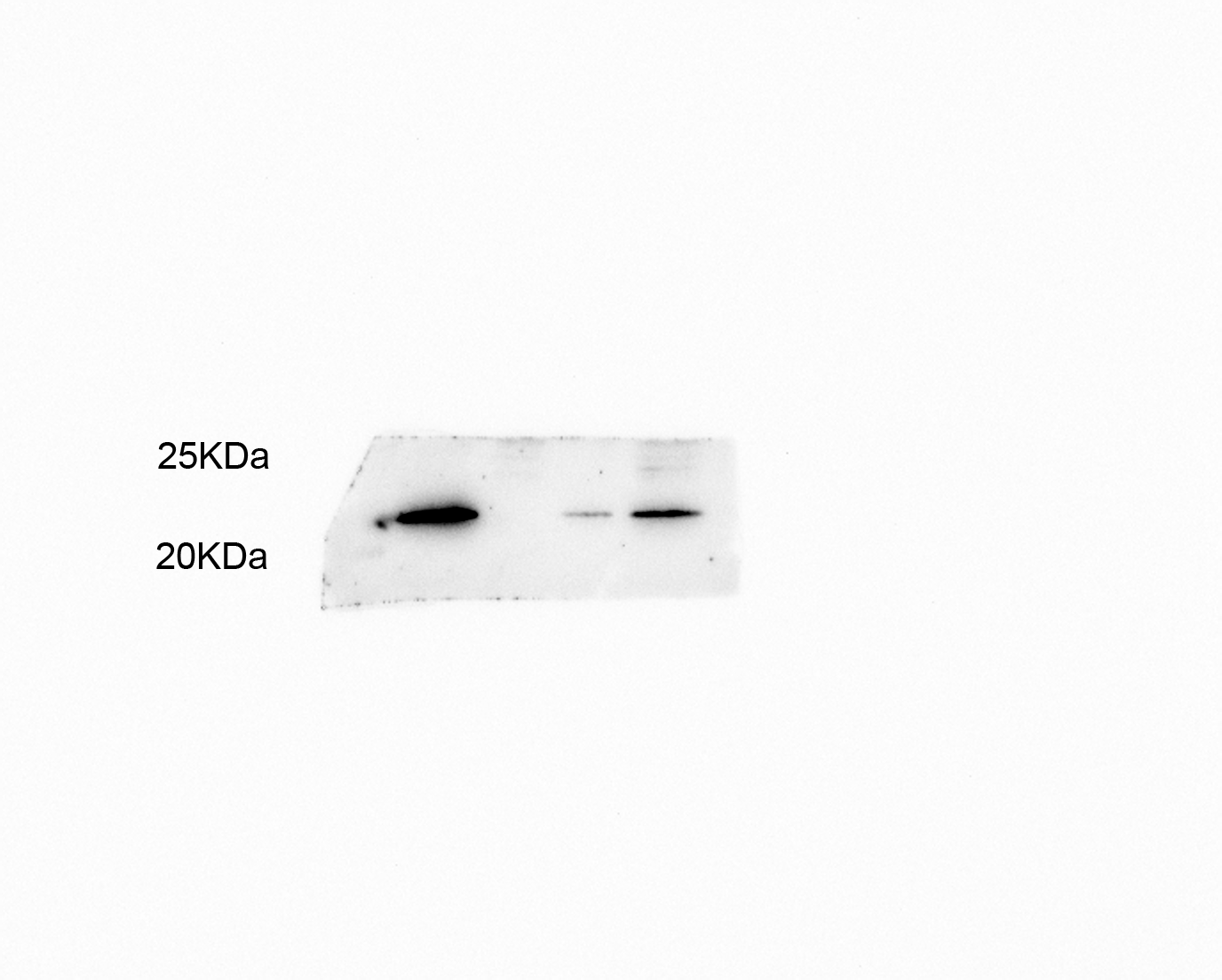

Supplement: Supplementary file 6 — Source data Fig. 5 [file 44321_2025_302_MOESM6_ESM.zip › Figure 5/5G/blot-myc.Tif]

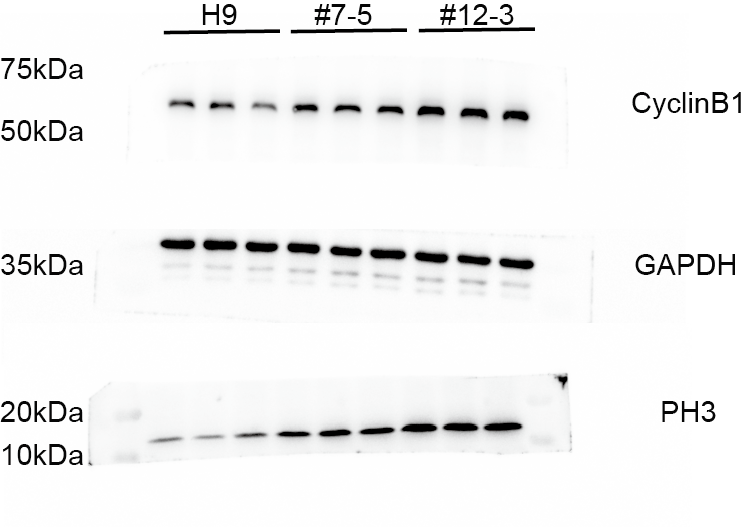

Supplement: Supplementary file 6 — Source data Fig. 5 [file 44321_2025_302_MOESM6_ESM.zip › Figure 5/5H/western.png]

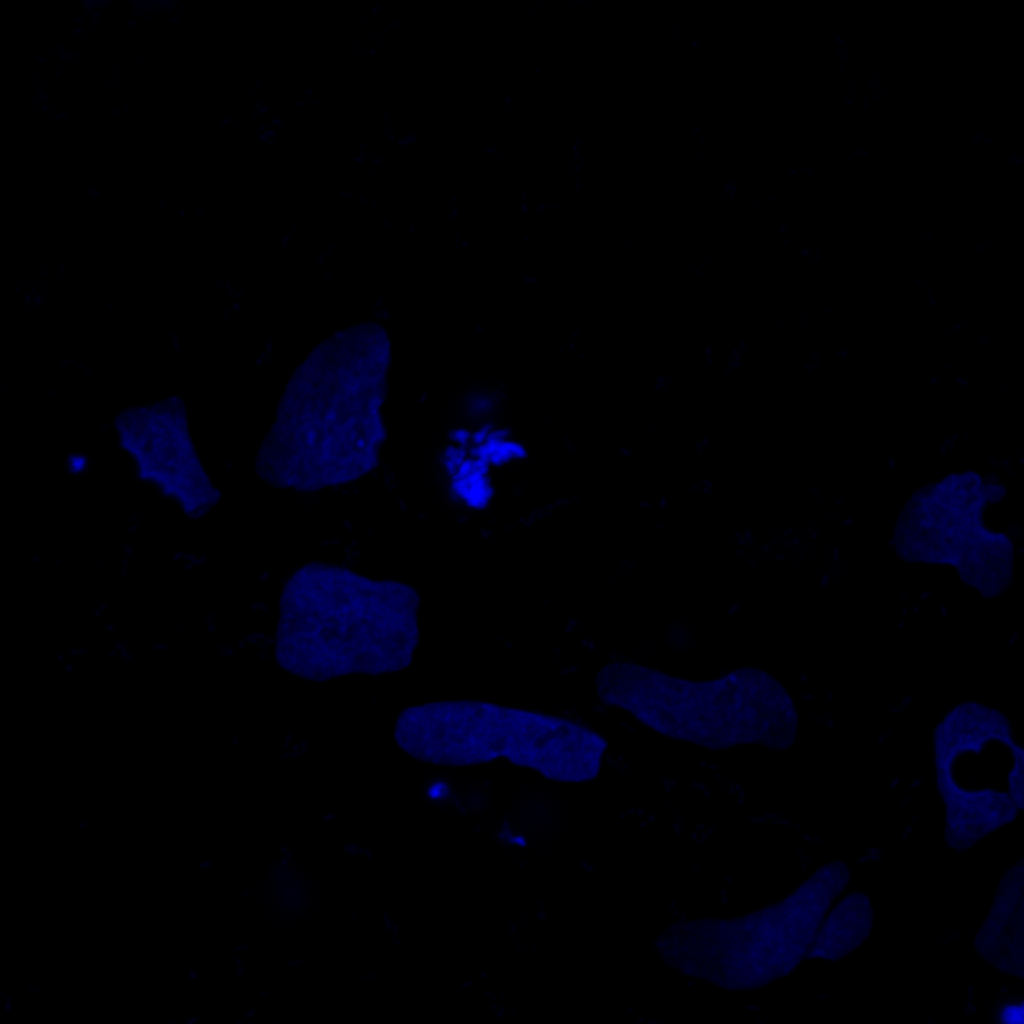

Supplement: Supplementary file 6 — Source data Fig. 5 [file 44321_2025_302_MOESM6_ESM.zip › Figure 5/5K/#12-3-DAPI.tif]

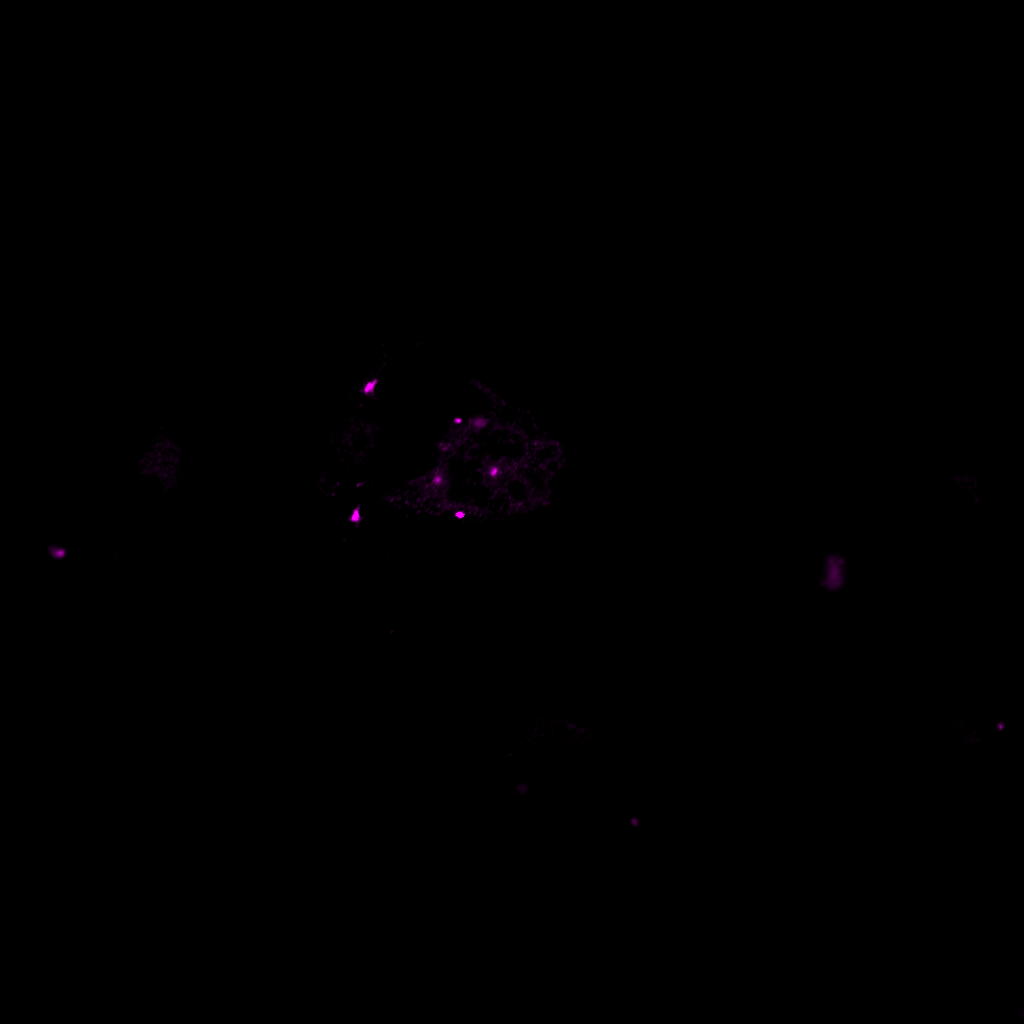

Supplement: Supplementary file 6 — Source data Fig. 5 [file 44321_2025_302_MOESM6_ESM.zip › Figure 5/5K/#12-3-PCNT.tif]

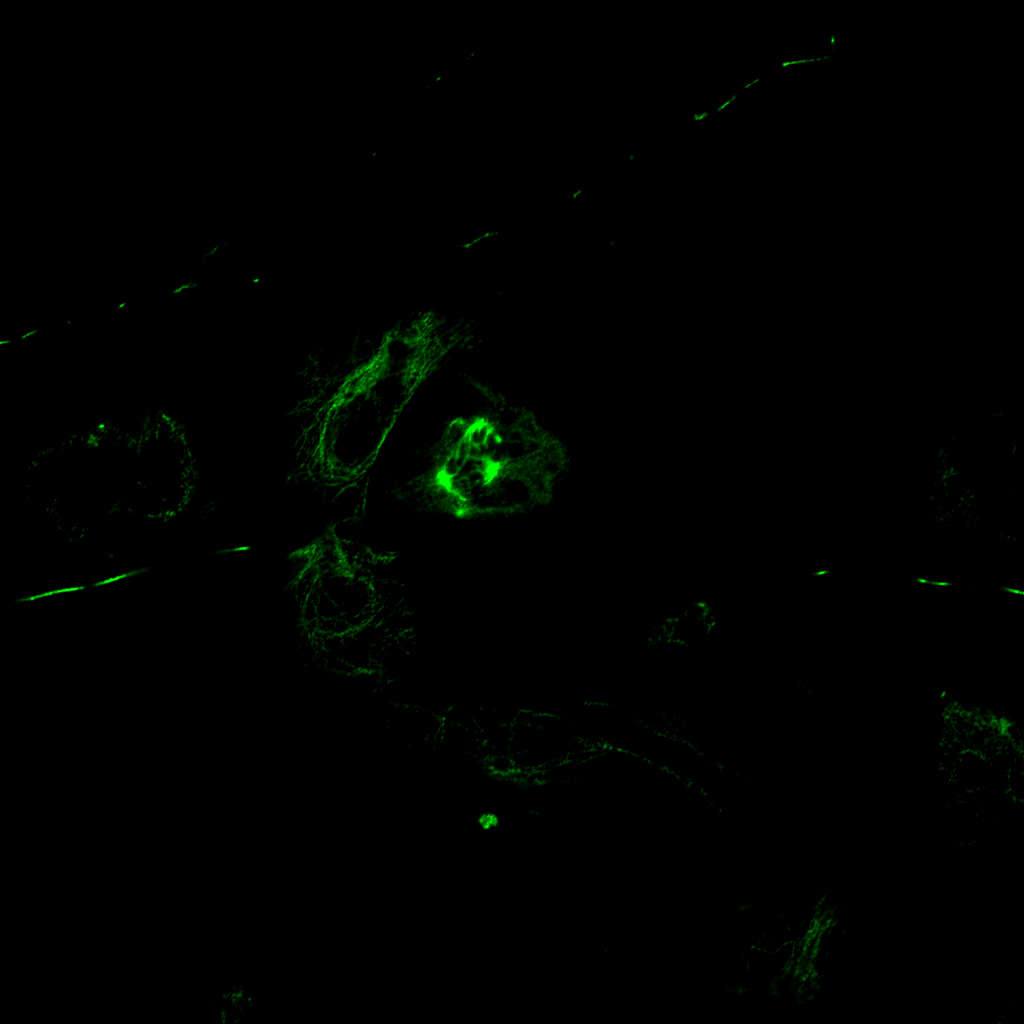

Supplement: Supplementary file 6 — Source data Fig. 5 [file 44321_2025_302_MOESM6_ESM.zip › Figure 5/5K/#12-3-atubulin.tif]

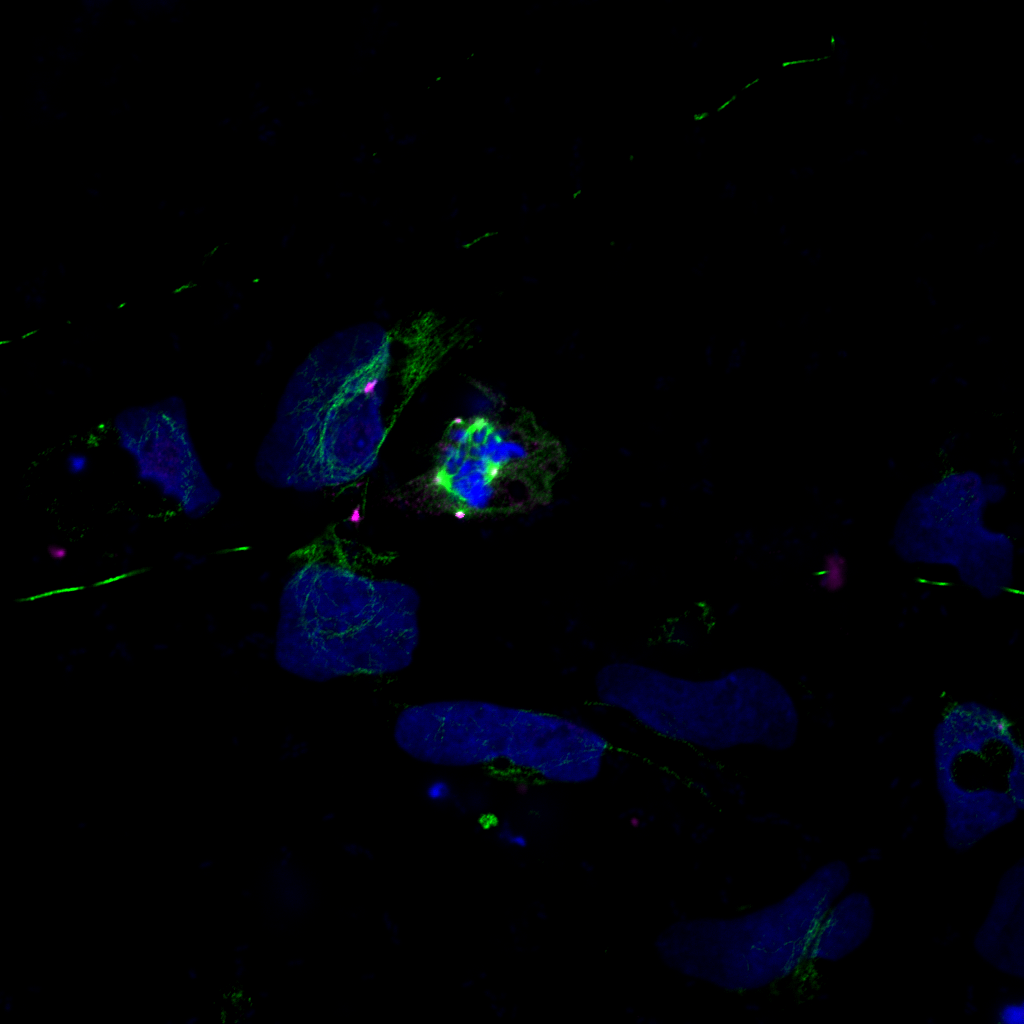

Supplement: Supplementary file 6 — Source data Fig. 5 [file 44321_2025_302_MOESM6_ESM.zip › Figure 5/5K/#12-3-merge.tif]

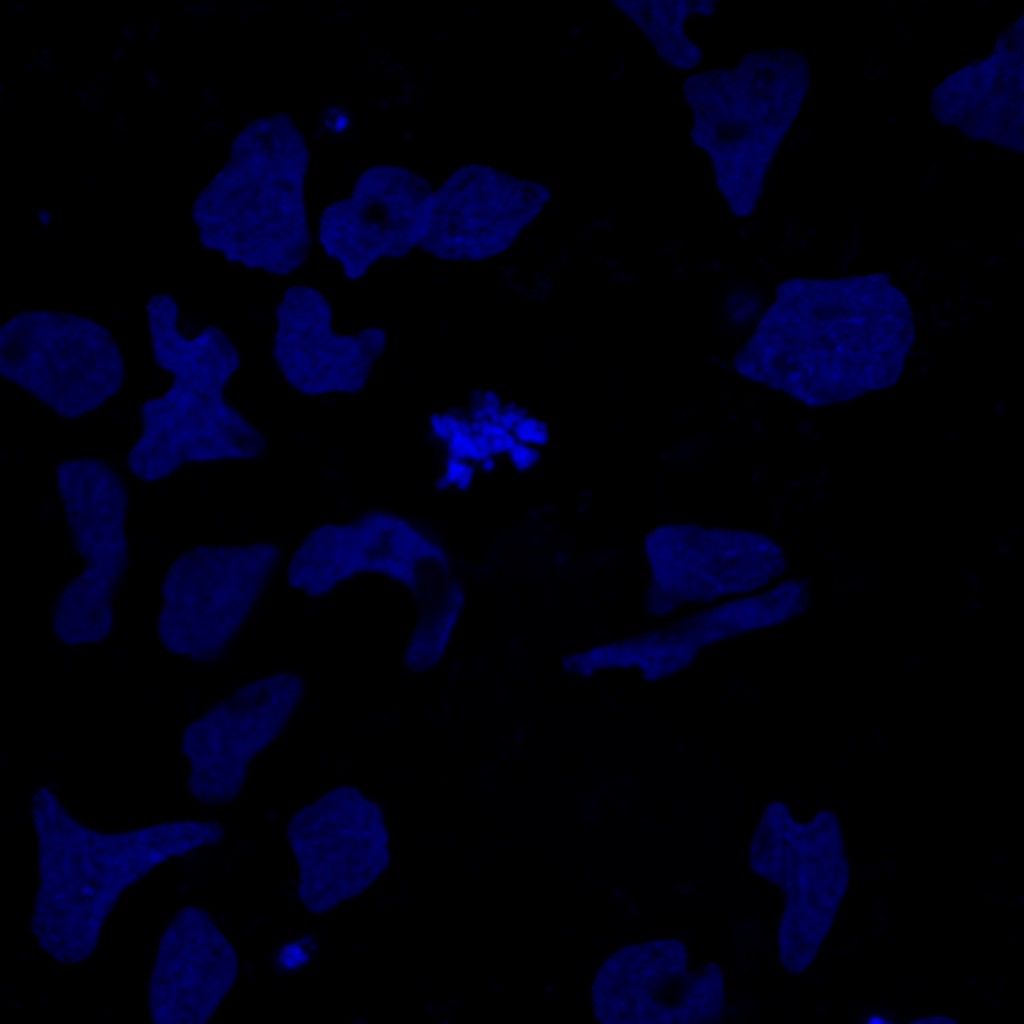

Supplement: Supplementary file 6 — Source data Fig. 5 [file 44321_2025_302_MOESM6_ESM.zip › Figure 5/5K/#7-5-DAPI.tif]

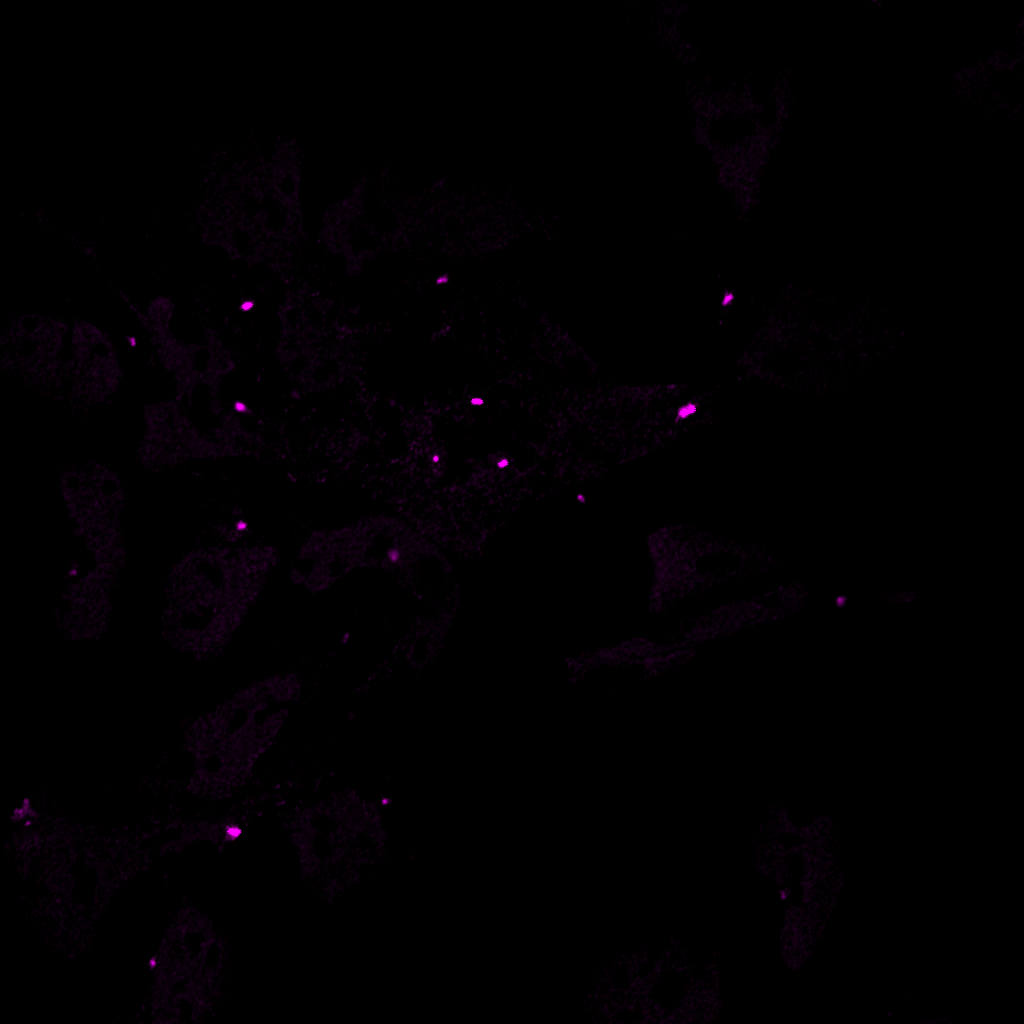

Supplement: Supplementary file 6 — Source data Fig. 5 [file 44321_2025_302_MOESM6_ESM.zip › Figure 5/5K/#7-5-PCNT.tif]

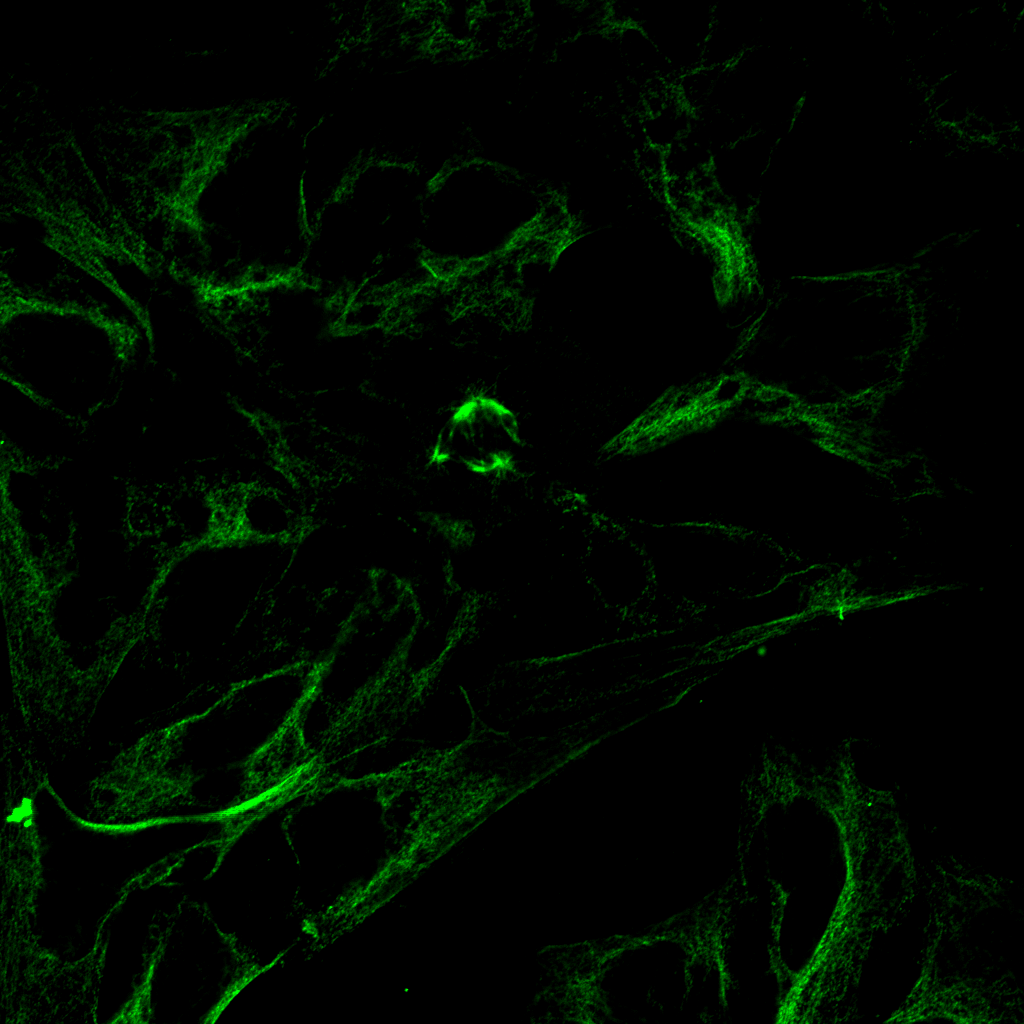

Supplement: Supplementary file 6 — Source data Fig. 5 [file 44321_2025_302_MOESM6_ESM.zip › Figure 5/5K/#7-5-atubulin.tif]

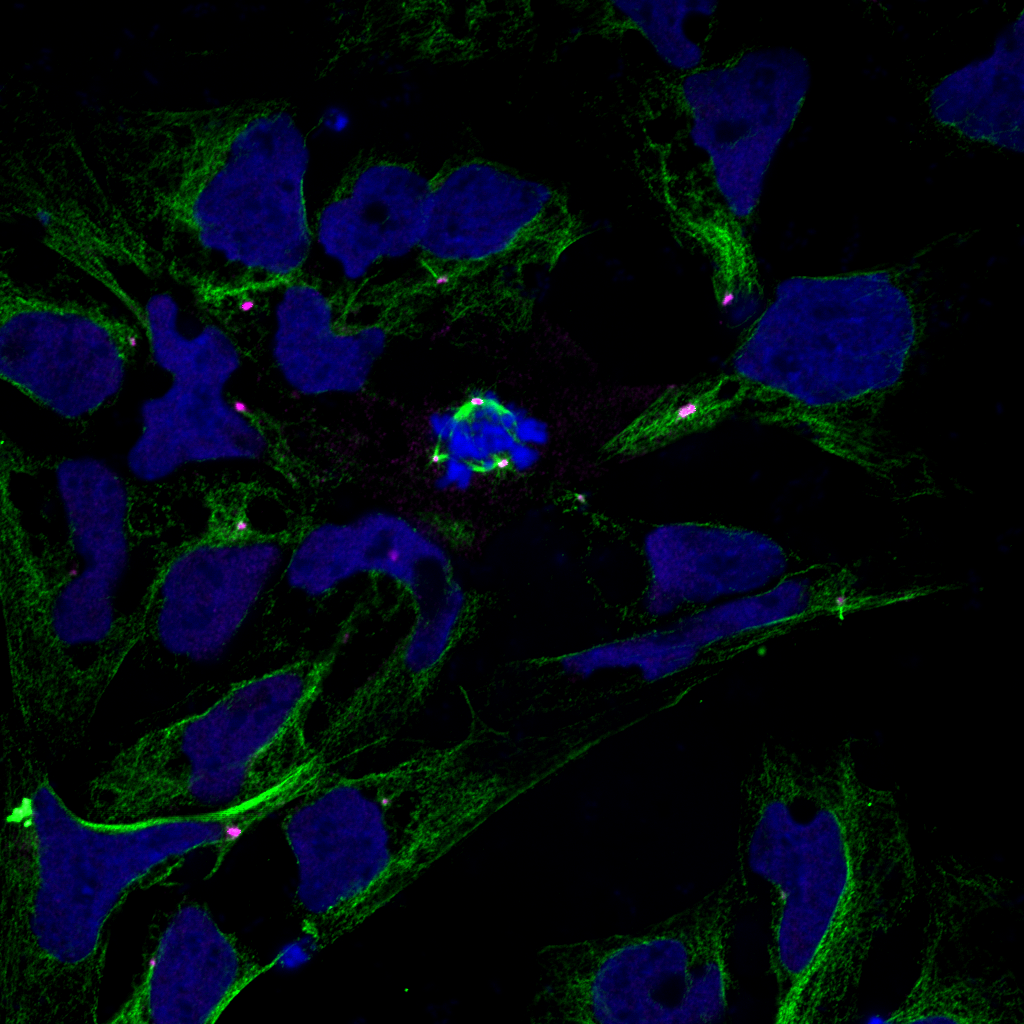

Supplement: Supplementary file 6 — Source data Fig. 5 [file 44321_2025_302_MOESM6_ESM.zip › Figure 5/5K/#7-5-merge.tif]

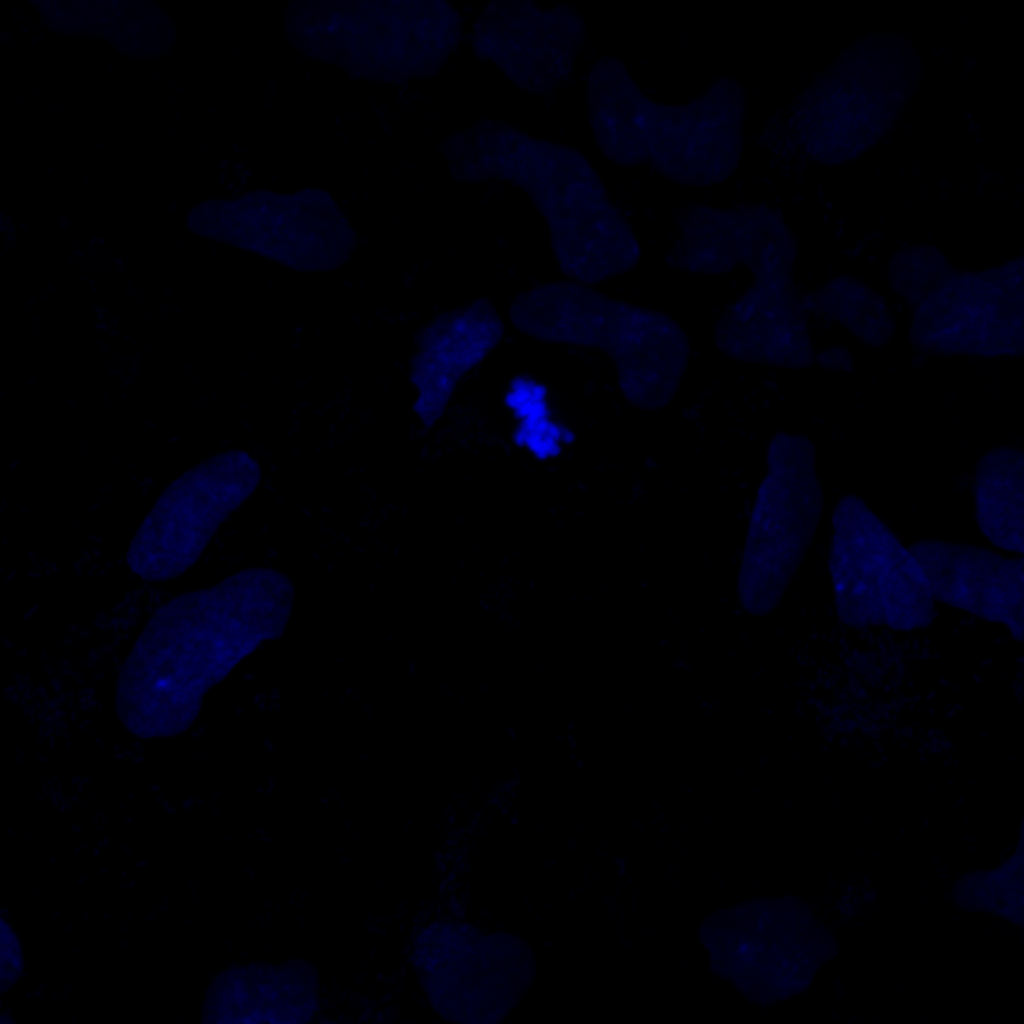

Supplement: Supplementary file 6 — Source data Fig. 5 [file 44321_2025_302_MOESM6_ESM.zip › Figure 5/5K/H9-DAPI.tif]

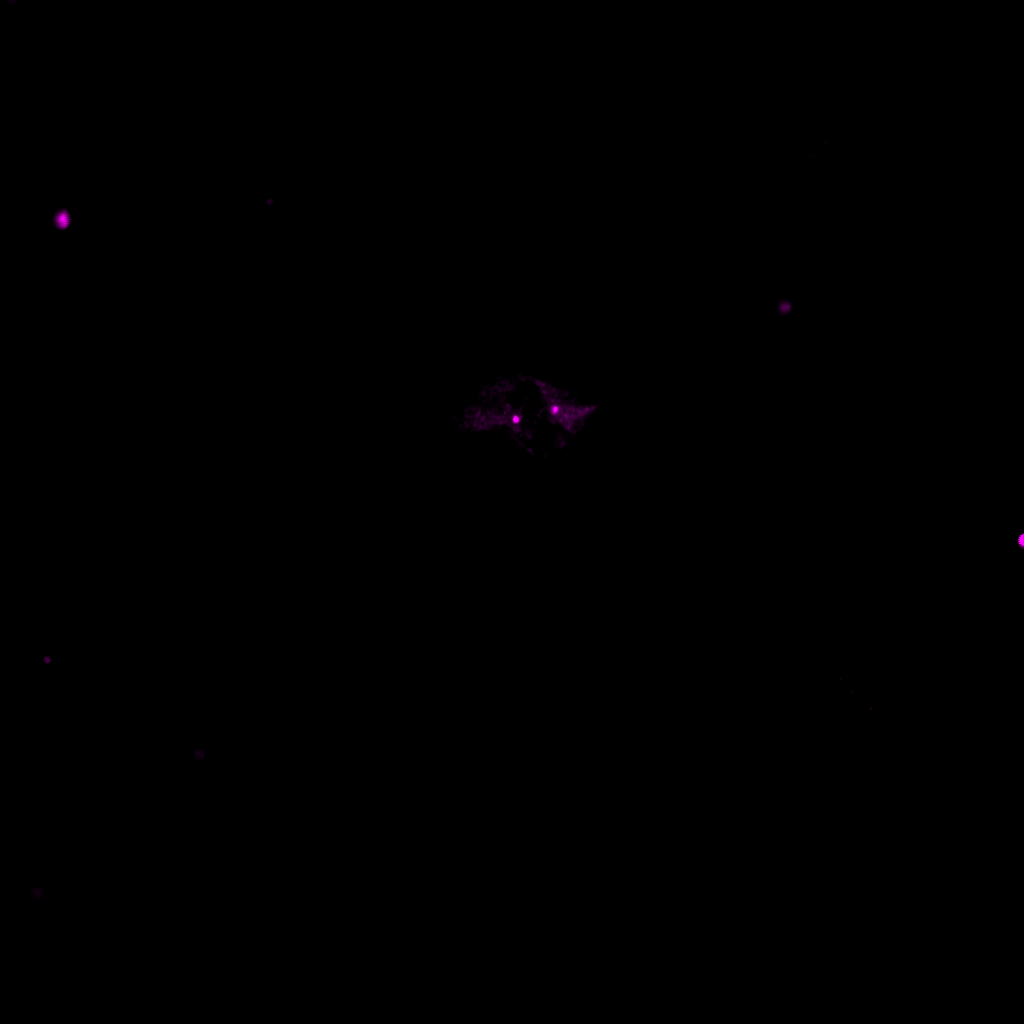

Supplement: Supplementary file 6 — Source data Fig. 5 [file 44321_2025_302_MOESM6_ESM.zip › Figure 5/5K/H9-PCNT.tif]

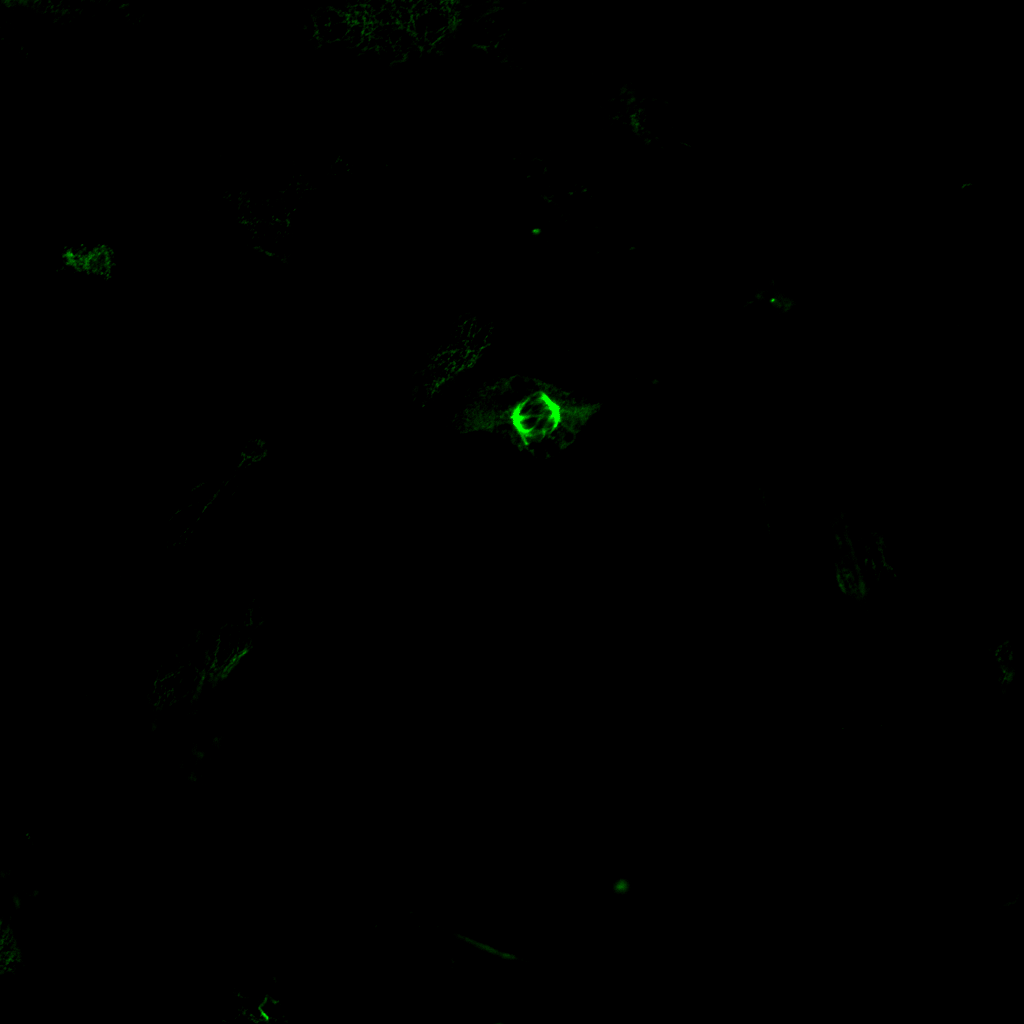

Supplement: Supplementary file 6 — Source data Fig. 5 [file 44321_2025_302_MOESM6_ESM.zip › Figure 5/5K/H9-atubulin.tif]

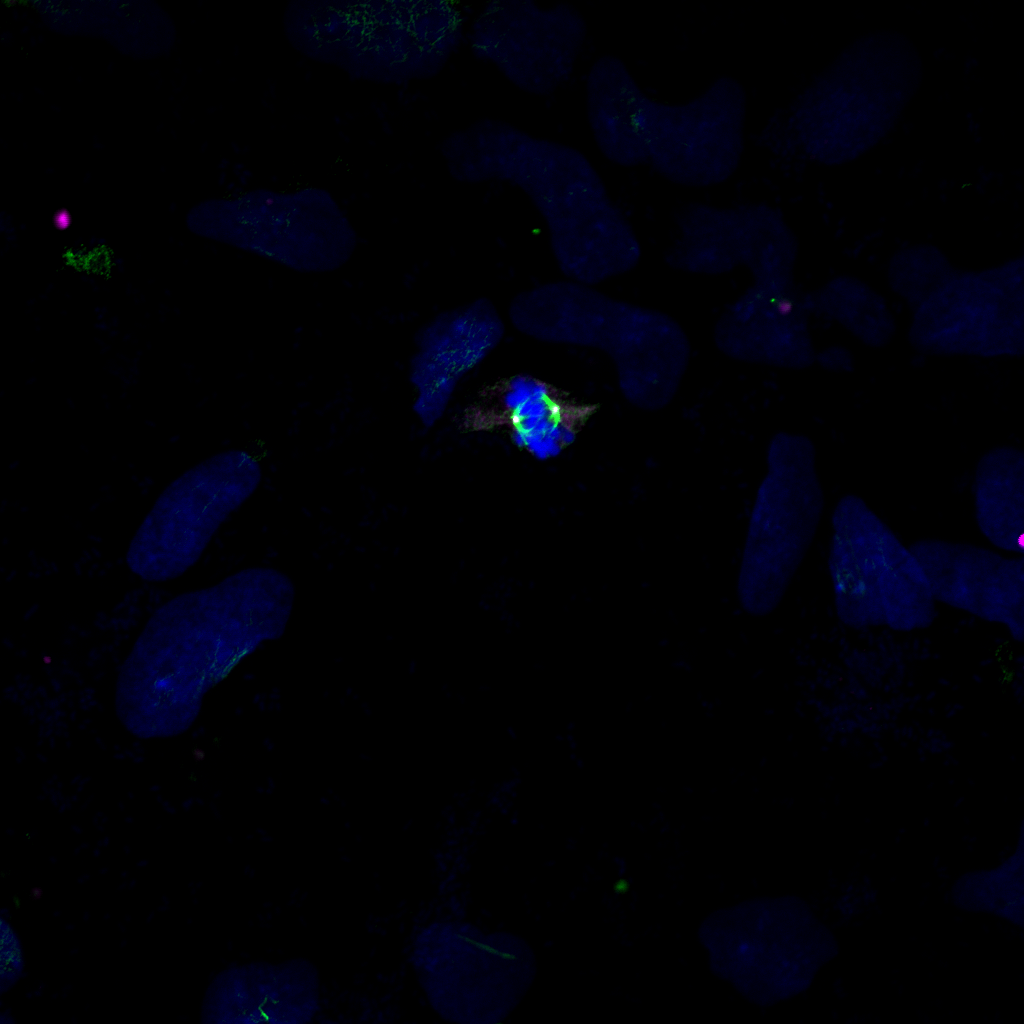

Supplement: Supplementary file 6 — Source data Fig. 5 [file 44321_2025_302_MOESM6_ESM.zip › Figure 5/5K/H9-merge.tif]

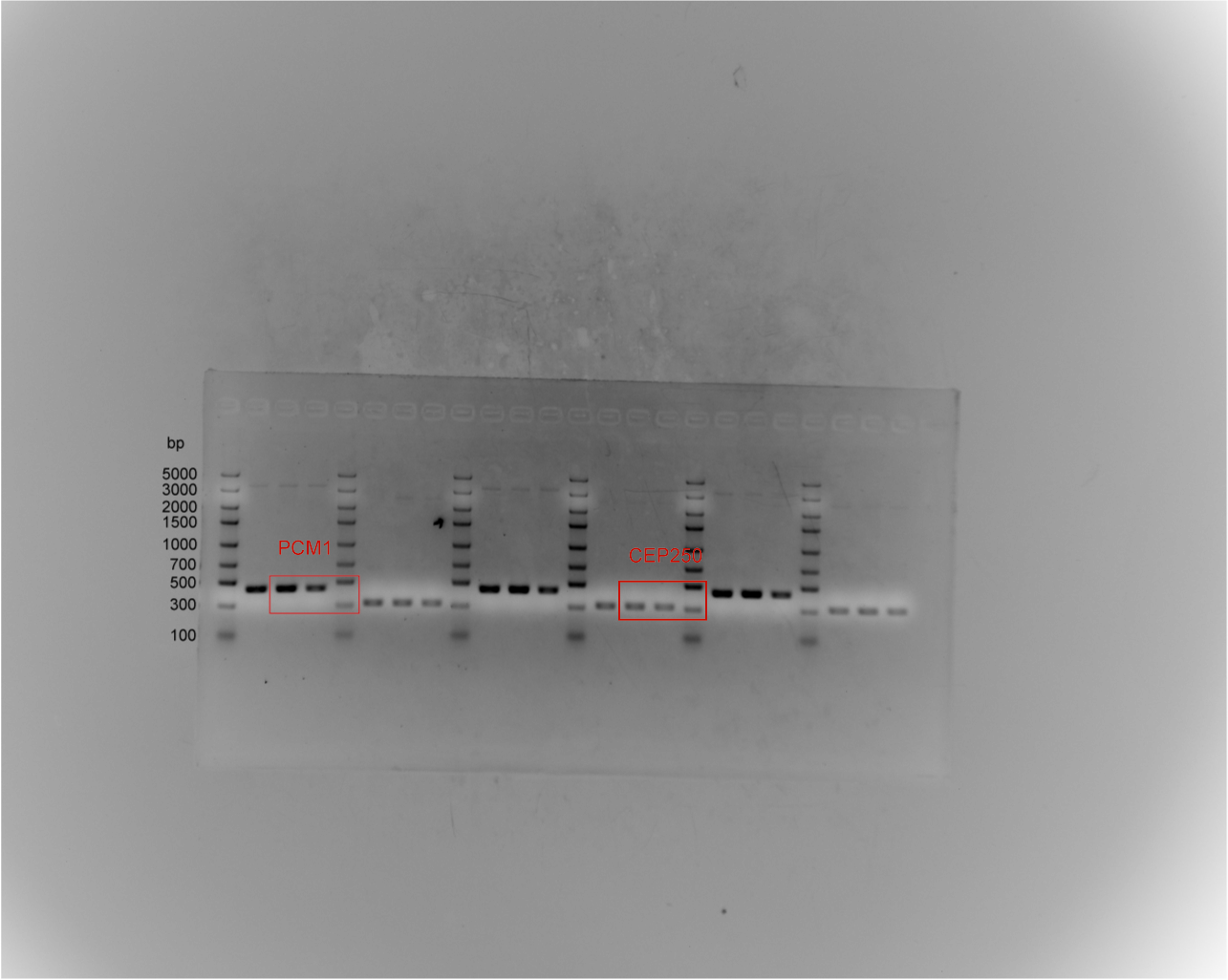

Supplement: Supplementary file 7 — Source data Fig. 6 [file 44321_2025_302_MOESM7_ESM.zip › Figure 6/6I-J/RTPCR.tif]

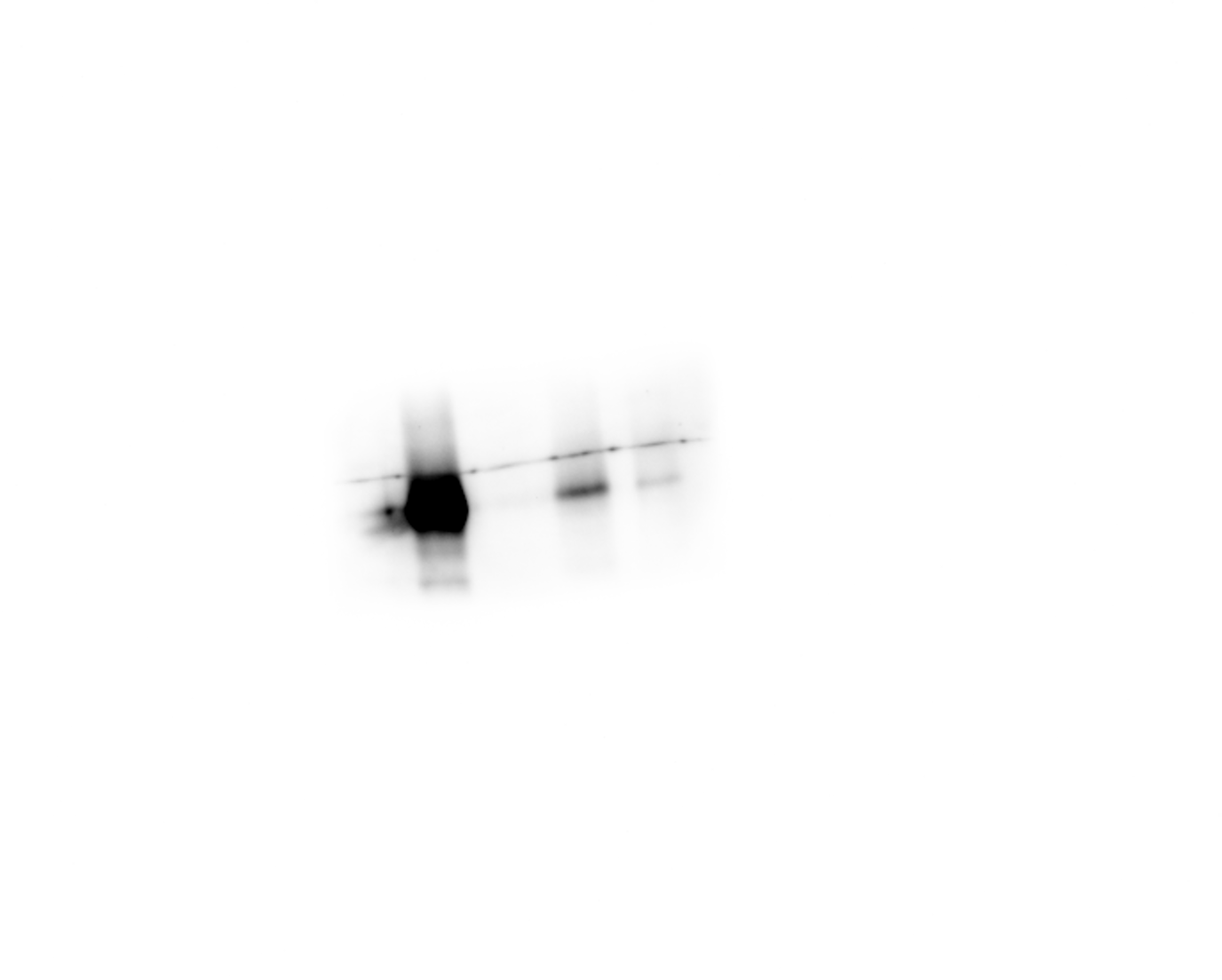

Supplement: Supplementary file 7 — Source data Fig. 6 [file 44321_2025_302_MOESM7_ESM.zip › Figure 6/6K/blot-flag.Tif]

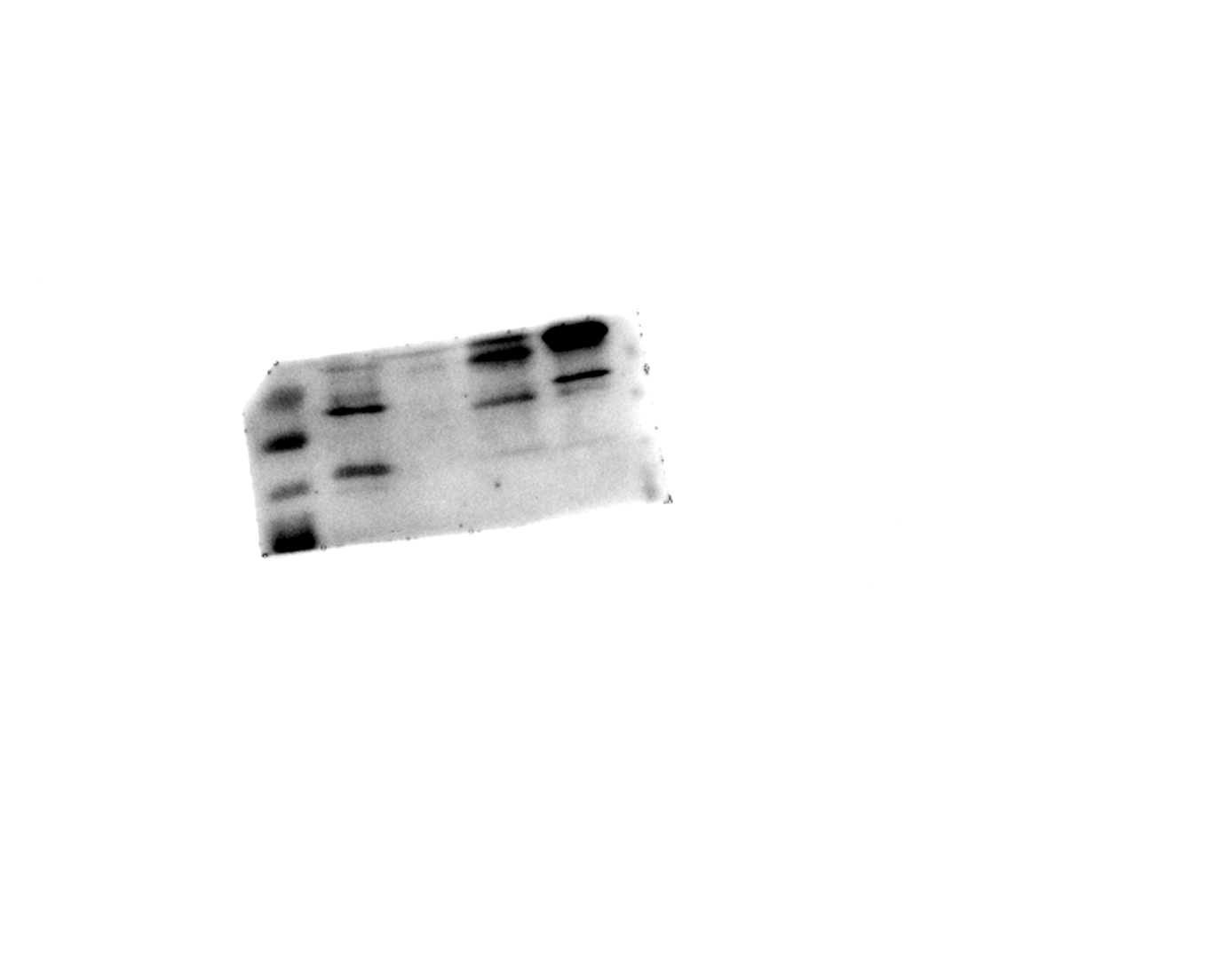

Supplement: Supplementary file 7 — Source data Fig. 6 [file 44321_2025_302_MOESM7_ESM.zip › Figure 6/6K/blot-myc.Tif]

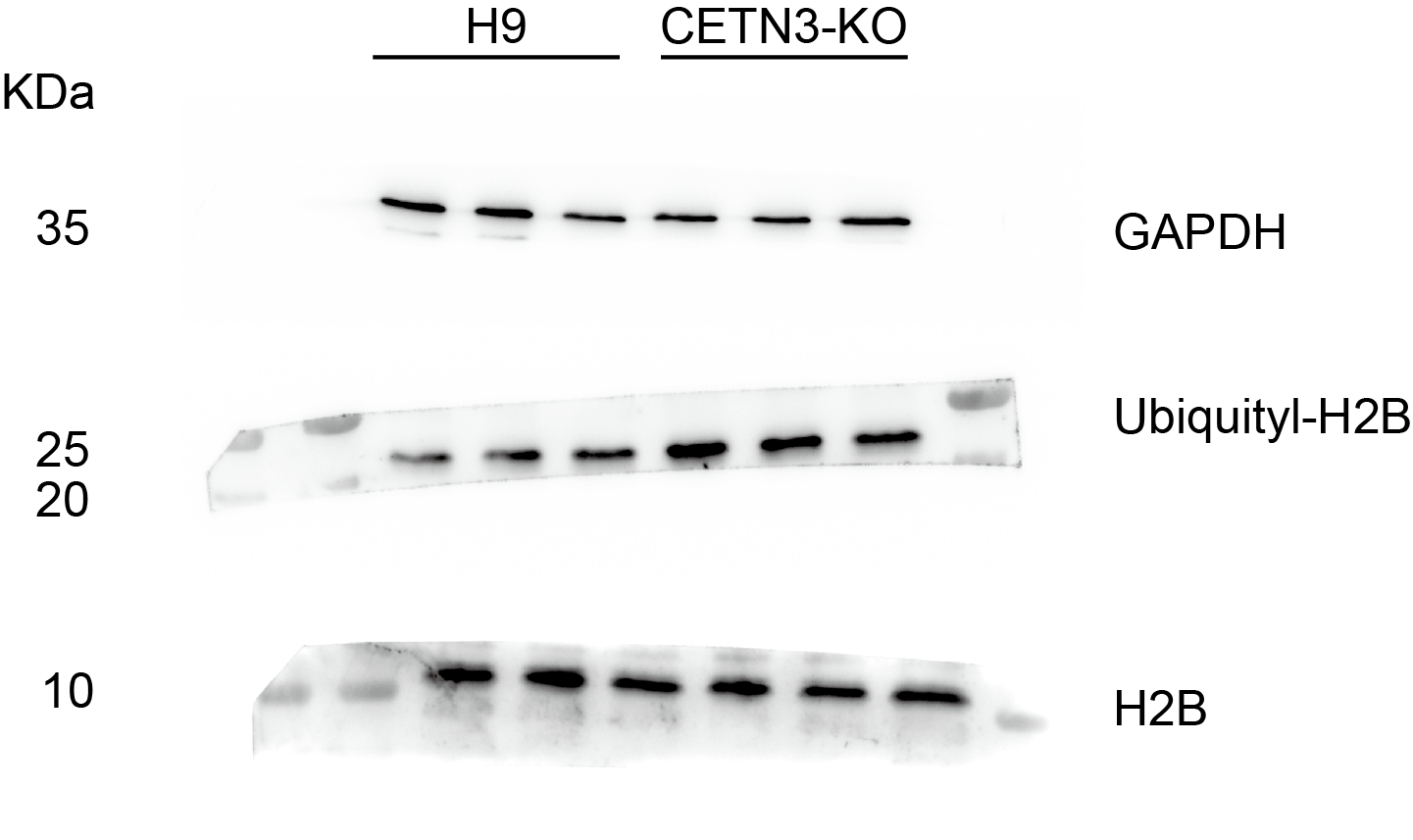

Supplement: Supplementary file 7 — Source data Fig. 6 [file 44321_2025_302_MOESM7_ESM.zip › Figure 6/6L/WB.png]

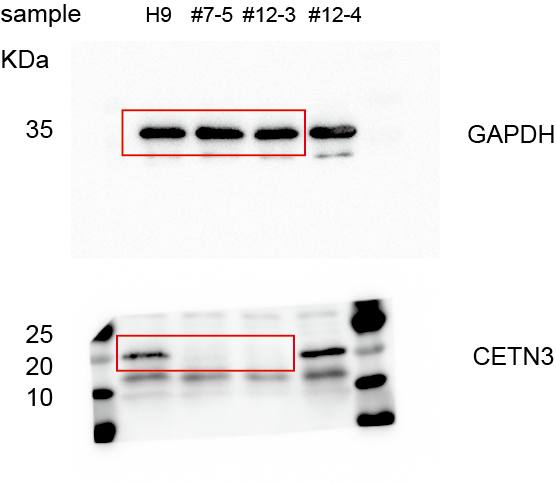

Supplement: Supplementary file 8 — Source data Fig. 2 [file 44321_2025_302_MOESM8_ESM.zip › Figure 2/2B/western blot.png]

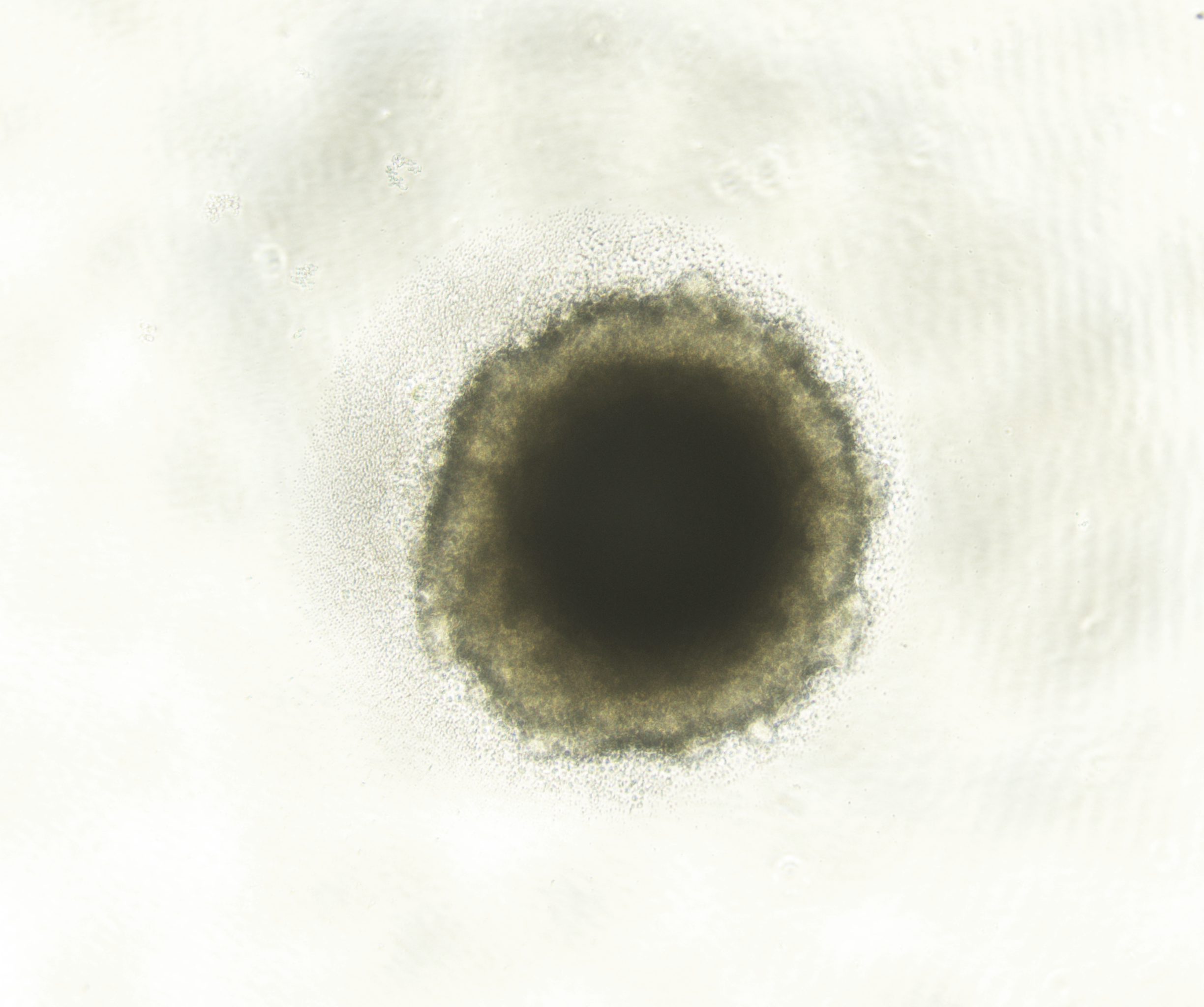

Supplement: Supplementary file 8 — Source data Fig. 2 [file 44321_2025_302_MOESM8_ESM.zip › Figure 2/2E/#12-3-Day10.tif]

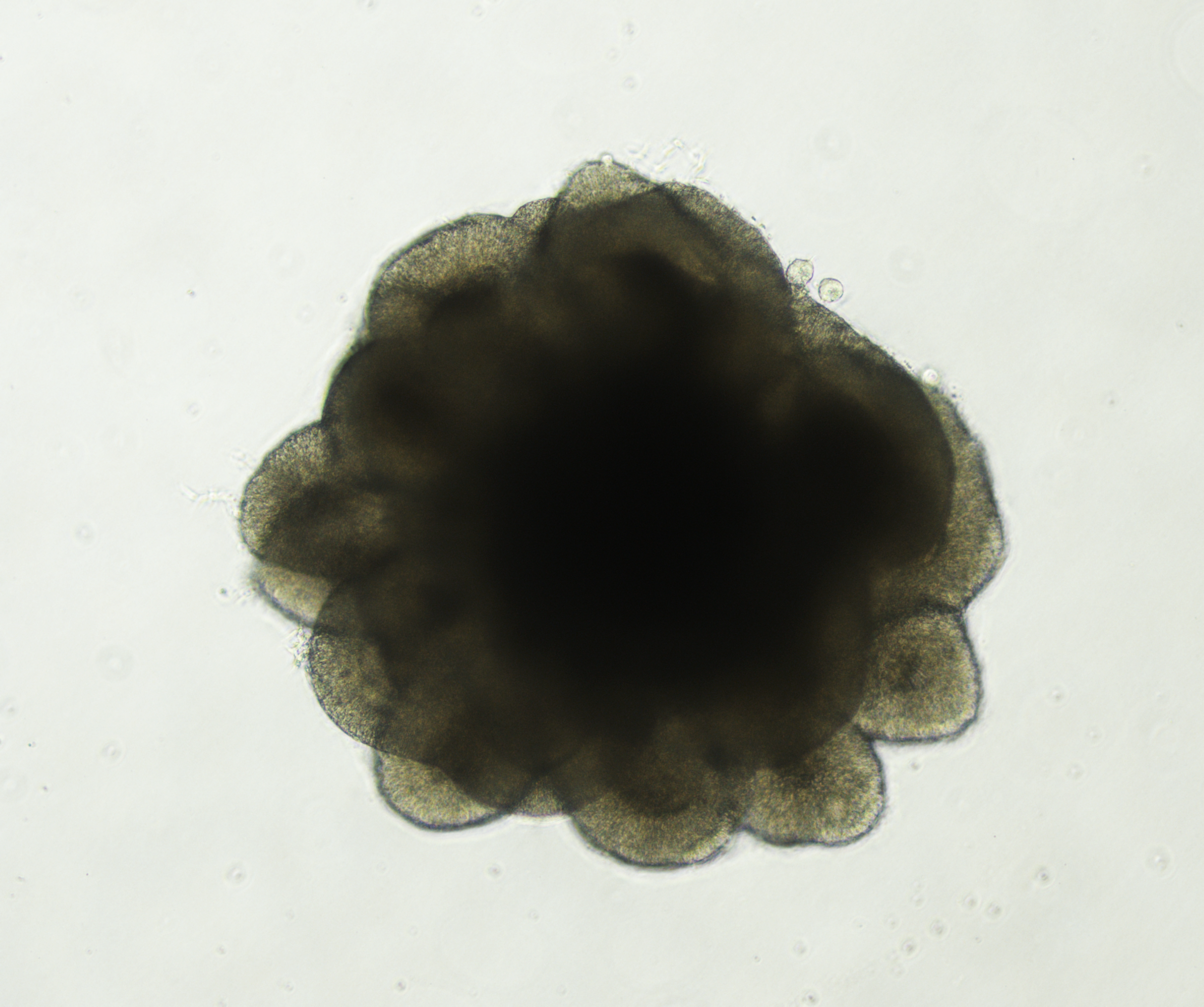

Supplement: Supplementary file 8 — Source data Fig. 2 [file 44321_2025_302_MOESM8_ESM.zip › Figure 2/2E/#12-3-Day15.tif]

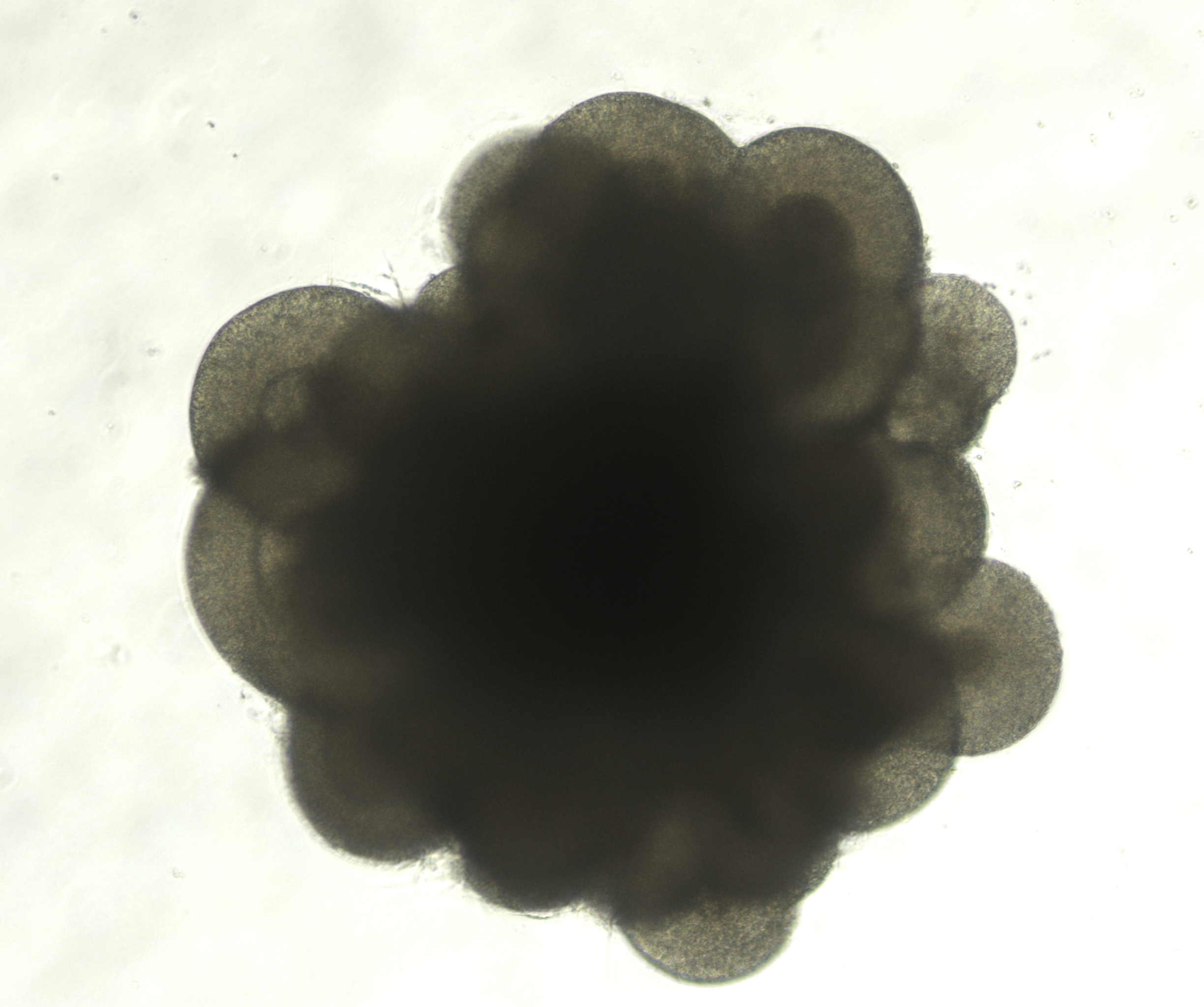

Supplement: Supplementary file 8 — Source data Fig. 2 [file 44321_2025_302_MOESM8_ESM.zip › Figure 2/2E/#12-3-Day20.tif]

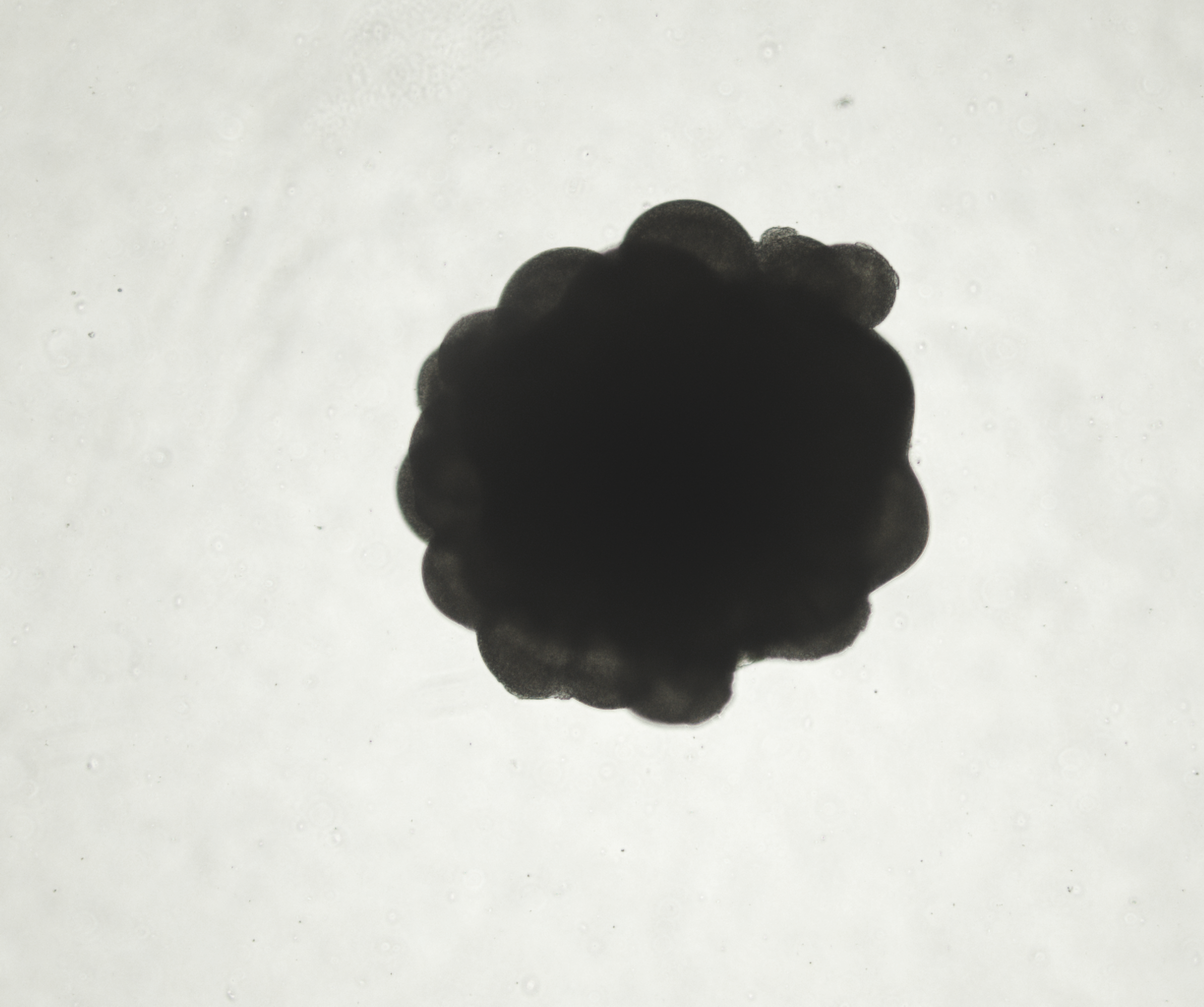

Supplement: Supplementary file 8 — Source data Fig. 2 [file 44321_2025_302_MOESM8_ESM.zip › Figure 2/2E/#12-3-Day25.tif]

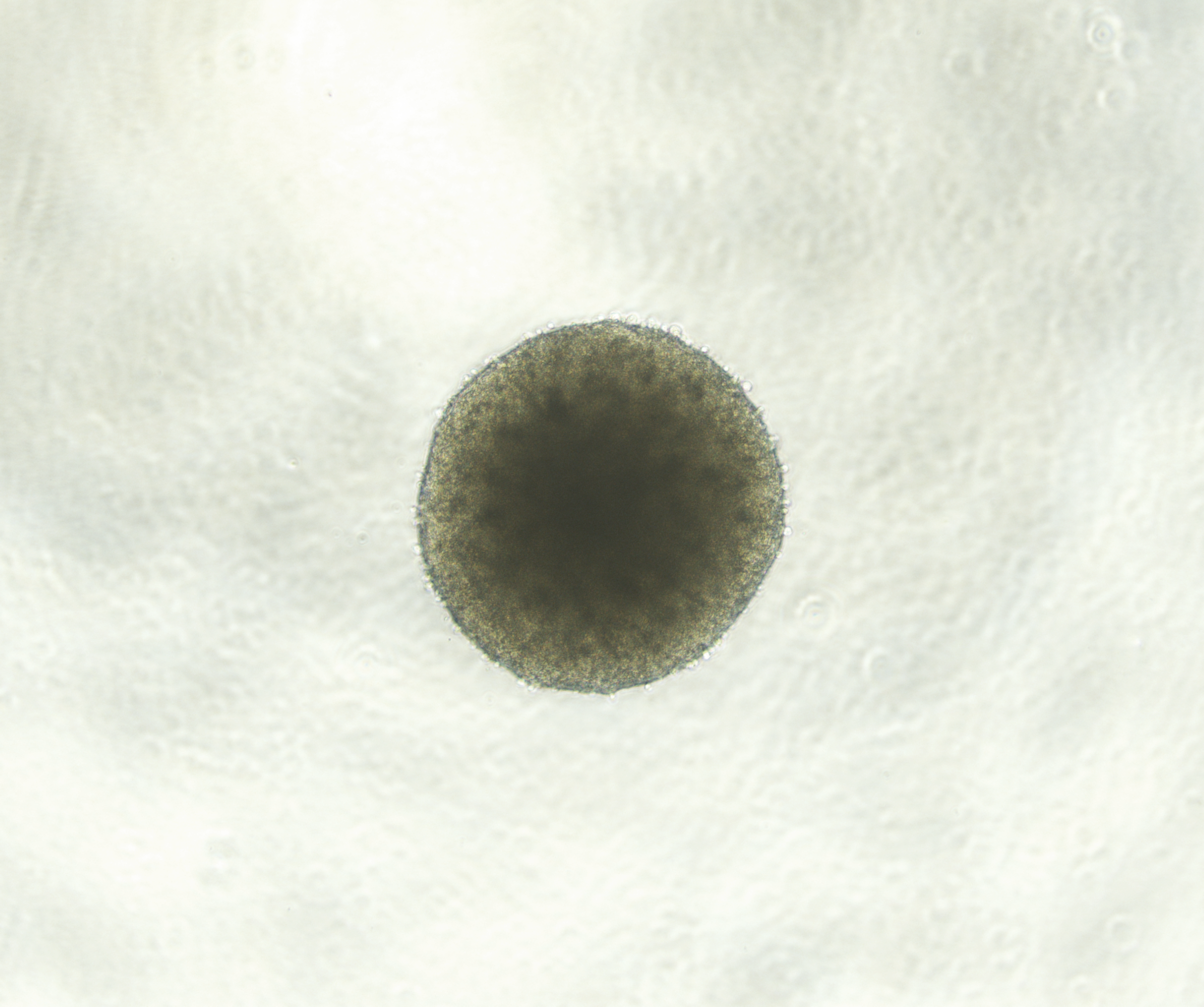

Supplement: Supplementary file 8 — Source data Fig. 2 [file 44321_2025_302_MOESM8_ESM.zip › Figure 2/2E/#12-3-Day3.tif]

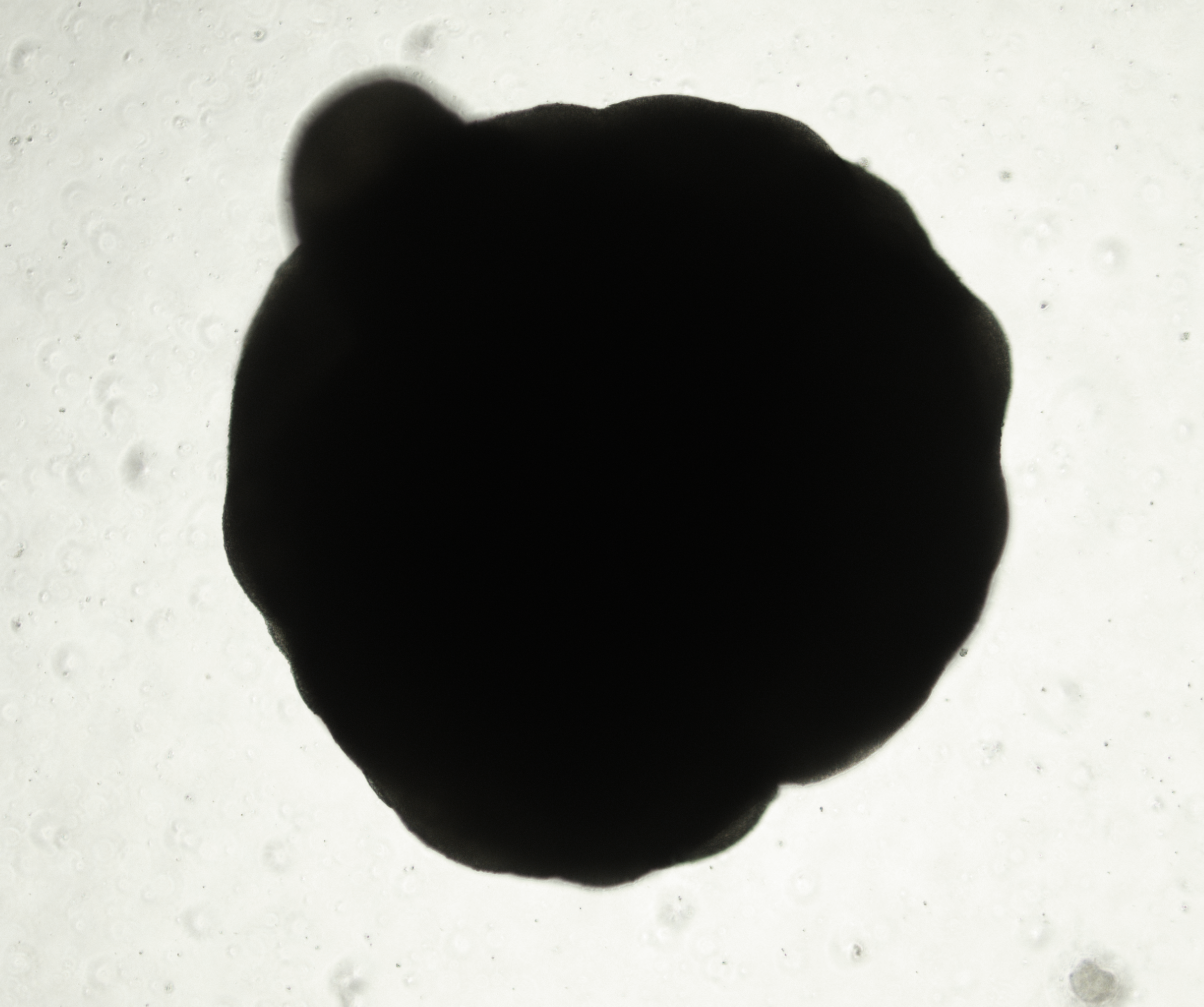

Supplement: Supplementary file 8 — Source data Fig. 2 [file 44321_2025_302_MOESM8_ESM.zip › Figure 2/2E/#12-3-Day35.tif]

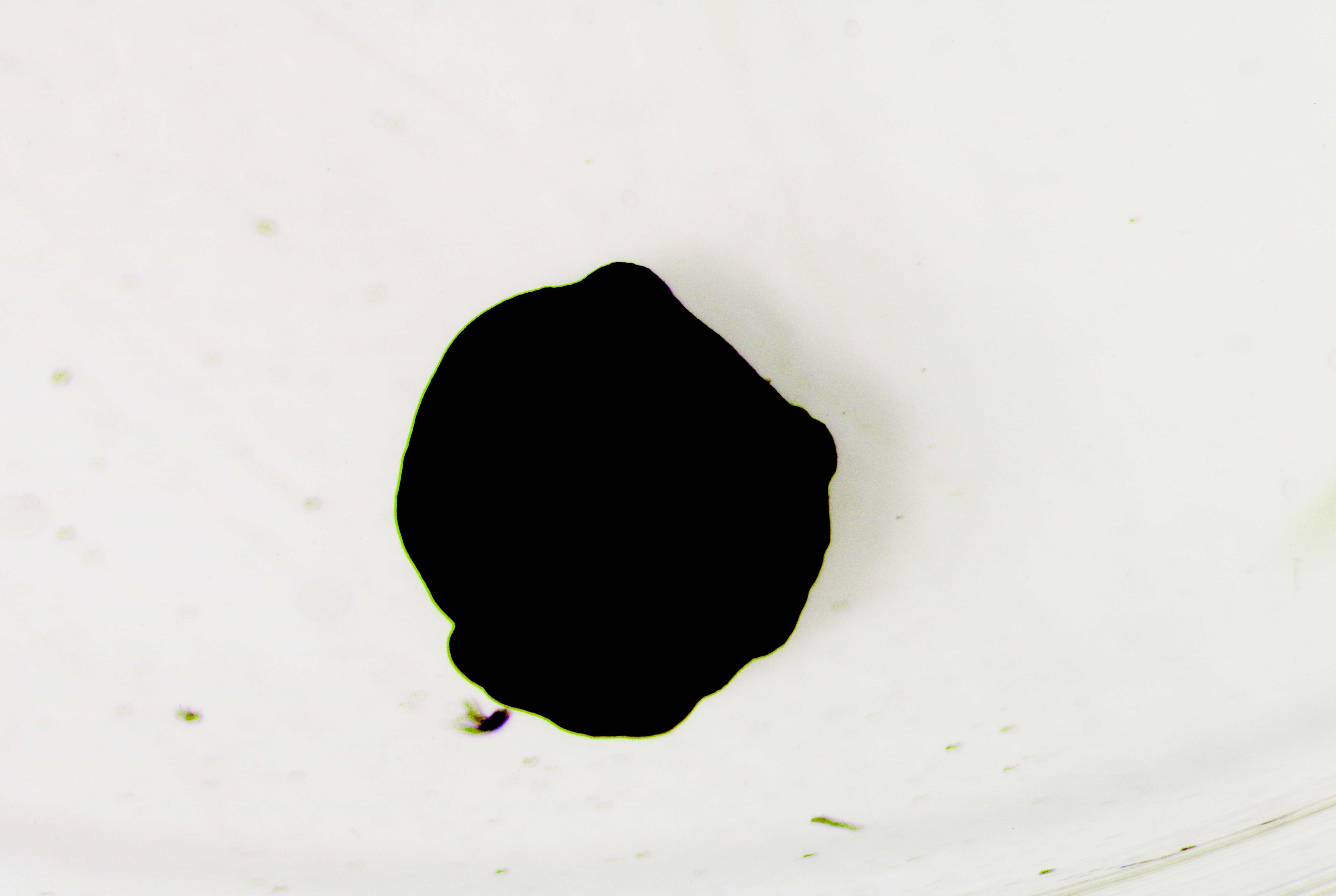

Supplement: Supplementary file 8 — Source data Fig. 2 [file 44321_2025_302_MOESM8_ESM.zip › Figure 2/2E/#12-3-Day45.png]

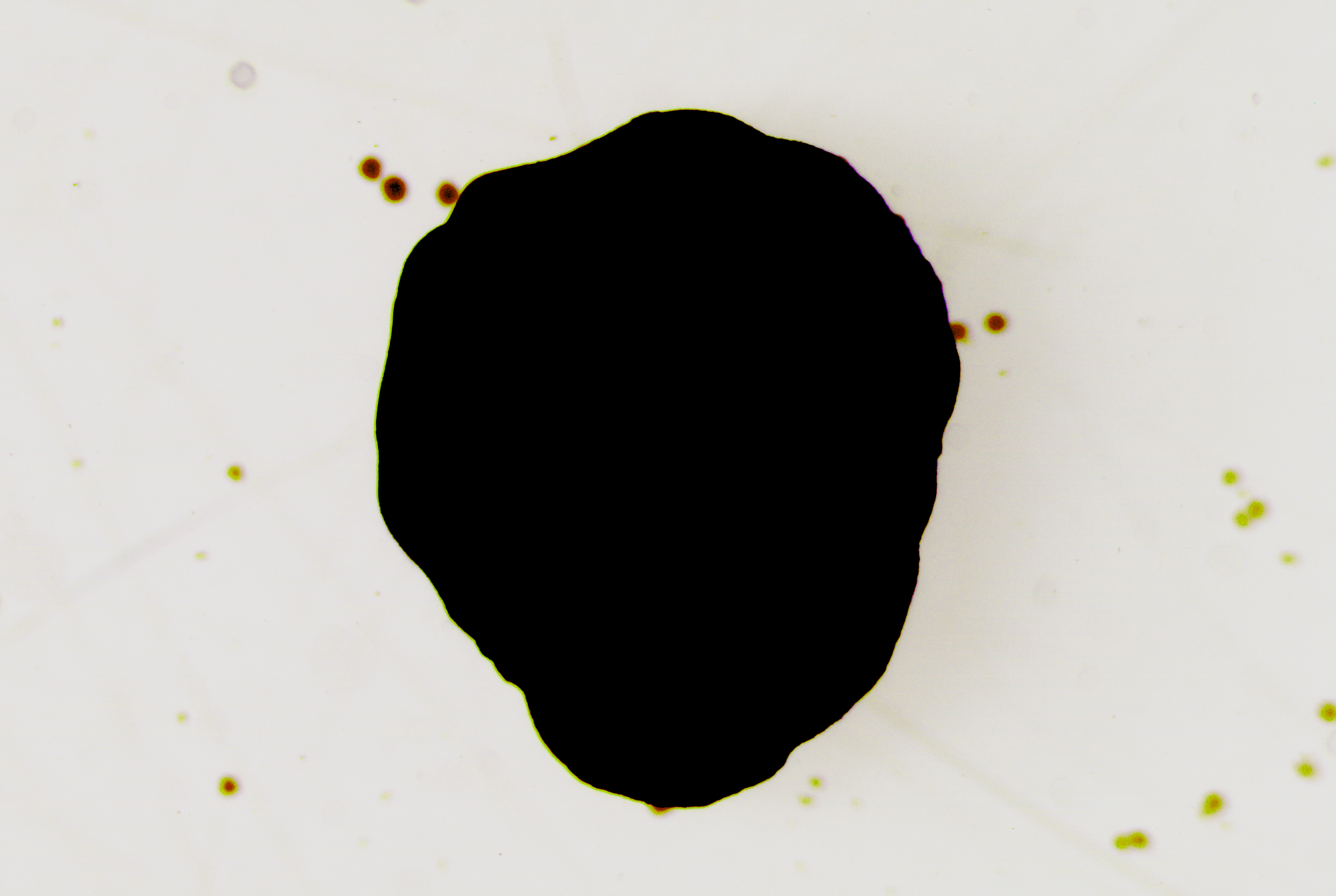

Supplement: Supplementary file 8 — Source data Fig. 2 [file 44321_2025_302_MOESM8_ESM.zip › Figure 2/2E/#12-3-Day60.png]

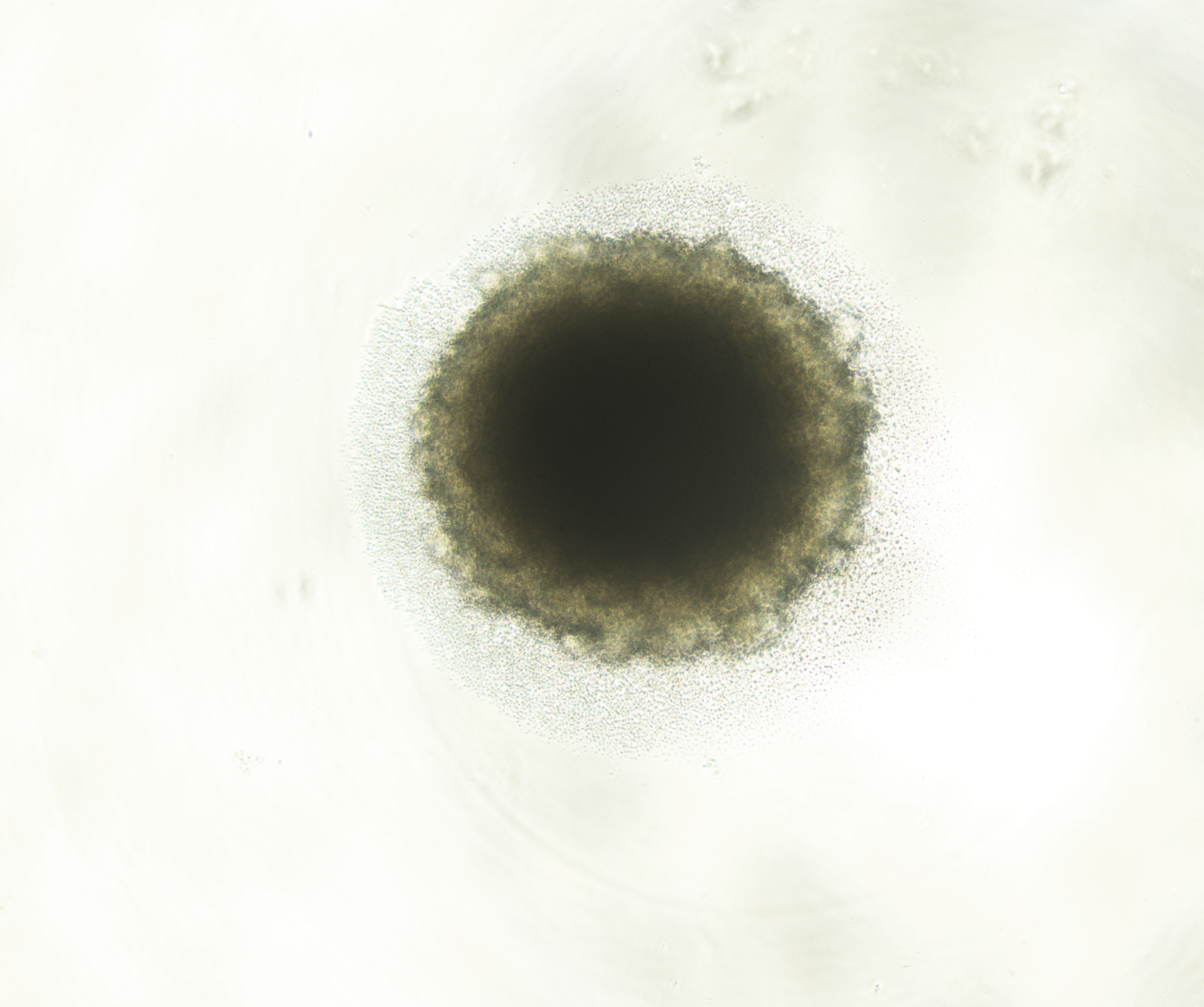

Supplement: Supplementary file 8 — Source data Fig. 2 [file 44321_2025_302_MOESM8_ESM.zip › Figure 2/2E/#7-5-Day10.tif]

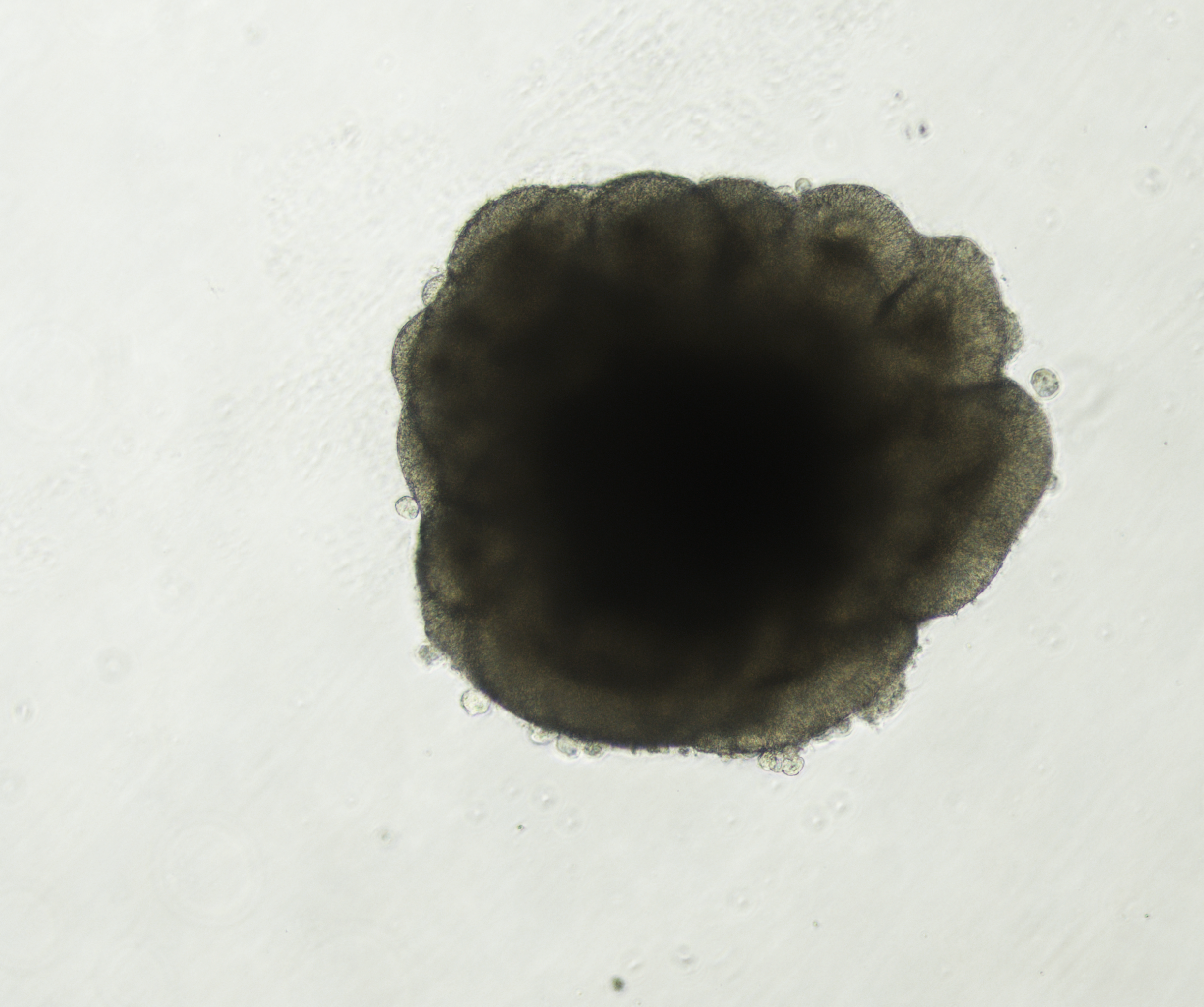

Supplement: Supplementary file 8 — Source data Fig. 2 [file 44321_2025_302_MOESM8_ESM.zip › Figure 2/2E/#7-5-Day15.tif]

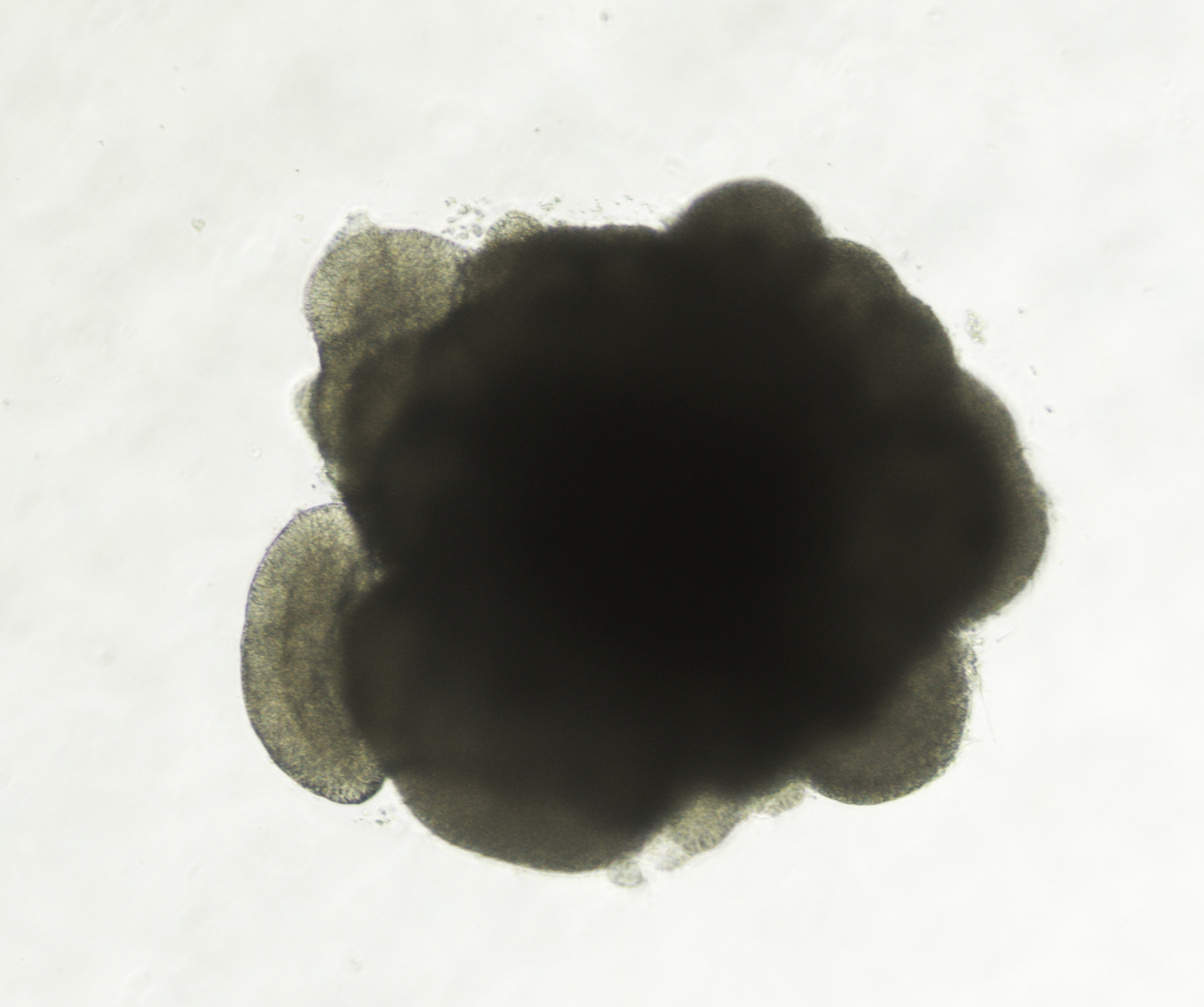

Supplement: Supplementary file 8 — Source data Fig. 2 [file 44321_2025_302_MOESM8_ESM.zip › Figure 2/2E/#7-5-Day20.tif]

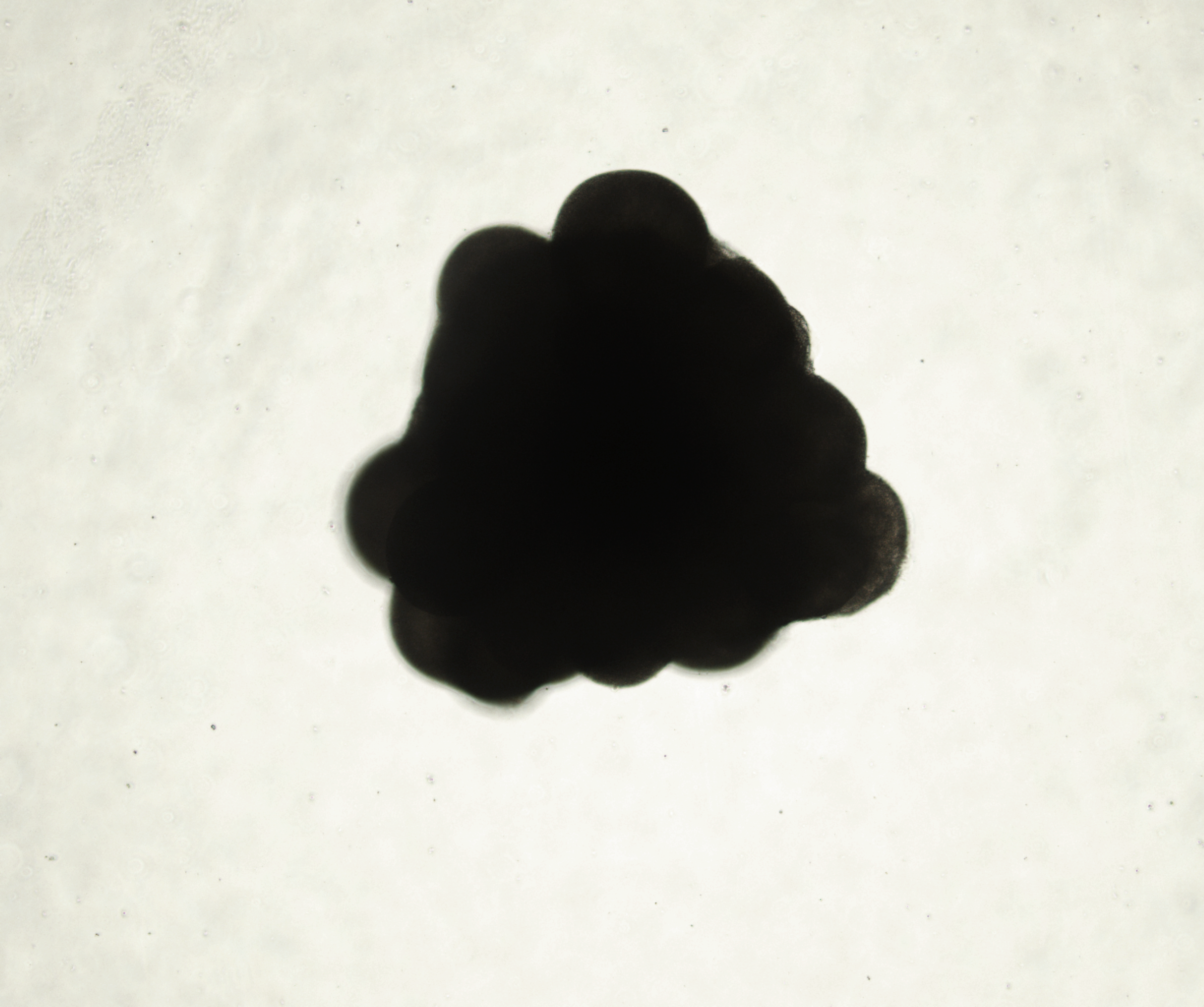

Supplement: Supplementary file 8 — Source data Fig. 2 [file 44321_2025_302_MOESM8_ESM.zip › Figure 2/2E/#7-5-Day25.tif]

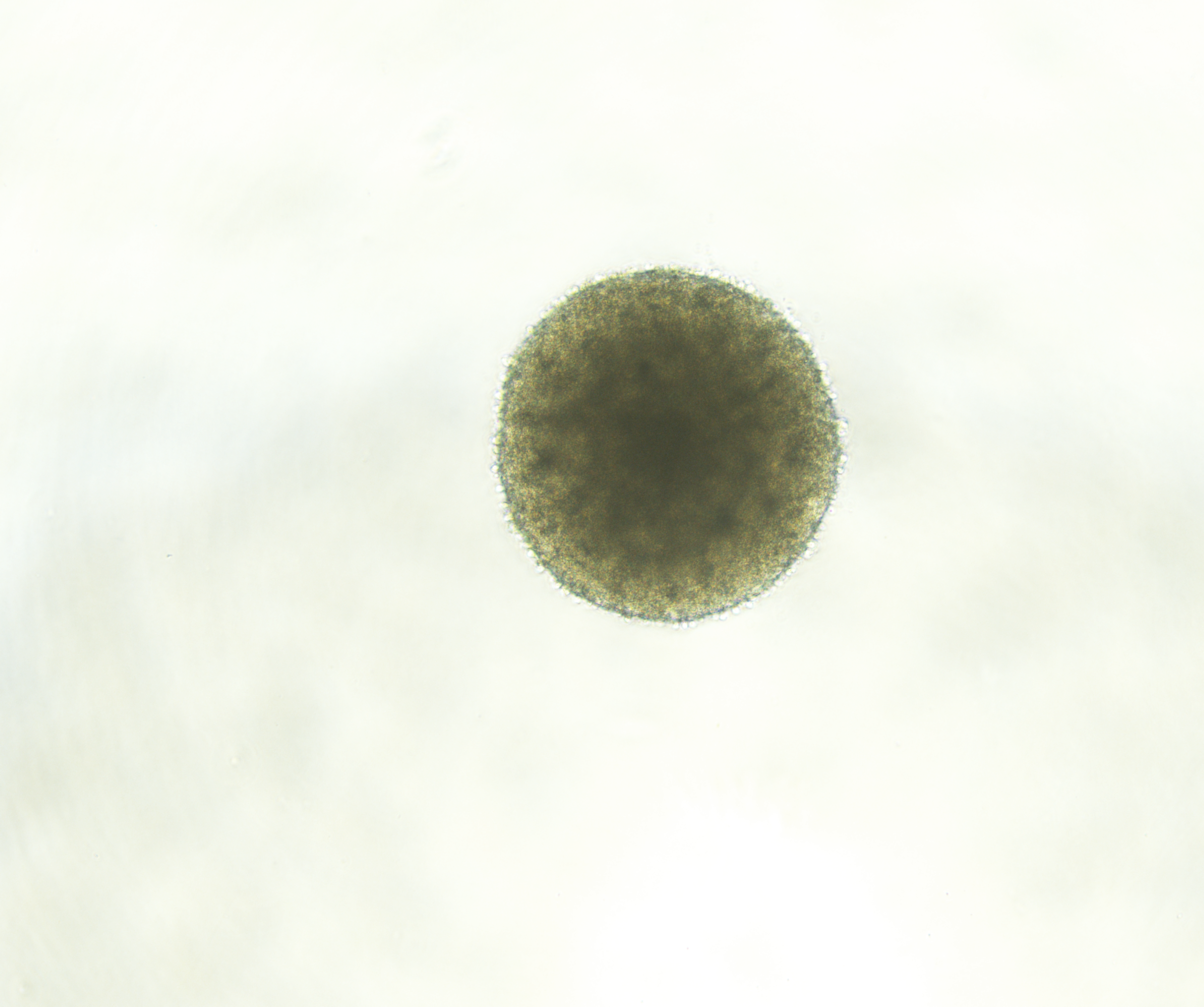

Supplement: Supplementary file 8 — Source data Fig. 2 [file 44321_2025_302_MOESM8_ESM.zip › Figure 2/2E/#7-5-Day3.tif]

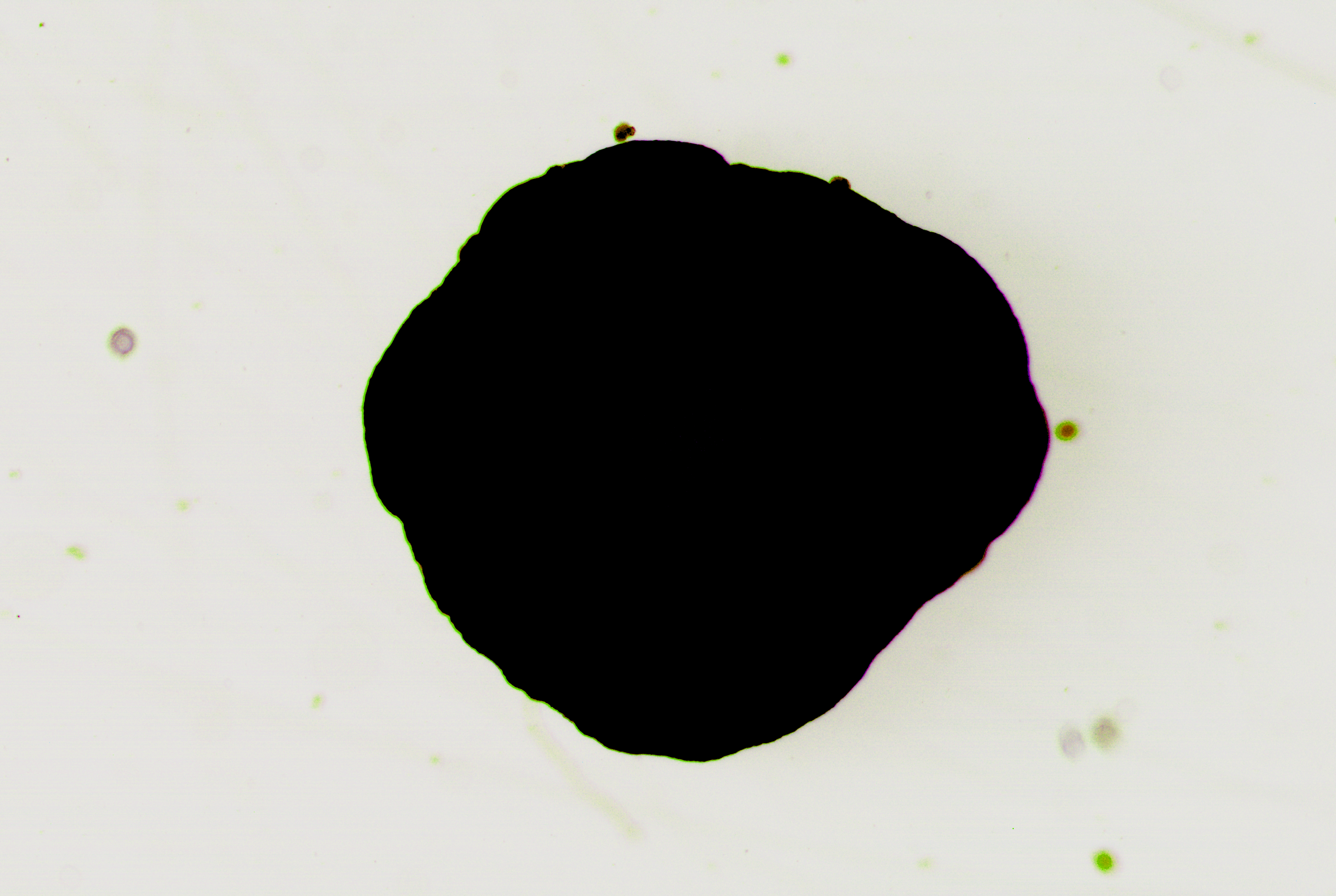

Supplement: Supplementary file 8 — Source data Fig. 2 [file 44321_2025_302_MOESM8_ESM.zip › Figure 2/2E/#7-5-Day60.png]

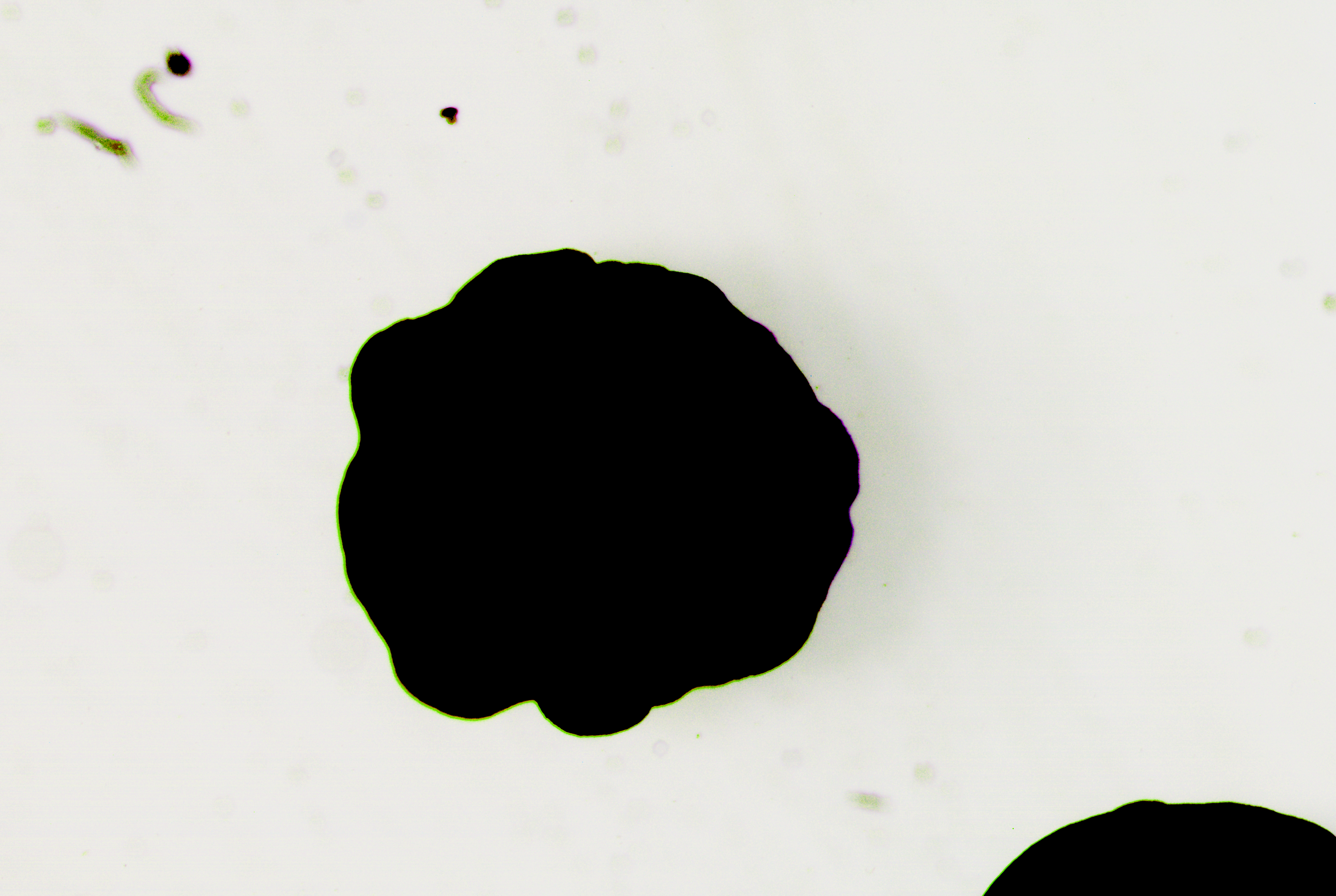

Supplement: Supplementary file 8 — Source data Fig. 2 [file 44321_2025_302_MOESM8_ESM.zip › Figure 2/2E/#7-5-day45.tif]

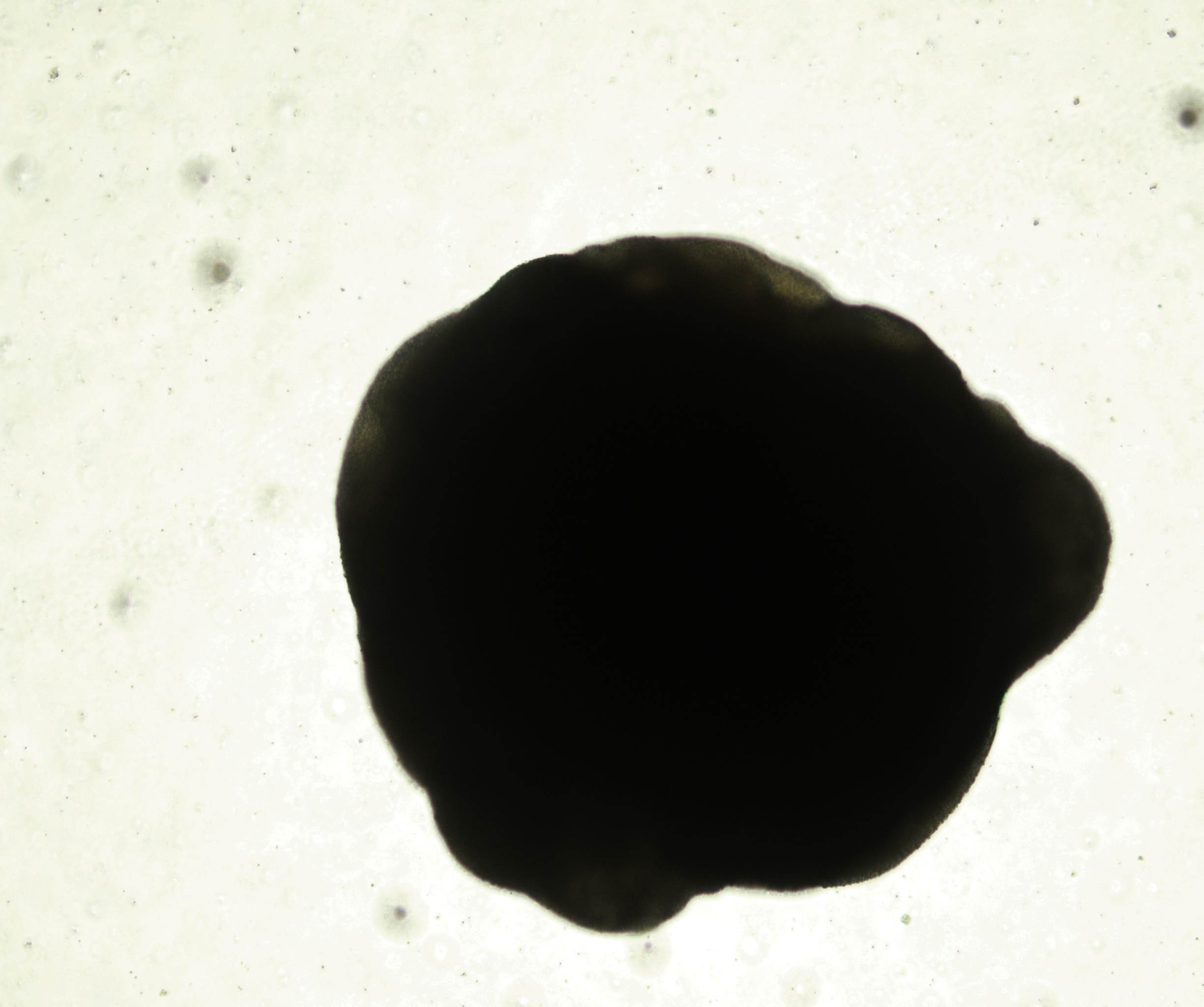

Supplement: Supplementary file 8 — Source data Fig. 2 [file 44321_2025_302_MOESM8_ESM.zip › Figure 2/2E/#7-5_Day35.tif]

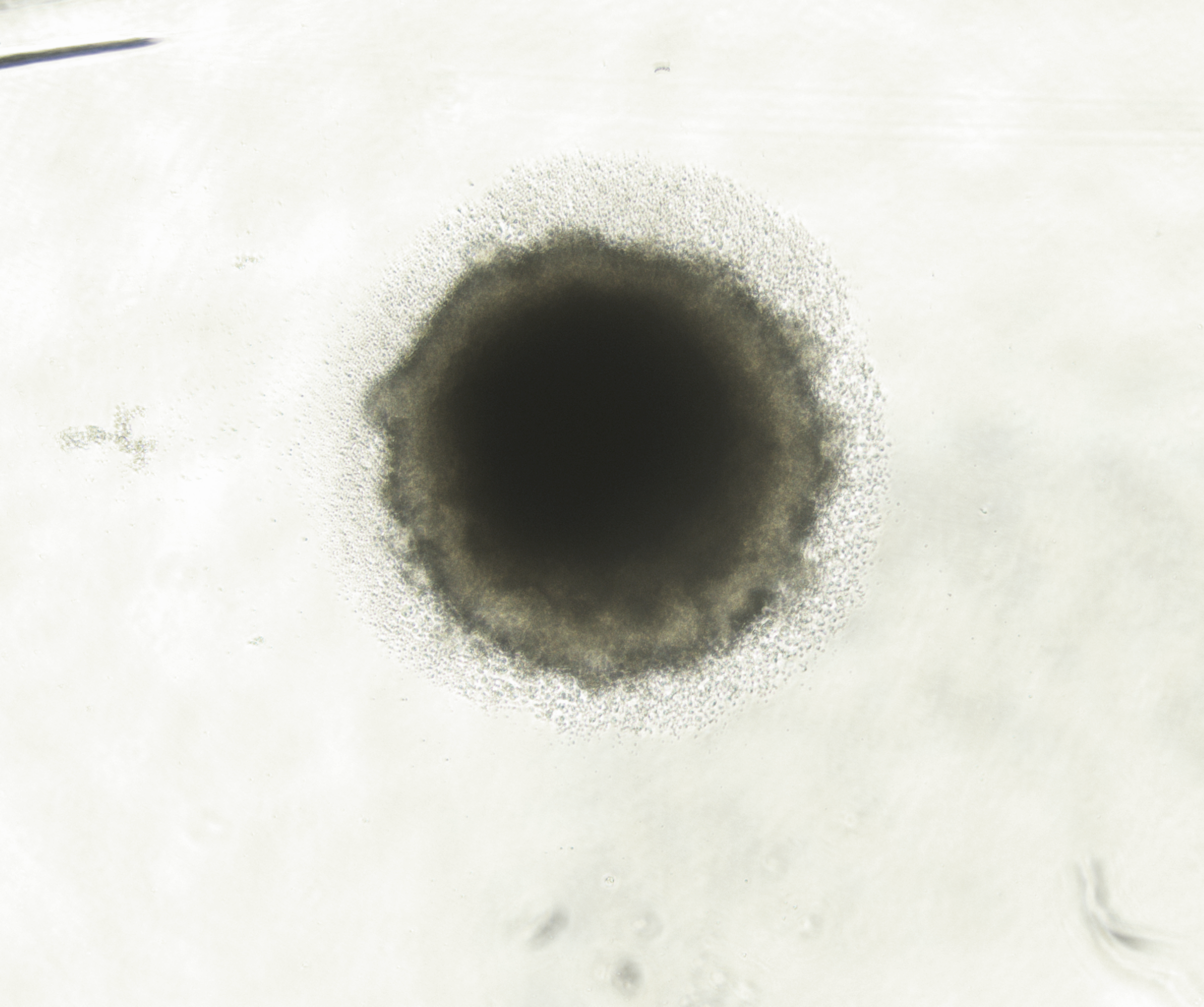

Supplement: Supplementary file 8 — Source data Fig. 2 [file 44321_2025_302_MOESM8_ESM.zip › Figure 2/2E/H9-Day10.tif]

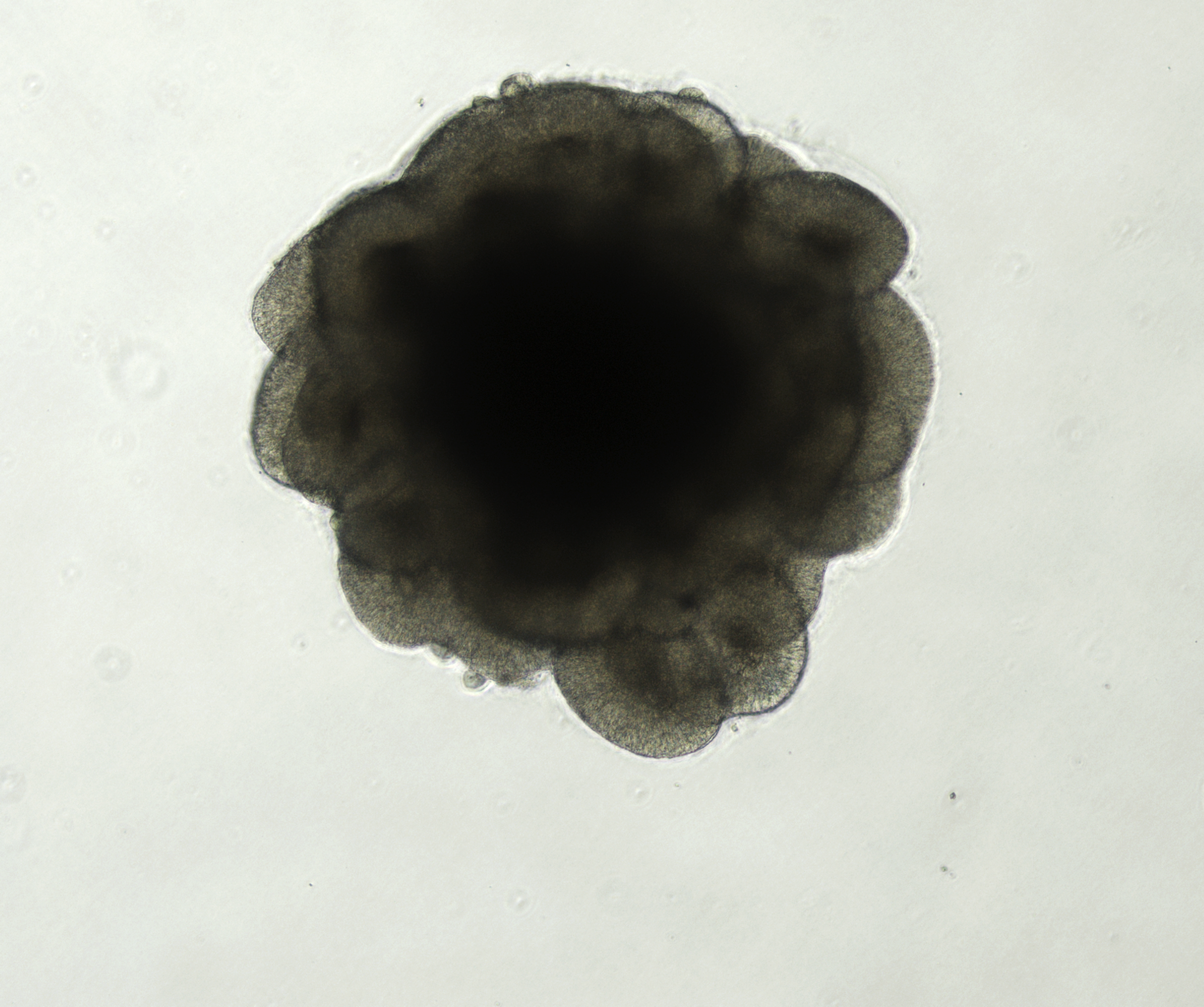

Supplement: Supplementary file 8 — Source data Fig. 2 [file 44321_2025_302_MOESM8_ESM.zip › Figure 2/2E/H9-Day15.tif]

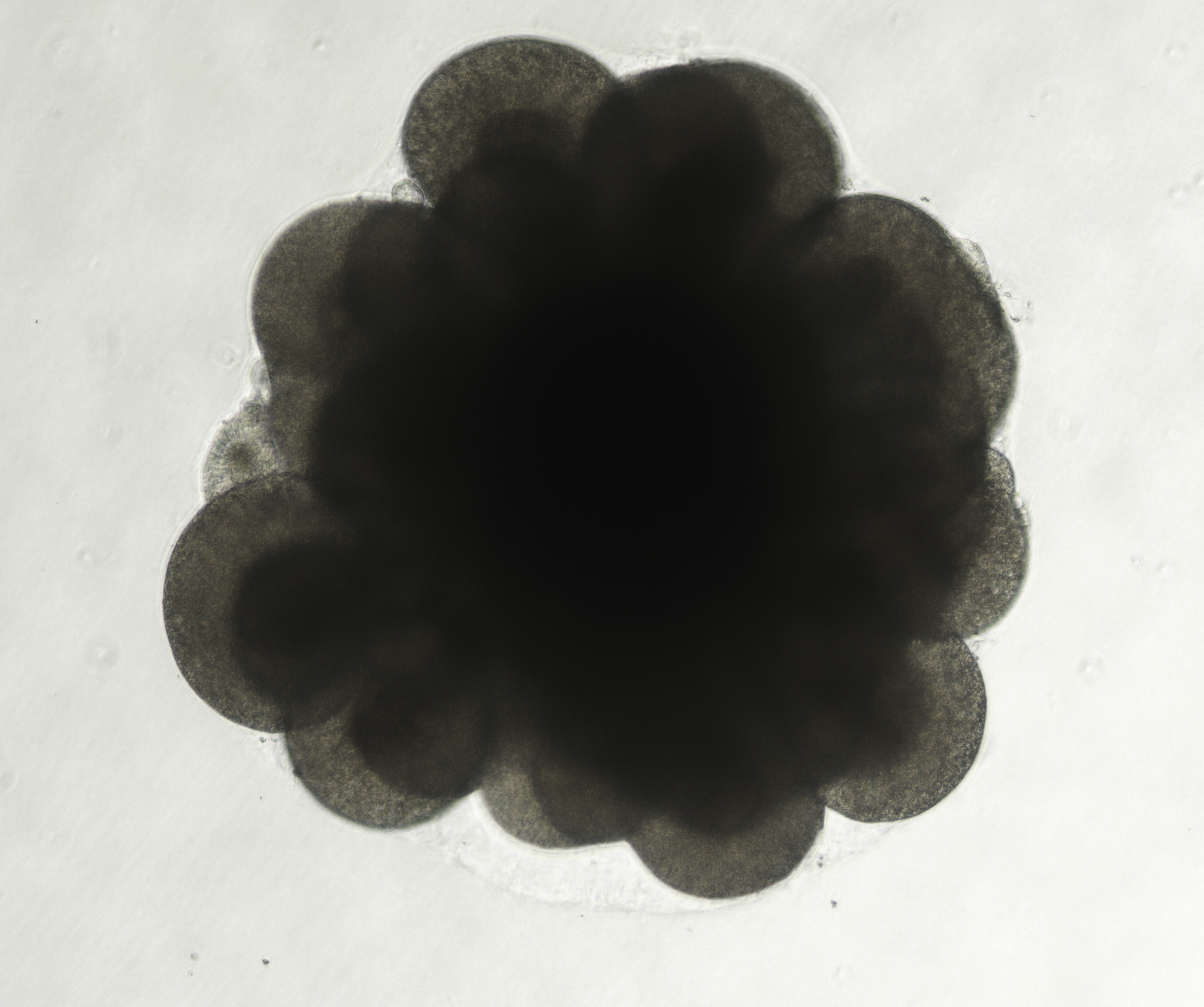

Supplement: Supplementary file 8 — Source data Fig. 2 [file 44321_2025_302_MOESM8_ESM.zip › Figure 2/2E/H9-Day20.tif]

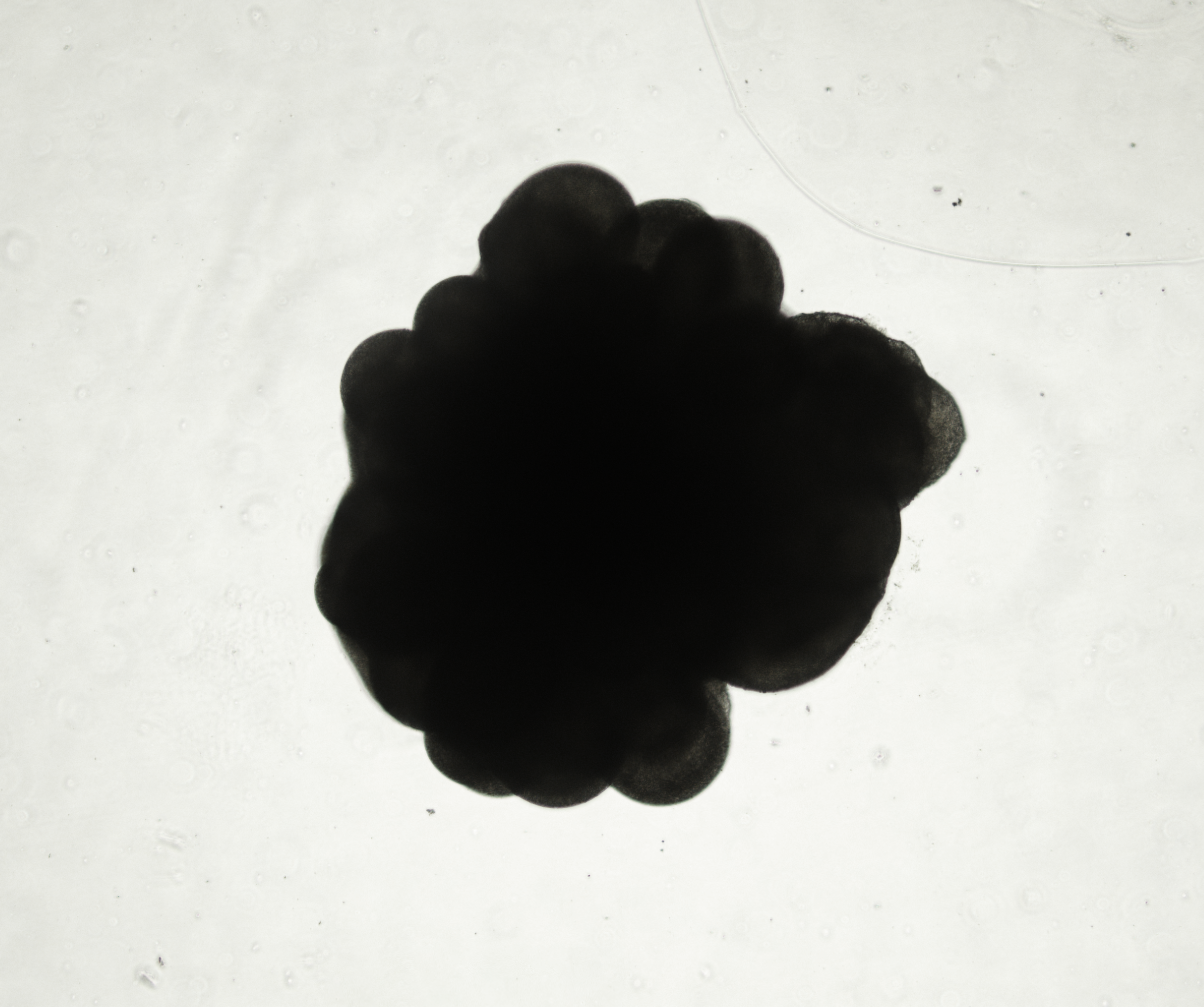

Supplement: Supplementary file 8 — Source data Fig. 2 [file 44321_2025_302_MOESM8_ESM.zip › Figure 2/2E/H9-Day25.tif]

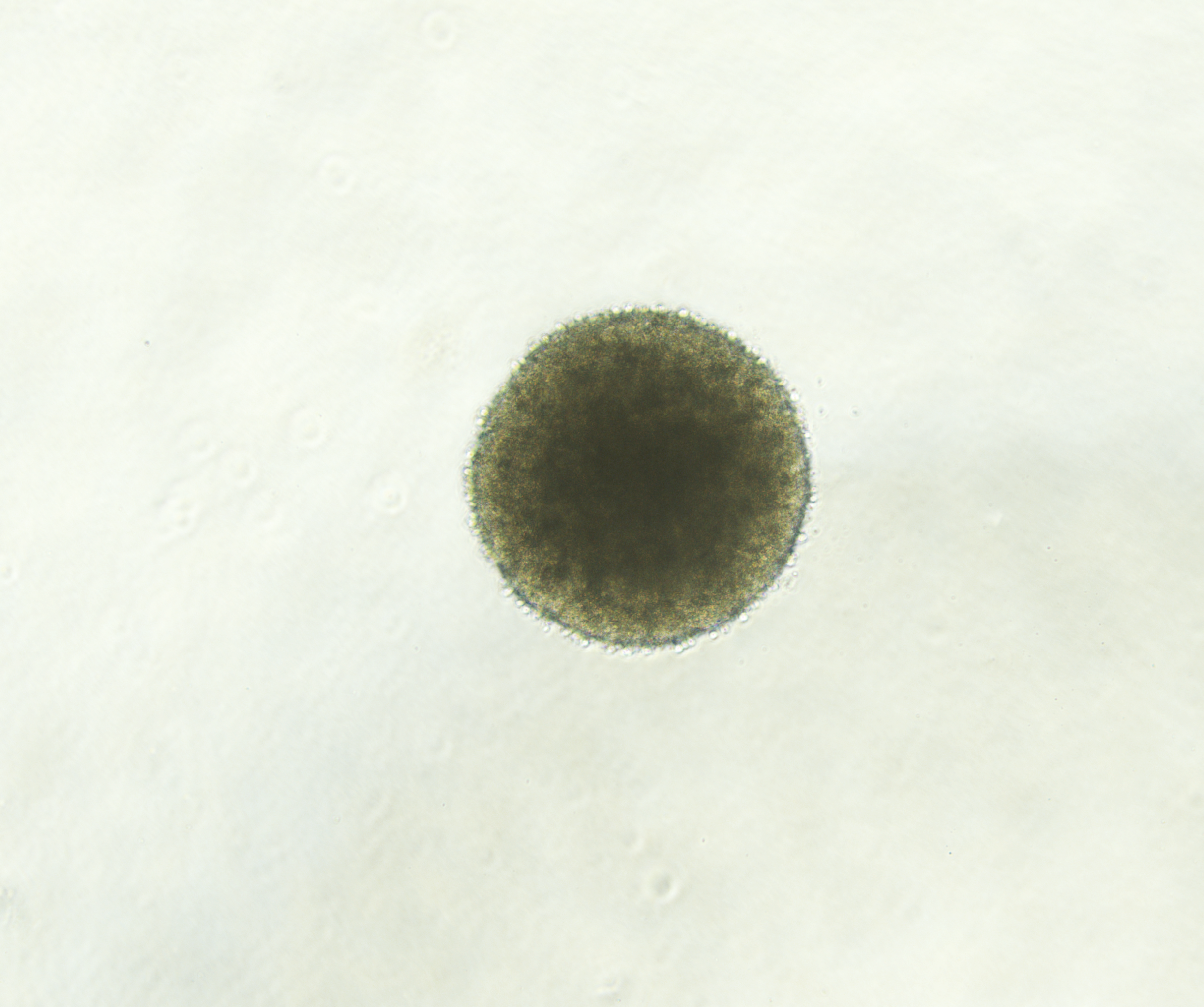

Supplement: Supplementary file 8 — Source data Fig. 2 [file 44321_2025_302_MOESM8_ESM.zip › Figure 2/2E/H9-Day3.tif]

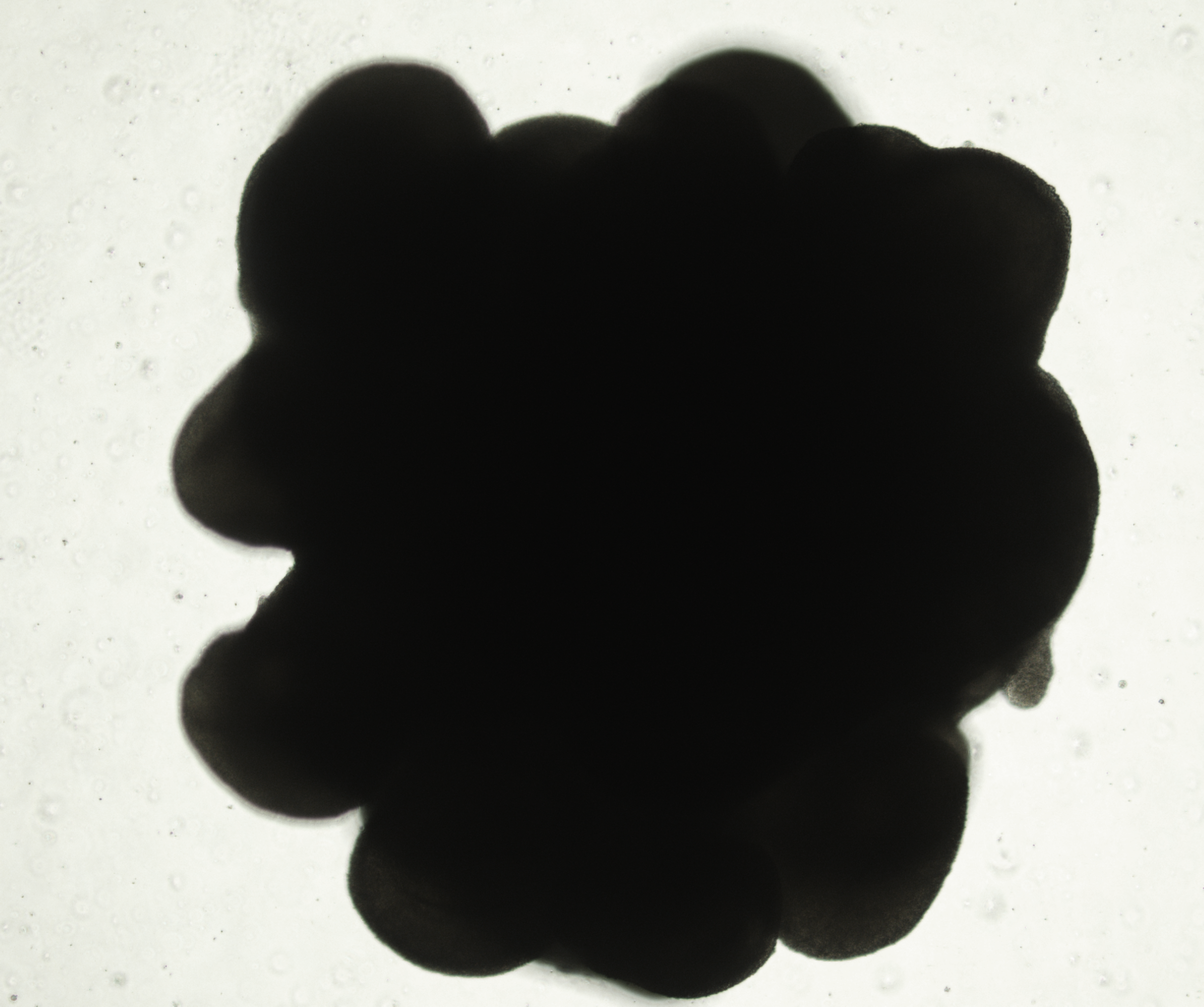

Supplement: Supplementary file 8 — Source data Fig. 2 [file 44321_2025_302_MOESM8_ESM.zip › Figure 2/2E/H9-Day35.tif]

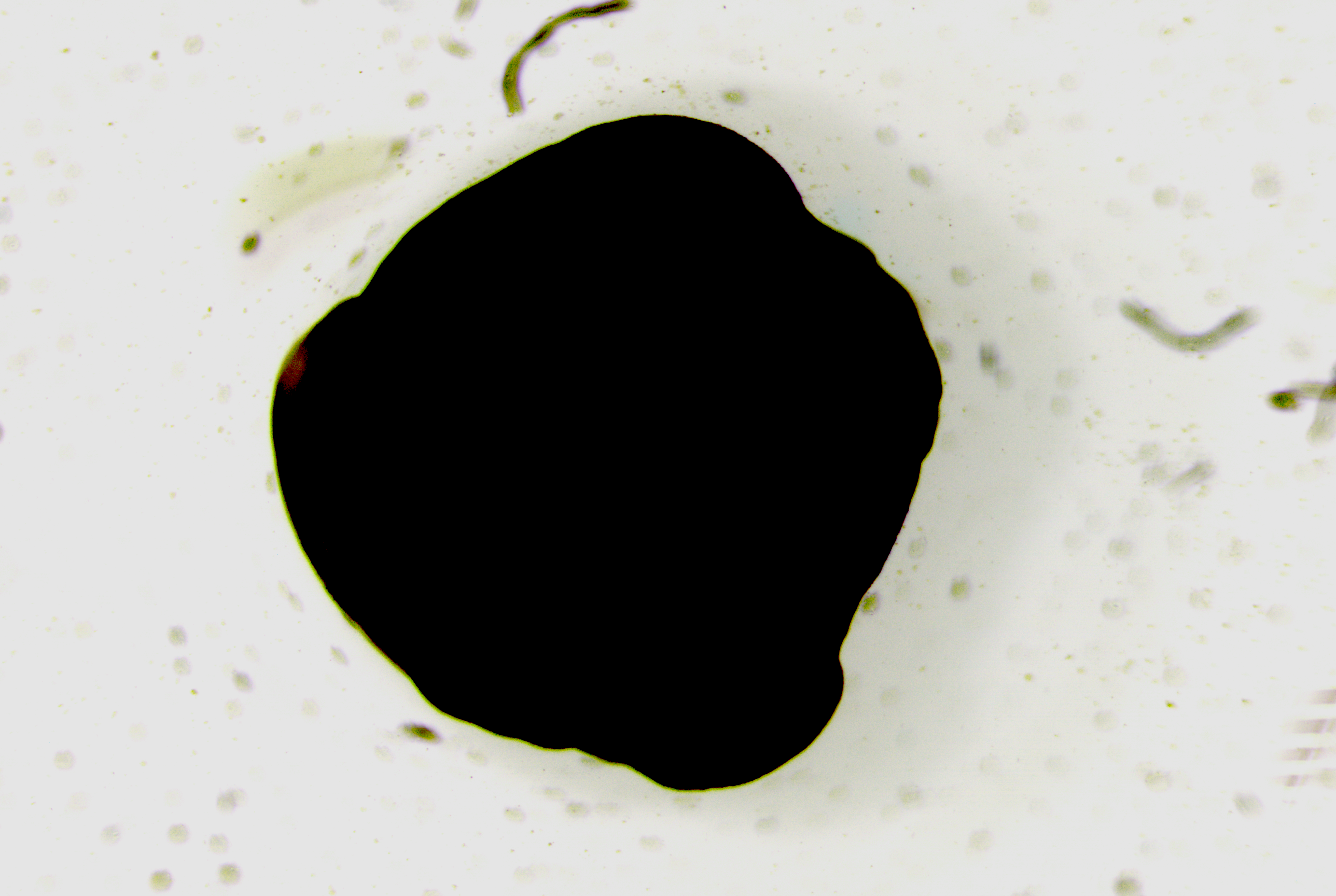

Supplement: Supplementary file 8 — Source data Fig. 2 [file 44321_2025_302_MOESM8_ESM.zip › Figure 2/2E/H9-Day45.png]

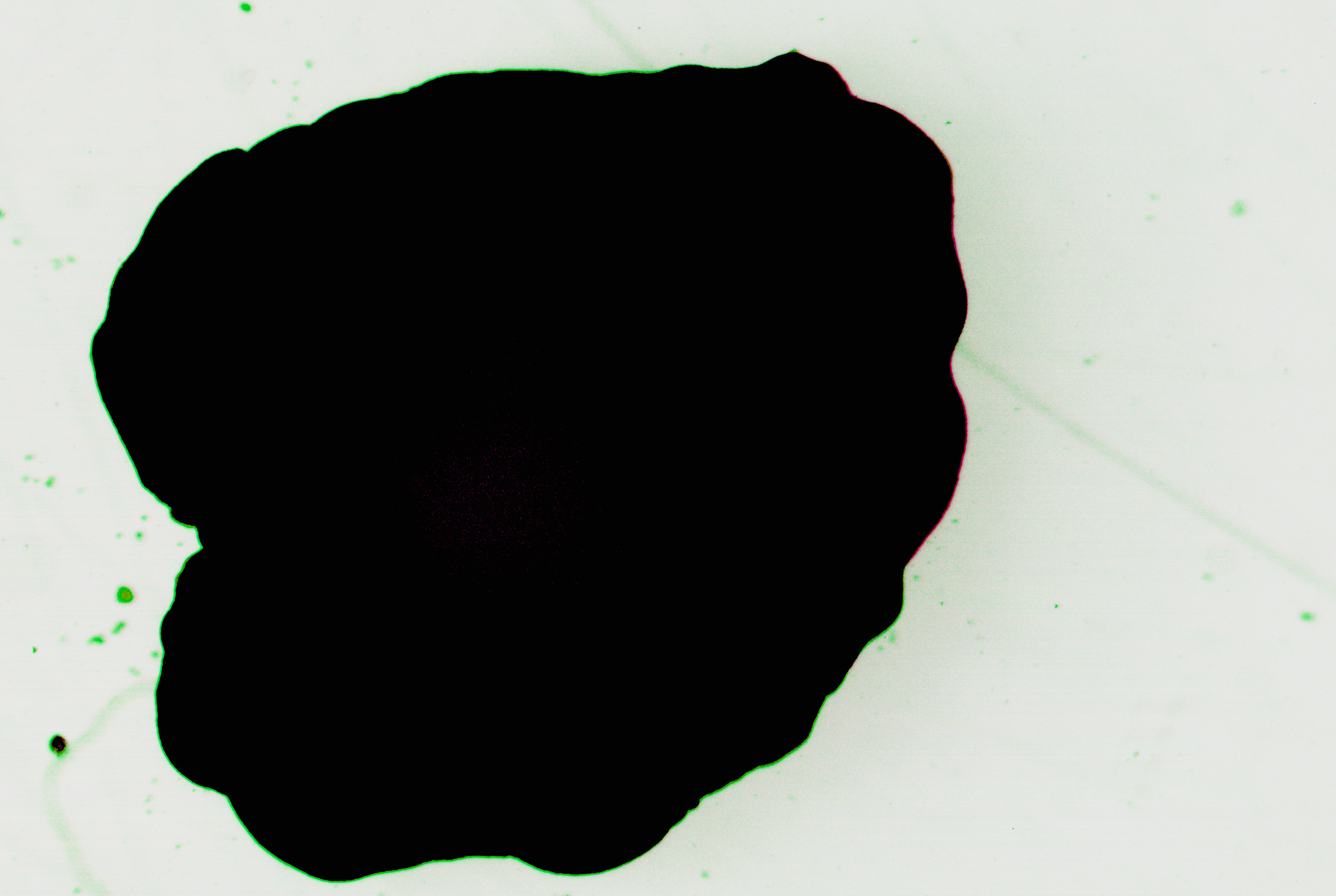

Supplement: Supplementary file 8 — Source data Fig. 2 [file 44321_2025_302_MOESM8_ESM.zip › Figure 2/2E/H9-Day60.png]

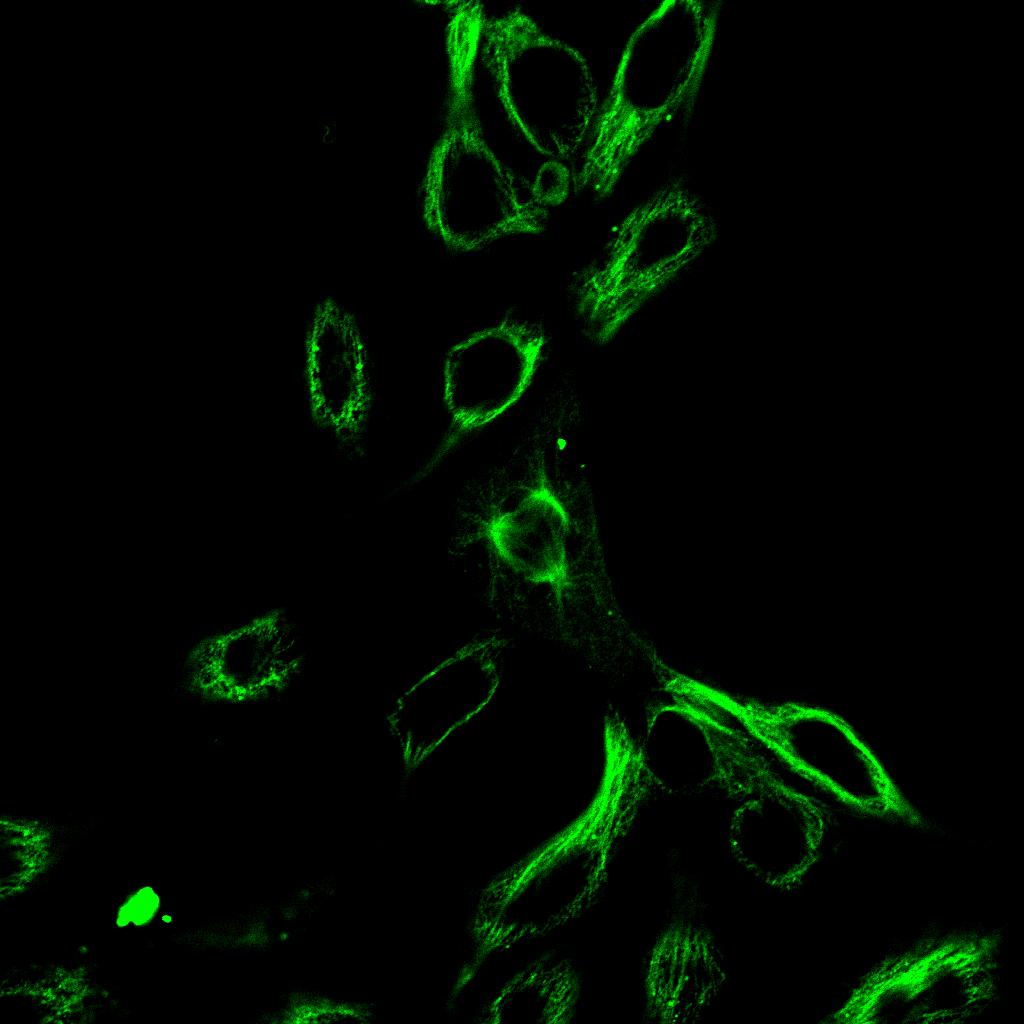

Supplement: Supplementary file 9 — Figure EV1 Source Data [file 44321_2025_302_MOESM9_ESM.zip › Figure EV1/EV1E/#7-5-atubulin.tif]

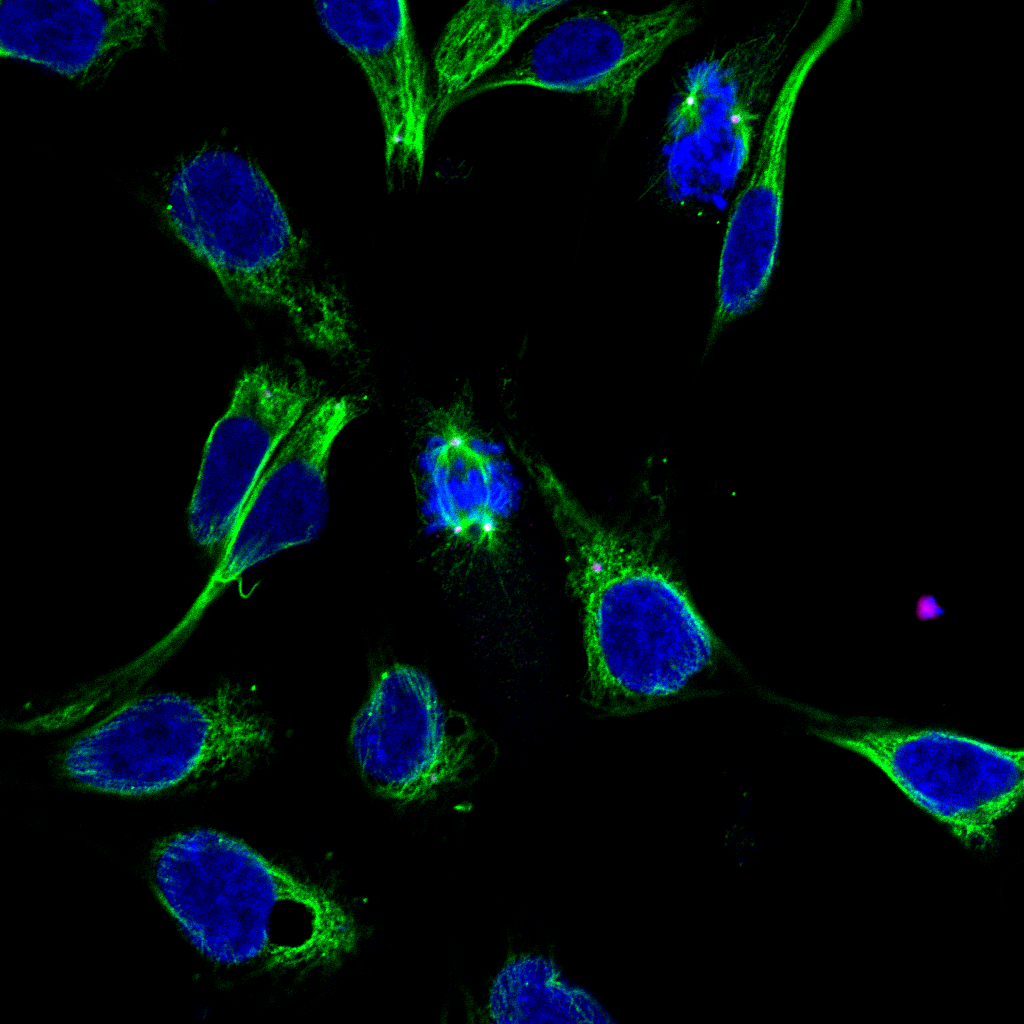

Supplement: Supplementary file 9 — Figure EV1 Source Data [file 44321_2025_302_MOESM9_ESM.zip › Figure EV1/EV1E/#12-3-merge.tif]

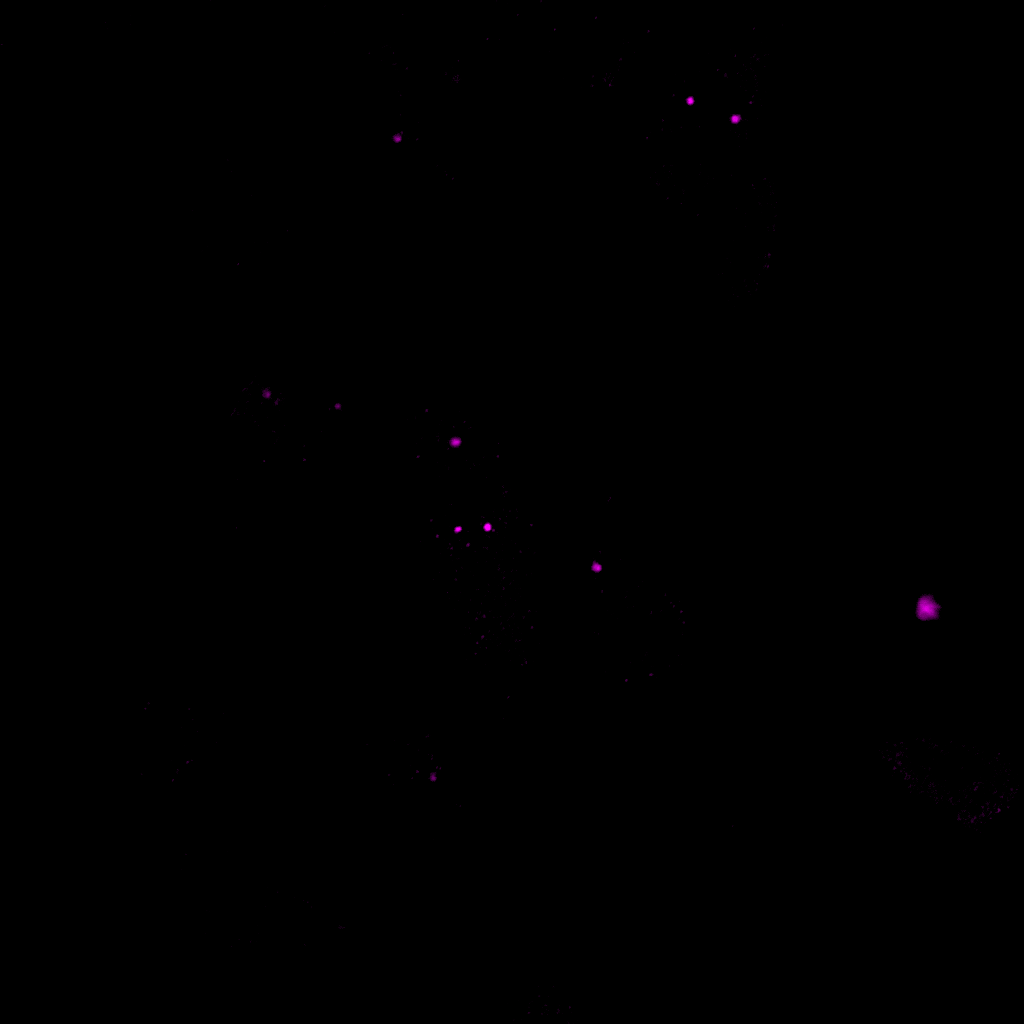

Supplement: Supplementary file 9 — Figure EV1 Source Data [file 44321_2025_302_MOESM9_ESM.zip › Figure EV1/EV1E/#12-3-PCNT.tif]

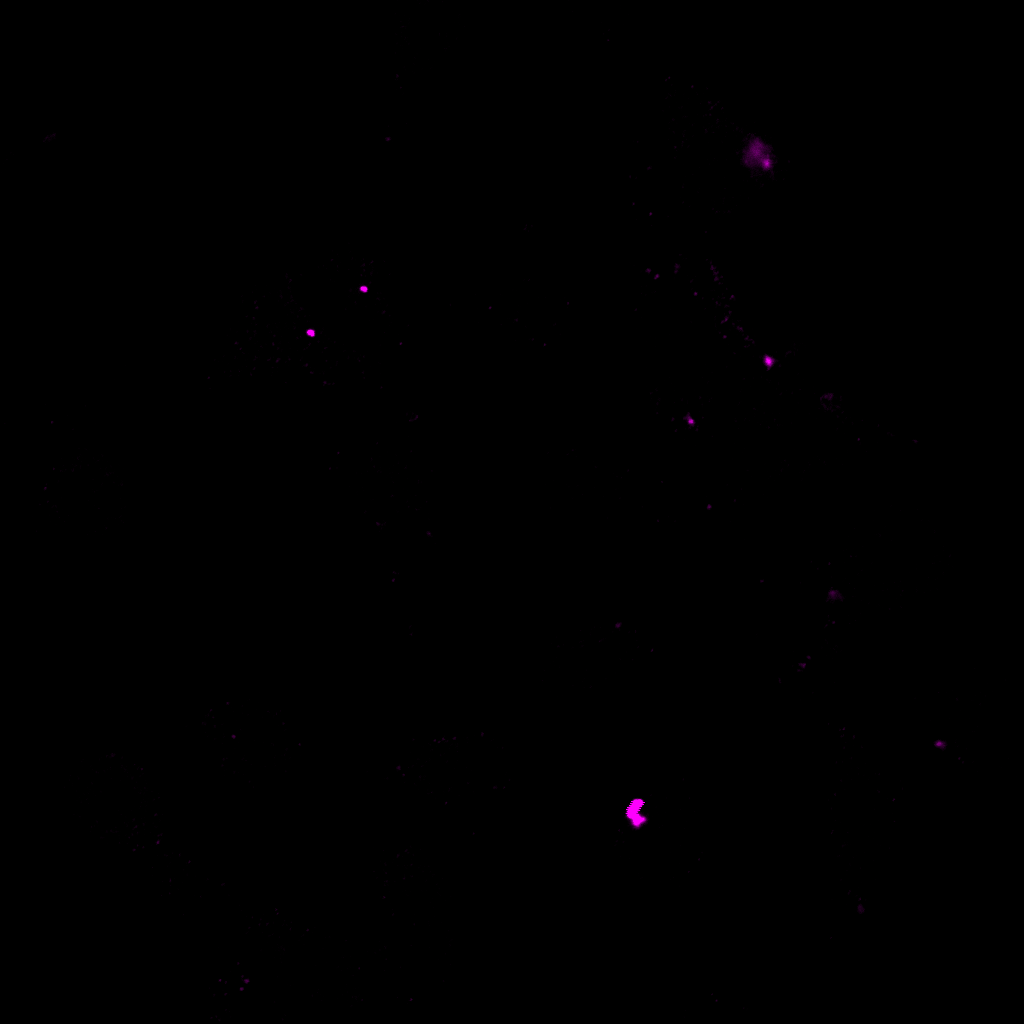

Supplement: Supplementary file 9 — Figure EV1 Source Data [file 44321_2025_302_MOESM9_ESM.zip › Figure EV1/EV1E/H9-PCNT.tif]

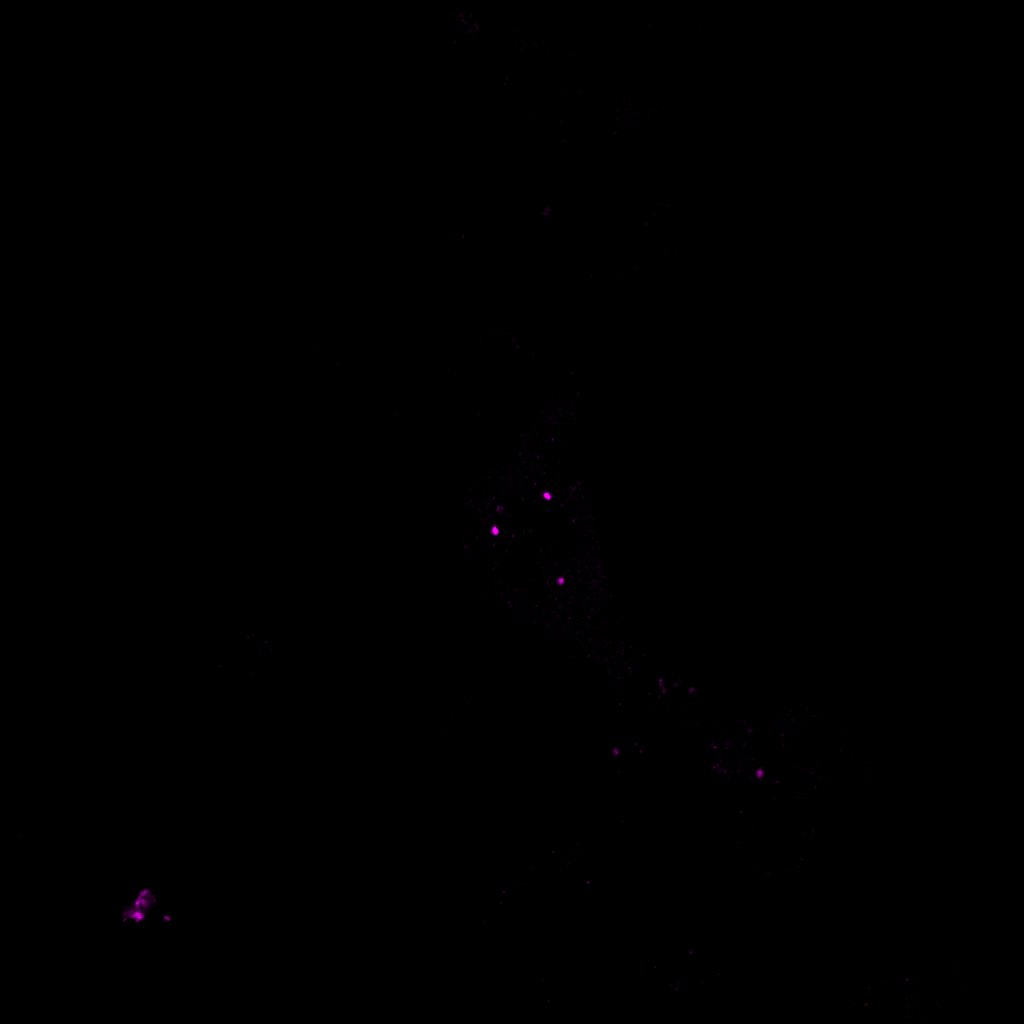

Supplement: Supplementary file 9 — Figure EV1 Source Data [file 44321_2025_302_MOESM9_ESM.zip › Figure EV1/EV1E/#7-5-PCNT.tif]

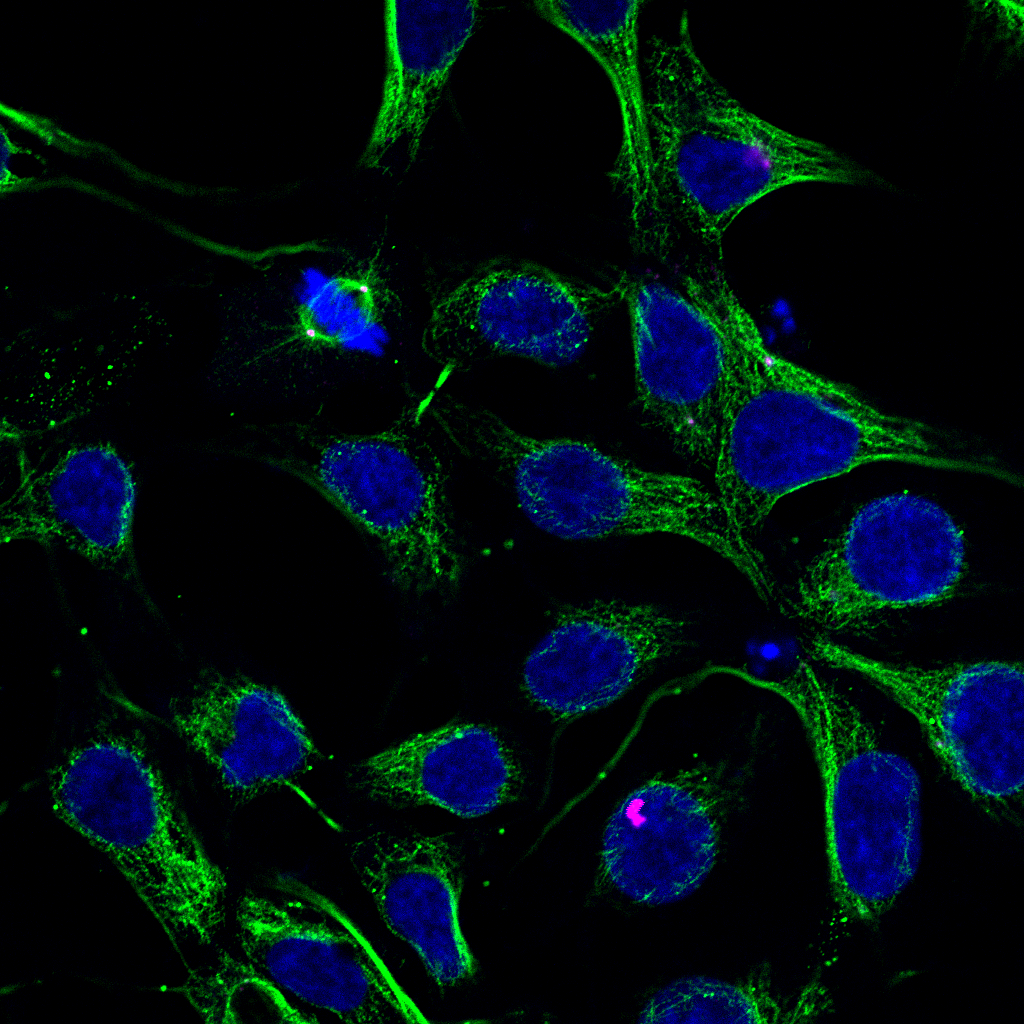

Supplement: Supplementary file 9 — Figure EV1 Source Data [file 44321_2025_302_MOESM9_ESM.zip › Figure EV1/EV1E/H9-merge.tif]

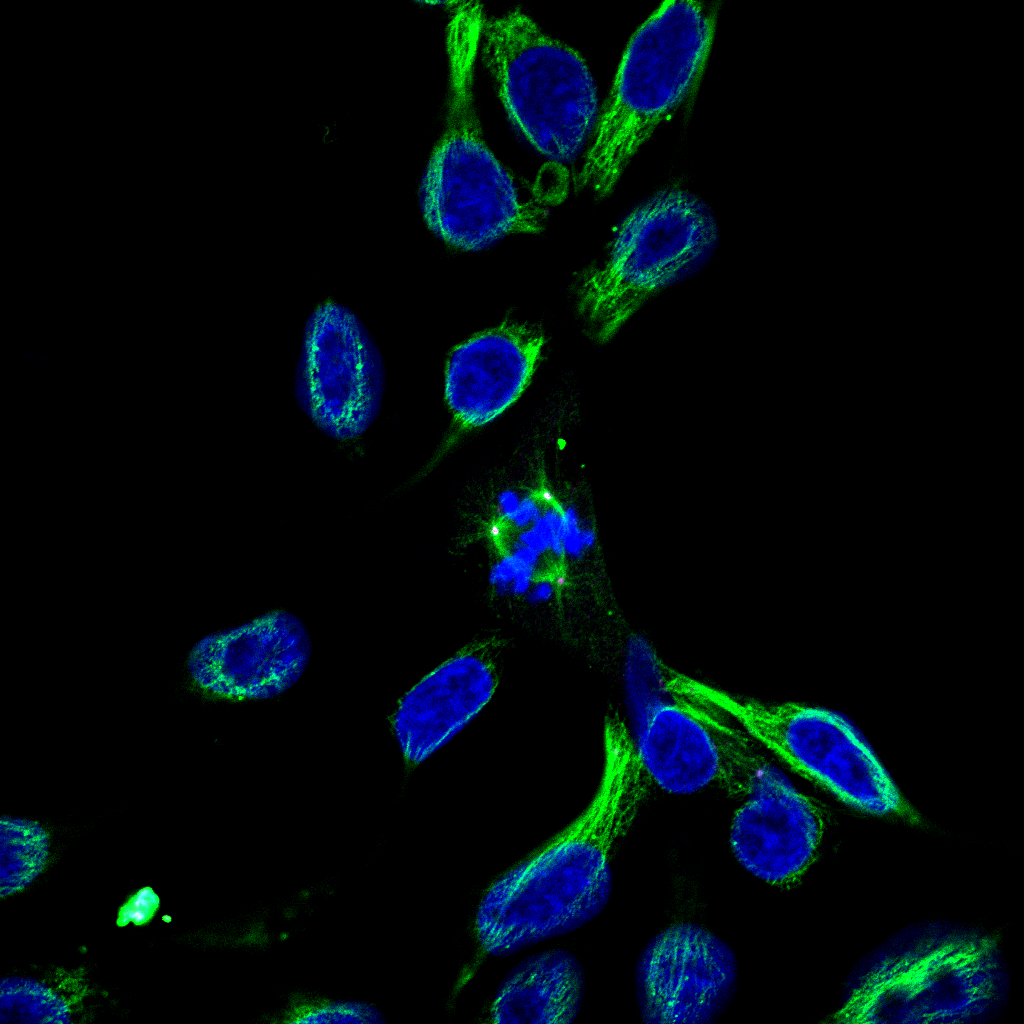

Supplement: Supplementary file 9 — Figure EV1 Source Data [file 44321_2025_302_MOESM9_ESM.zip › Figure EV1/EV1E/#7-5-merge.tif]

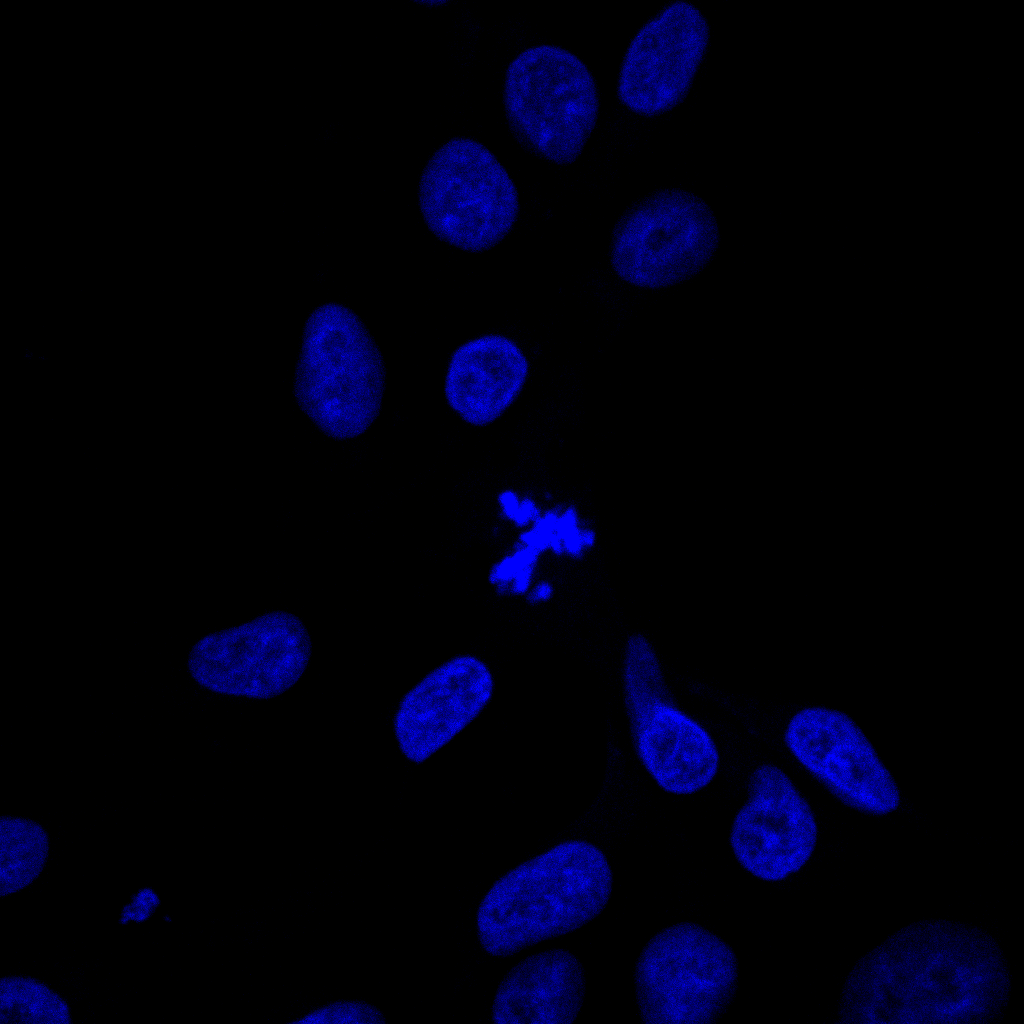

Supplement: Supplementary file 9 — Figure EV1 Source Data [file 44321_2025_302_MOESM9_ESM.zip › Figure EV1/EV1E/#7-5-DAPI.tif]

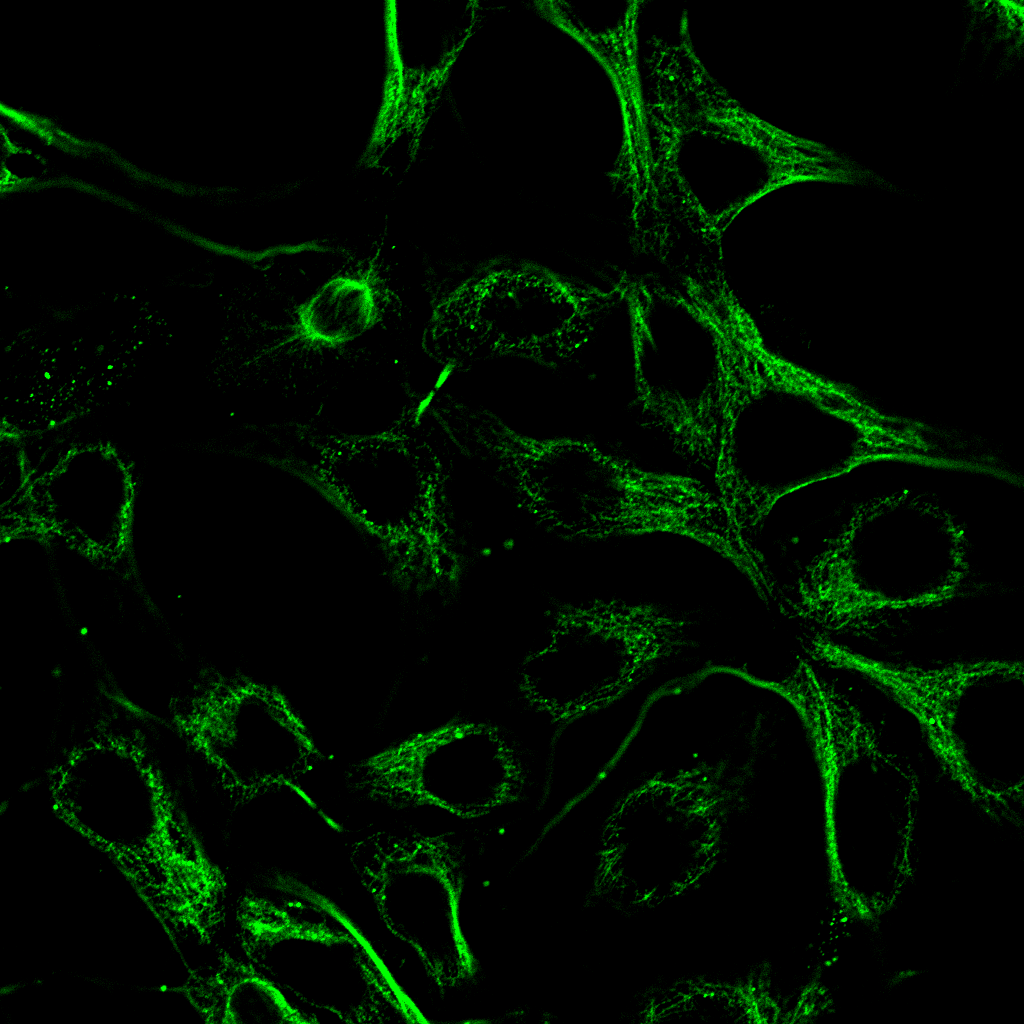

Supplement: Supplementary file 9 — Figure EV1 Source Data [file 44321_2025_302_MOESM9_ESM.zip › Figure EV1/EV1E/H9-atubulin.tif]

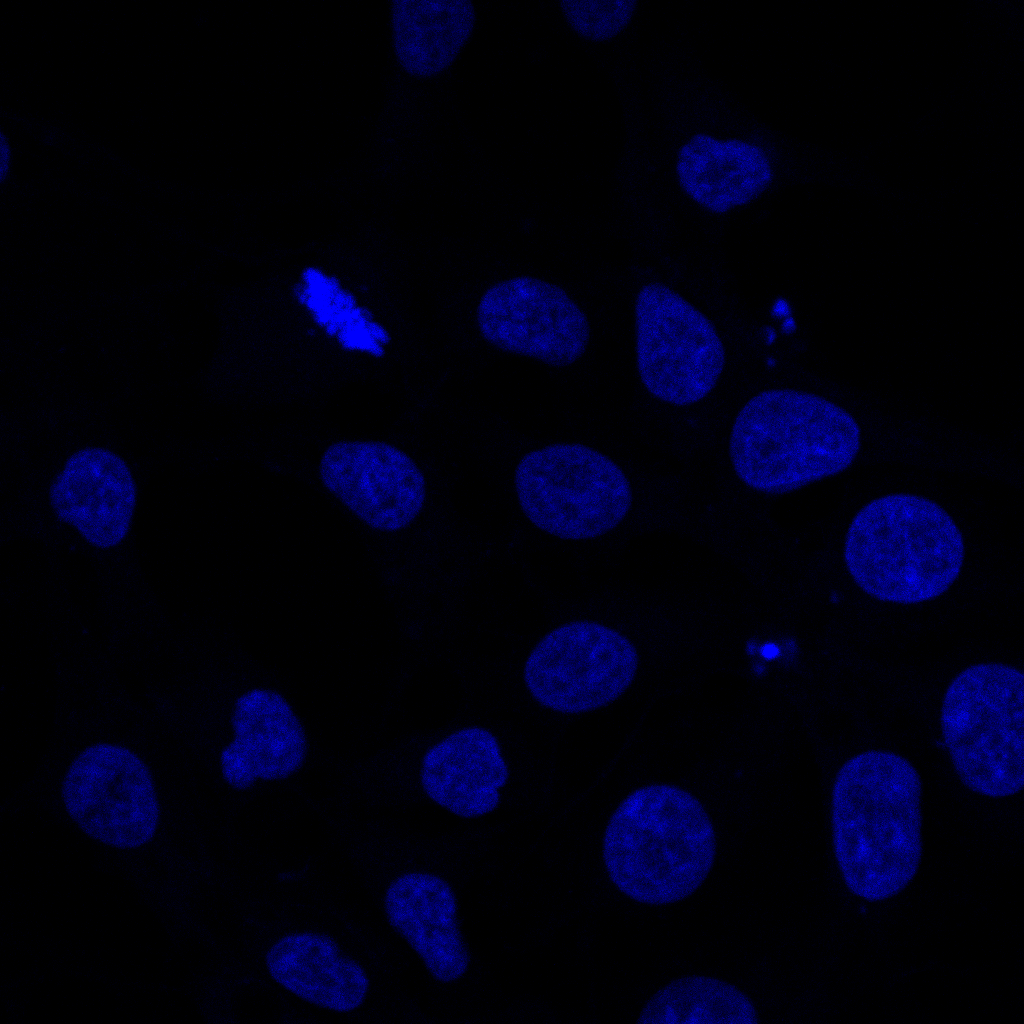

Supplement: Supplementary file 9 — Figure EV1 Source Data [file 44321_2025_302_MOESM9_ESM.zip › Figure EV1/EV1E/H9-DAPI.tif]

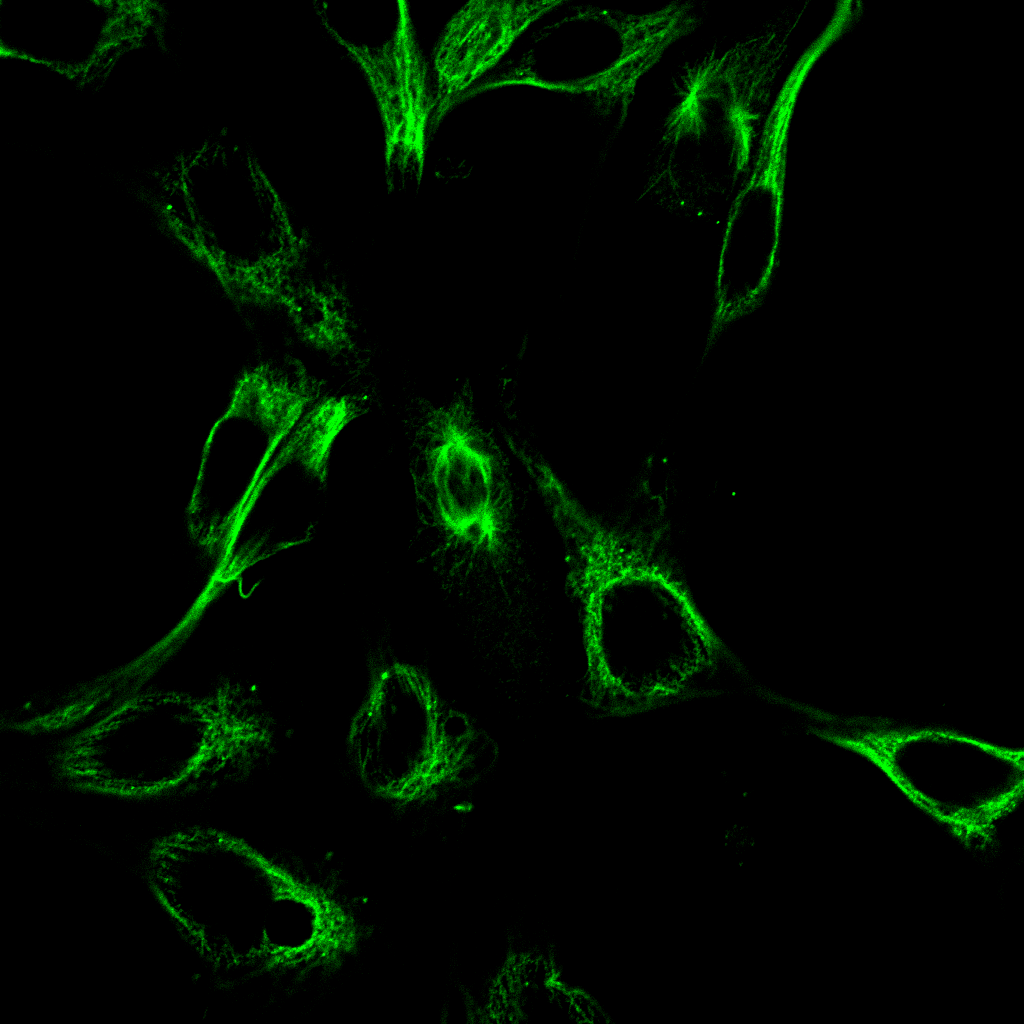

Supplement: Supplementary file 9 — Figure EV1 Source Data [file 44321_2025_302_MOESM9_ESM.zip › Figure EV1/EV1E/#12-3-atubulin.tif]

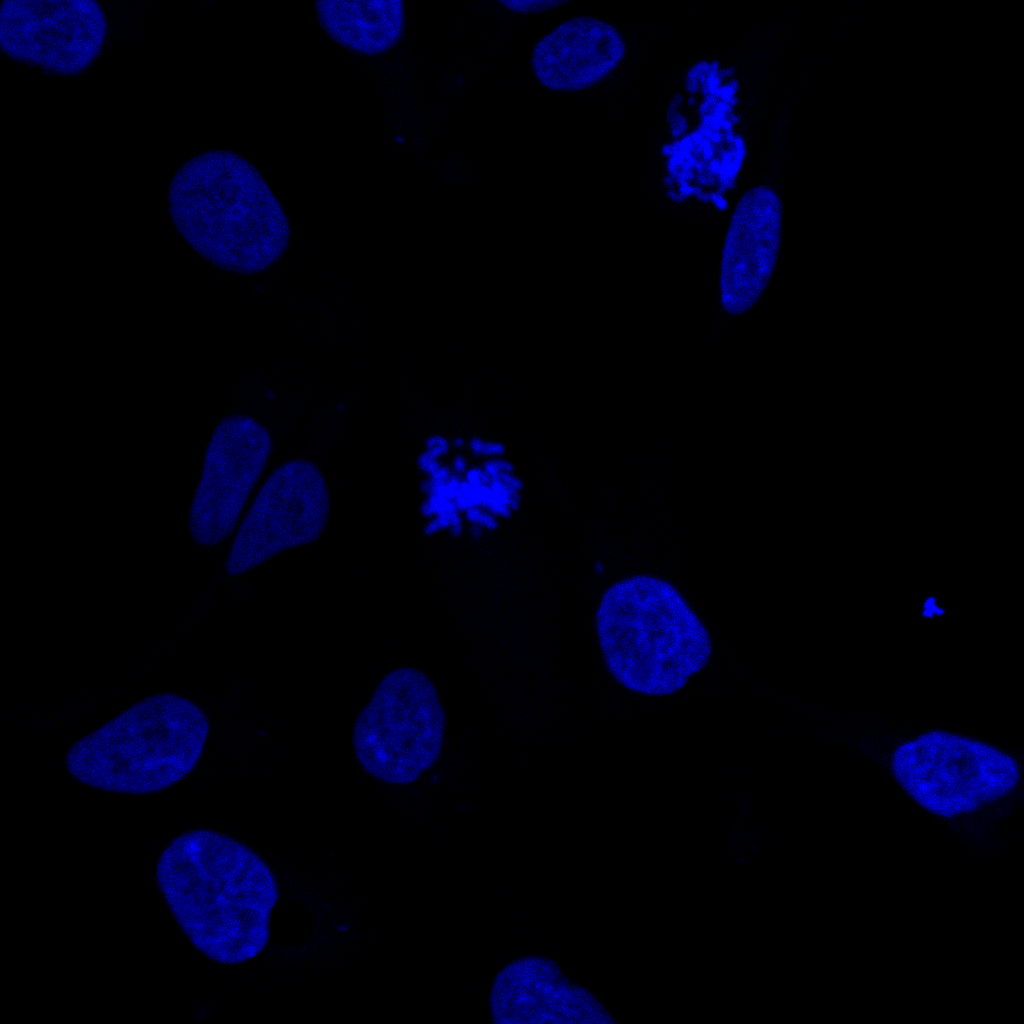

Supplement: Supplementary file 9 — Figure EV1 Source Data [file 44321_2025_302_MOESM9_ESM.zip › Figure EV1/EV1E/#12-3-DAPI.tif]

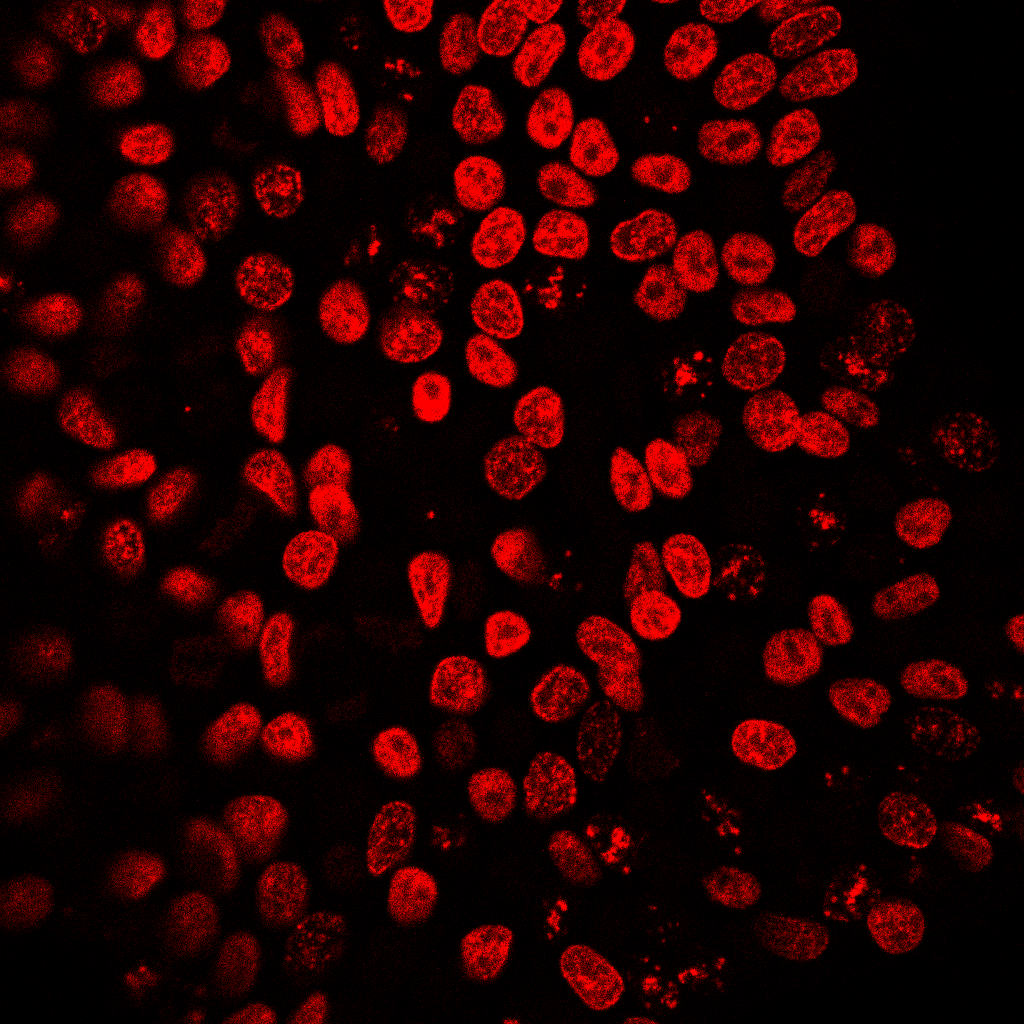

Supplement: Supplementary file 9 — Figure EV1 Source Data [file 44321_2025_302_MOESM9_ESM.zip › Figure EV1/EV1B/H9-EdU.tif]

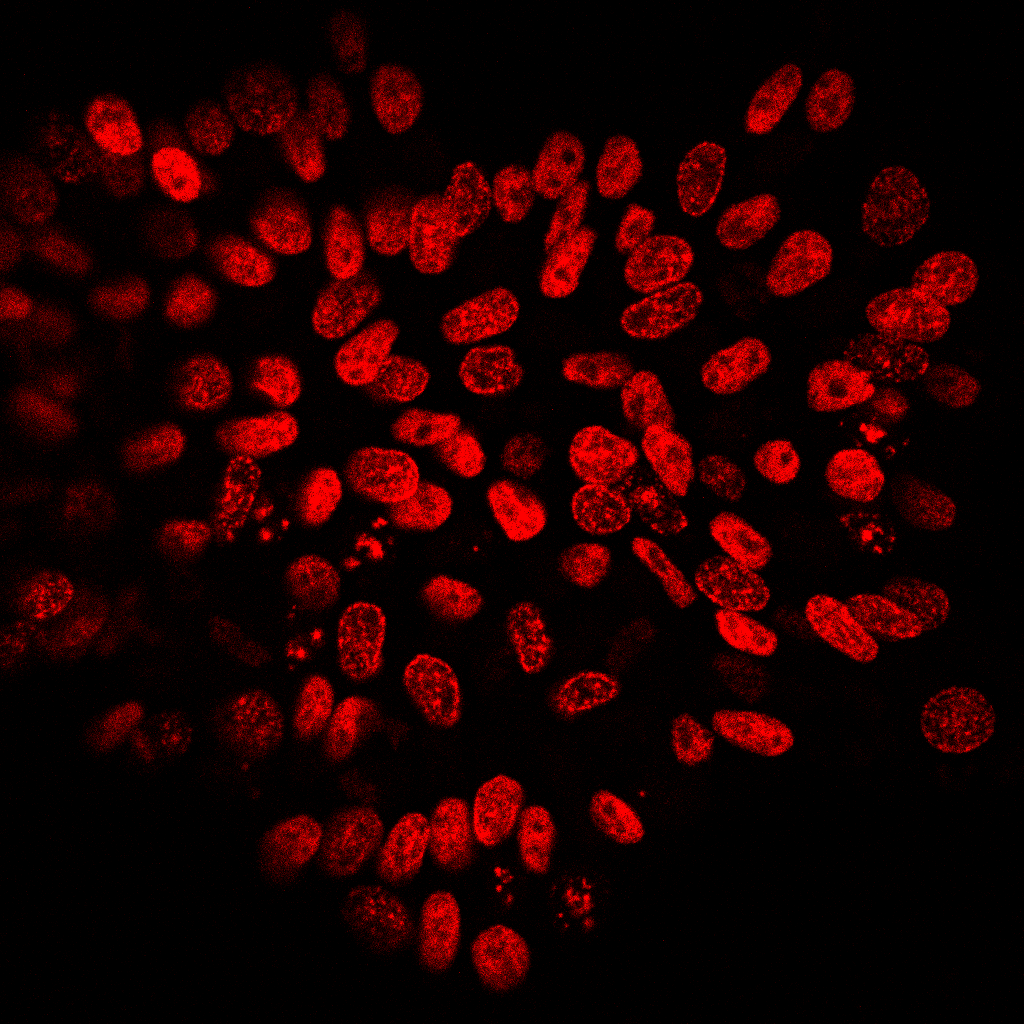

Supplement: Supplementary file 9 — Figure EV1 Source Data [file 44321_2025_302_MOESM9_ESM.zip › Figure EV1/EV1B/#12-3-EdU.tif]

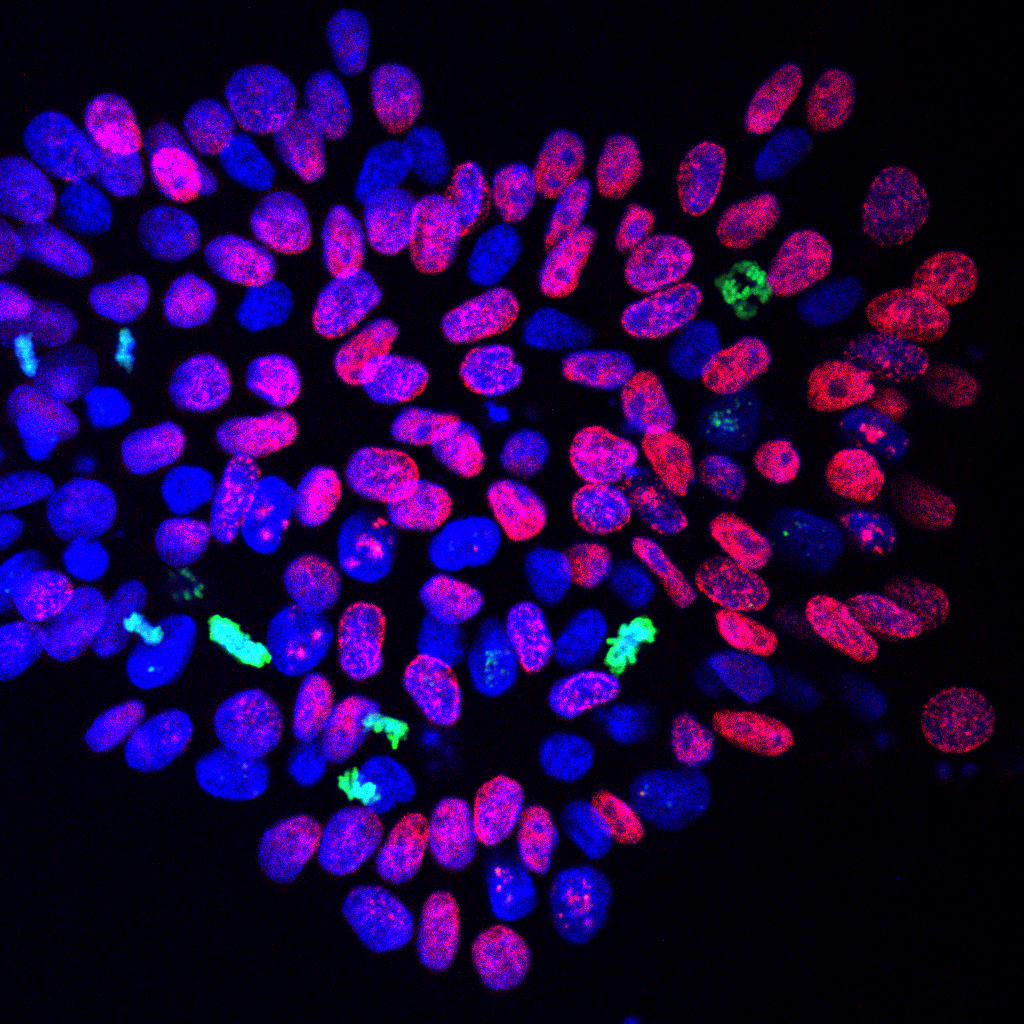

Supplement: Supplementary file 9 — Figure EV1 Source Data [file 44321_2025_302_MOESM9_ESM.zip › Figure EV1/EV1B/#12-3-merge.tif]

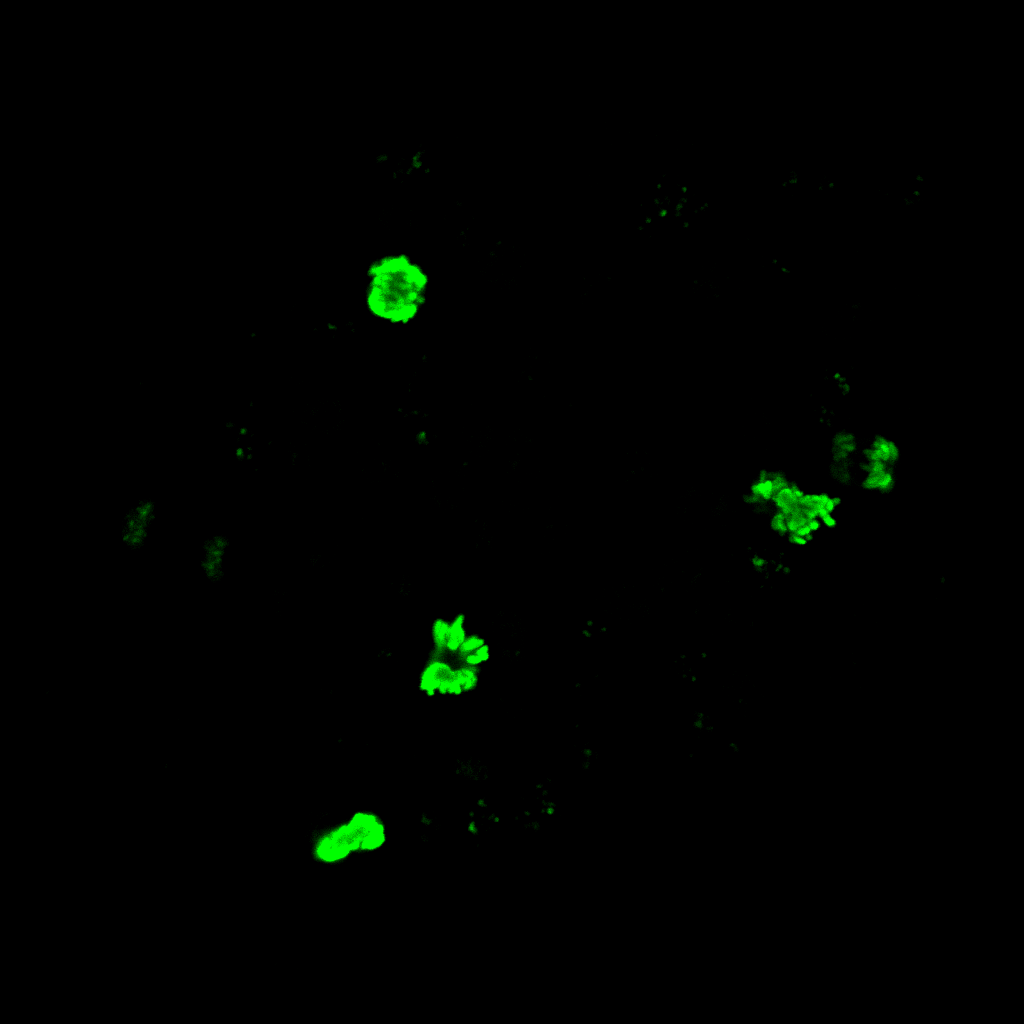

Supplement: Supplementary file 9 — Figure EV1 Source Data [file 44321_2025_302_MOESM9_ESM.zip › Figure EV1/EV1B/#7-5-PH3.tif]

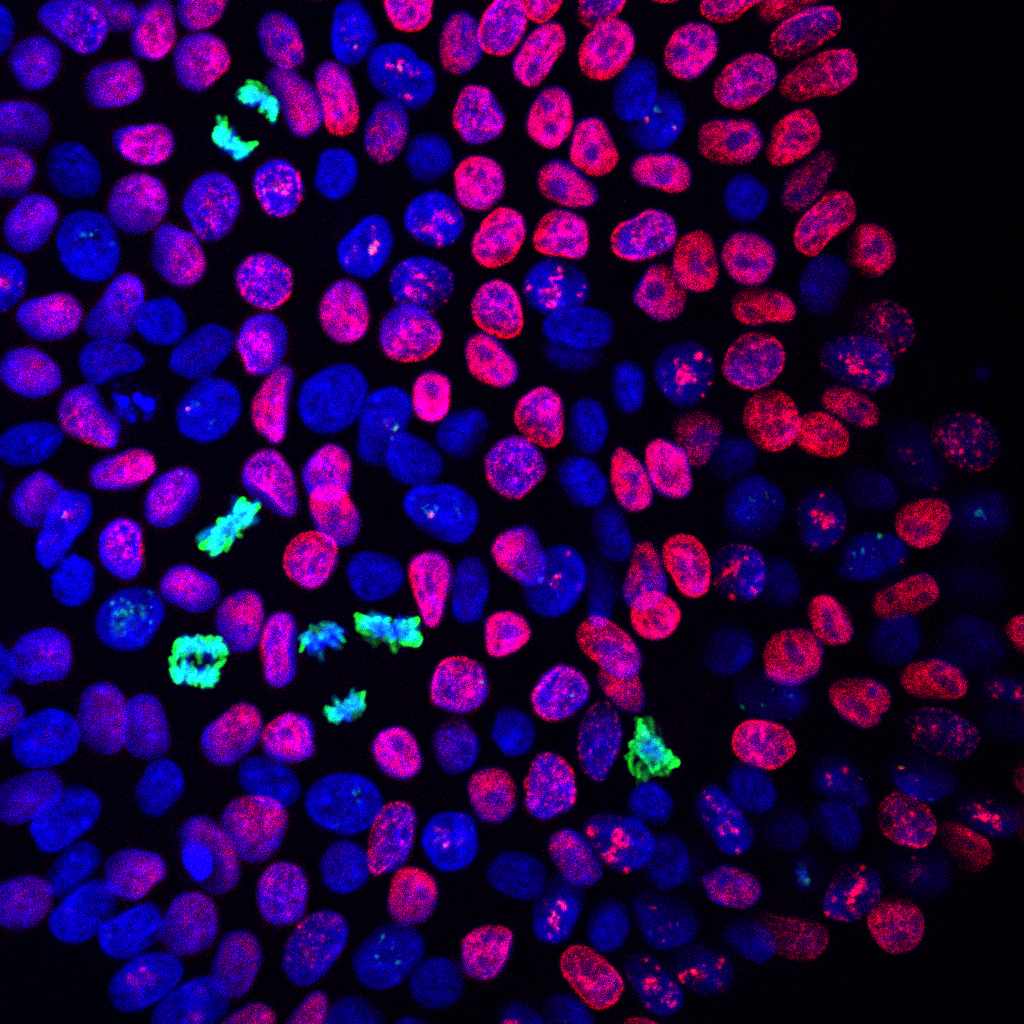

Supplement: Supplementary file 9 — Figure EV1 Source Data [file 44321_2025_302_MOESM9_ESM.zip › Figure EV1/EV1B/H9-merge.tif]

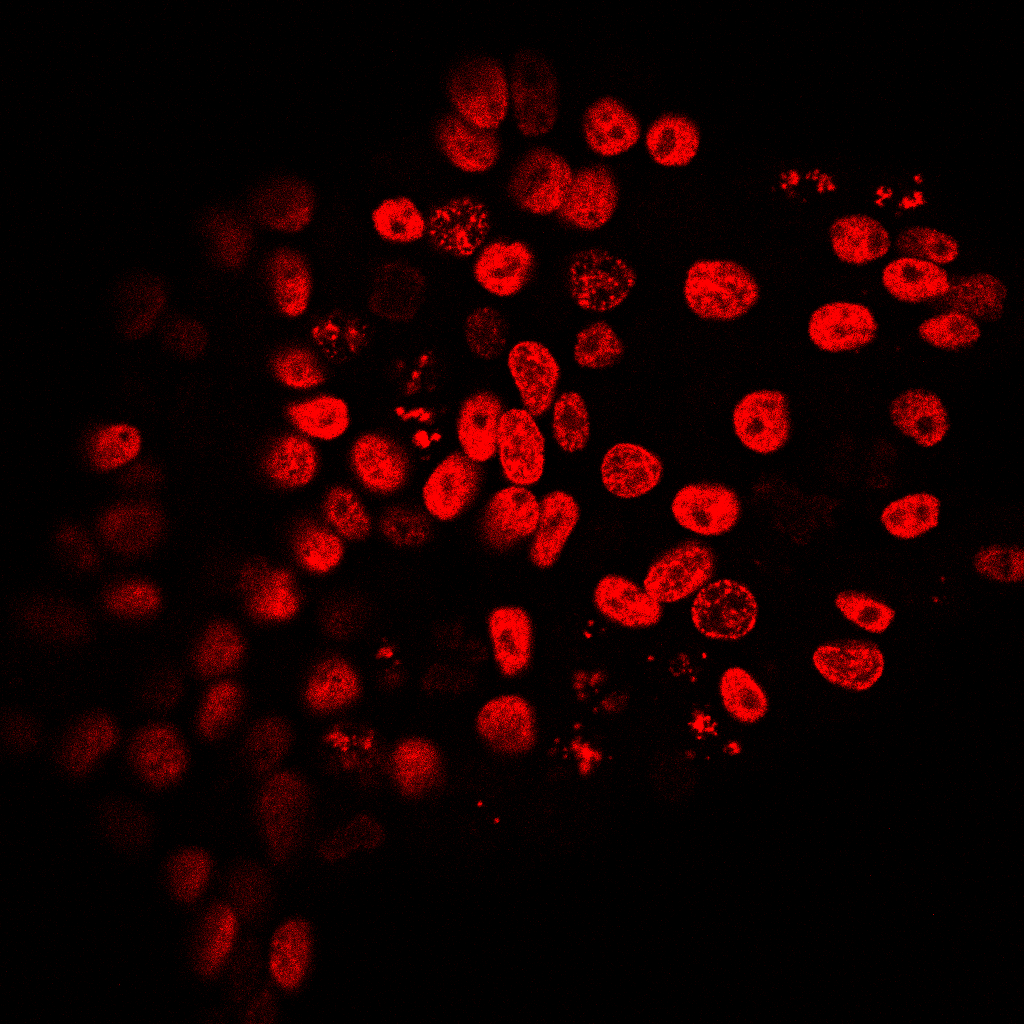

Supplement: Supplementary file 9 — Figure EV1 Source Data [file 44321_2025_302_MOESM9_ESM.zip › Figure EV1/EV1B/#7-5-EdU.tif]

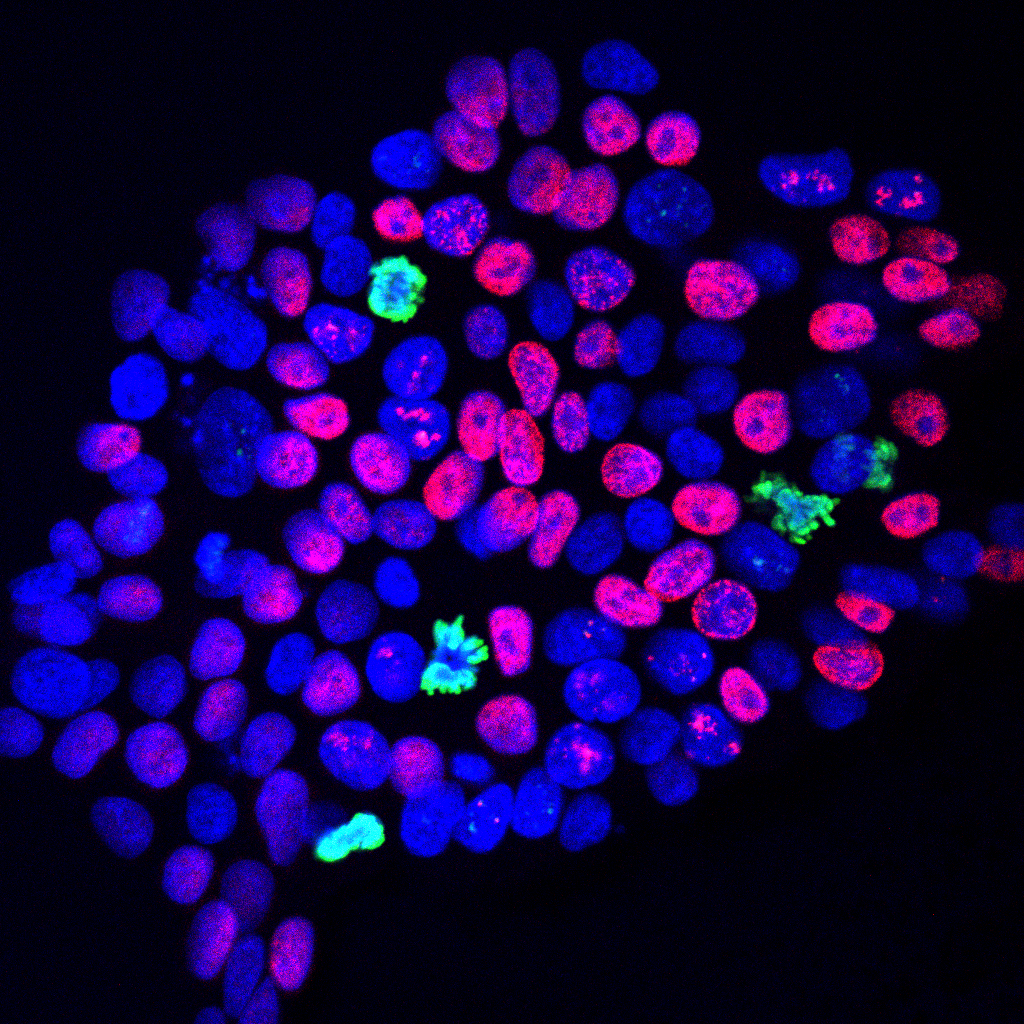

Supplement: Supplementary file 9 — Figure EV1 Source Data [file 44321_2025_302_MOESM9_ESM.zip › Figure EV1/EV1B/#7-5-merge.tif]

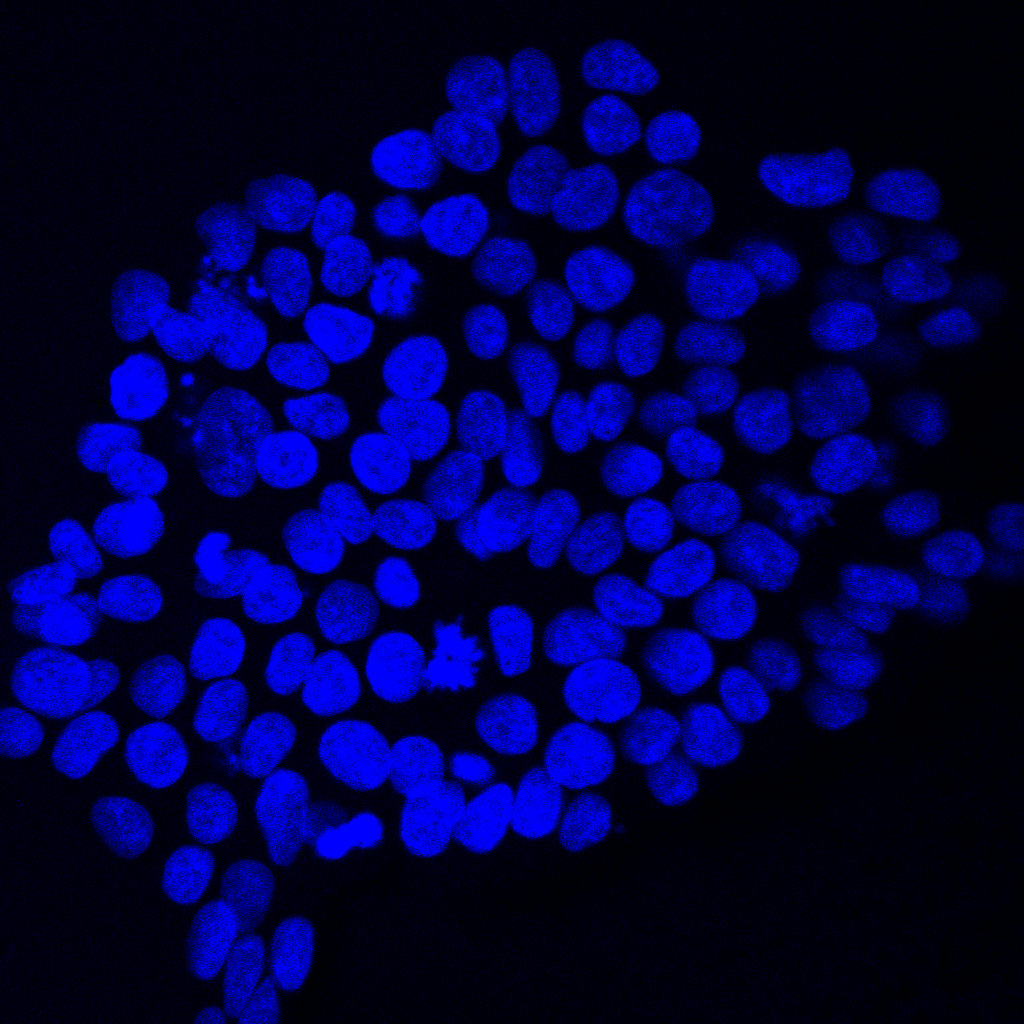

Supplement: Supplementary file 9 — Figure EV1 Source Data [file 44321_2025_302_MOESM9_ESM.zip › Figure EV1/EV1B/#7-5-DAPI.tif]

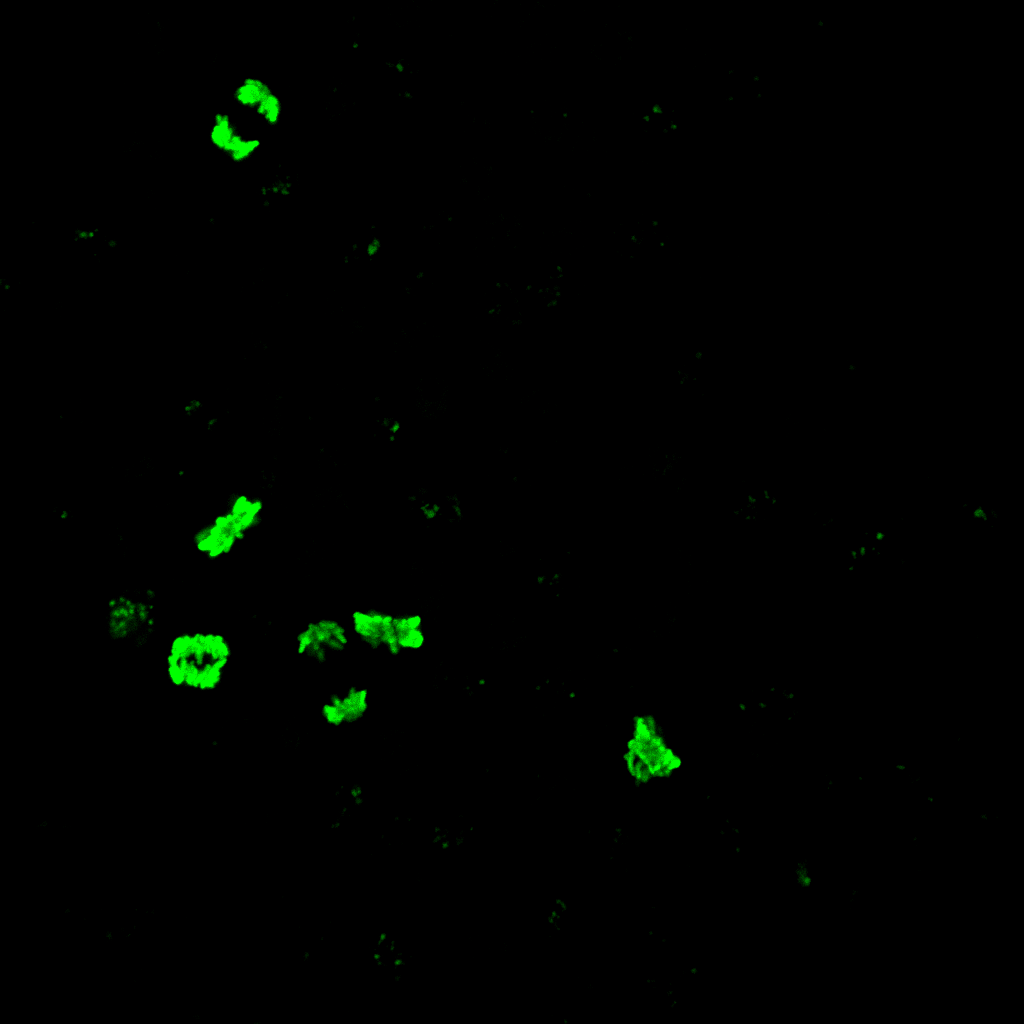

Supplement: Supplementary file 9 — Figure EV1 Source Data [file 44321_2025_302_MOESM9_ESM.zip › Figure EV1/EV1B/H9-PH3.tif]

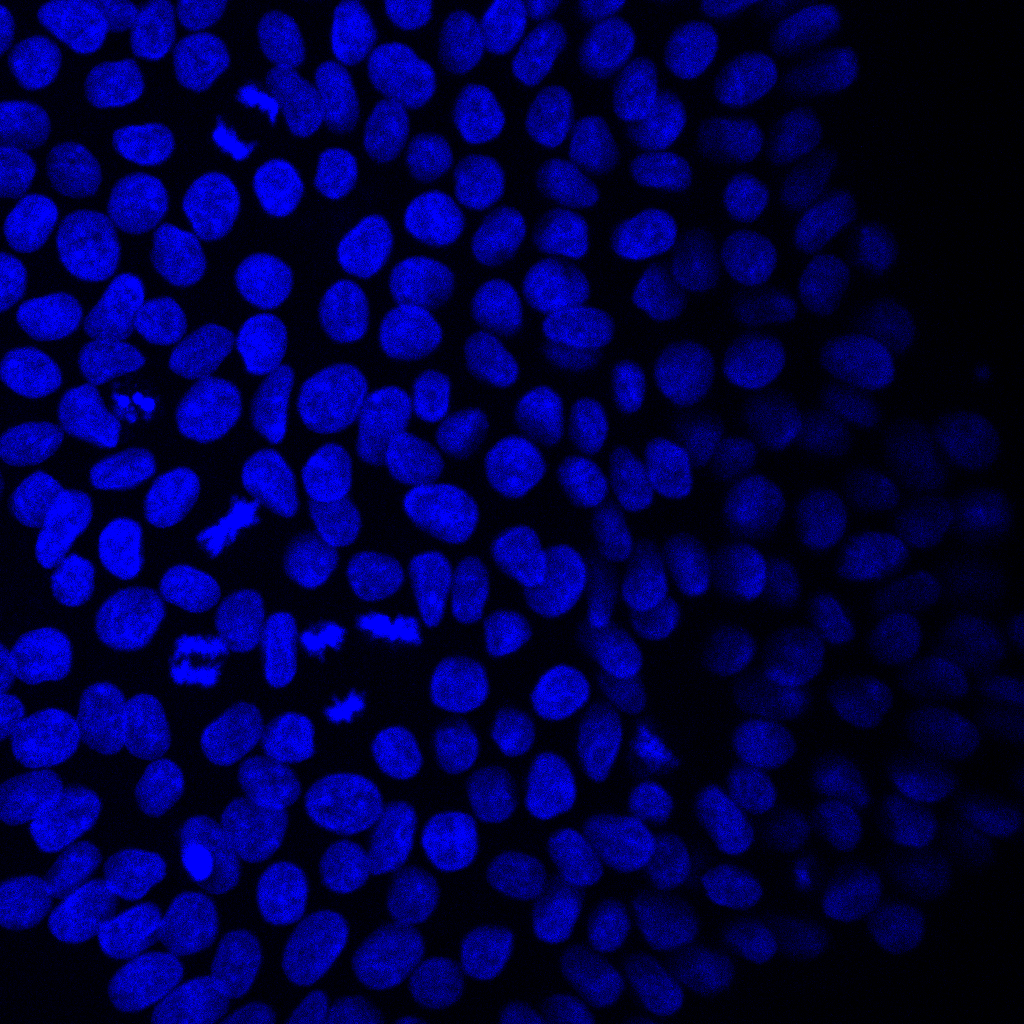

Supplement: Supplementary file 9 — Figure EV1 Source Data [file 44321_2025_302_MOESM9_ESM.zip › Figure EV1/EV1B/H9-DAPI.tif]

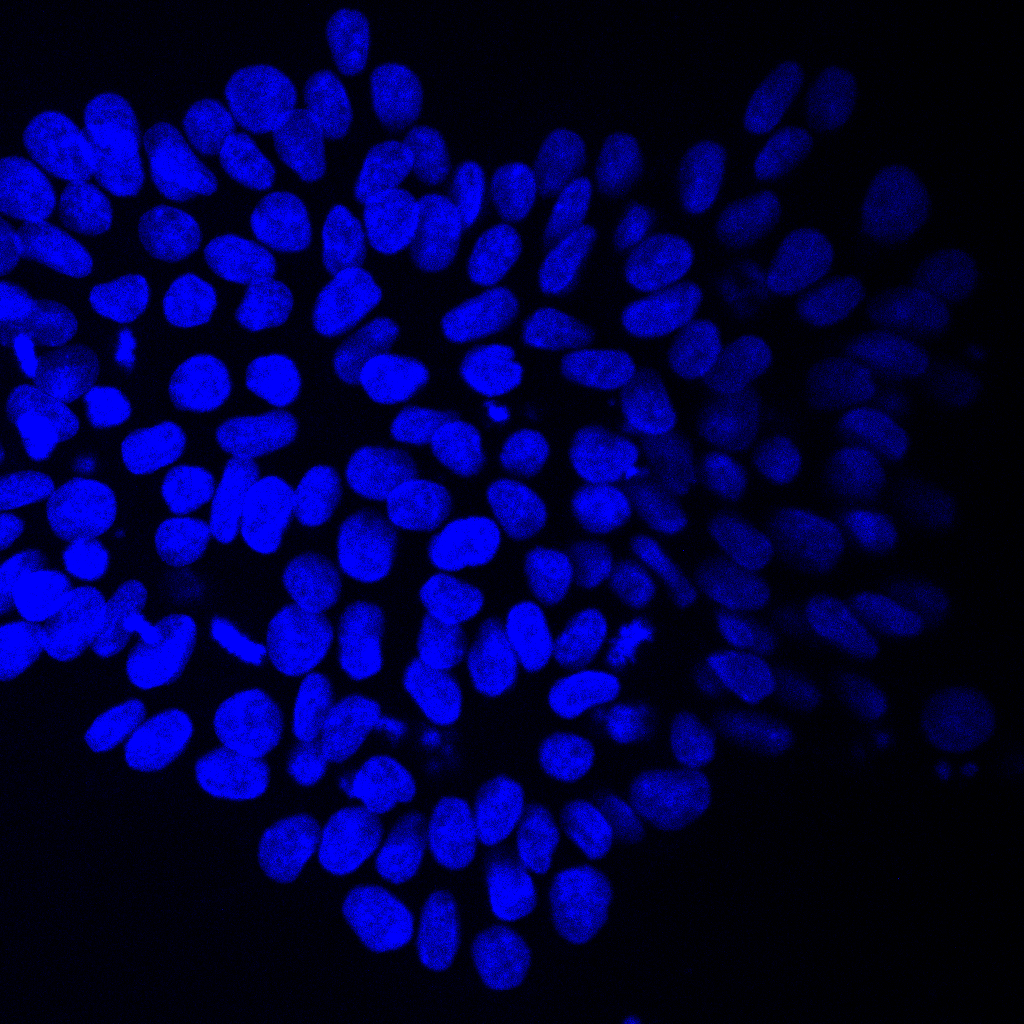

Supplement: Supplementary file 9 — Figure EV1 Source Data [file 44321_2025_302_MOESM9_ESM.zip › Figure EV1/EV1B/#12-3-DAPI.tif]

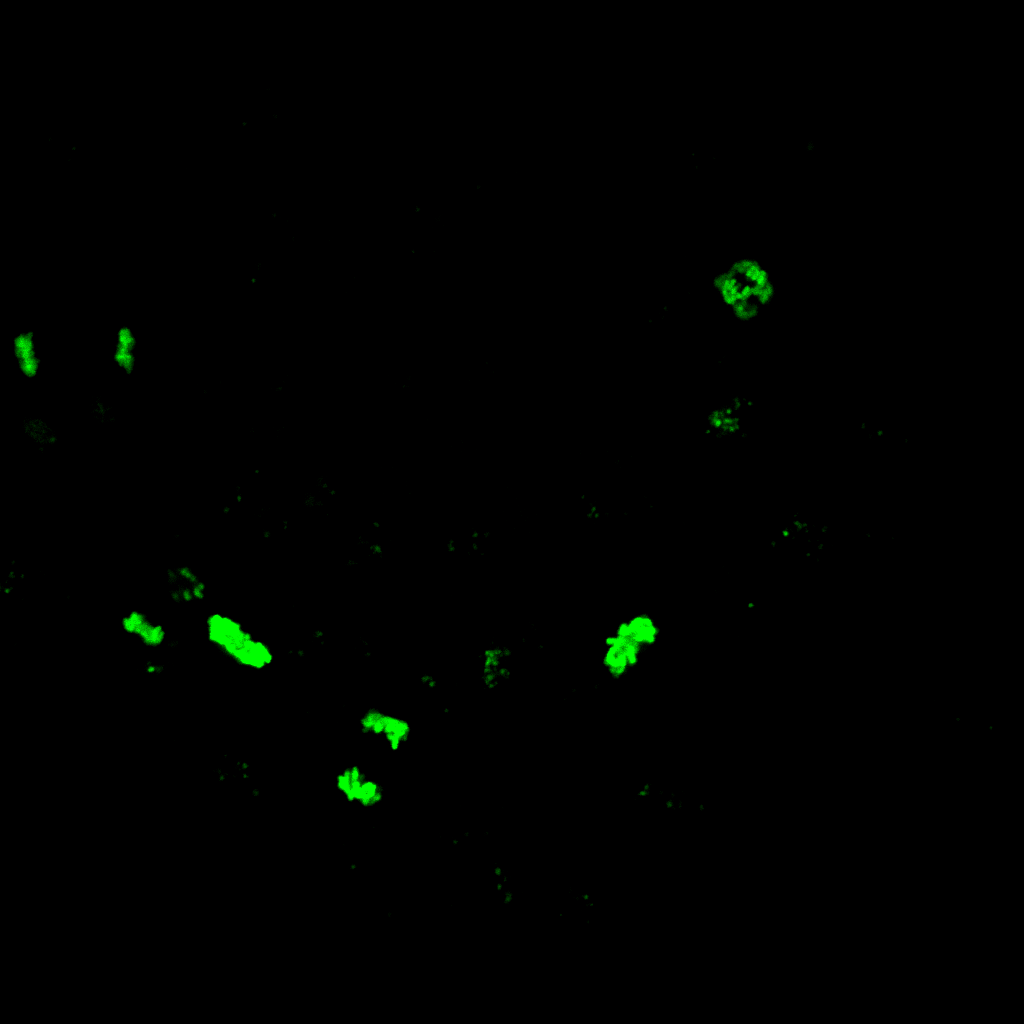

Supplement: Supplementary file 9 — Figure EV1 Source Data [file 44321_2025_302_MOESM9_ESM.zip › Figure EV1/EV1B/#12-3-PH3.tif]

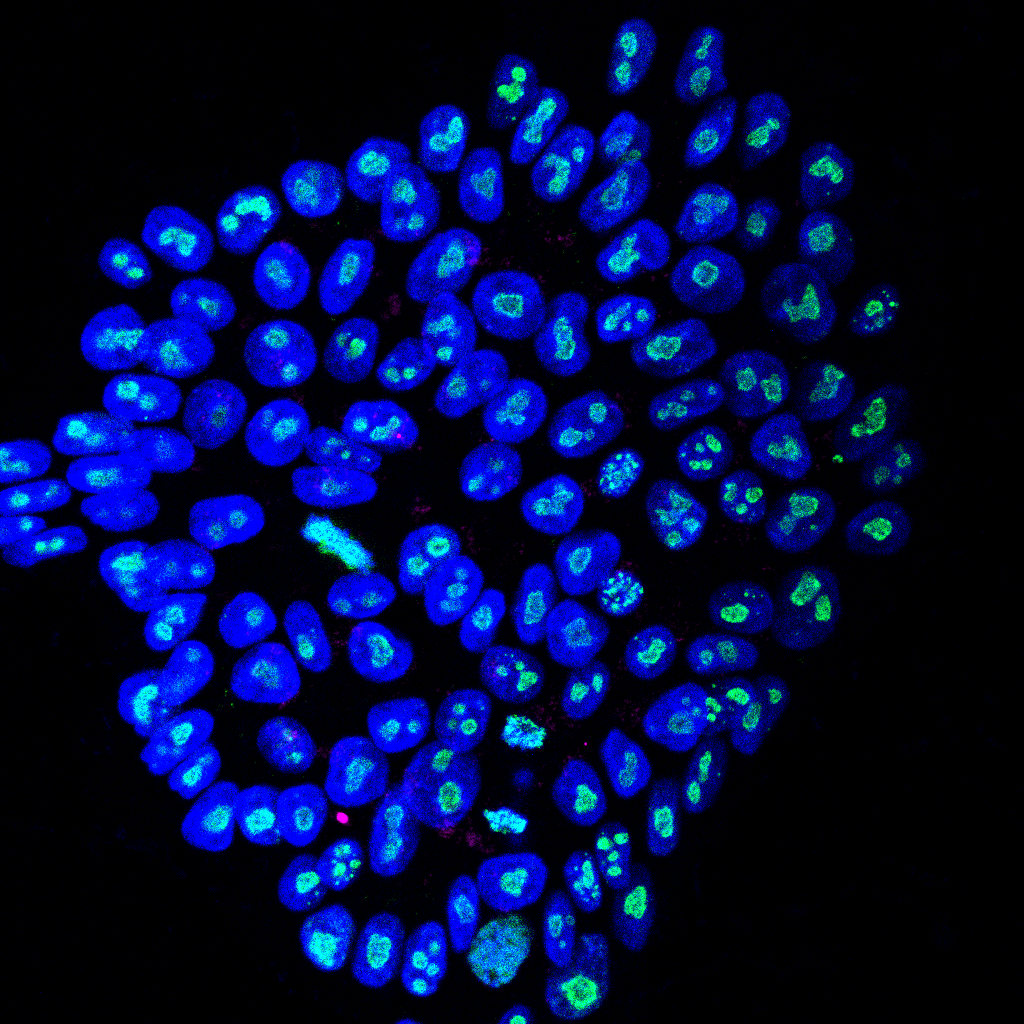

Supplement: Supplementary file 9 — Figure EV1 Source Data [file 44321_2025_302_MOESM9_ESM.zip › Figure EV1/EV1A/#12-3-merge.tif]

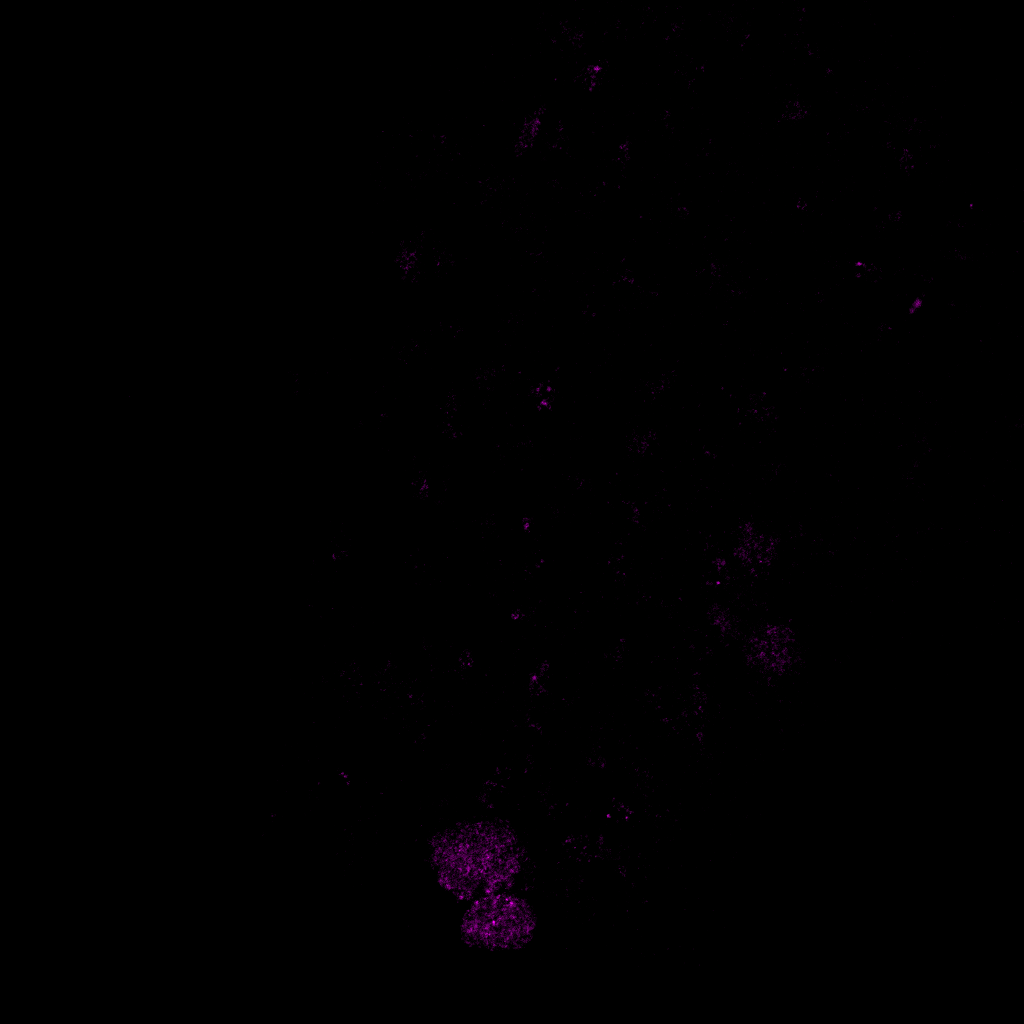

Supplement: Supplementary file 9 — Figure EV1 Source Data [file 44321_2025_302_MOESM9_ESM.zip › Figure EV1/EV1A/H9-Caspase3.tif]

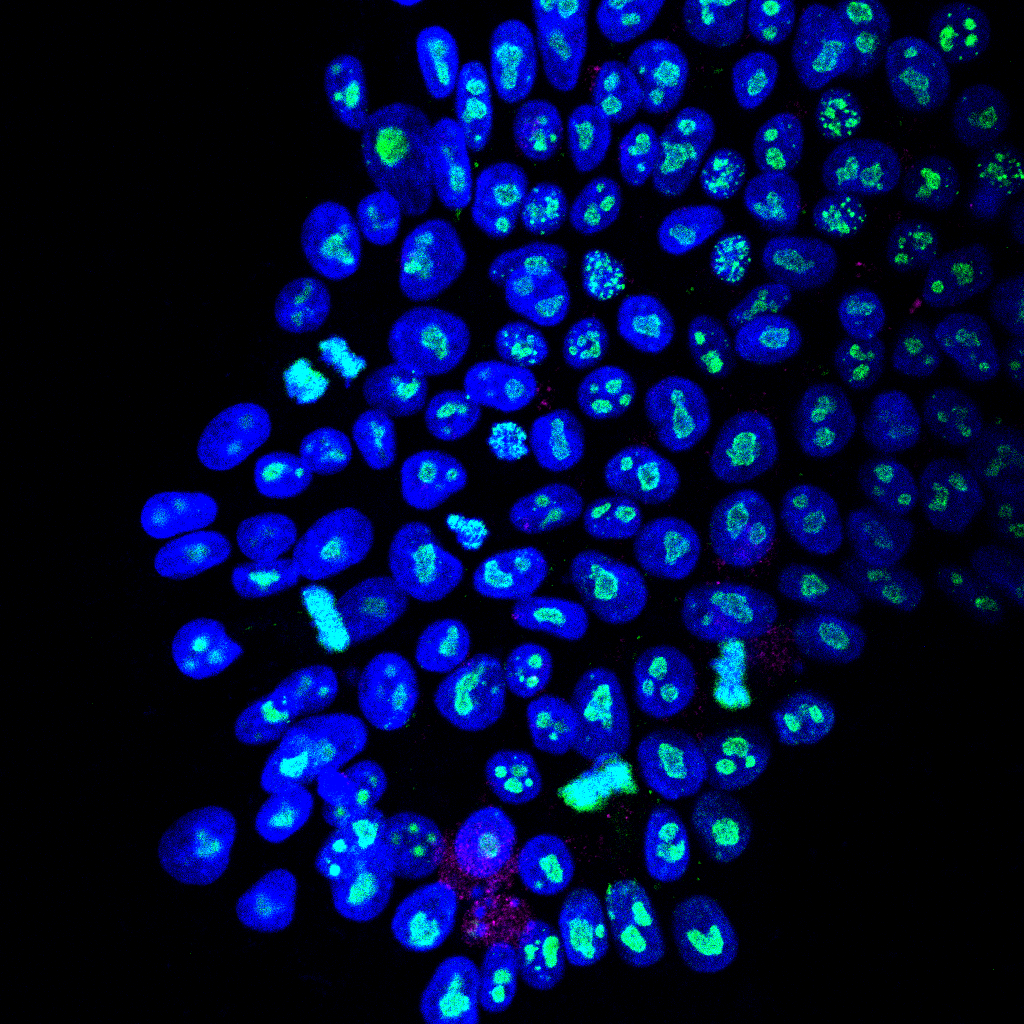

Supplement: Supplementary file 9 — Figure EV1 Source Data [file 44321_2025_302_MOESM9_ESM.zip › Figure EV1/EV1A/H9-merge.tif]

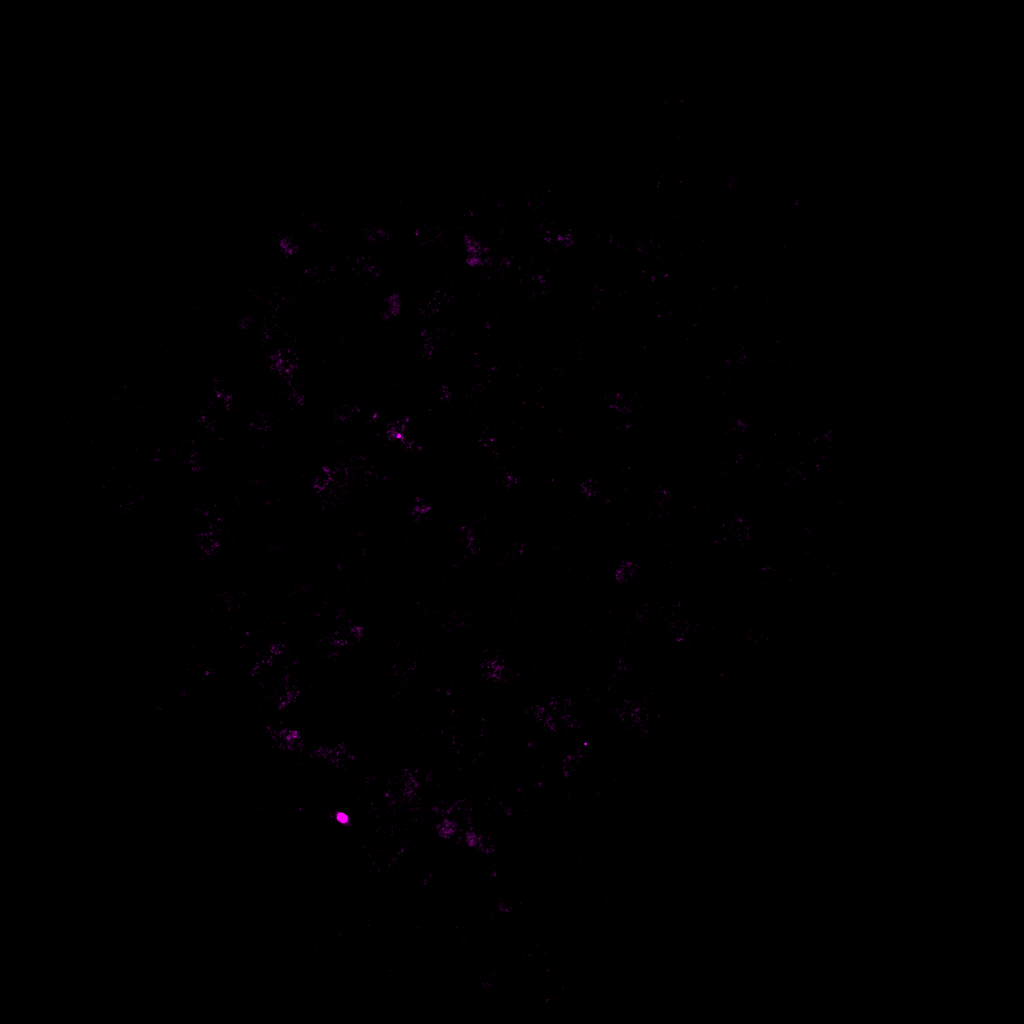

Supplement: Supplementary file 9 — Figure EV1 Source Data [file 44321_2025_302_MOESM9_ESM.zip › Figure EV1/EV1A/#12-3-Caspase3.tif]

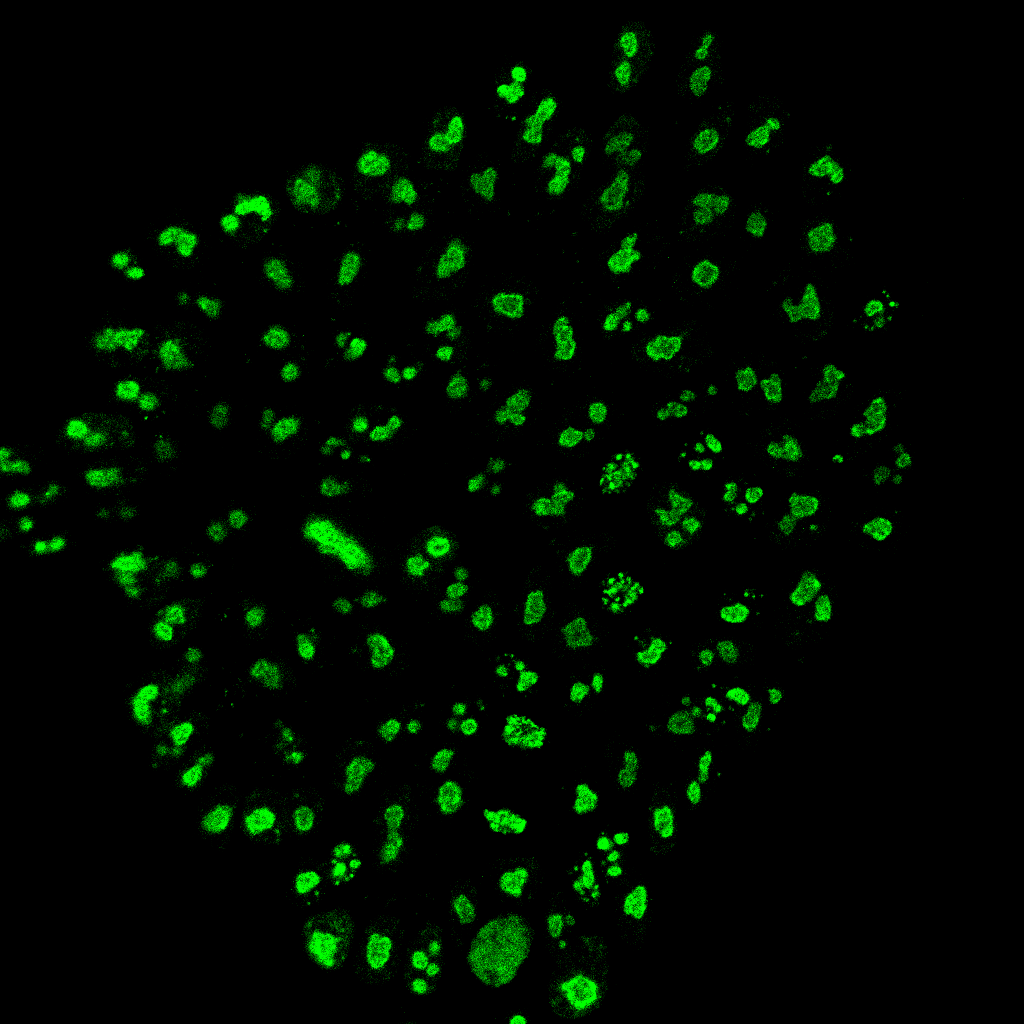

Supplement: Supplementary file 9 — Figure EV1 Source Data [file 44321_2025_302_MOESM9_ESM.zip › Figure EV1/EV1A/#12-3-Ki67.tif]

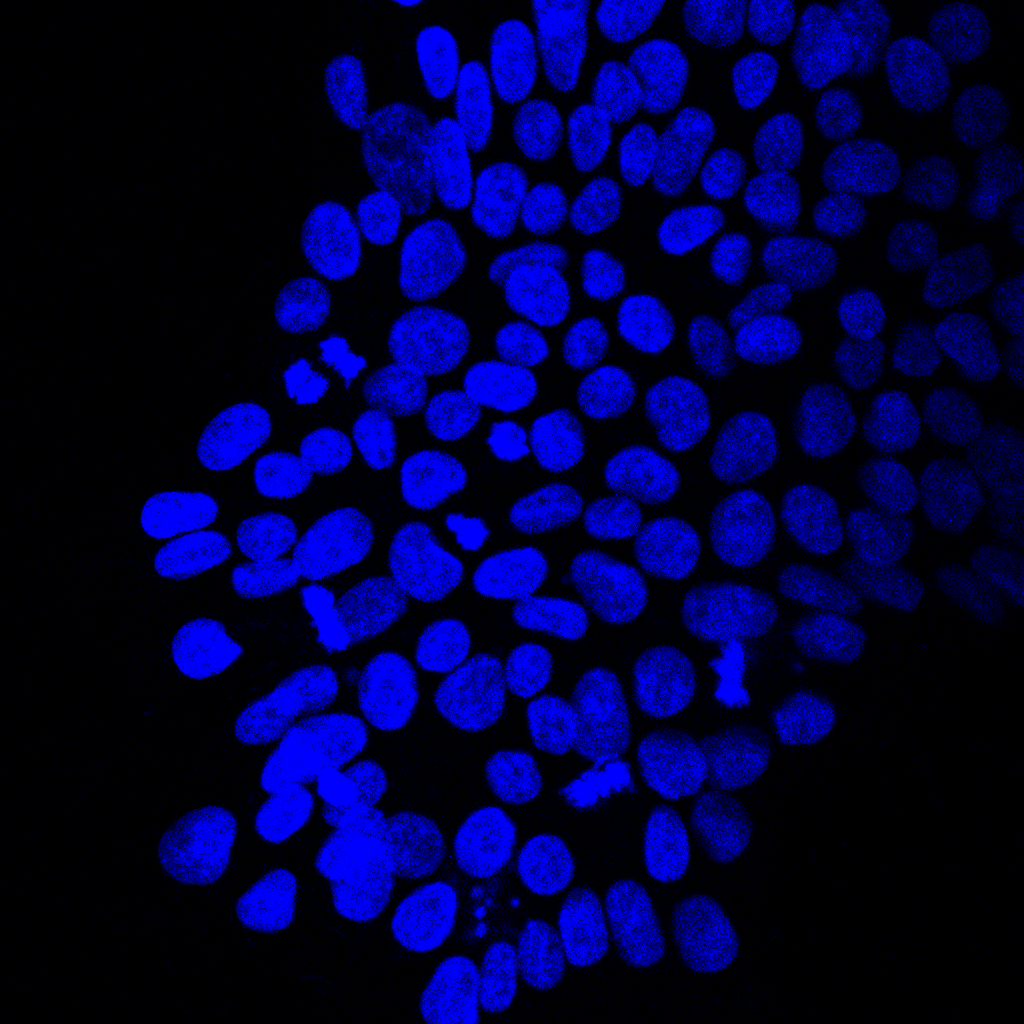

Supplement: Supplementary file 9 — Figure EV1 Source Data [file 44321_2025_302_MOESM9_ESM.zip › Figure EV1/EV1A/H9-DAPI.tif]

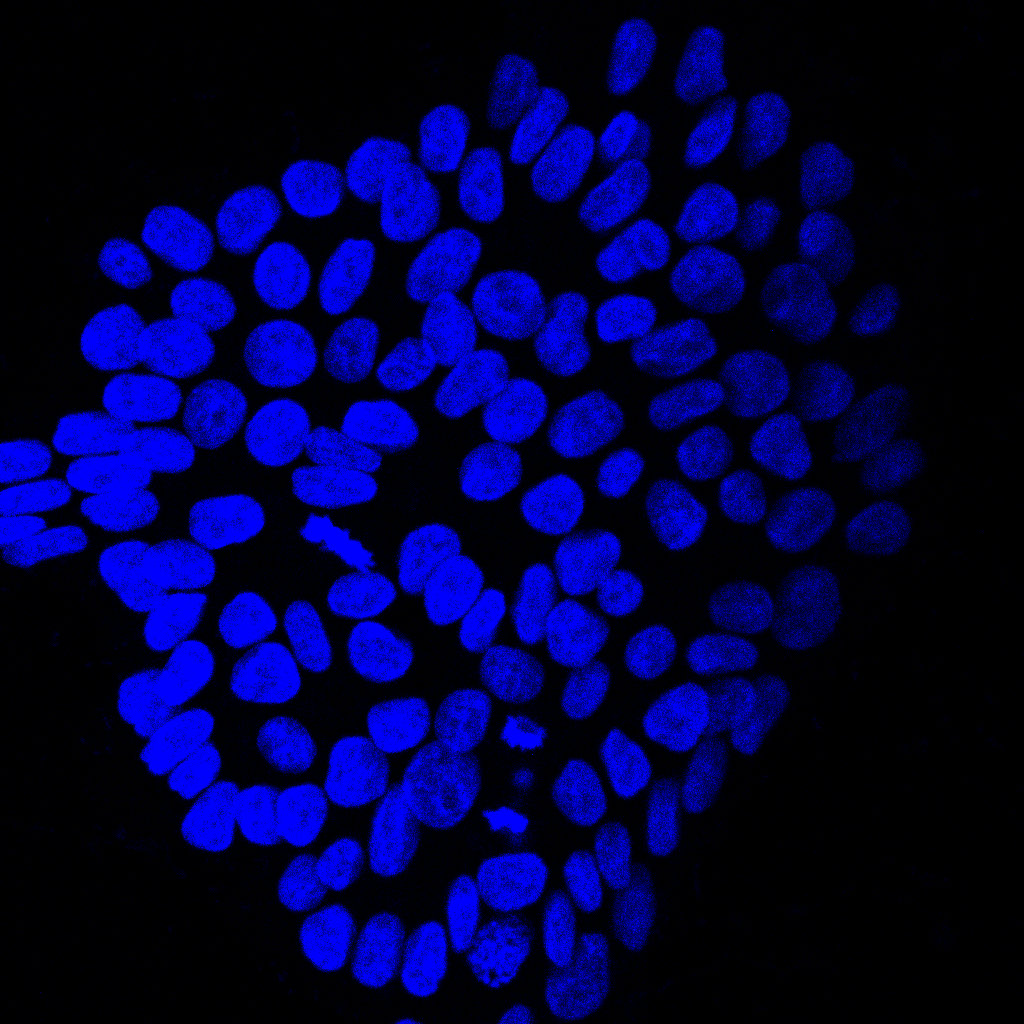

Supplement: Supplementary file 9 — Figure EV1 Source Data [file 44321_2025_302_MOESM9_ESM.zip › Figure EV1/EV1A/#12-3-DAPI.tif]

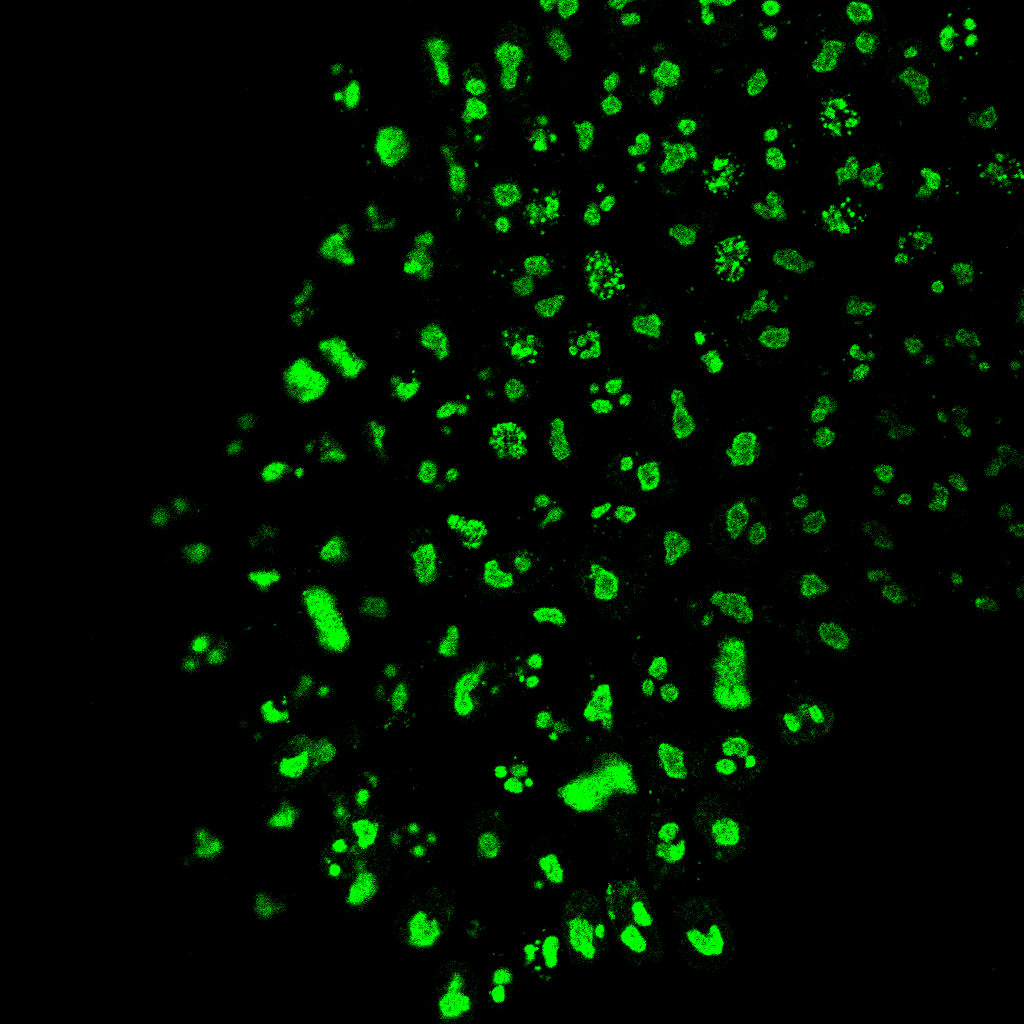

Supplement: Supplementary file 9 — Figure EV1 Source Data [file 44321_2025_302_MOESM9_ESM.zip › Figure EV1/EV1A/H9-Ki67.tif]

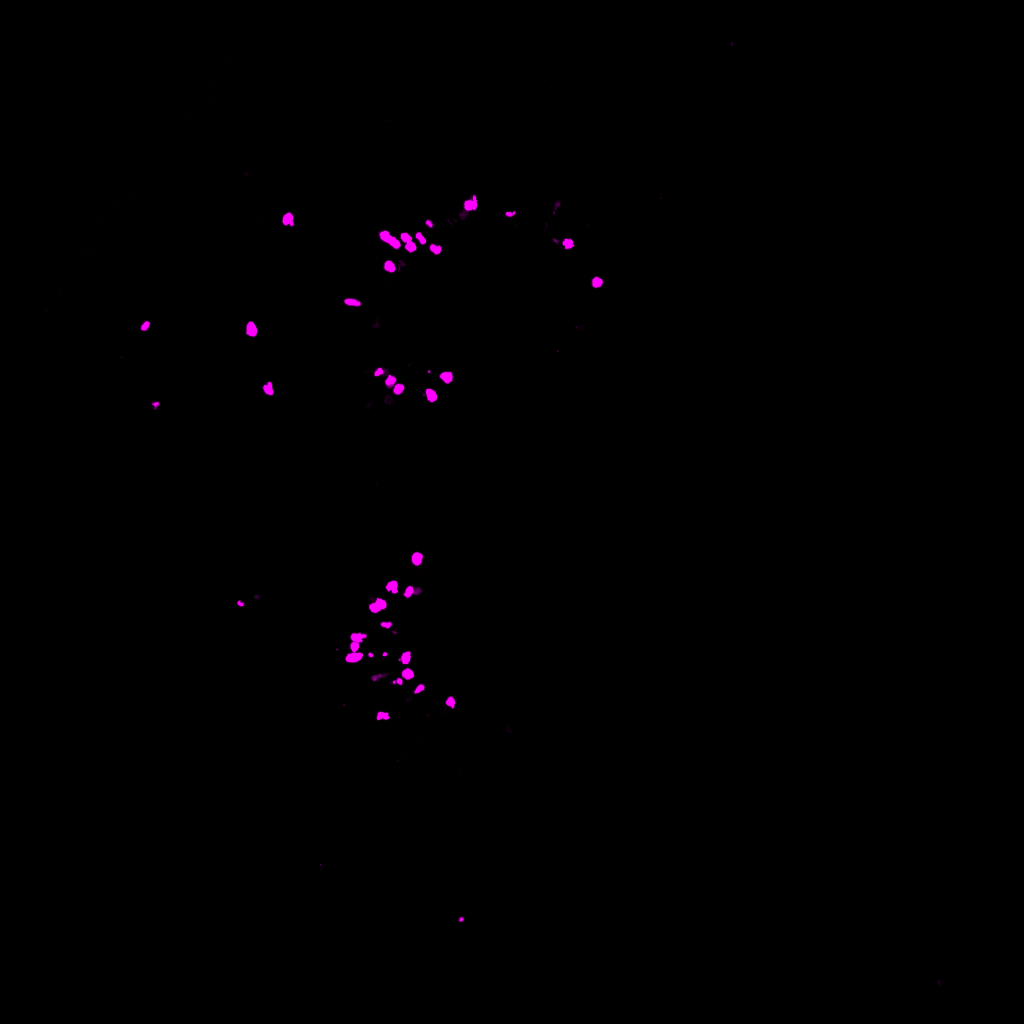

Supplement: Supplementary file 10 — Figure EV2 Source Data [file 44321_2025_302_MOESM10_ESM.zip › Figure EV2/EV2E/#15-4-PH3.tif]

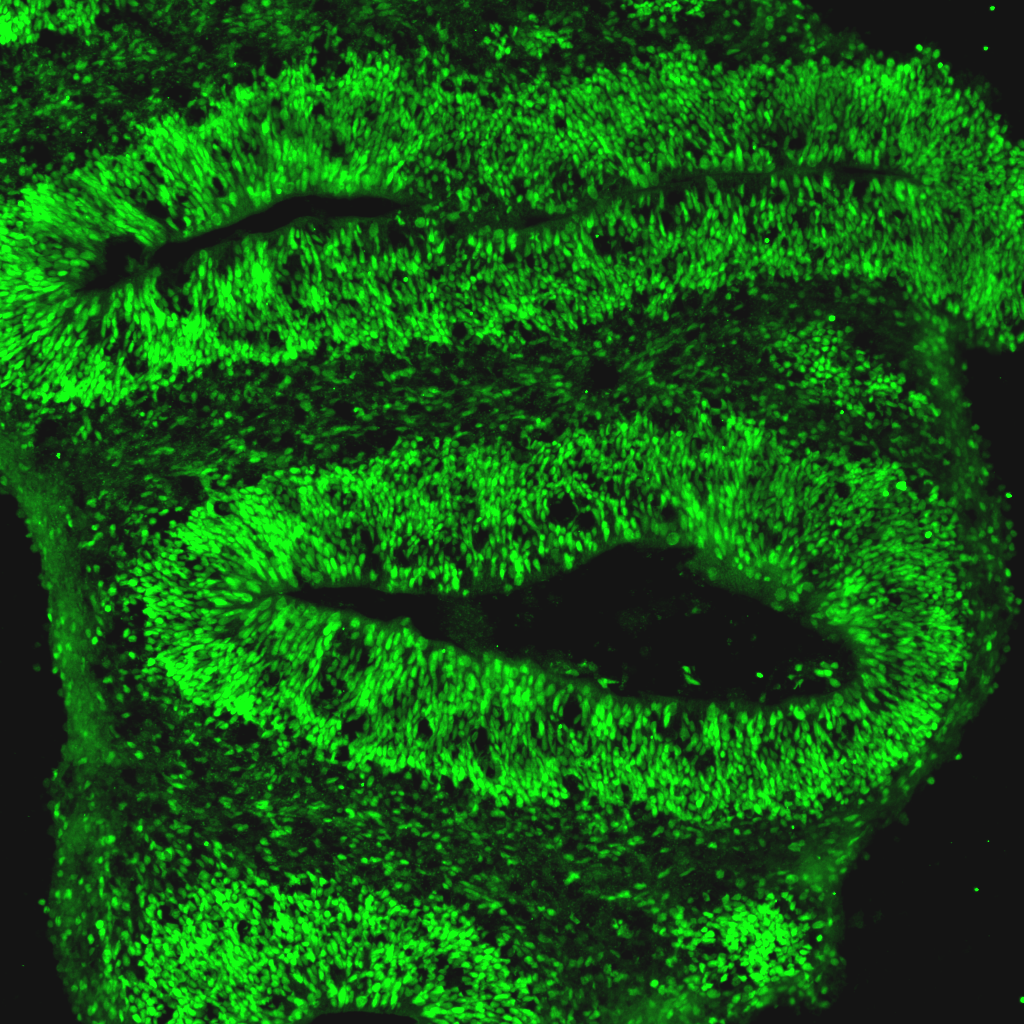

Supplement: Supplementary file 10 — Figure EV2 Source Data [file 44321_2025_302_MOESM10_ESM.zip › Figure EV2/EV2E/H1-PAX6.tif]

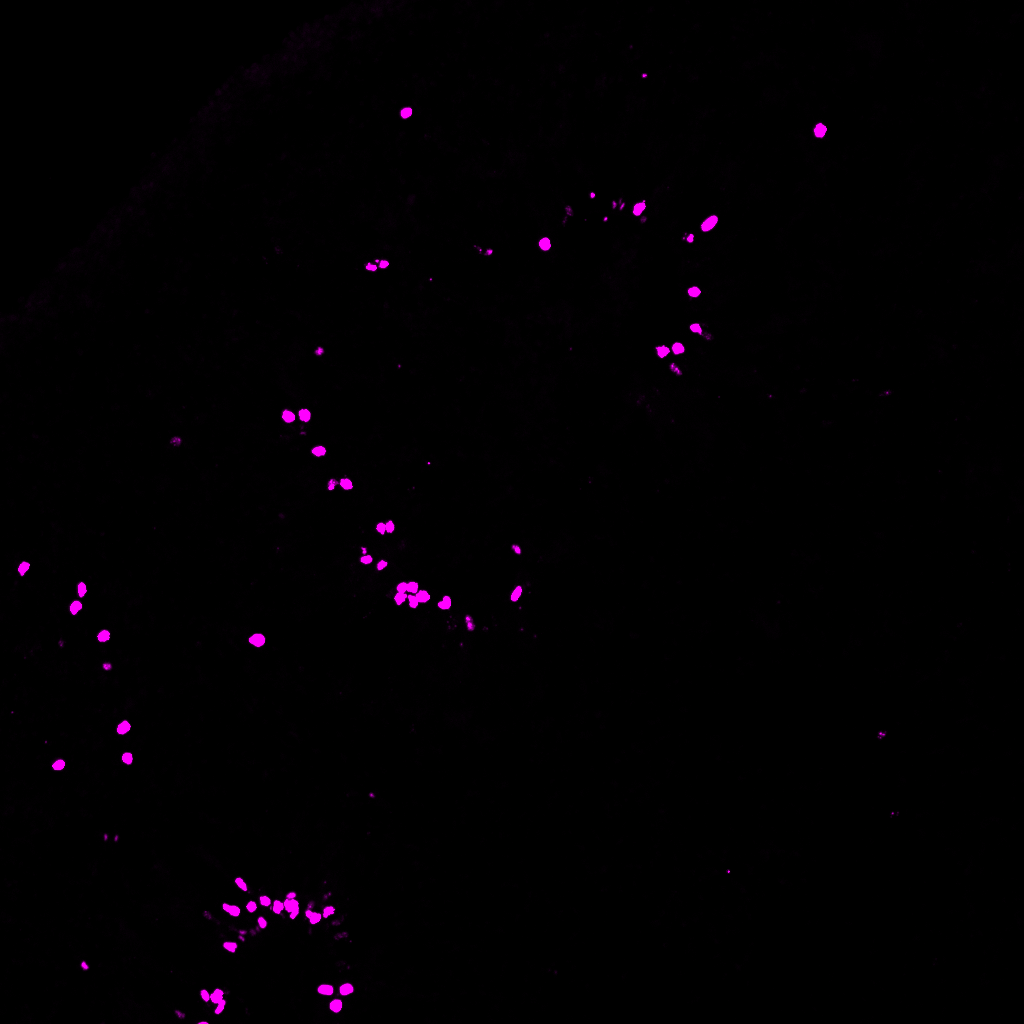

Supplement: Supplementary file 10 — Figure EV2 Source Data [file 44321_2025_302_MOESM10_ESM.zip › Figure EV2/EV2E/#6-6-PH3.tif]

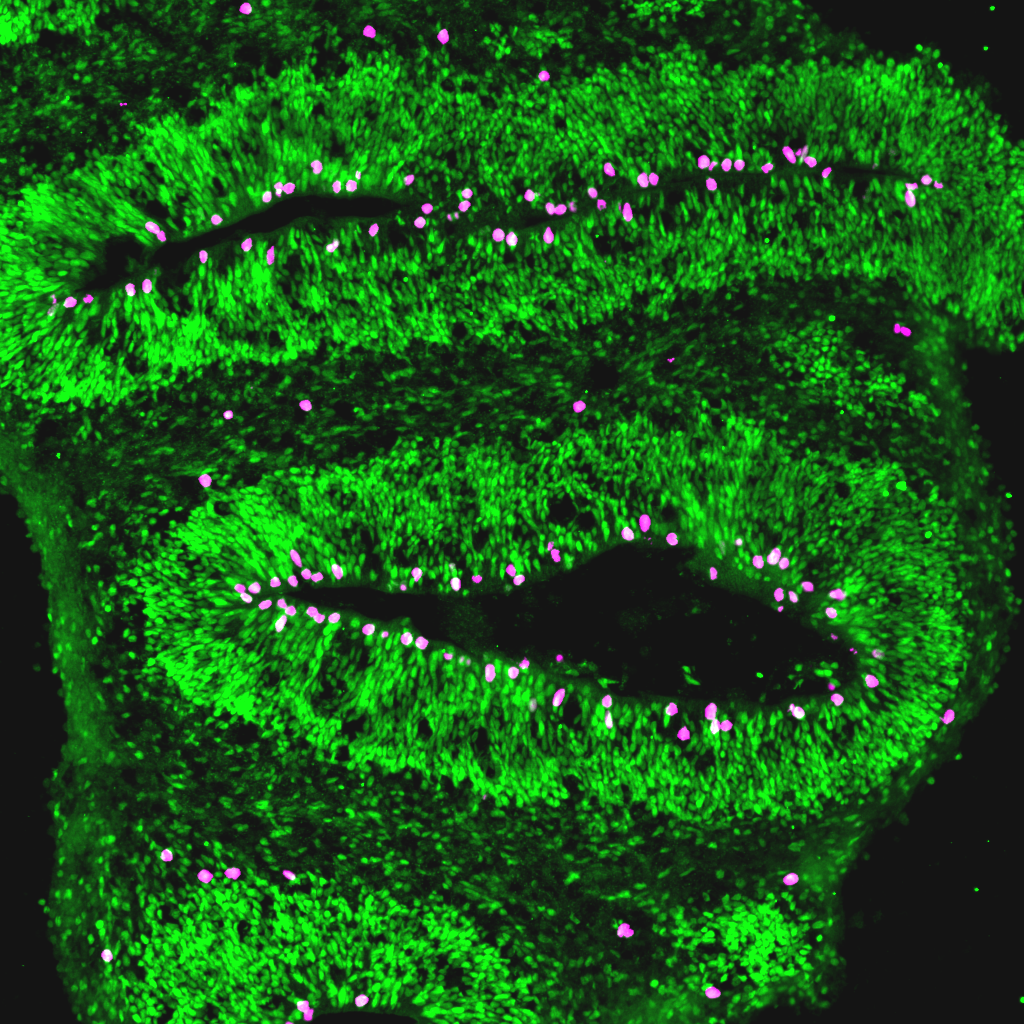

Supplement: Supplementary file 10 — Figure EV2 Source Data [file 44321_2025_302_MOESM10_ESM.zip › Figure EV2/EV2E/H1-merge.tif]

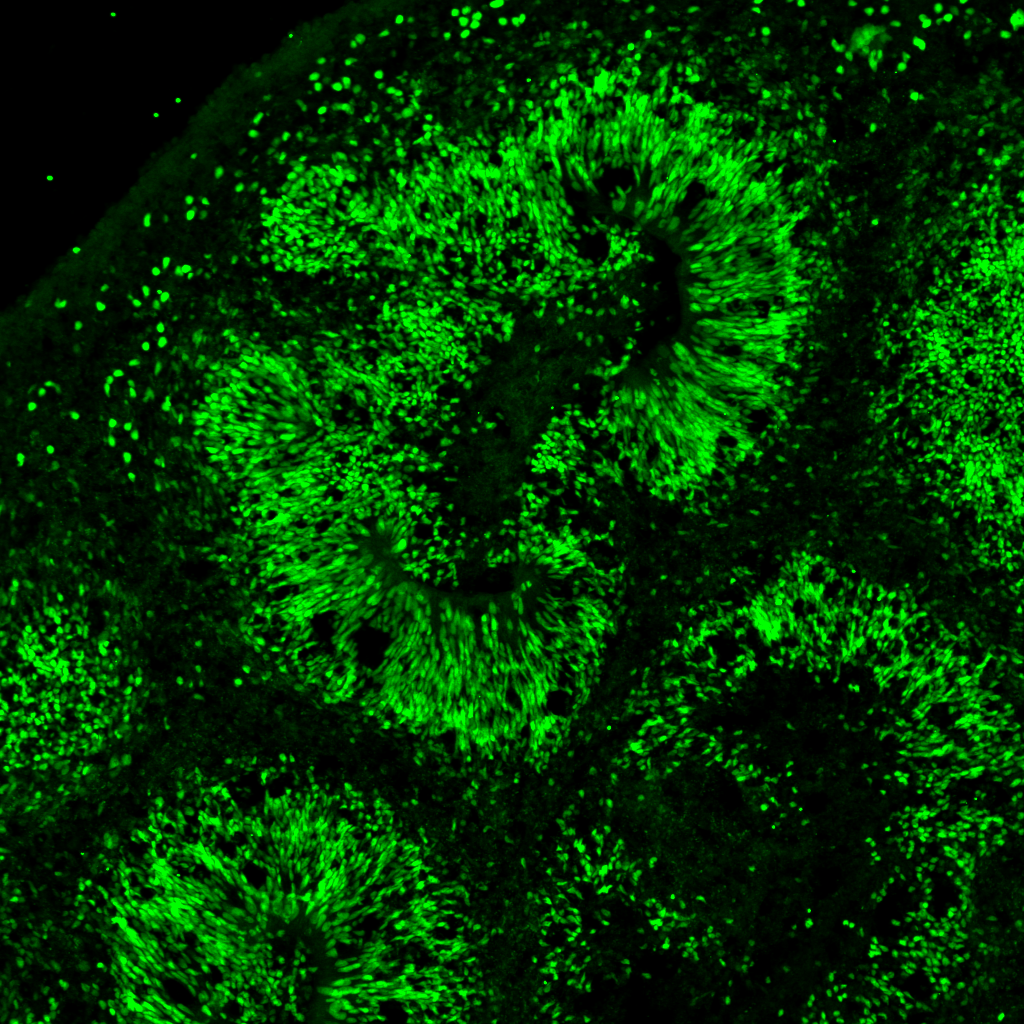

Supplement: Supplementary file 10 — Figure EV2 Source Data [file 44321_2025_302_MOESM10_ESM.zip › Figure EV2/EV2E/#6-6-PAX6.tif]

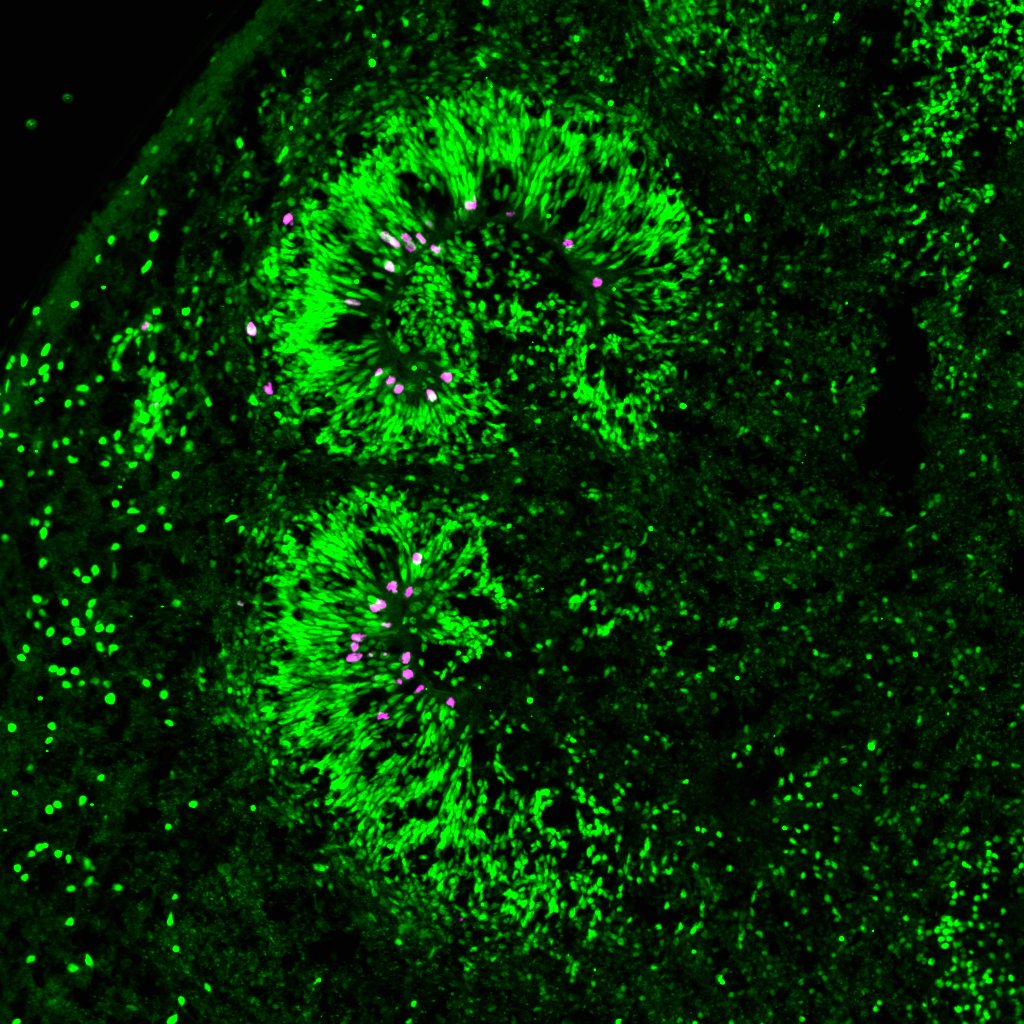

Supplement: Supplementary file 10 — Figure EV2 Source Data [file 44321_2025_302_MOESM10_ESM.zip › Figure EV2/EV2E/#15-4-merge.tif]

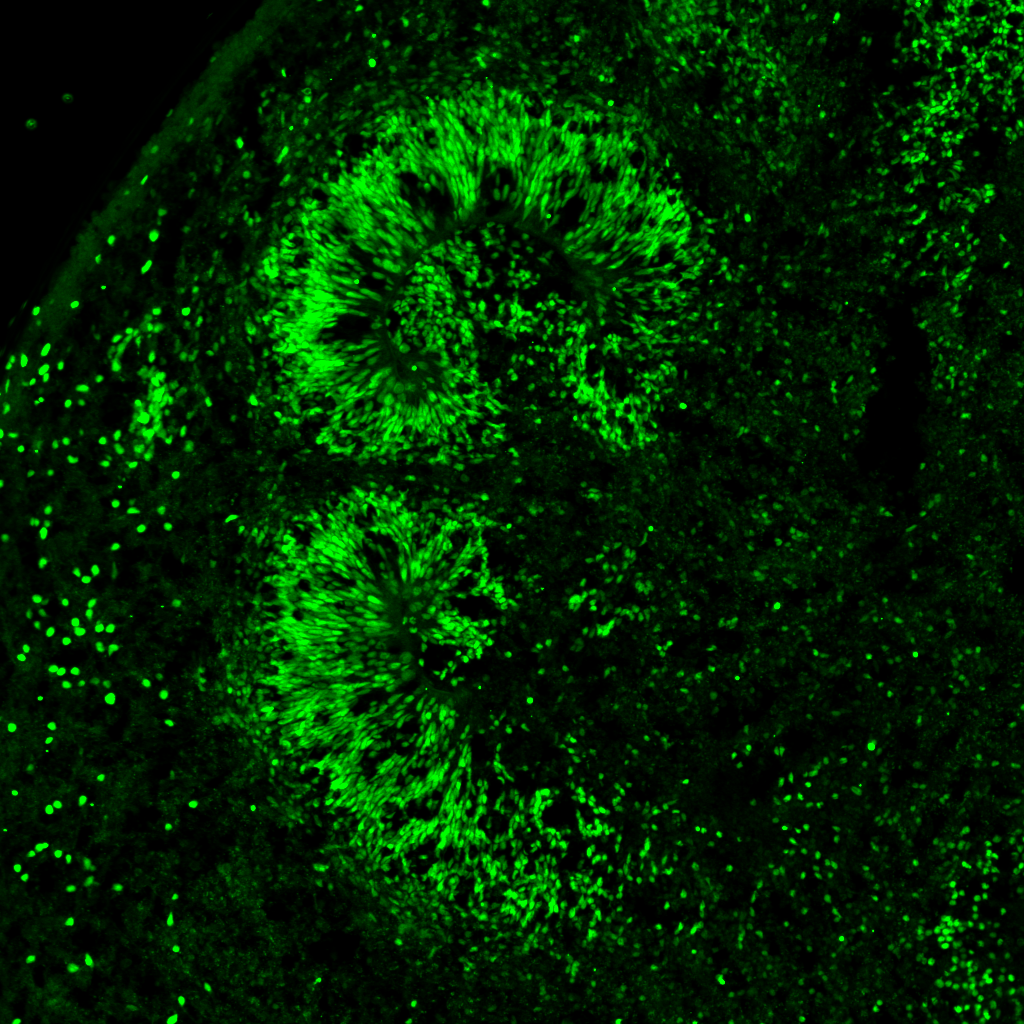

Supplement: Supplementary file 10 — Figure EV2 Source Data [file 44321_2025_302_MOESM10_ESM.zip › Figure EV2/EV2E/#15-4-PAX6.tif]

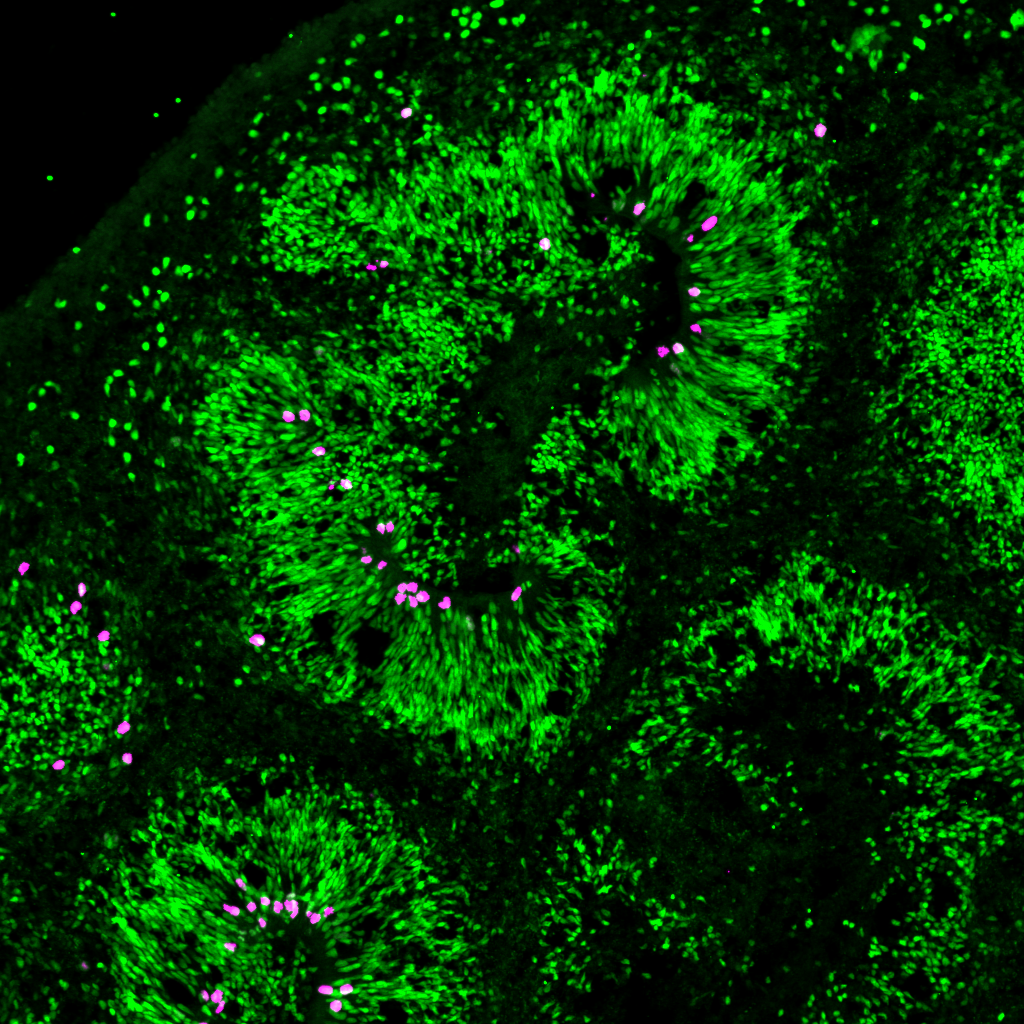

Supplement: Supplementary file 10 — Figure EV2 Source Data [file 44321_2025_302_MOESM10_ESM.zip › Figure EV2/EV2E/#6-6-merge.tif]

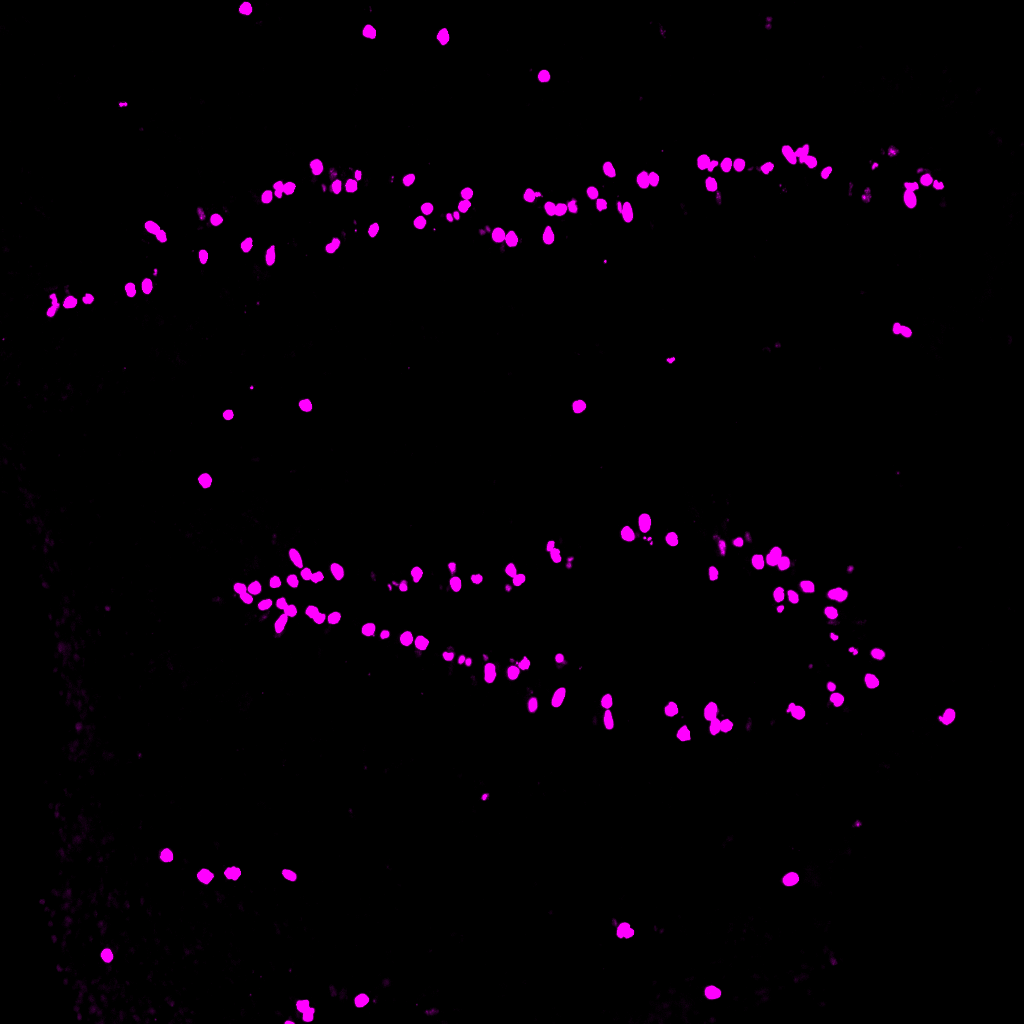

Supplement: Supplementary file 10 — Figure EV2 Source Data [file 44321_2025_302_MOESM10_ESM.zip › Figure EV2/EV2E/H1-PH3.tif]

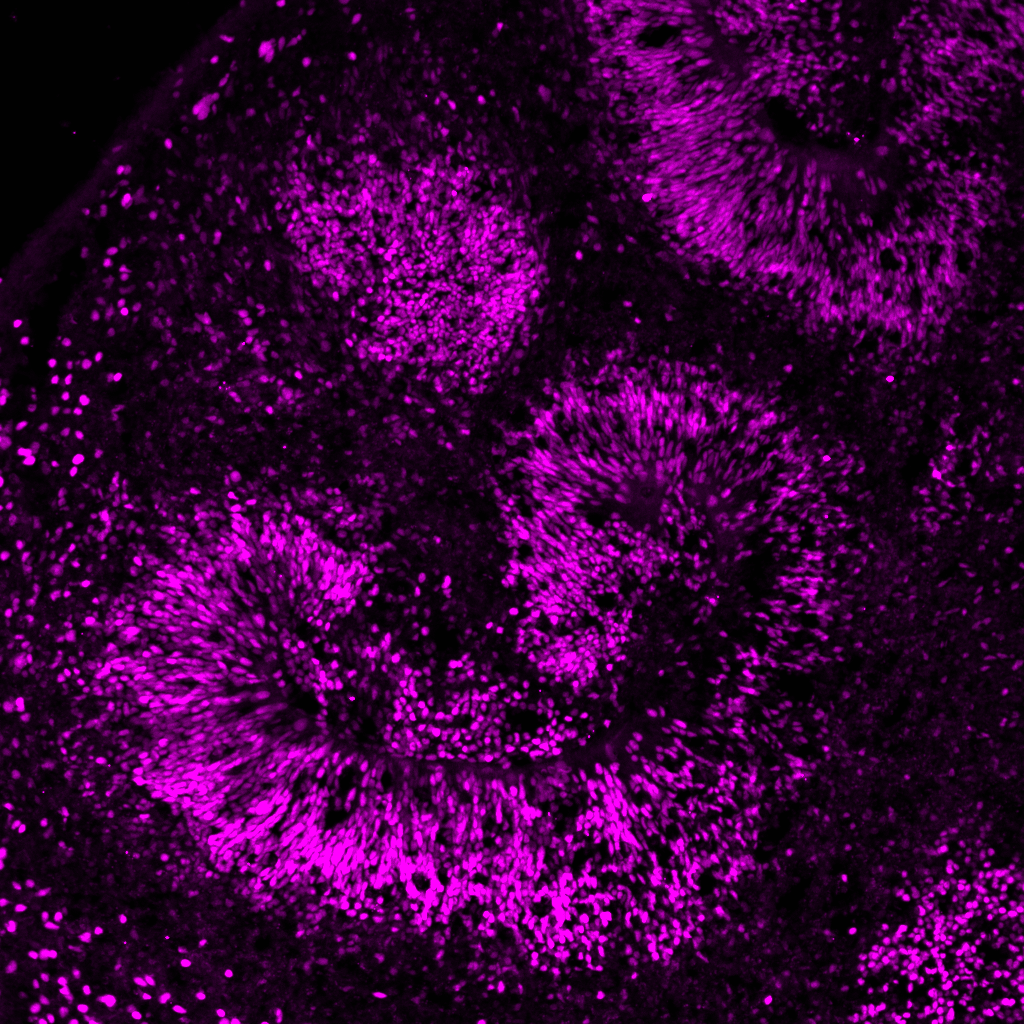

Supplement: Supplementary file 10 — Figure EV2 Source Data [file 44321_2025_302_MOESM10_ESM.zip › Figure EV2/EV2D/#6-6_PAX6.tif]

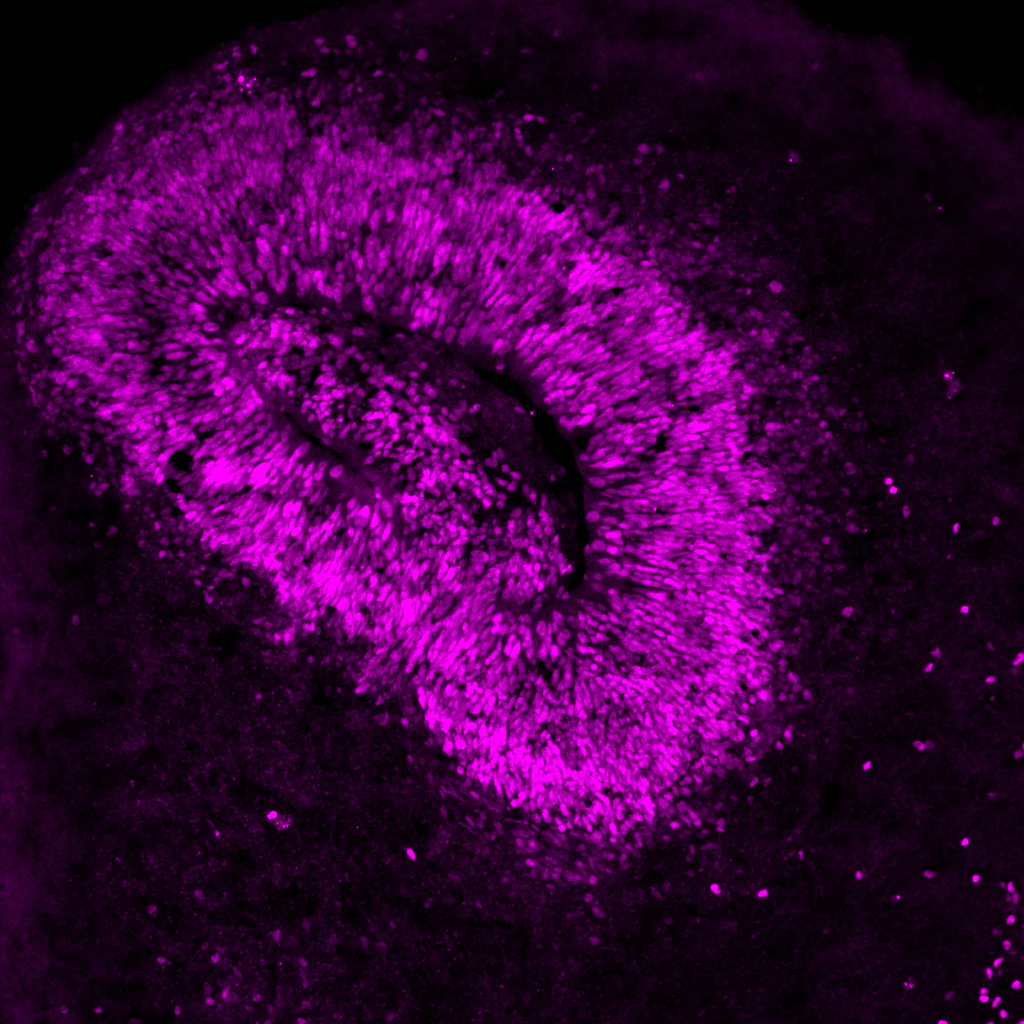

Supplement: Supplementary file 10 — Figure EV2 Source Data [file 44321_2025_302_MOESM10_ESM.zip › Figure EV2/EV2D/#15-4_PAX6.tif]

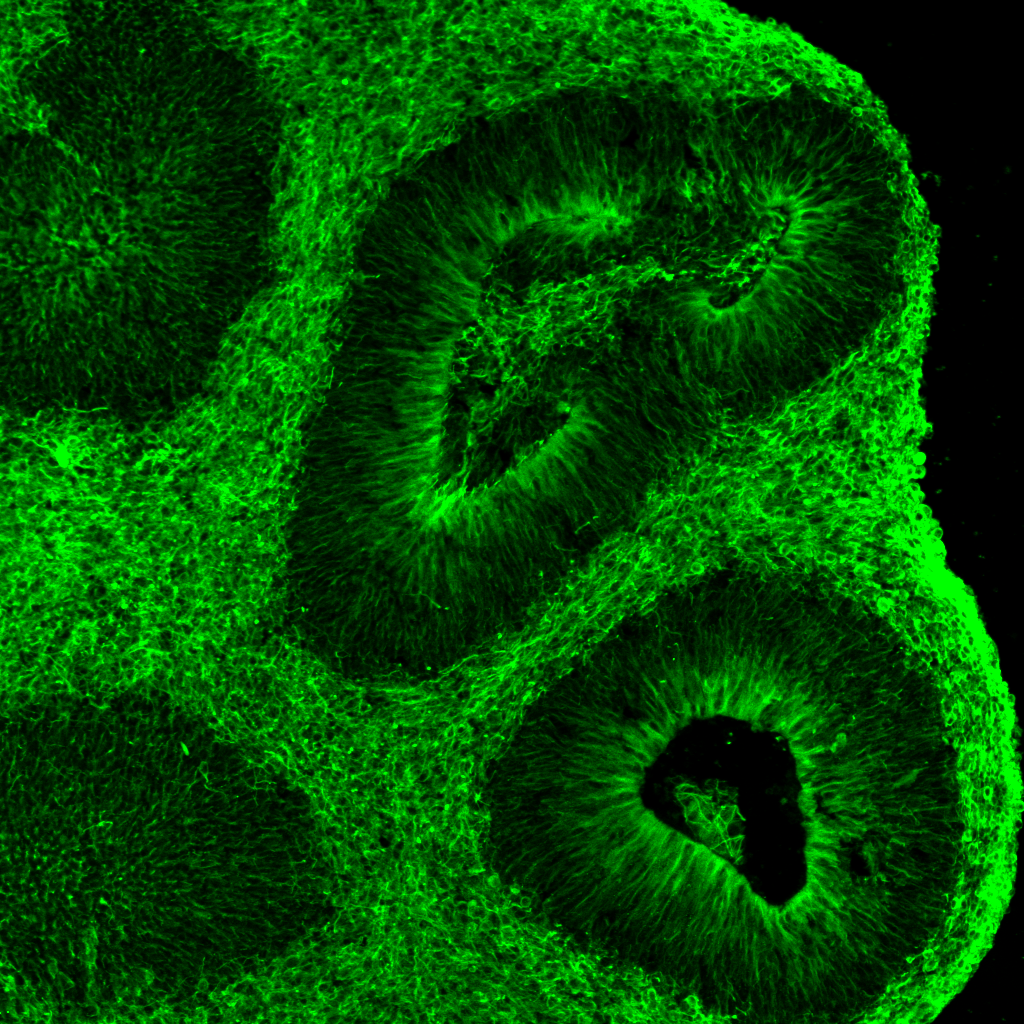

Supplement: Supplementary file 10 — Figure EV2 Source Data [file 44321_2025_302_MOESM10_ESM.zip › Figure EV2/EV2D/H1_TUJ1.tif]

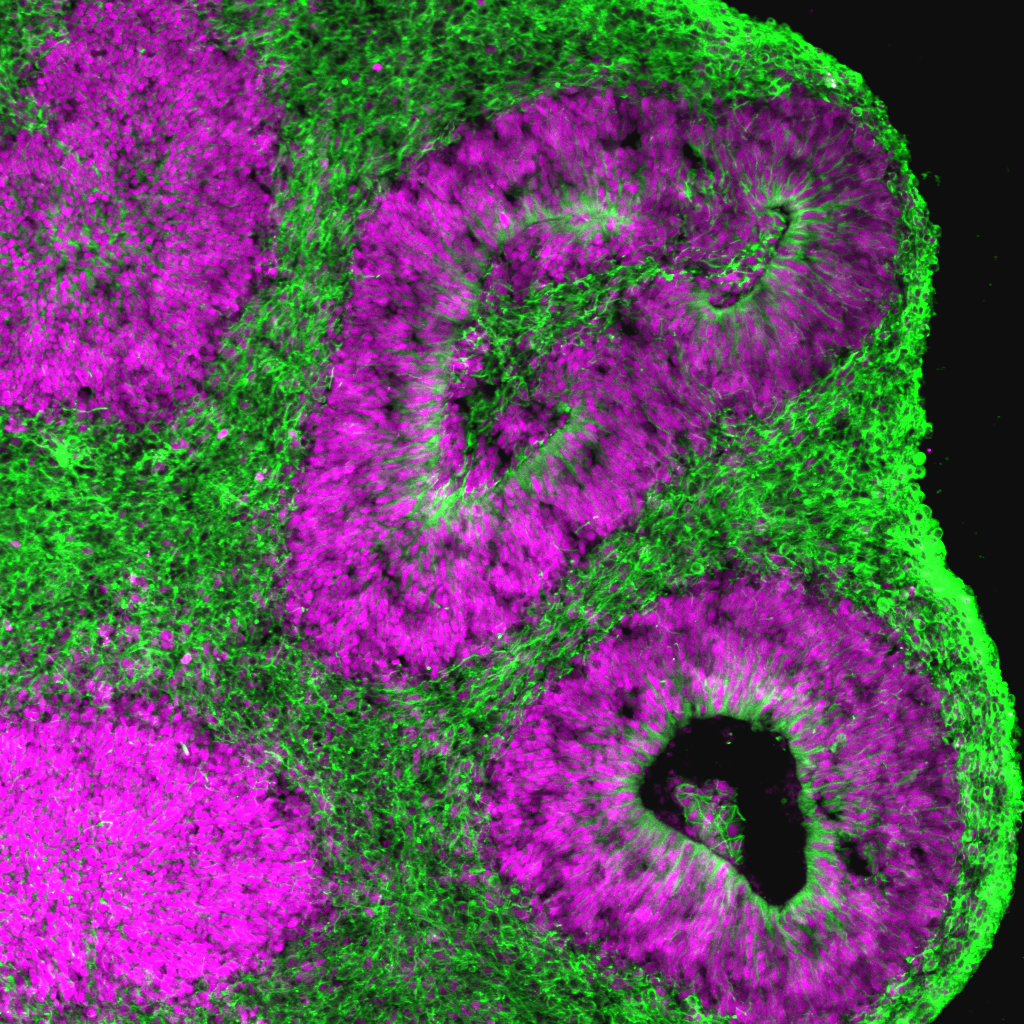

Supplement: Supplementary file 10 — Figure EV2 Source Data [file 44321_2025_302_MOESM10_ESM.zip › Figure EV2/EV2D/H1_merge.tif]

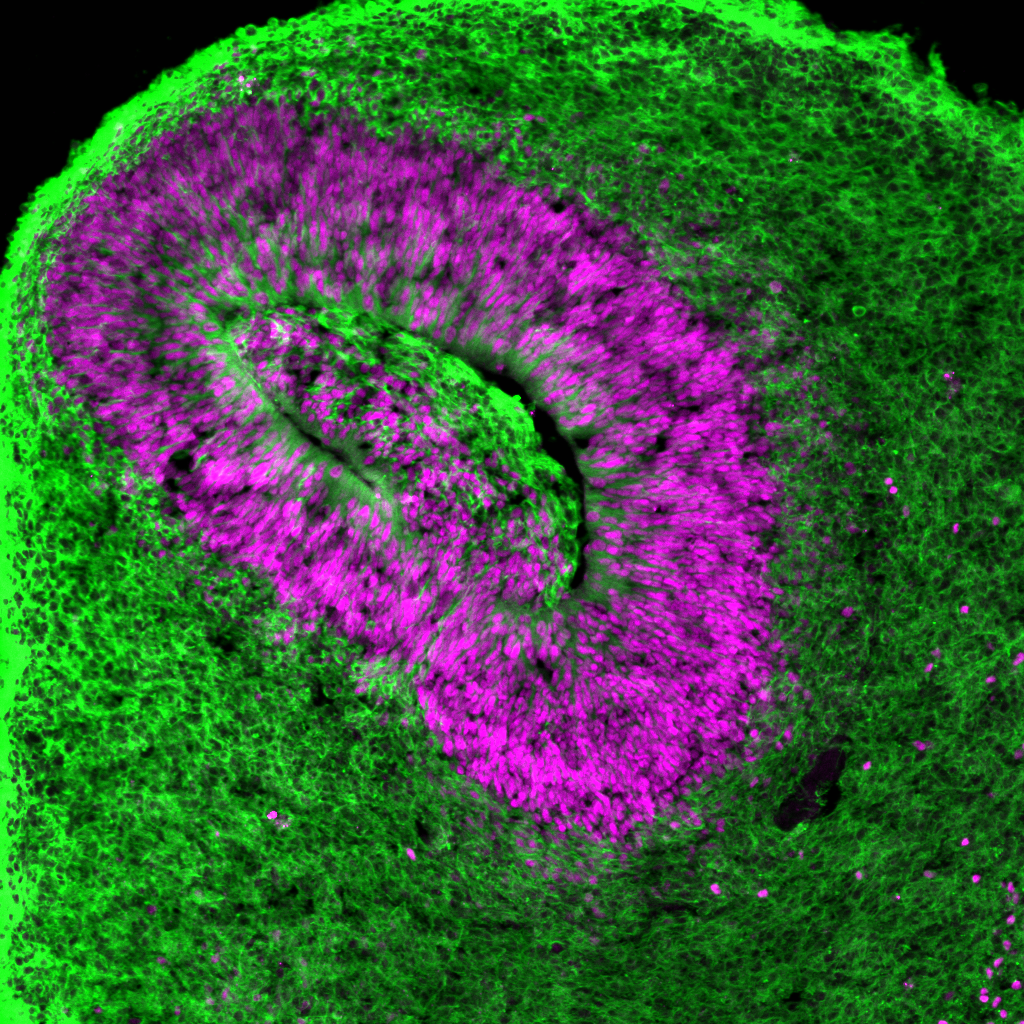

Supplement: Supplementary file 10 — Figure EV2 Source Data [file 44321_2025_302_MOESM10_ESM.zip › Figure EV2/EV2D/#15-4_merge.tif]

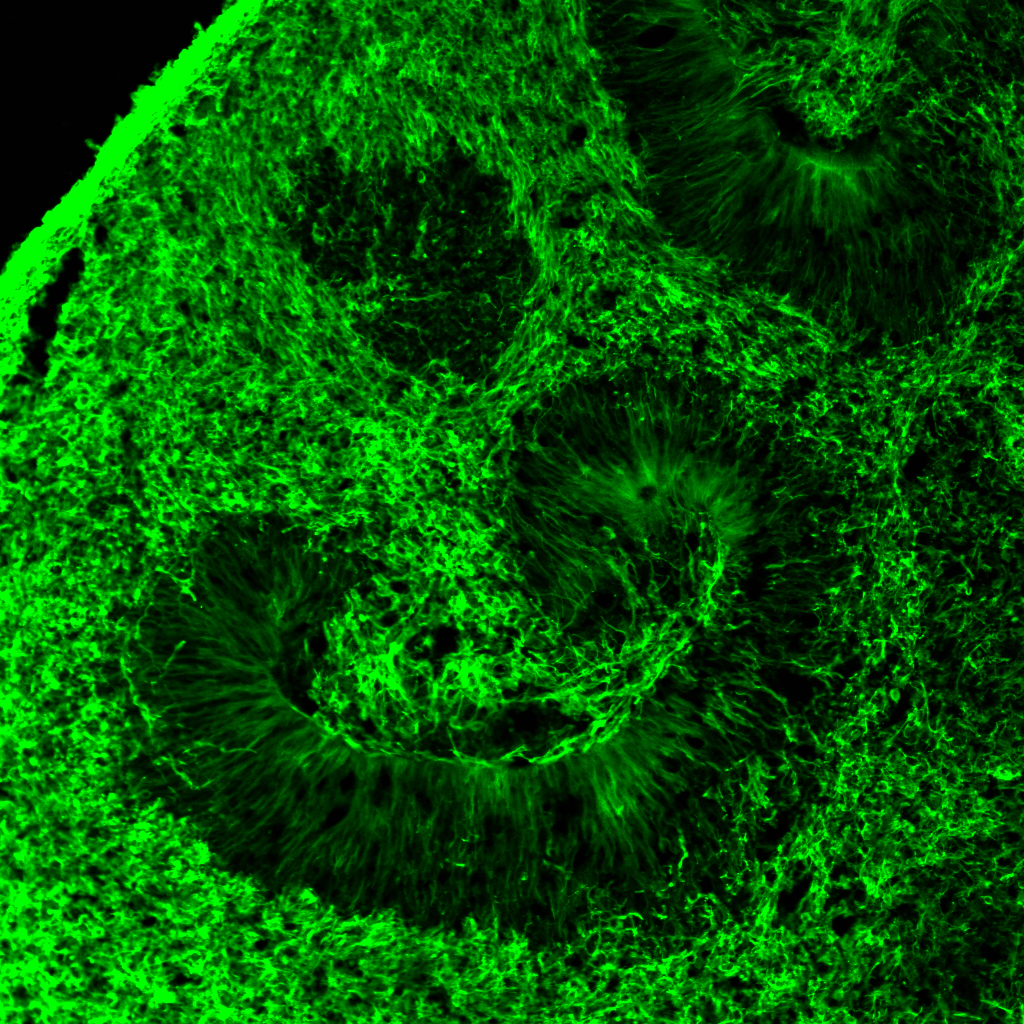

Supplement: Supplementary file 10 — Figure EV2 Source Data [file 44321_2025_302_MOESM10_ESM.zip › Figure EV2/EV2D/#6-6_TUJ1.tif]

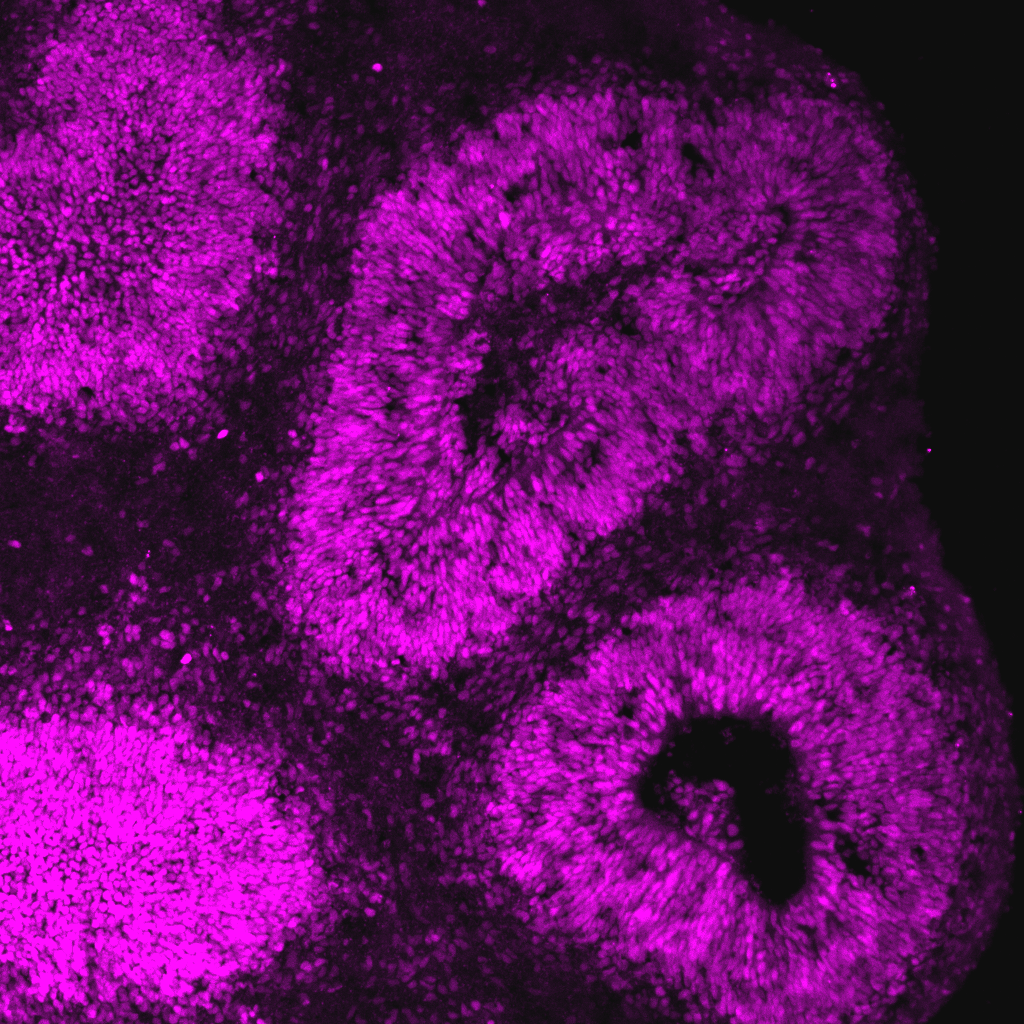

Supplement: Supplementary file 10 — Figure EV2 Source Data [file 44321_2025_302_MOESM10_ESM.zip › Figure EV2/EV2D/H1_PAX6.tif]

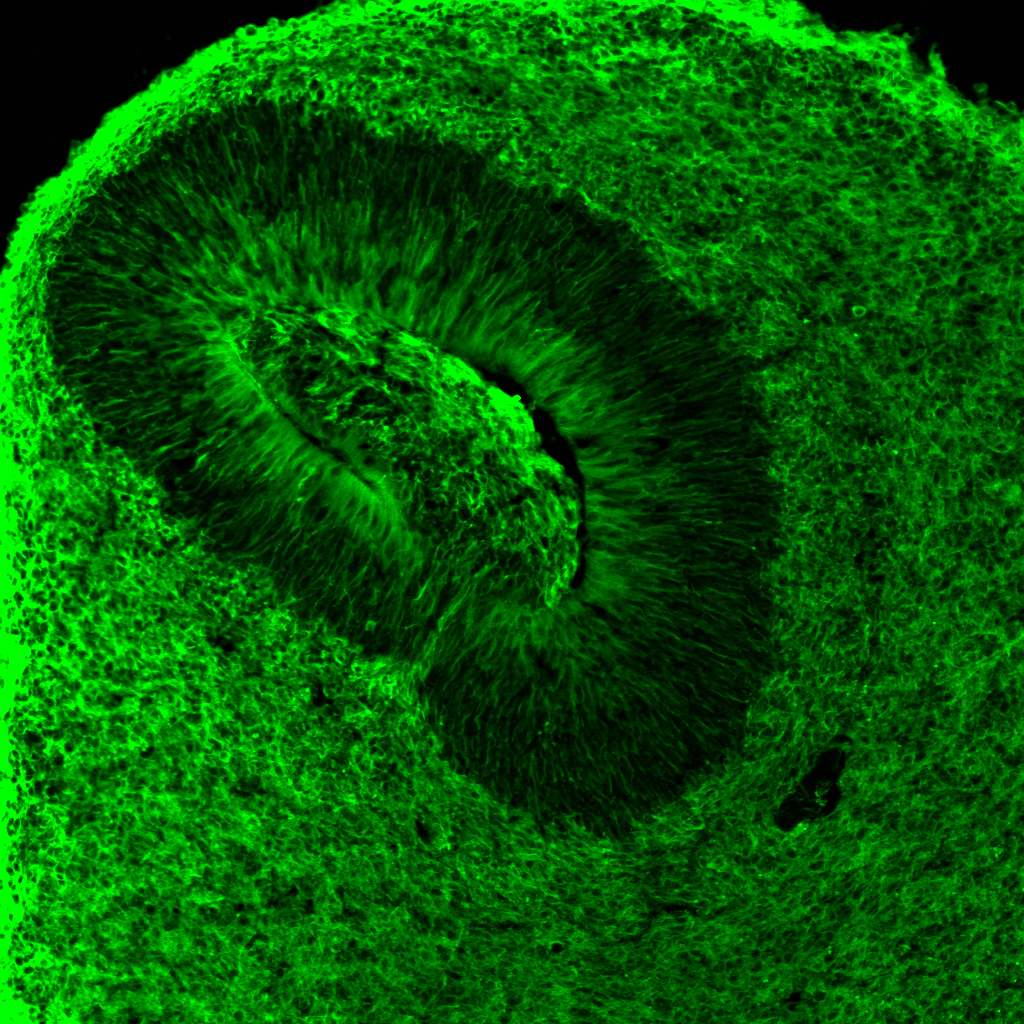

Supplement: Supplementary file 10 — Figure EV2 Source Data [file 44321_2025_302_MOESM10_ESM.zip › Figure EV2/EV2D/#15-4_TUJ1.tif]

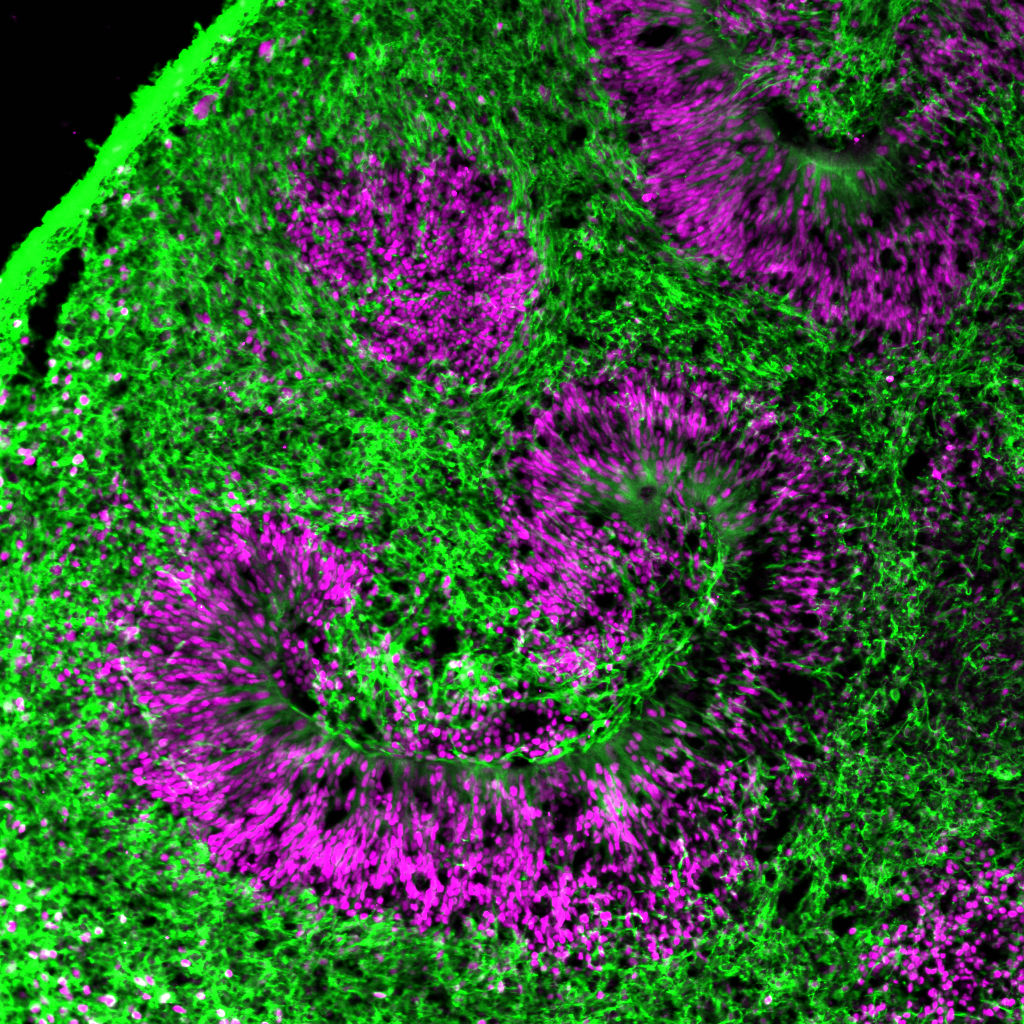

Supplement: Supplementary file 10 — Figure EV2 Source Data [file 44321_2025_302_MOESM10_ESM.zip › Figure EV2/EV2D/#6-6_merge.tif]
