# Supplementary figures and images for: CETN3 deficiency induces microcephaly by disrupting neural stem/progenitor cell fate through impaired centrosome assembly and RNA splicing (part 3 of 5)
Source: EMBO Mol Med. 2025 Sep 8;17(10):2735–61. doi: 10.1038/s44321-025-00302-7 (PMC12514221; doi:10.1038/s44321-025-00302-7)

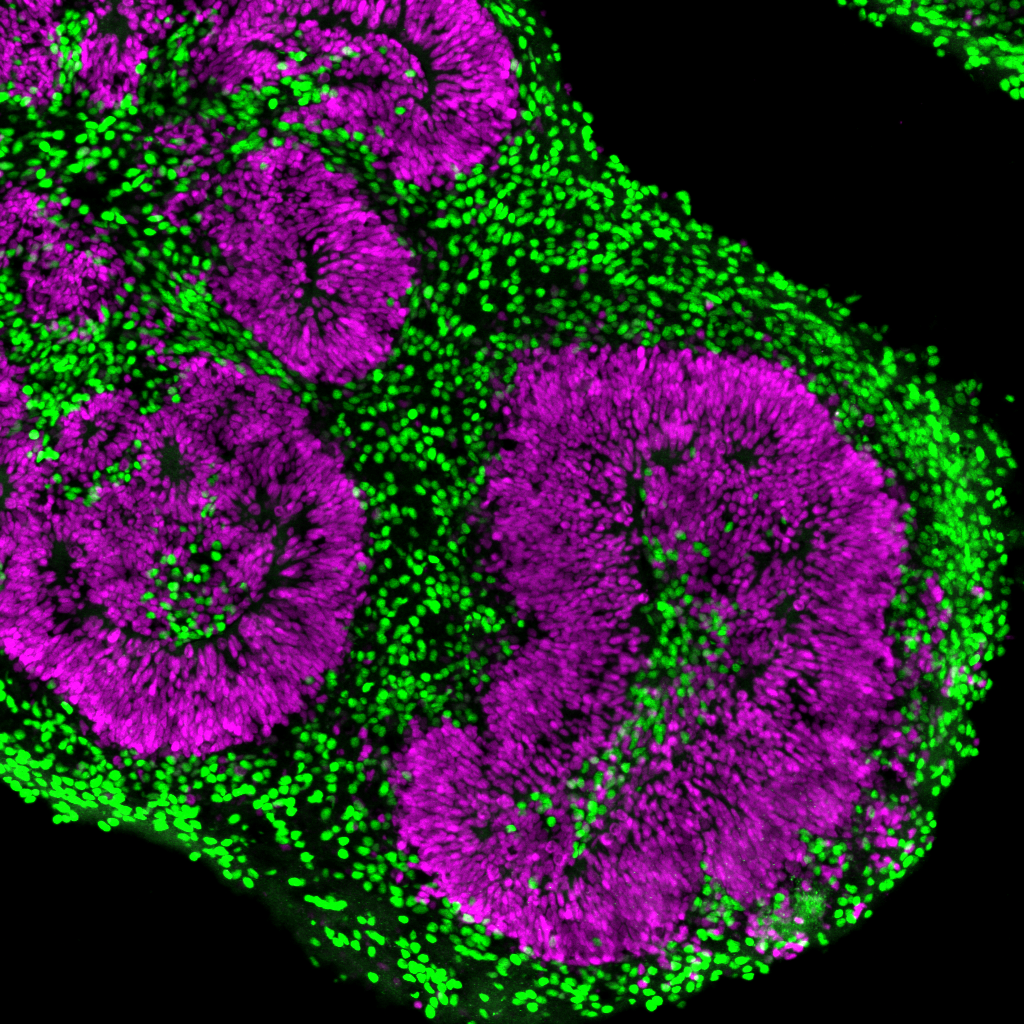

Supplement: Supplementary file 10 — Figure EV2 Source Data [file 44321_2025_302_MOESM10_ESM.zip › Figure EV2/EV2C/H1_merge.tif]

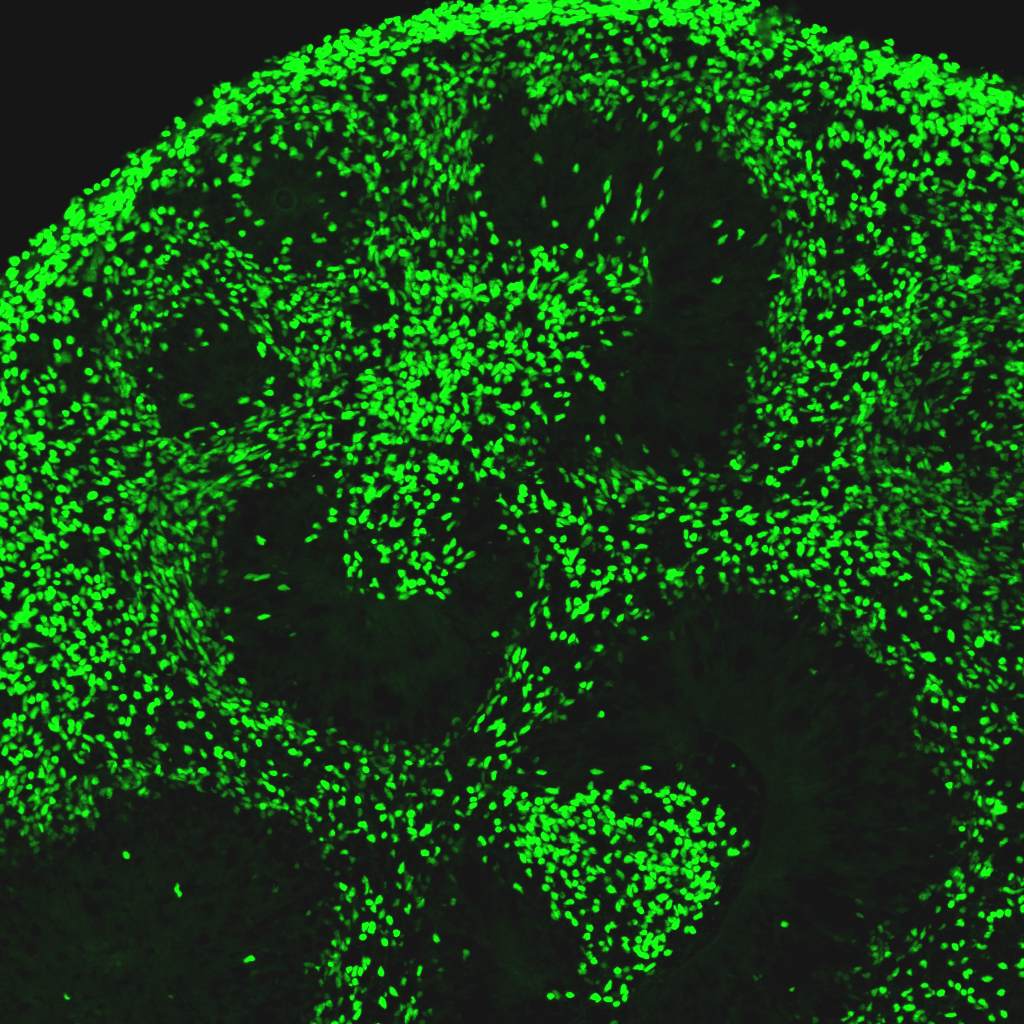

Supplement: Supplementary file 10 — Figure EV2 Source Data [file 44321_2025_302_MOESM10_ESM.zip › Figure EV2/EV2C/#6-6_TBR1.tif]

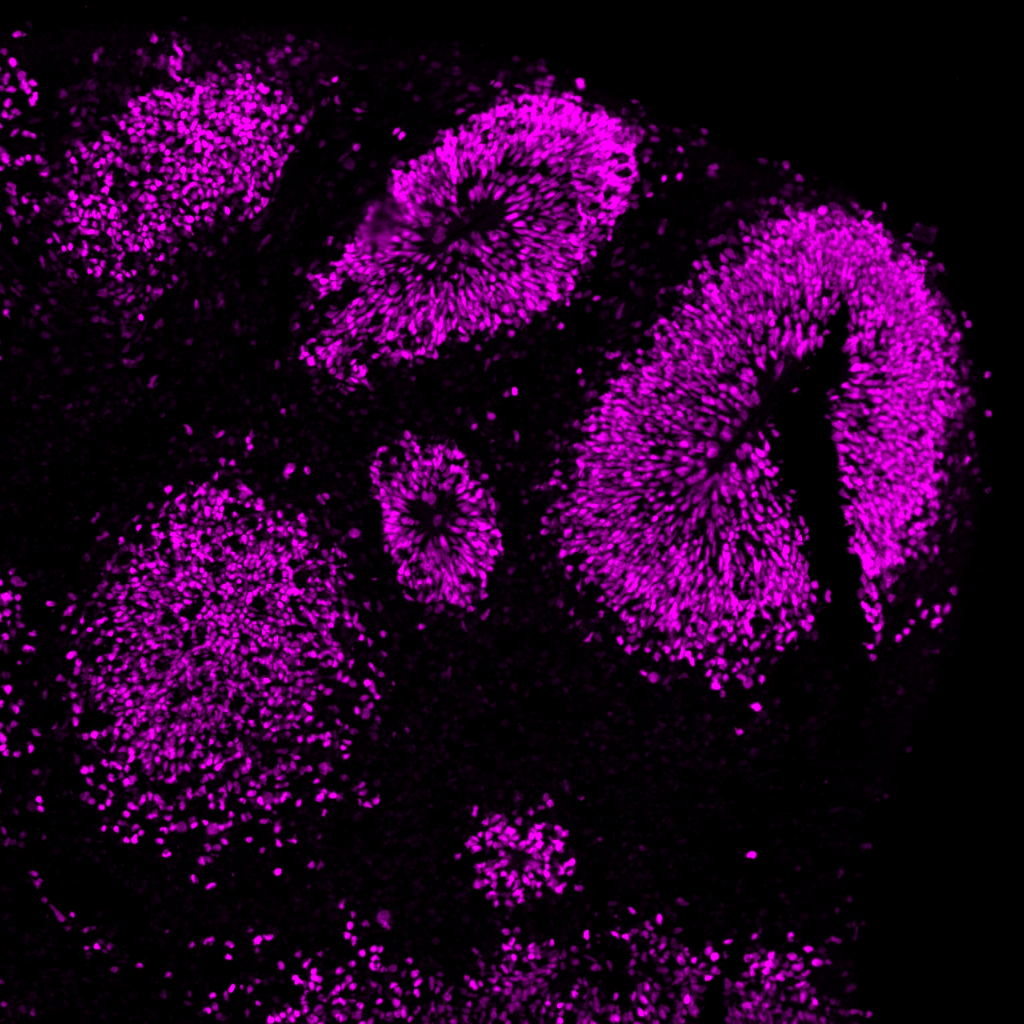

Supplement: Supplementary file 10 — Figure EV2 Source Data [file 44321_2025_302_MOESM10_ESM.zip › Figure EV2/EV2C/#15-4_SOX2.tif]

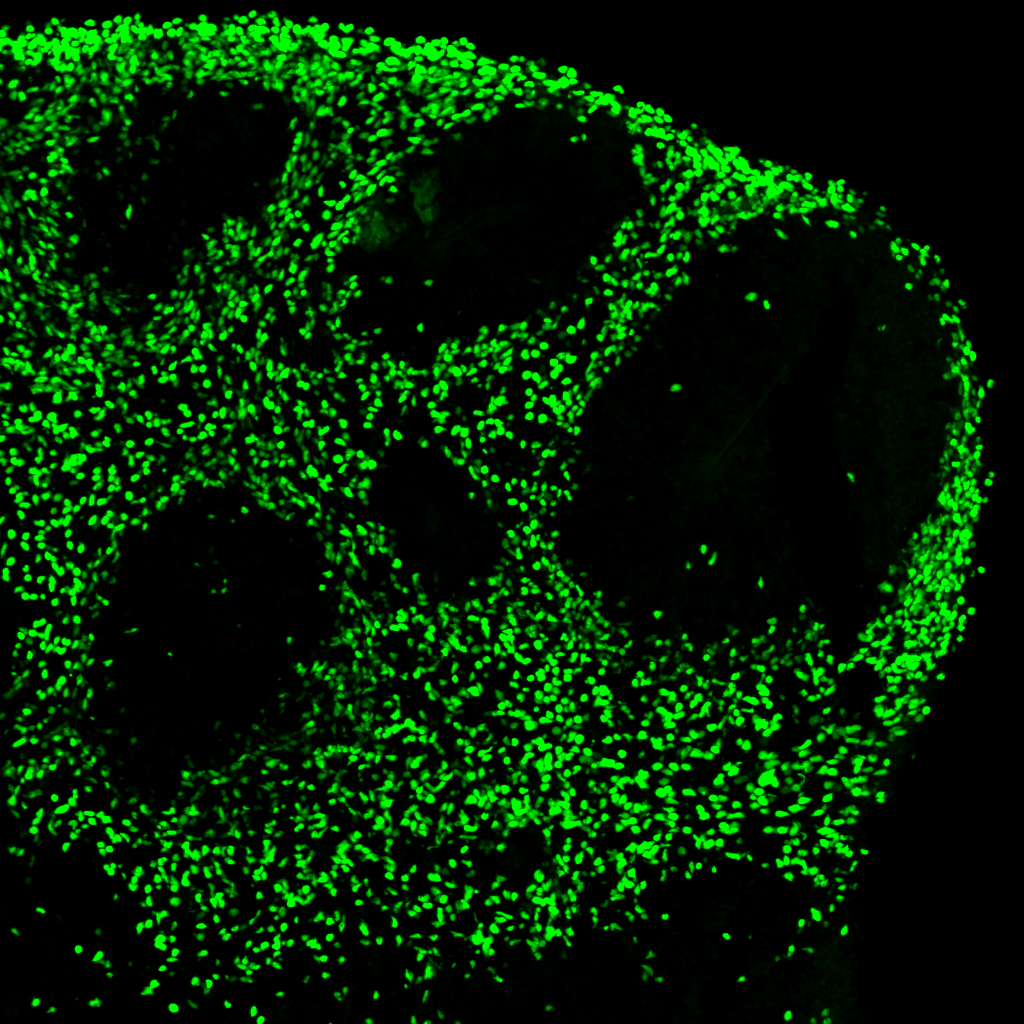

Supplement: Supplementary file 10 — Figure EV2 Source Data [file 44321_2025_302_MOESM10_ESM.zip › Figure EV2/EV2C/#15-4_TBR1.tif]

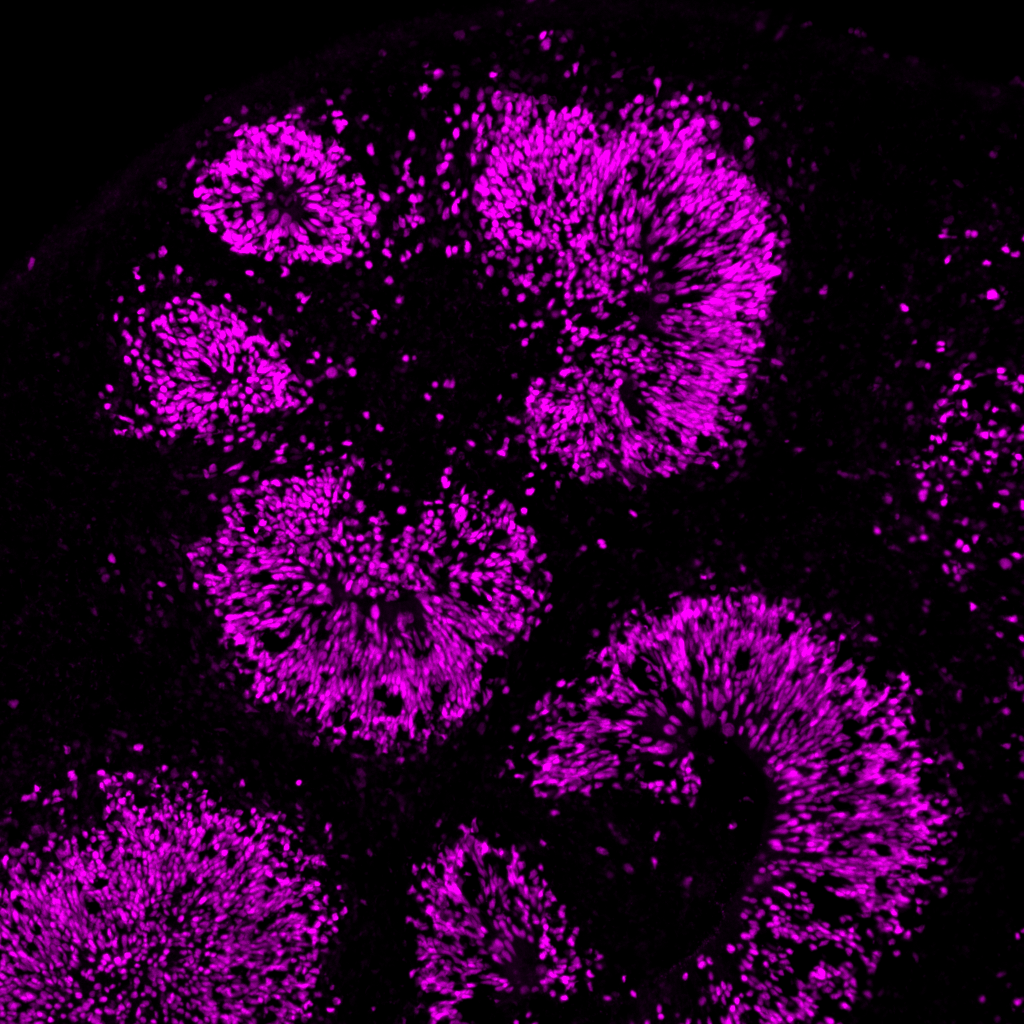

Supplement: Supplementary file 10 — Figure EV2 Source Data [file 44321_2025_302_MOESM10_ESM.zip › Figure EV2/EV2C/#6-6_SOX2.tif]

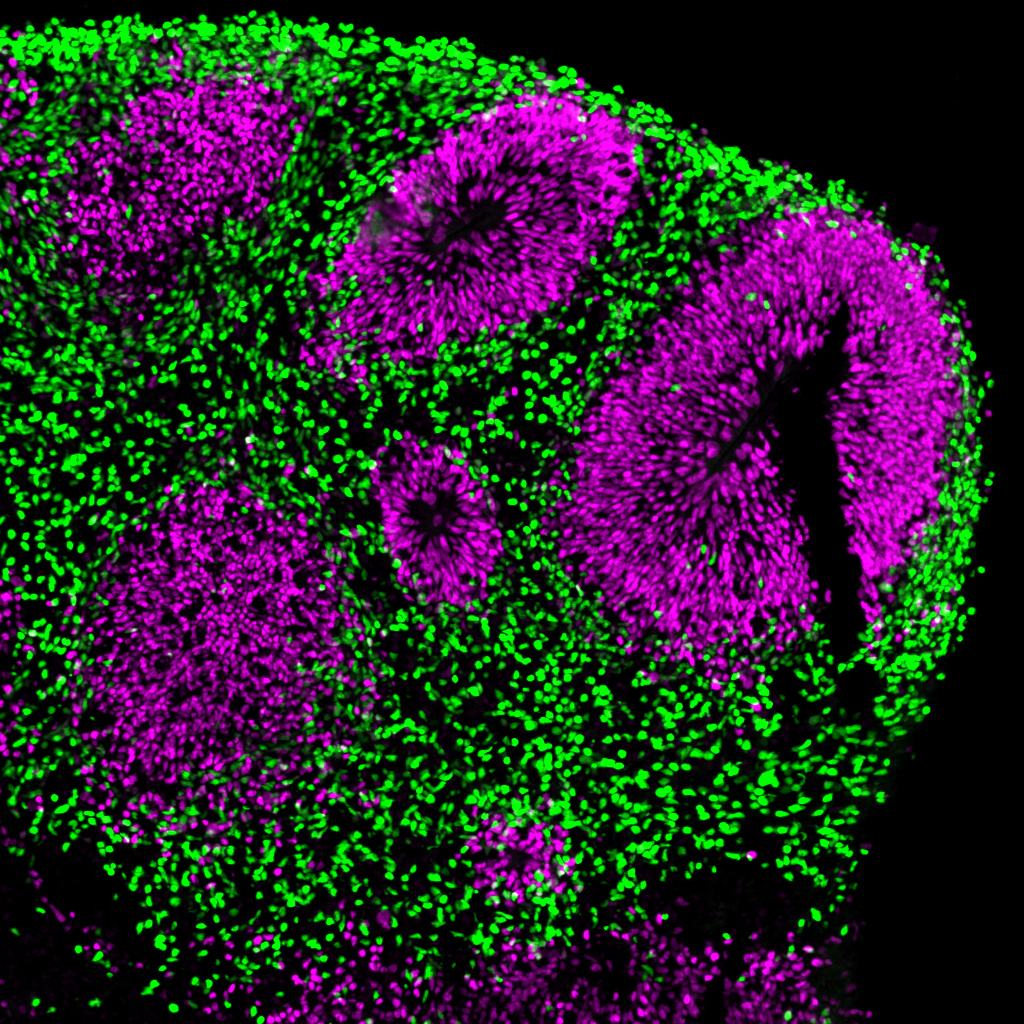

Supplement: Supplementary file 10 — Figure EV2 Source Data [file 44321_2025_302_MOESM10_ESM.zip › Figure EV2/EV2C/#15-4_merge.tif]

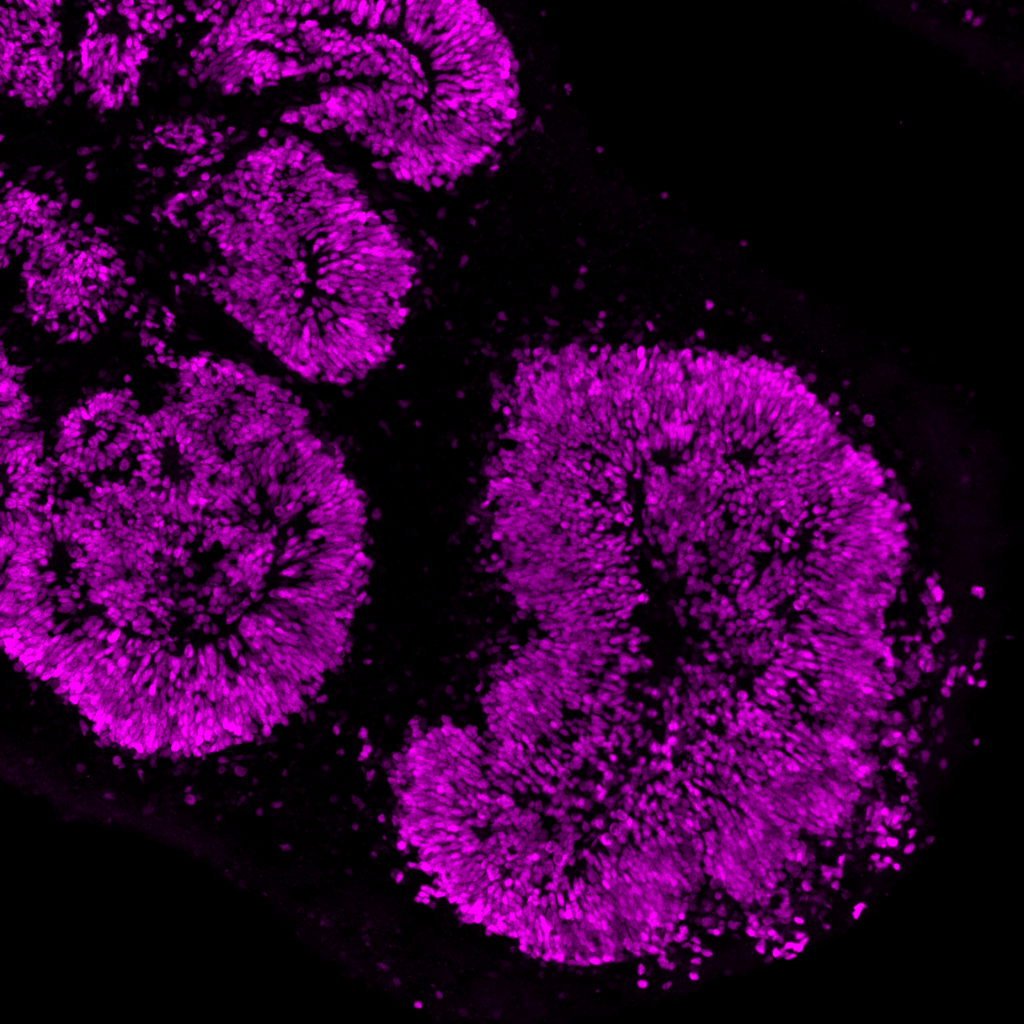

Supplement: Supplementary file 10 — Figure EV2 Source Data [file 44321_2025_302_MOESM10_ESM.zip › Figure EV2/EV2C/H1_SOX2.tif]

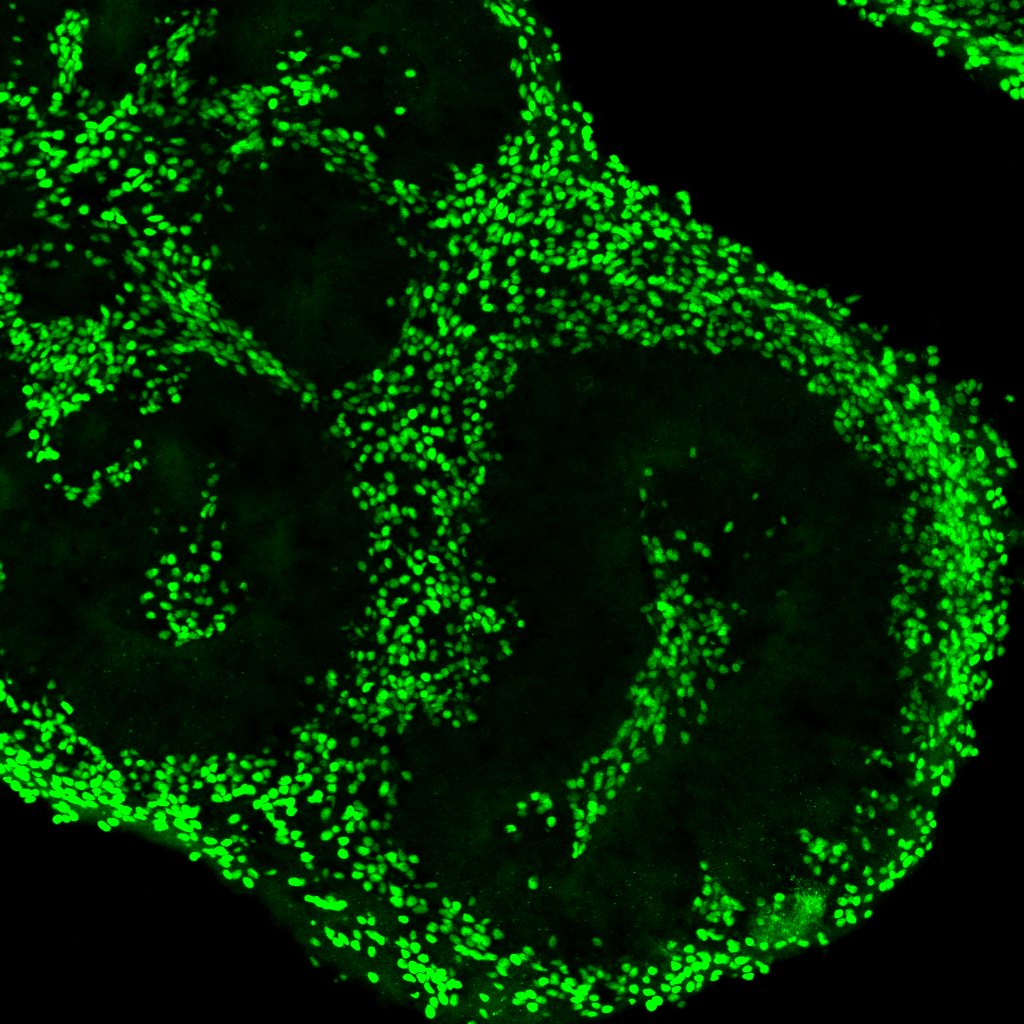

Supplement: Supplementary file 10 — Figure EV2 Source Data [file 44321_2025_302_MOESM10_ESM.zip › Figure EV2/EV2C/H1_TBR1.tif]

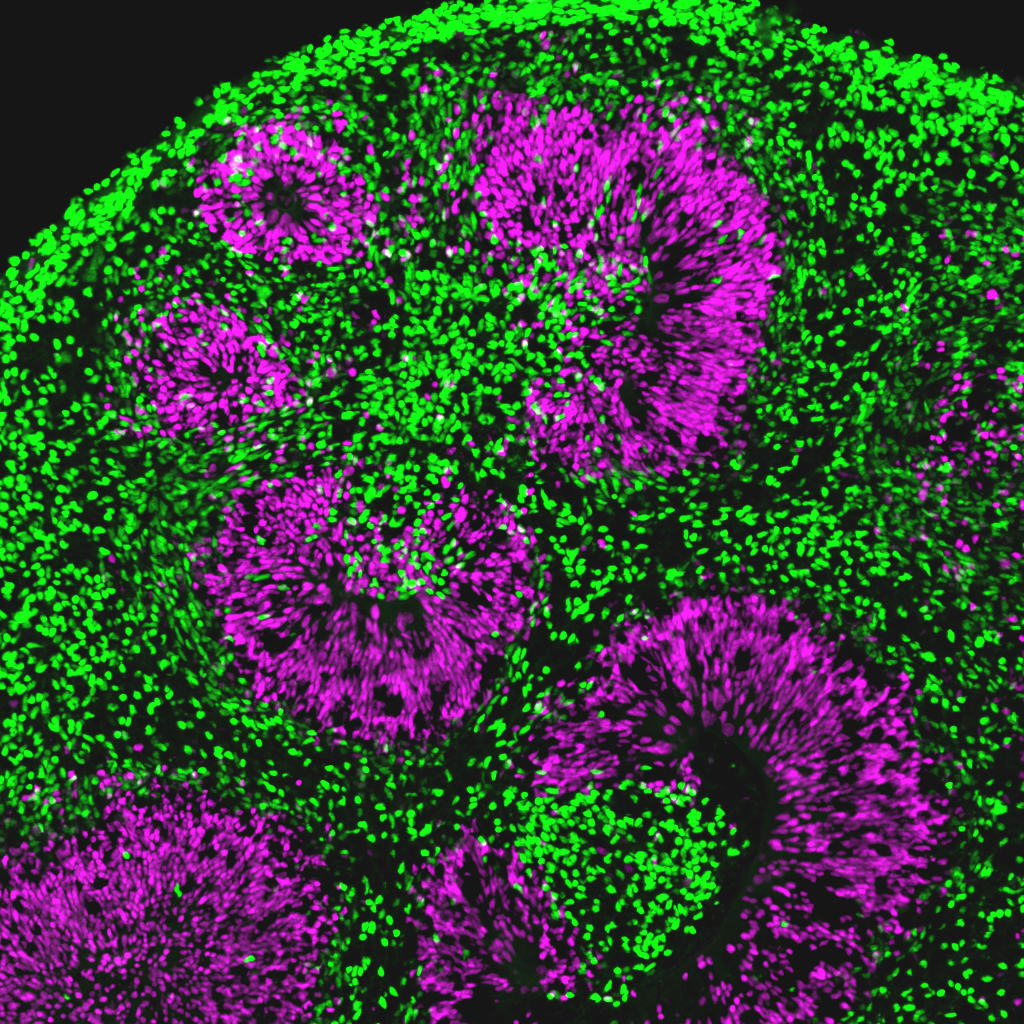

Supplement: Supplementary file 10 — Figure EV2 Source Data [file 44321_2025_302_MOESM10_ESM.zip › Figure EV2/EV2C/#6-6_merge.tif]

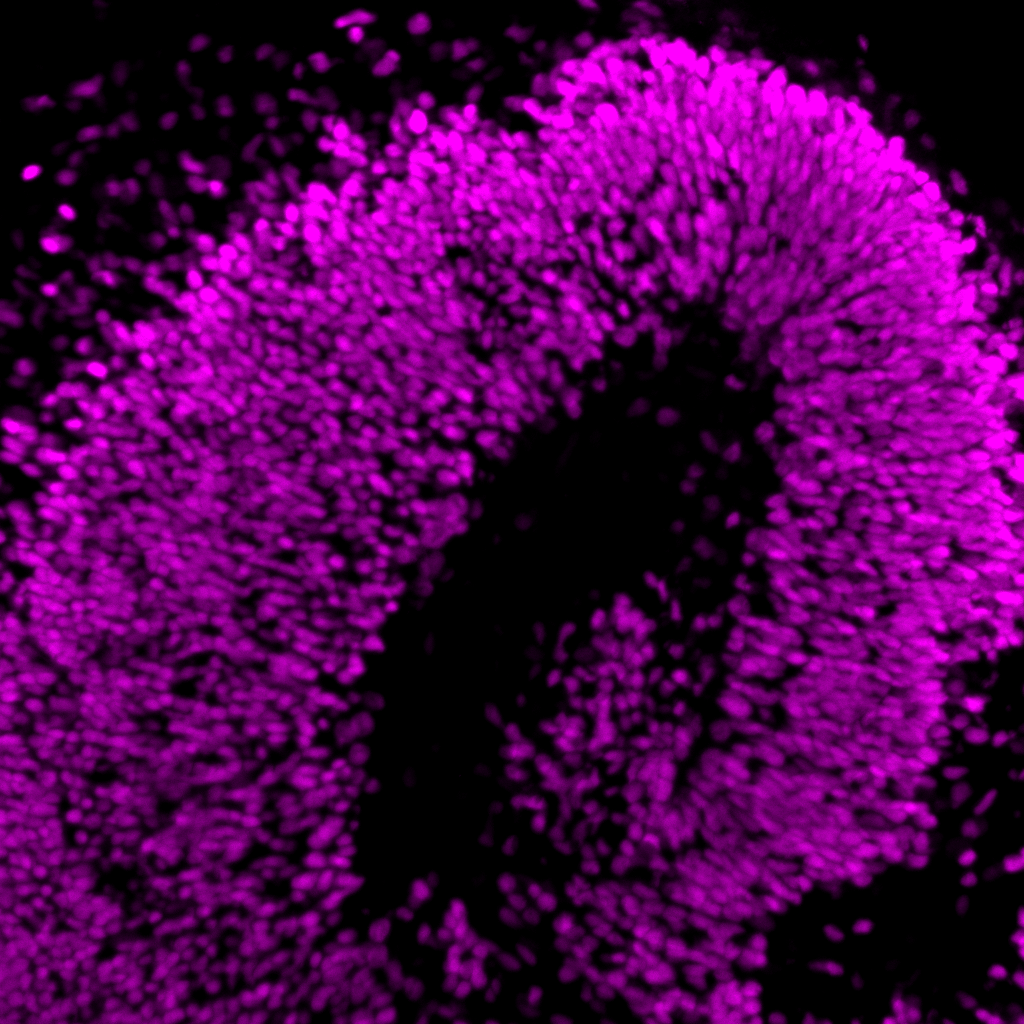

Supplement: Supplementary file 10 — Figure EV2 Source Data [file 44321_2025_302_MOESM10_ESM.zip › Figure EV2/EV2F/H1-SOX2.tif]

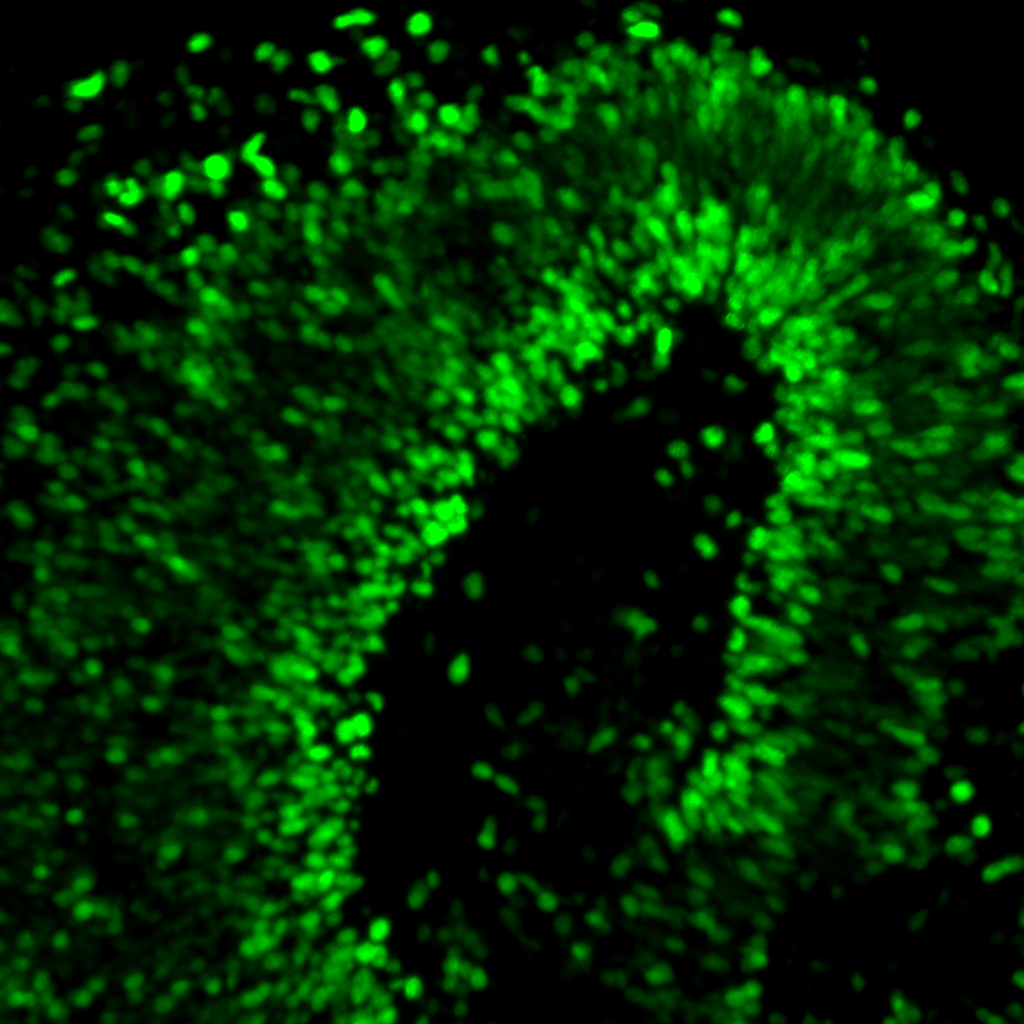

Supplement: Supplementary file 10 — Figure EV2 Source Data [file 44321_2025_302_MOESM10_ESM.zip › Figure EV2/EV2F/H1-Ki67.tif]

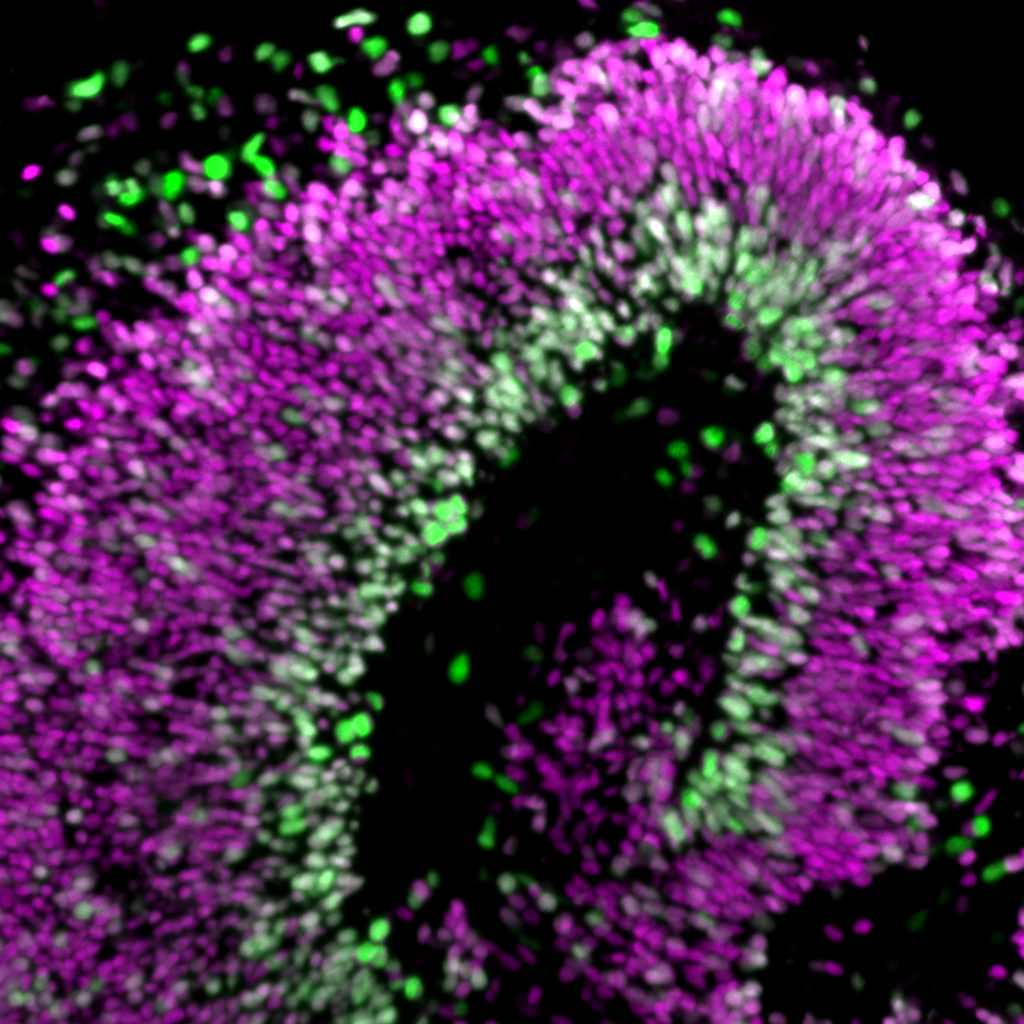

Supplement: Supplementary file 10 — Figure EV2 Source Data [file 44321_2025_302_MOESM10_ESM.zip › Figure EV2/EV2F/H1-merge.tif]

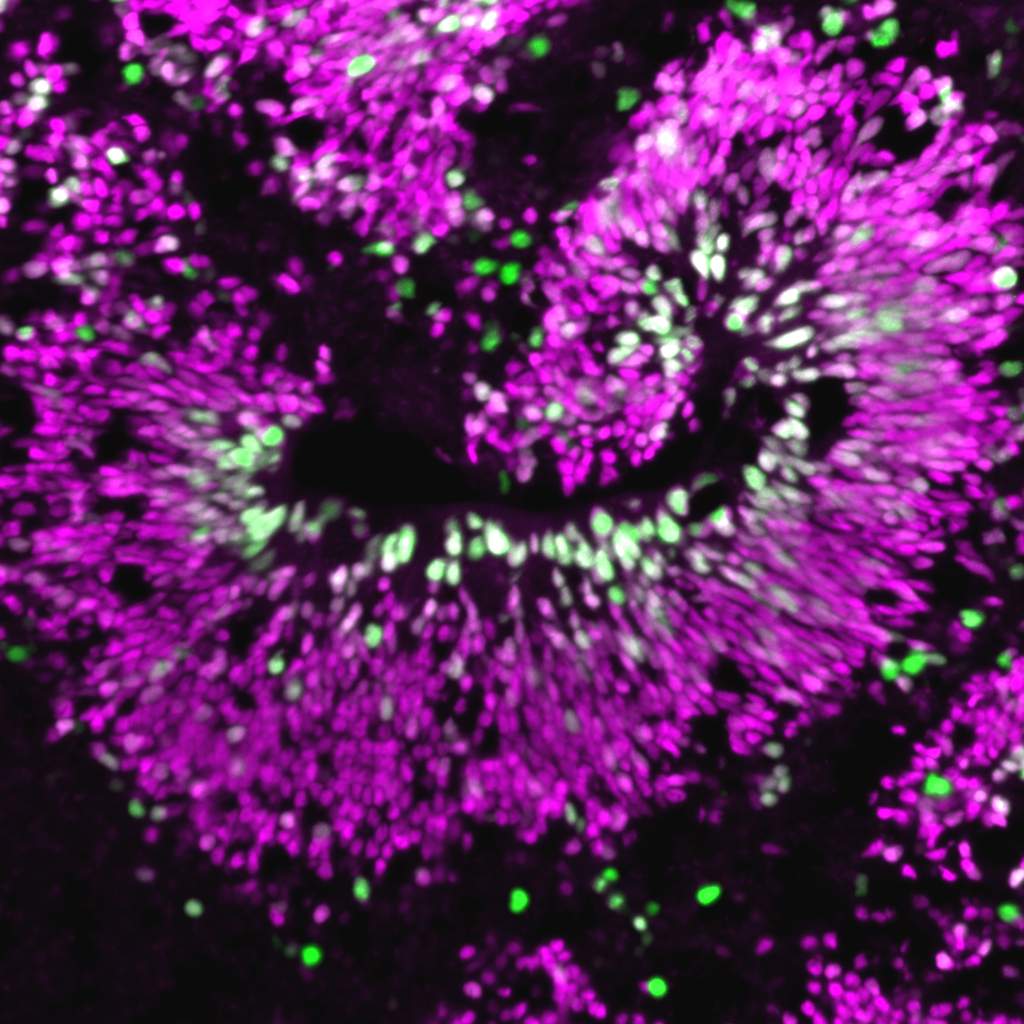

Supplement: Supplementary file 10 — Figure EV2 Source Data [file 44321_2025_302_MOESM10_ESM.zip › Figure EV2/EV2F/#15-4-merge.tif]

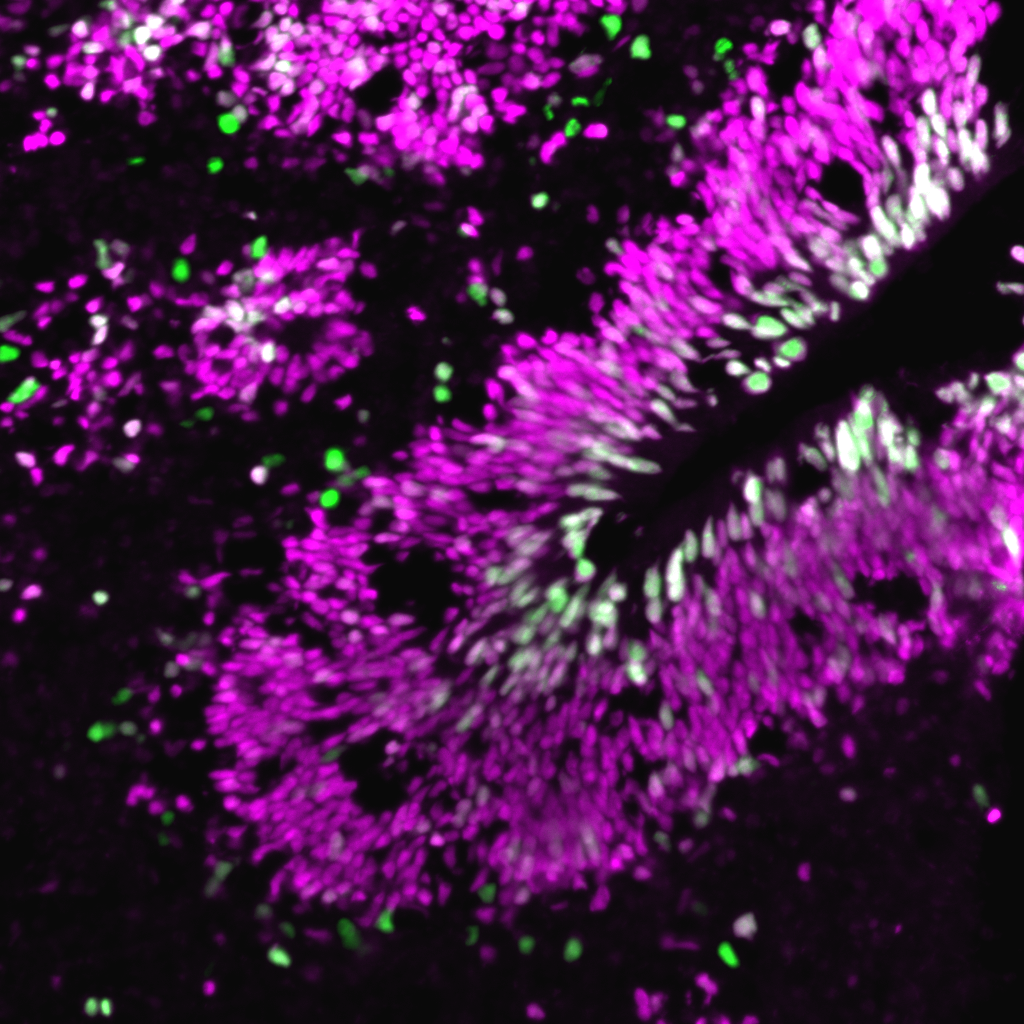

Supplement: Supplementary file 10 — Figure EV2 Source Data [file 44321_2025_302_MOESM10_ESM.zip › Figure EV2/EV2F/#6-6-merge.tif]

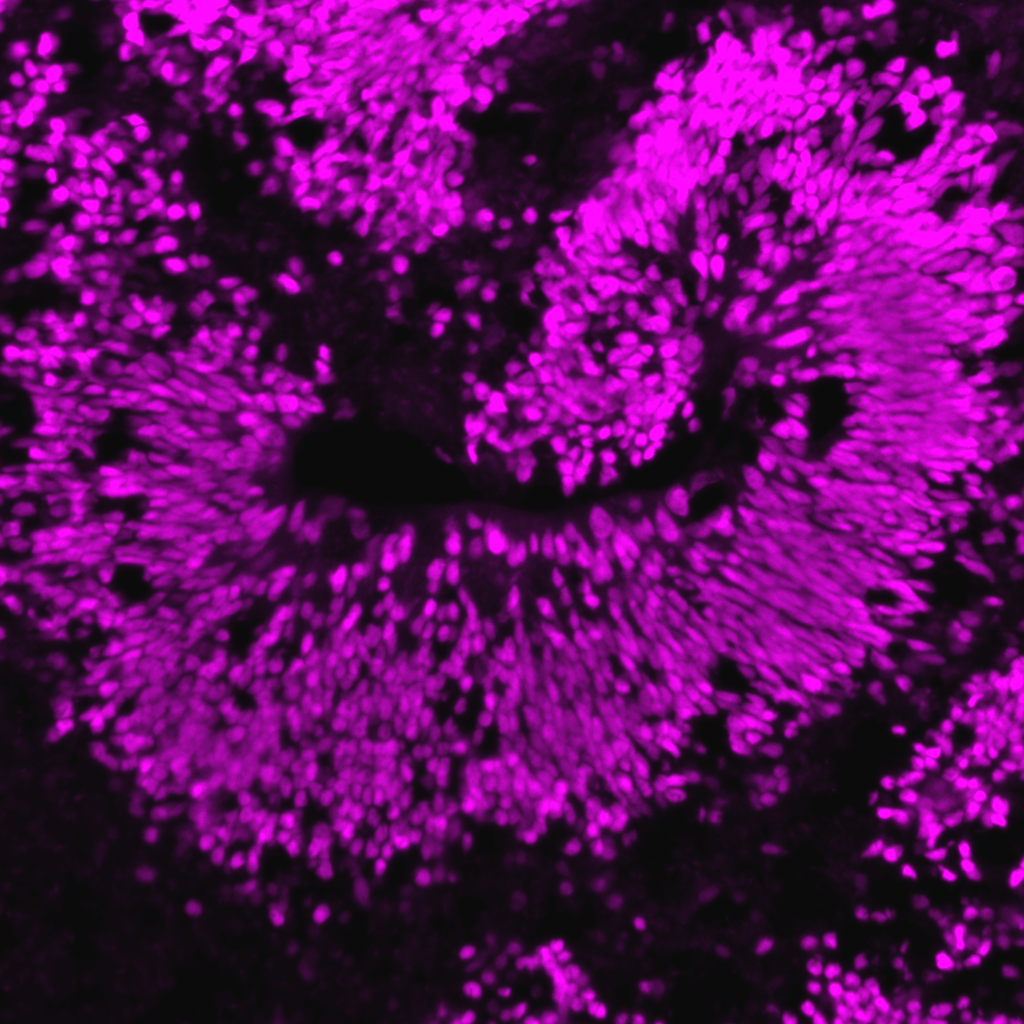

Supplement: Supplementary file 10 — Figure EV2 Source Data [file 44321_2025_302_MOESM10_ESM.zip › Figure EV2/EV2F/#15-4-SOX2.tif]

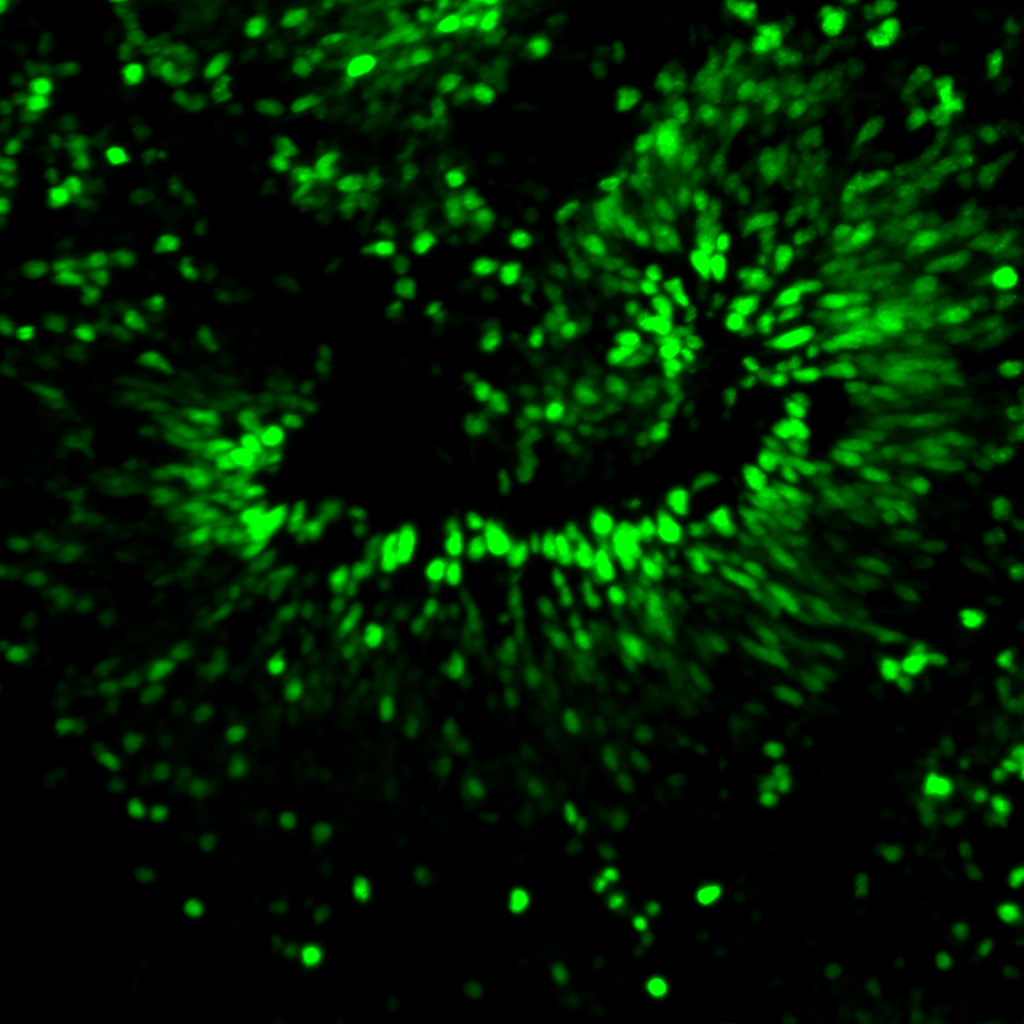

Supplement: Supplementary file 10 — Figure EV2 Source Data [file 44321_2025_302_MOESM10_ESM.zip › Figure EV2/EV2F/#15-4-Ki67.tif]

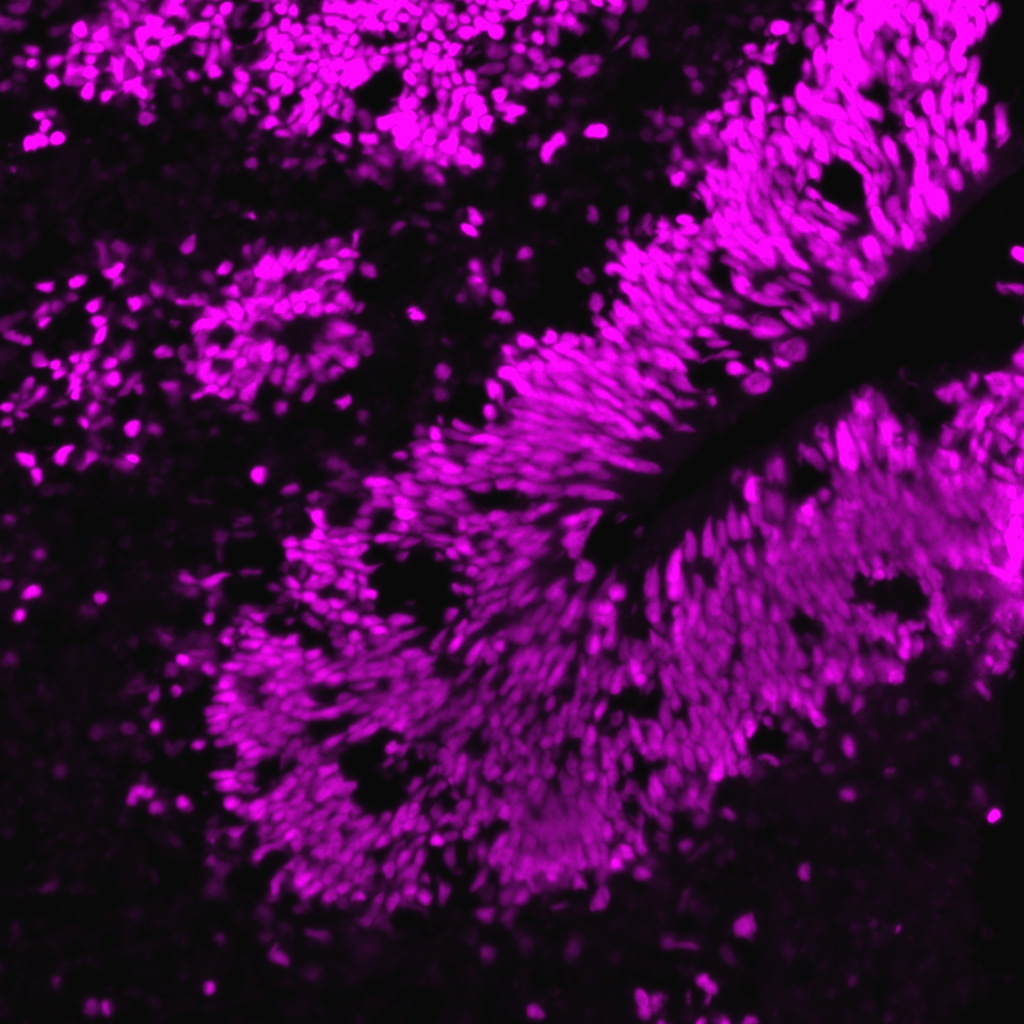

Supplement: Supplementary file 10 — Figure EV2 Source Data [file 44321_2025_302_MOESM10_ESM.zip › Figure EV2/EV2F/#6-6-SOX2.tif]

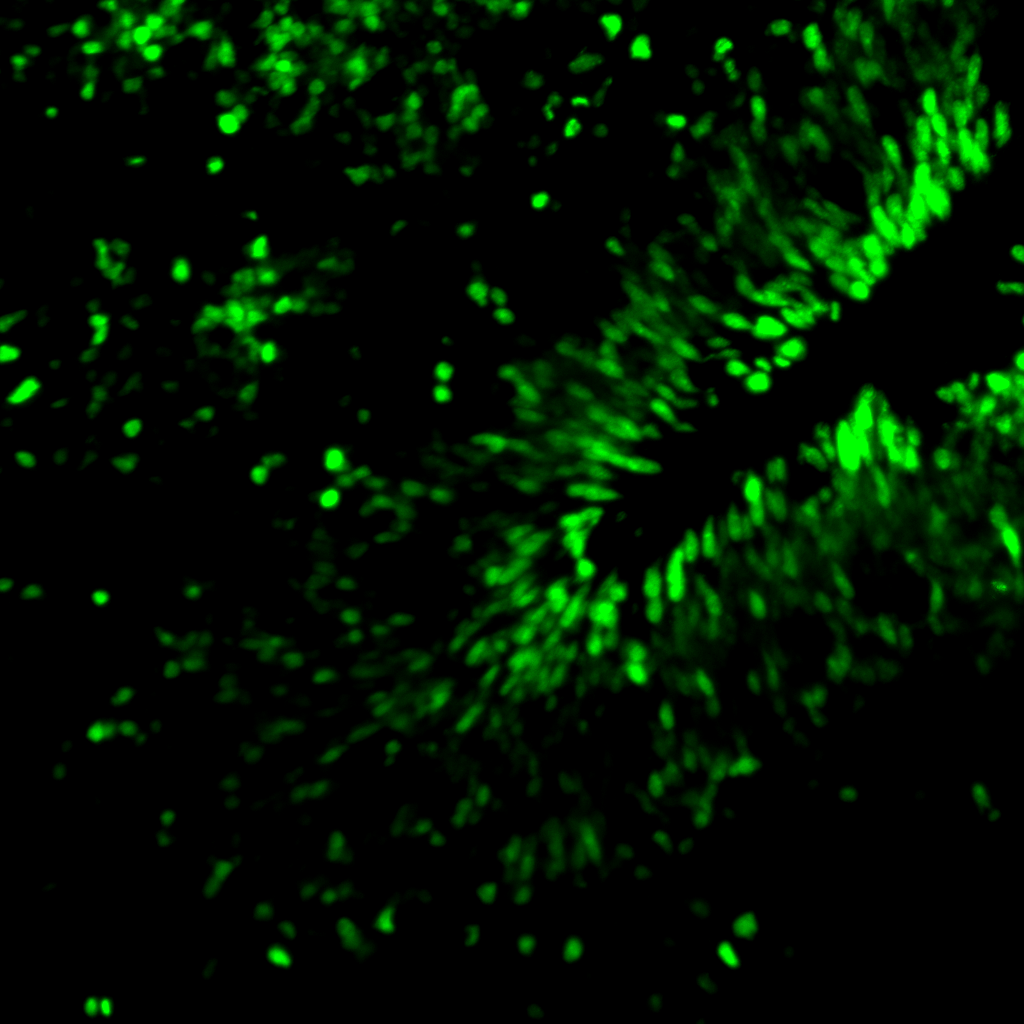

Supplement: Supplementary file 10 — Figure EV2 Source Data [file 44321_2025_302_MOESM10_ESM.zip › Figure EV2/EV2F/#6-6-Ki67.tif]

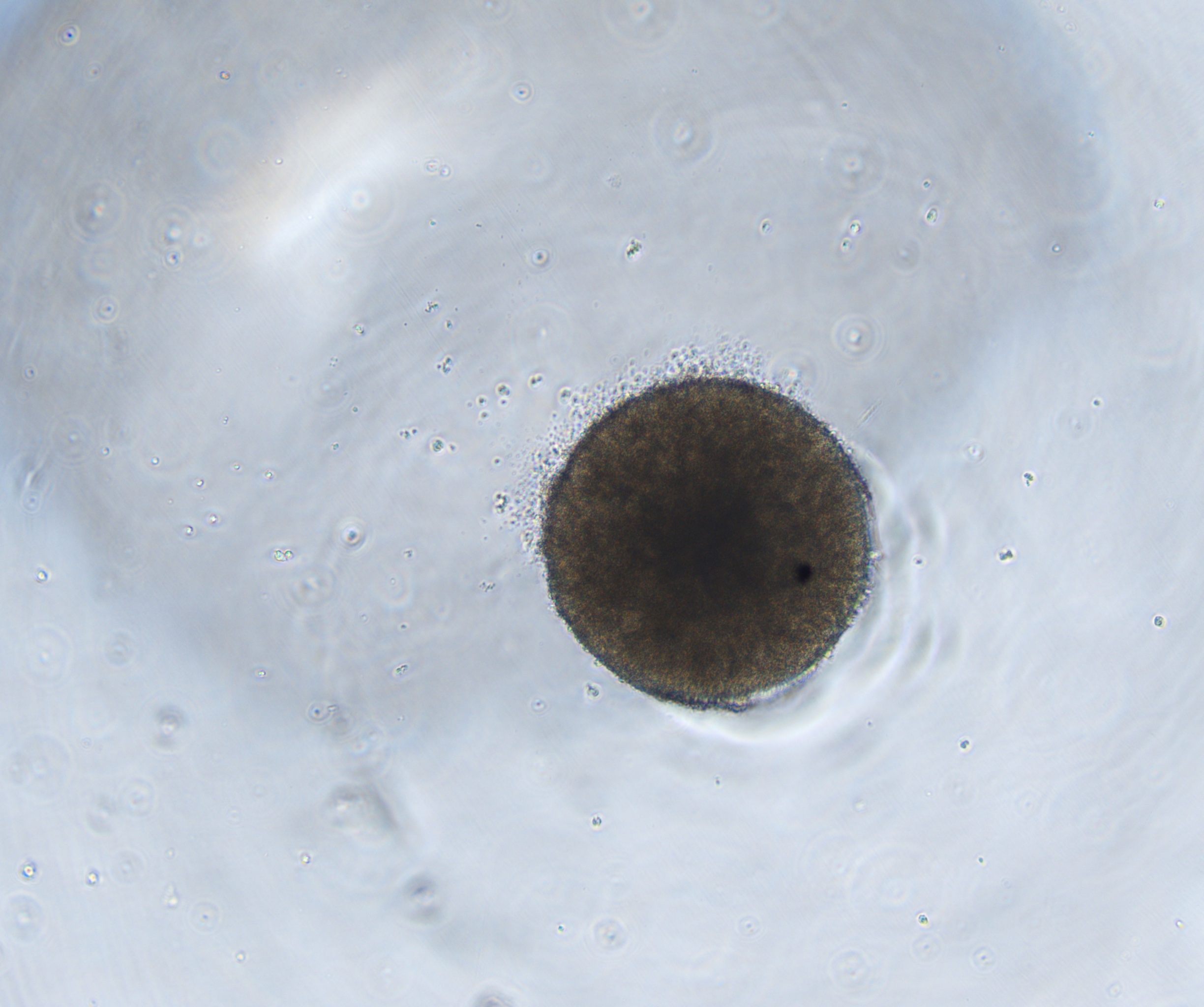

Supplement: Supplementary file 10 — Figure EV2 Source Data [file 44321_2025_302_MOESM10_ESM.zip › Figure EV2/EV2A/Day3_H1.jpg]

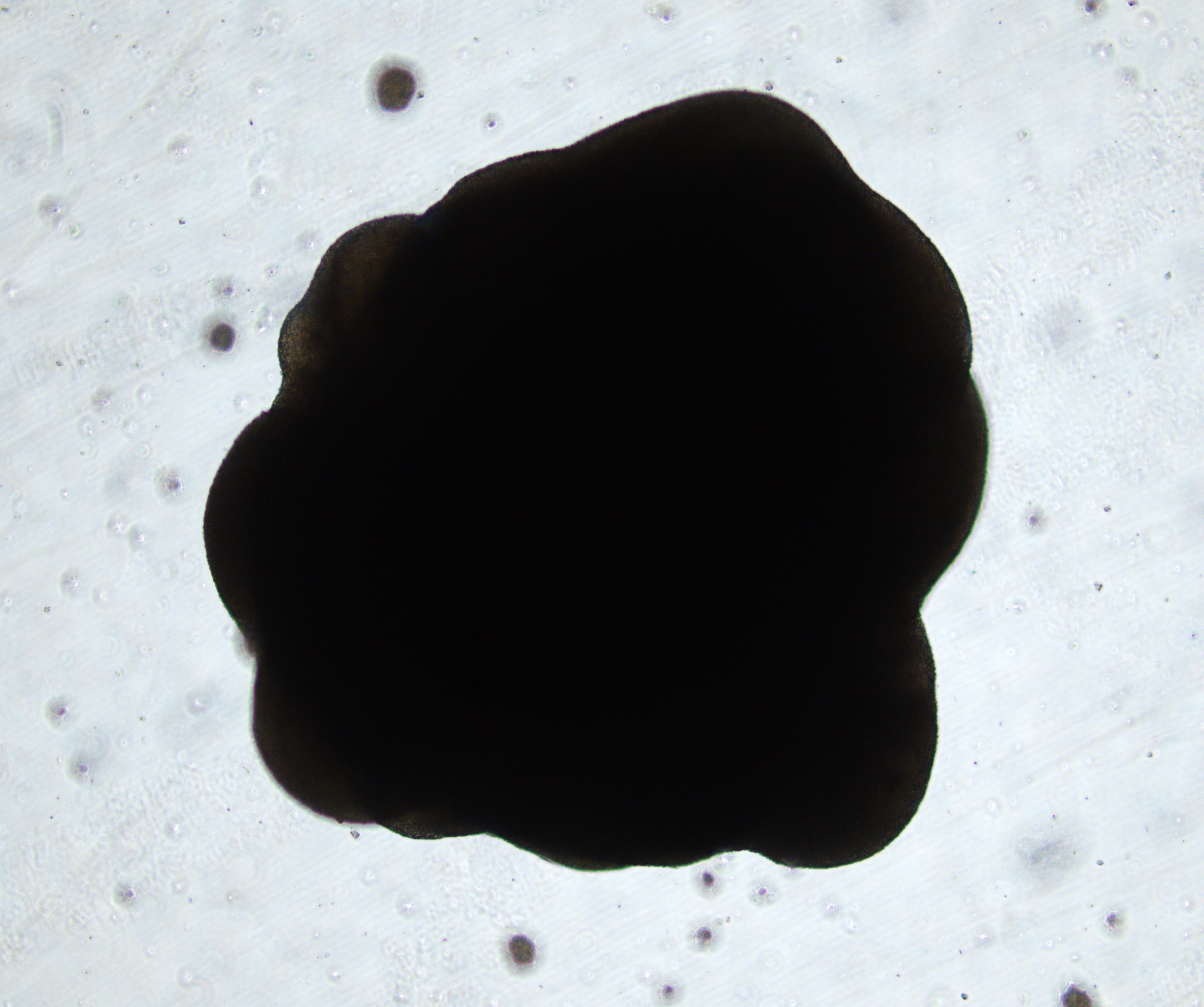

Supplement: Supplementary file 10 — Figure EV2 Source Data [file 44321_2025_302_MOESM10_ESM.zip › Figure EV2/EV2A/Day45_15-4.jpeg]

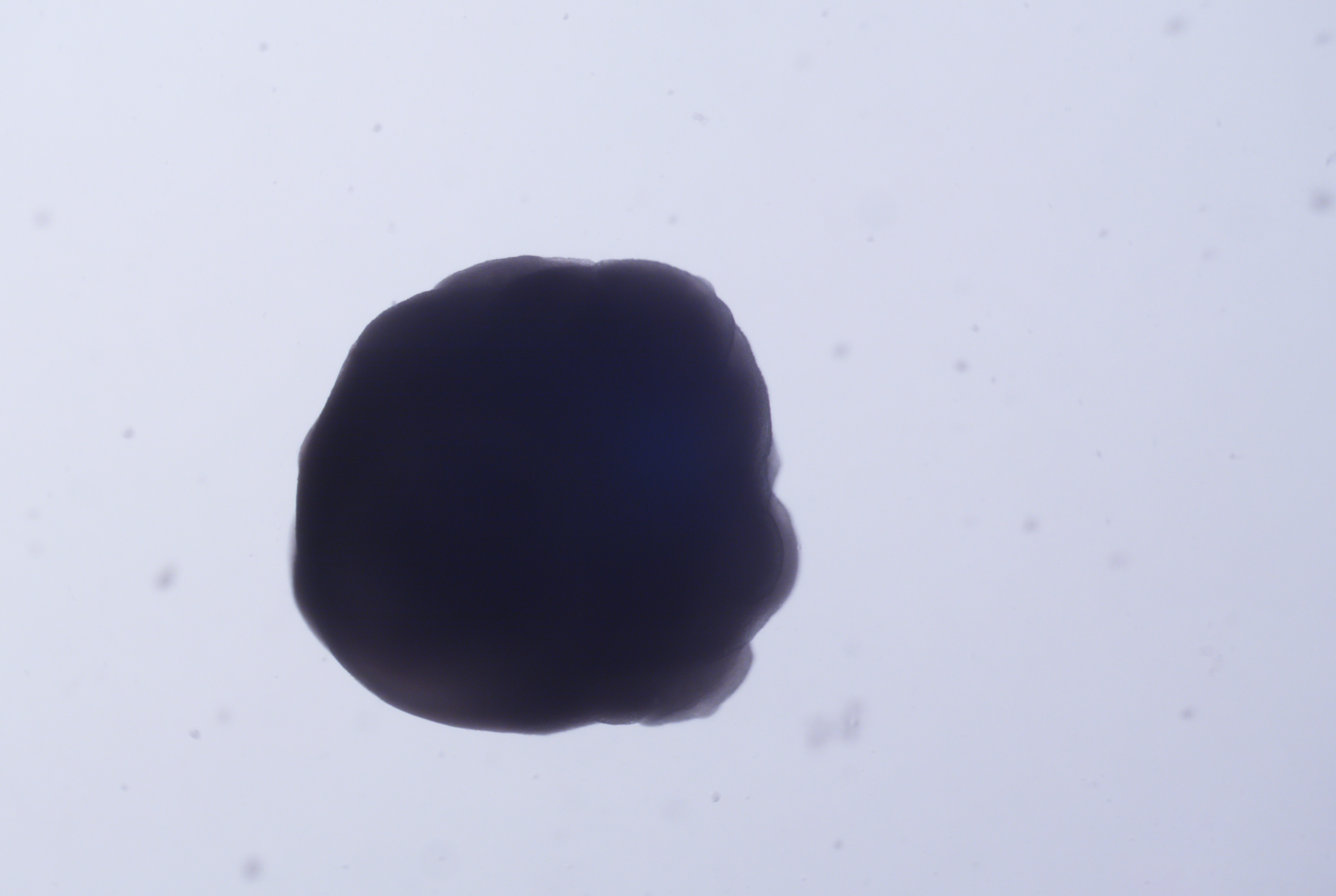

Supplement: Supplementary file 10 — Figure EV2 Source Data [file 44321_2025_302_MOESM10_ESM.zip › Figure EV2/EV2A/Day60_6-6.jpeg]

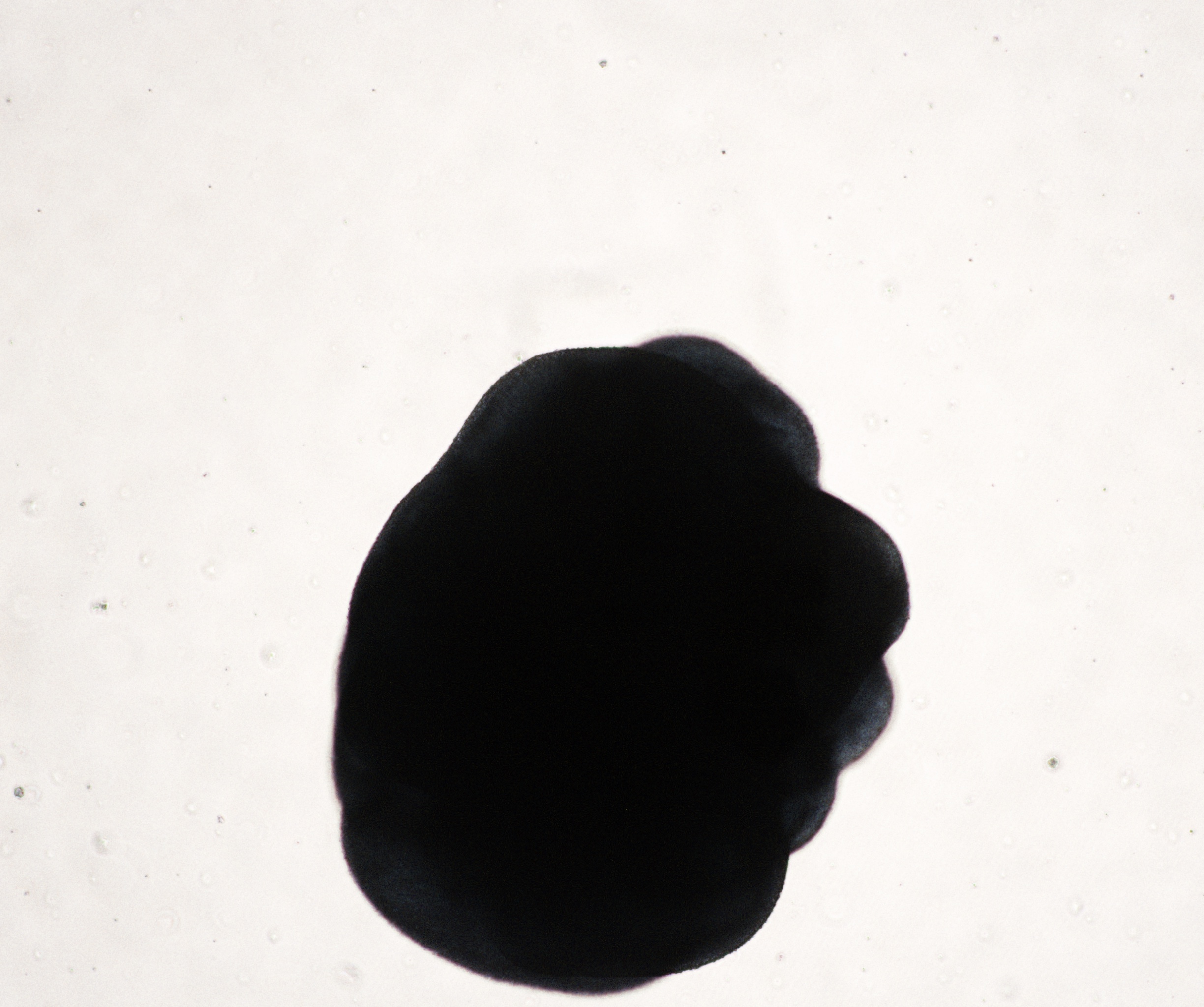

Supplement: Supplementary file 10 — Figure EV2 Source Data [file 44321_2025_302_MOESM10_ESM.zip › Figure EV2/EV2A/Day35_6-6.jpeg]

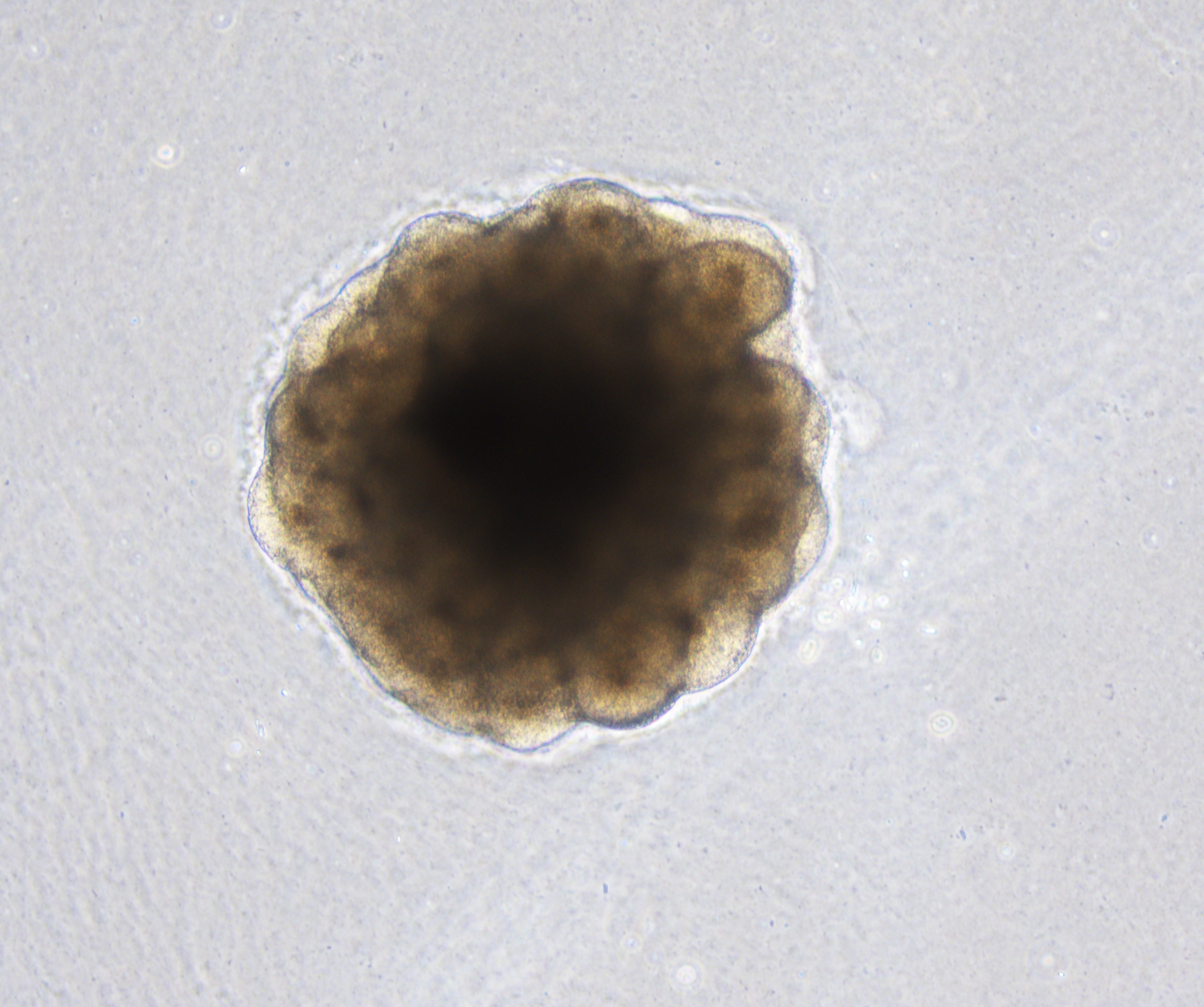

Supplement: Supplementary file 10 — Figure EV2 Source Data [file 44321_2025_302_MOESM10_ESM.zip › Figure EV2/EV2A/Day15_15-4.jpeg]

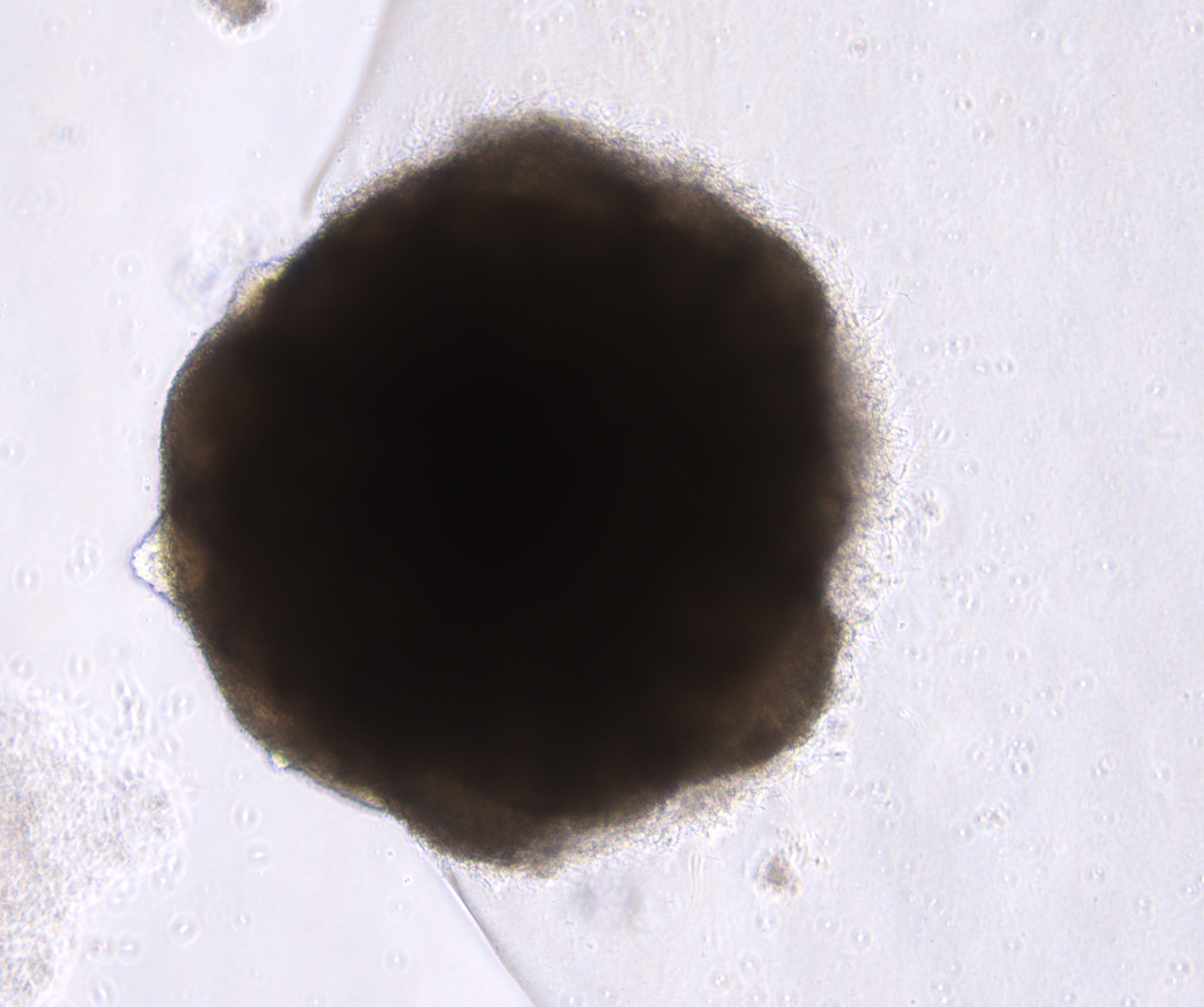

Supplement: Supplementary file 10 — Figure EV2 Source Data [file 44321_2025_302_MOESM10_ESM.zip › Figure EV2/EV2A/Day20_15-4.jpeg]

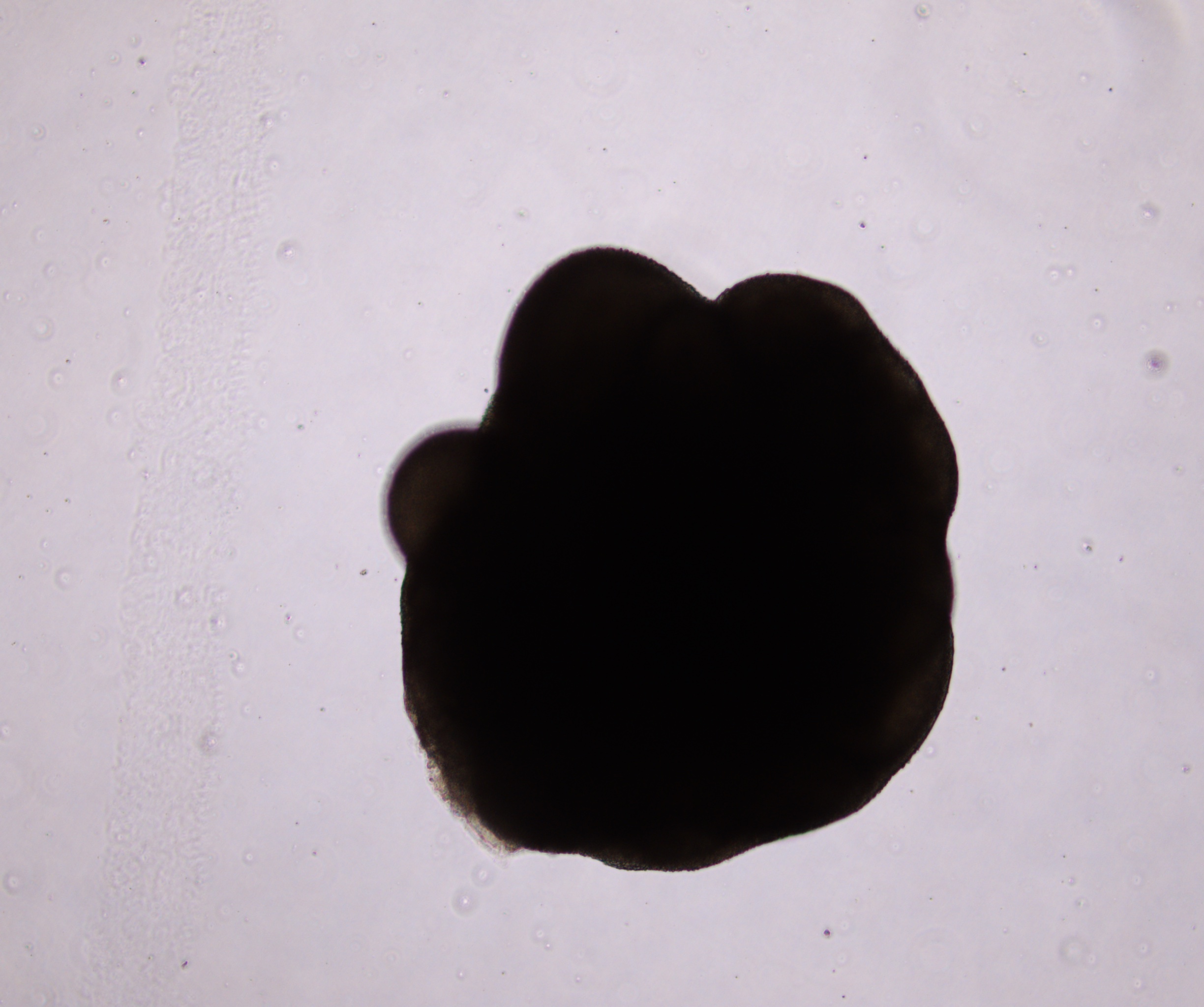

Supplement: Supplementary file 10 — Figure EV2 Source Data [file 44321_2025_302_MOESM10_ESM.zip › Figure EV2/EV2A/Day30_H1.jpeg]

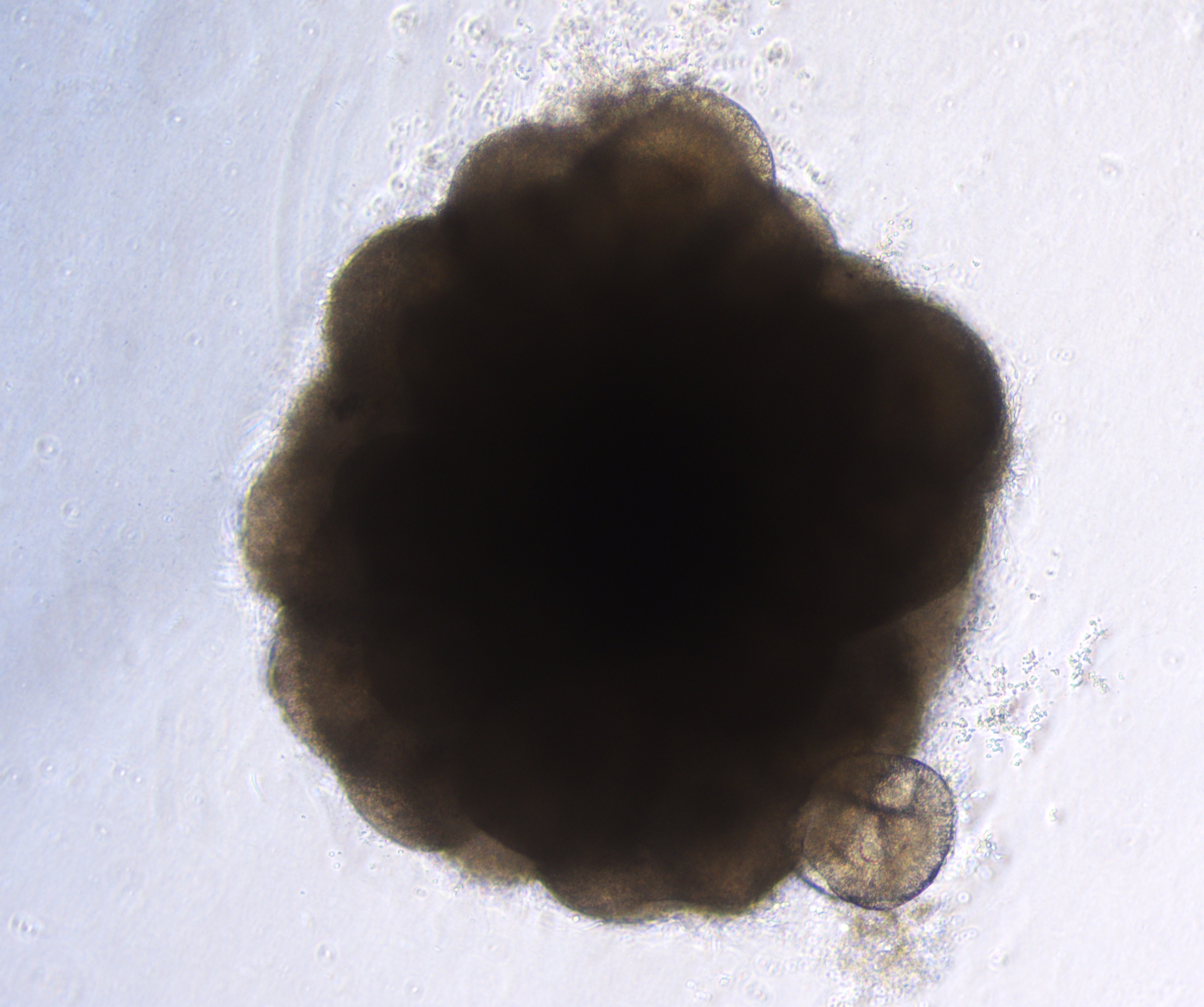

Supplement: Supplementary file 10 — Figure EV2 Source Data [file 44321_2025_302_MOESM10_ESM.zip › Figure EV2/EV2A/Day20_H1.jpeg]

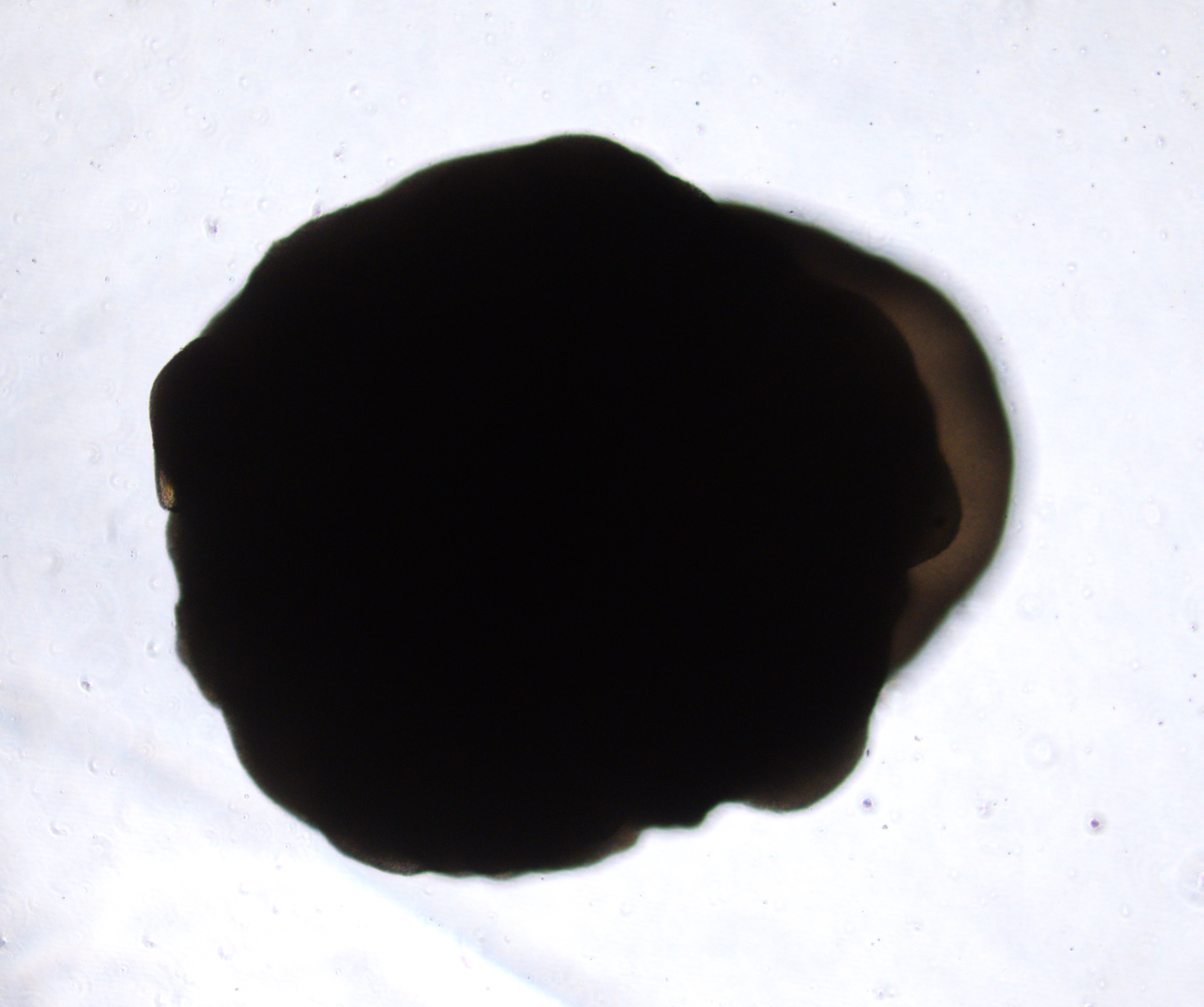

Supplement: Supplementary file 10 — Figure EV2 Source Data [file 44321_2025_302_MOESM10_ESM.zip › Figure EV2/EV2A/Day45_6-6.jpeg]

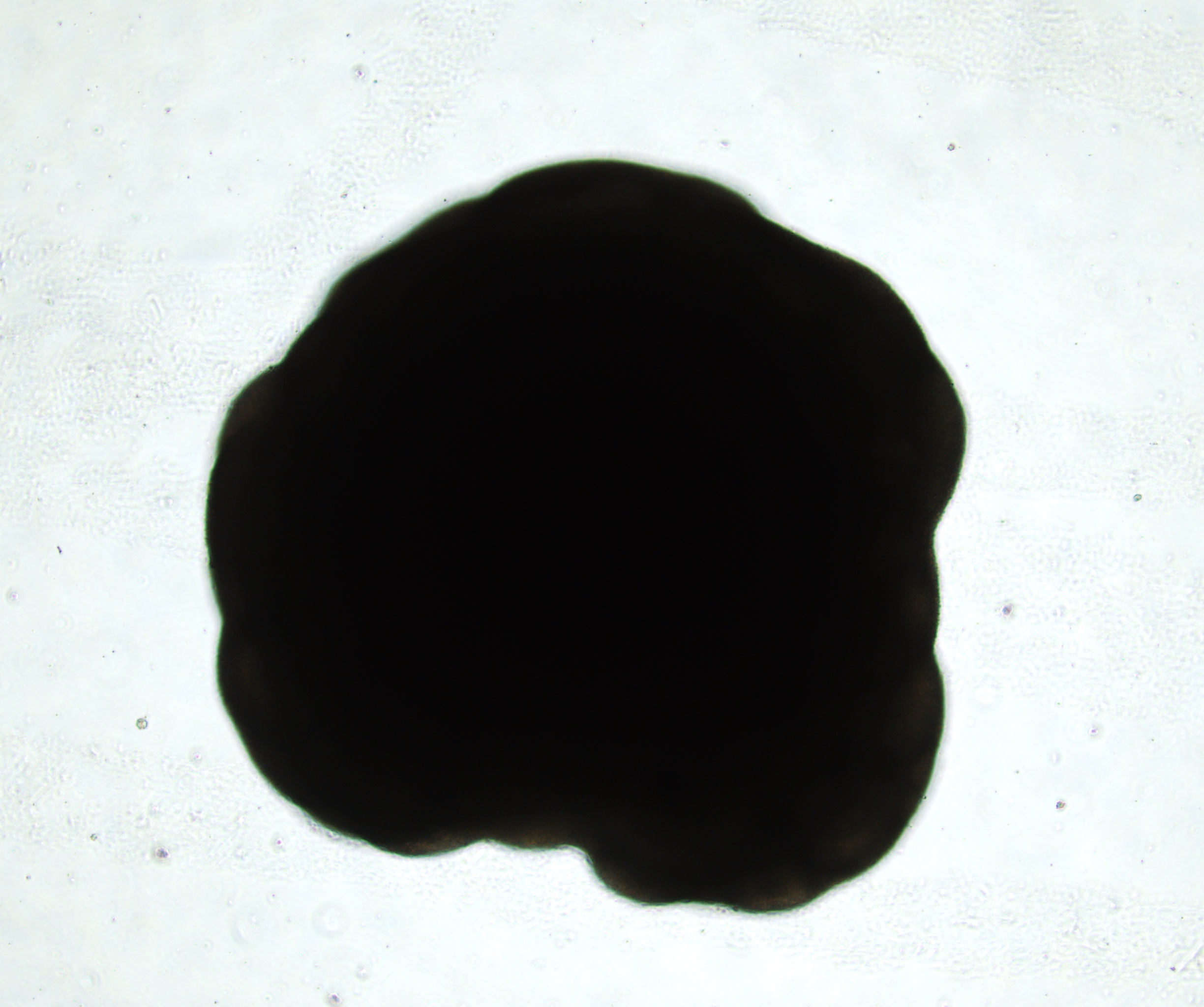

Supplement: Supplementary file 10 — Figure EV2 Source Data [file 44321_2025_302_MOESM10_ESM.zip › Figure EV2/EV2A/Day40_H1.jpeg]

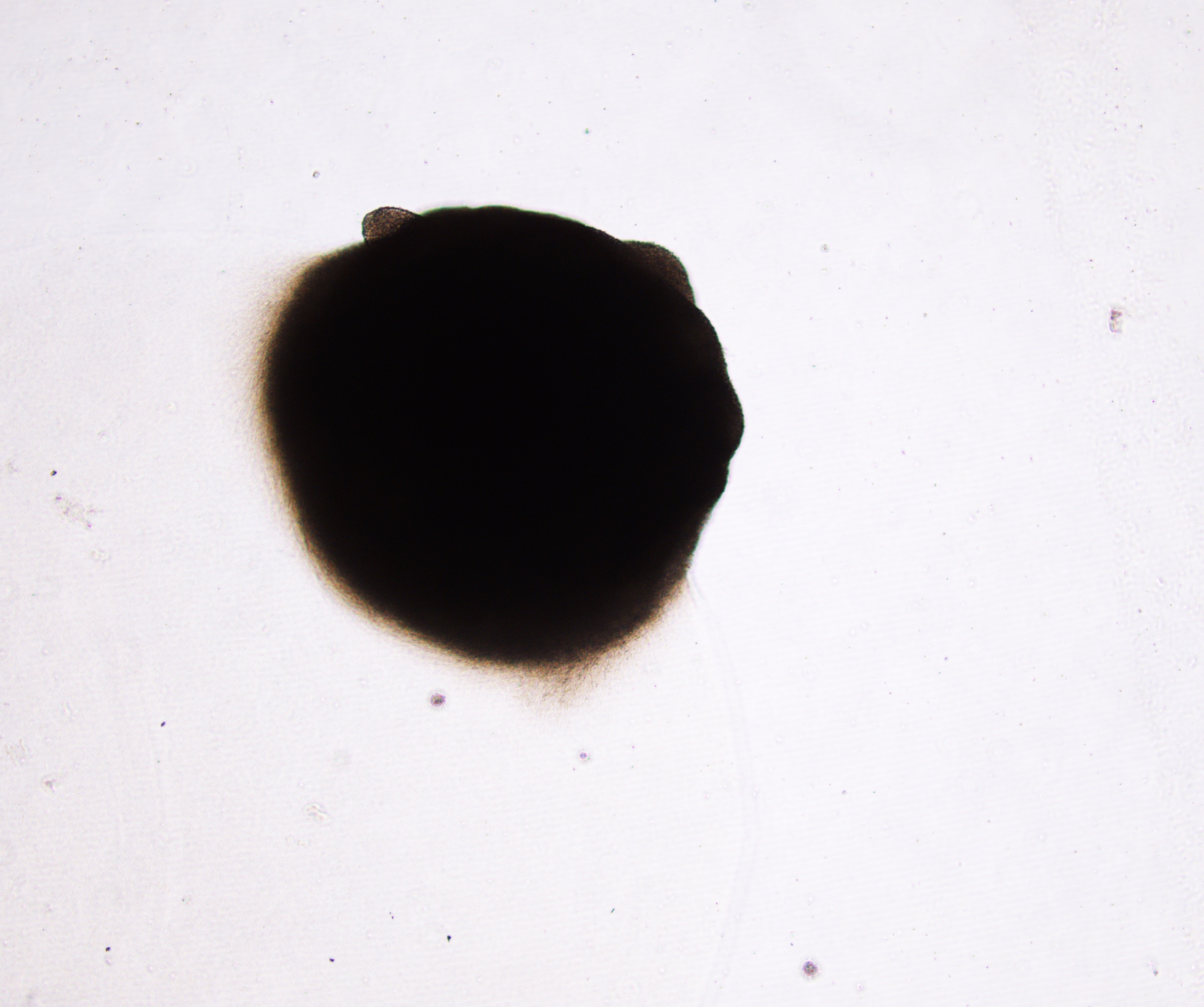

Supplement: Supplementary file 10 — Figure EV2 Source Data [file 44321_2025_302_MOESM10_ESM.zip › Figure EV2/EV2A/Day25_6-6.jpeg]

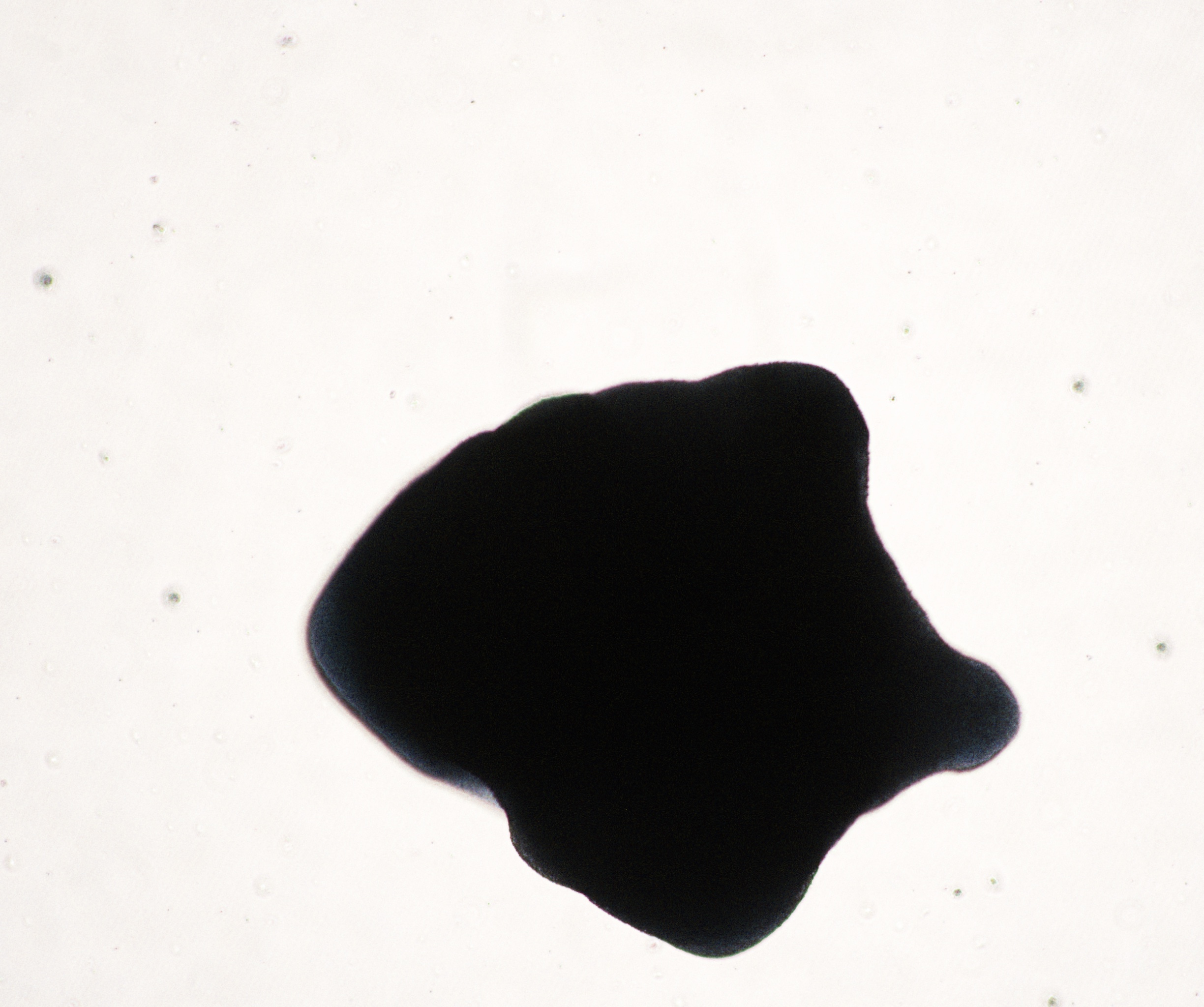

Supplement: Supplementary file 10 — Figure EV2 Source Data [file 44321_2025_302_MOESM10_ESM.zip › Figure EV2/EV2A/Day35_15-4.jpeg]

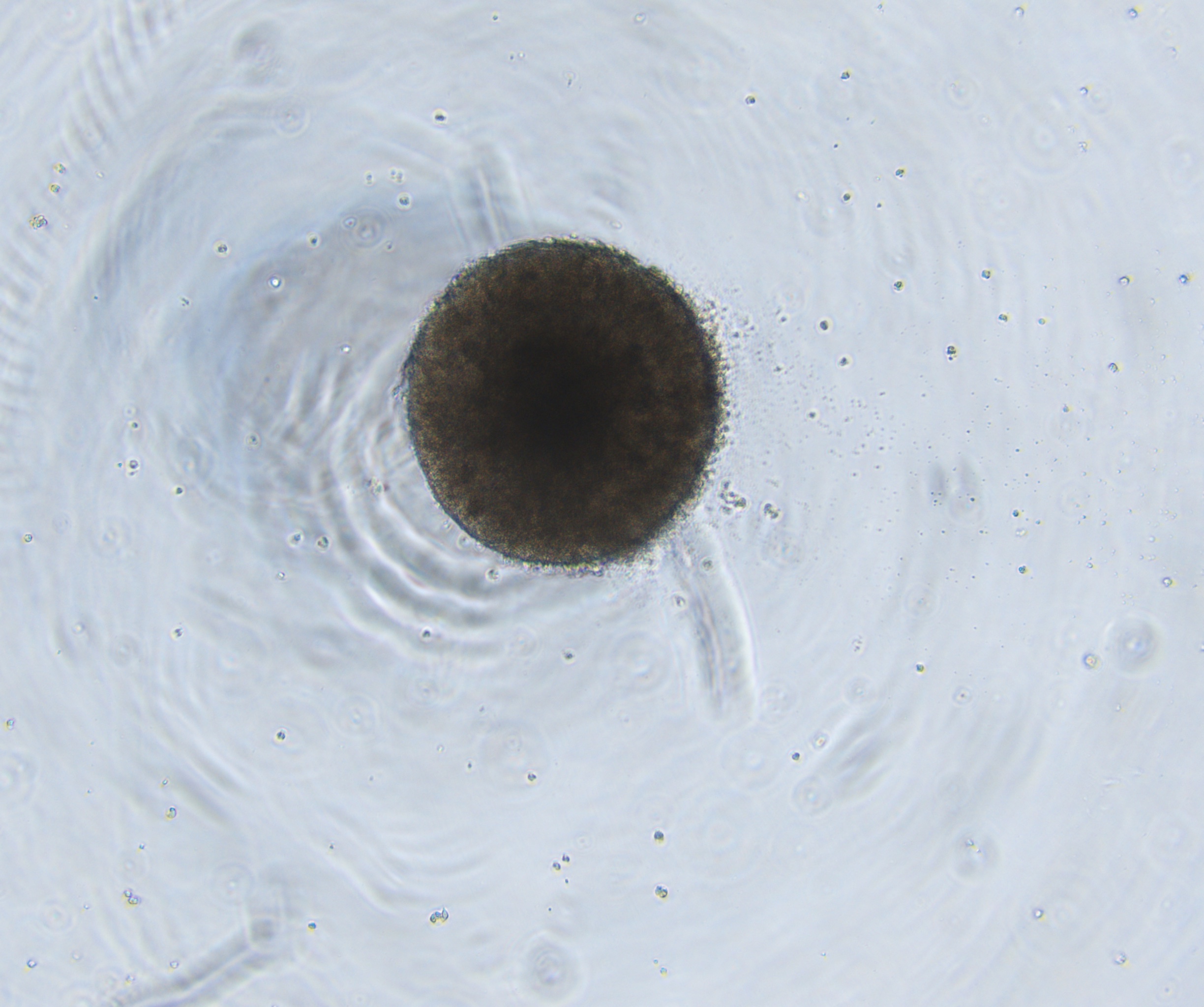

Supplement: Supplementary file 10 — Figure EV2 Source Data [file 44321_2025_302_MOESM10_ESM.zip › Figure EV2/EV2A/Day3_6-6.jpeg]

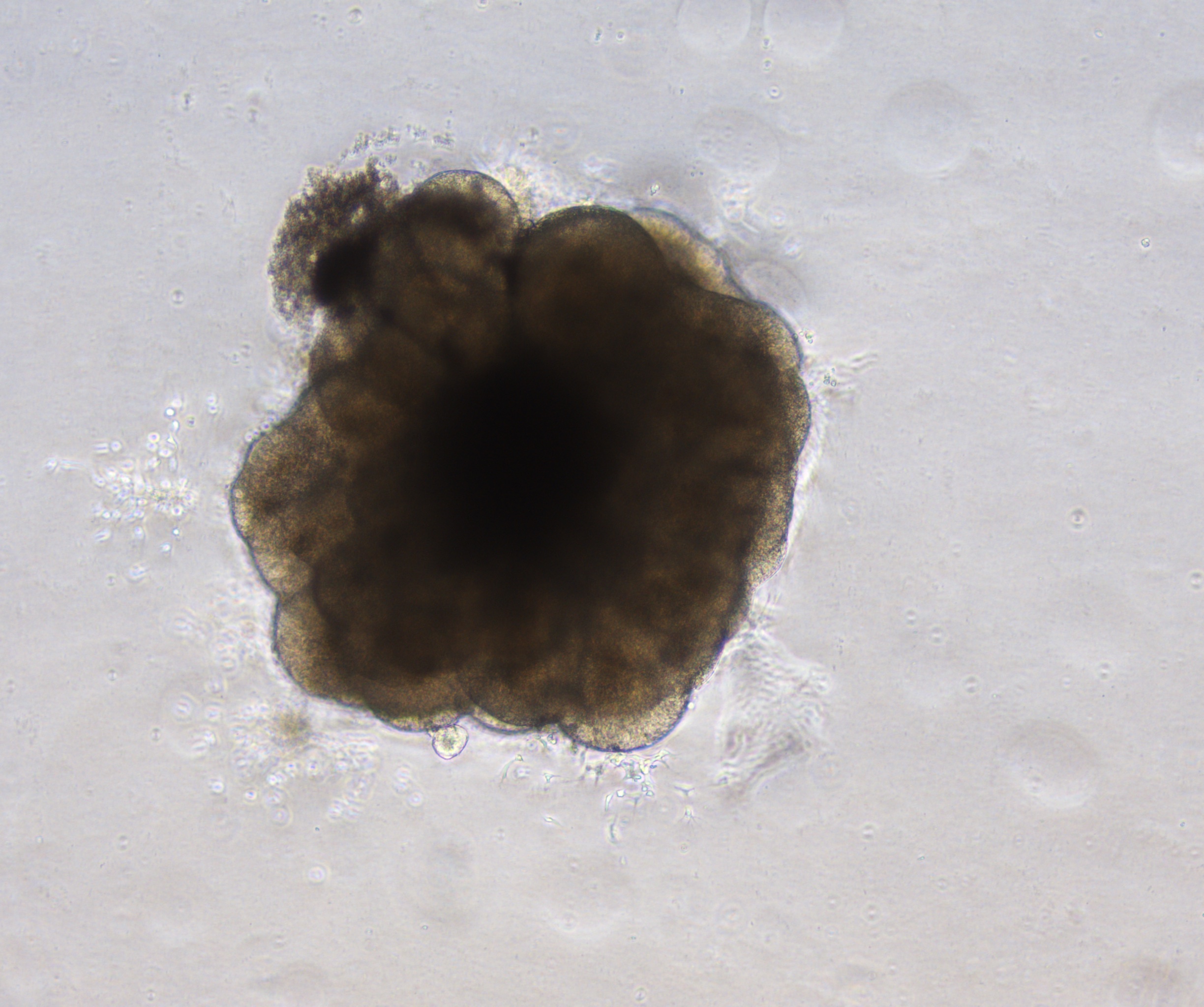

Supplement: Supplementary file 10 — Figure EV2 Source Data [file 44321_2025_302_MOESM10_ESM.zip › Figure EV2/EV2A/Day15_H1.jpeg]

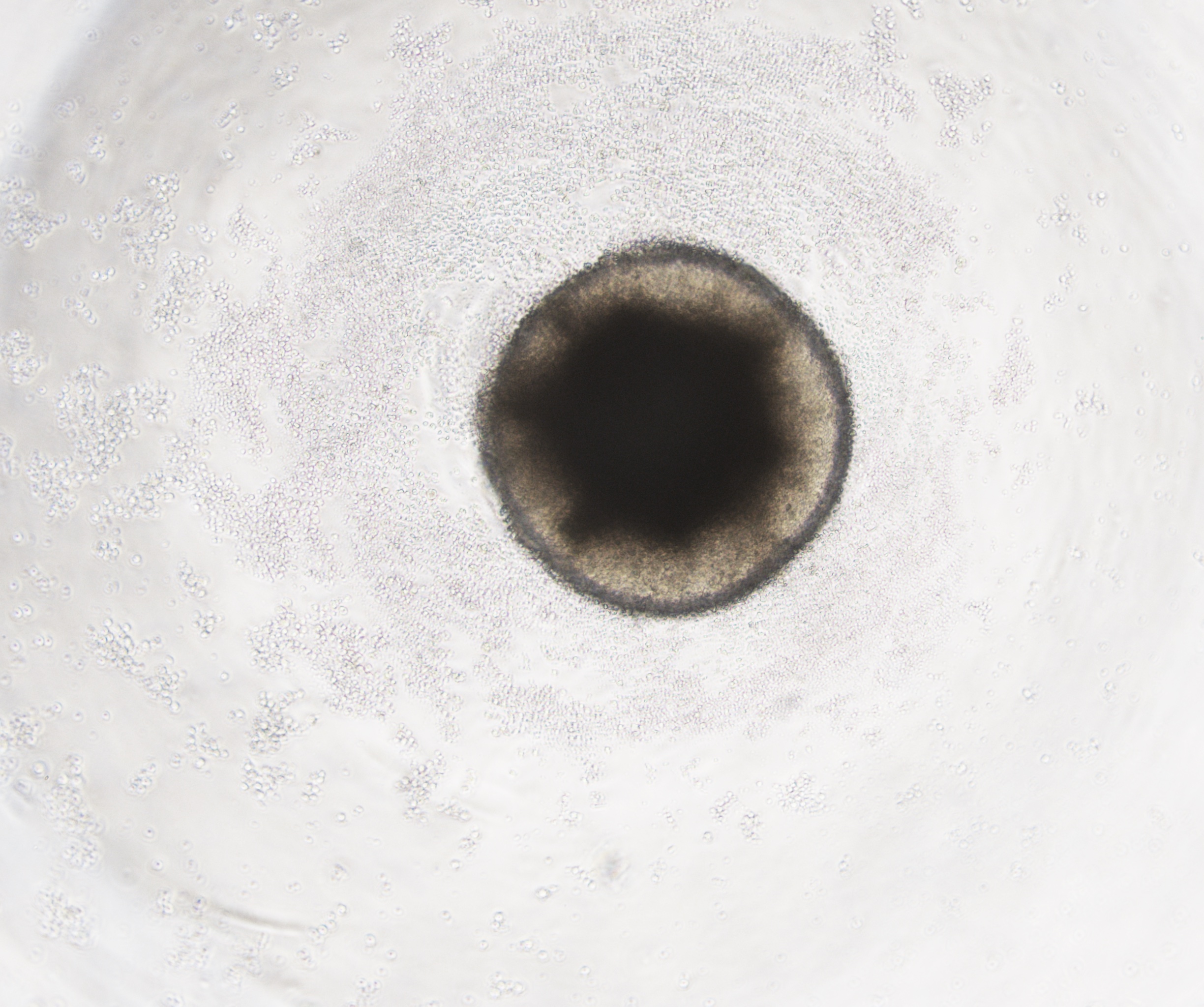

Supplement: Supplementary file 10 — Figure EV2 Source Data [file 44321_2025_302_MOESM10_ESM.zip › Figure EV2/EV2A/Day10_6-6.jpeg]

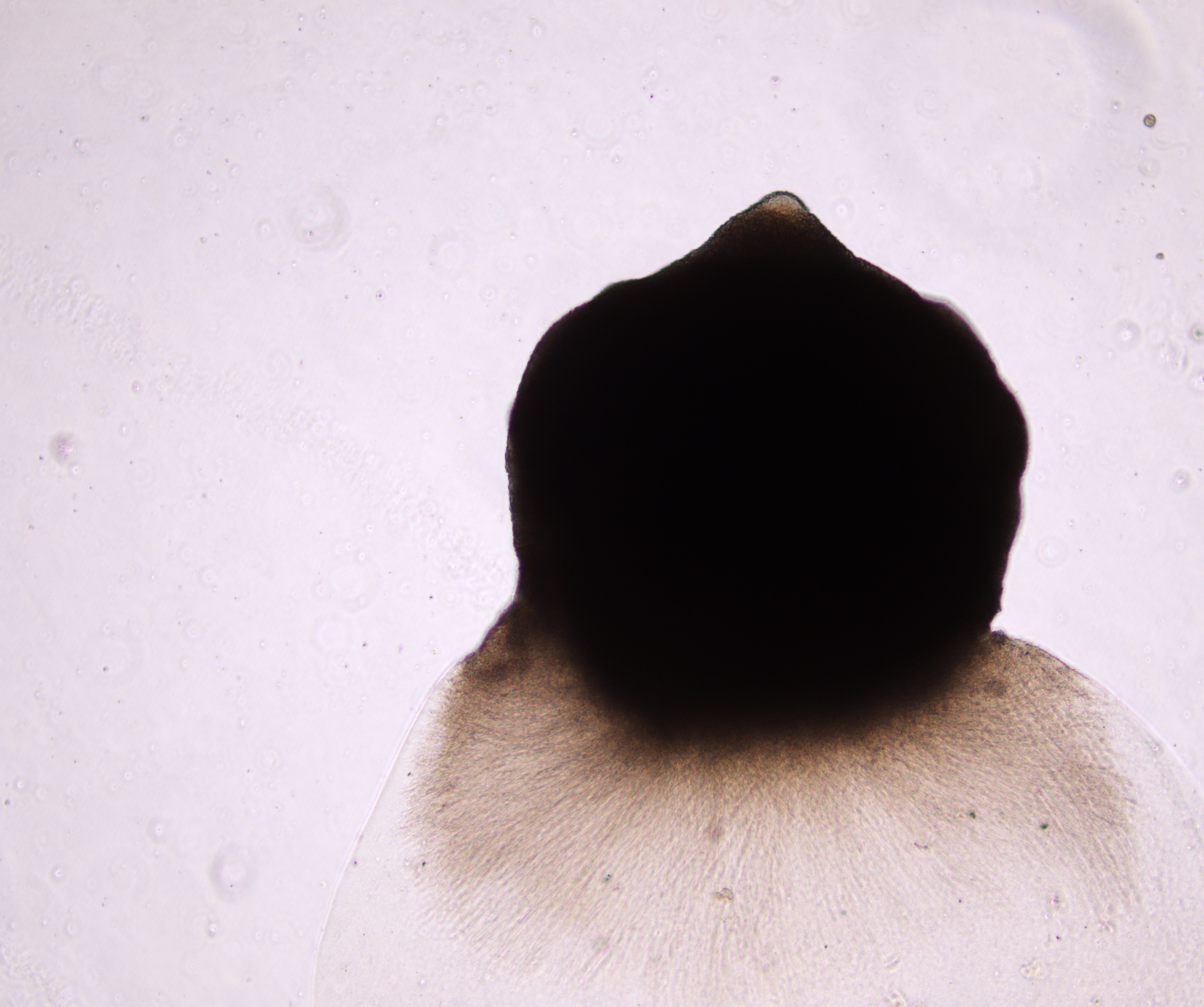

Supplement: Supplementary file 10 — Figure EV2 Source Data [file 44321_2025_302_MOESM10_ESM.zip › Figure EV2/EV2A/Day30_15-4.jpeg]

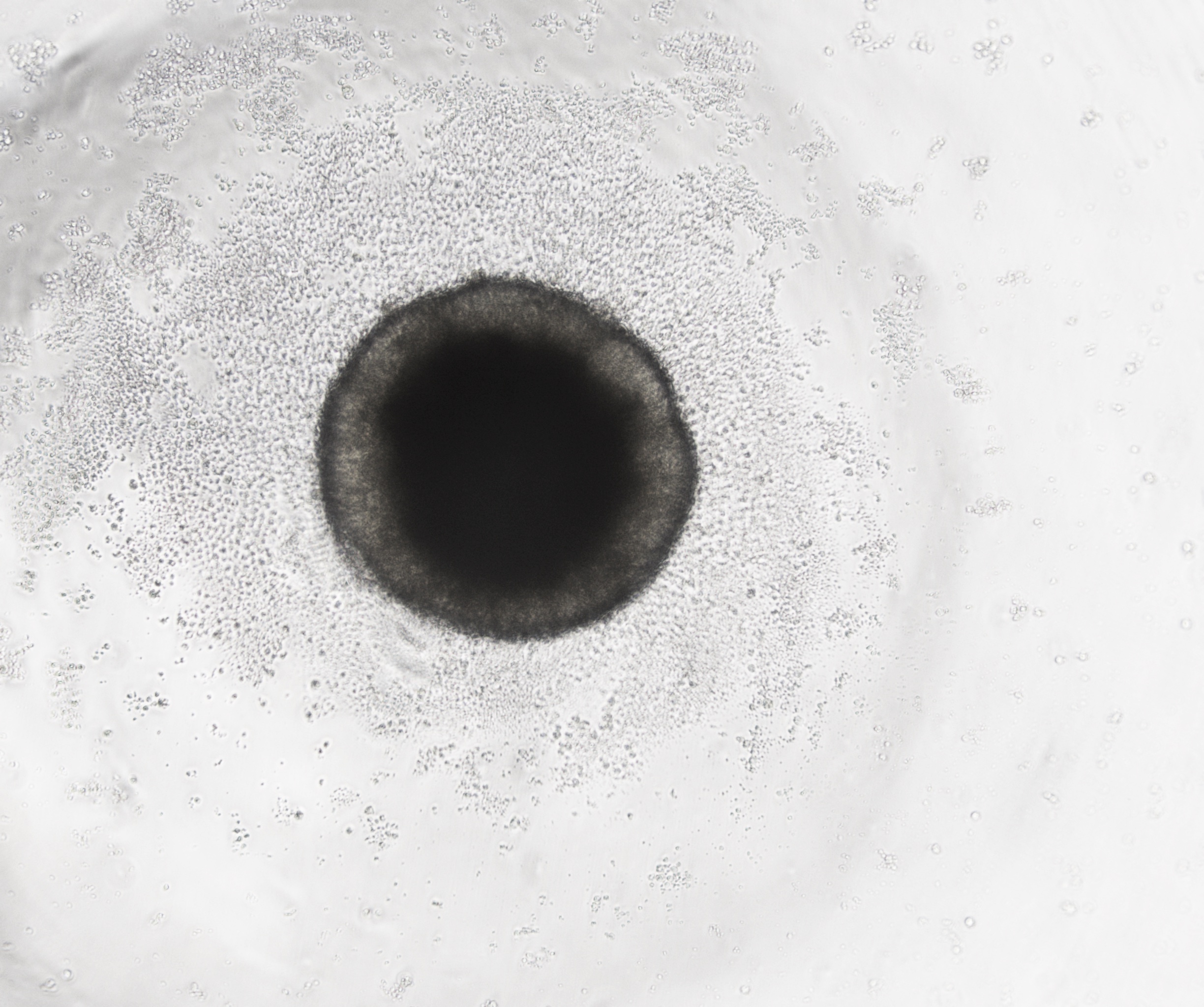

Supplement: Supplementary file 10 — Figure EV2 Source Data [file 44321_2025_302_MOESM10_ESM.zip › Figure EV2/EV2A/Day10_H1.jpeg]

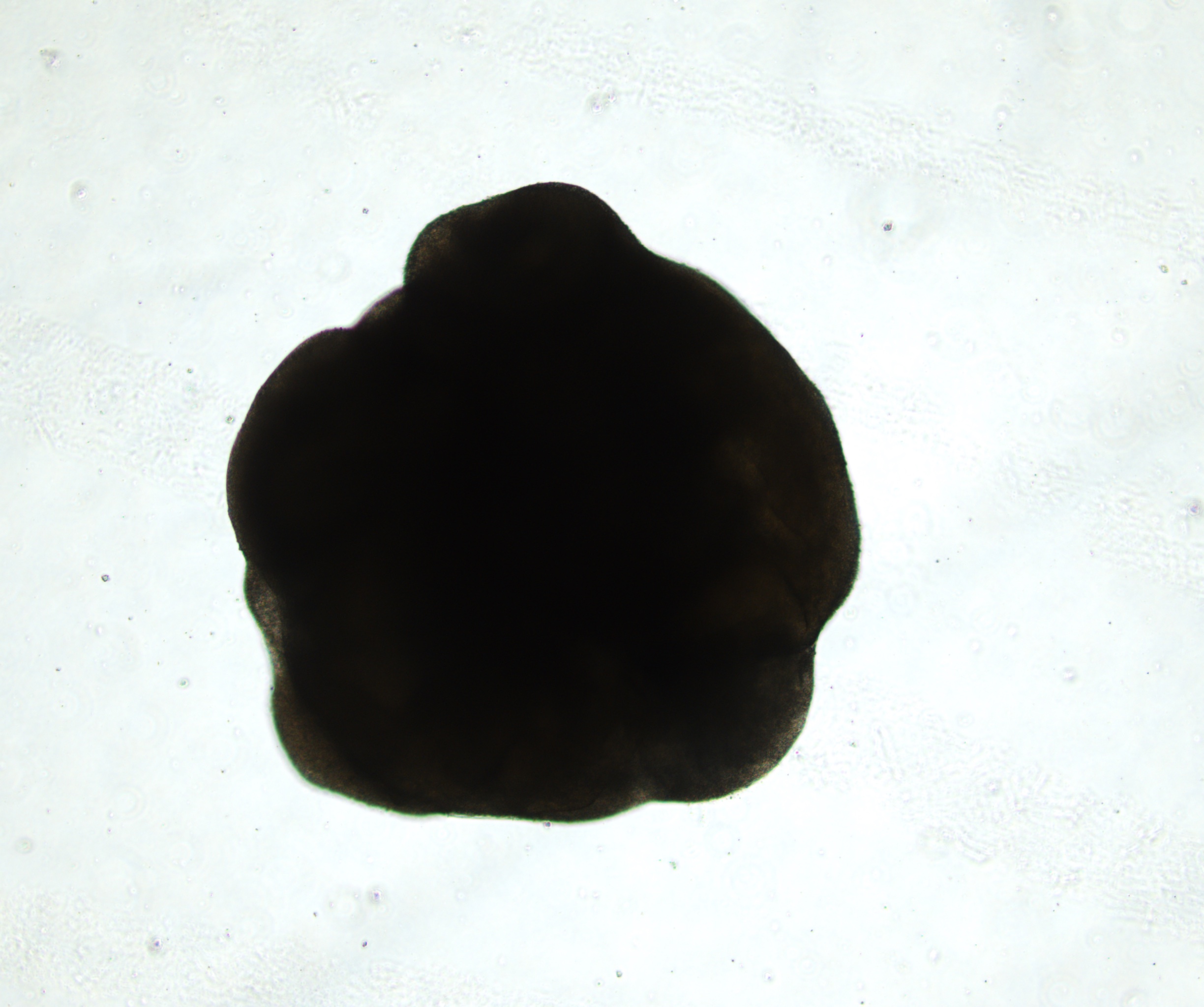

Supplement: Supplementary file 10 — Figure EV2 Source Data [file 44321_2025_302_MOESM10_ESM.zip › Figure EV2/EV2A/Day40_6-6.jpeg]

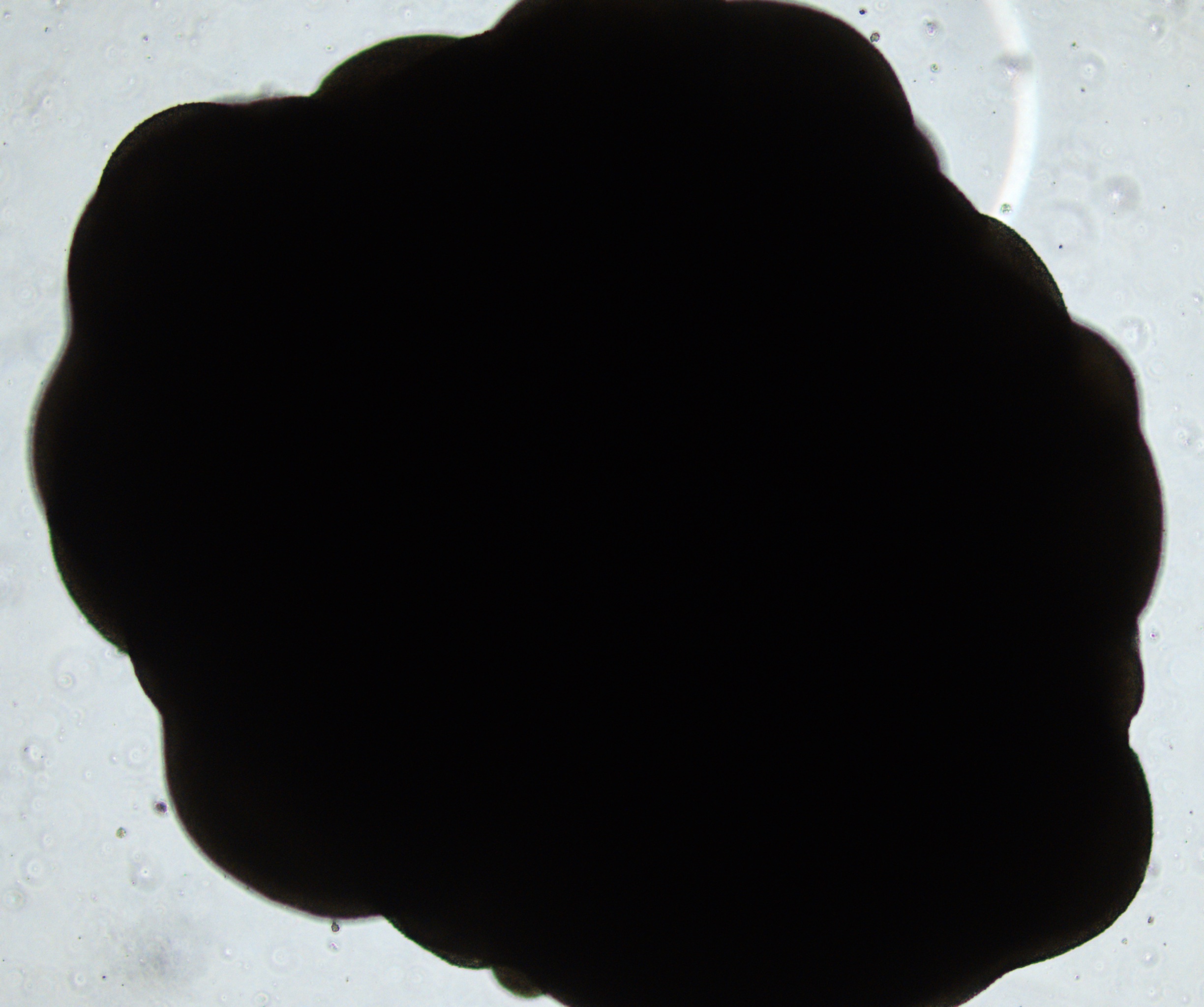

Supplement: Supplementary file 10 — Figure EV2 Source Data [file 44321_2025_302_MOESM10_ESM.zip › Figure EV2/EV2A/Day45_H1.jpeg]

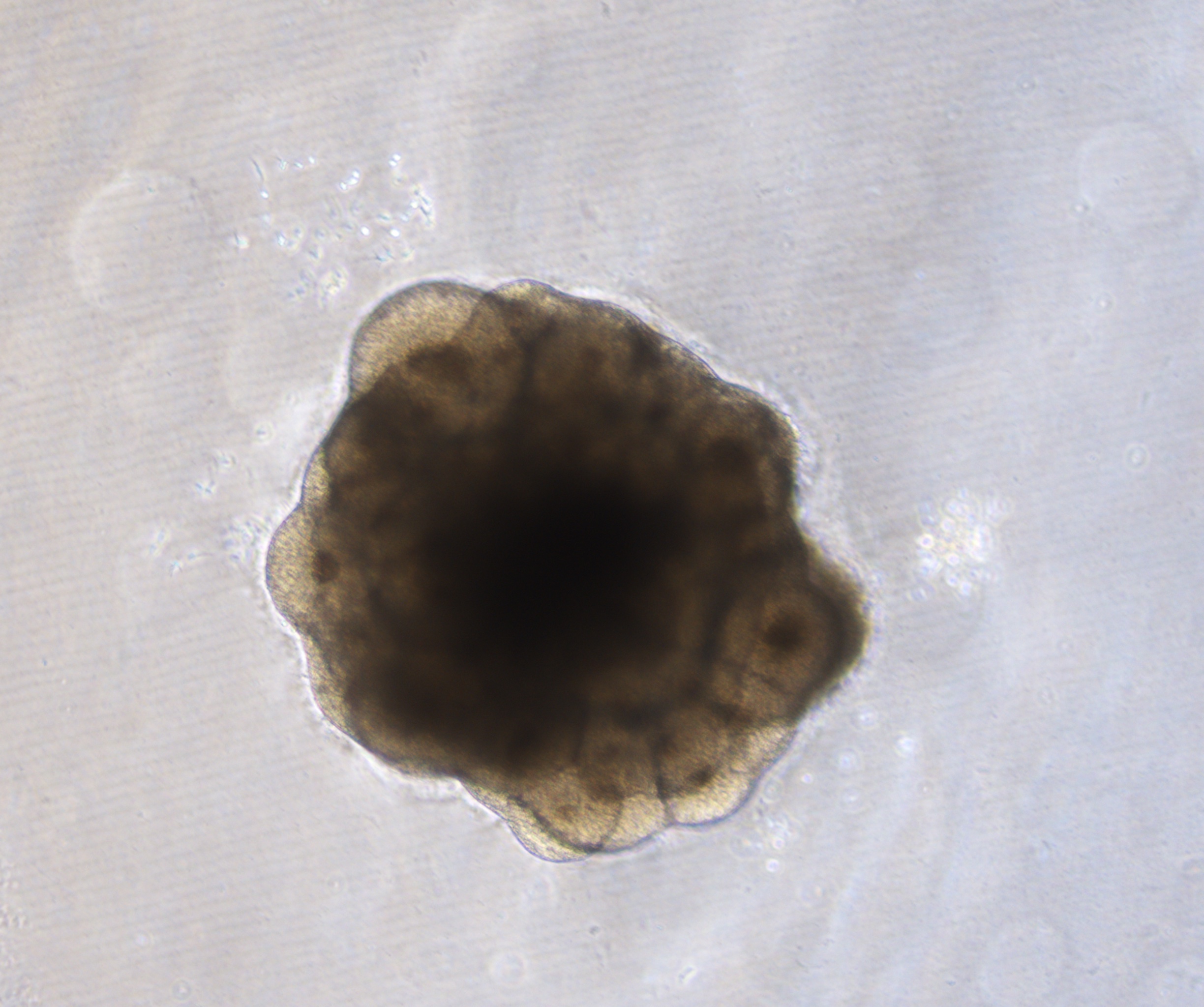

Supplement: Supplementary file 10 — Figure EV2 Source Data [file 44321_2025_302_MOESM10_ESM.zip › Figure EV2/EV2A/Day15_6-6.jpeg]

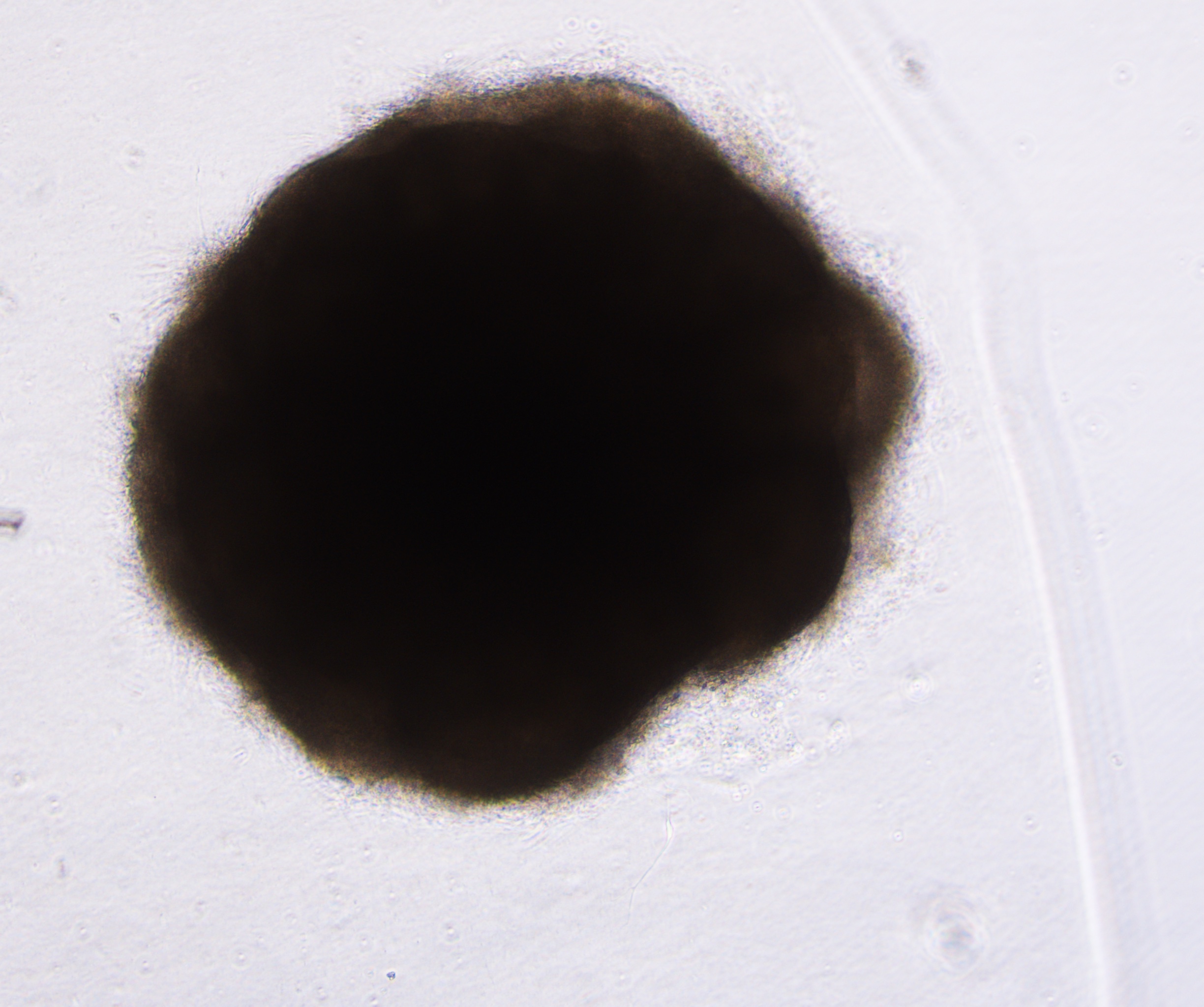

Supplement: Supplementary file 10 — Figure EV2 Source Data [file 44321_2025_302_MOESM10_ESM.zip › Figure EV2/EV2A/Day20_6-6.jpeg]

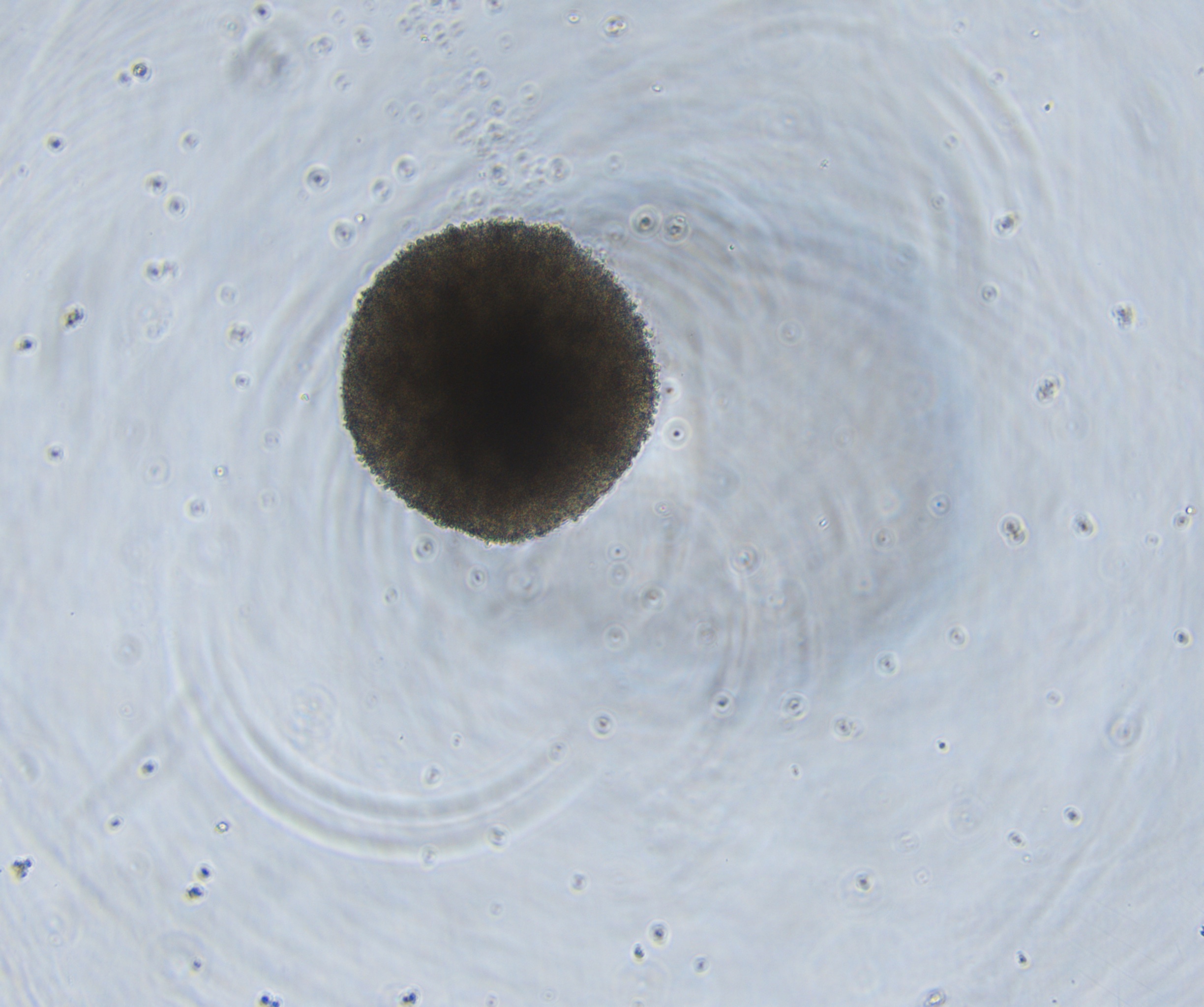

Supplement: Supplementary file 10 — Figure EV2 Source Data [file 44321_2025_302_MOESM10_ESM.zip › Figure EV2/EV2A/Day3_15-4.jpeg]

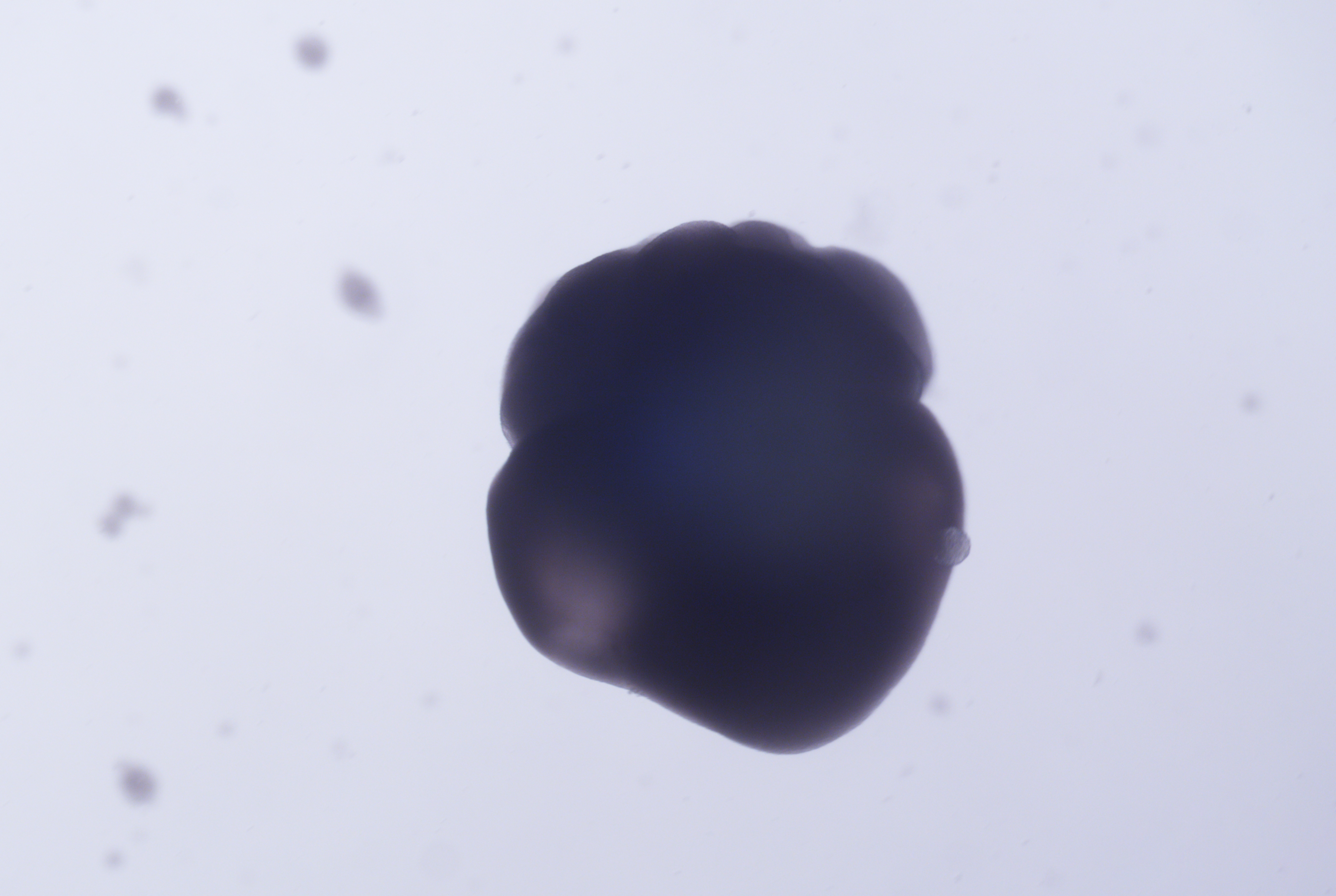

Supplement: Supplementary file 10 — Figure EV2 Source Data [file 44321_2025_302_MOESM10_ESM.zip › Figure EV2/EV2A/Day60_15-4.jpeg]

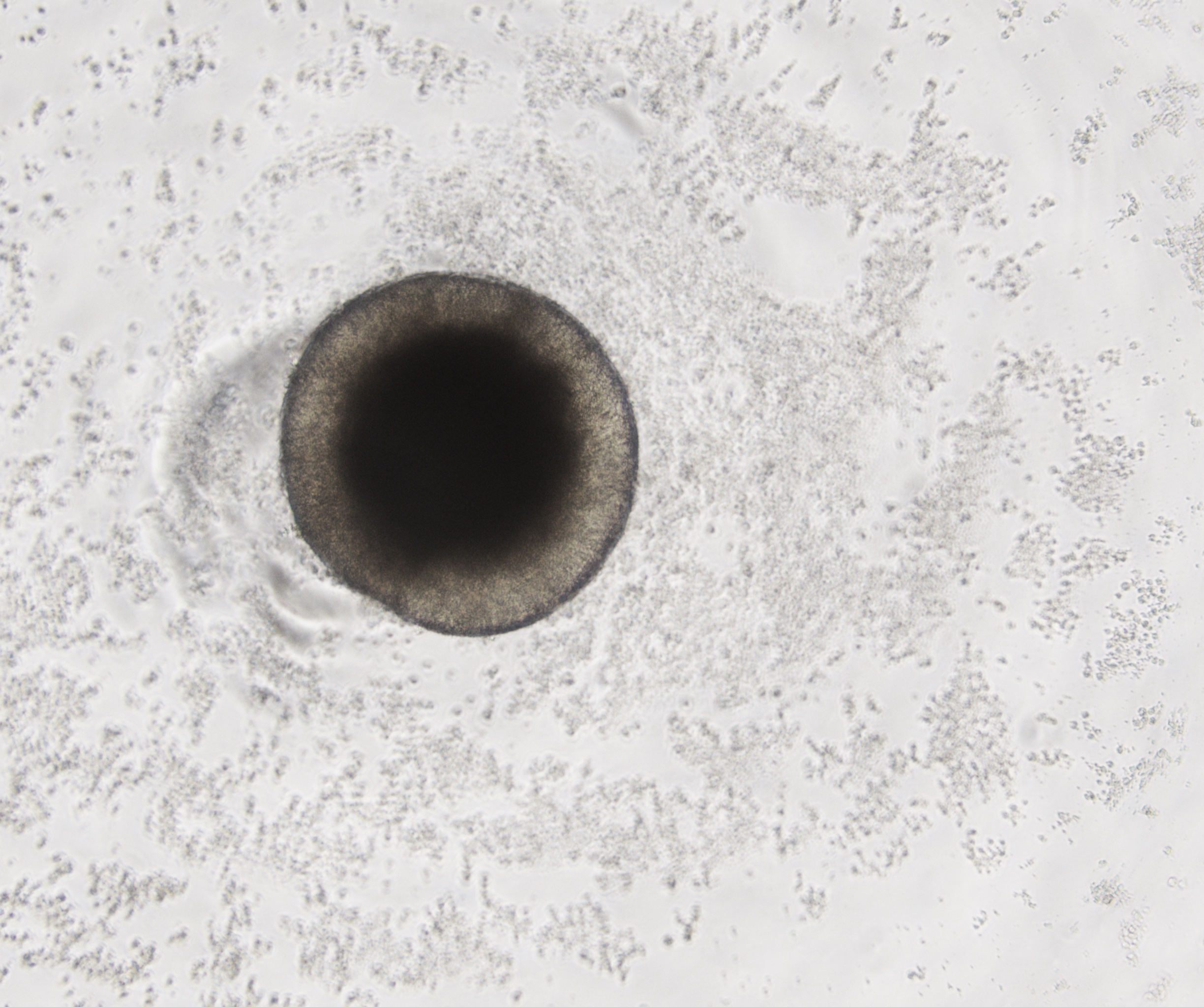

Supplement: Supplementary file 10 — Figure EV2 Source Data [file 44321_2025_302_MOESM10_ESM.zip › Figure EV2/EV2A/Day10_15-4.jpeg]

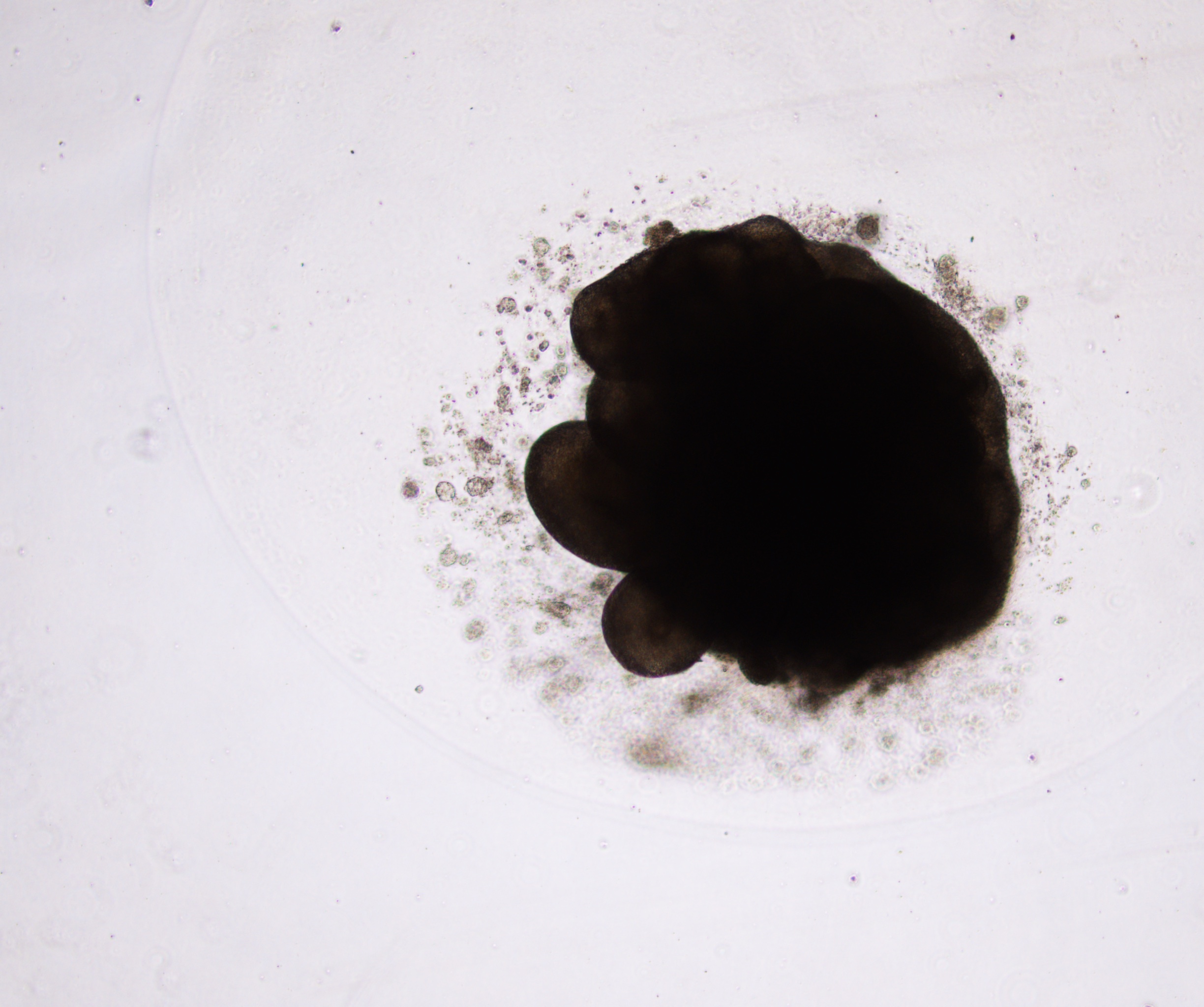

Supplement: Supplementary file 10 — Figure EV2 Source Data [file 44321_2025_302_MOESM10_ESM.zip › Figure EV2/EV2A/Day25_H1.jpeg]

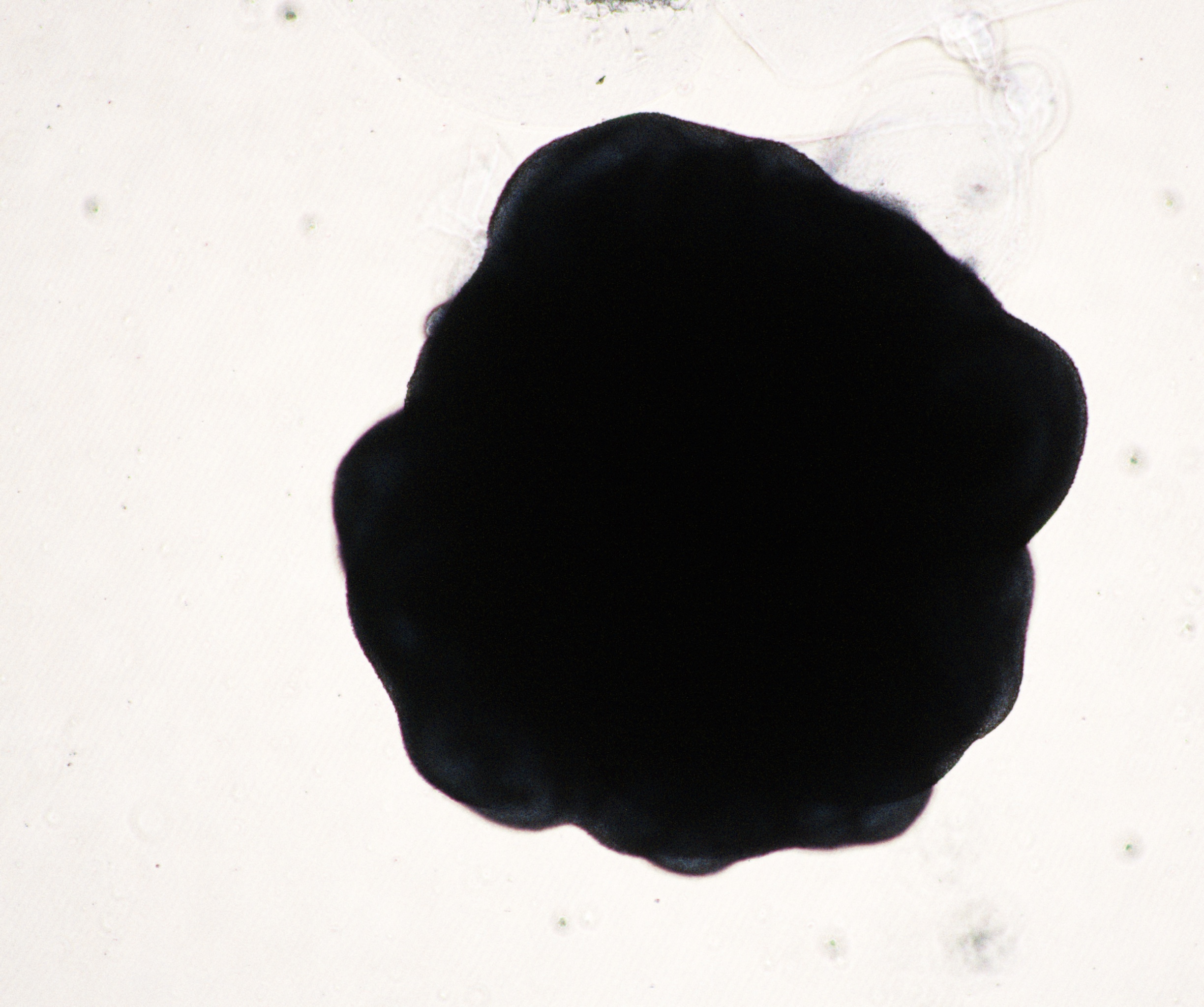

Supplement: Supplementary file 10 — Figure EV2 Source Data [file 44321_2025_302_MOESM10_ESM.zip › Figure EV2/EV2A/Day35_H1.jpeg]

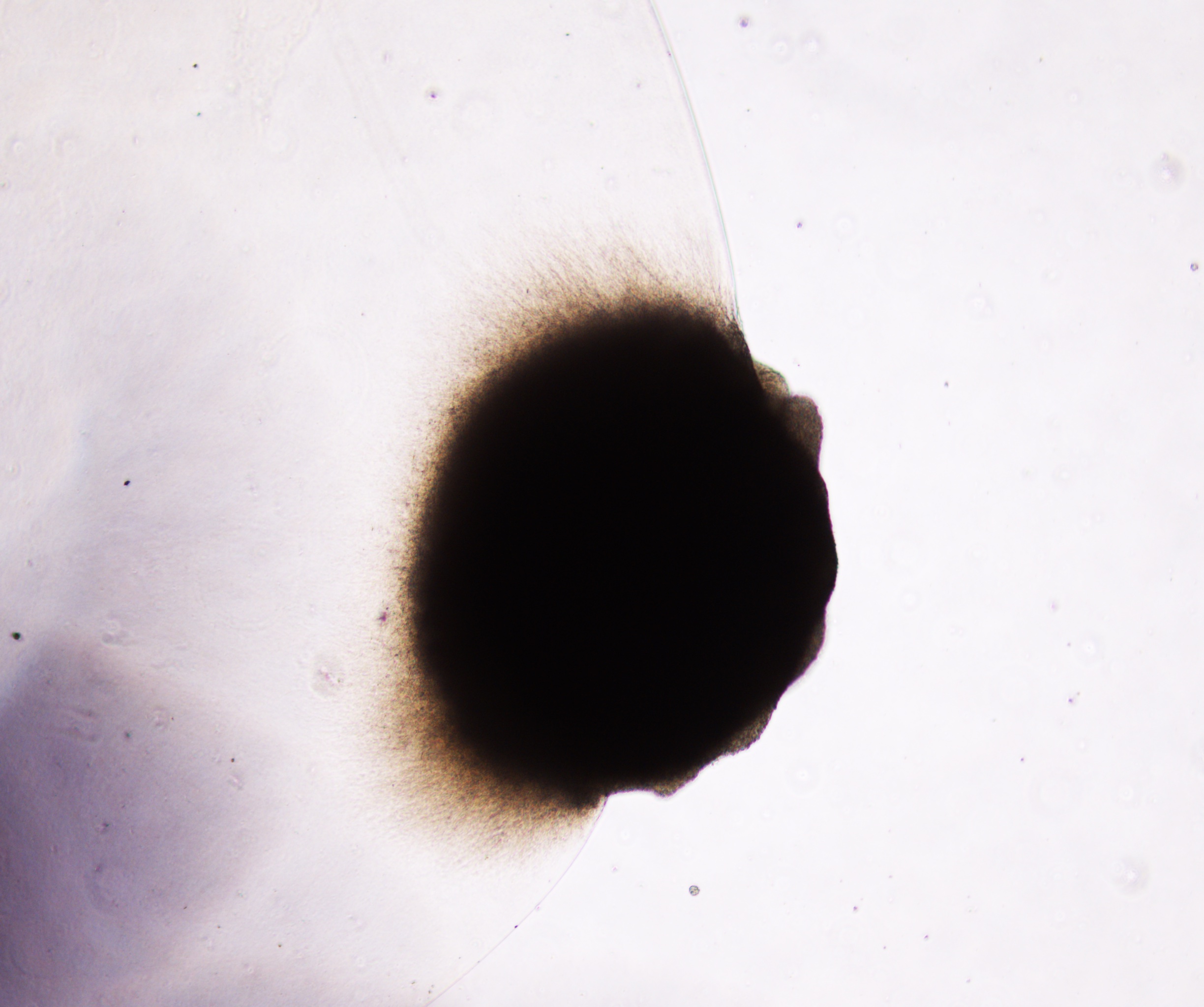

Supplement: Supplementary file 10 — Figure EV2 Source Data [file 44321_2025_302_MOESM10_ESM.zip › Figure EV2/EV2A/Day25_15-4.jpeg]

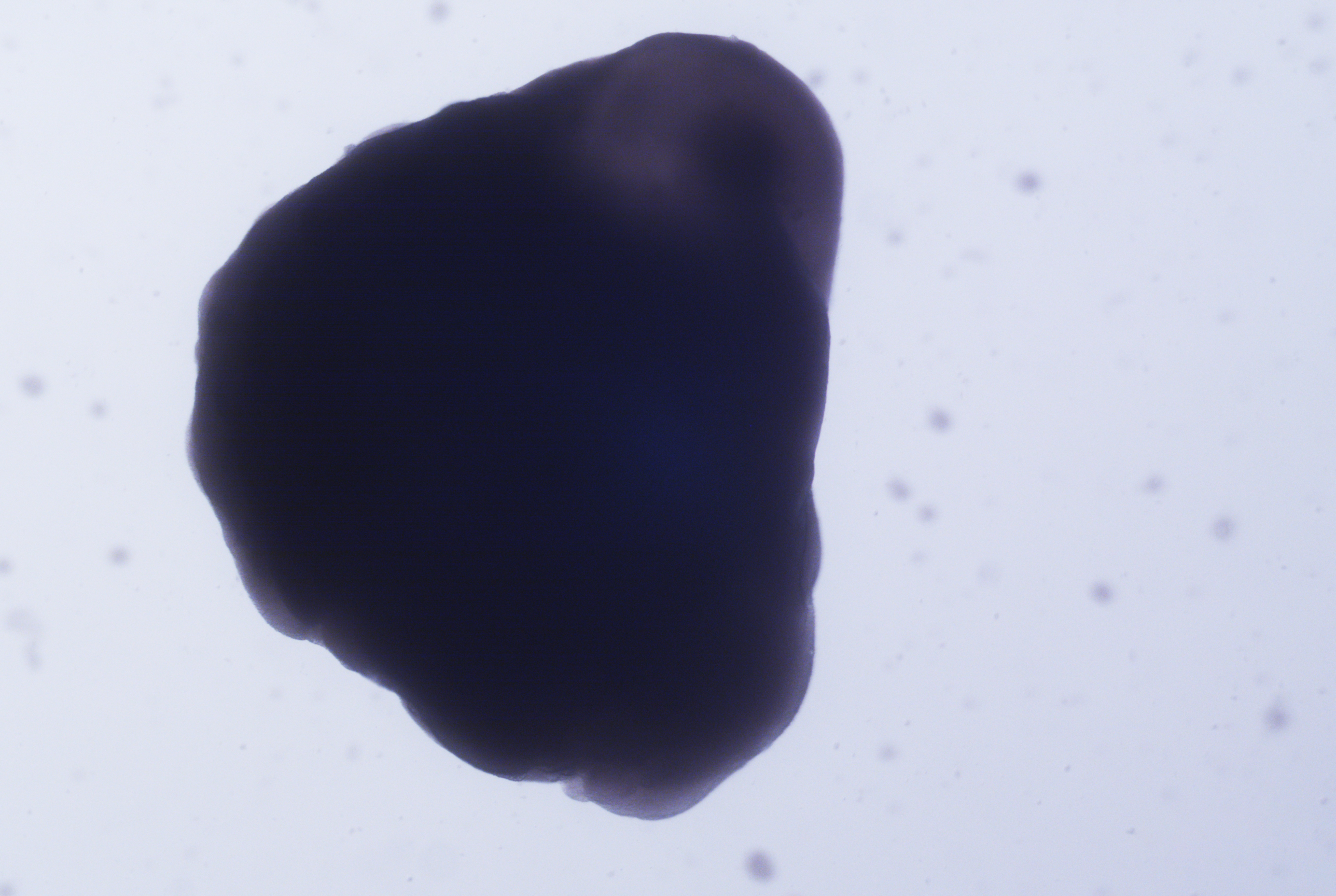

Supplement: Supplementary file 10 — Figure EV2 Source Data [file 44321_2025_302_MOESM10_ESM.zip › Figure EV2/EV2A/Day60_H1.jpeg]

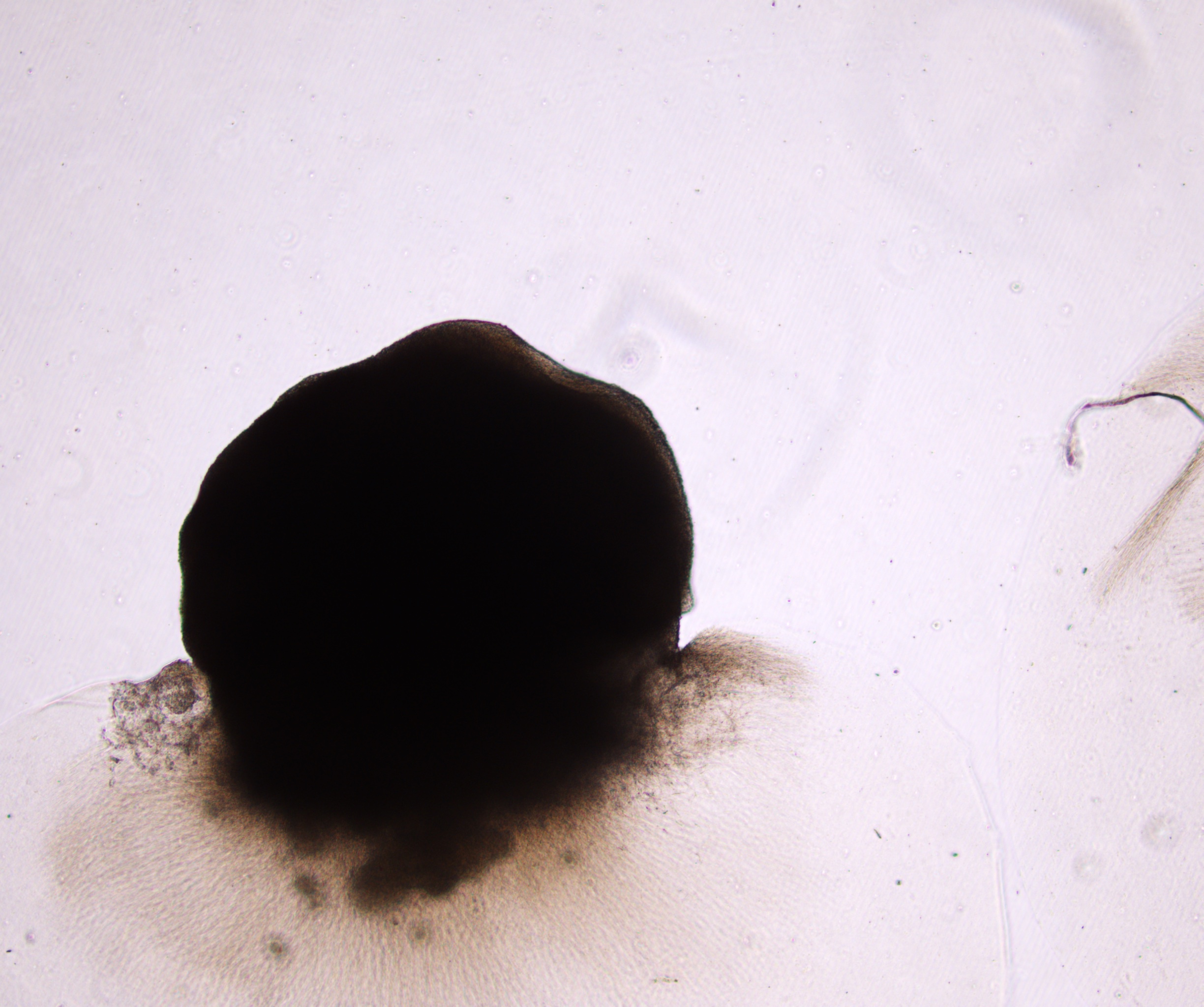

Supplement: Supplementary file 10 — Figure EV2 Source Data [file 44321_2025_302_MOESM10_ESM.zip › Figure EV2/EV2A/Day30_6-6.jpeg]

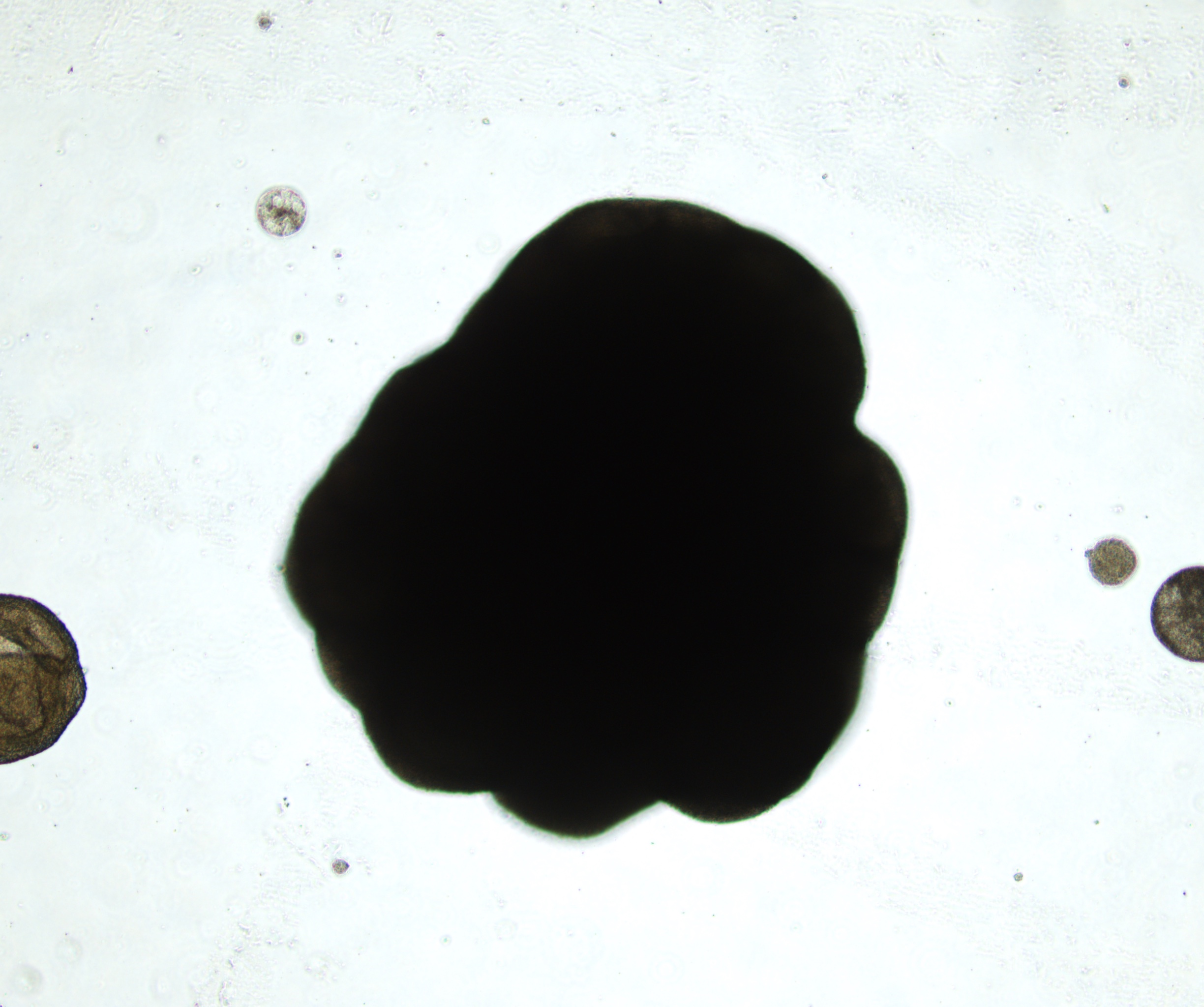

Supplement: Supplementary file 10 — Figure EV2 Source Data [file 44321_2025_302_MOESM10_ESM.zip › Figure EV2/EV2A/Day40_15-4.jpeg]

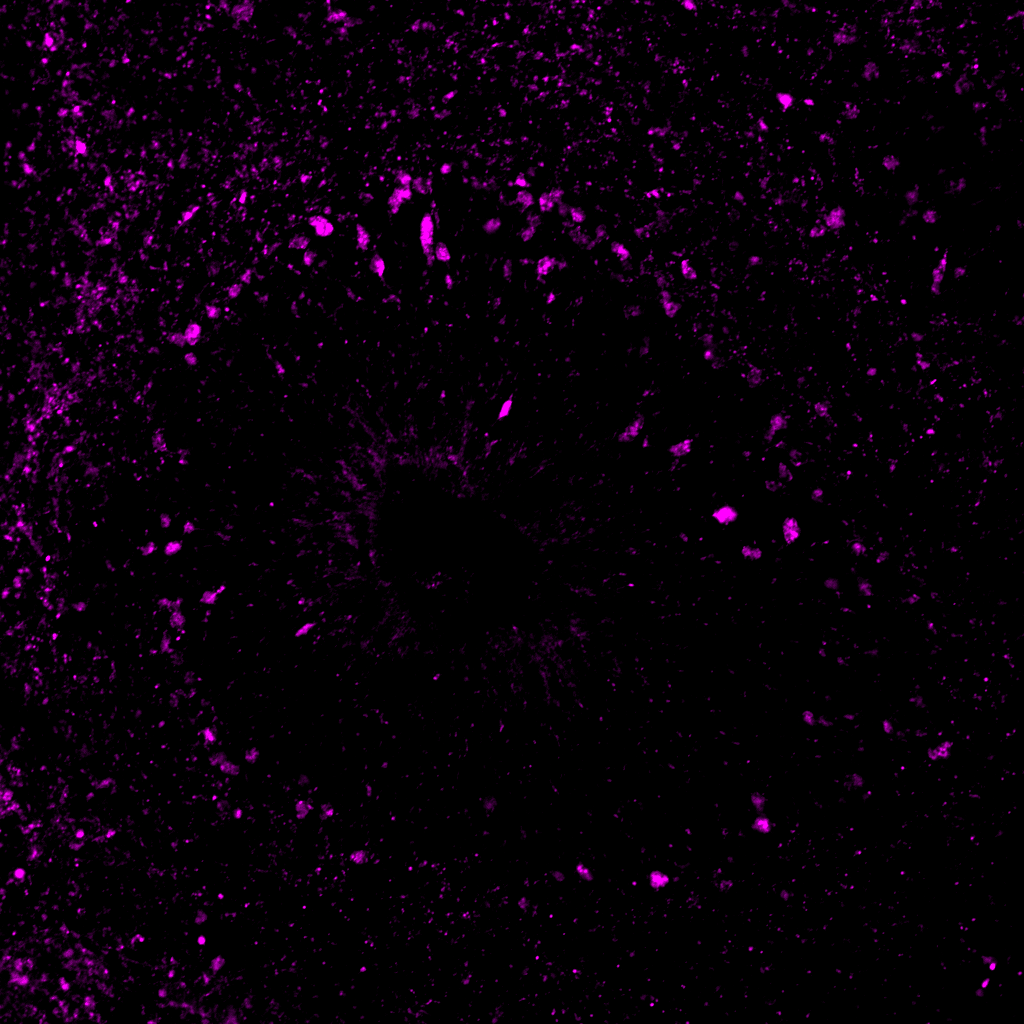

Supplement: Supplementary file 10 — Figure EV2 Source Data [file 44321_2025_302_MOESM10_ESM.zip › Figure EV2/EV2G/H1-caspase3.tif]

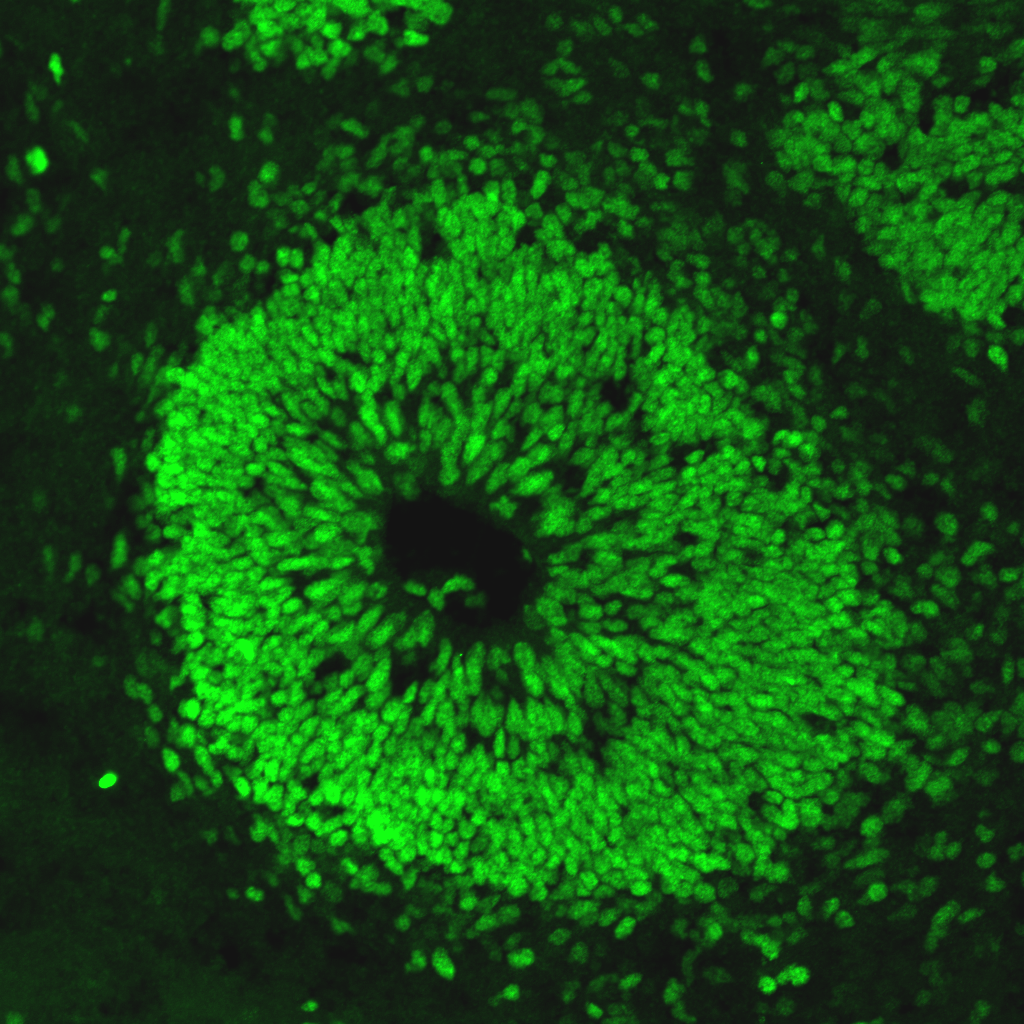

Supplement: Supplementary file 10 — Figure EV2 Source Data [file 44321_2025_302_MOESM10_ESM.zip › Figure EV2/EV2G/H1-PAX6.tif]

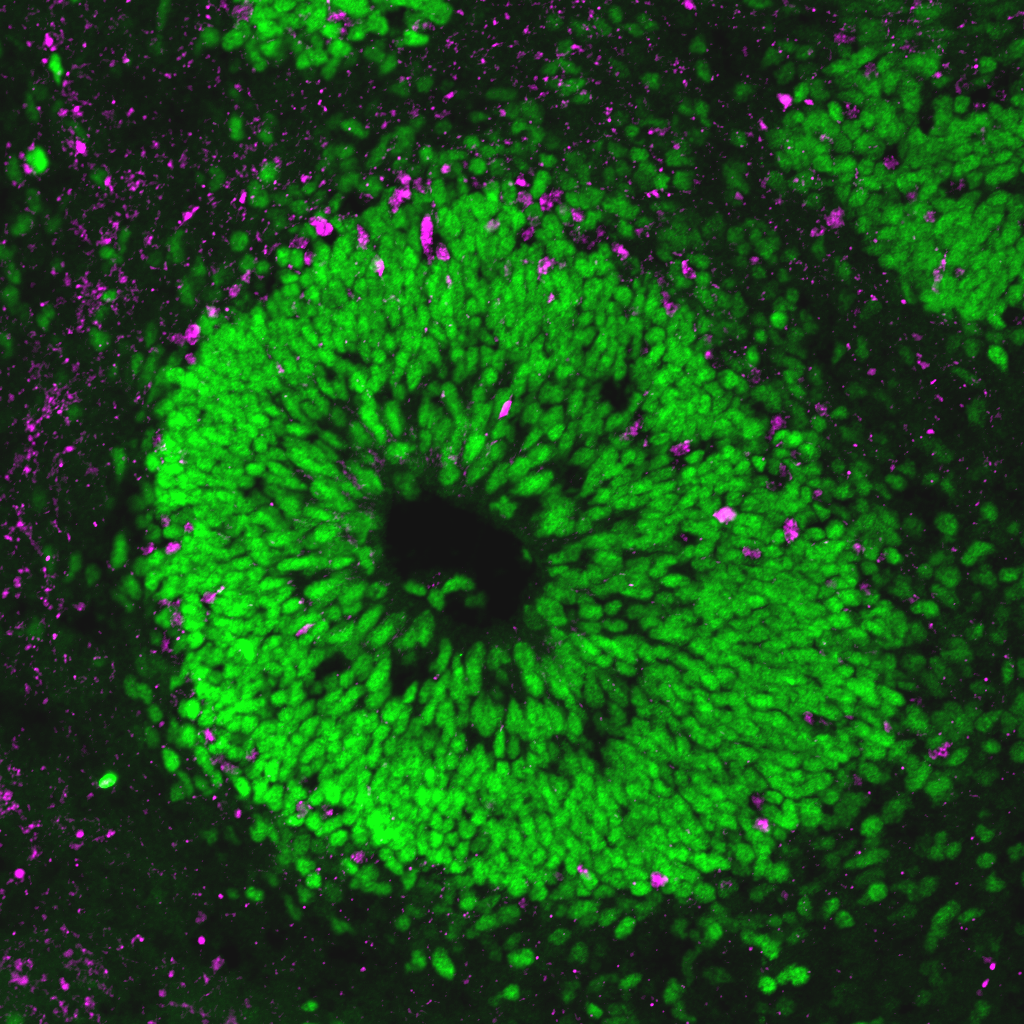

Supplement: Supplementary file 10 — Figure EV2 Source Data [file 44321_2025_302_MOESM10_ESM.zip › Figure EV2/EV2G/H1-merge.tif]

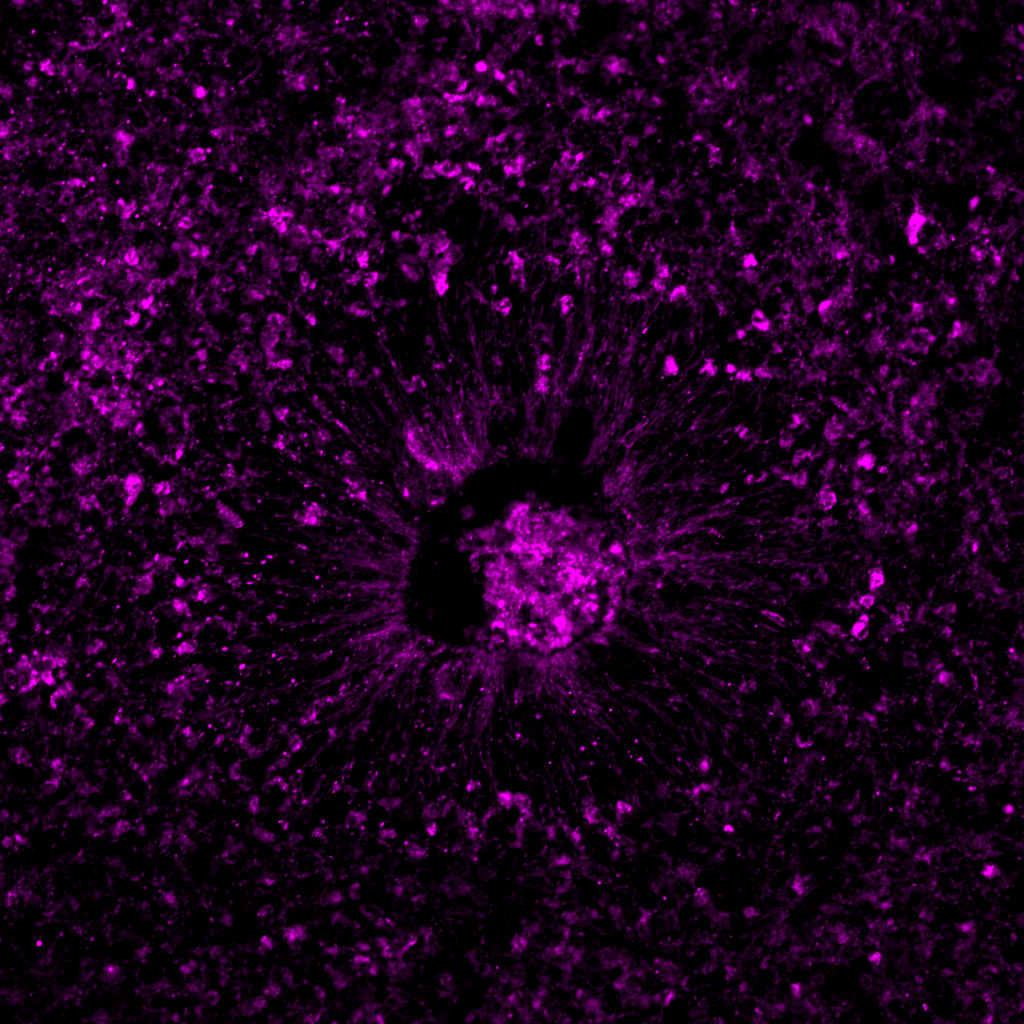

Supplement: Supplementary file 10 — Figure EV2 Source Data [file 44321_2025_302_MOESM10_ESM.zip › Figure EV2/EV2G/#6-6-caspase3.tif]

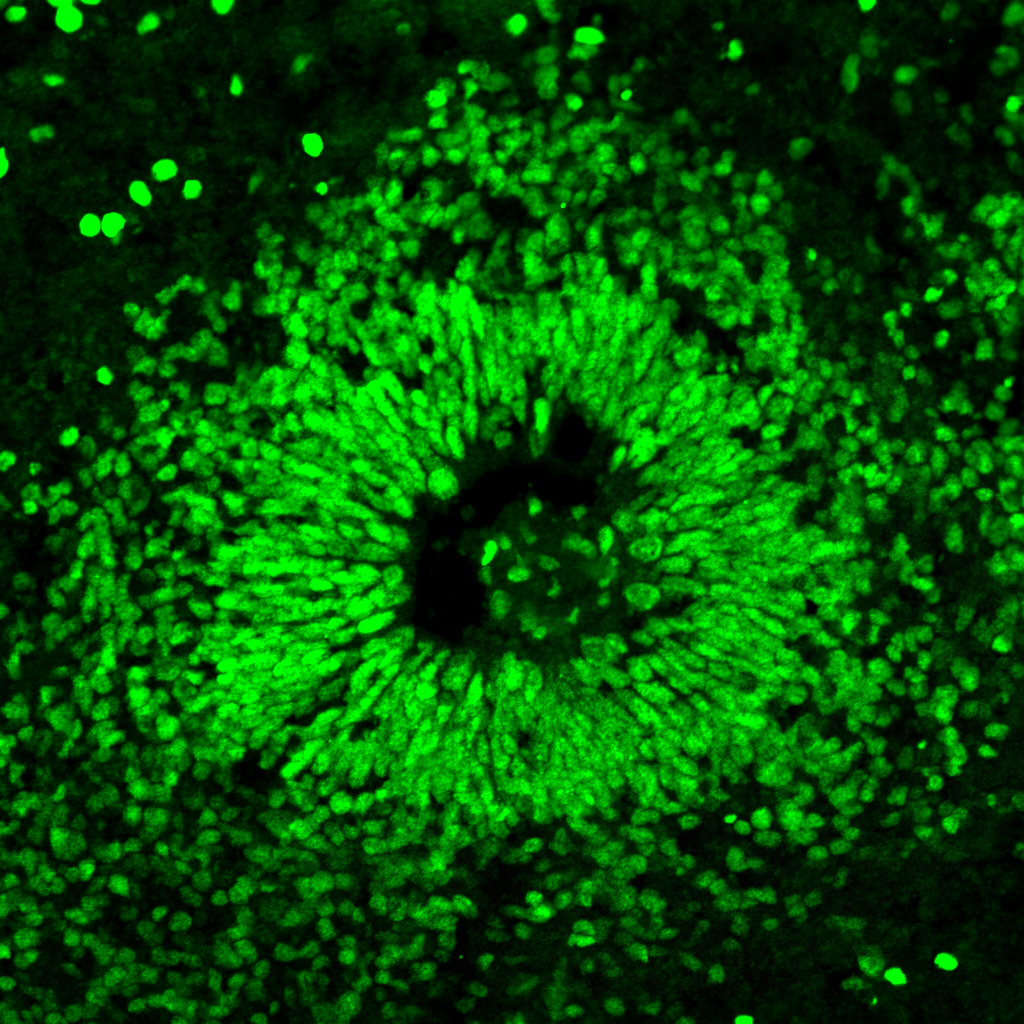

Supplement: Supplementary file 10 — Figure EV2 Source Data [file 44321_2025_302_MOESM10_ESM.zip › Figure EV2/EV2G/#6-6-PAX6.tif]

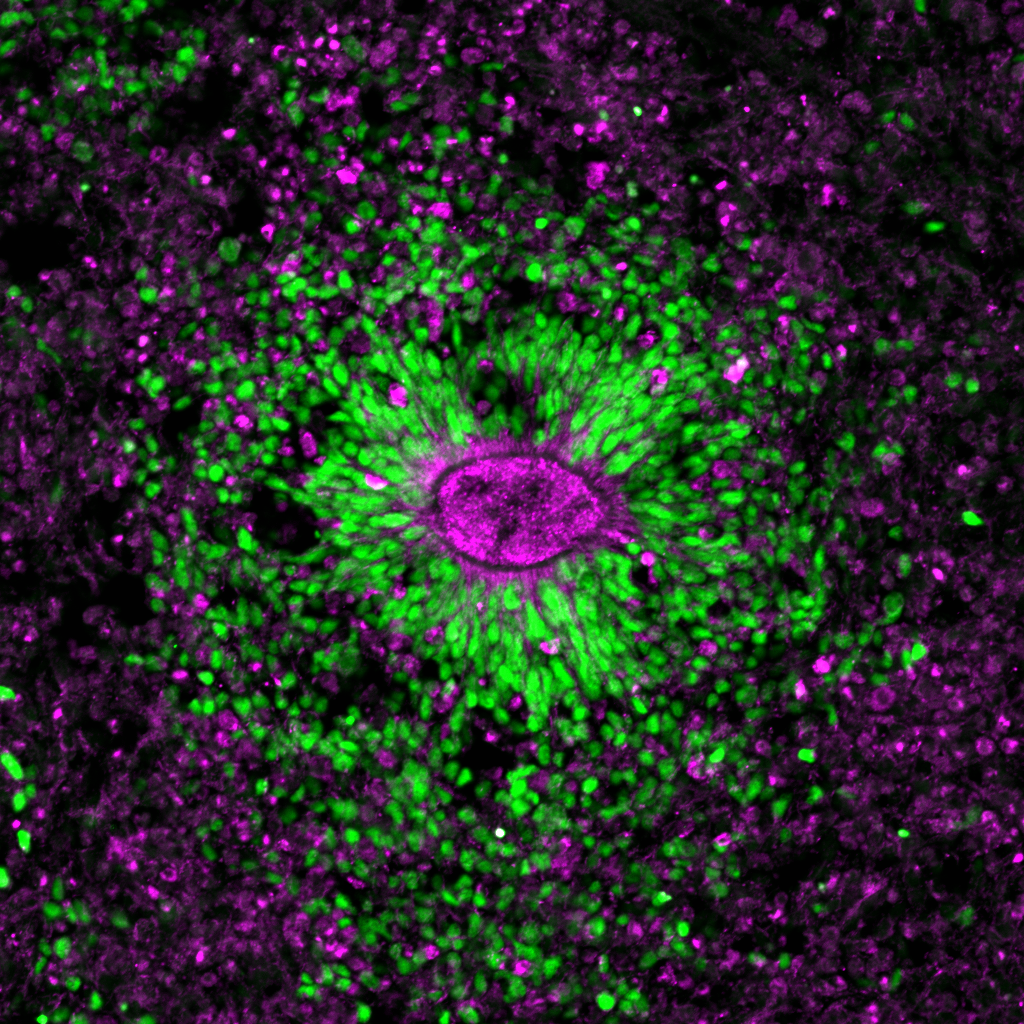

Supplement: Supplementary file 10 — Figure EV2 Source Data [file 44321_2025_302_MOESM10_ESM.zip › Figure EV2/EV2G/#15-4-merge.tif]

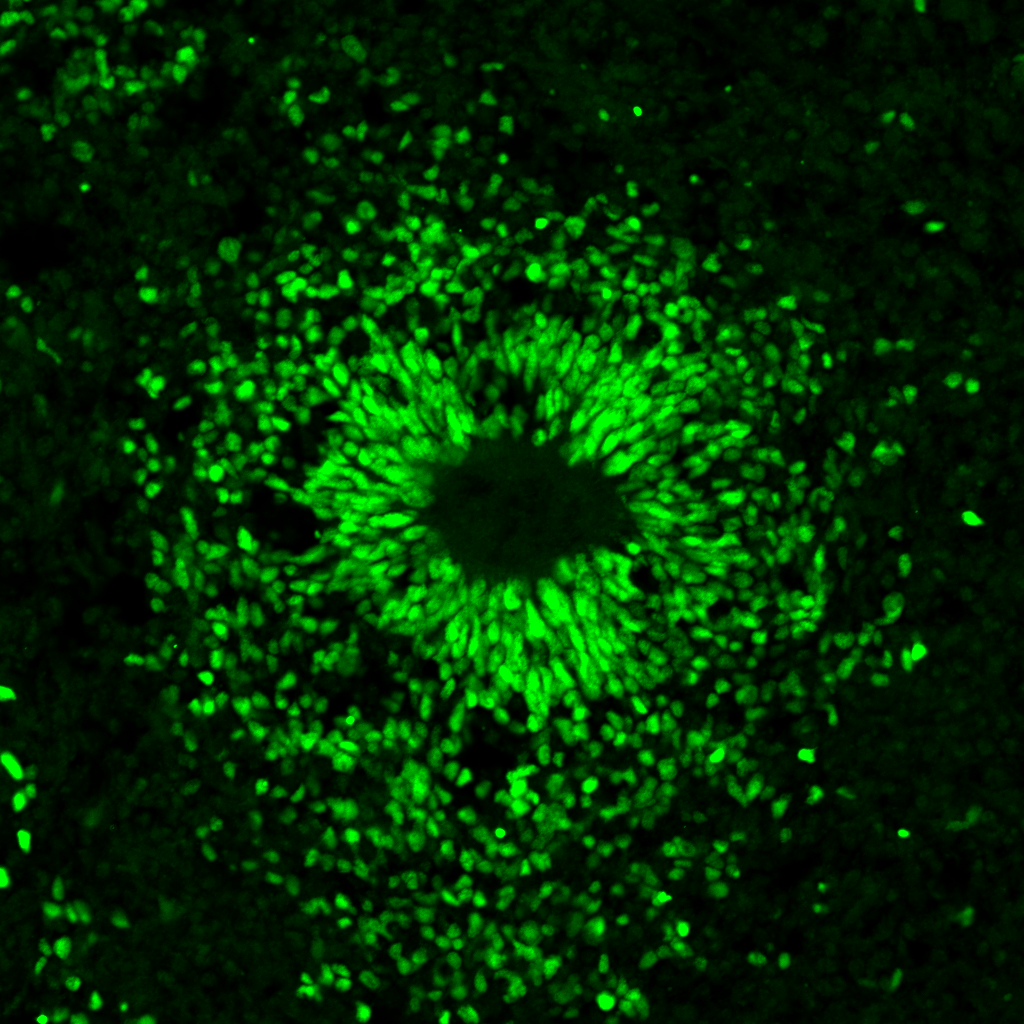

Supplement: Supplementary file 10 — Figure EV2 Source Data [file 44321_2025_302_MOESM10_ESM.zip › Figure EV2/EV2G/#15-4-PAX6.tif]

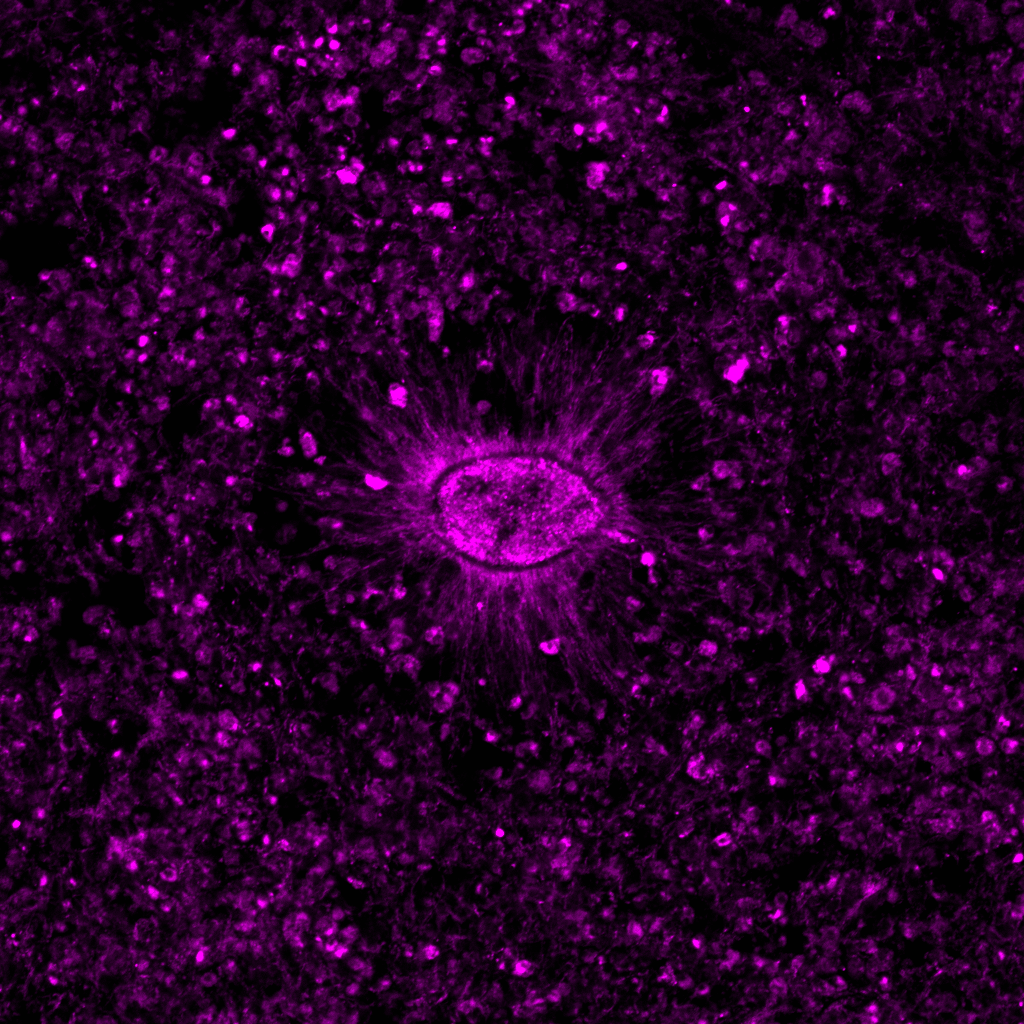

Supplement: Supplementary file 10 — Figure EV2 Source Data [file 44321_2025_302_MOESM10_ESM.zip › Figure EV2/EV2G/#15-4-caspase3.tif]

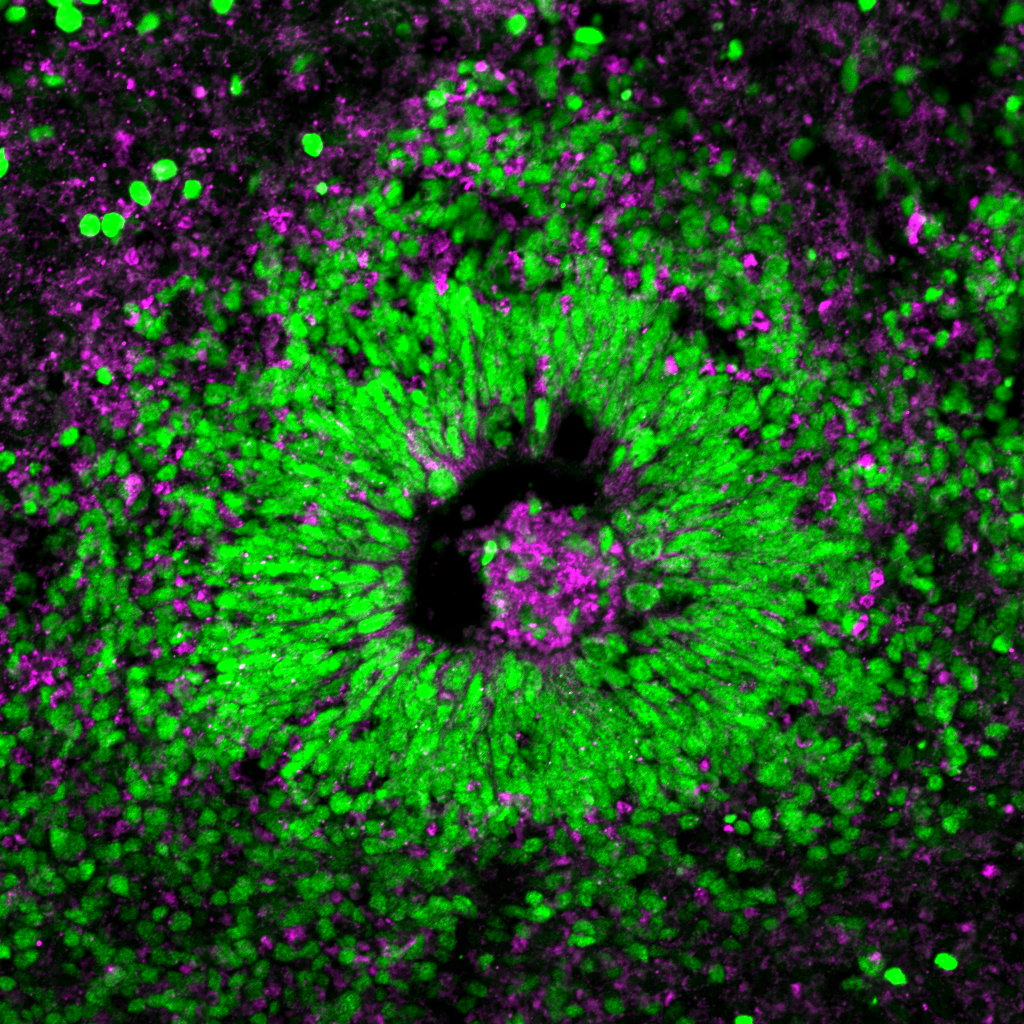

Supplement: Supplementary file 10 — Figure EV2 Source Data [file 44321_2025_302_MOESM10_ESM.zip › Figure EV2/EV2G/#6-6-merge.tif]

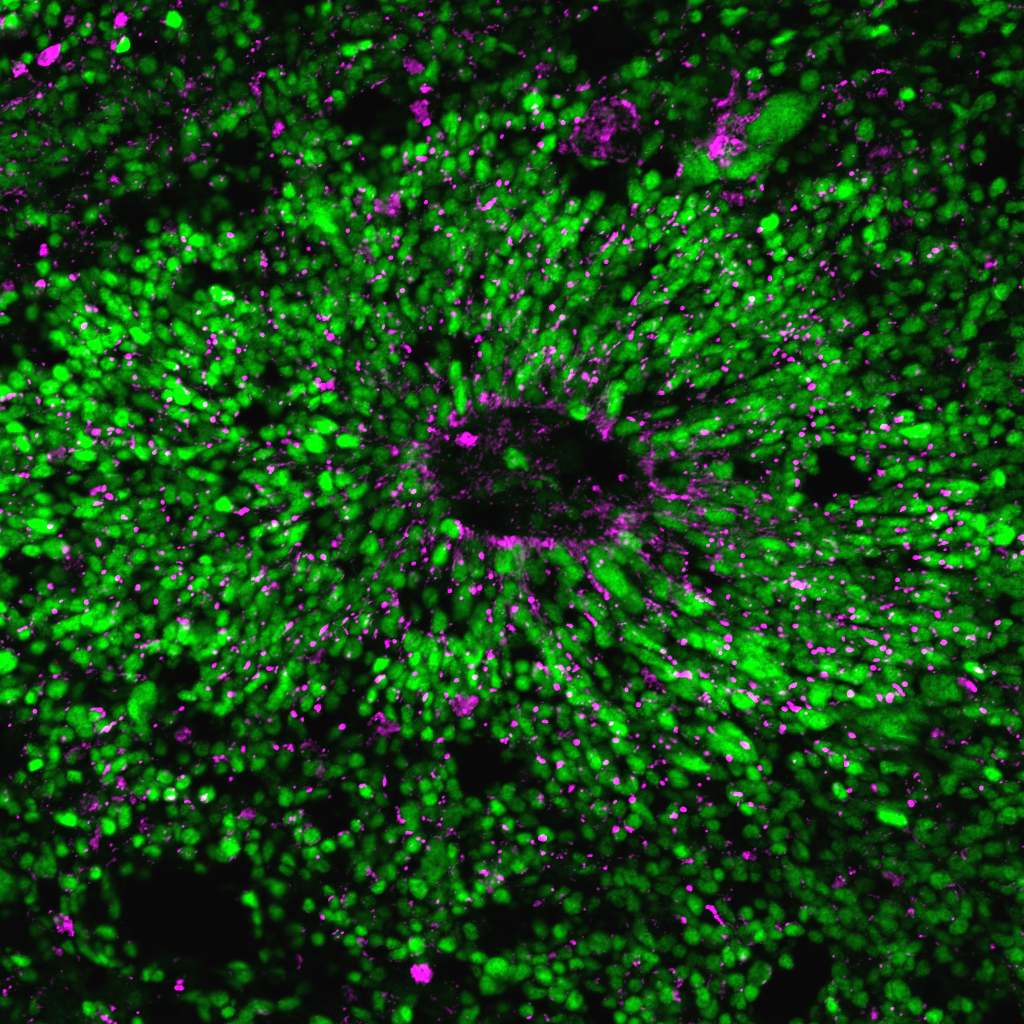

Supplement: Supplementary file 11 — Figure EV3 Source Data [file 44321_2025_302_MOESM11_ESM.zip › Figure EV3/EV3G/#10-6-merge.tif]

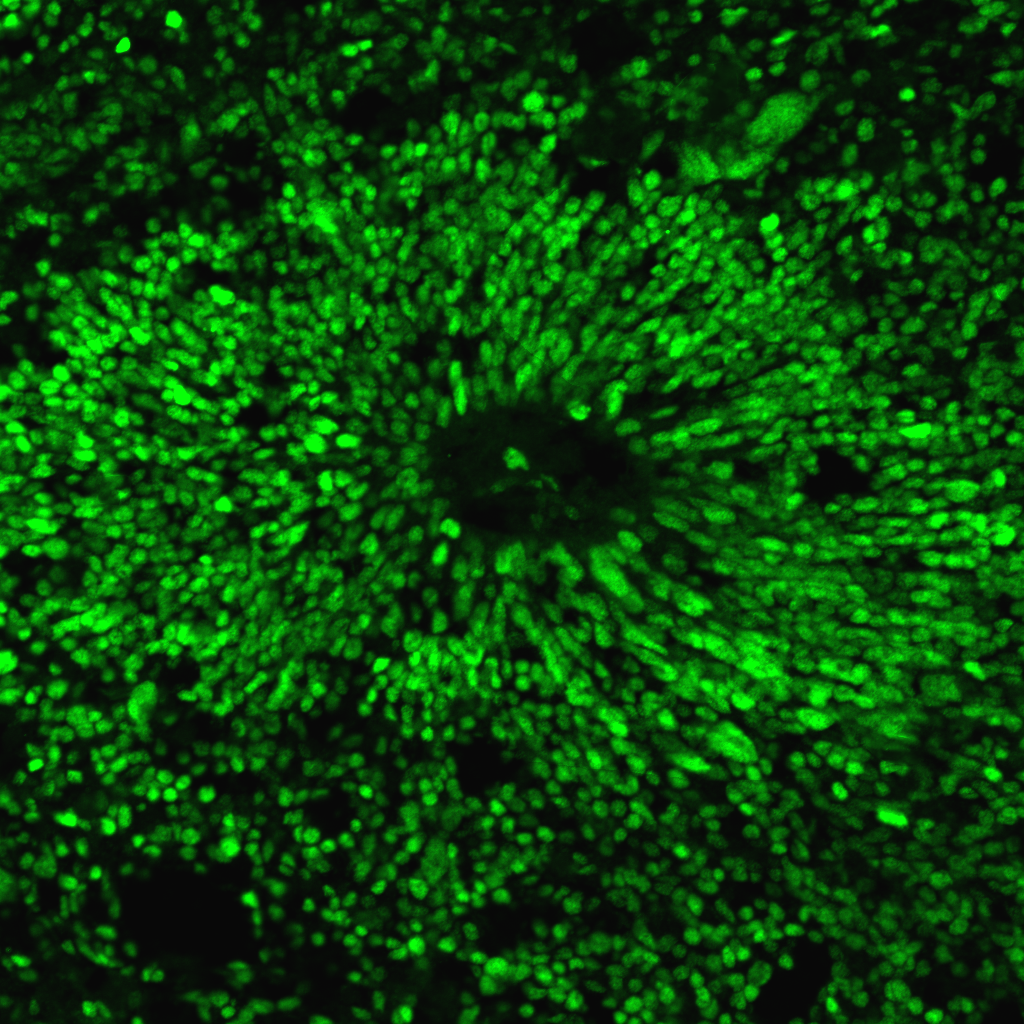

Supplement: Supplementary file 11 — Figure EV3 Source Data [file 44321_2025_302_MOESM11_ESM.zip › Figure EV3/EV3G/#10-6-PAX6.tif]

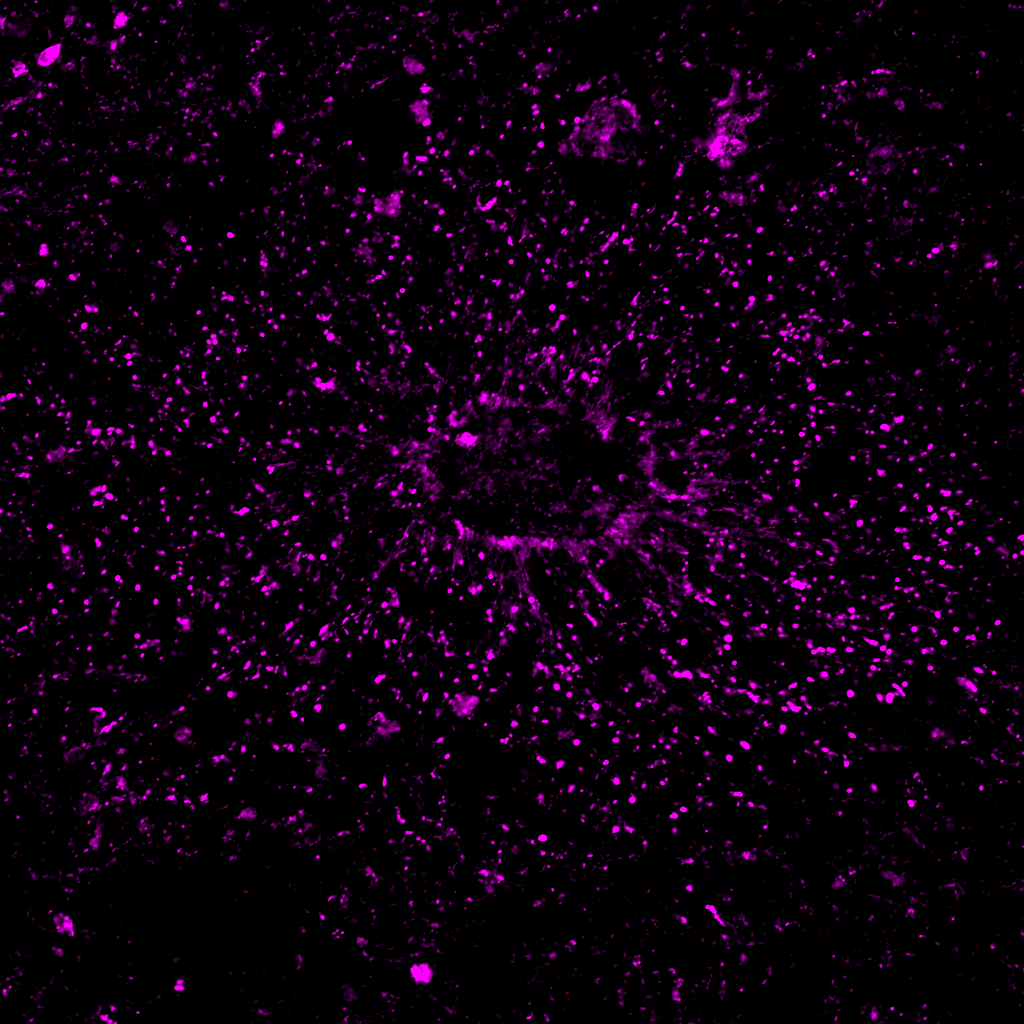

Supplement: Supplementary file 11 — Figure EV3 Source Data [file 44321_2025_302_MOESM11_ESM.zip › Figure EV3/EV3G/#10-6-caspase3.tif]

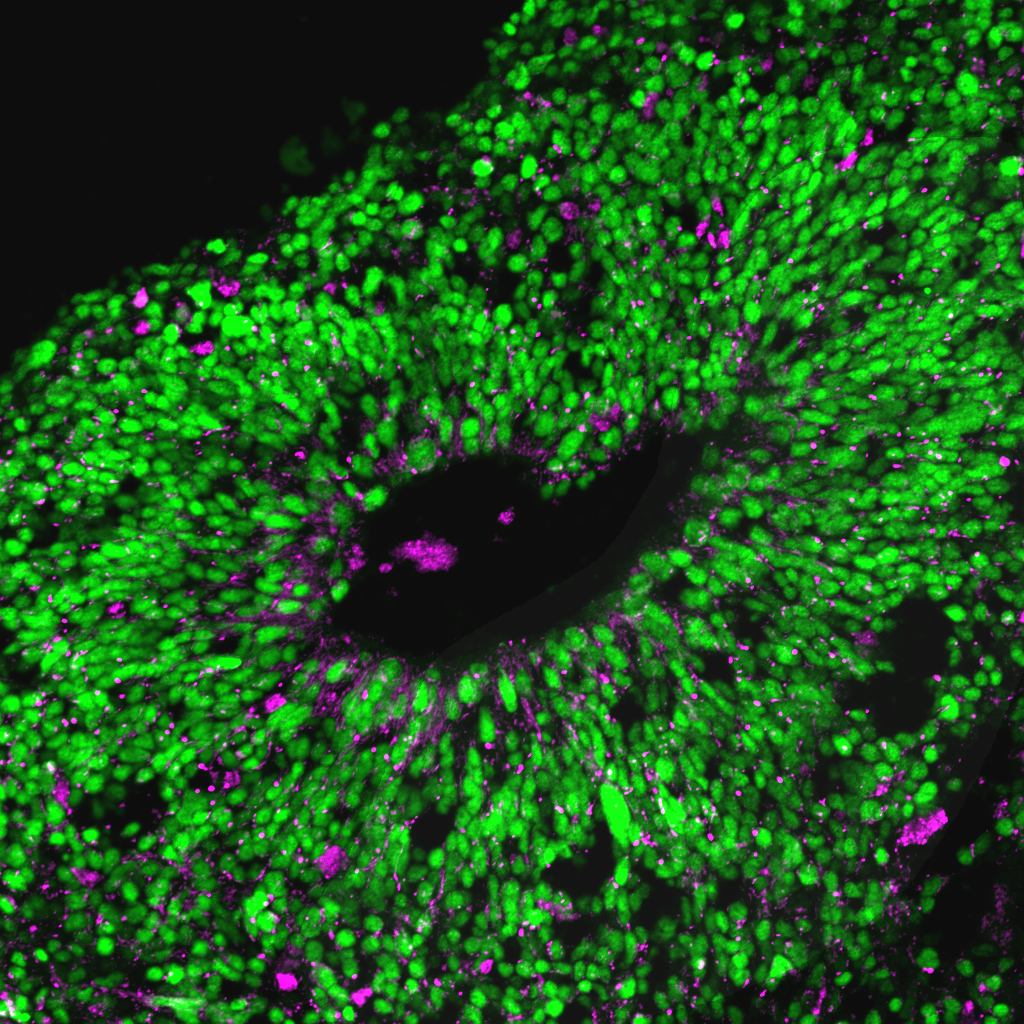

Supplement: Supplementary file 11 — Figure EV3 Source Data [file 44321_2025_302_MOESM11_ESM.zip › Figure EV3/EV3G/#4-1-merge.tif]

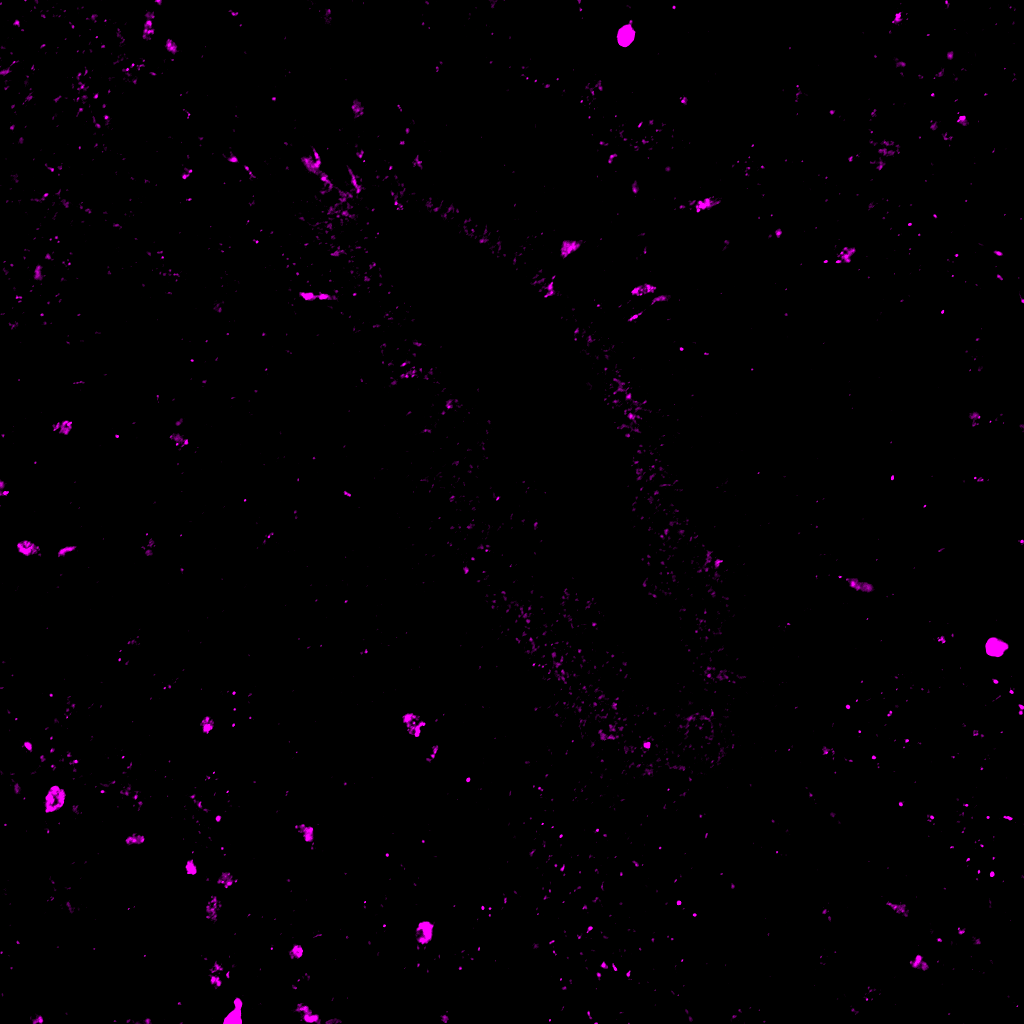

Supplement: Supplementary file 11 — Figure EV3 Source Data [file 44321_2025_302_MOESM11_ESM.zip › Figure EV3/EV3G/WT-caspase3.tif]

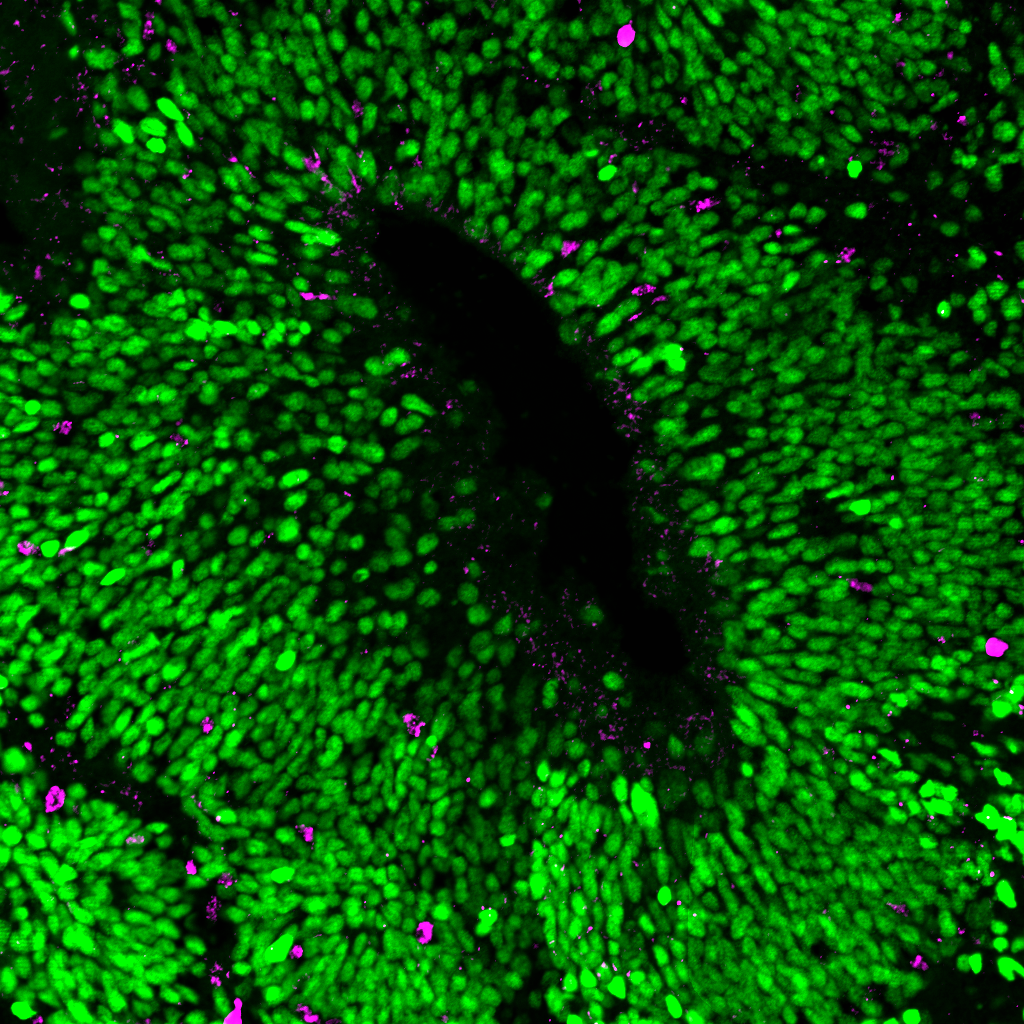

Supplement: Supplementary file 11 — Figure EV3 Source Data [file 44321_2025_302_MOESM11_ESM.zip › Figure EV3/EV3G/WT-merge.tif]

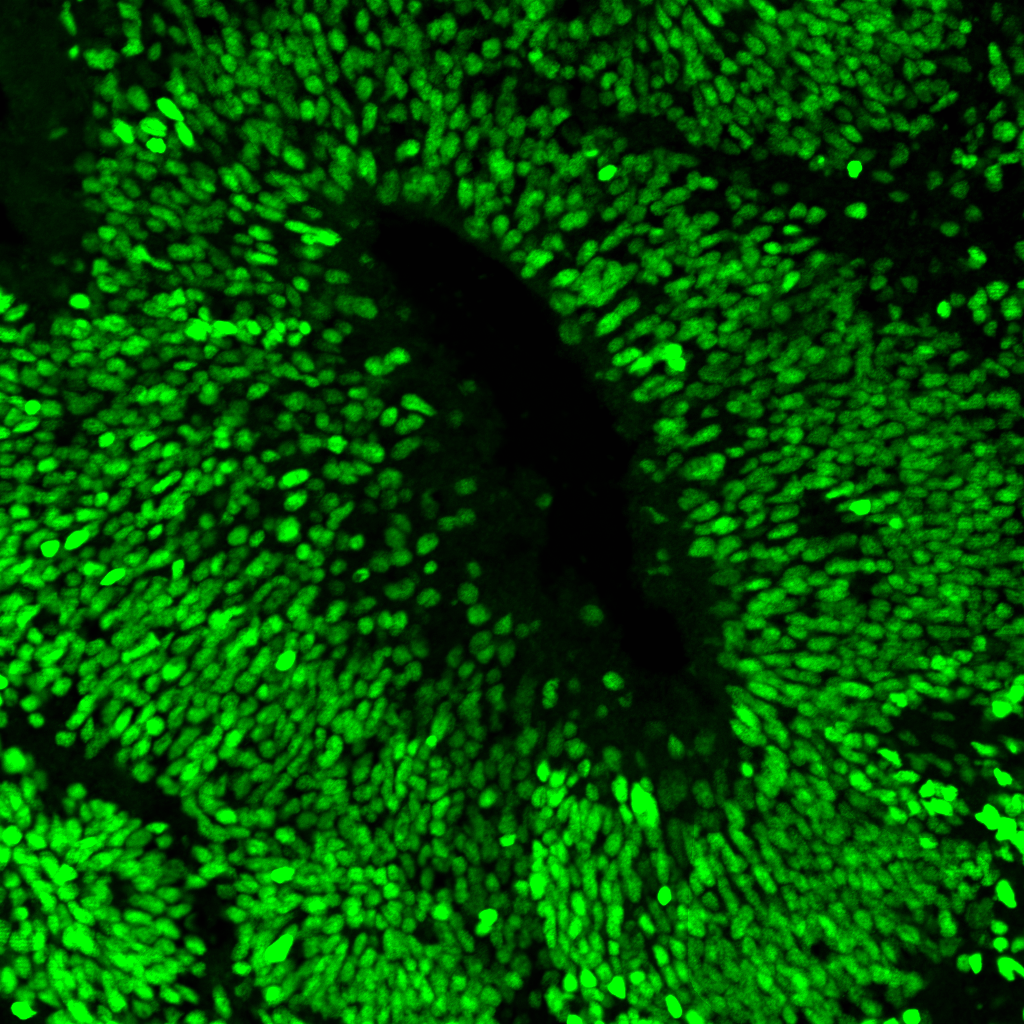

Supplement: Supplementary file 11 — Figure EV3 Source Data [file 44321_2025_302_MOESM11_ESM.zip › Figure EV3/EV3G/WT-PAX6.tif]

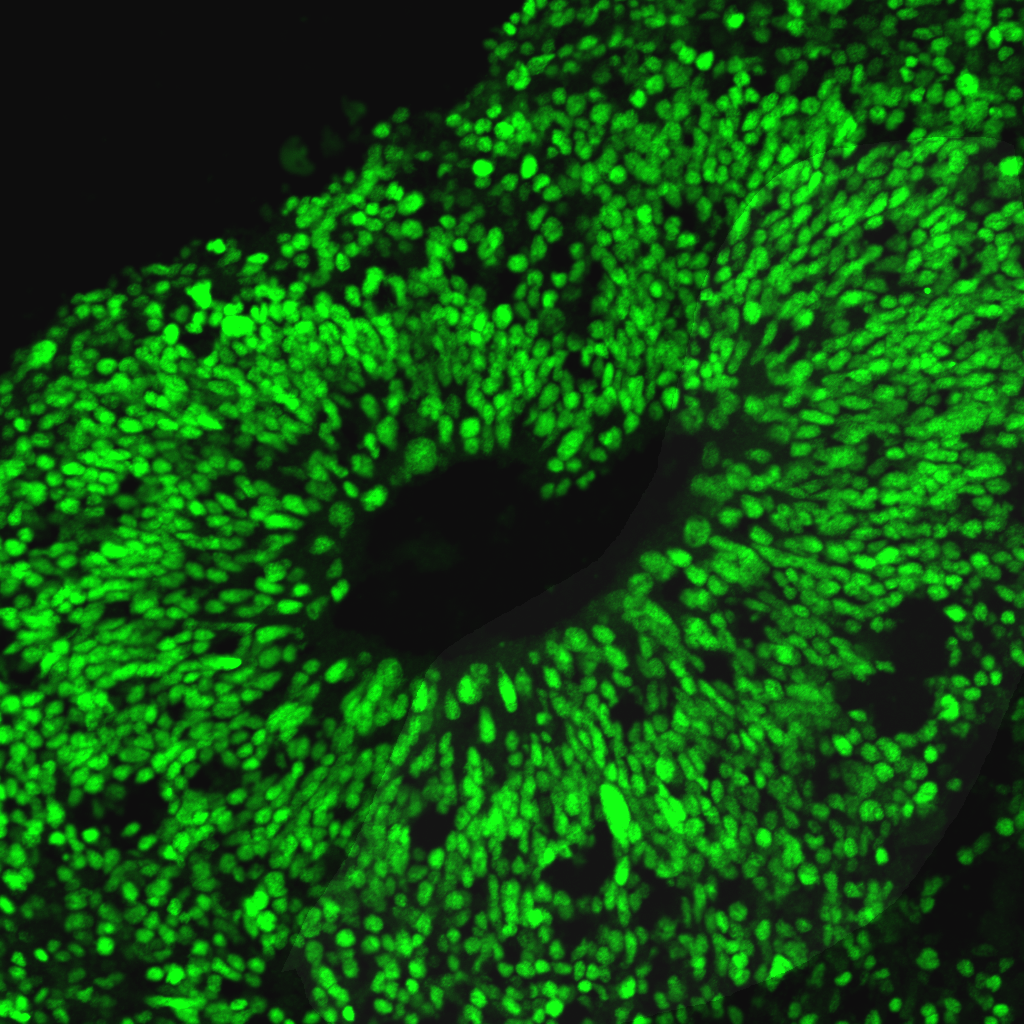

Supplement: Supplementary file 11 — Figure EV3 Source Data [file 44321_2025_302_MOESM11_ESM.zip › Figure EV3/EV3G/#4-1-PAX6.tif]

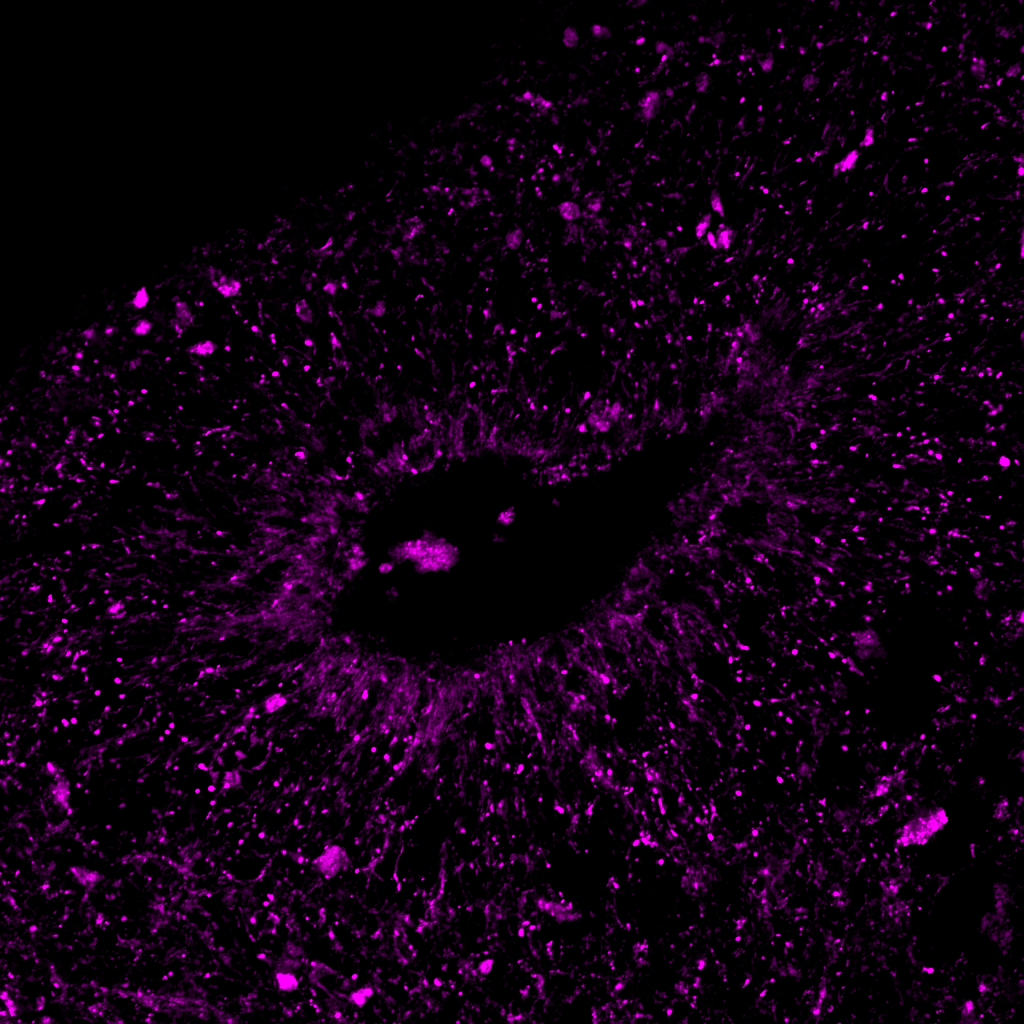

Supplement: Supplementary file 11 — Figure EV3 Source Data [file 44321_2025_302_MOESM11_ESM.zip › Figure EV3/EV3G/#4-1-caspase3.tif]

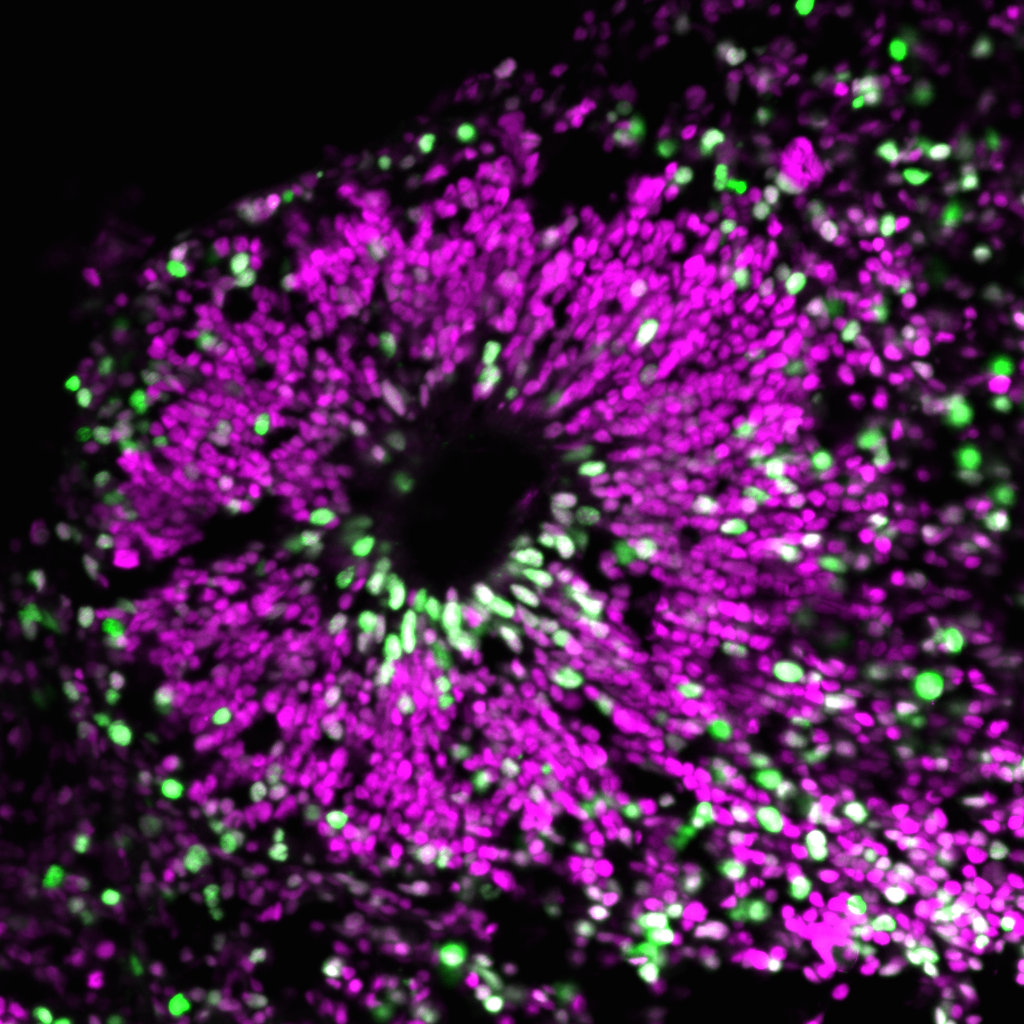

Supplement: Supplementary file 11 — Figure EV3 Source Data [file 44321_2025_302_MOESM11_ESM.zip › Figure EV3/EV3F/#10-6_merge.tif]

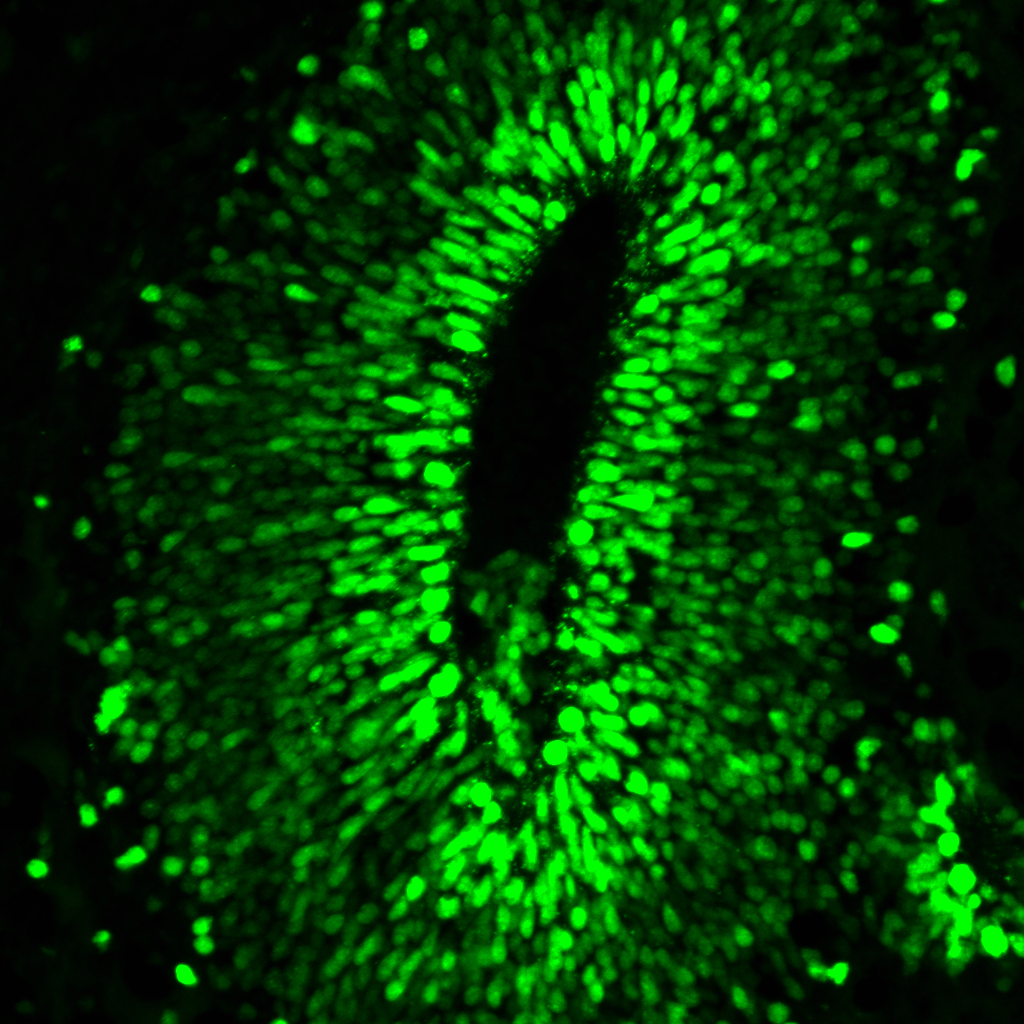

Supplement: Supplementary file 11 — Figure EV3 Source Data [file 44321_2025_302_MOESM11_ESM.zip › Figure EV3/EV3F/WT_Ki67.tif]

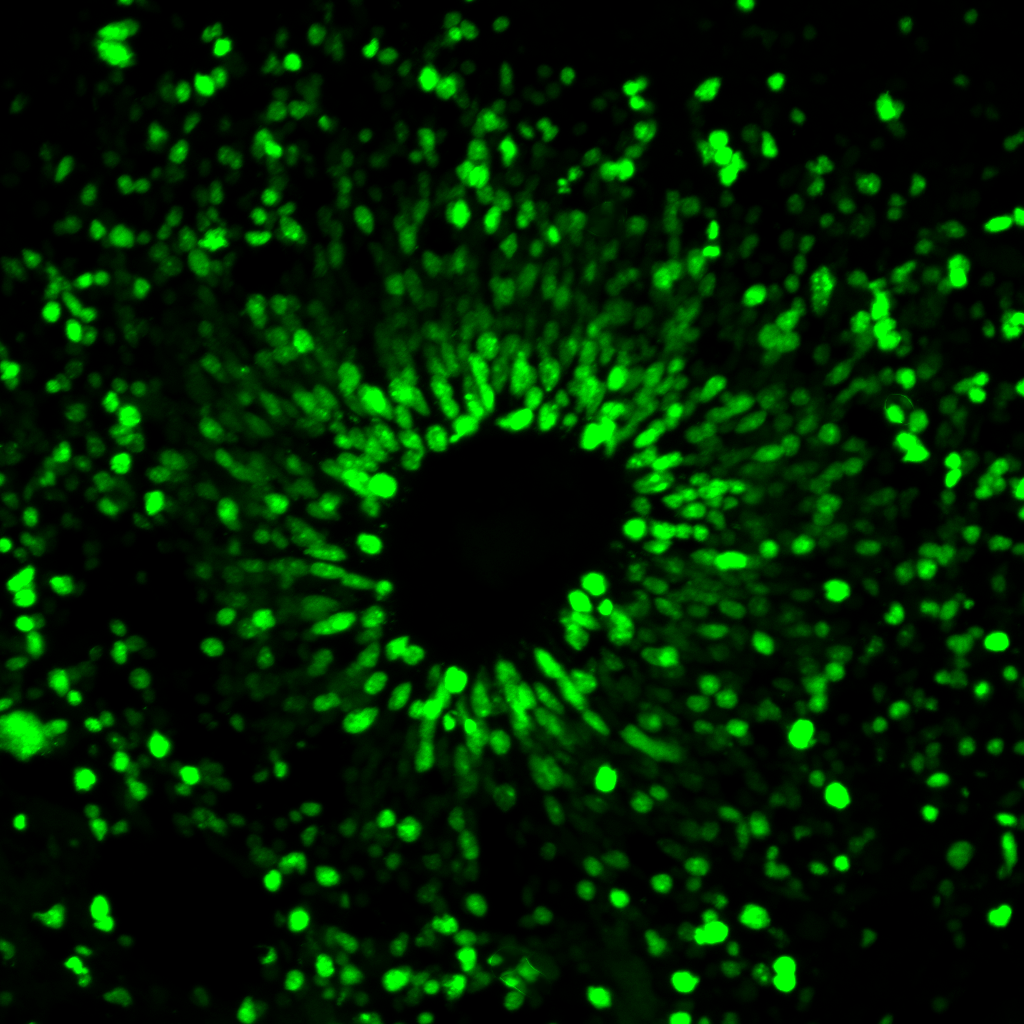

Supplement: Supplementary file 11 — Figure EV3 Source Data [file 44321_2025_302_MOESM11_ESM.zip › Figure EV3/EV3F/#4-1_Ki67.tif]

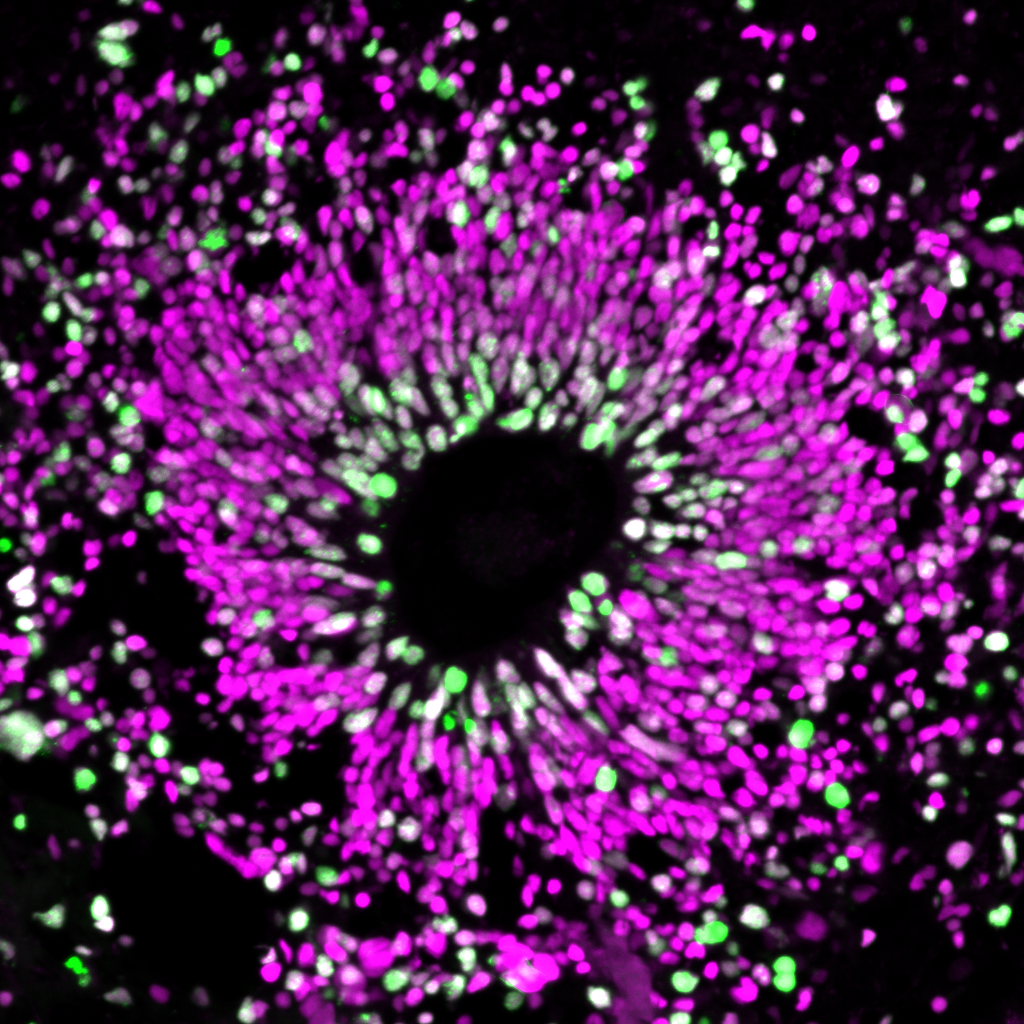

Supplement: Supplementary file 11 — Figure EV3 Source Data [file 44321_2025_302_MOESM11_ESM.zip › Figure EV3/EV3F/#4-1_merge.tif]

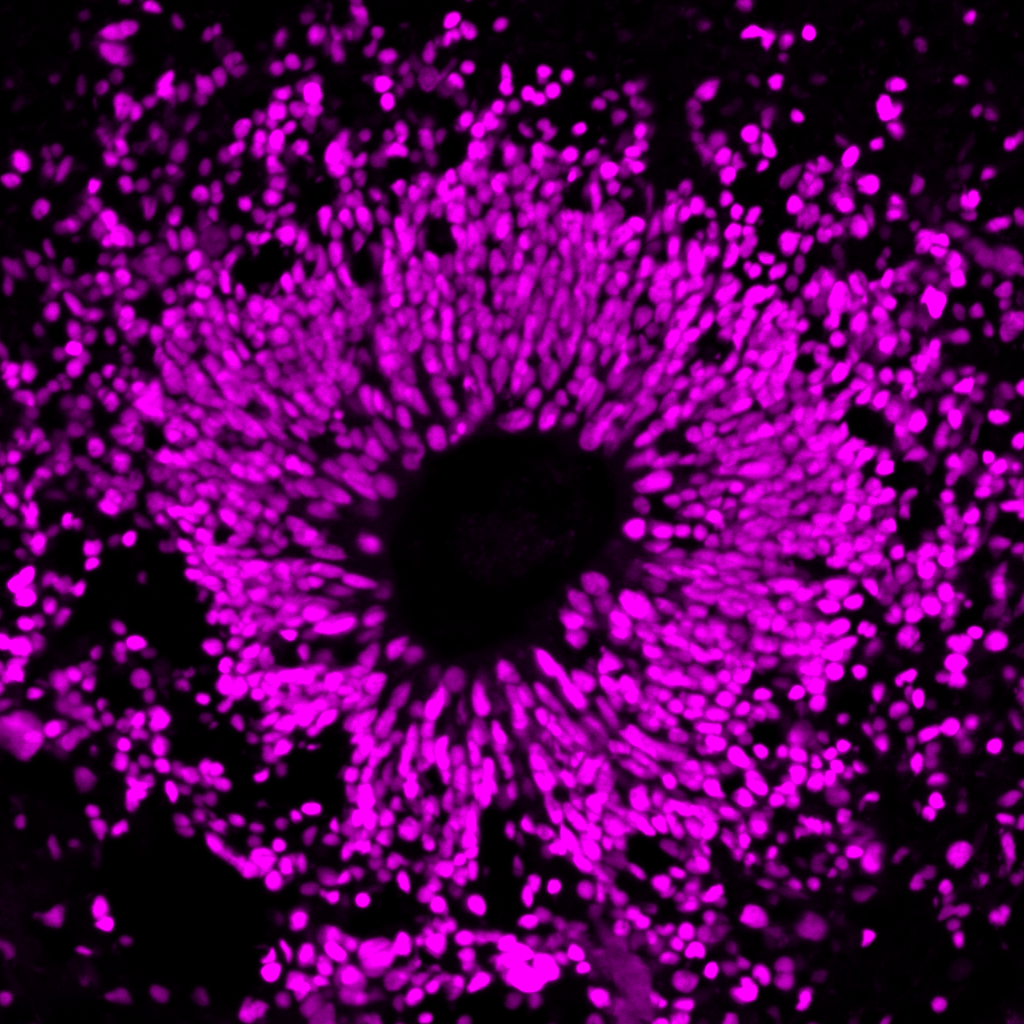

Supplement: Supplementary file 11 — Figure EV3 Source Data [file 44321_2025_302_MOESM11_ESM.zip › Figure EV3/EV3F/#4-1_SOX2.tif]

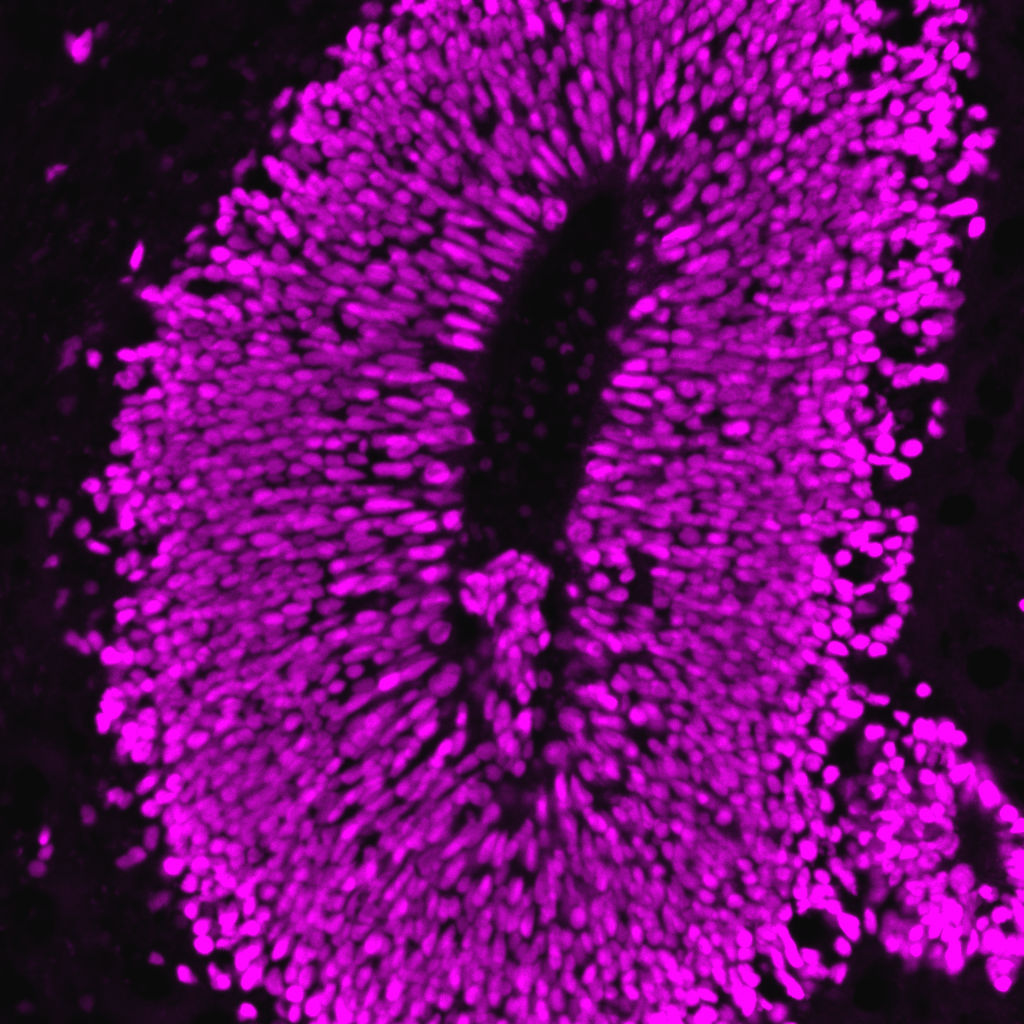

Supplement: Supplementary file 11 — Figure EV3 Source Data [file 44321_2025_302_MOESM11_ESM.zip › Figure EV3/EV3F/WT_SOX2.tif]

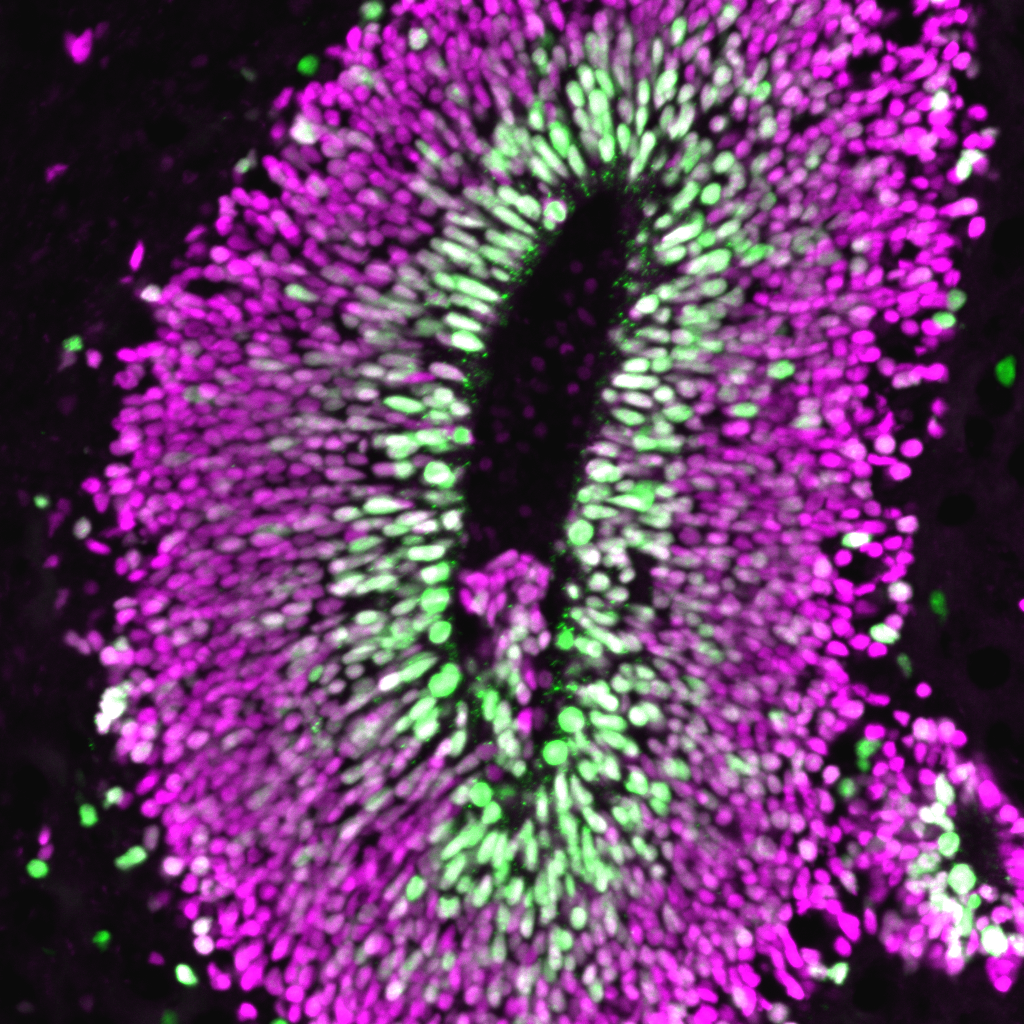

Supplement: Supplementary file 11 — Figure EV3 Source Data [file 44321_2025_302_MOESM11_ESM.zip › Figure EV3/EV3F/WT_merge.tif]

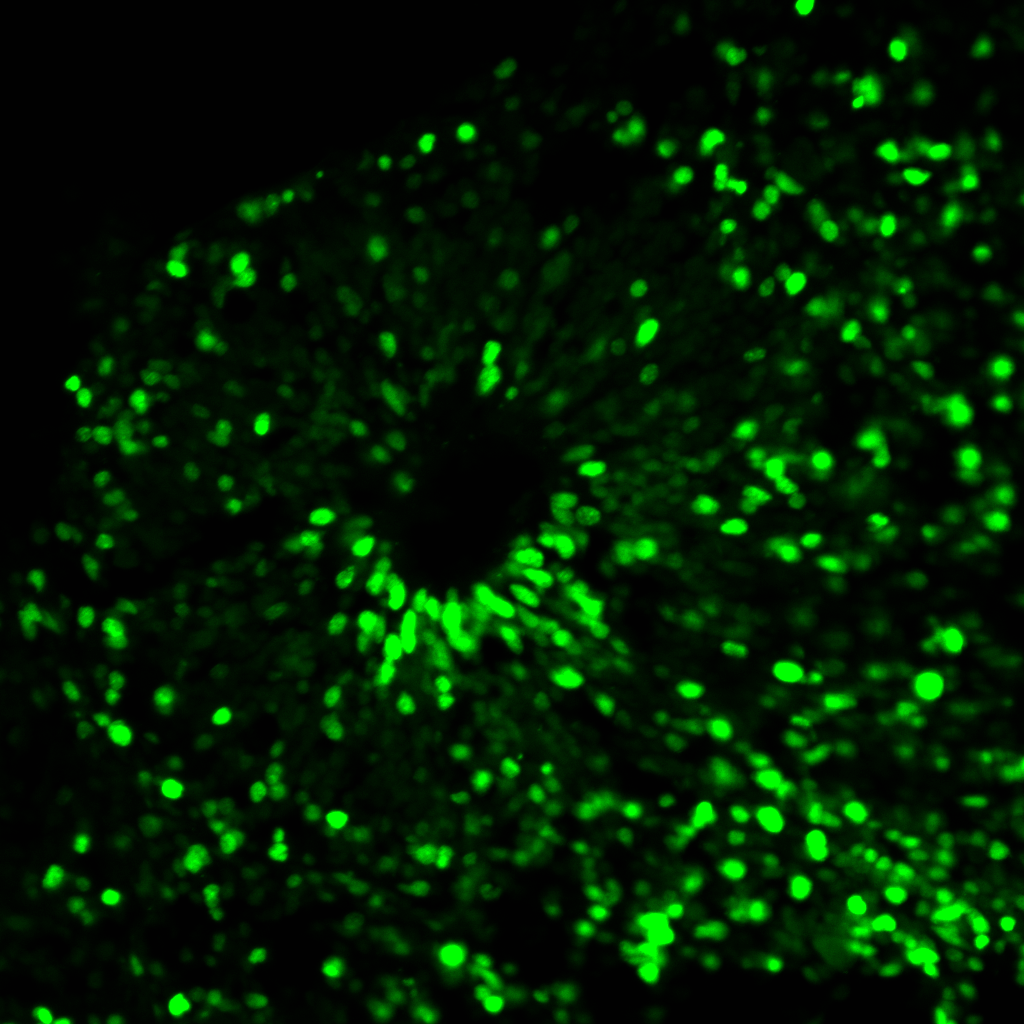

Supplement: Supplementary file 11 — Figure EV3 Source Data [file 44321_2025_302_MOESM11_ESM.zip › Figure EV3/EV3F/#10-6_Ki67.tif]

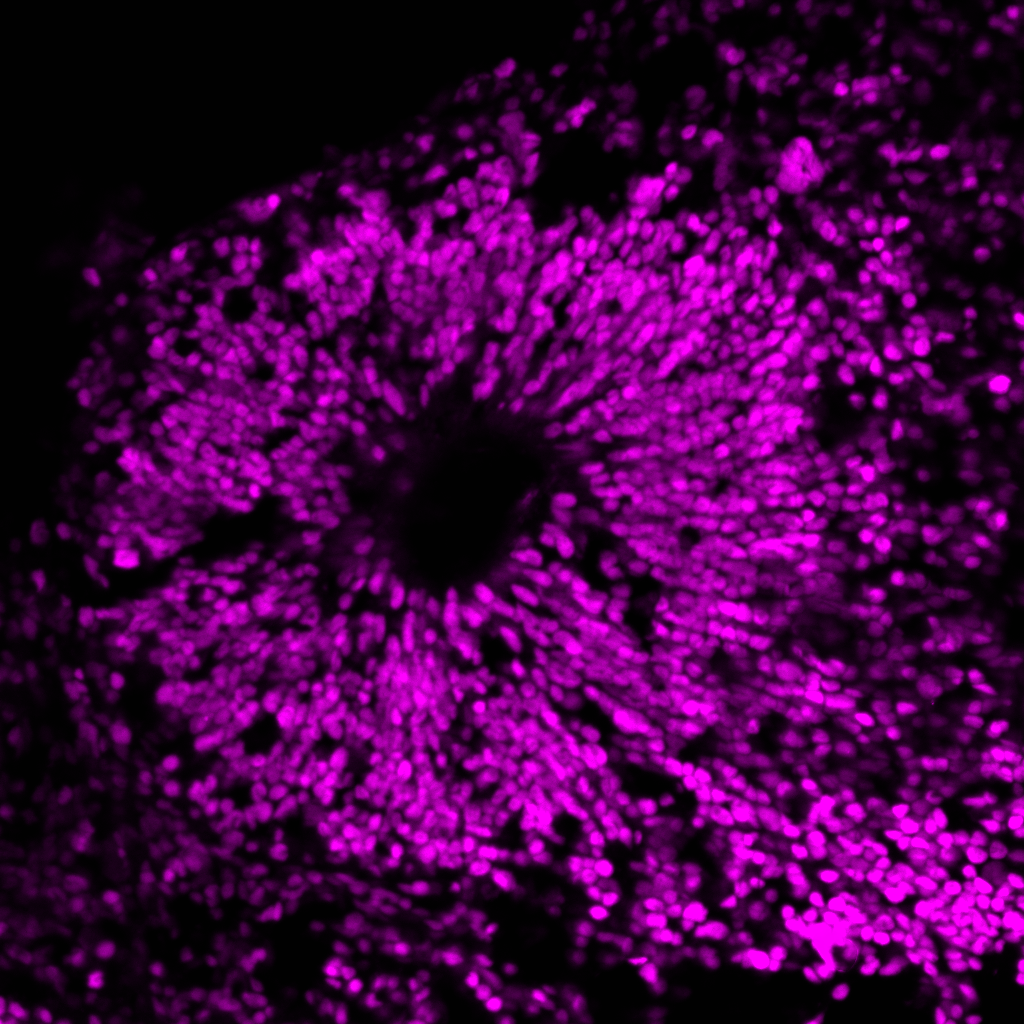

Supplement: Supplementary file 11 — Figure EV3 Source Data [file 44321_2025_302_MOESM11_ESM.zip › Figure EV3/EV3F/#10-6_SOX2.tif]

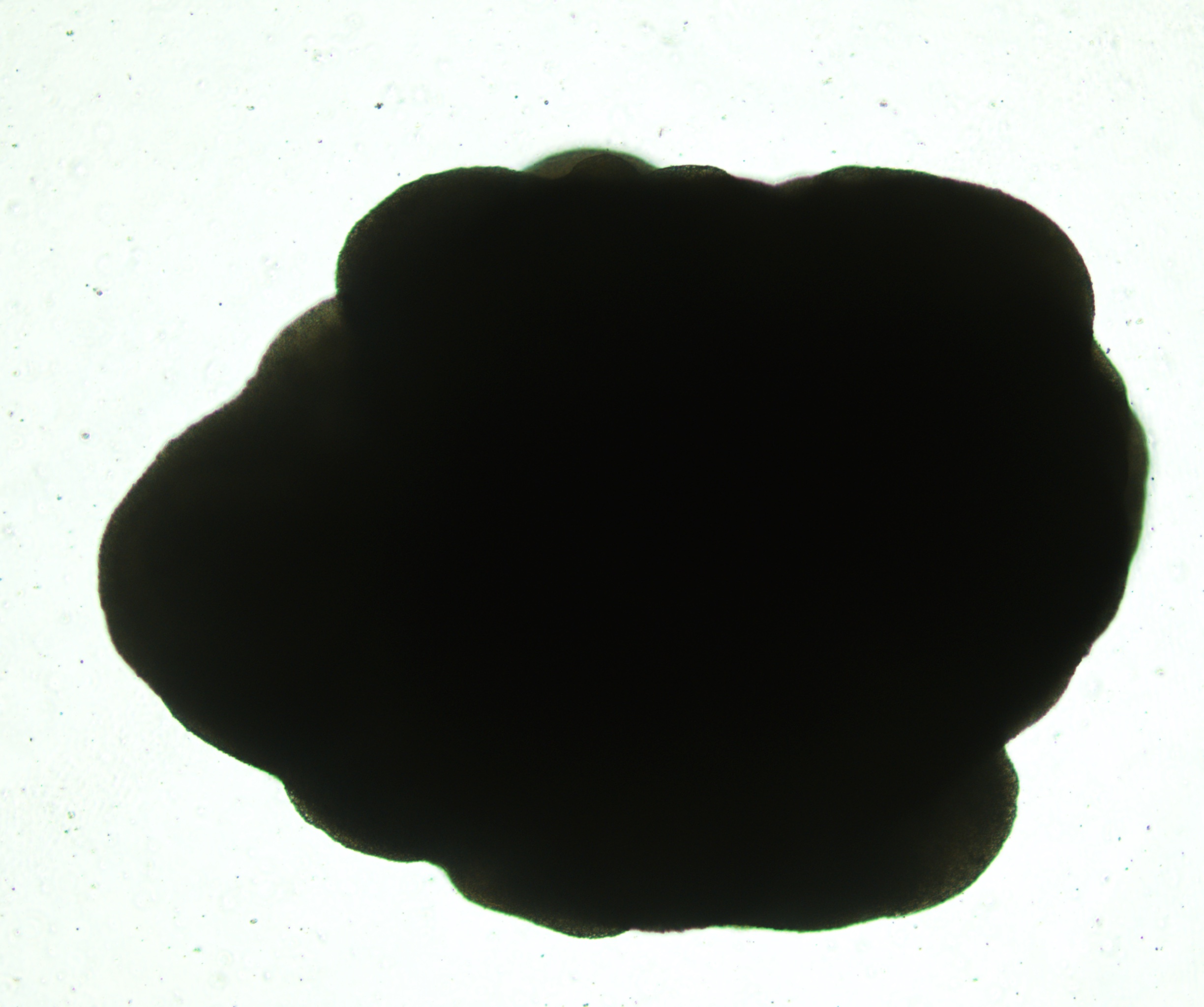

Supplement: Supplementary file 11 — Figure EV3 Source Data [file 44321_2025_302_MOESM11_ESM.zip › Figure EV3/EV3A/Day40_WT.jpeg]

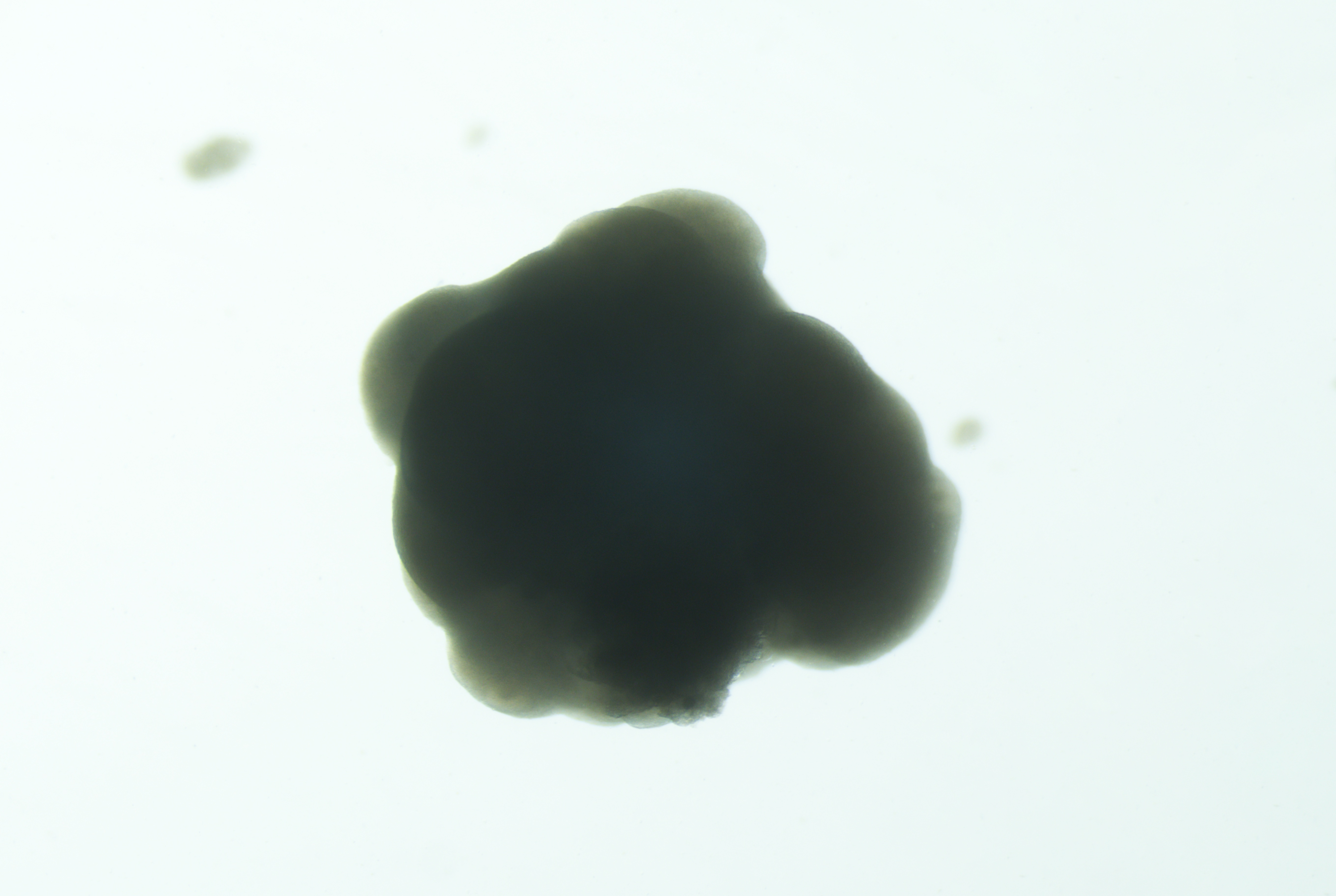

Supplement: Supplementary file 11 — Figure EV3 Source Data [file 44321_2025_302_MOESM11_ESM.zip › Figure EV3/EV3A/Day50_WT.jpeg]

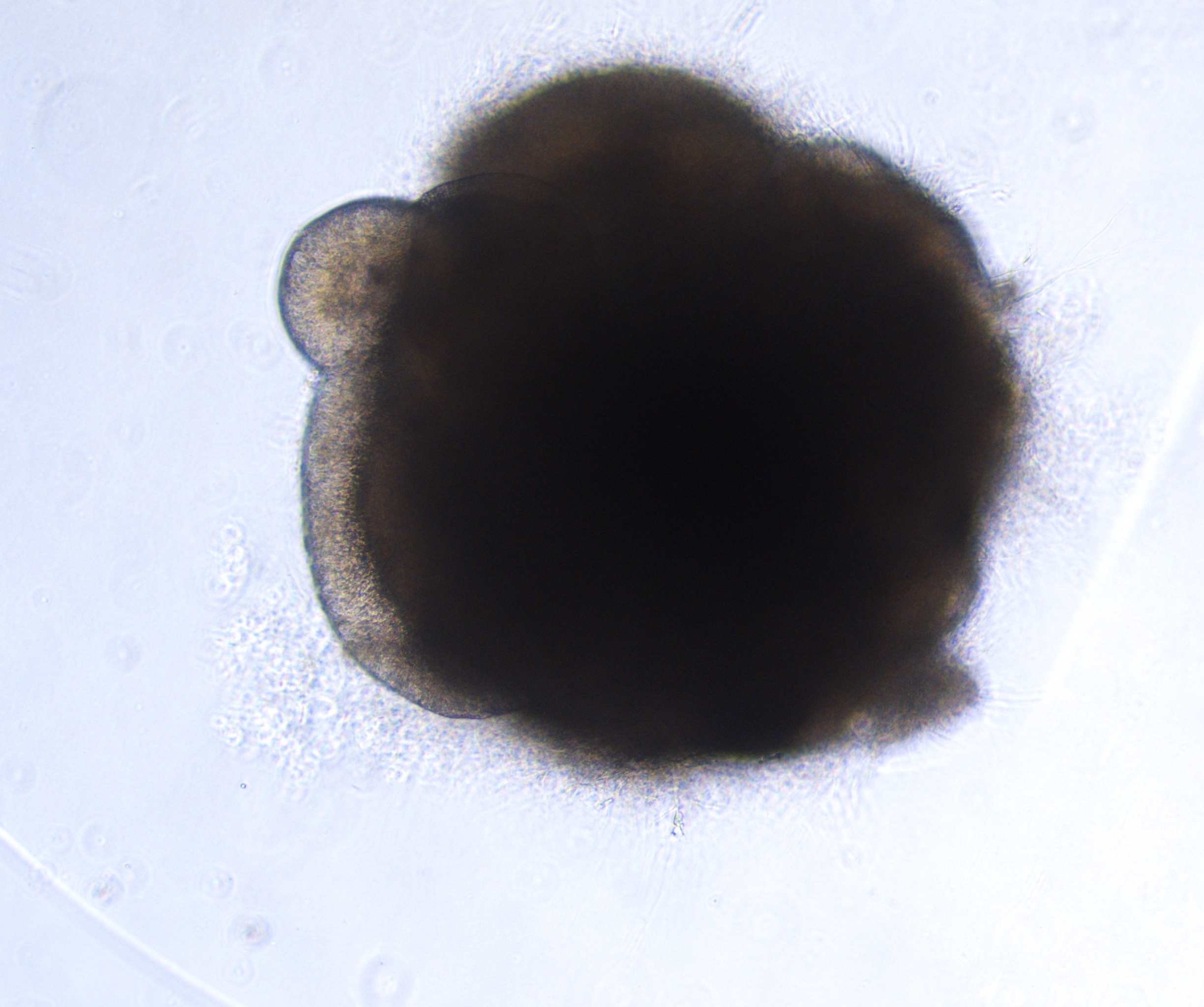

Supplement: Supplementary file 11 — Figure EV3 Source Data [file 44321_2025_302_MOESM11_ESM.zip › Figure EV3/EV3A/Day20_10-6.jpeg]

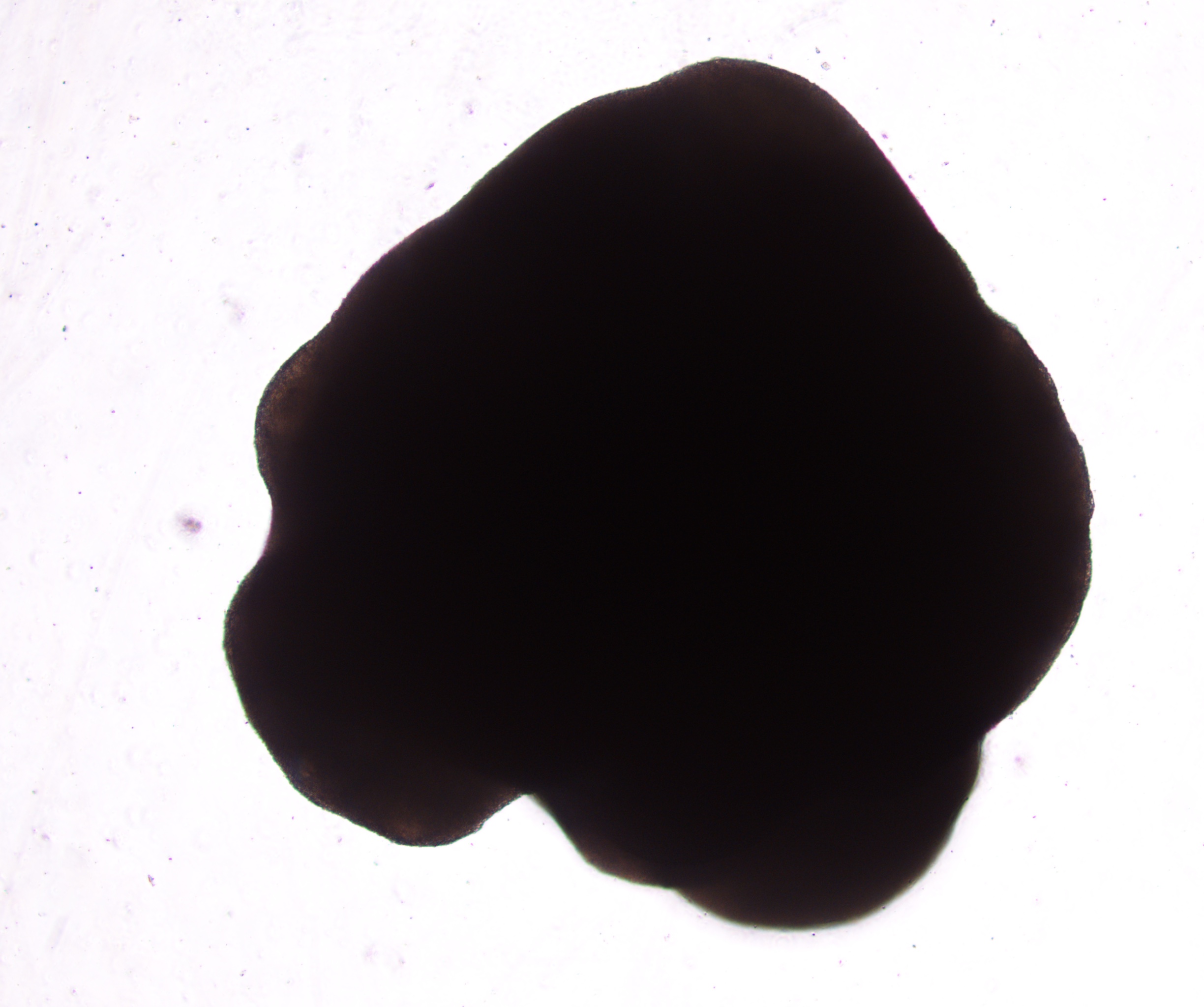

Supplement: Supplementary file 11 — Figure EV3 Source Data [file 44321_2025_302_MOESM11_ESM.zip › Figure EV3/EV3A/Day45_4-1.jpeg]

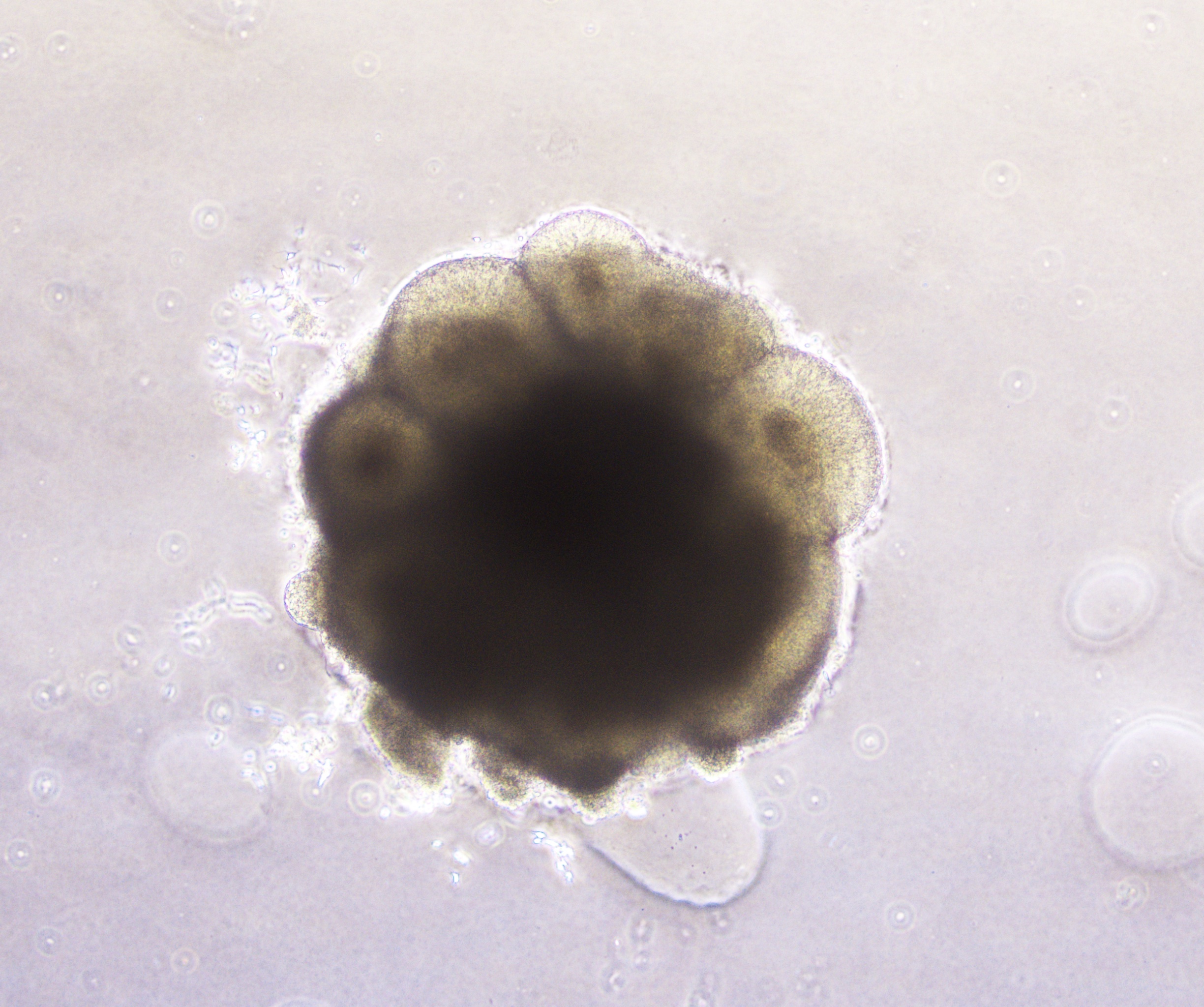

Supplement: Supplementary file 11 — Figure EV3 Source Data [file 44321_2025_302_MOESM11_ESM.zip › Figure EV3/EV3A/Day15_10-6.jpeg]

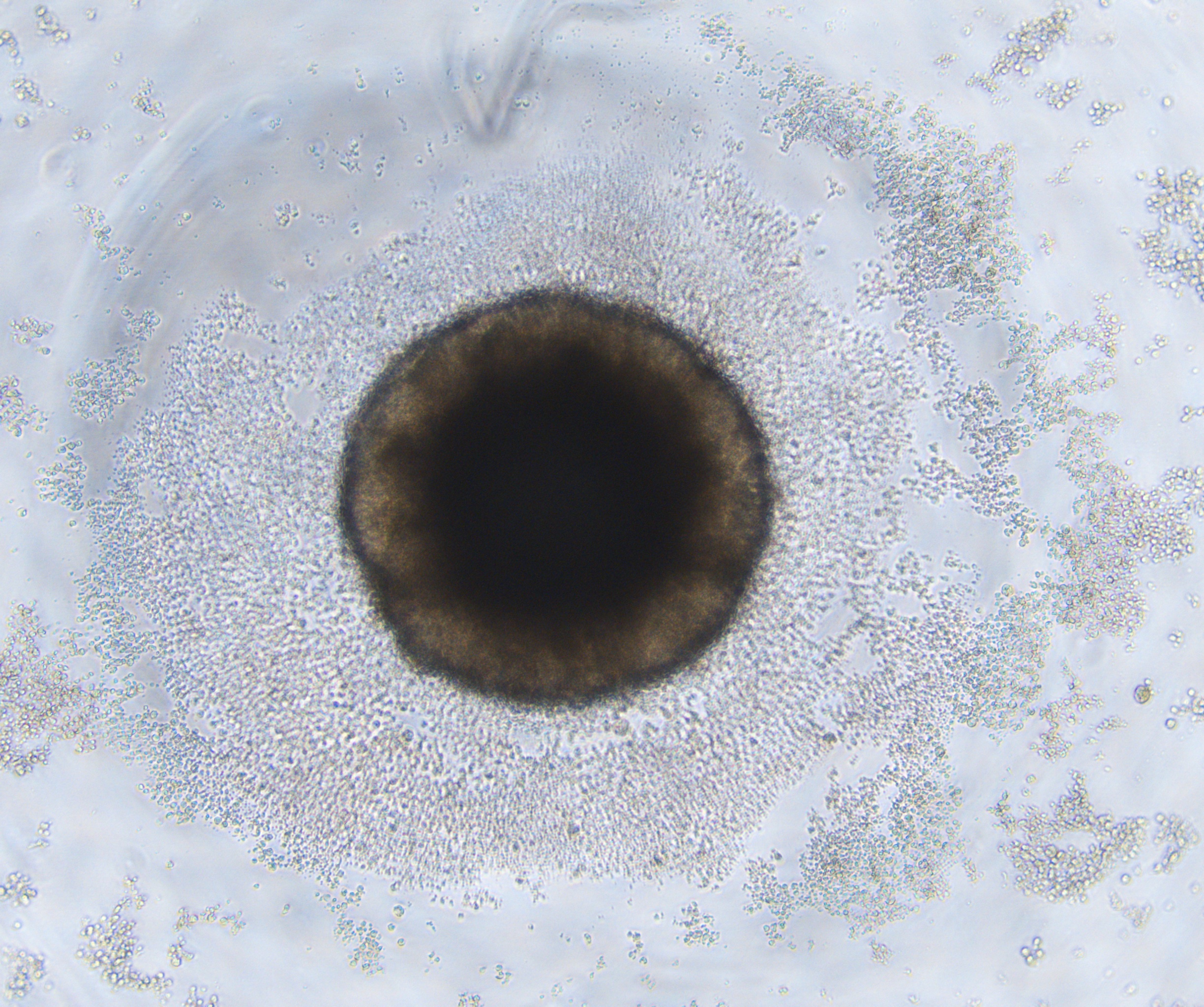

Supplement: Supplementary file 11 — Figure EV3 Source Data [file 44321_2025_302_MOESM11_ESM.zip › Figure EV3/EV3A/Day10_4-1.jpeg]

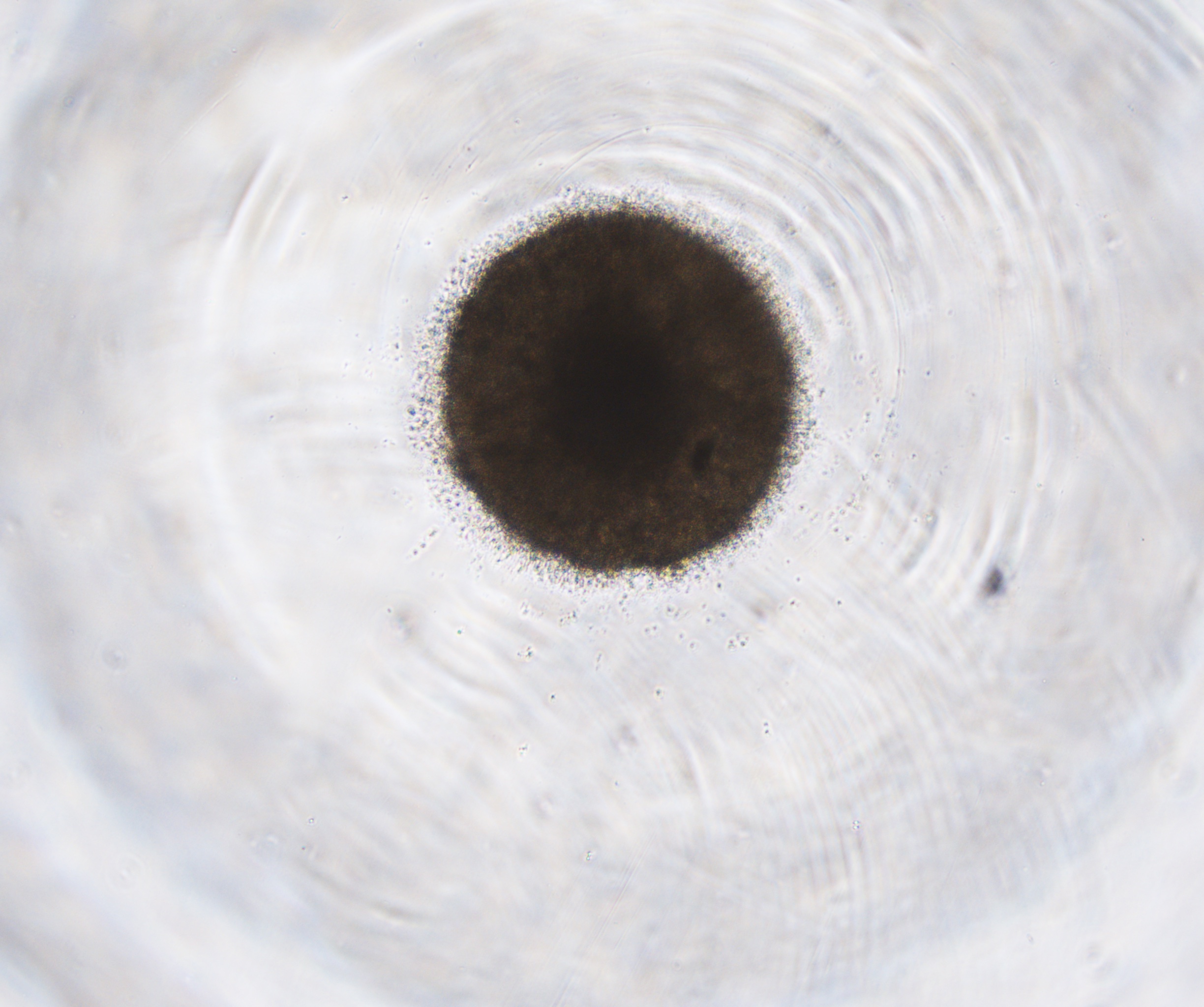

Supplement: Supplementary file 11 — Figure EV3 Source Data [file 44321_2025_302_MOESM11_ESM.zip › Figure EV3/EV3A/Day3_4-1.jpeg]

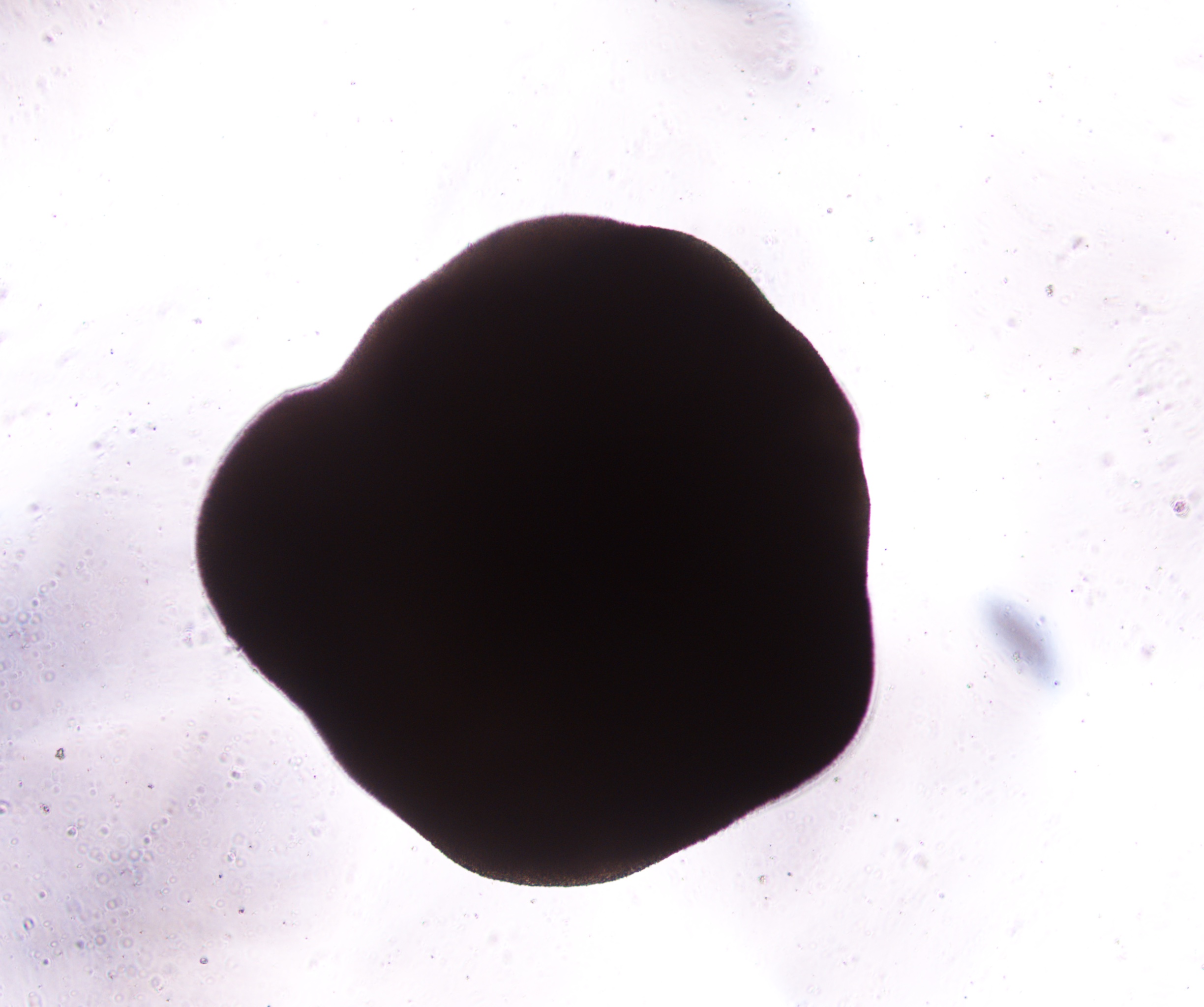

Supplement: Supplementary file 11 — Figure EV3 Source Data [file 44321_2025_302_MOESM11_ESM.zip › Figure EV3/EV3A/Day45_10-6.jpeg]

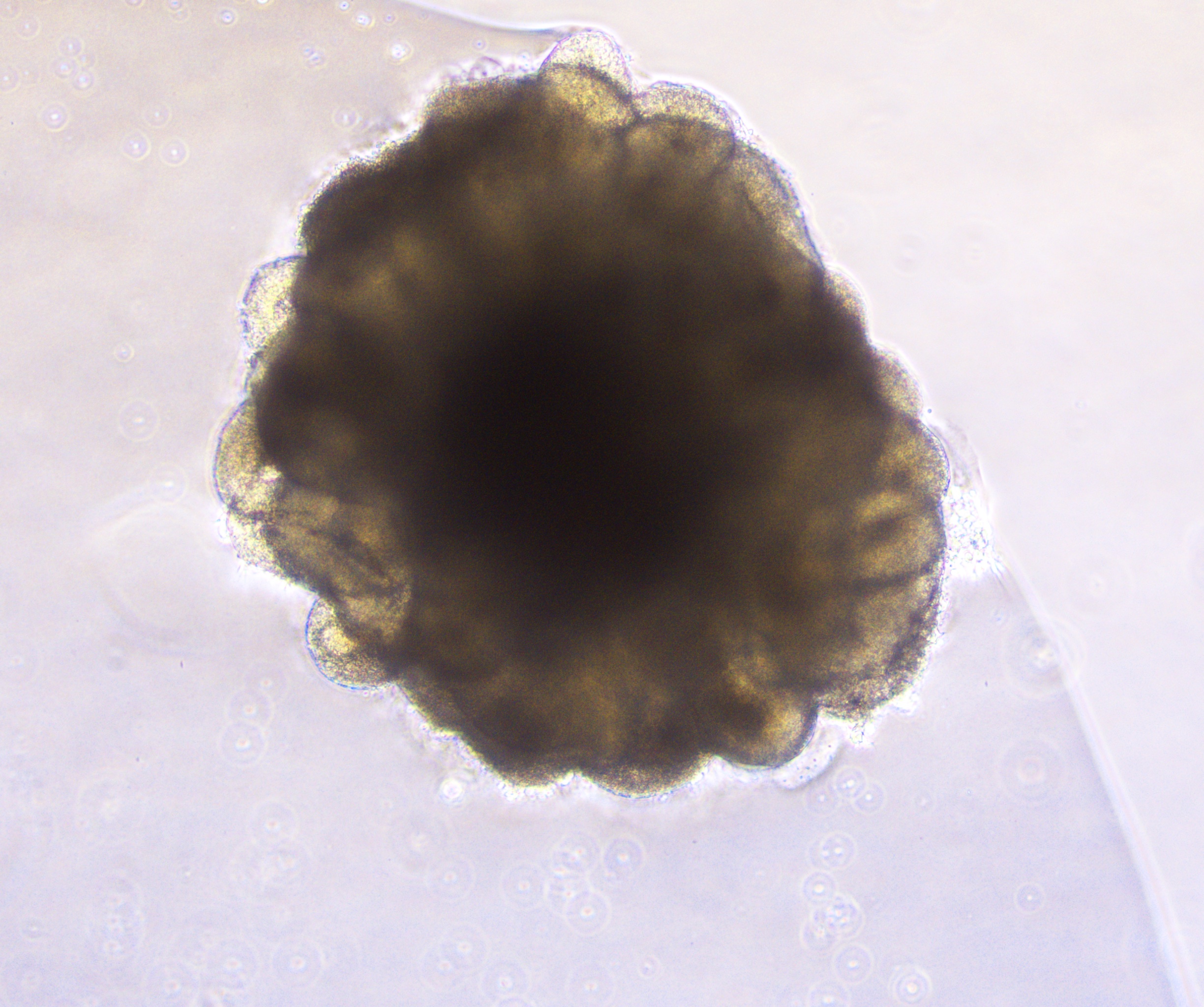

Supplement: Supplementary file 11 — Figure EV3 Source Data [file 44321_2025_302_MOESM11_ESM.zip › Figure EV3/EV3A/Day15_WT.jpeg]

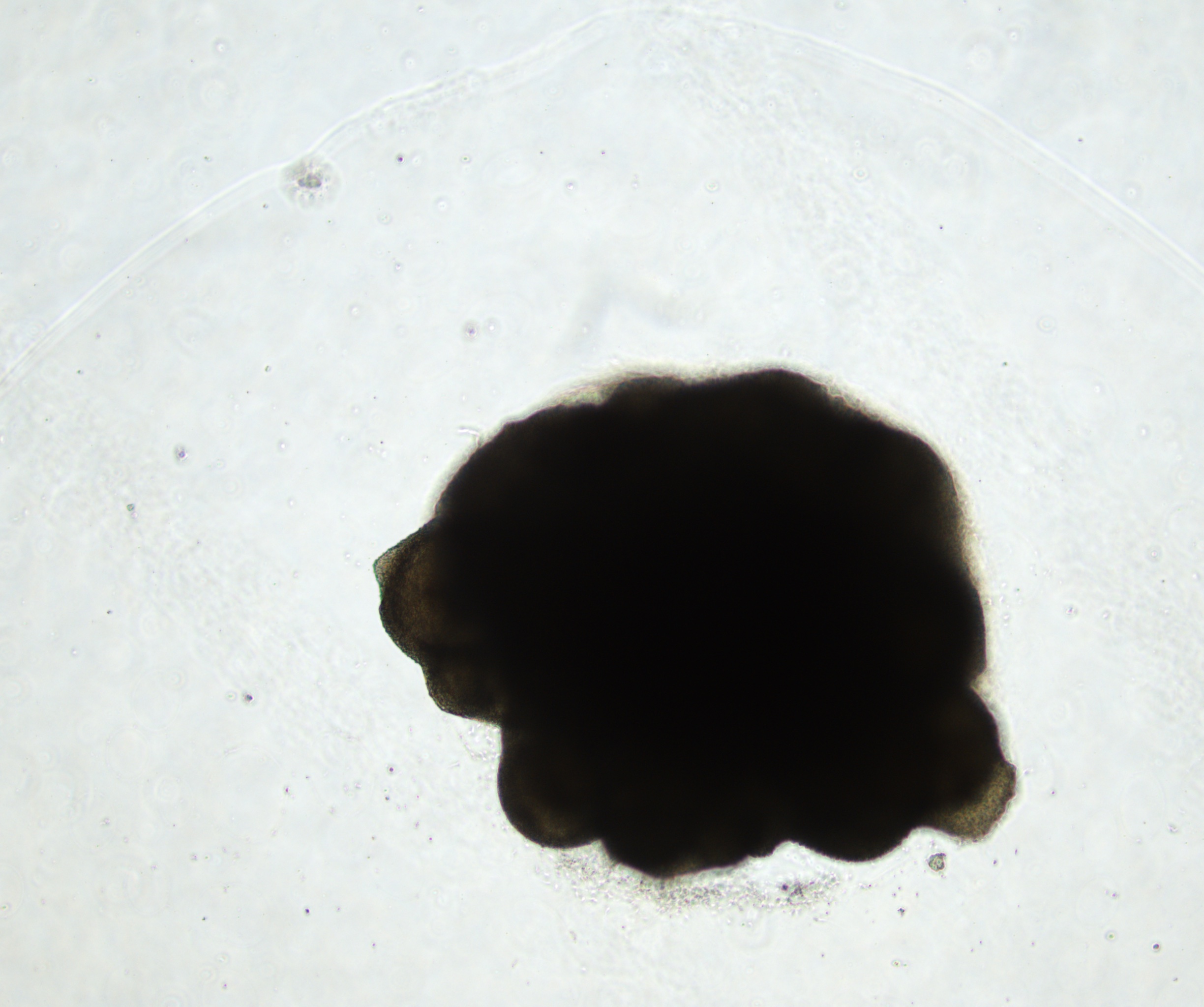

Supplement: Supplementary file 11 — Figure EV3 Source Data [file 44321_2025_302_MOESM11_ESM.zip › Figure EV3/EV3A/Day25_4-1.jpeg]

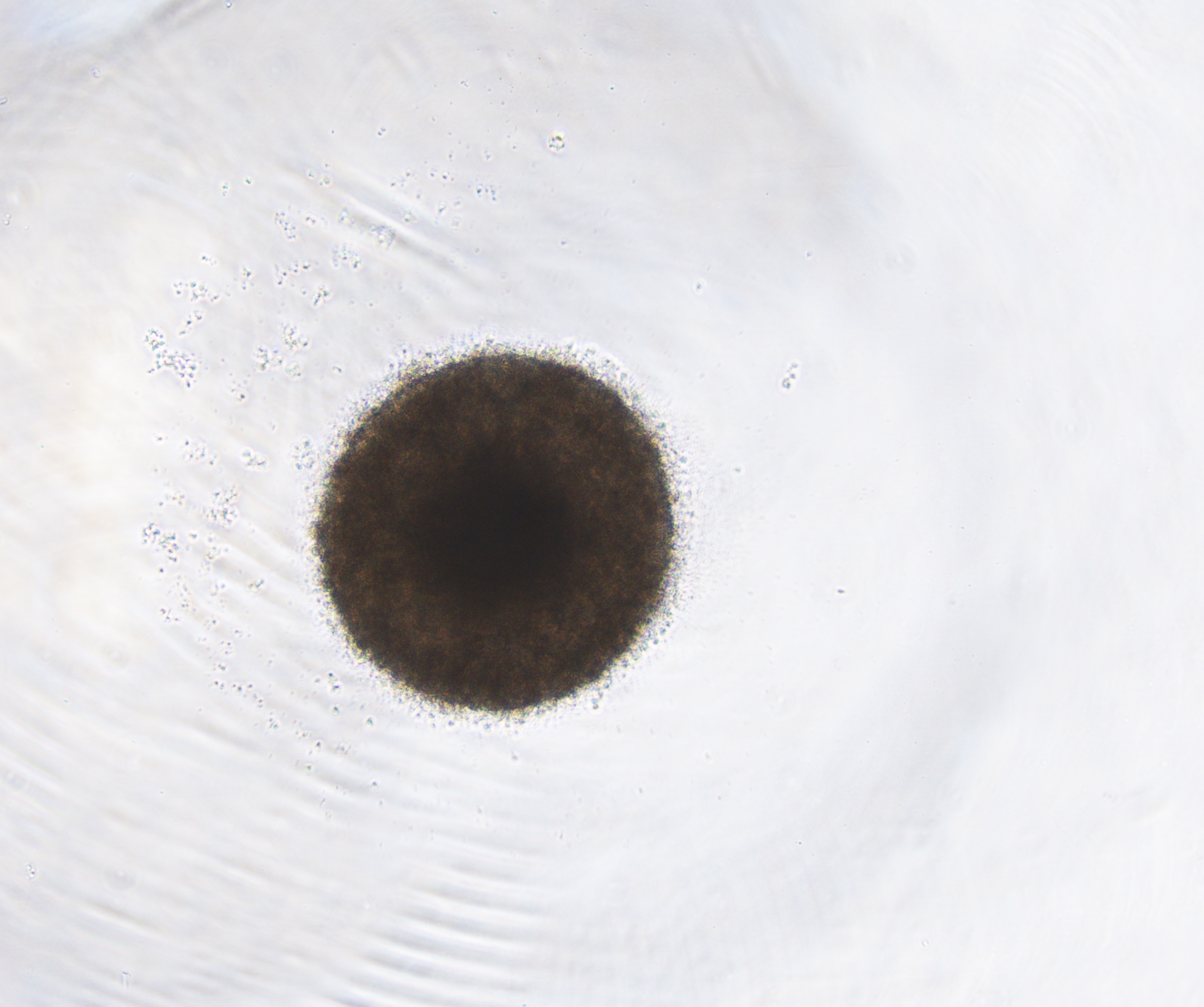

Supplement: Supplementary file 11 — Figure EV3 Source Data [file 44321_2025_302_MOESM11_ESM.zip › Figure EV3/EV3A/Day3_WT.jpeg]

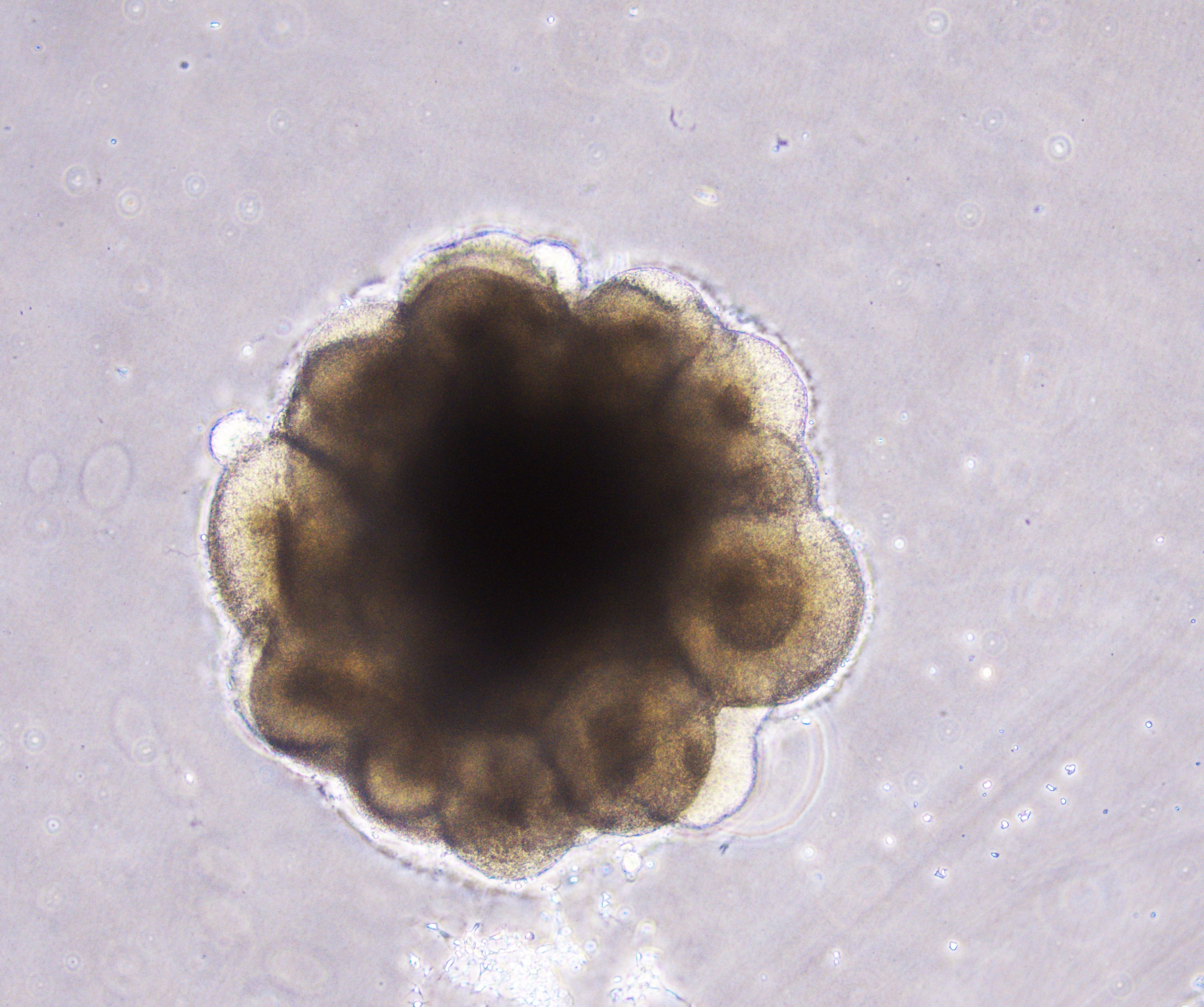

Supplement: Supplementary file 11 — Figure EV3 Source Data [file 44321_2025_302_MOESM11_ESM.zip › Figure EV3/EV3A/Day15_4-4.jpeg]

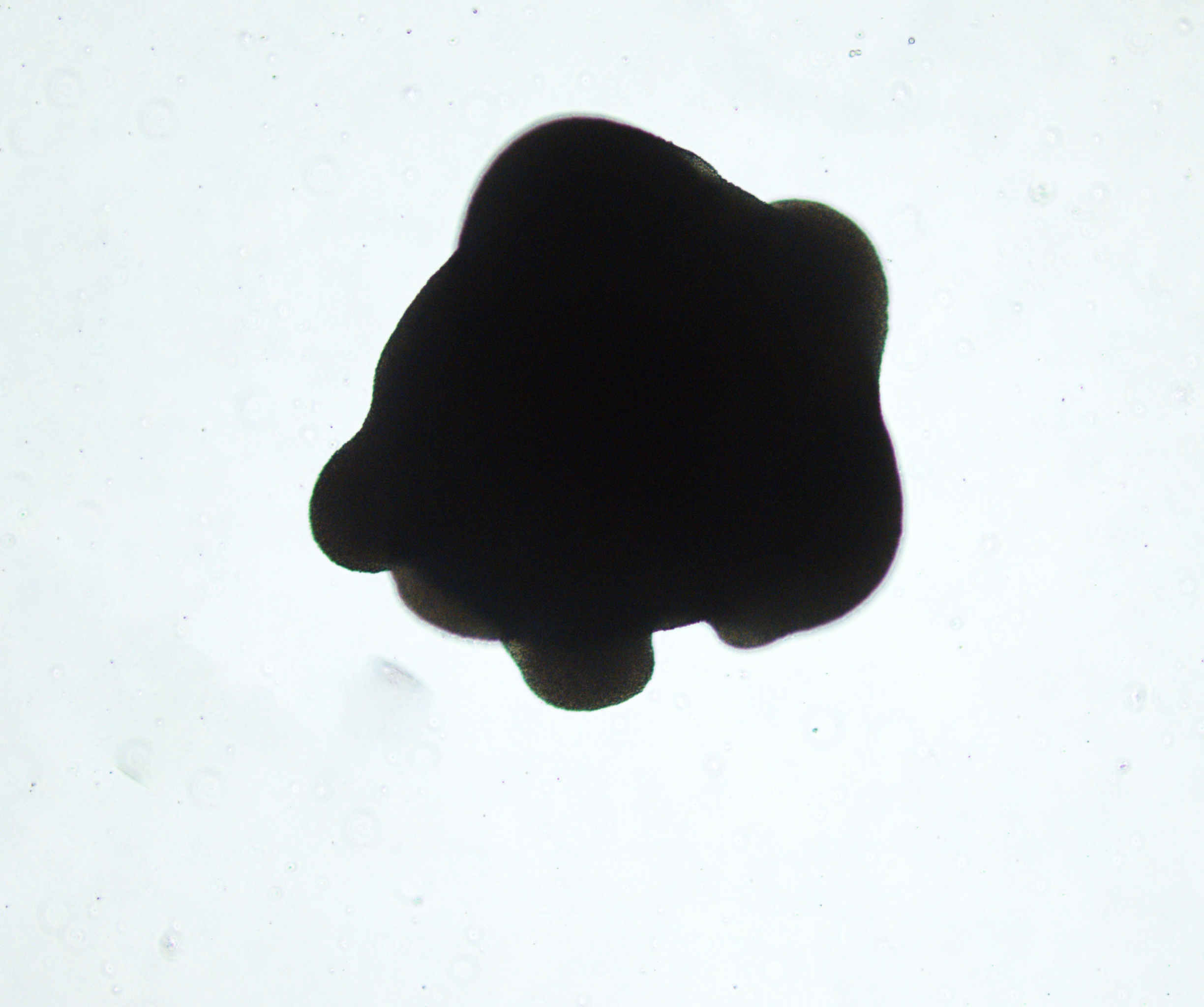

Supplement: Supplementary file 11 — Figure EV3 Source Data [file 44321_2025_302_MOESM11_ESM.zip › Figure EV3/EV3A/Day35_10-6.jpeg]

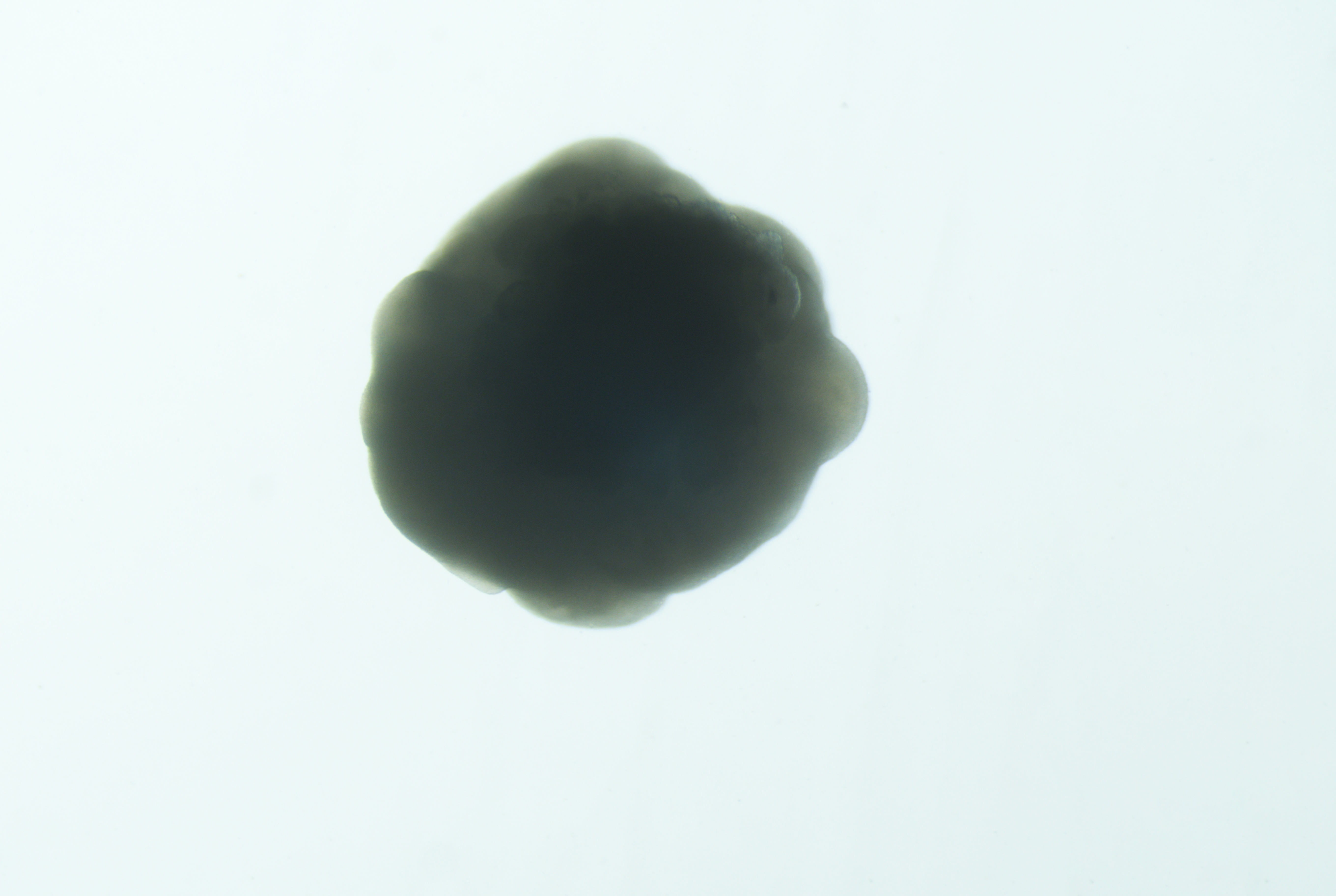

Supplement: Supplementary file 11 — Figure EV3 Source Data [file 44321_2025_302_MOESM11_ESM.zip › Figure EV3/EV3A/Day50_10-6.jpeg]

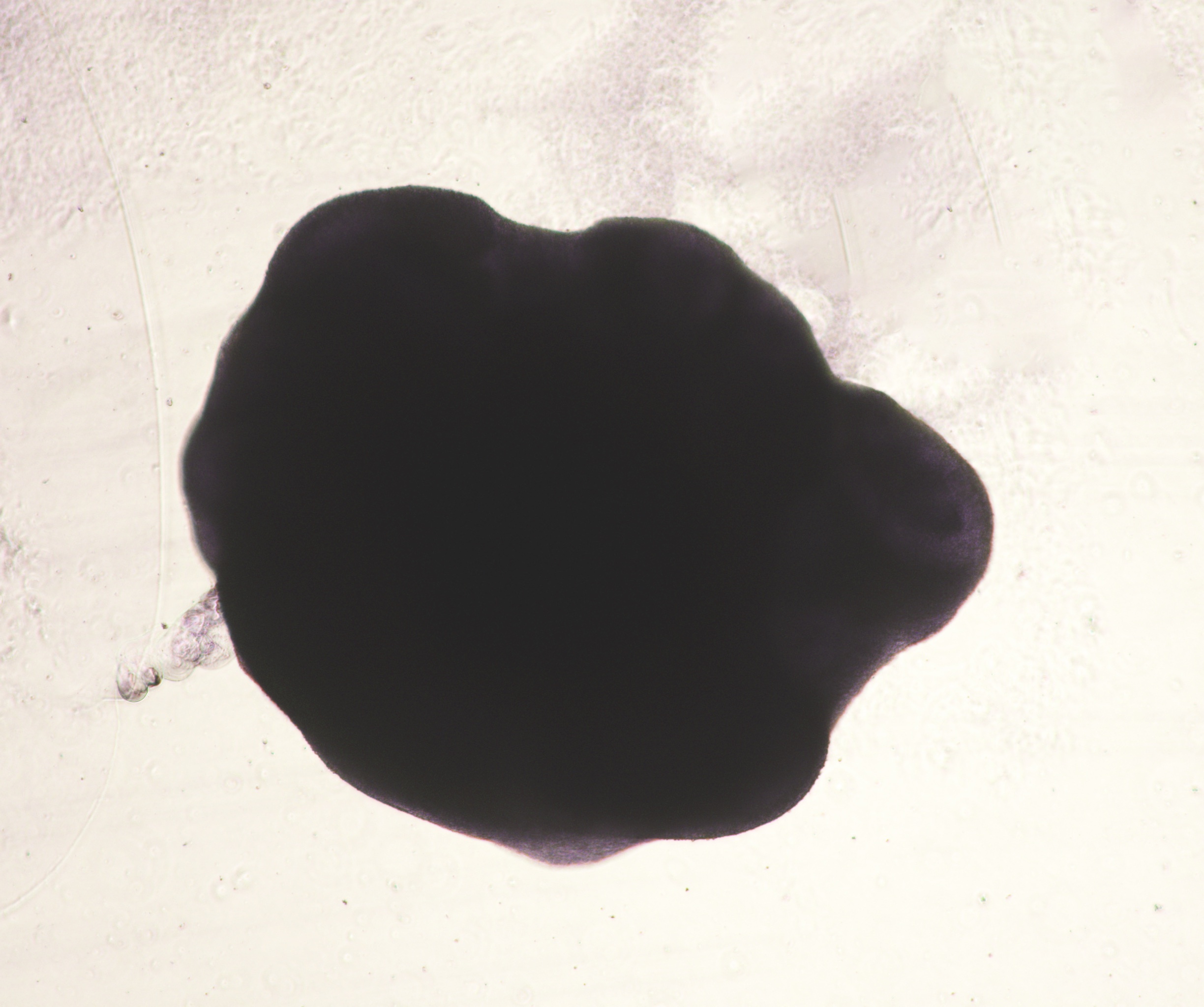

Supplement: Supplementary file 11 — Figure EV3 Source Data [file 44321_2025_302_MOESM11_ESM.zip › Figure EV3/EV3A/Day30_WT.jpeg]

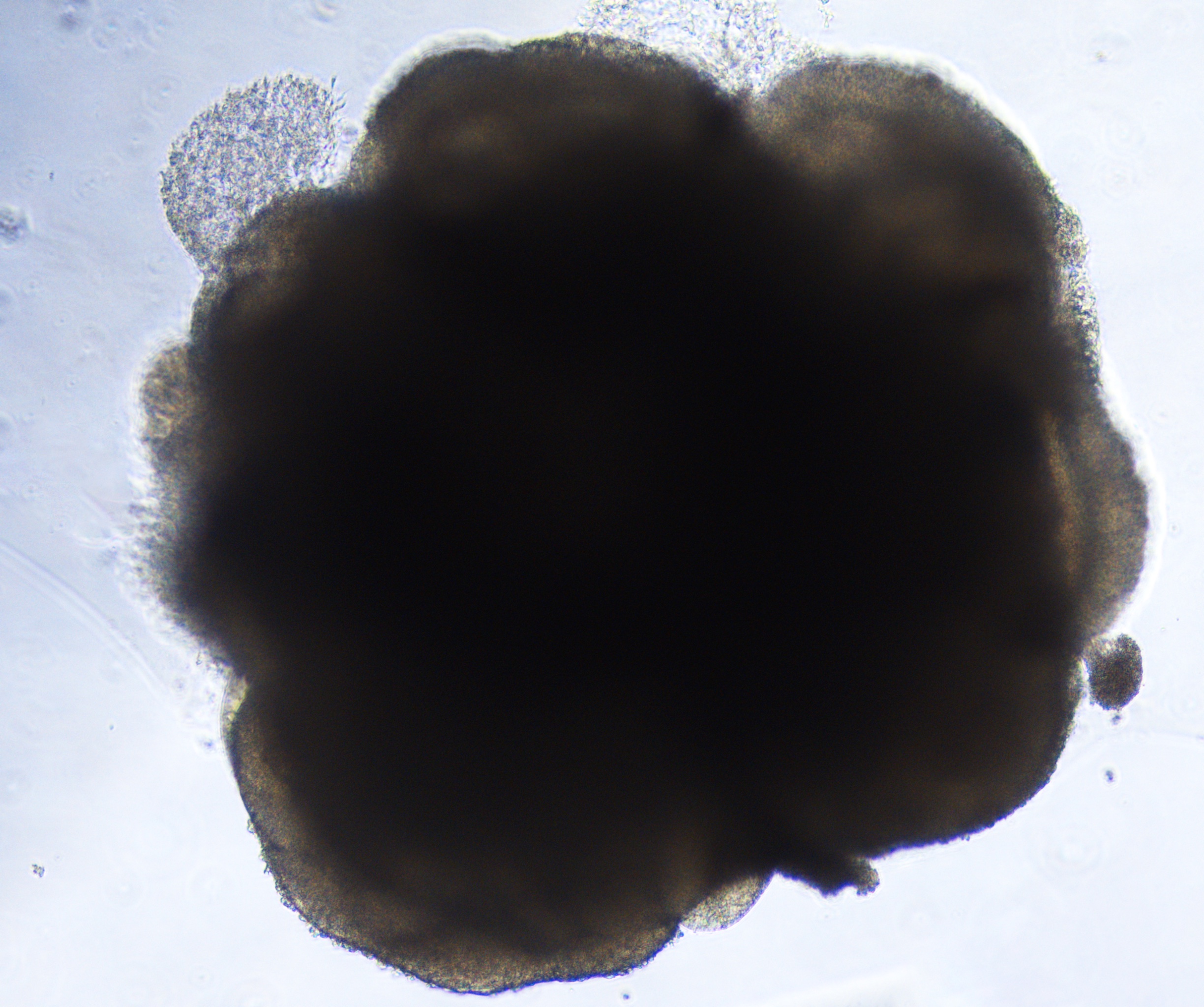

Supplement: Supplementary file 11 — Figure EV3 Source Data [file 44321_2025_302_MOESM11_ESM.zip › Figure EV3/EV3A/Day20_WT.jpeg]

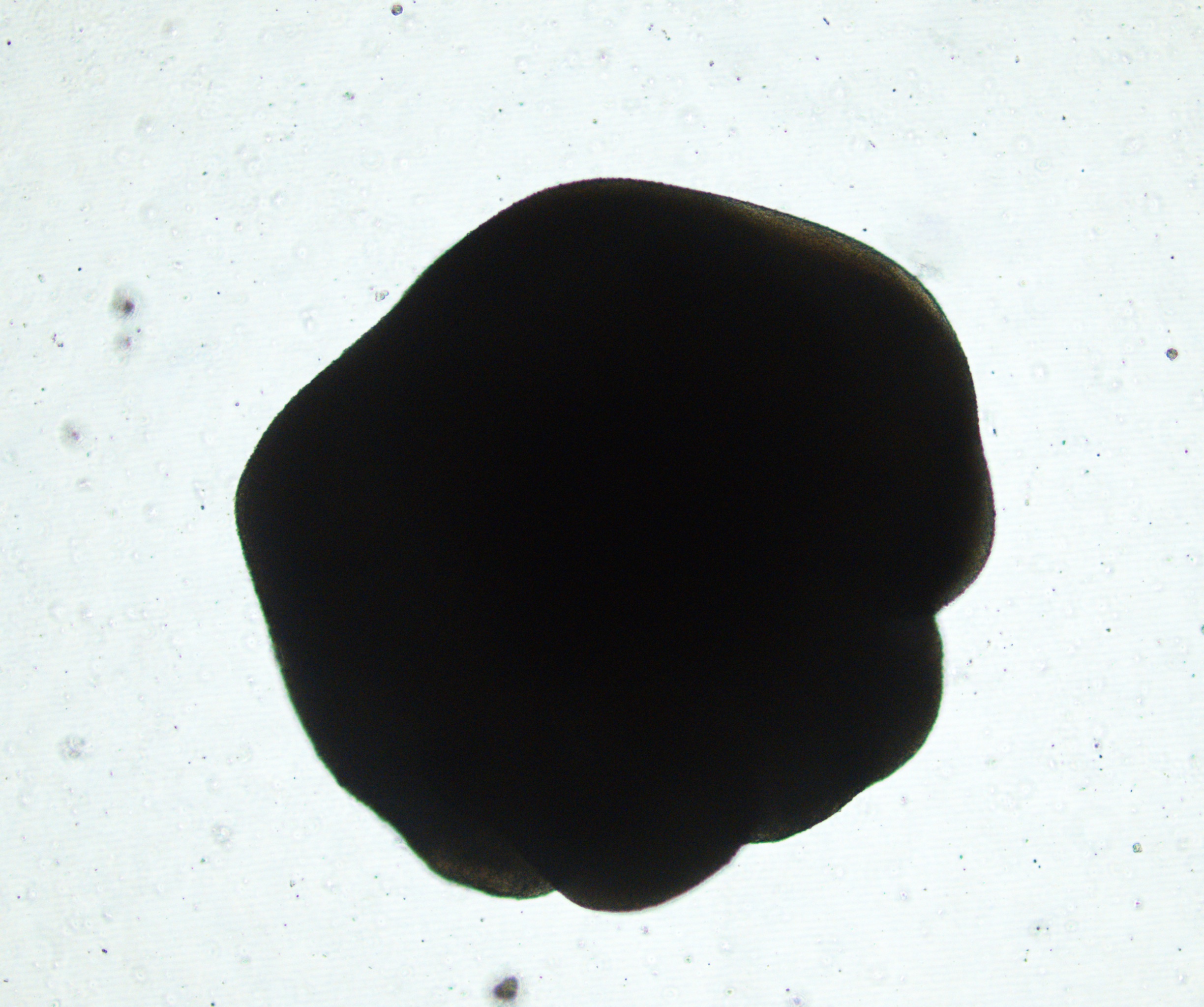

Supplement: Supplementary file 11 — Figure EV3 Source Data [file 44321_2025_302_MOESM11_ESM.zip › Figure EV3/EV3A/Day35_4-1.jpeg]

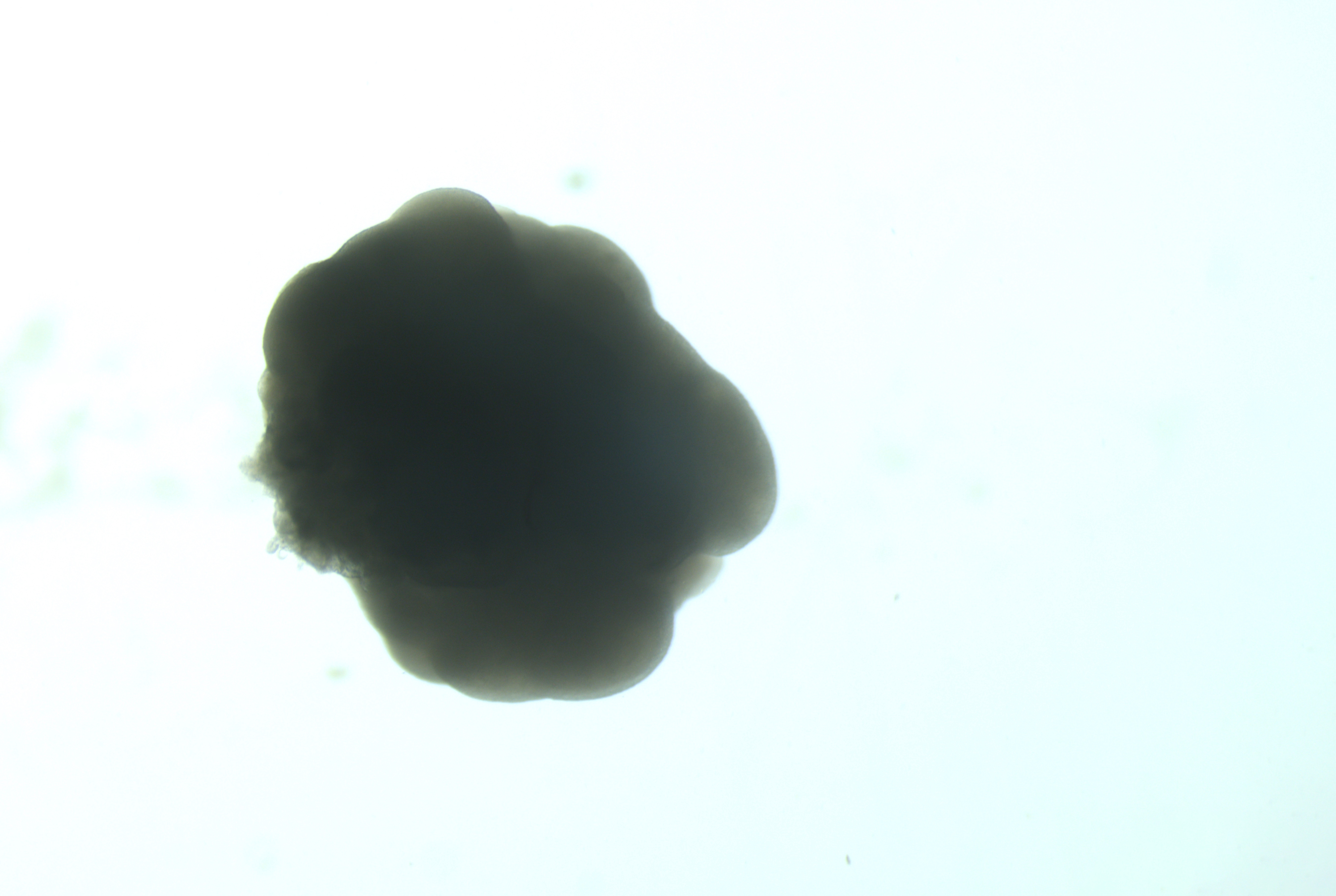

Supplement: Supplementary file 11 — Figure EV3 Source Data [file 44321_2025_302_MOESM11_ESM.zip › Figure EV3/EV3A/Day50_4-1.jpeg]

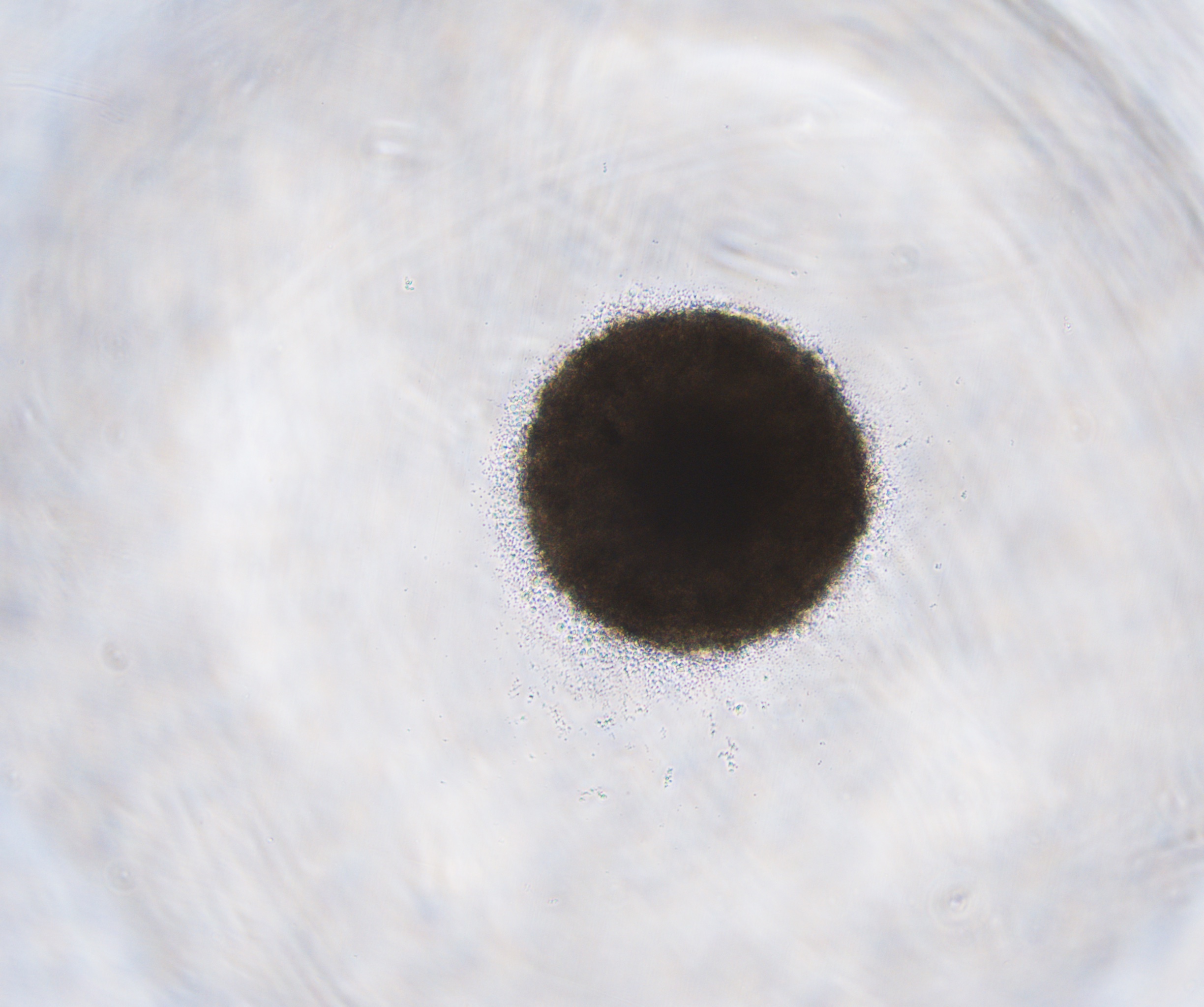

Supplement: Supplementary file 11 — Figure EV3 Source Data [file 44321_2025_302_MOESM11_ESM.zip › Figure EV3/EV3A/Day3_10-6.jpeg]

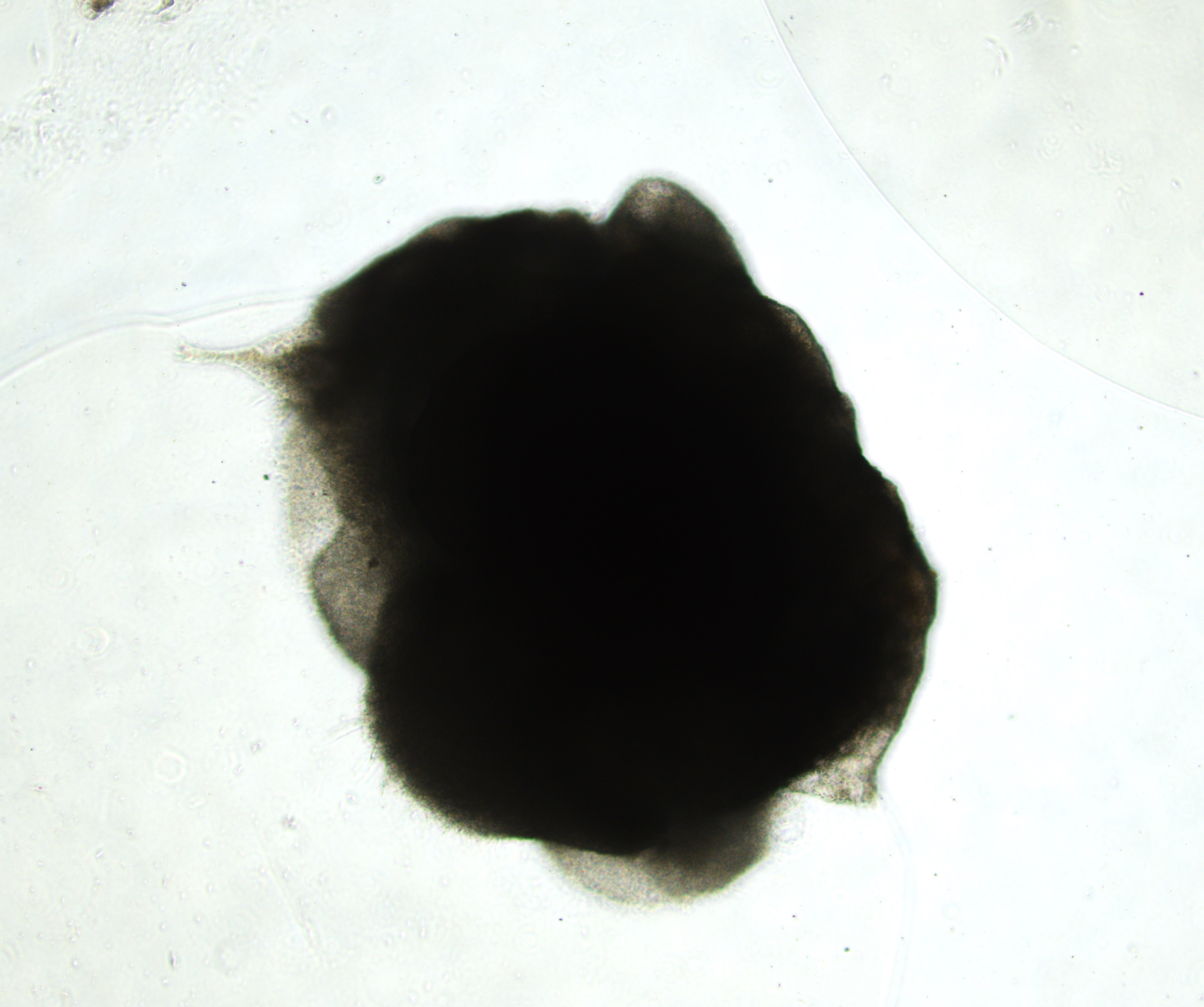

Supplement: Supplementary file 11 — Figure EV3 Source Data [file 44321_2025_302_MOESM11_ESM.zip › Figure EV3/EV3A/Day25_WT.jpeg]

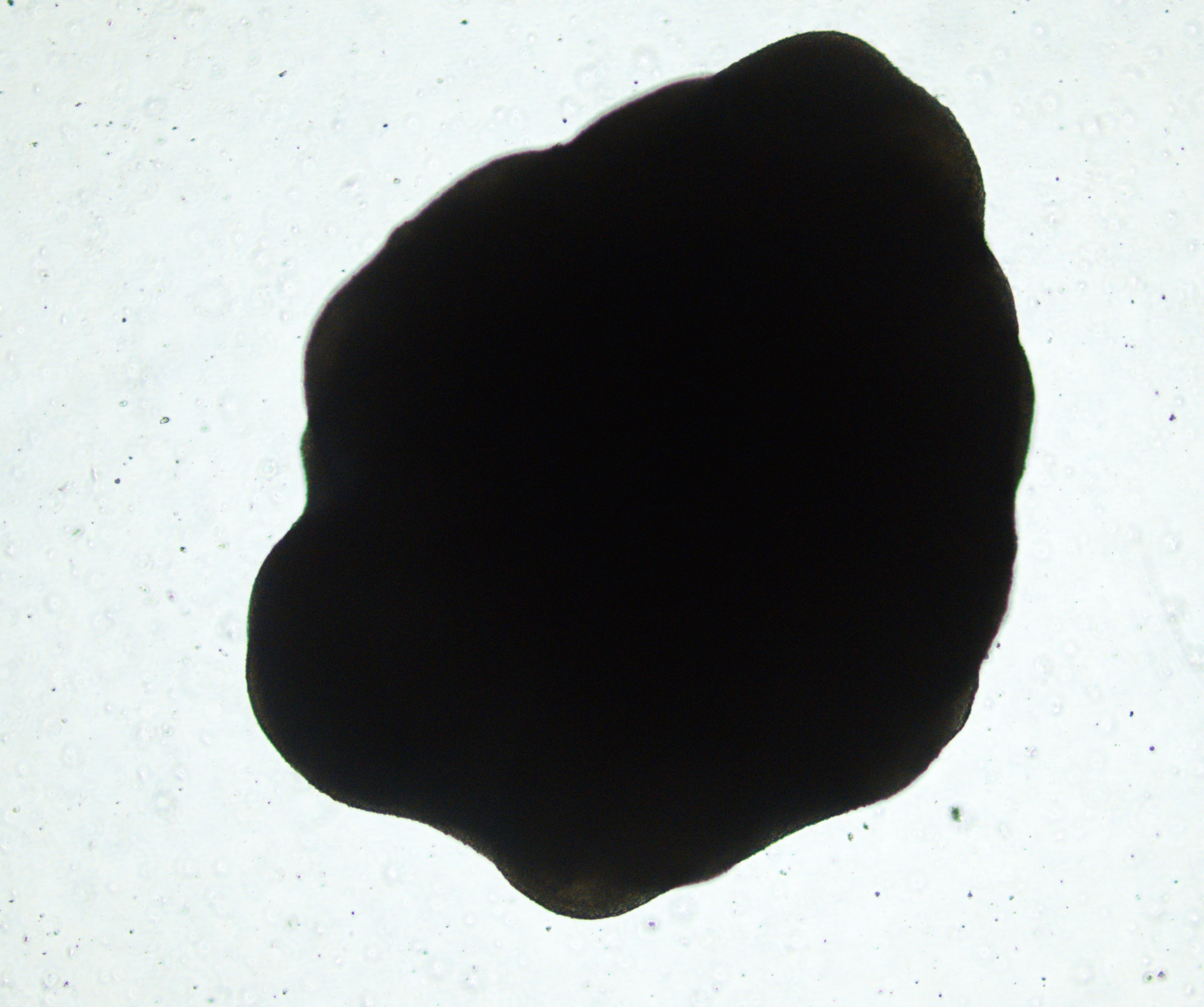

Supplement: Supplementary file 11 — Figure EV3 Source Data [file 44321_2025_302_MOESM11_ESM.zip › Figure EV3/EV3A/Day35_WT.jpeg]

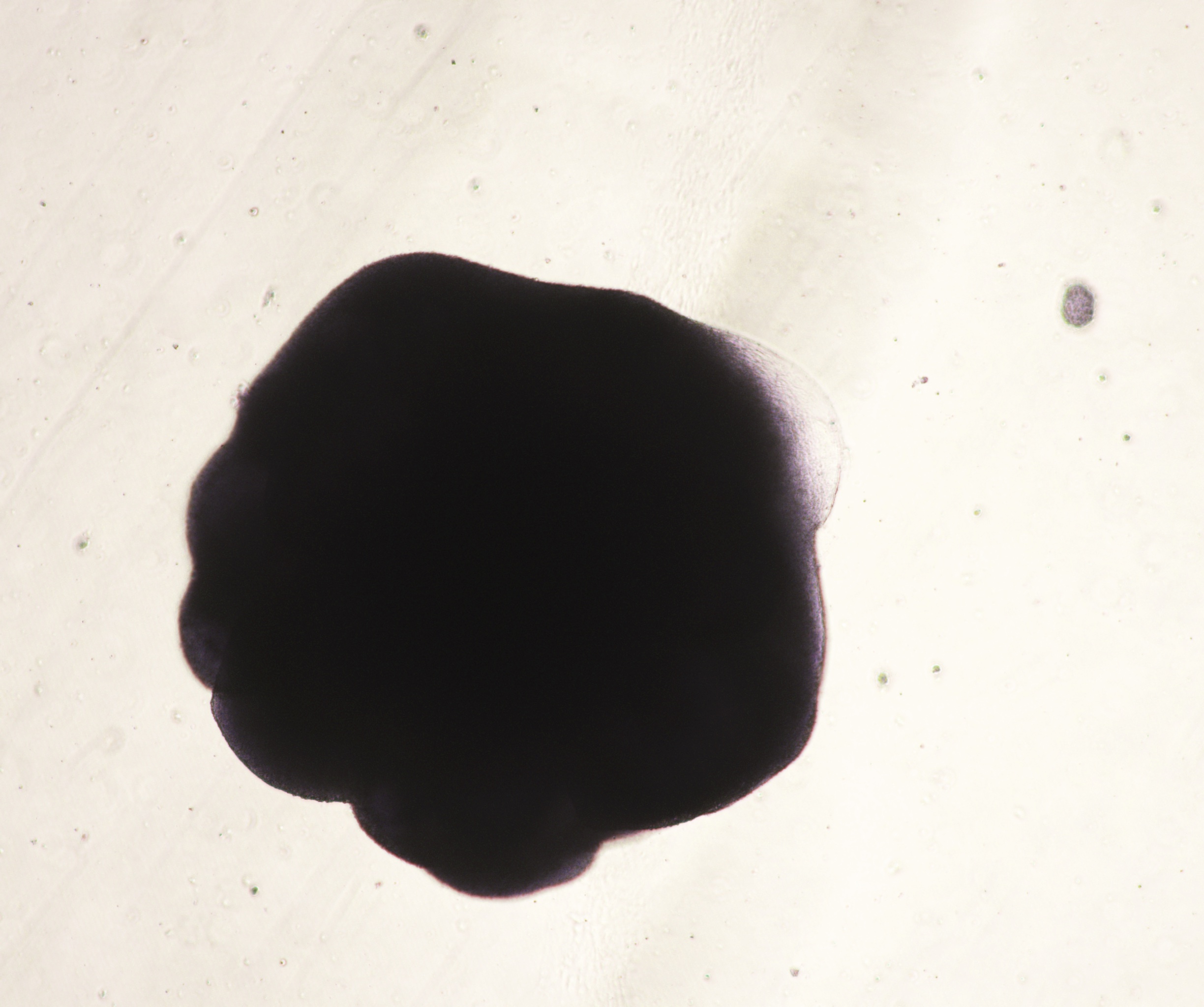

Supplement: Supplementary file 11 — Figure EV3 Source Data [file 44321_2025_302_MOESM11_ESM.zip › Figure EV3/EV3A/Day30_4-1.jpeg]

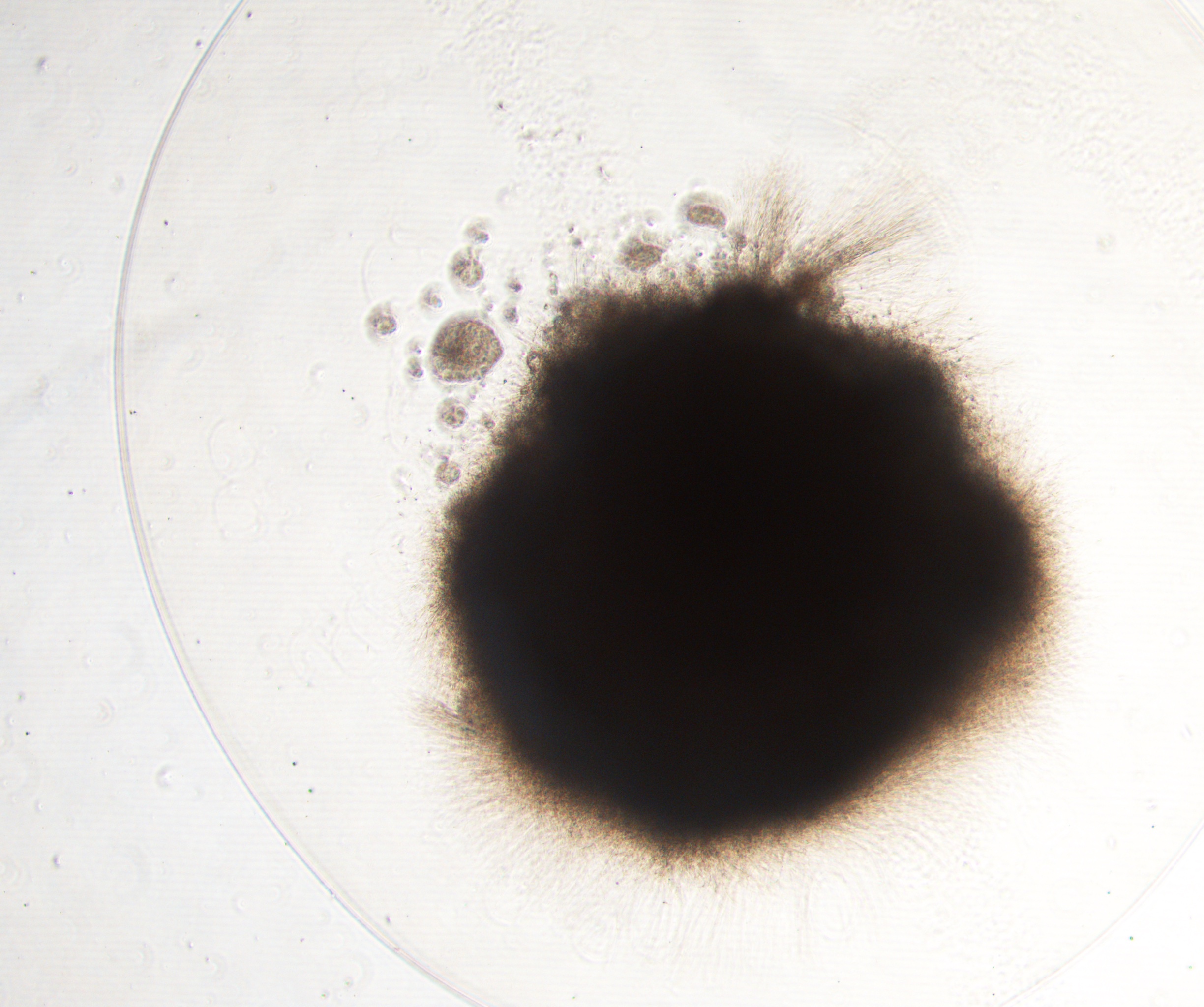

Supplement: Supplementary file 11 — Figure EV3 Source Data [file 44321_2025_302_MOESM11_ESM.zip › Figure EV3/EV3A/Day30_10-6.jpeg]

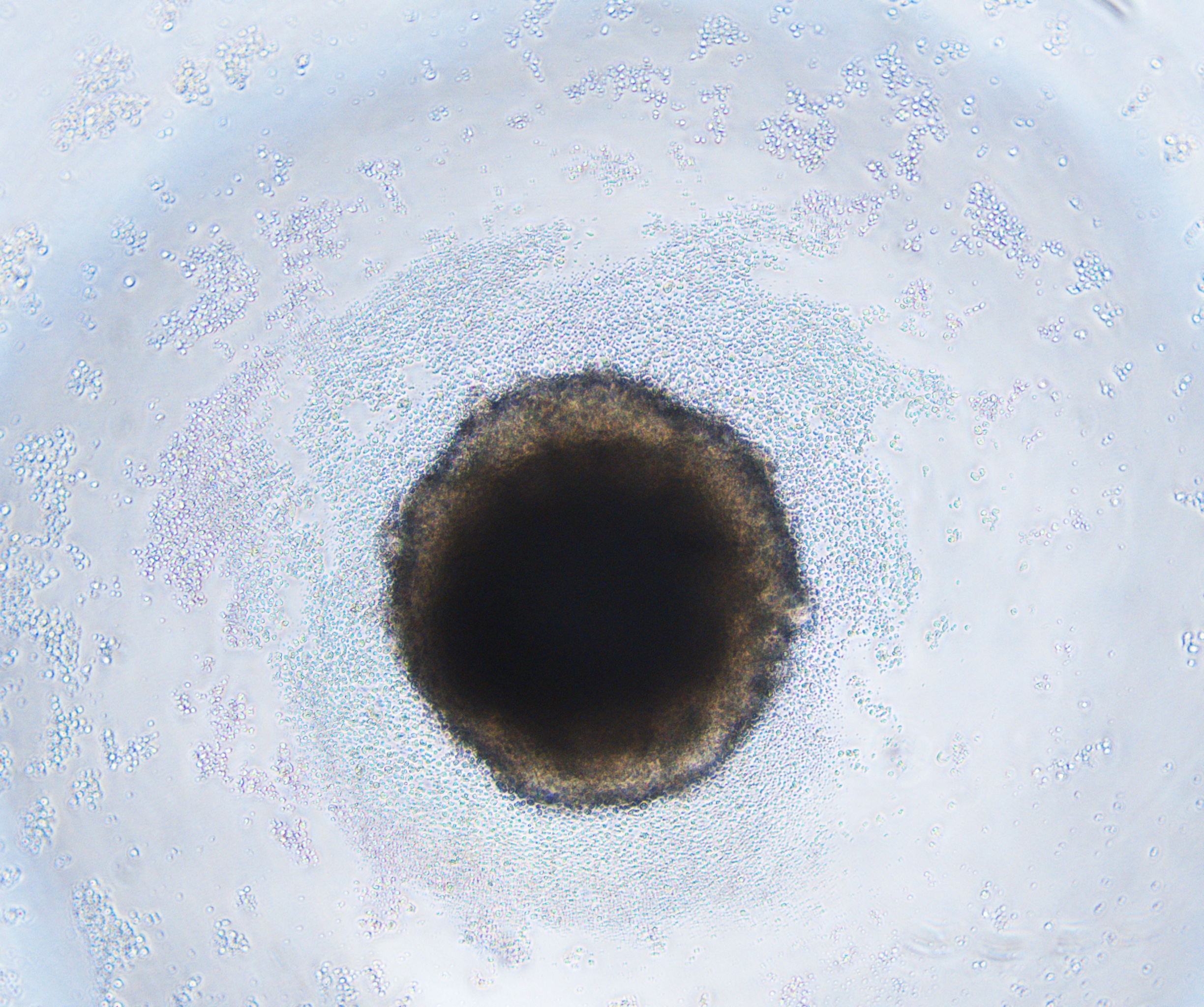

Supplement: Supplementary file 11 — Figure EV3 Source Data [file 44321_2025_302_MOESM11_ESM.zip › Figure EV3/EV3A/Day10_WT.jpeg]

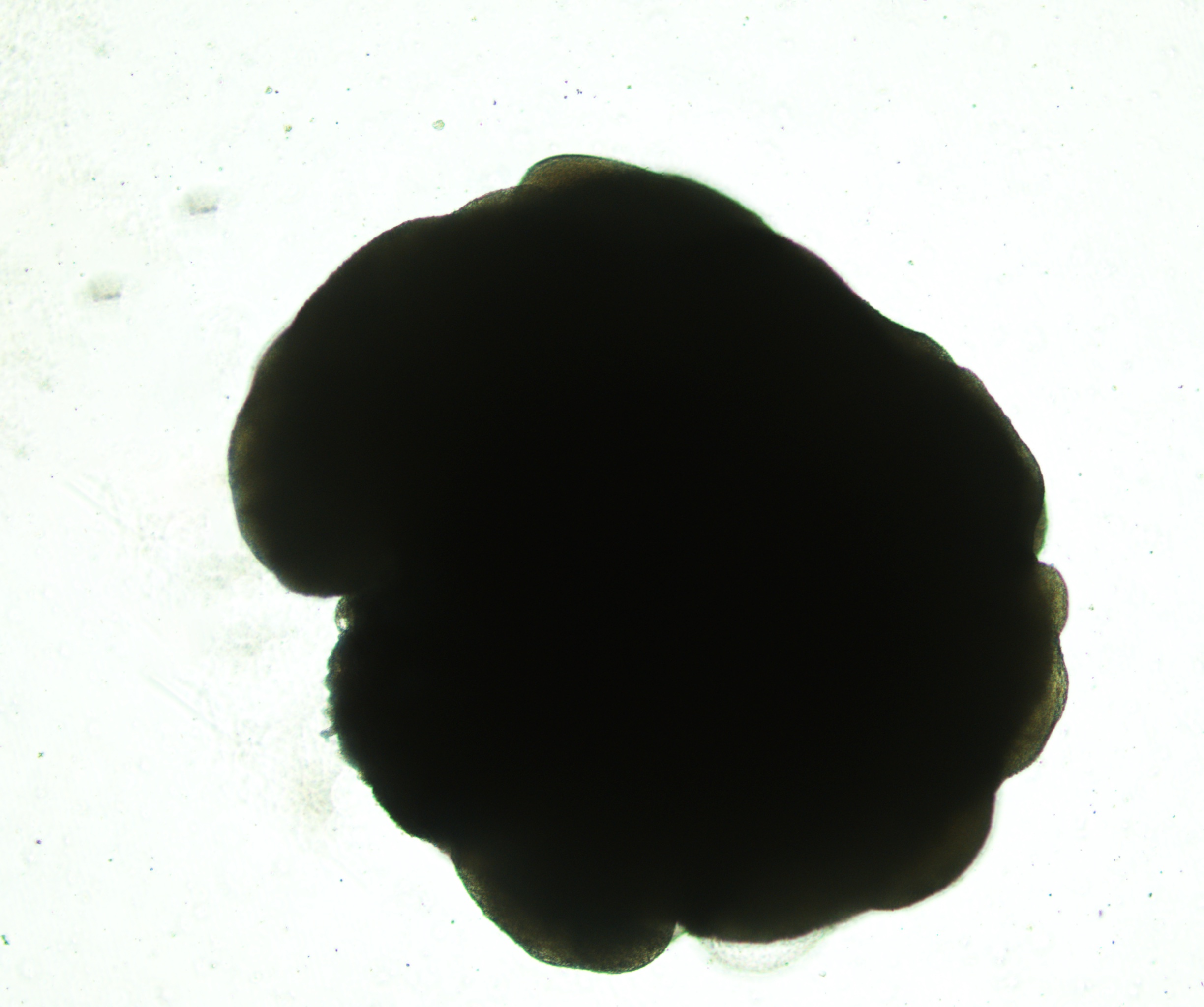

Supplement: Supplementary file 11 — Figure EV3 Source Data [file 44321_2025_302_MOESM11_ESM.zip › Figure EV3/EV3A/Day40_4-1.jpeg]
