# Supplementary figures and images for: CETN3 deficiency induces microcephaly by disrupting neural stem/progenitor cell fate through impaired centrosome assembly and RNA splicing (part 4 of 5)
Source: EMBO Mol Med. 2025 Sep 8;17(10):2735–61. doi: 10.1038/s44321-025-00302-7 (PMC12514221; doi:10.1038/s44321-025-00302-7)

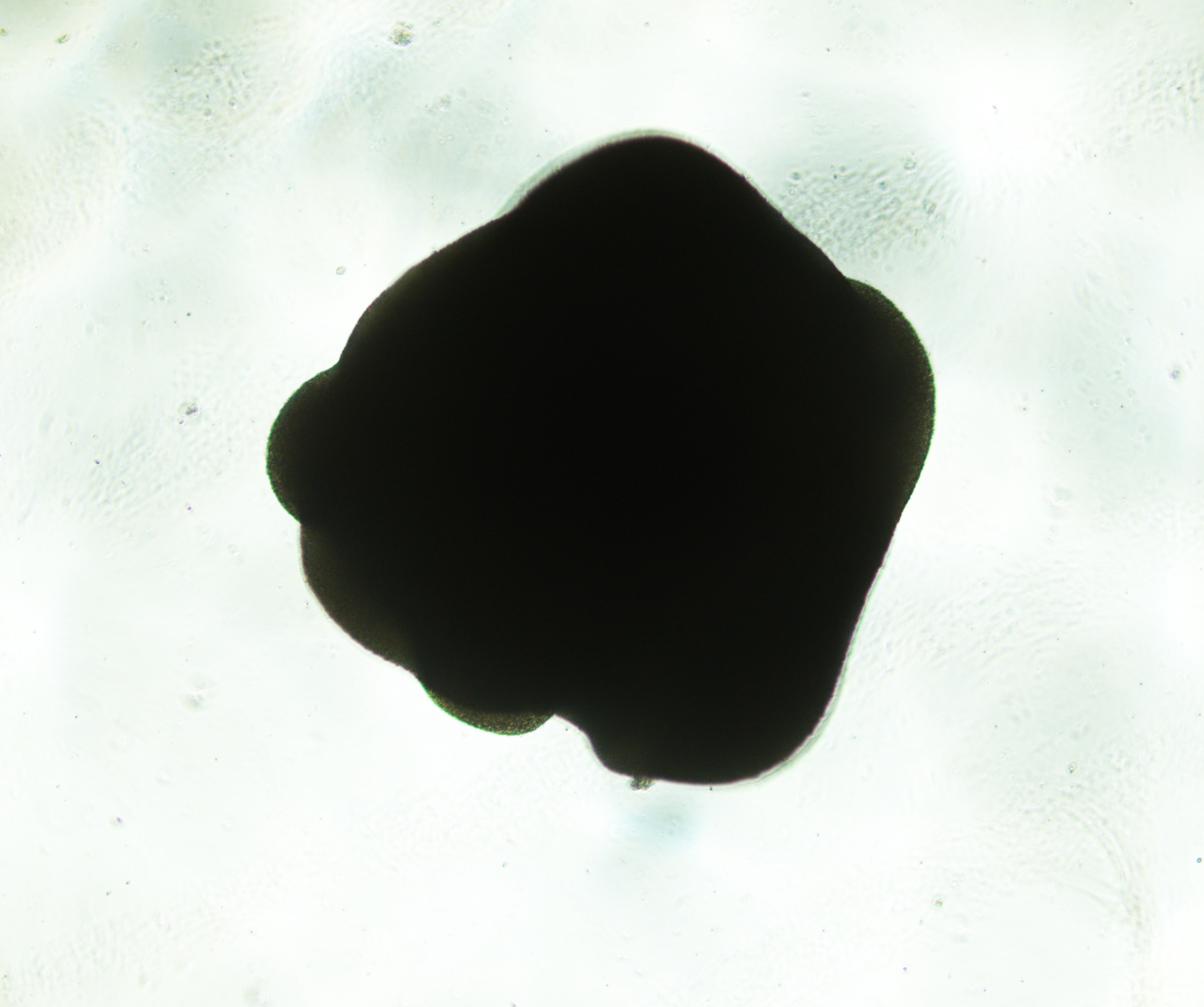

Supplement: Supplementary file 11 — Figure EV3 Source Data [file 44321_2025_302_MOESM11_ESM.zip › Figure EV3/EV3A/Day40_10-6.jpeg]

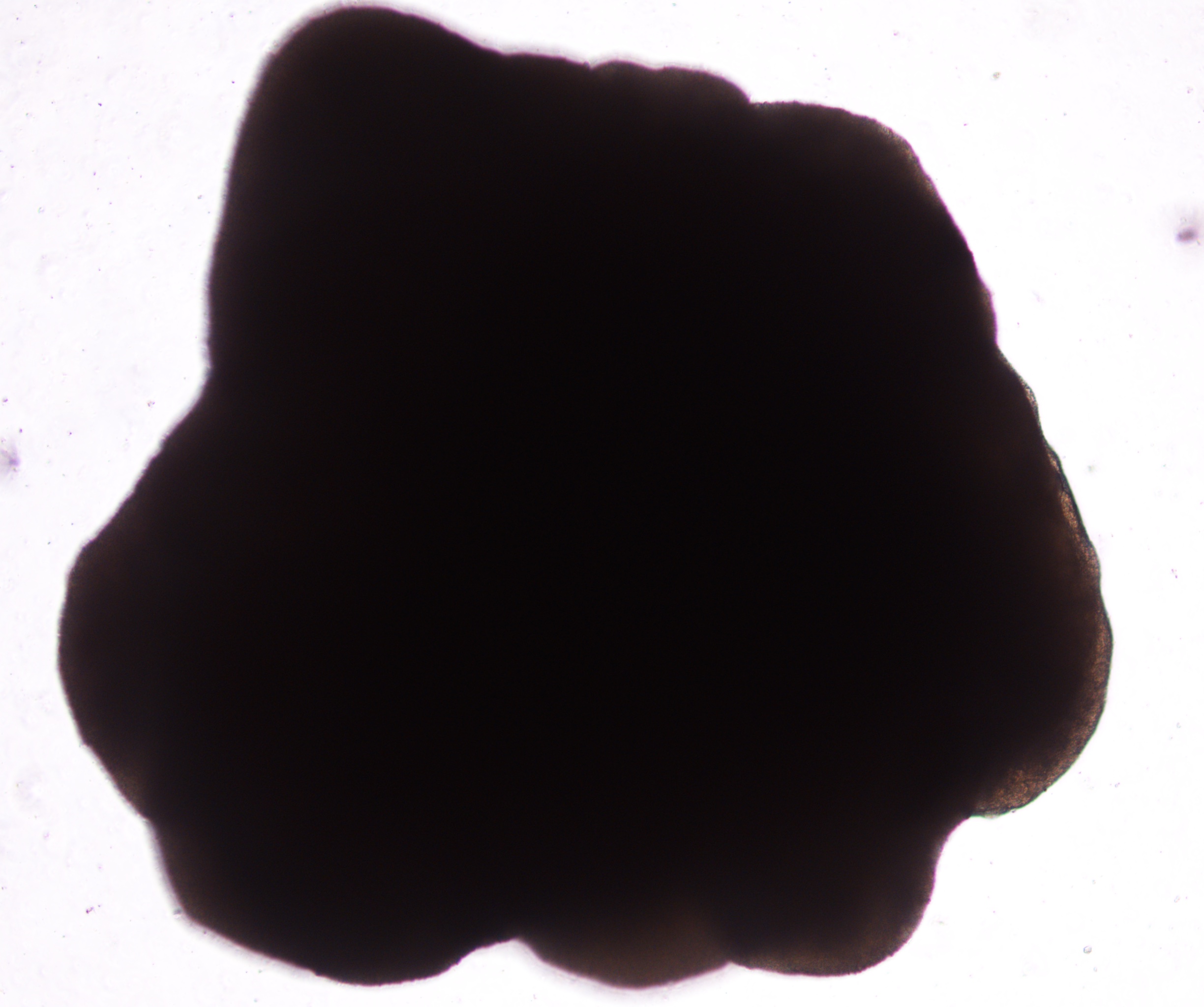

Supplement: Supplementary file 11 — Figure EV3 Source Data [file 44321_2025_302_MOESM11_ESM.zip › Figure EV3/EV3A/Day45_WT.jpeg]

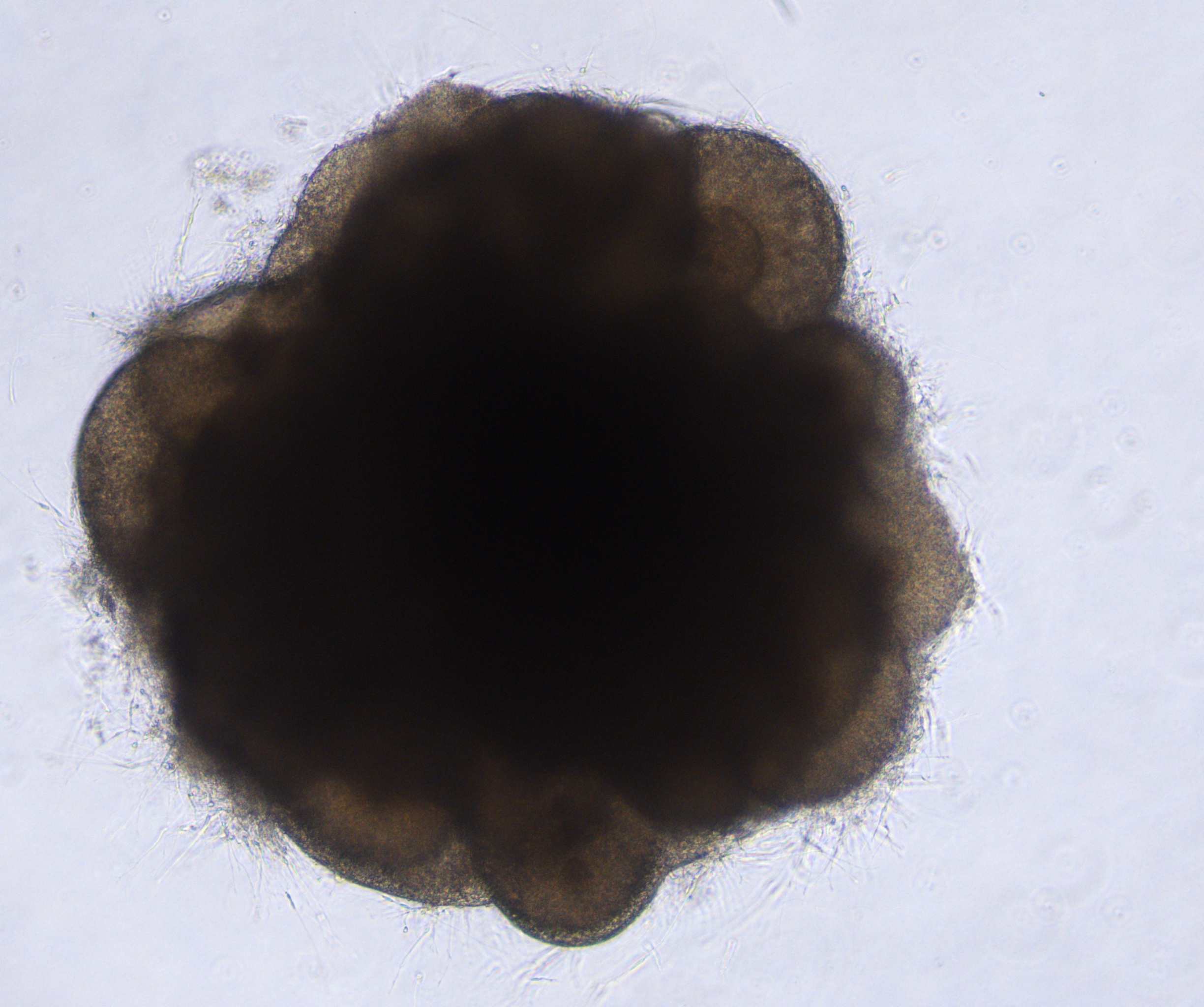

Supplement: Supplementary file 11 — Figure EV3 Source Data [file 44321_2025_302_MOESM11_ESM.zip › Figure EV3/EV3A/Day20_4-1.jpeg]

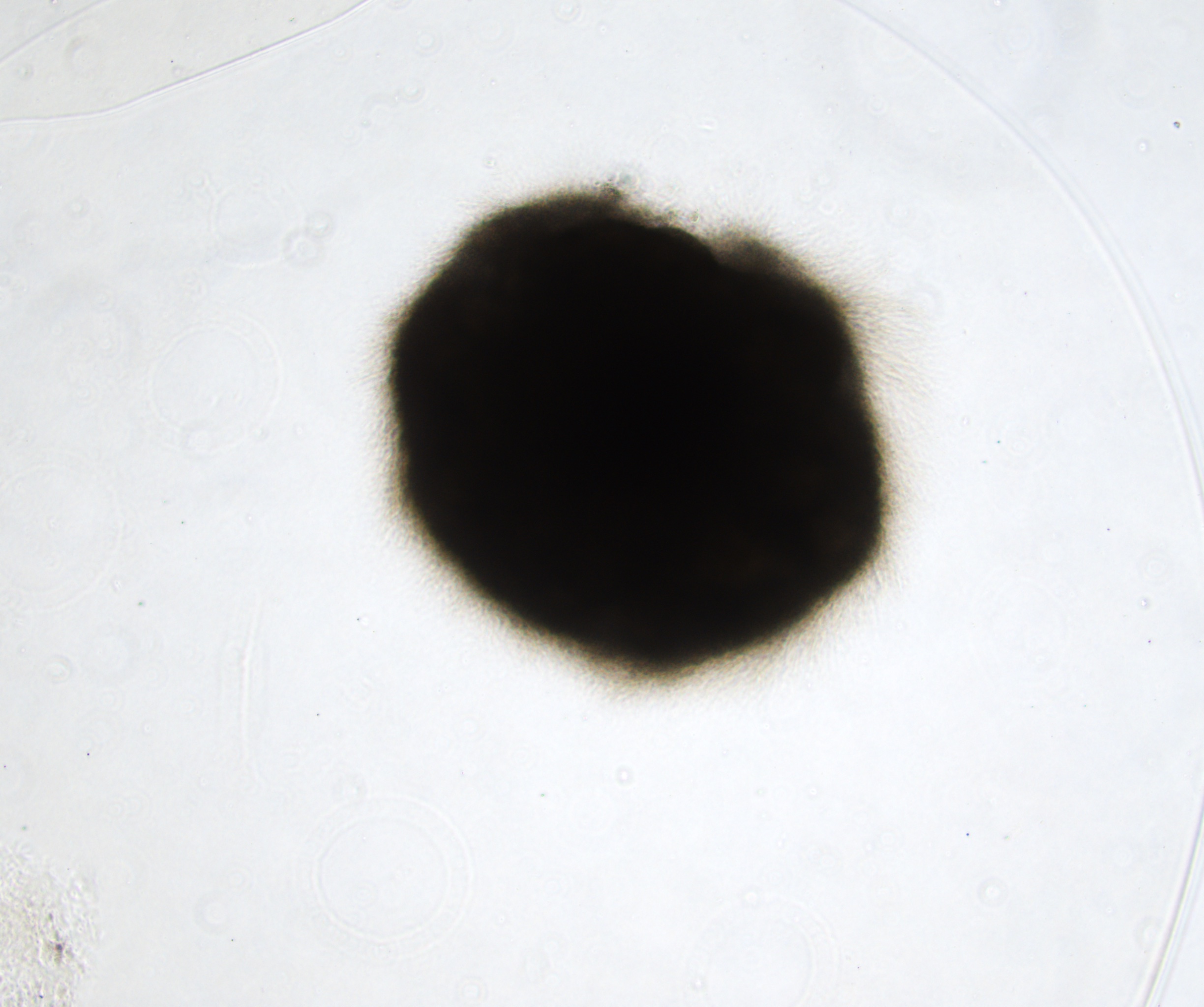

Supplement: Supplementary file 11 — Figure EV3 Source Data [file 44321_2025_302_MOESM11_ESM.zip › Figure EV3/EV3A/Day25_10-6.jpeg]

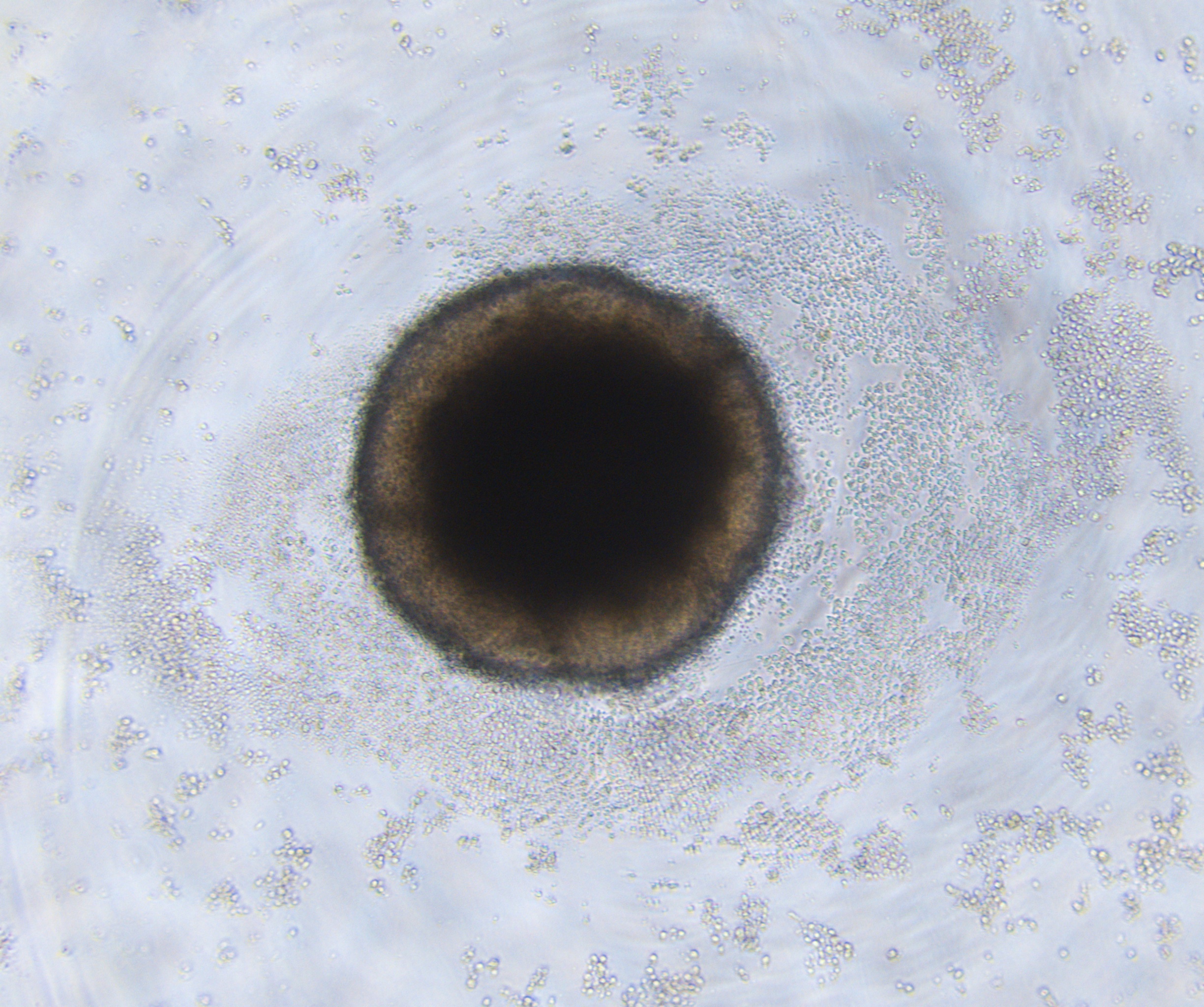

Supplement: Supplementary file 11 — Figure EV3 Source Data [file 44321_2025_302_MOESM11_ESM.zip › Figure EV3/EV3A/Day10_10-6.jpeg]

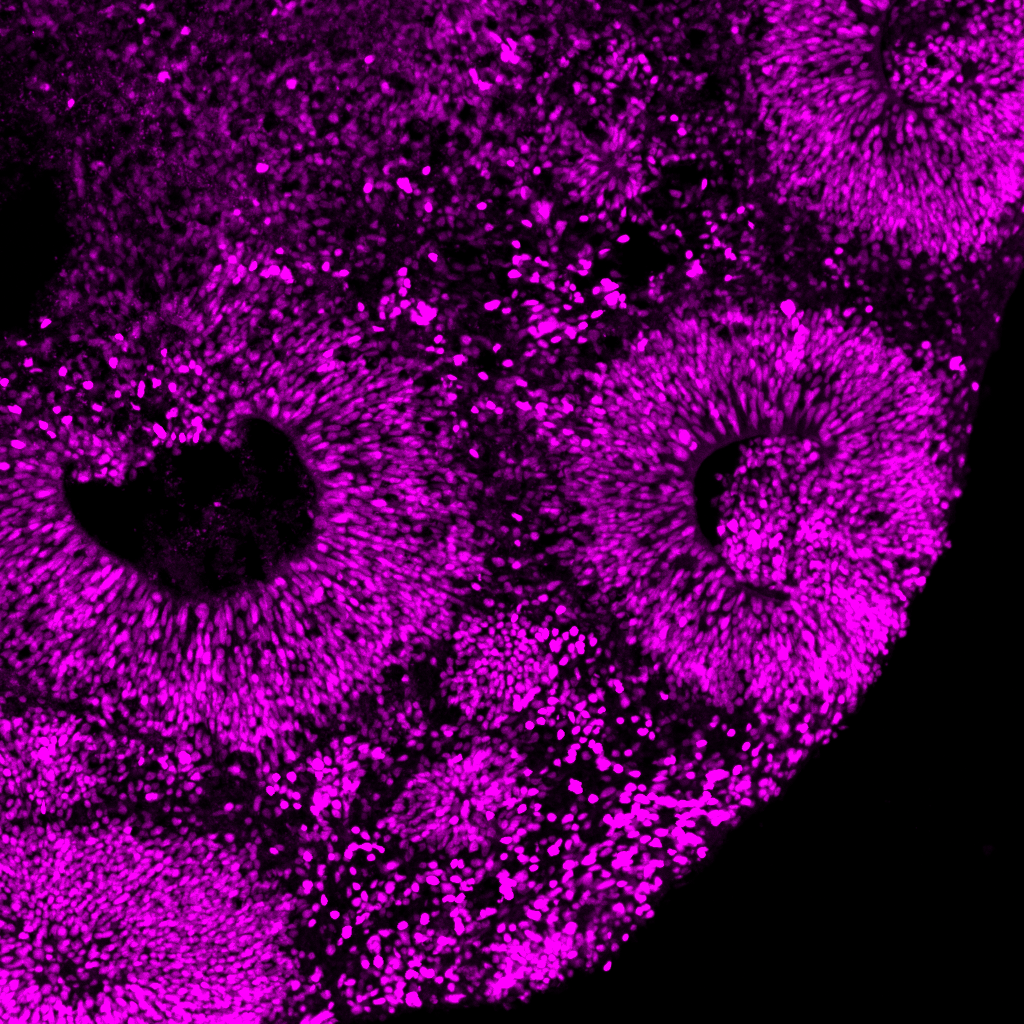

Supplement: Supplementary file 11 — Figure EV3 Source Data [file 44321_2025_302_MOESM11_ESM.zip › Figure EV3/EV3D/WT_PAX6.tif]

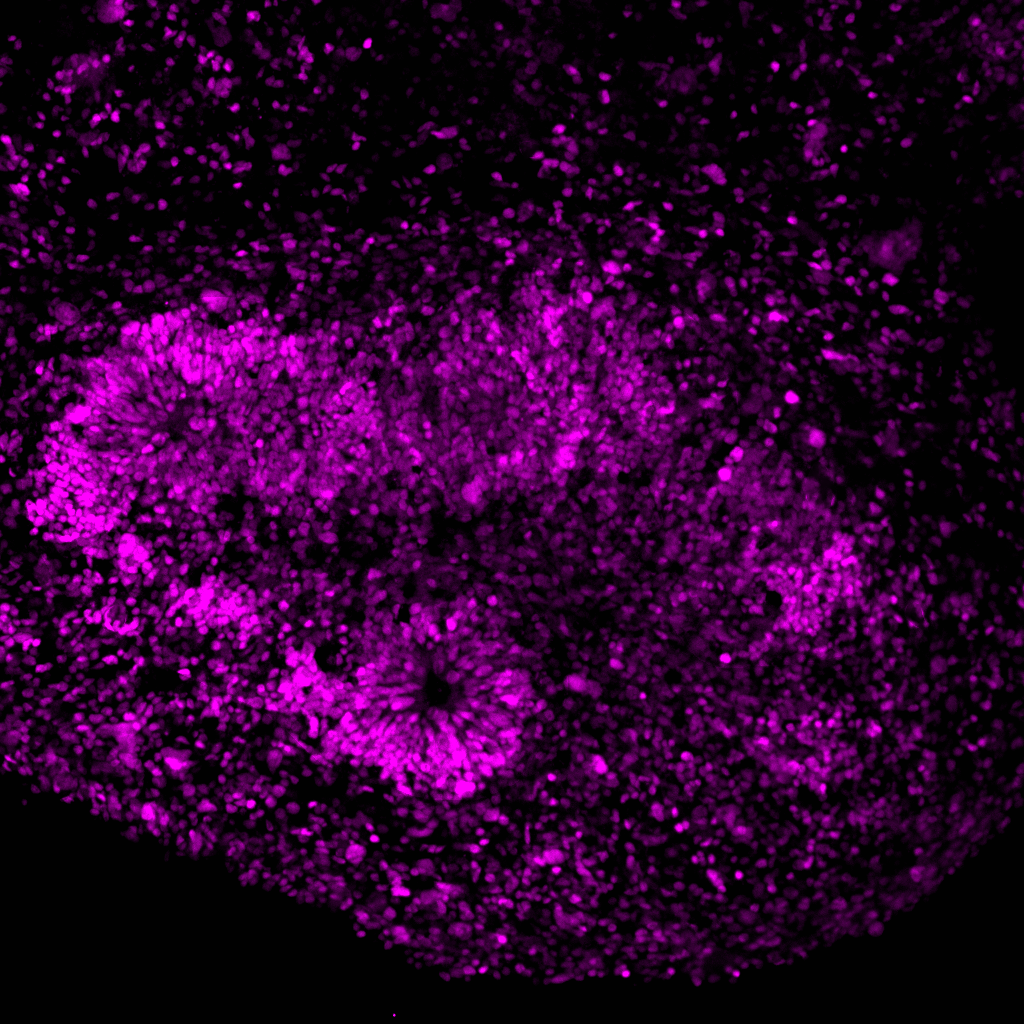

Supplement: Supplementary file 11 — Figure EV3 Source Data [file 44321_2025_302_MOESM11_ESM.zip › Figure EV3/EV3D/#4-1_PAX6.tif]

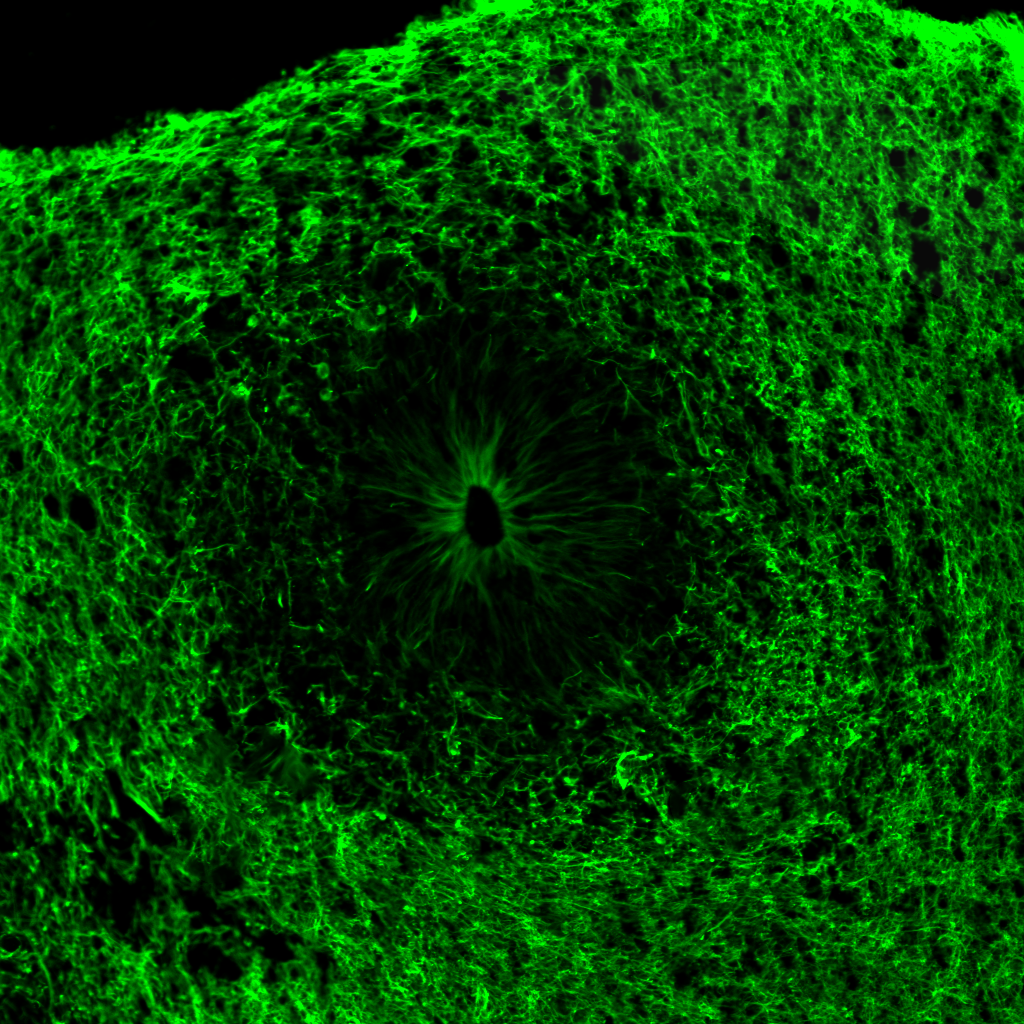

Supplement: Supplementary file 11 — Figure EV3 Source Data [file 44321_2025_302_MOESM11_ESM.zip › Figure EV3/EV3D/#10-6_TUJ1.tif]

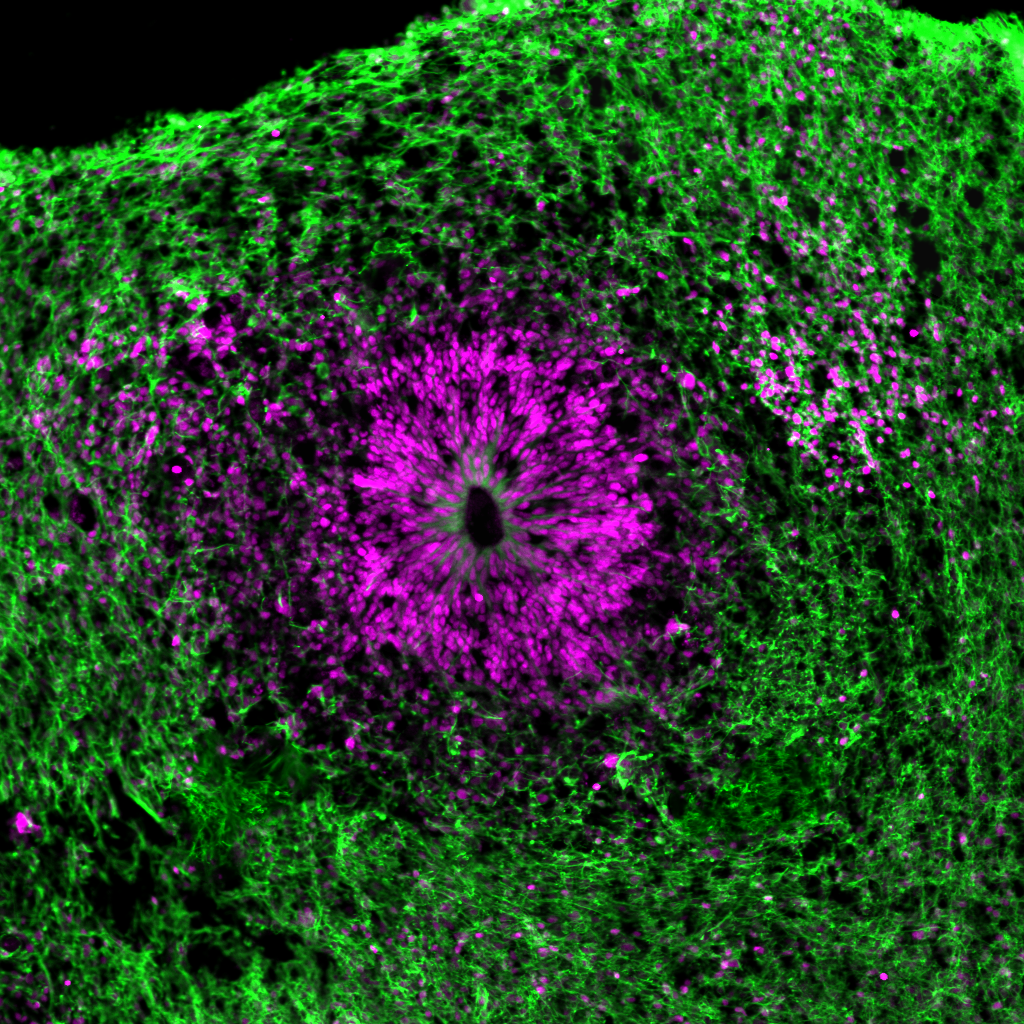

Supplement: Supplementary file 11 — Figure EV3 Source Data [file 44321_2025_302_MOESM11_ESM.zip › Figure EV3/EV3D/#10-6_merge.tif]

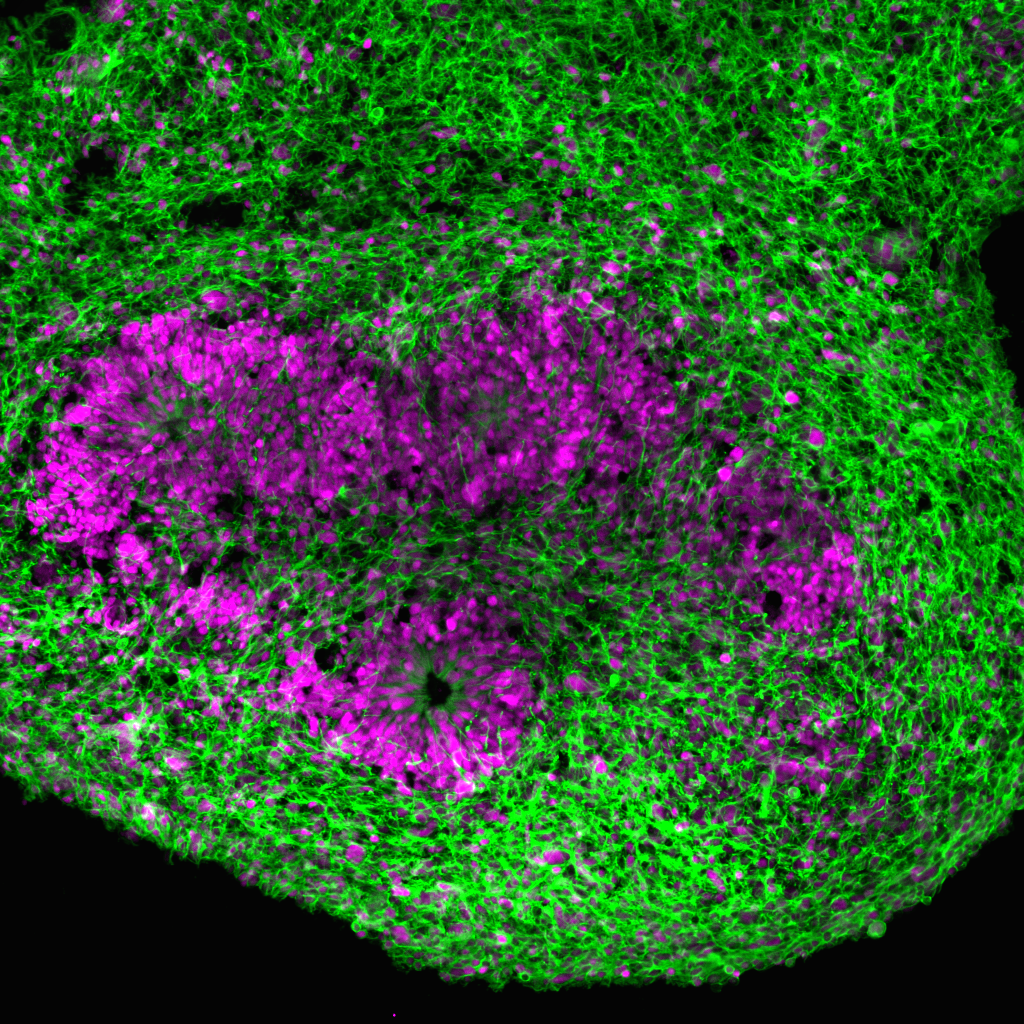

Supplement: Supplementary file 11 — Figure EV3 Source Data [file 44321_2025_302_MOESM11_ESM.zip › Figure EV3/EV3D/#4-1_merge.tif]

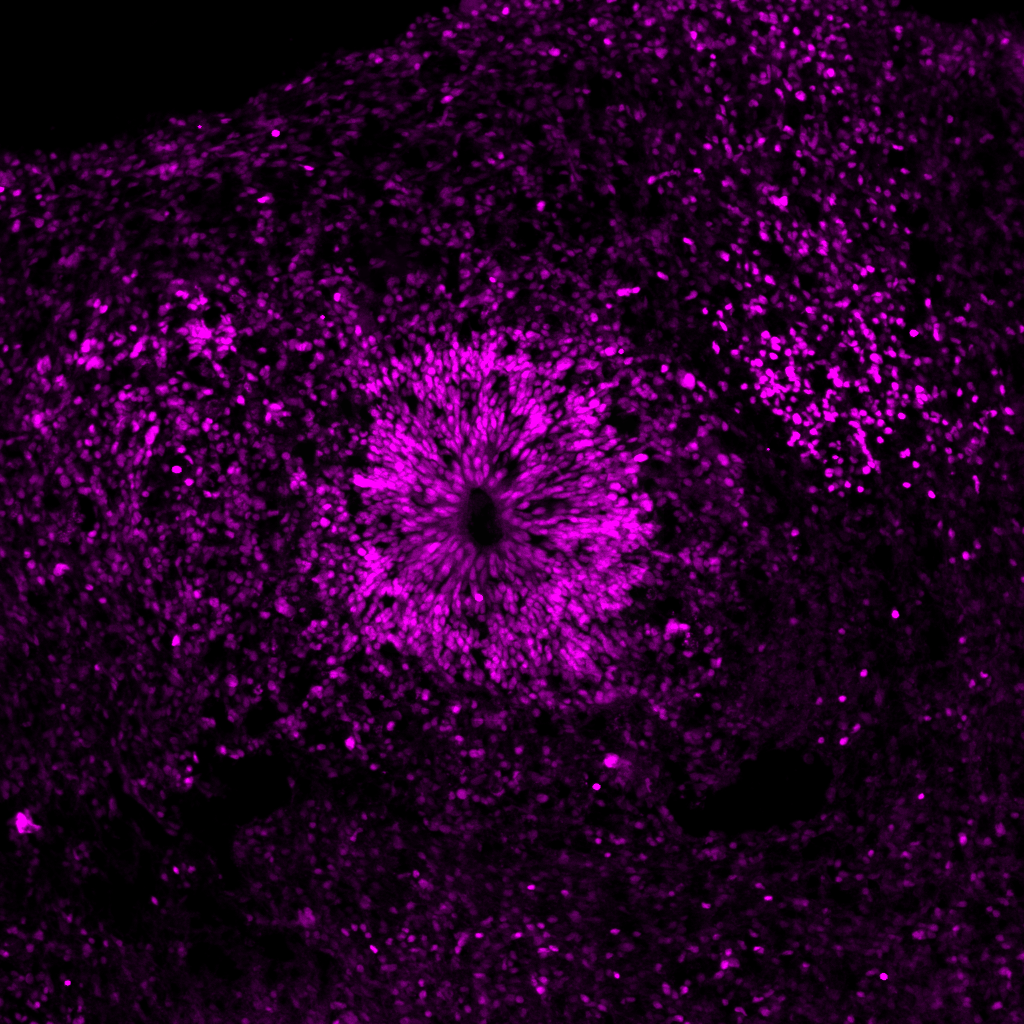

Supplement: Supplementary file 11 — Figure EV3 Source Data [file 44321_2025_302_MOESM11_ESM.zip › Figure EV3/EV3D/#10-6_PAX6.tif]

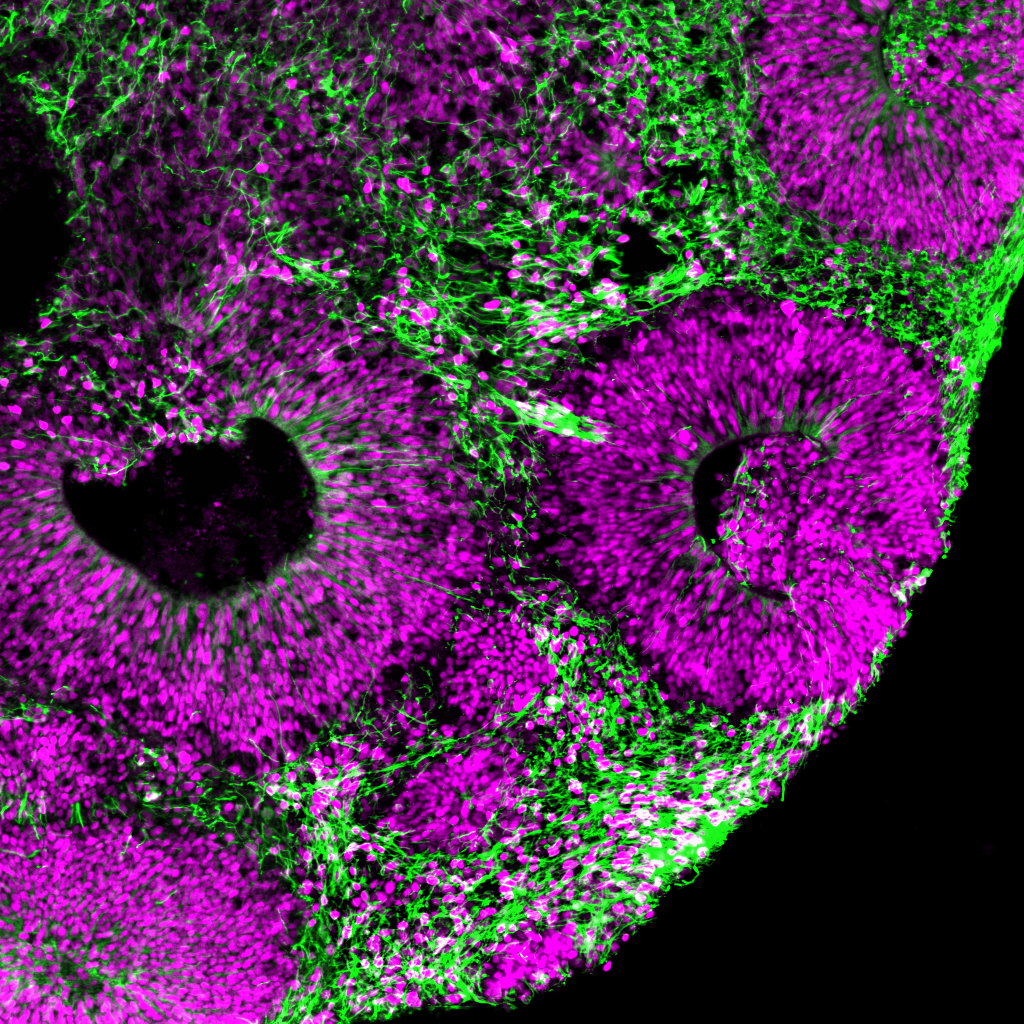

Supplement: Supplementary file 11 — Figure EV3 Source Data [file 44321_2025_302_MOESM11_ESM.zip › Figure EV3/EV3D/WT_merge.tif]

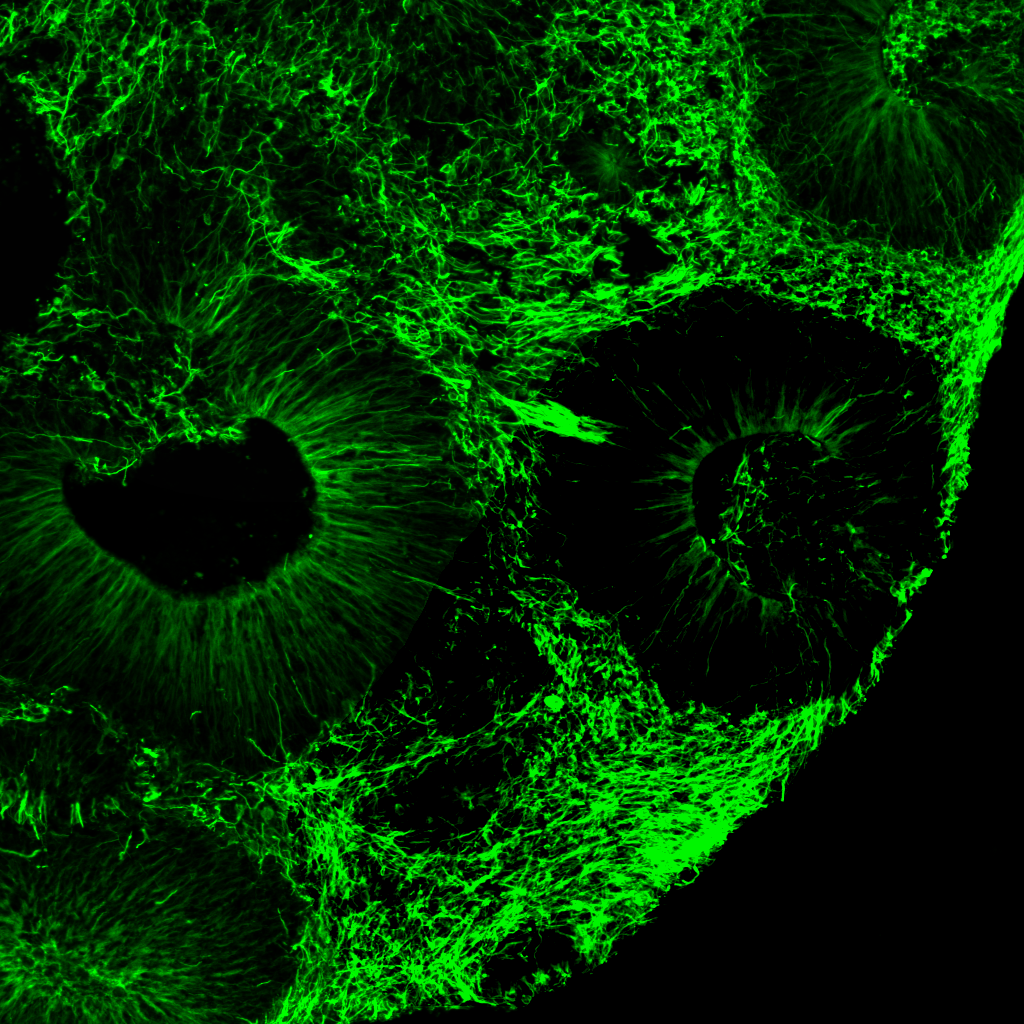

Supplement: Supplementary file 11 — Figure EV3 Source Data [file 44321_2025_302_MOESM11_ESM.zip › Figure EV3/EV3D/WT_TUJ1.tif]

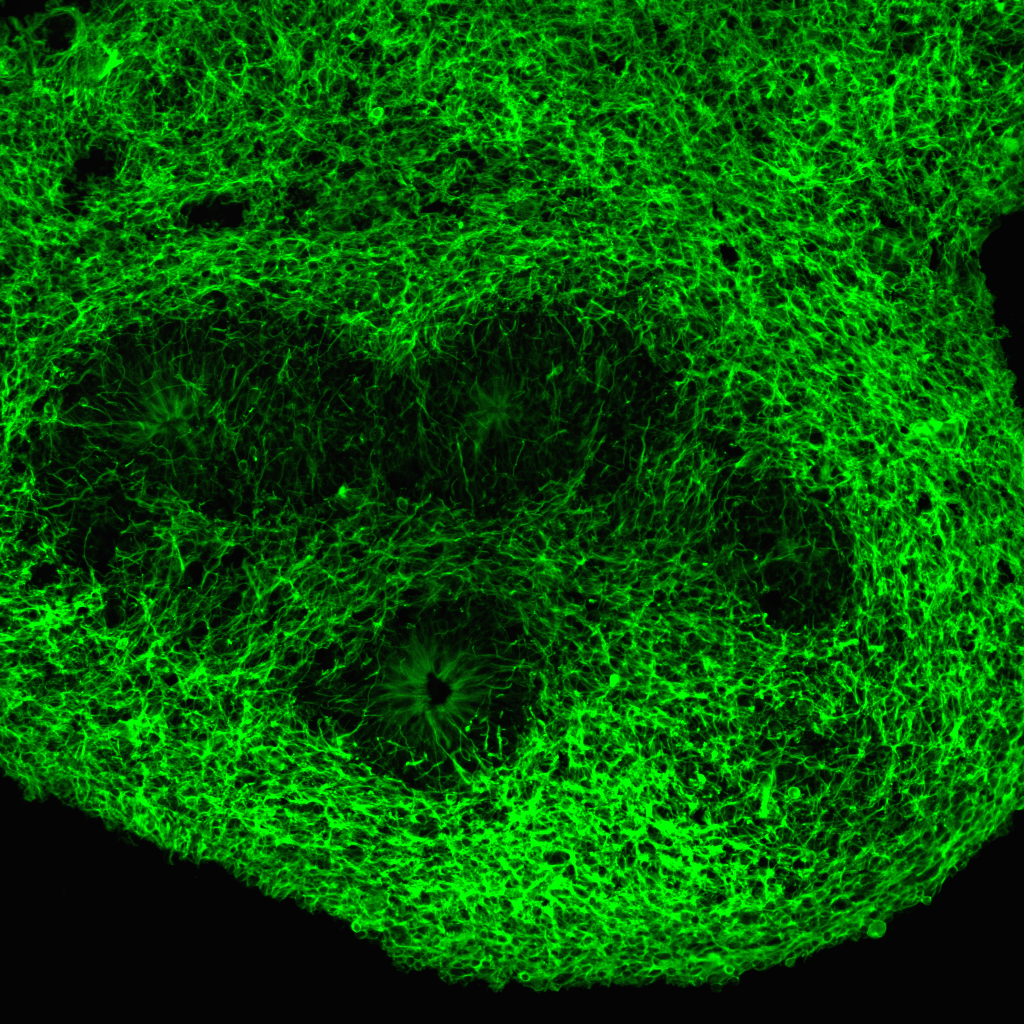

Supplement: Supplementary file 11 — Figure EV3 Source Data [file 44321_2025_302_MOESM11_ESM.zip › Figure EV3/EV3D/#4-1_TUJ1.tif]

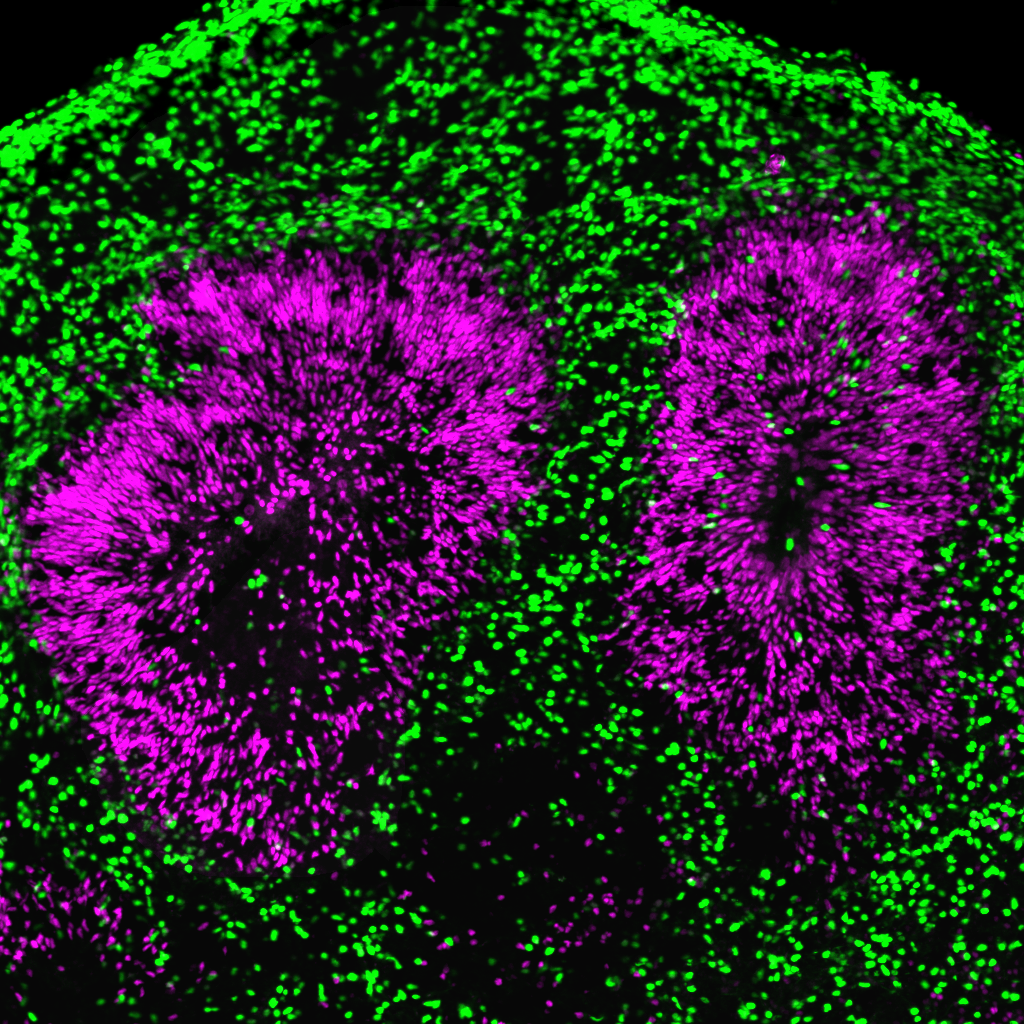

Supplement: Supplementary file 11 — Figure EV3 Source Data [file 44321_2025_302_MOESM11_ESM.zip › Figure EV3/EV3C/#10-6_merge.tif]

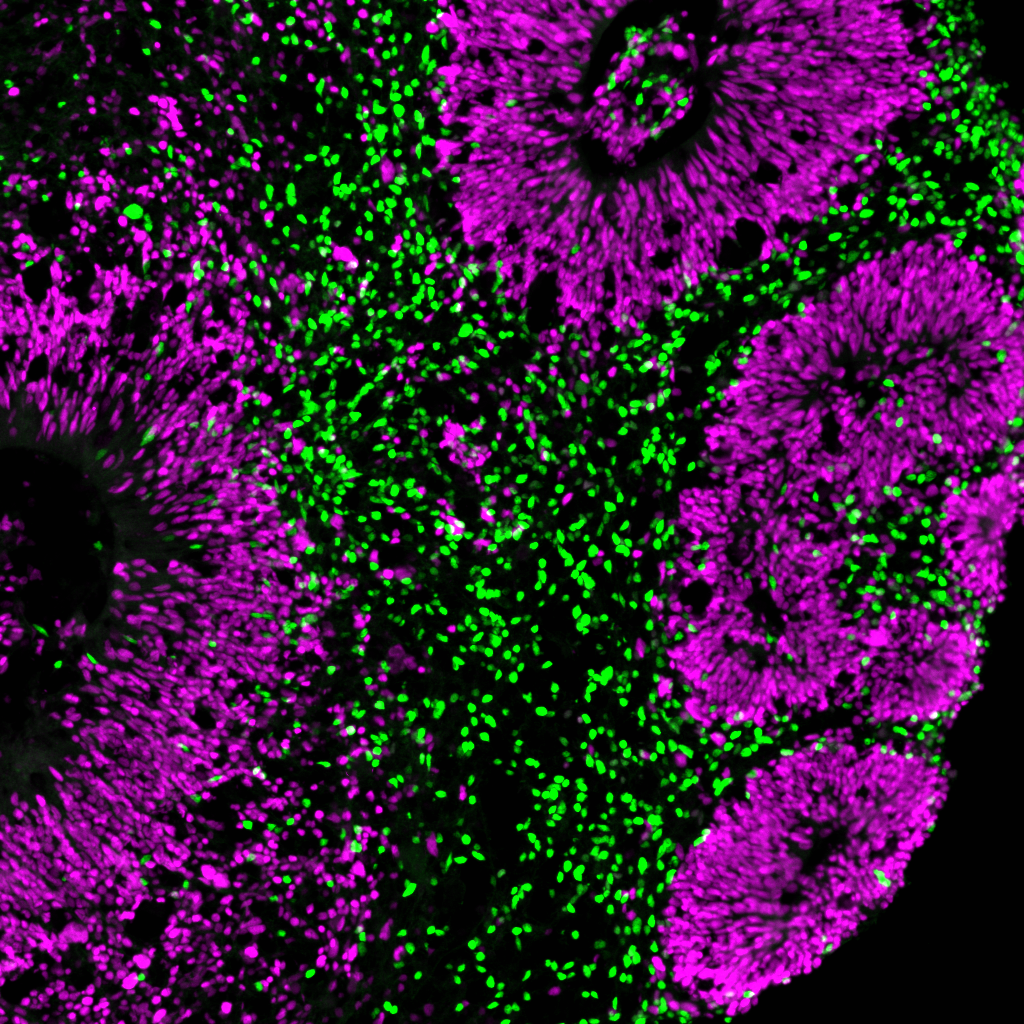

Supplement: Supplementary file 11 — Figure EV3 Source Data [file 44321_2025_302_MOESM11_ESM.zip › Figure EV3/EV3C/#4-1_merge.tif]

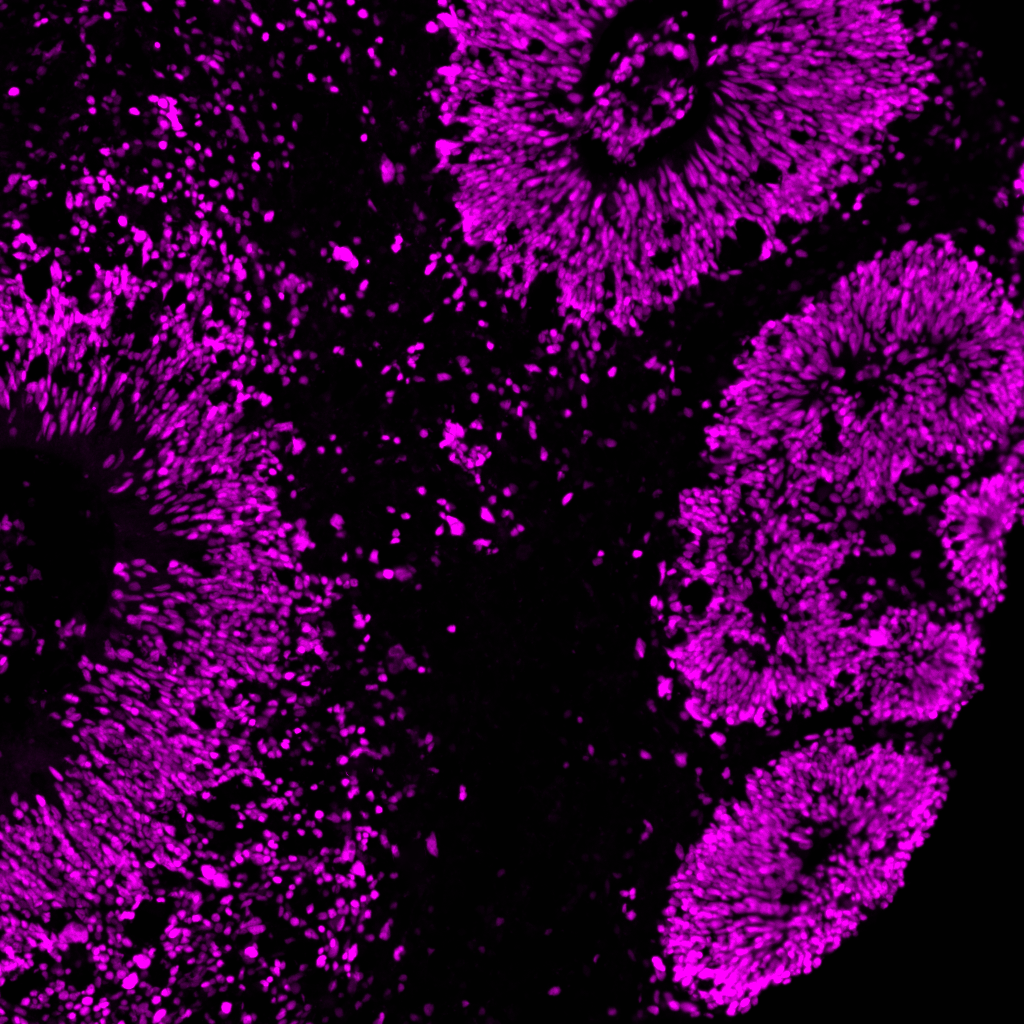

Supplement: Supplementary file 11 — Figure EV3 Source Data [file 44321_2025_302_MOESM11_ESM.zip › Figure EV3/EV3C/#4-1_SOX2.tif]

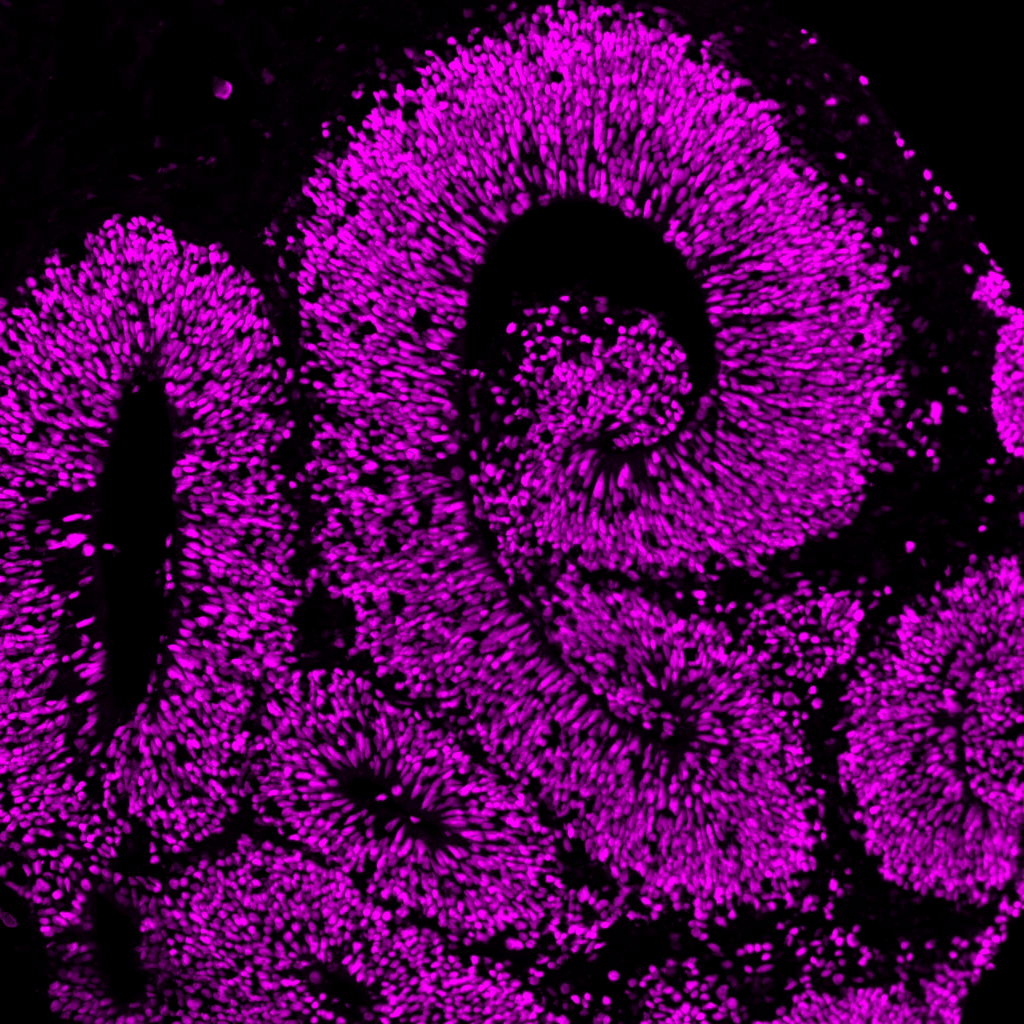

Supplement: Supplementary file 11 — Figure EV3 Source Data [file 44321_2025_302_MOESM11_ESM.zip › Figure EV3/EV3C/WT_SOX2.tif]

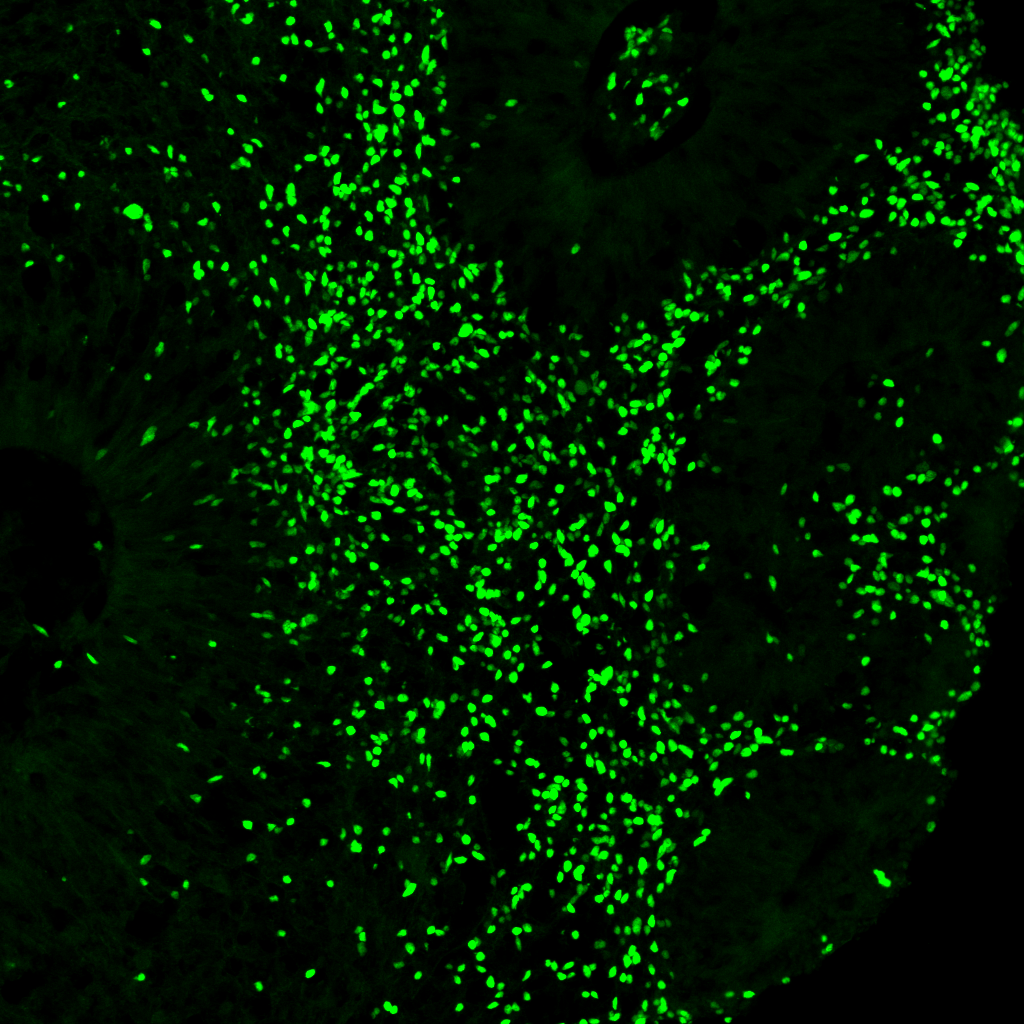

Supplement: Supplementary file 11 — Figure EV3 Source Data [file 44321_2025_302_MOESM11_ESM.zip › Figure EV3/EV3C/#4-1_TBR1.tif]

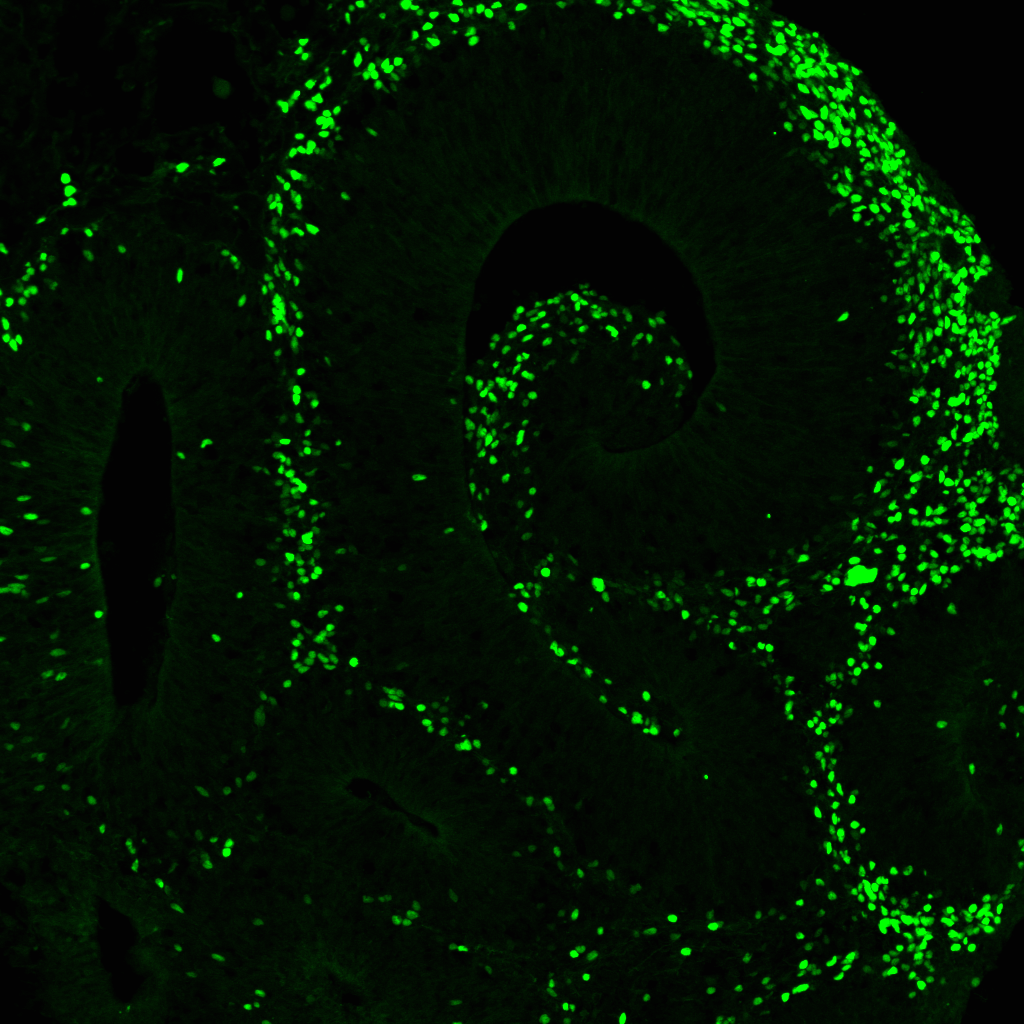

Supplement: Supplementary file 11 — Figure EV3 Source Data [file 44321_2025_302_MOESM11_ESM.zip › Figure EV3/EV3C/WT_TBR1.tif]

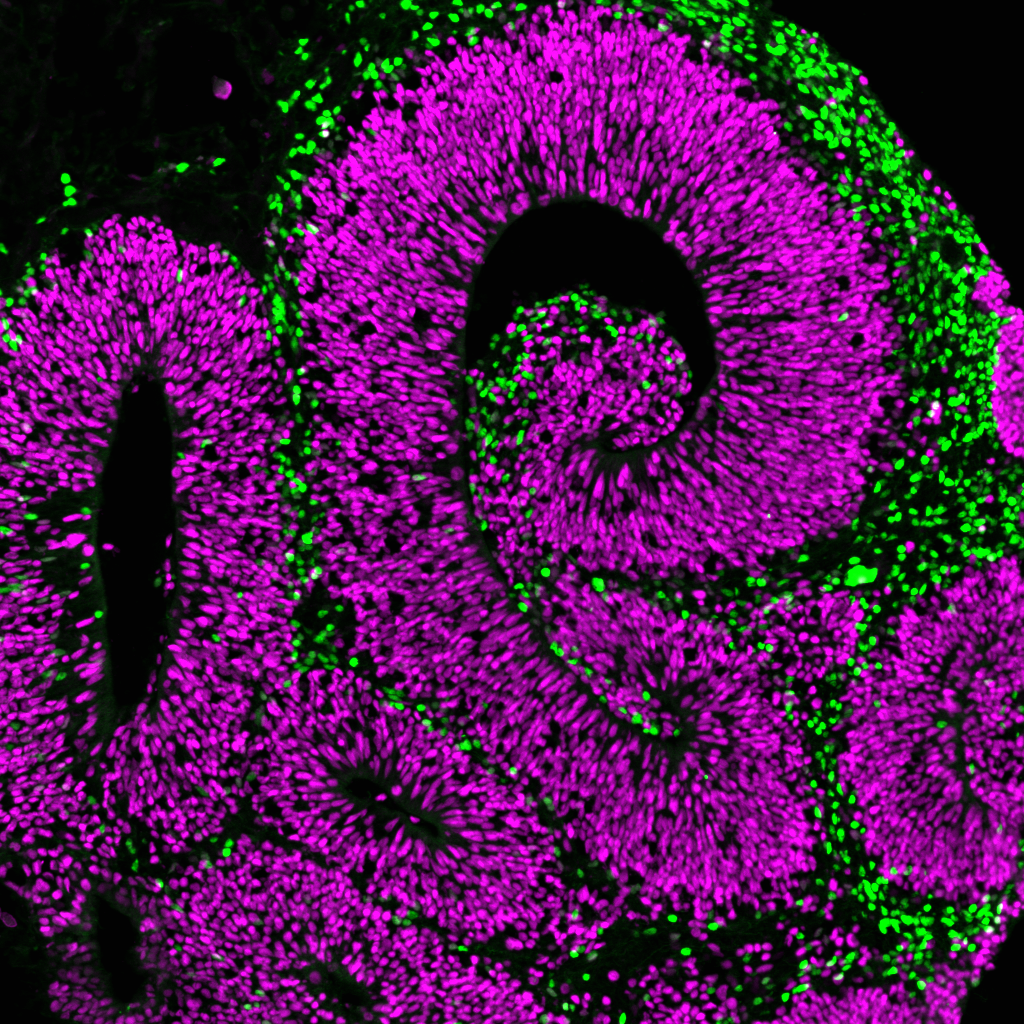

Supplement: Supplementary file 11 — Figure EV3 Source Data [file 44321_2025_302_MOESM11_ESM.zip › Figure EV3/EV3C/WT_merge.tif]

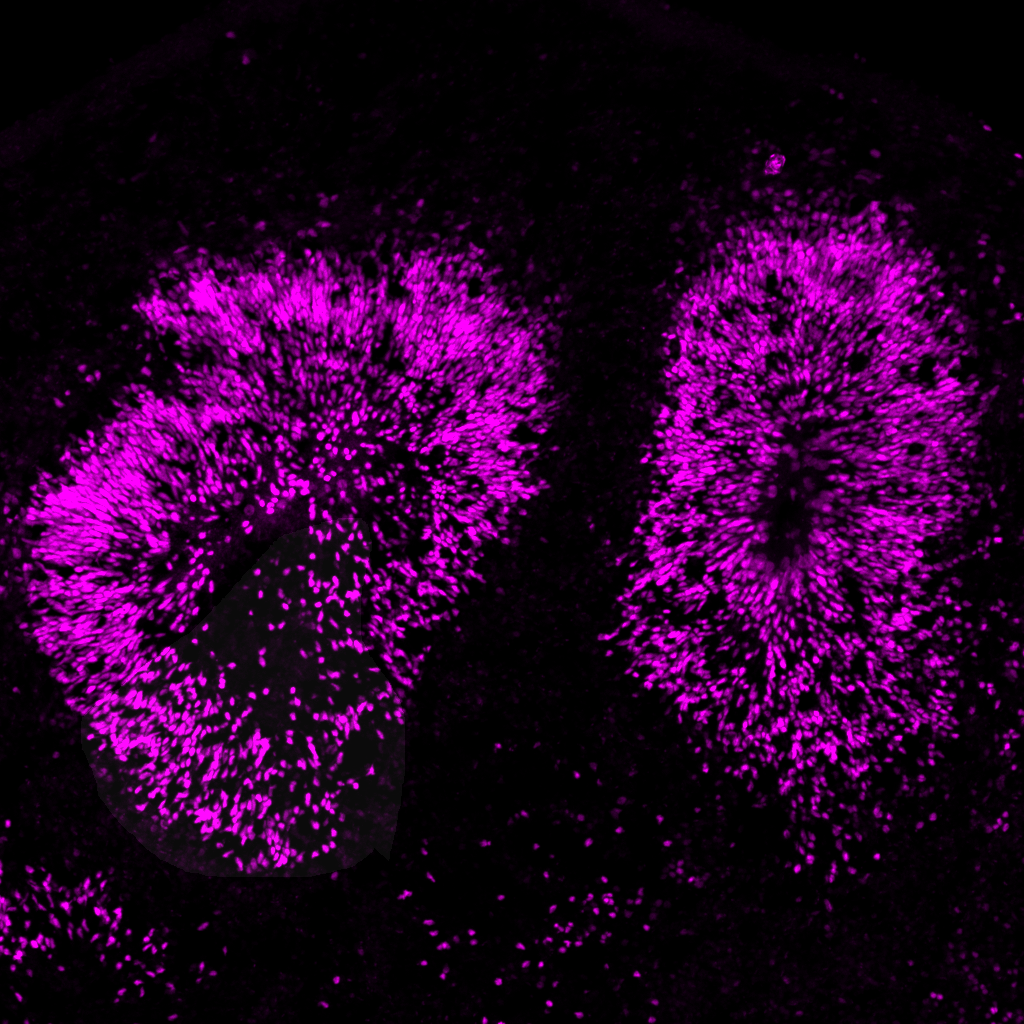

Supplement: Supplementary file 11 — Figure EV3 Source Data [file 44321_2025_302_MOESM11_ESM.zip › Figure EV3/EV3C/#10-6_SOX2.tif]

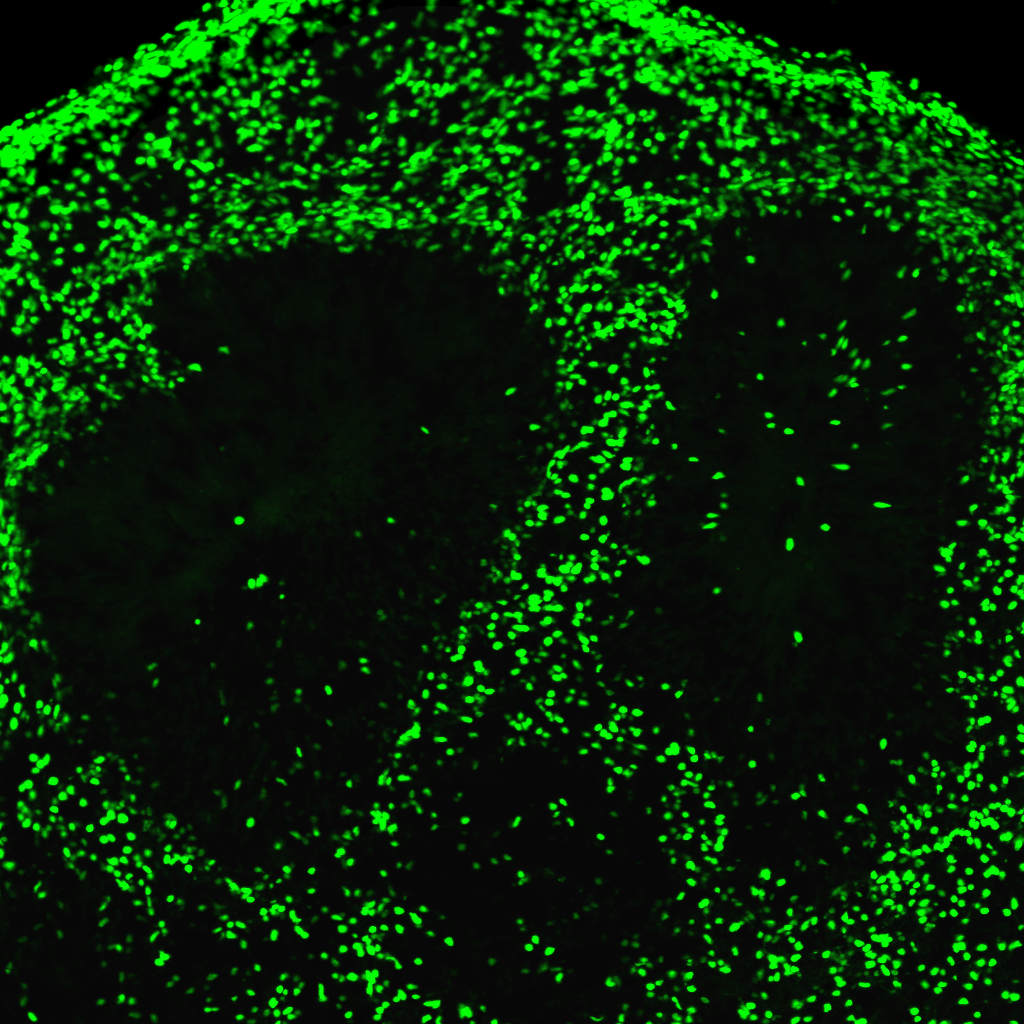

Supplement: Supplementary file 11 — Figure EV3 Source Data [file 44321_2025_302_MOESM11_ESM.zip › Figure EV3/EV3C/#10-6_TBR1.tif]

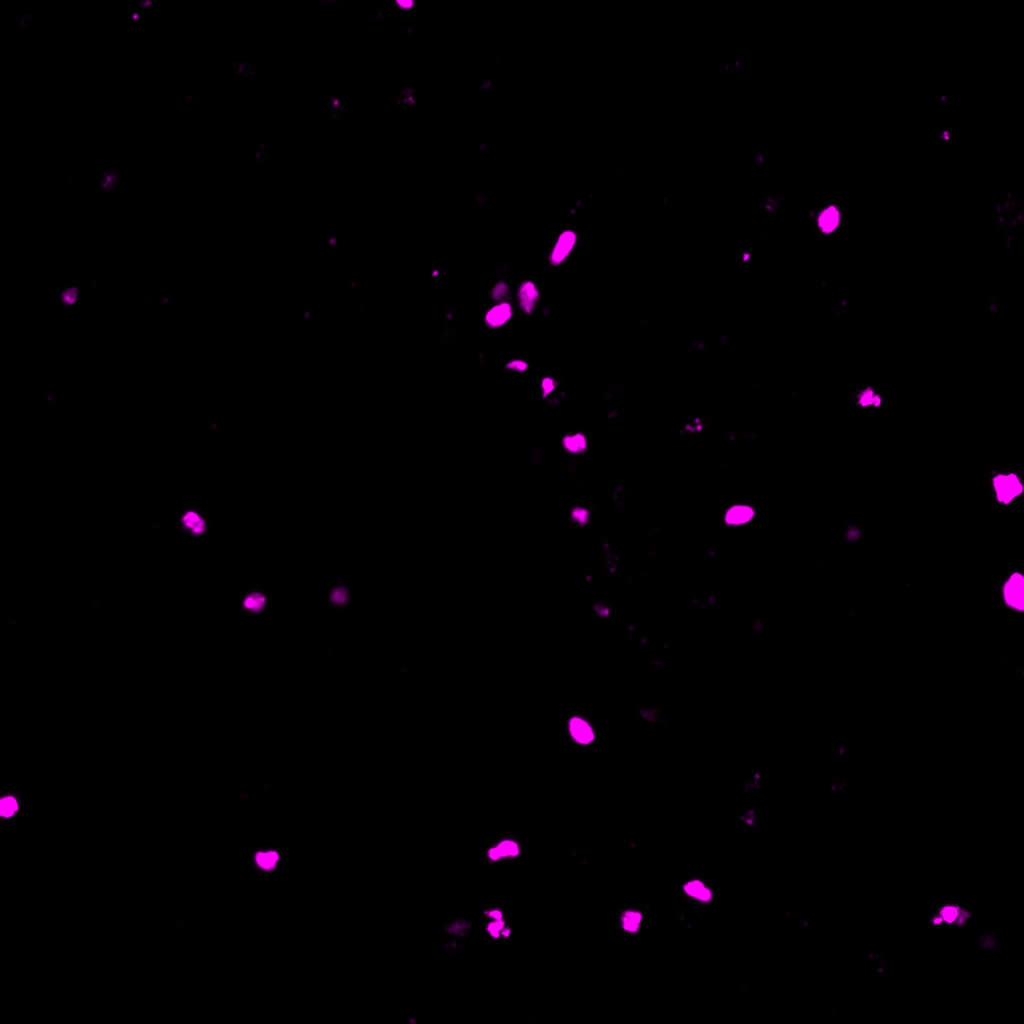

Supplement: Supplementary file 11 — Figure EV3 Source Data [file 44321_2025_302_MOESM11_ESM.zip › Figure EV3/EV3E/#4-1_PH3.tif]

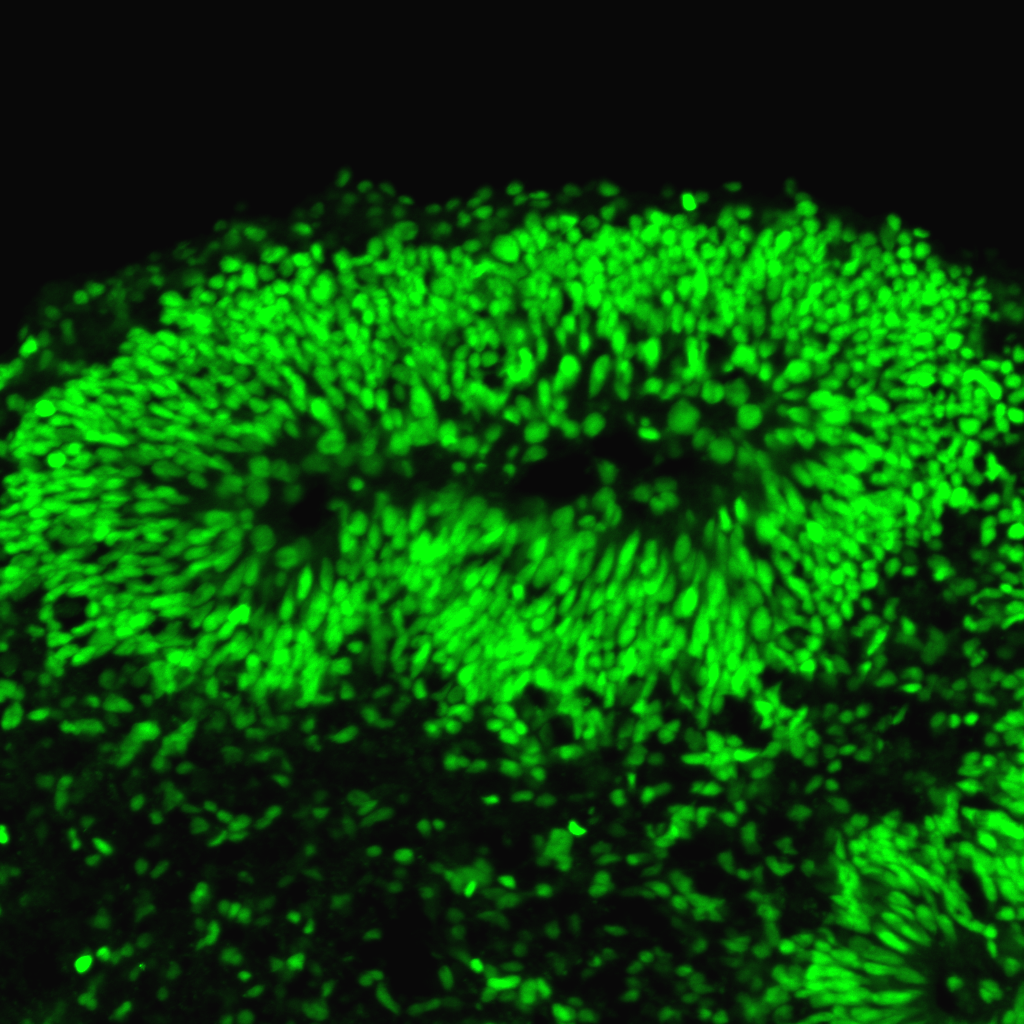

Supplement: Supplementary file 11 — Figure EV3 Source Data [file 44321_2025_302_MOESM11_ESM.zip › Figure EV3/EV3E/WT_PAX6.tif]

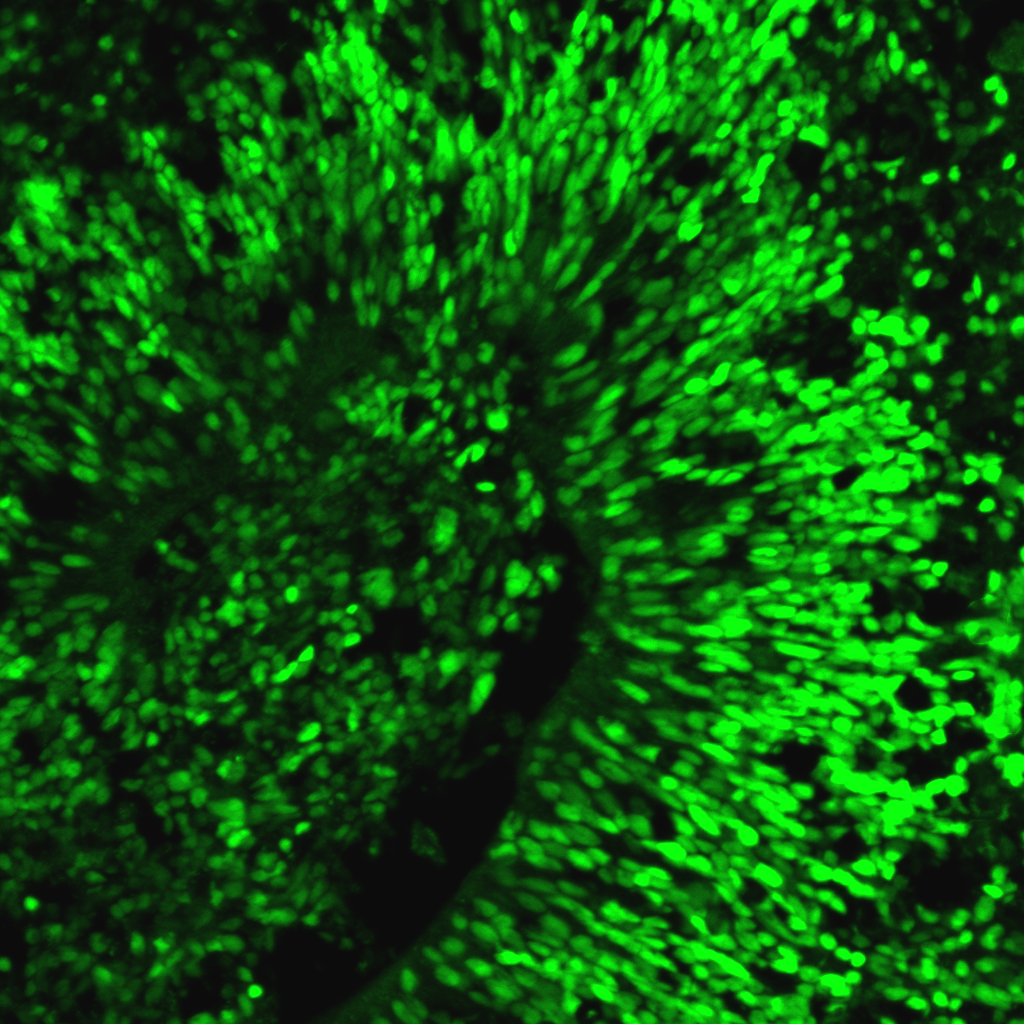

Supplement: Supplementary file 11 — Figure EV3 Source Data [file 44321_2025_302_MOESM11_ESM.zip › Figure EV3/EV3E/#4-1_PAX6.tif]

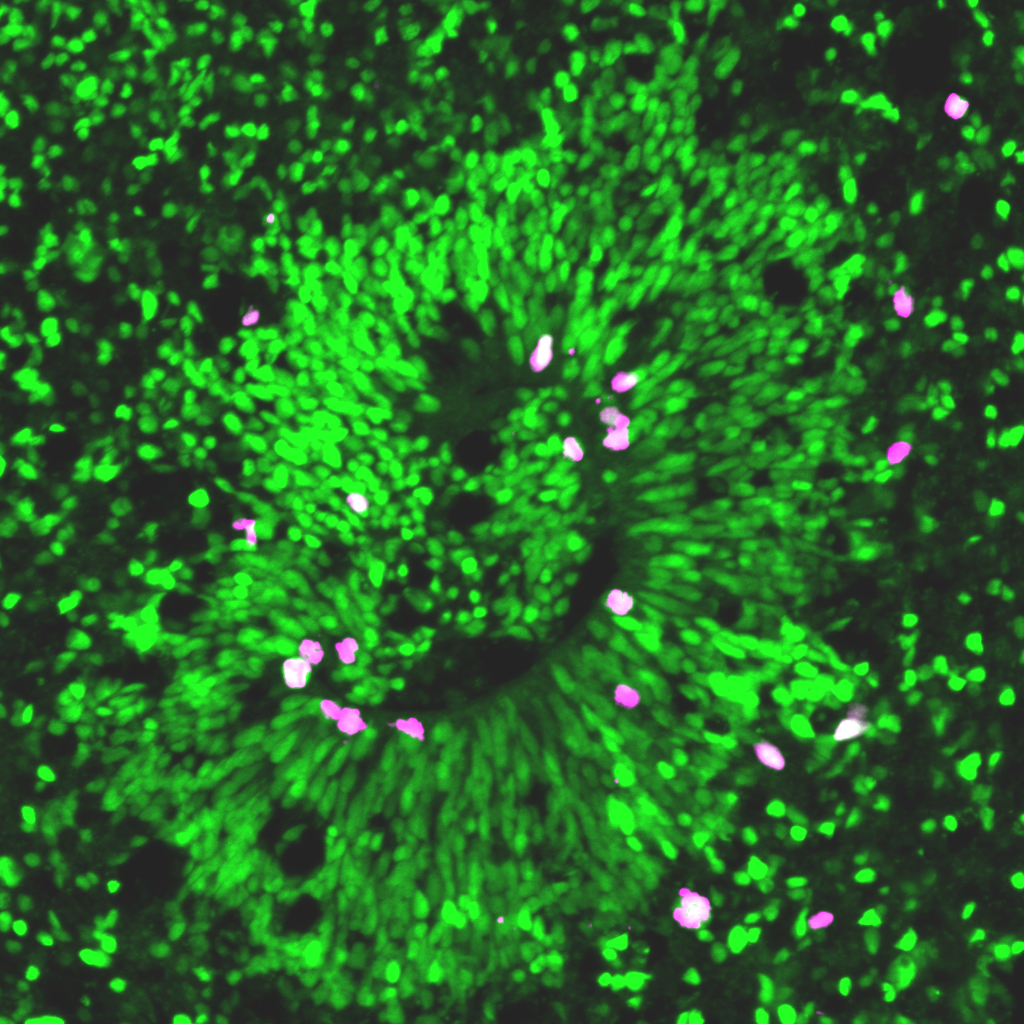

Supplement: Supplementary file 11 — Figure EV3 Source Data [file 44321_2025_302_MOESM11_ESM.zip › Figure EV3/EV3E/#10-6_merge.tif]

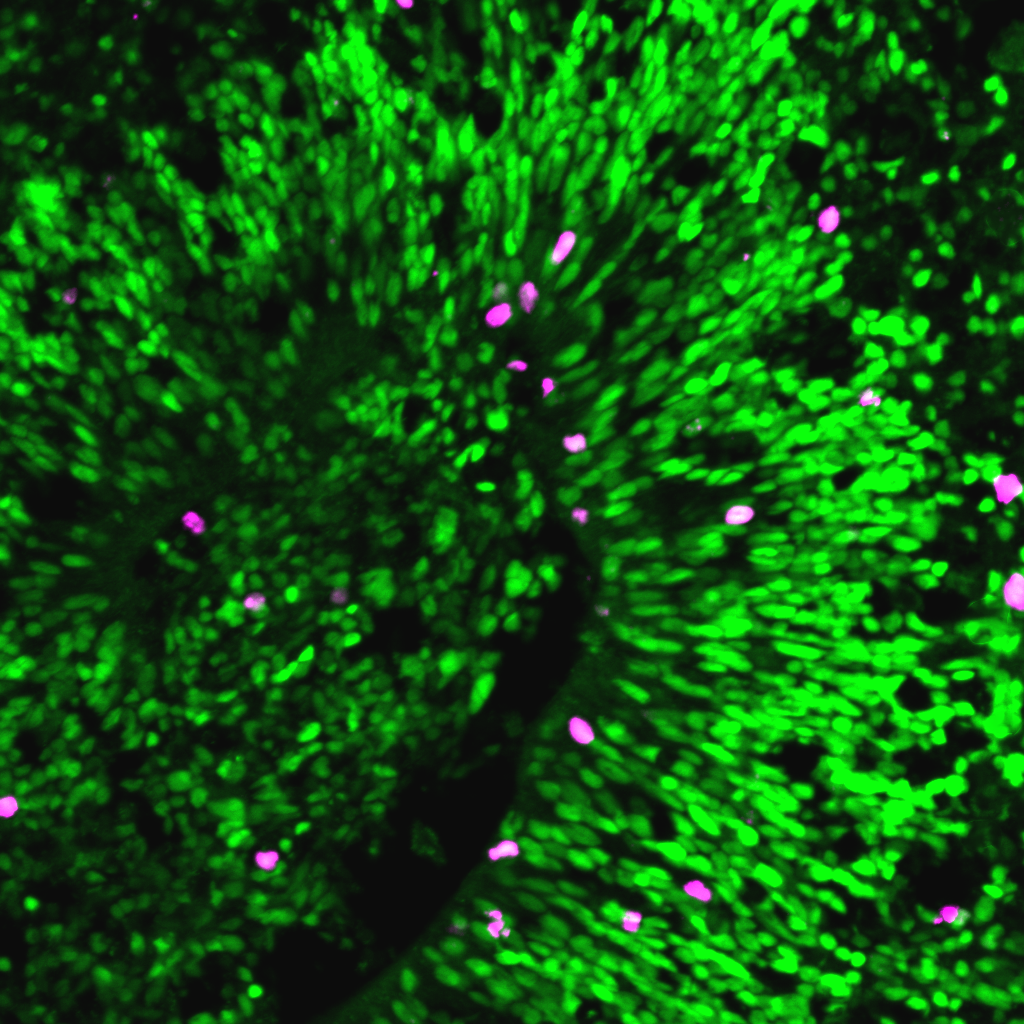

Supplement: Supplementary file 11 — Figure EV3 Source Data [file 44321_2025_302_MOESM11_ESM.zip › Figure EV3/EV3E/#4-1_merge.tif]

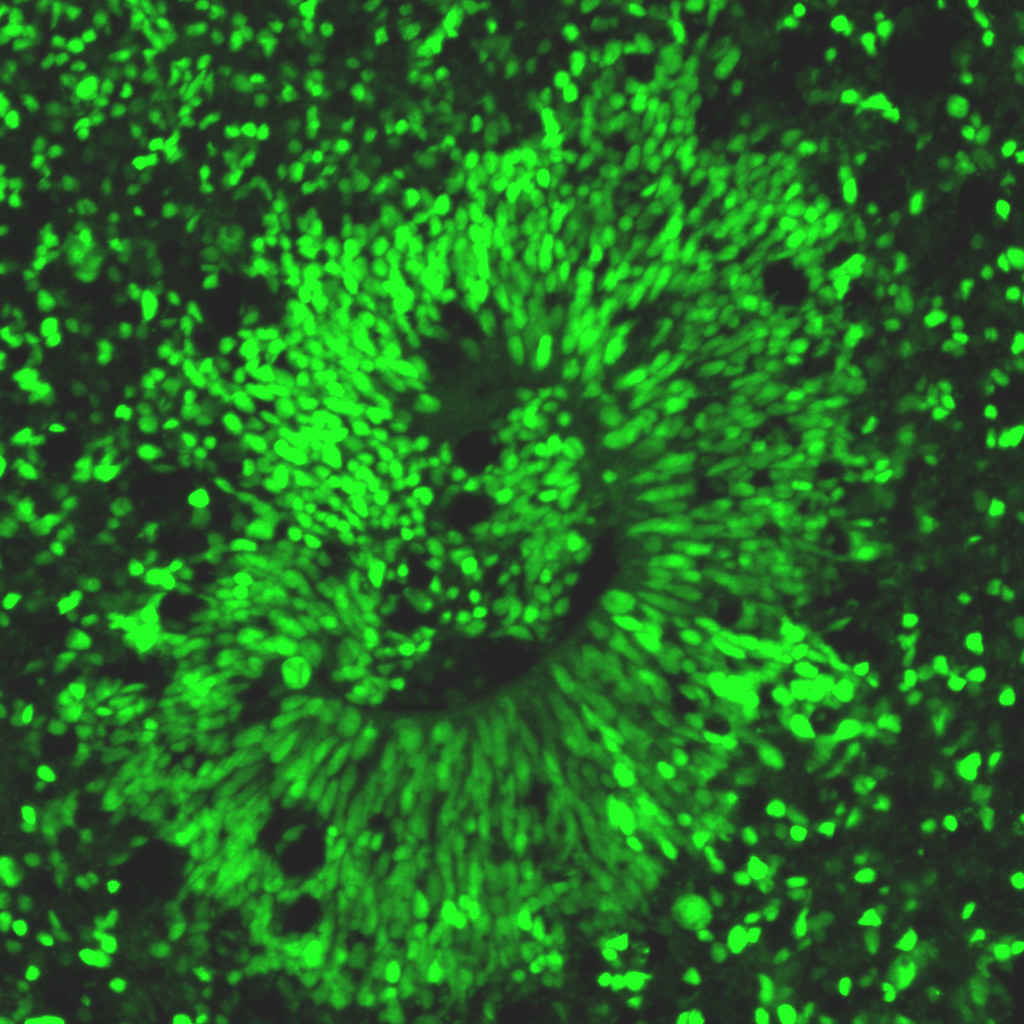

Supplement: Supplementary file 11 — Figure EV3 Source Data [file 44321_2025_302_MOESM11_ESM.zip › Figure EV3/EV3E/#10-6_PAX6.tif]

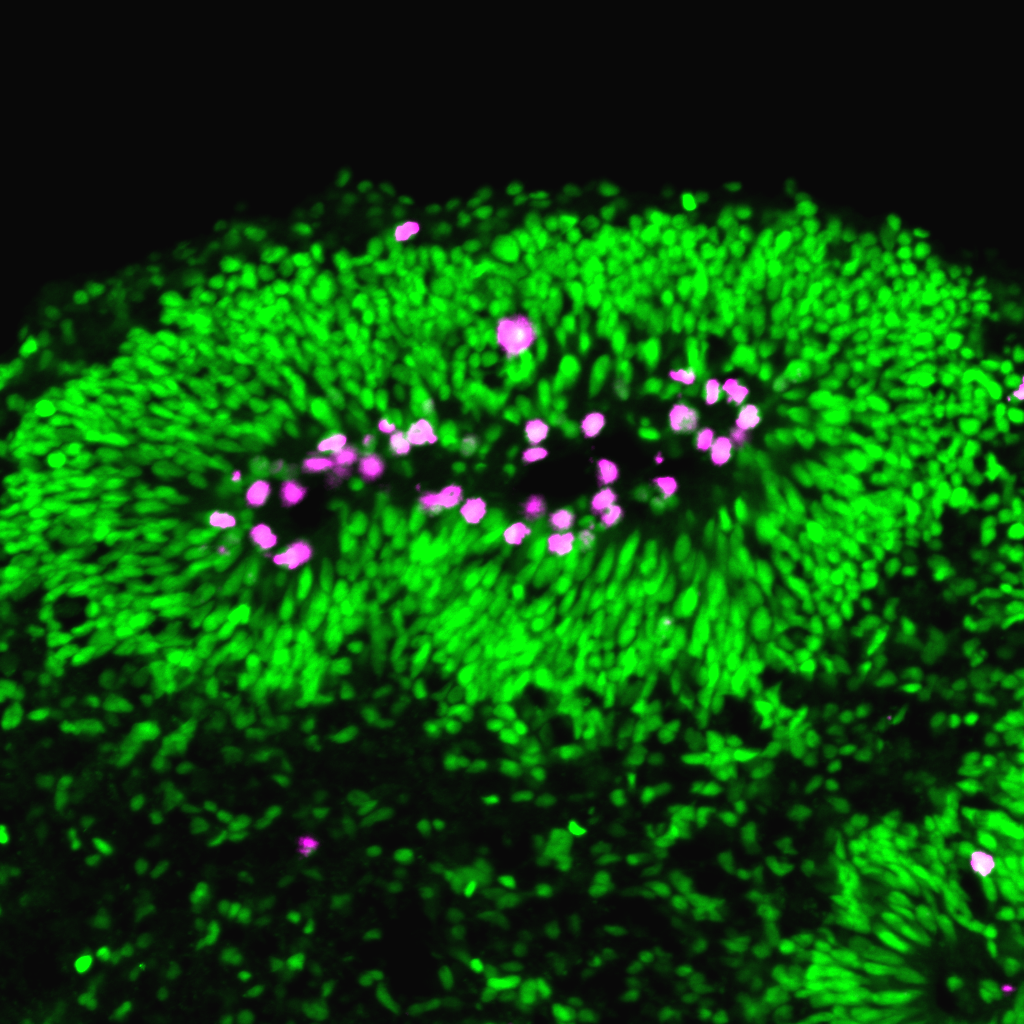

Supplement: Supplementary file 11 — Figure EV3 Source Data [file 44321_2025_302_MOESM11_ESM.zip › Figure EV3/EV3E/WT_merge.tif]

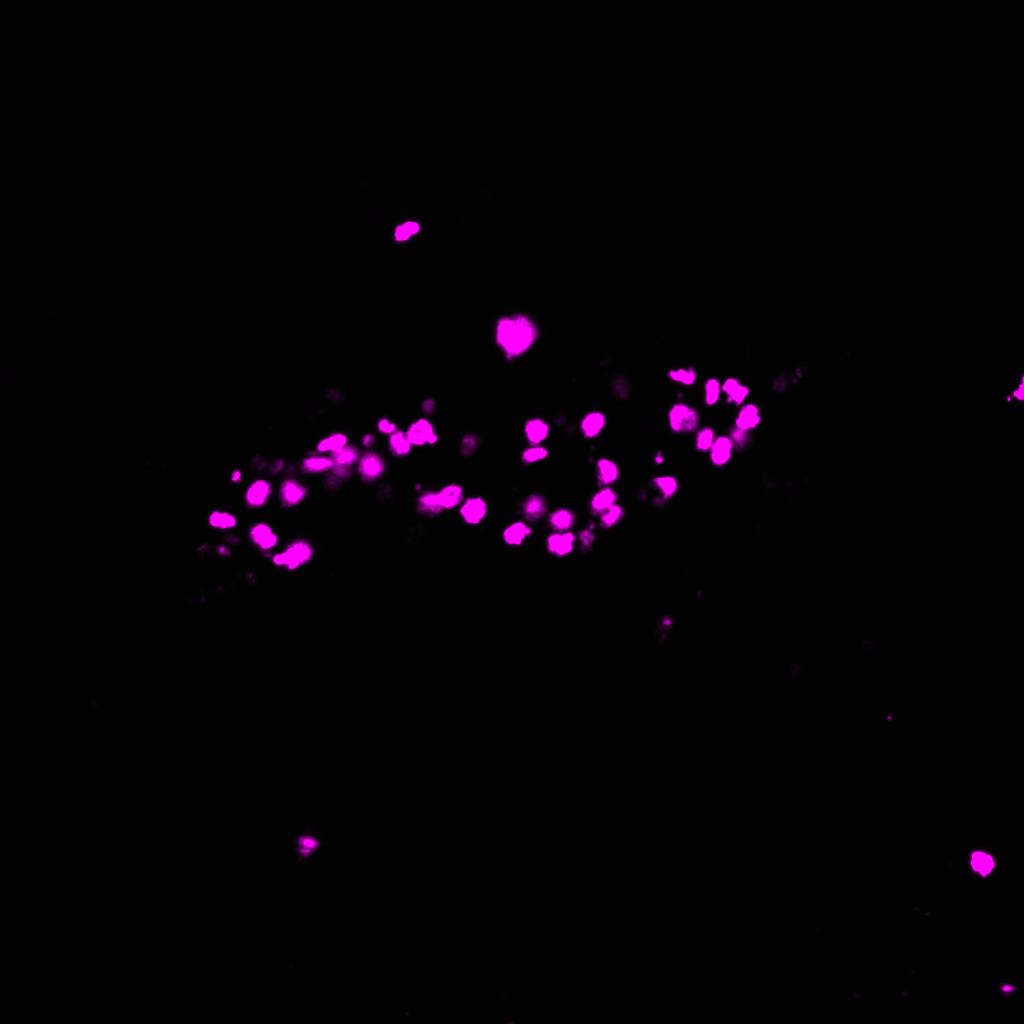

Supplement: Supplementary file 11 — Figure EV3 Source Data [file 44321_2025_302_MOESM11_ESM.zip › Figure EV3/EV3E/WT_PH3.tif]

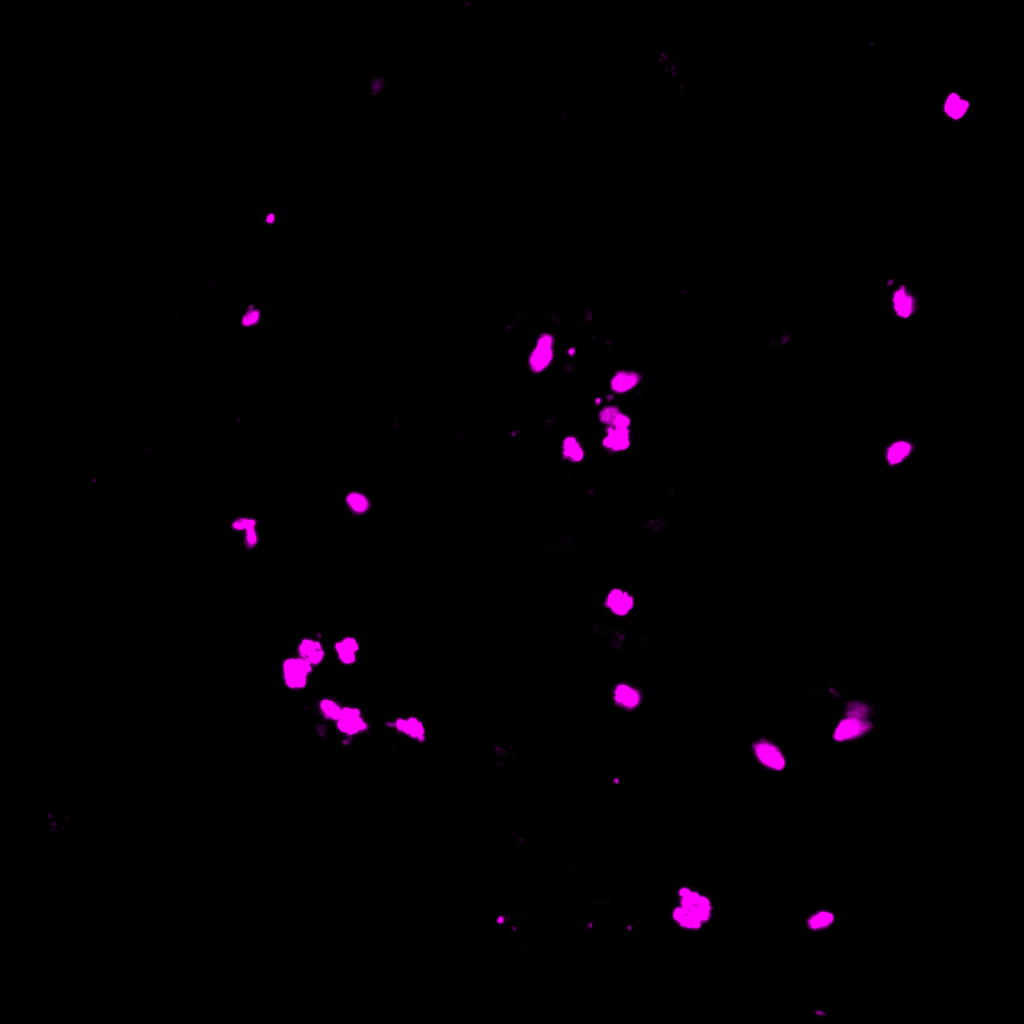

Supplement: Supplementary file 11 — Figure EV3 Source Data [file 44321_2025_302_MOESM11_ESM.zip › Figure EV3/EV3E/#10-6_PH3.tif]

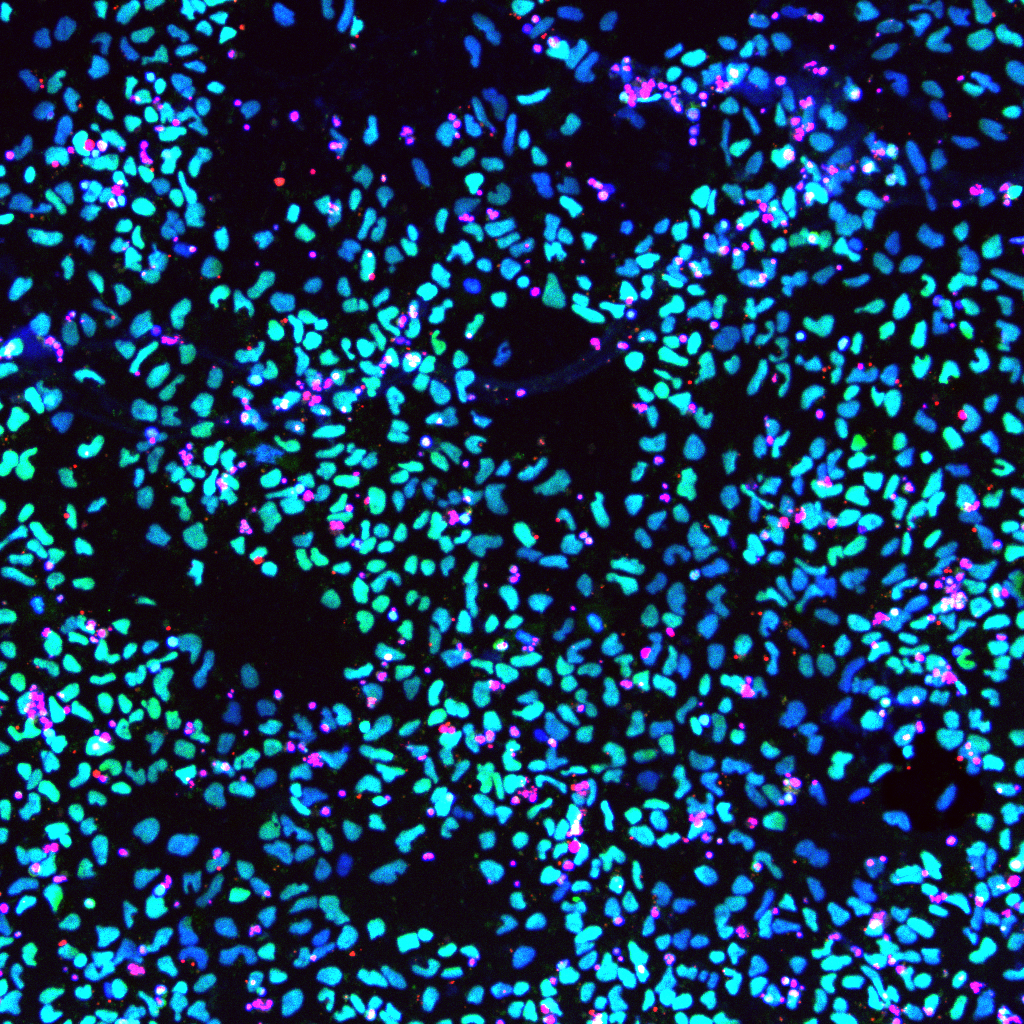

Supplement: Supplementary file 12 — Figure EV4 Source Data [file 44321_2025_302_MOESM12_ESM.zip › Figure EV4/EV4F/#12-3-merge.tif]

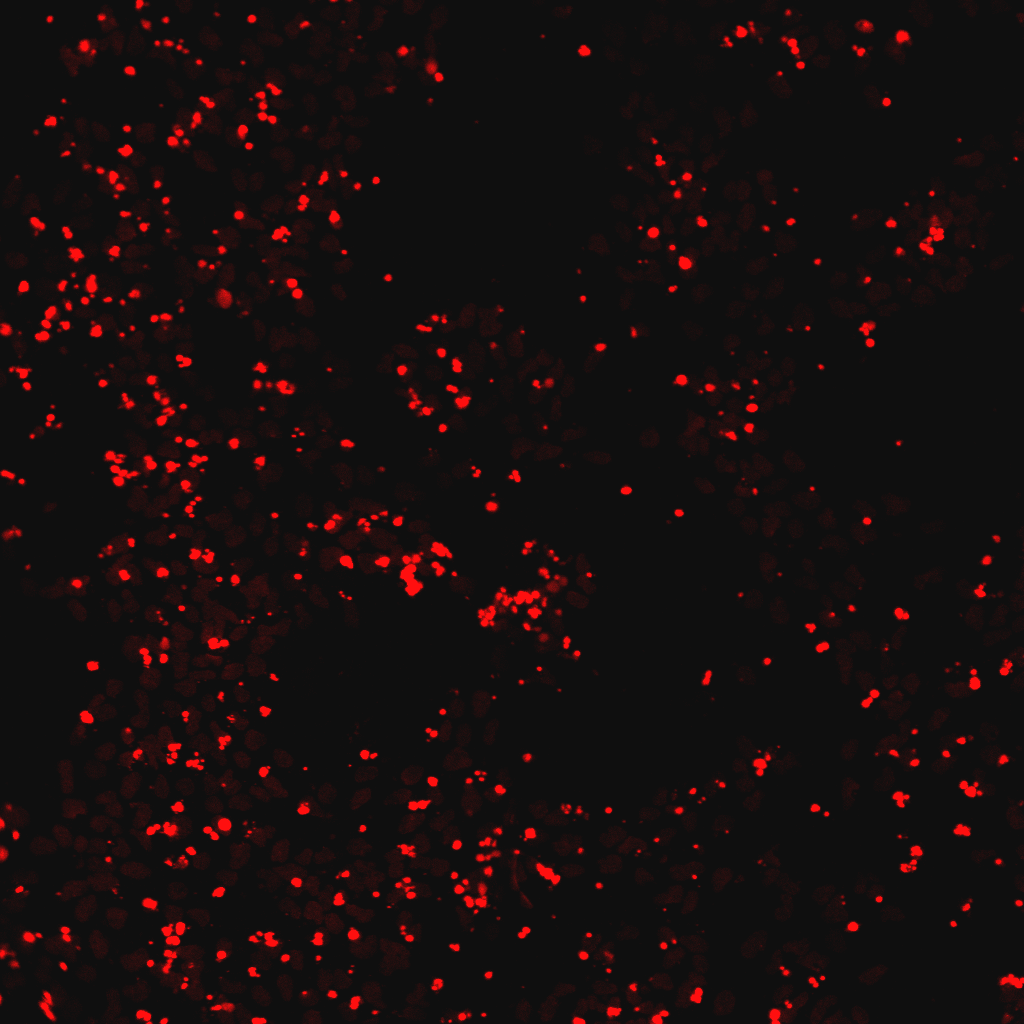

Supplement: Supplementary file 12 — Figure EV4 Source Data [file 44321_2025_302_MOESM12_ESM.zip › Figure EV4/EV4F/#7-5-TUNEL.tif]

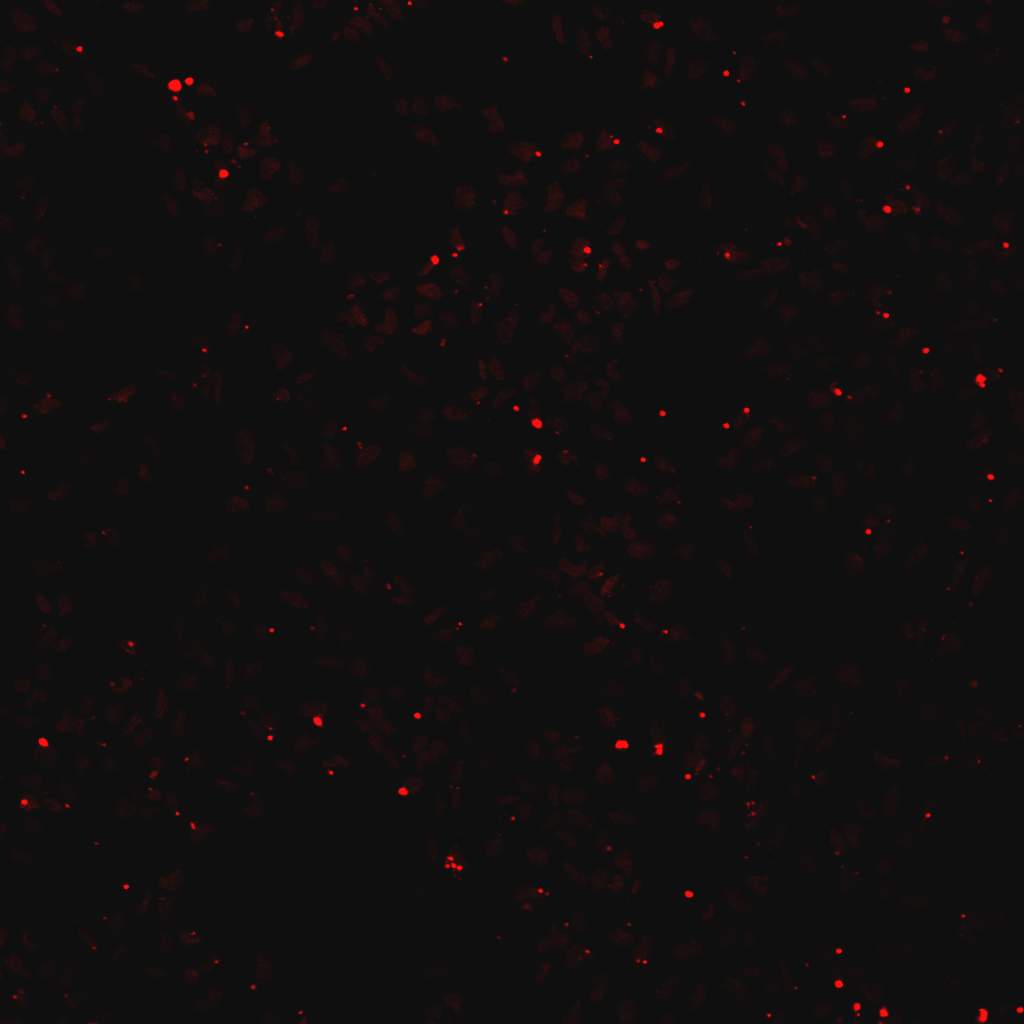

Supplement: Supplementary file 12 — Figure EV4 Source Data [file 44321_2025_302_MOESM12_ESM.zip › Figure EV4/EV4F/H9-TUNEL.tif]

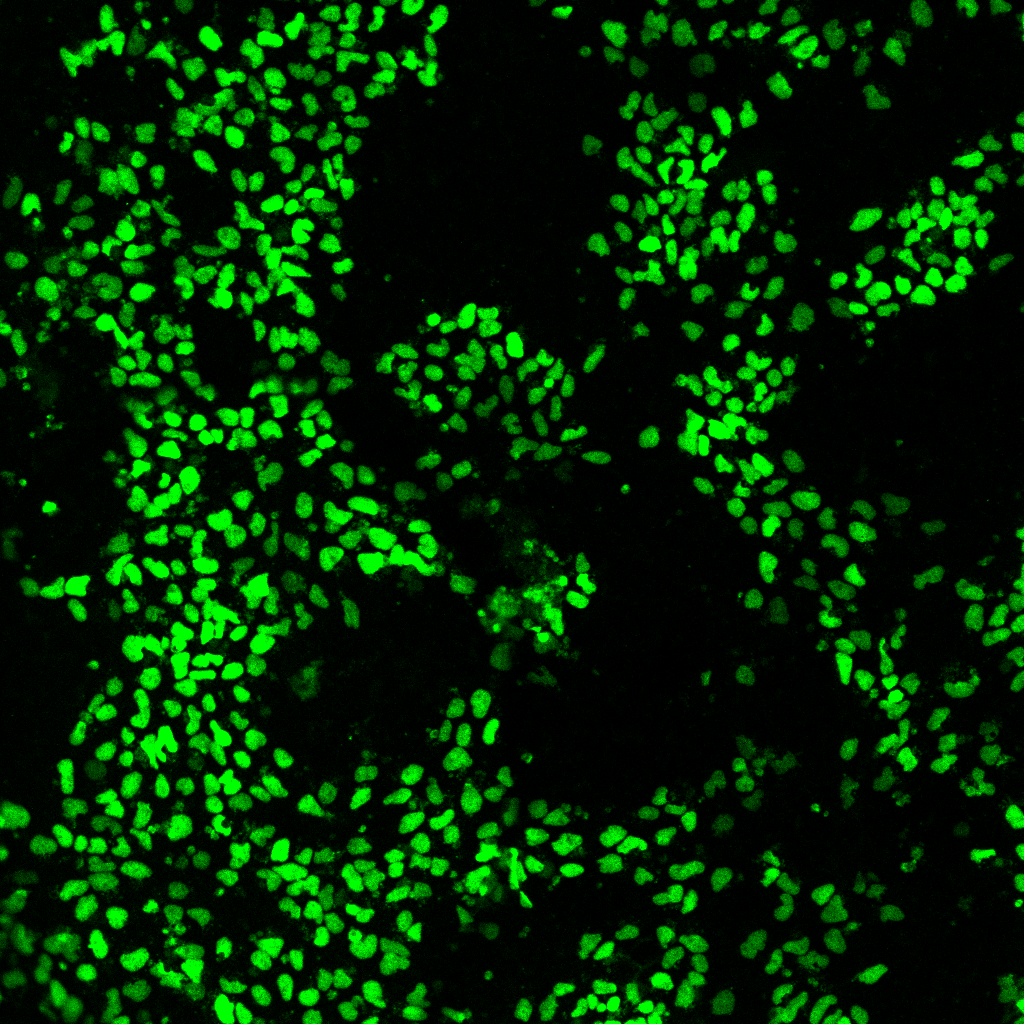

Supplement: Supplementary file 12 — Figure EV4 Source Data [file 44321_2025_302_MOESM12_ESM.zip › Figure EV4/EV4F/#7-5-PAX6.tif]

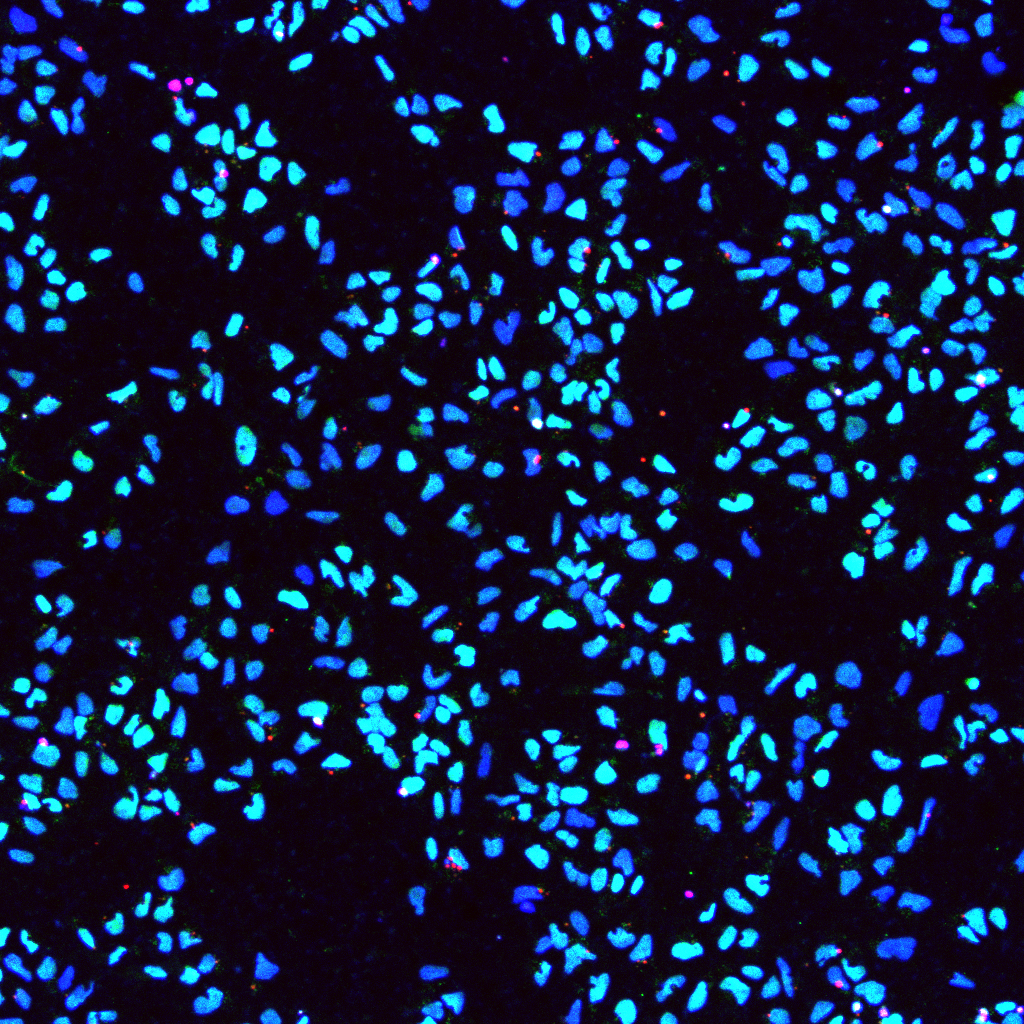

Supplement: Supplementary file 12 — Figure EV4 Source Data [file 44321_2025_302_MOESM12_ESM.zip › Figure EV4/EV4F/H9-merge.tif]

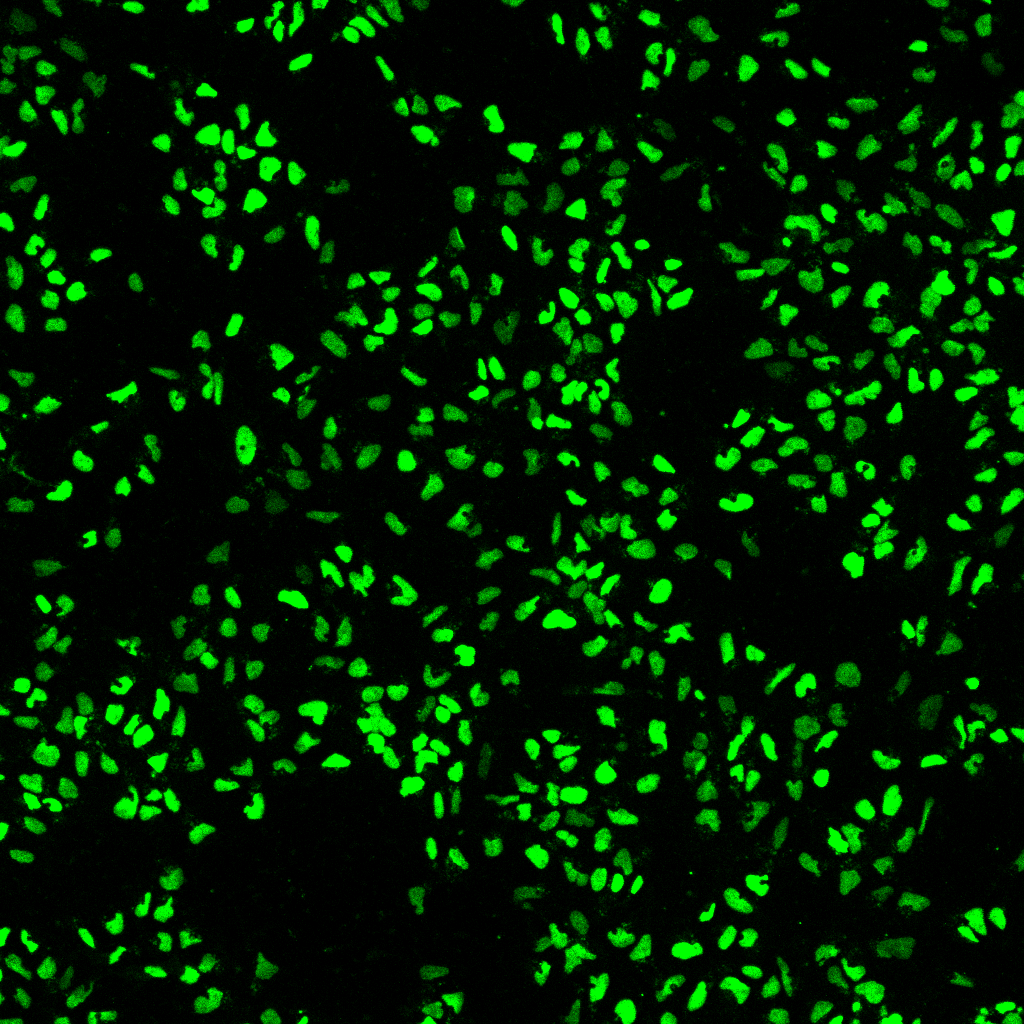

Supplement: Supplementary file 12 — Figure EV4 Source Data [file 44321_2025_302_MOESM12_ESM.zip › Figure EV4/EV4F/H9-PAX6.tif]

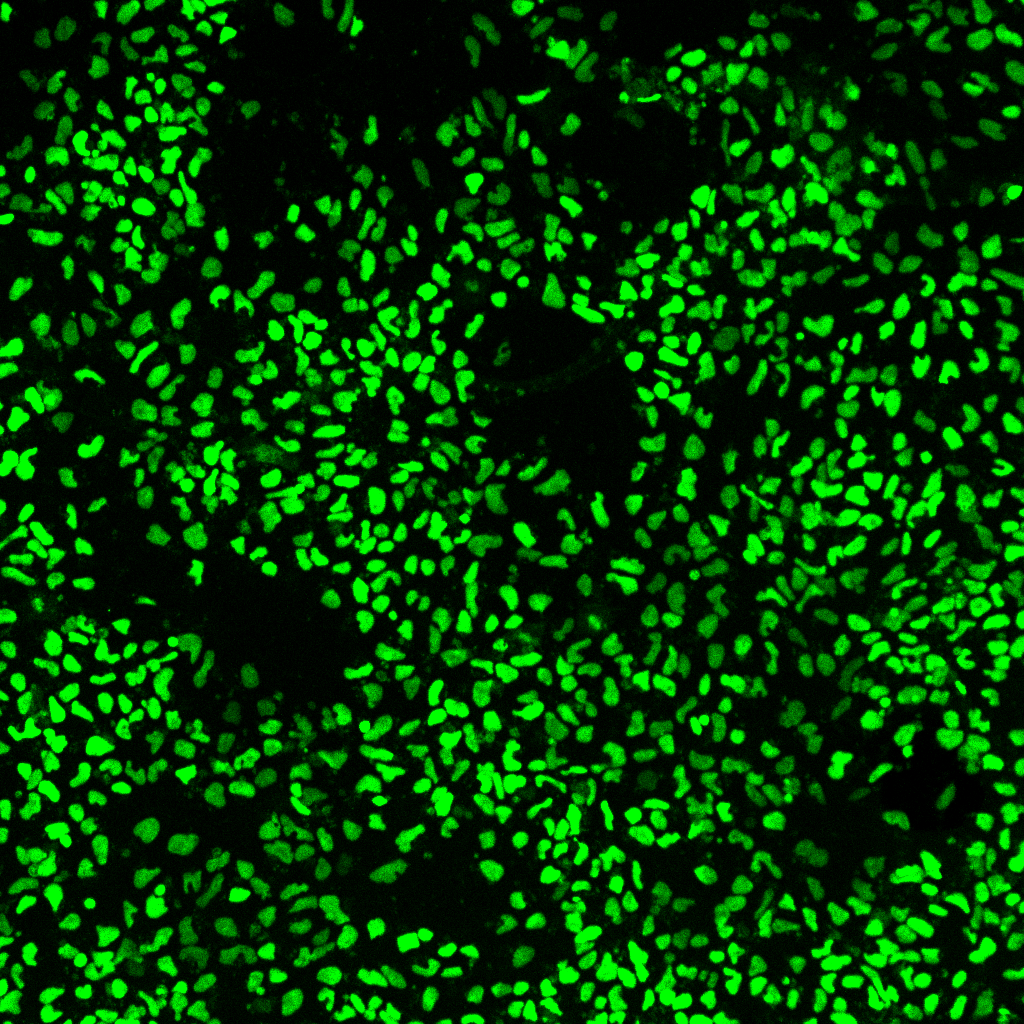

Supplement: Supplementary file 12 — Figure EV4 Source Data [file 44321_2025_302_MOESM12_ESM.zip › Figure EV4/EV4F/#12-3-PAX6.tif]

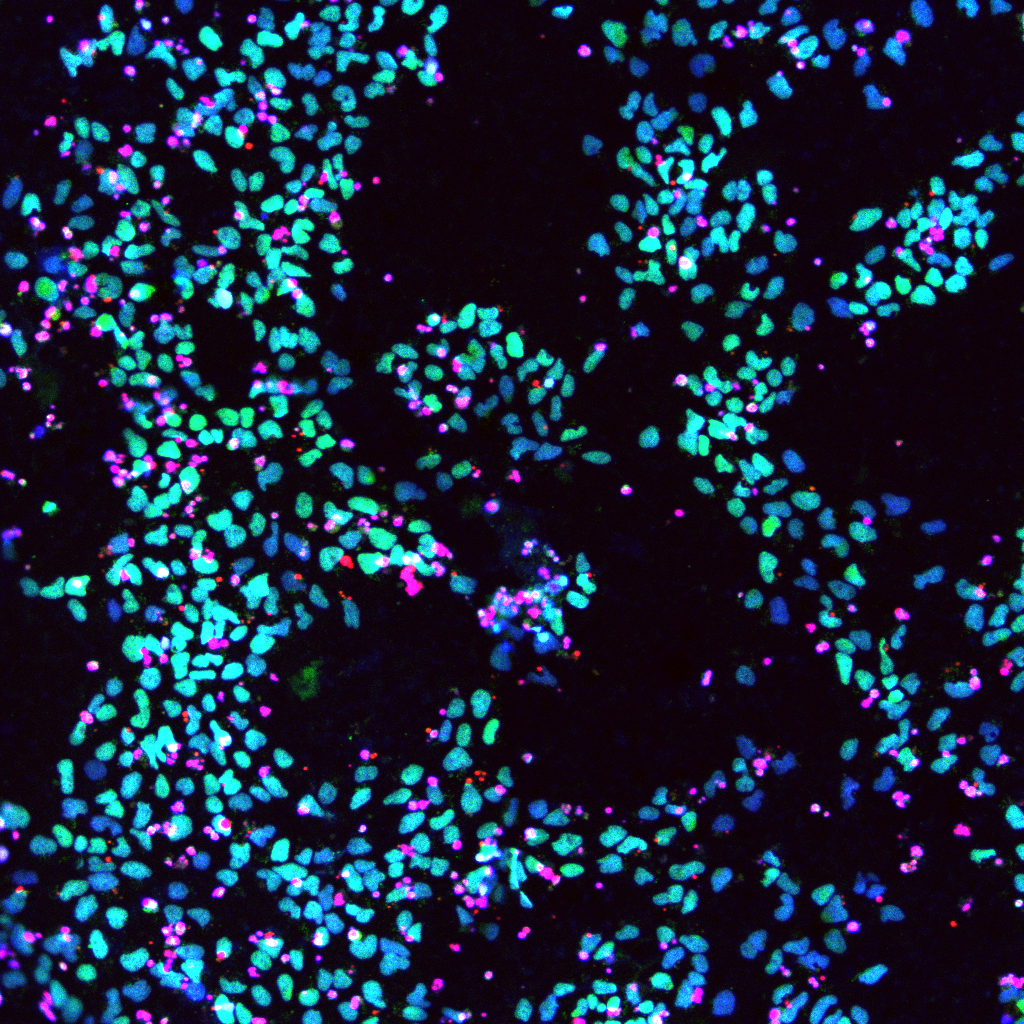

Supplement: Supplementary file 12 — Figure EV4 Source Data [file 44321_2025_302_MOESM12_ESM.zip › Figure EV4/EV4F/#7-5-merge.tif]

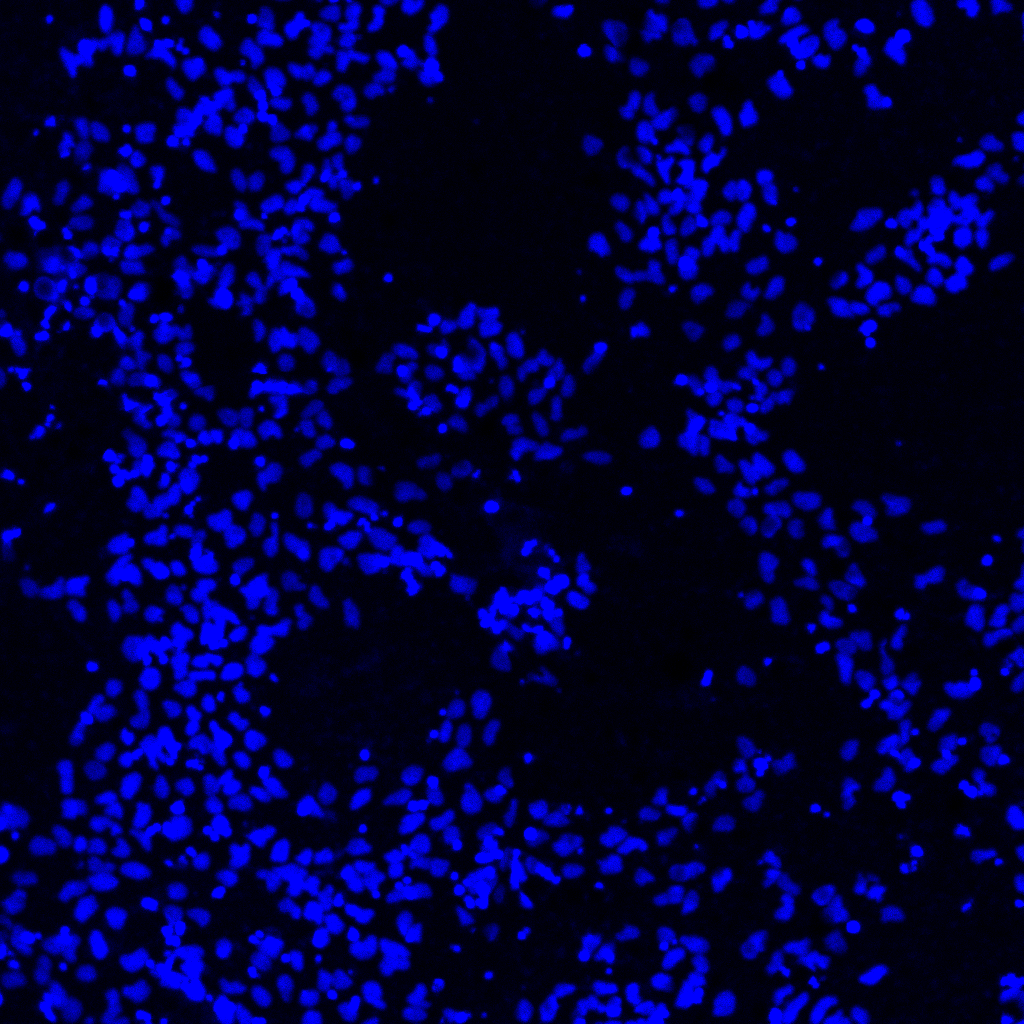

Supplement: Supplementary file 12 — Figure EV4 Source Data [file 44321_2025_302_MOESM12_ESM.zip › Figure EV4/EV4F/#7-5-DAPI.tif]

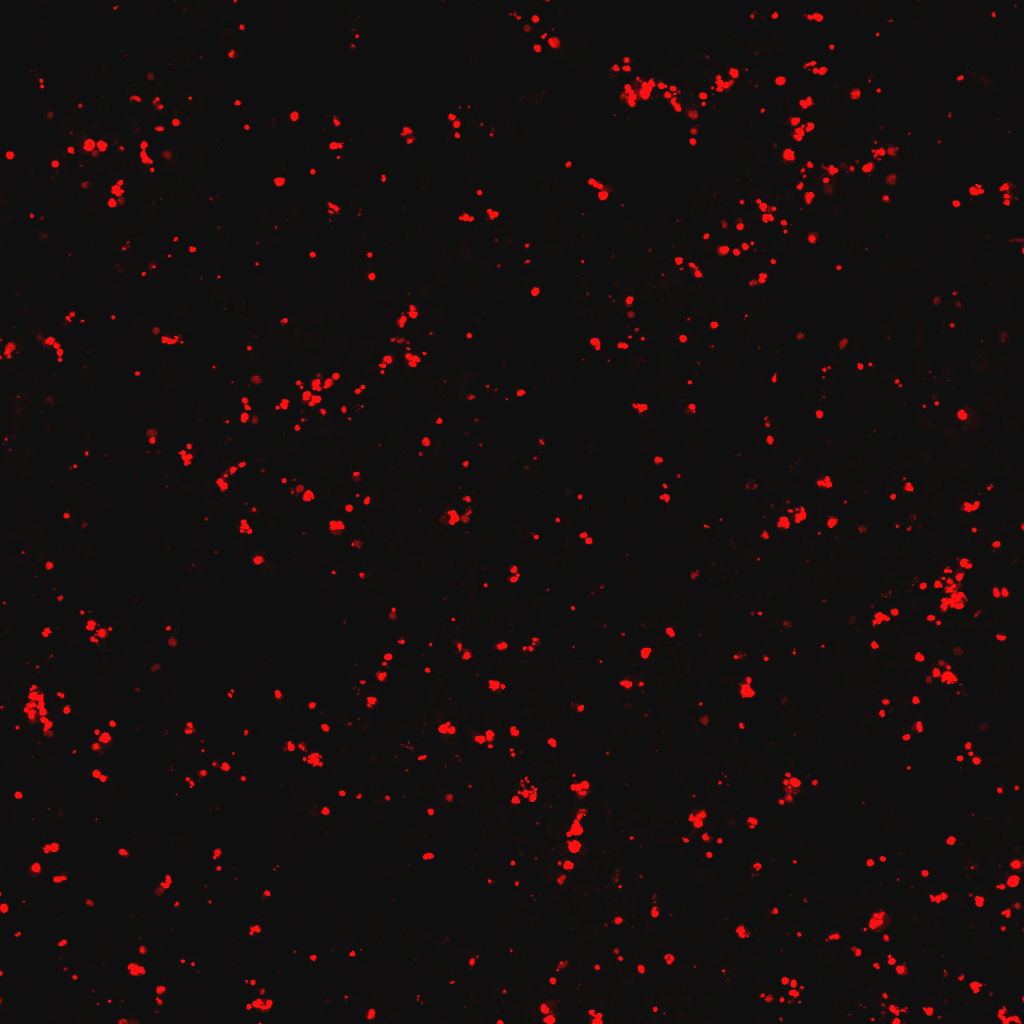

Supplement: Supplementary file 12 — Figure EV4 Source Data [file 44321_2025_302_MOESM12_ESM.zip › Figure EV4/EV4F/#12-3-TUNEL.tif]

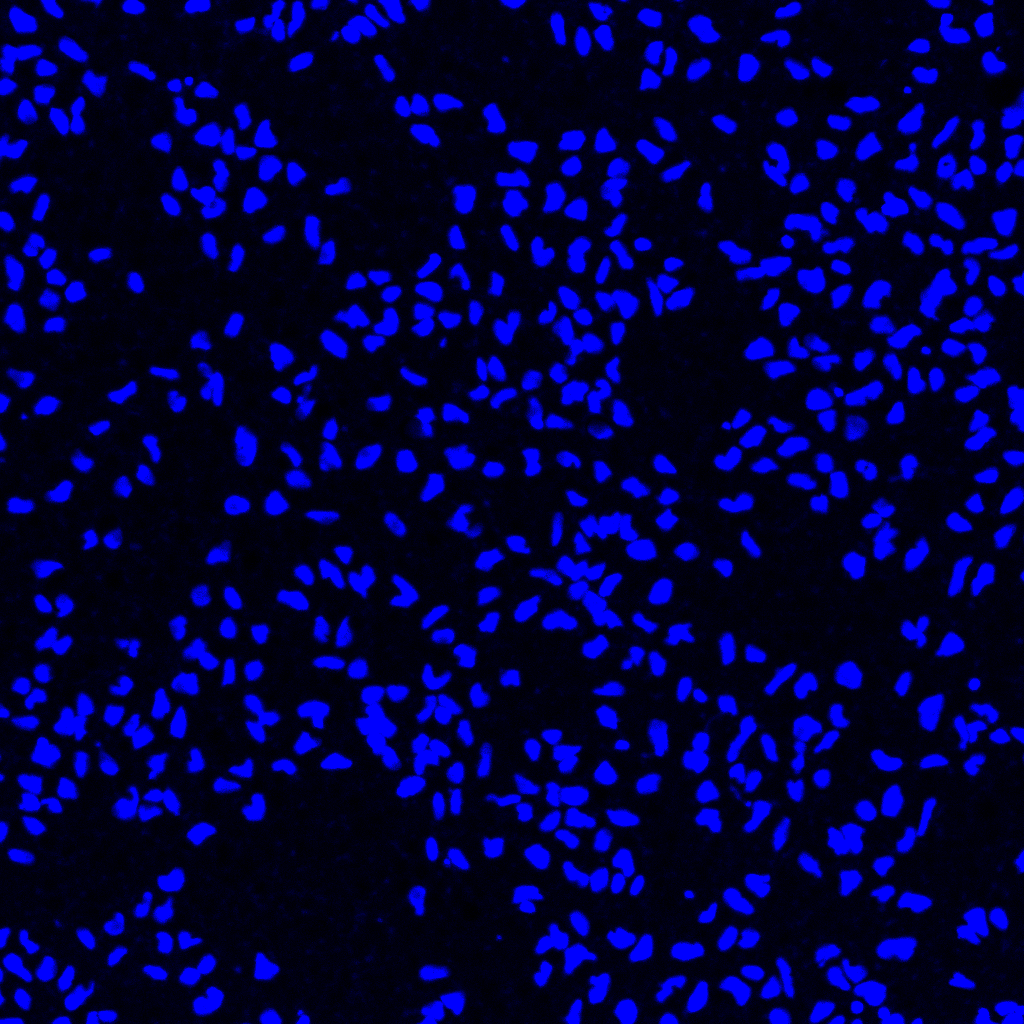

Supplement: Supplementary file 12 — Figure EV4 Source Data [file 44321_2025_302_MOESM12_ESM.zip › Figure EV4/EV4F/H9-DAPI.tif]

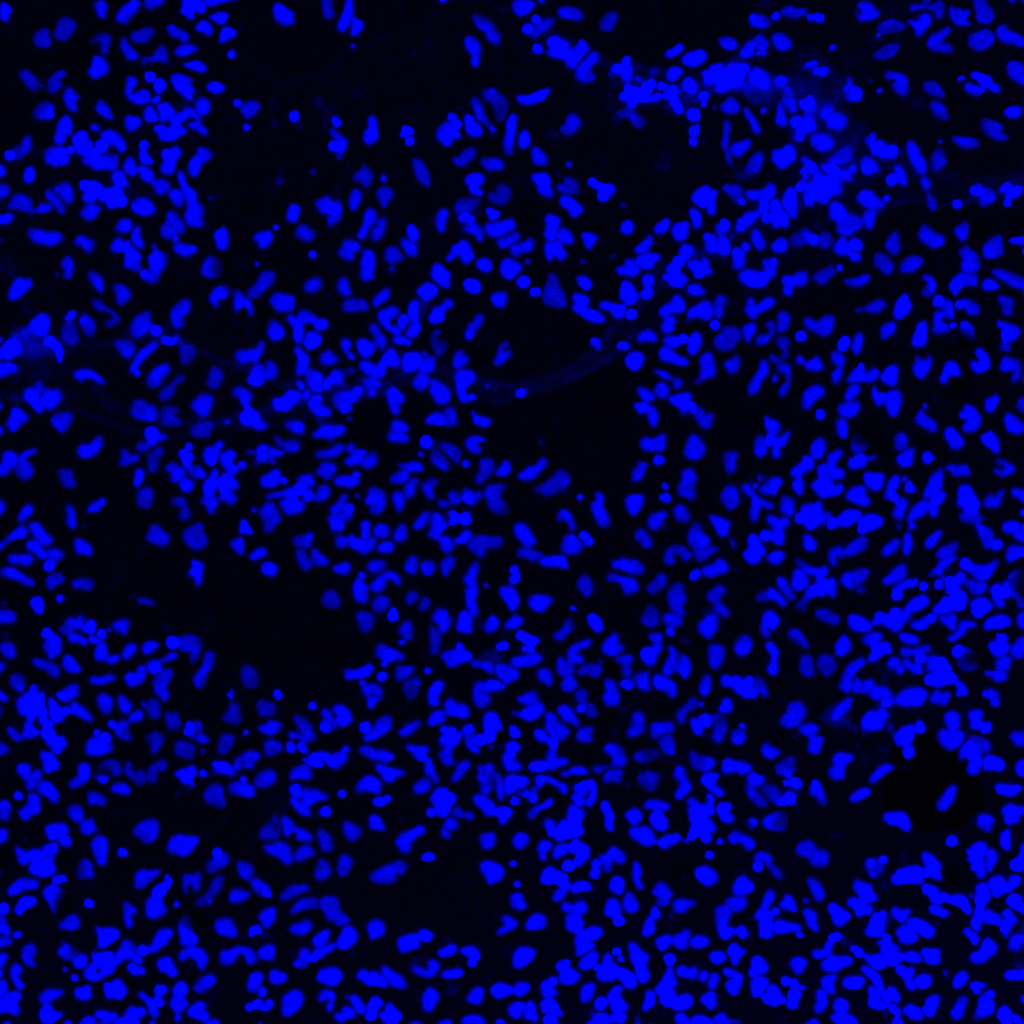

Supplement: Supplementary file 12 — Figure EV4 Source Data [file 44321_2025_302_MOESM12_ESM.zip › Figure EV4/EV4F/#12-3-DAPI.tif]

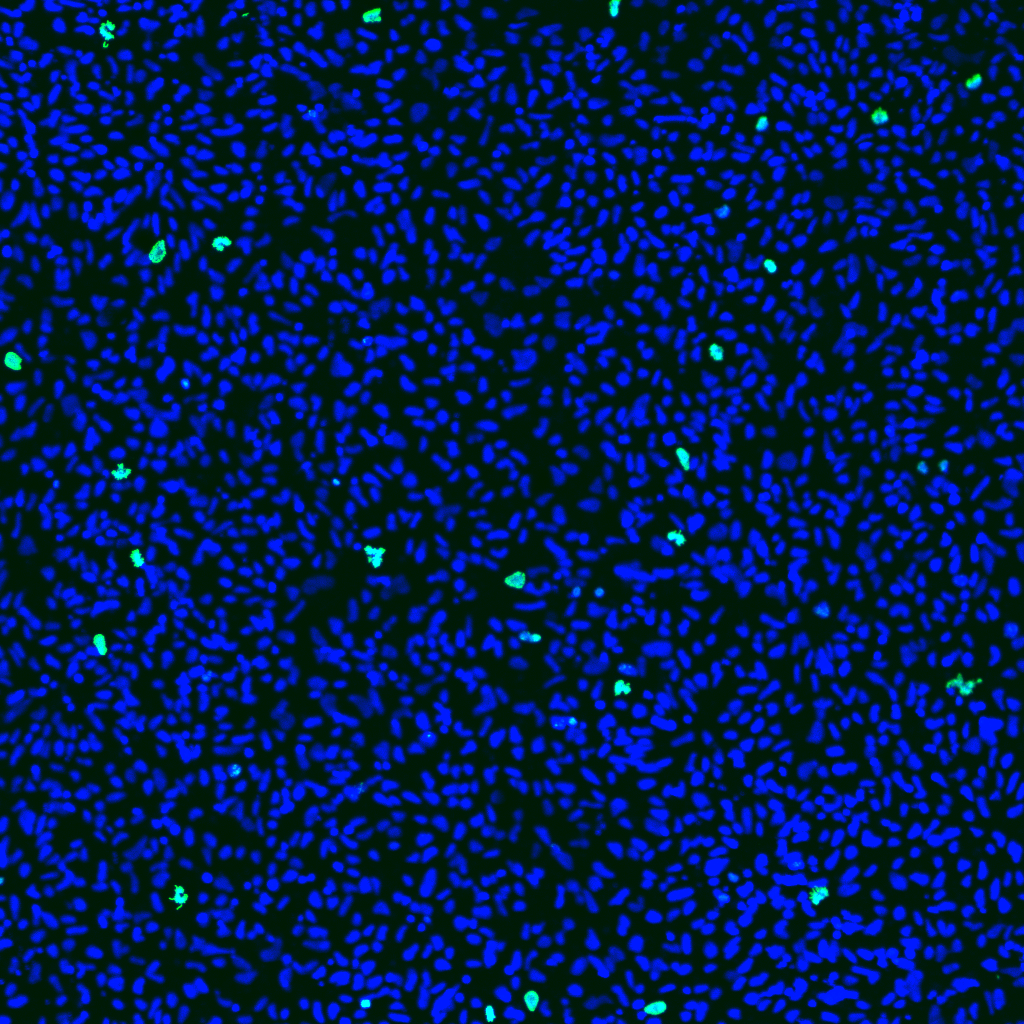

Supplement: Supplementary file 12 — Figure EV4 Source Data [file 44321_2025_302_MOESM12_ESM.zip › Figure EV4/EV4D/#12-3-merge.tif]

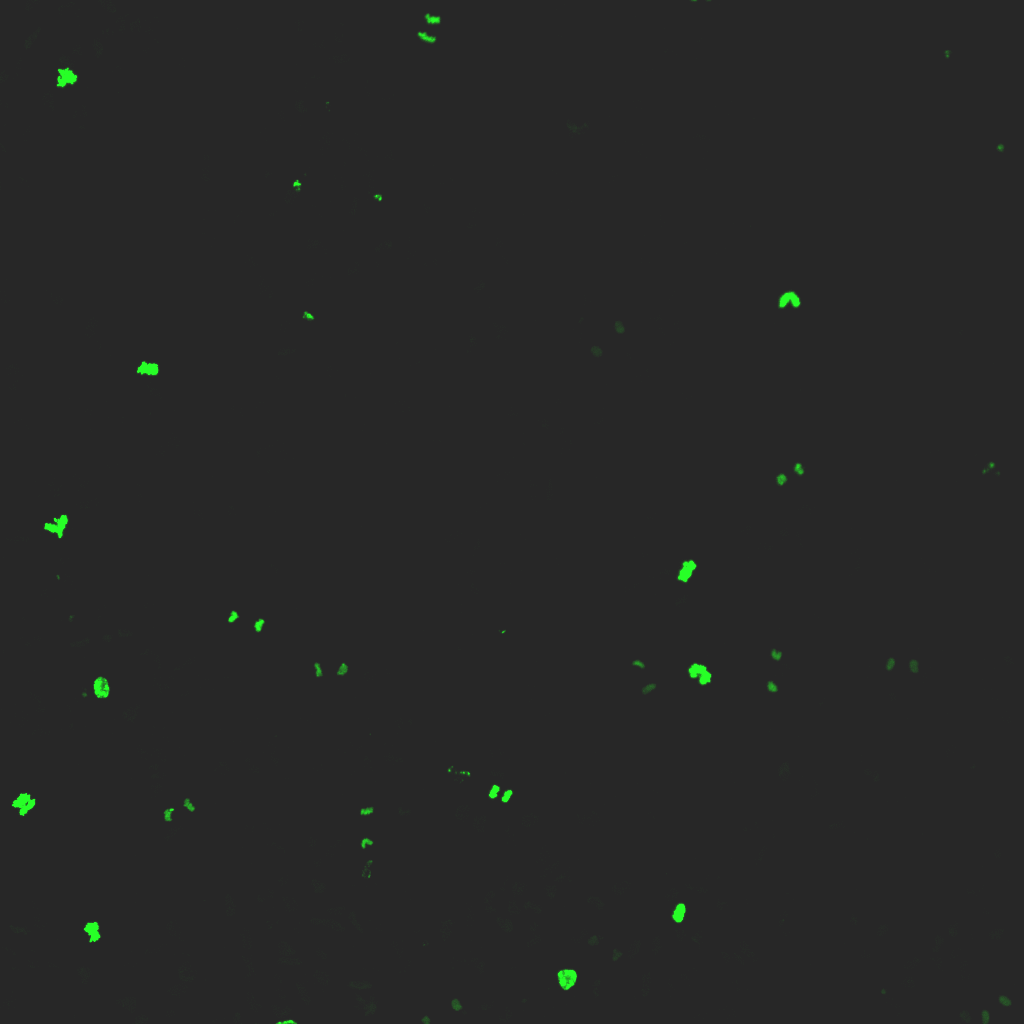

Supplement: Supplementary file 12 — Figure EV4 Source Data [file 44321_2025_302_MOESM12_ESM.zip › Figure EV4/EV4D/#7-5-PH3.tif]

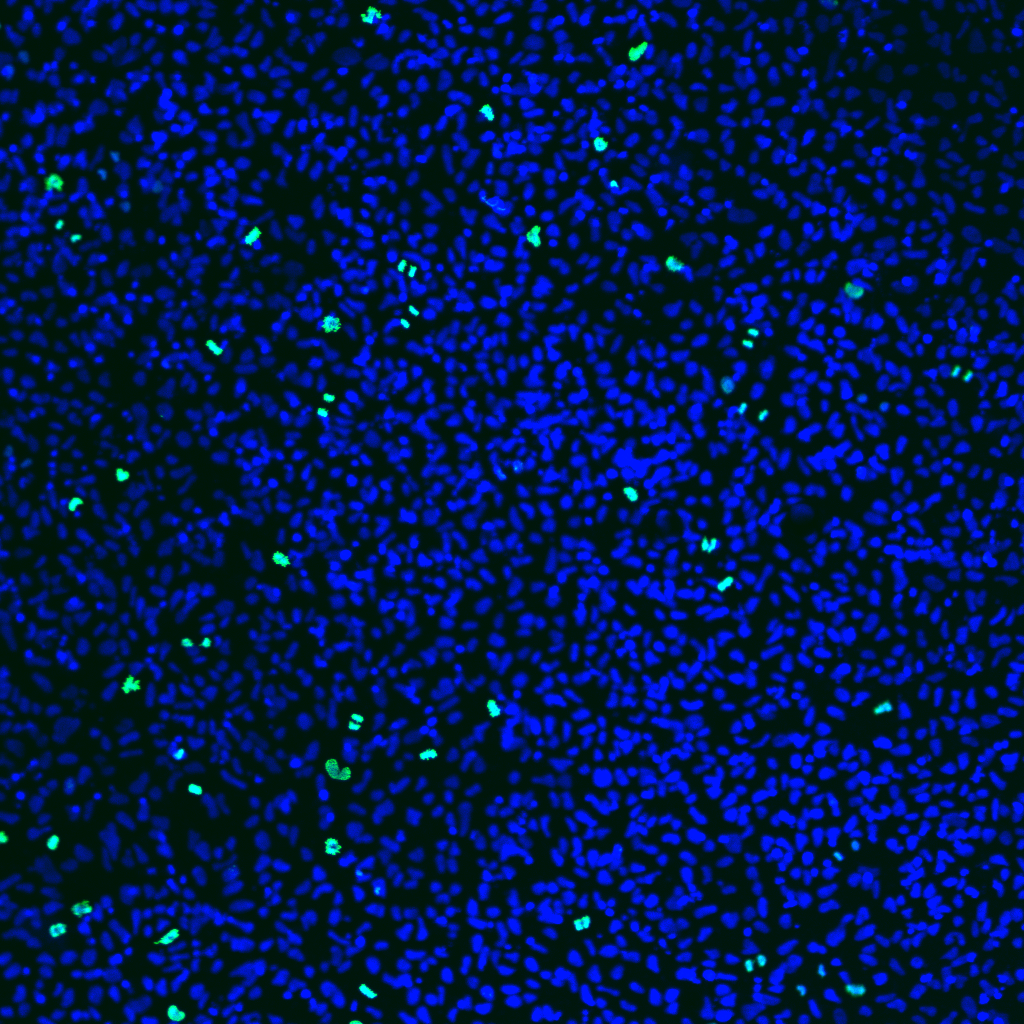

Supplement: Supplementary file 12 — Figure EV4 Source Data [file 44321_2025_302_MOESM12_ESM.zip › Figure EV4/EV4D/H9-merge.tif]

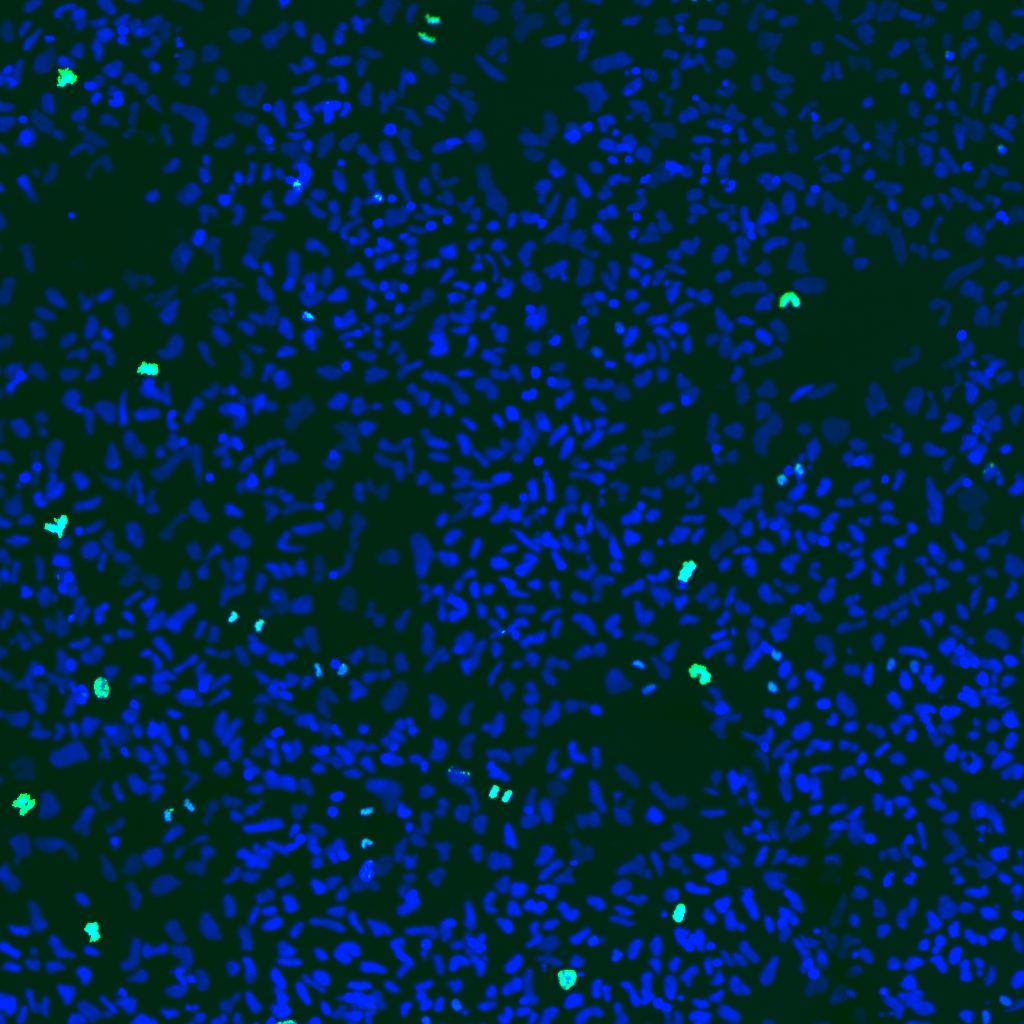

Supplement: Supplementary file 12 — Figure EV4 Source Data [file 44321_2025_302_MOESM12_ESM.zip › Figure EV4/EV4D/#7-5-merge.tif]

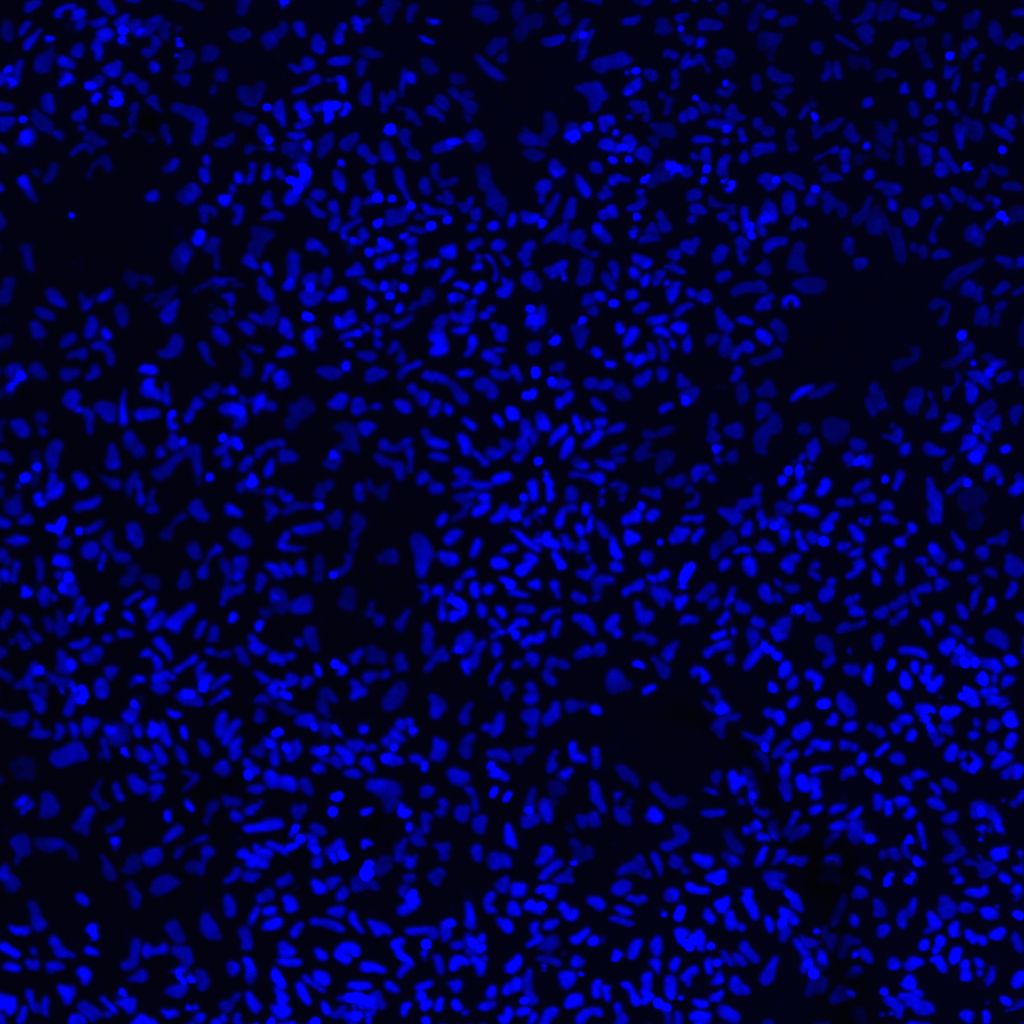

Supplement: Supplementary file 12 — Figure EV4 Source Data [file 44321_2025_302_MOESM12_ESM.zip › Figure EV4/EV4D/#7-5-DAPI.tif]

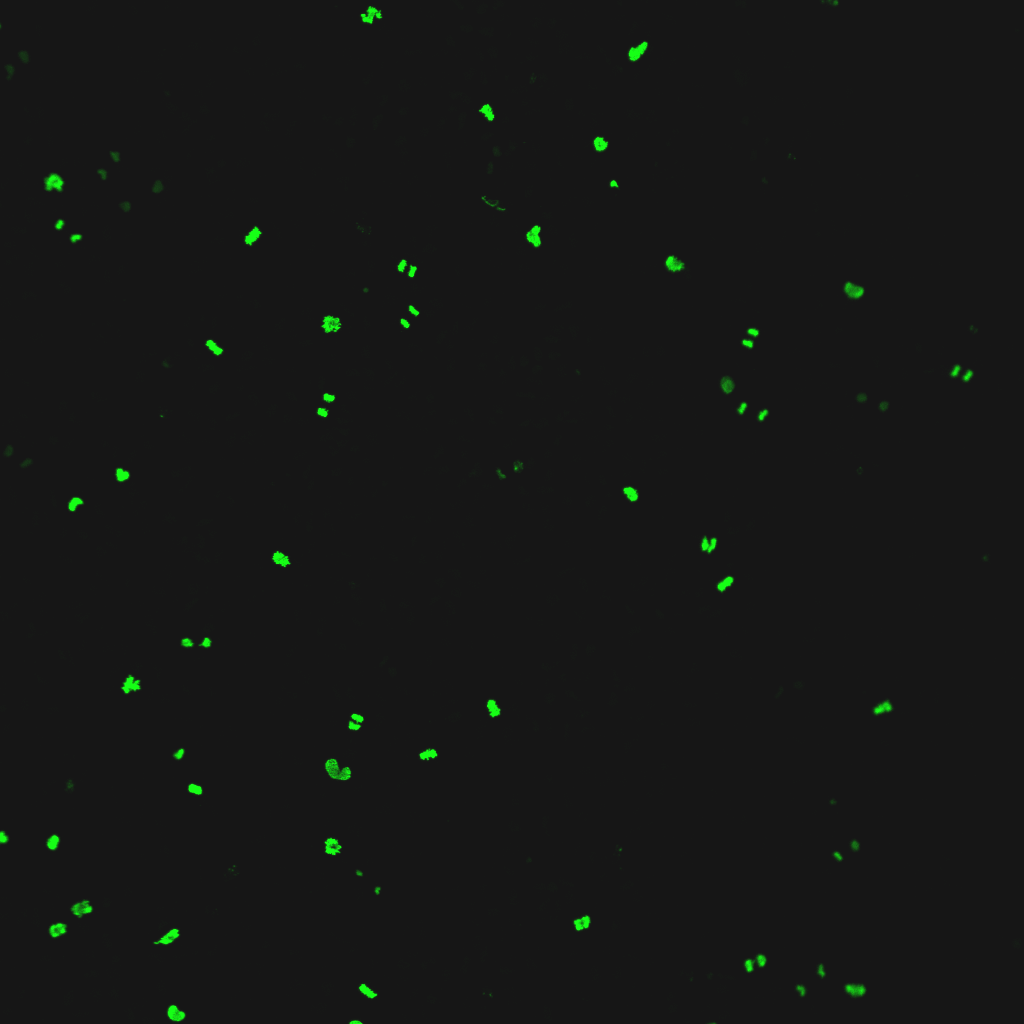

Supplement: Supplementary file 12 — Figure EV4 Source Data [file 44321_2025_302_MOESM12_ESM.zip › Figure EV4/EV4D/H9-PH3.tif]

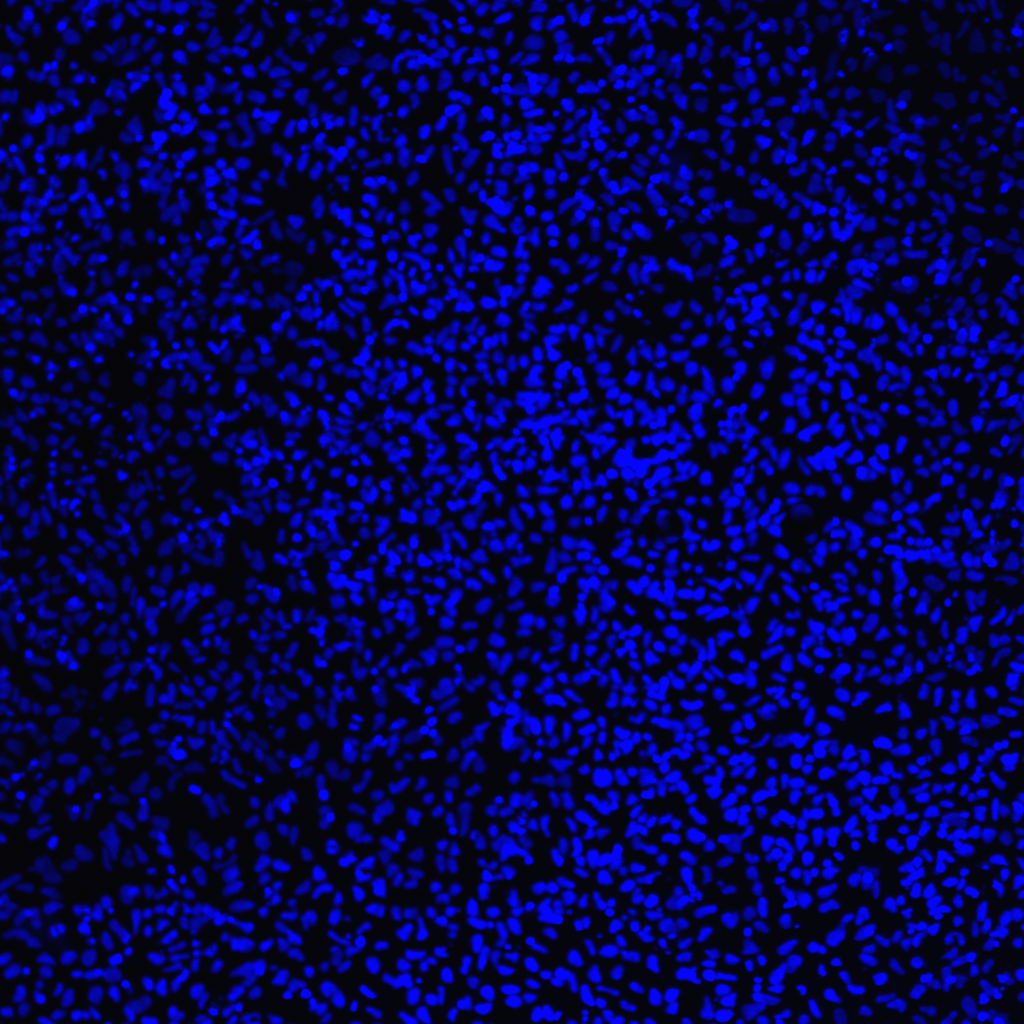

Supplement: Supplementary file 12 — Figure EV4 Source Data [file 44321_2025_302_MOESM12_ESM.zip › Figure EV4/EV4D/H9-DAPI.tif]

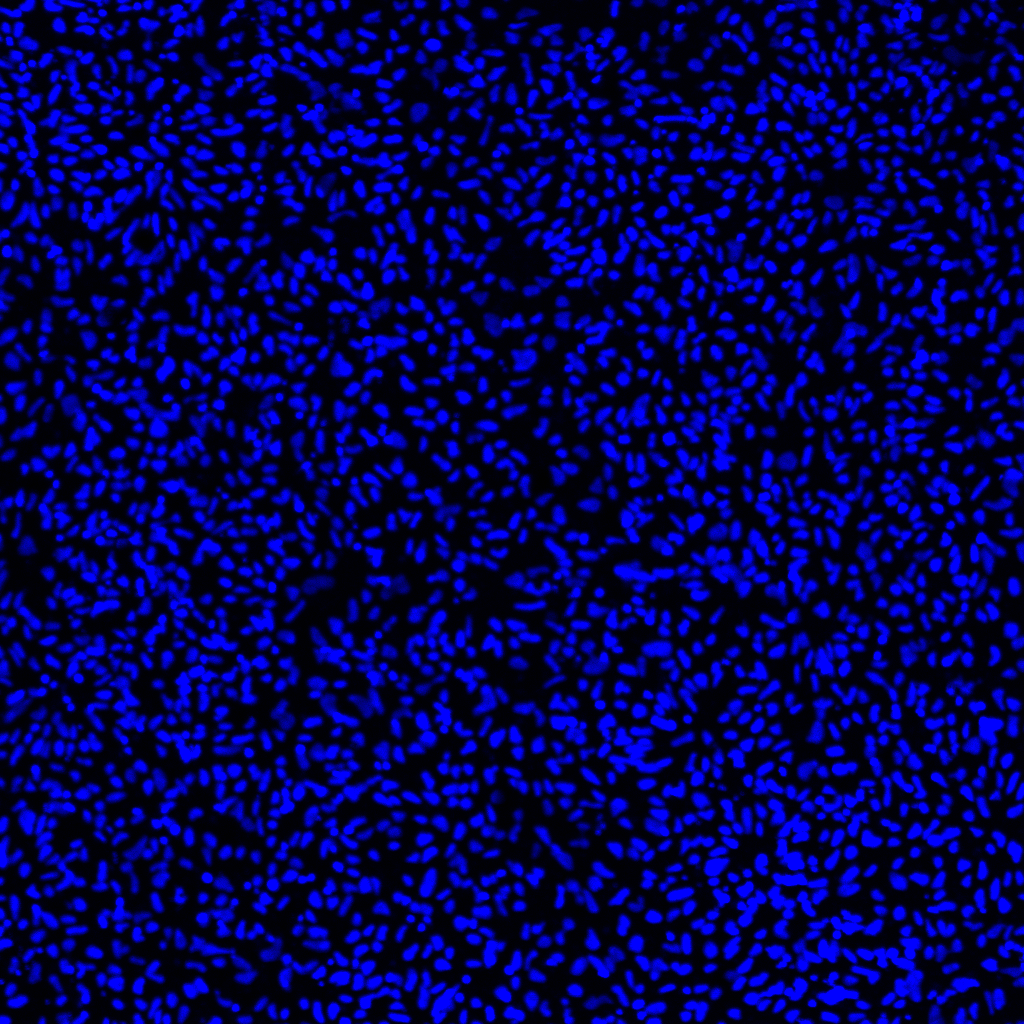

Supplement: Supplementary file 12 — Figure EV4 Source Data [file 44321_2025_302_MOESM12_ESM.zip › Figure EV4/EV4D/#12-3-DAPI.tif]

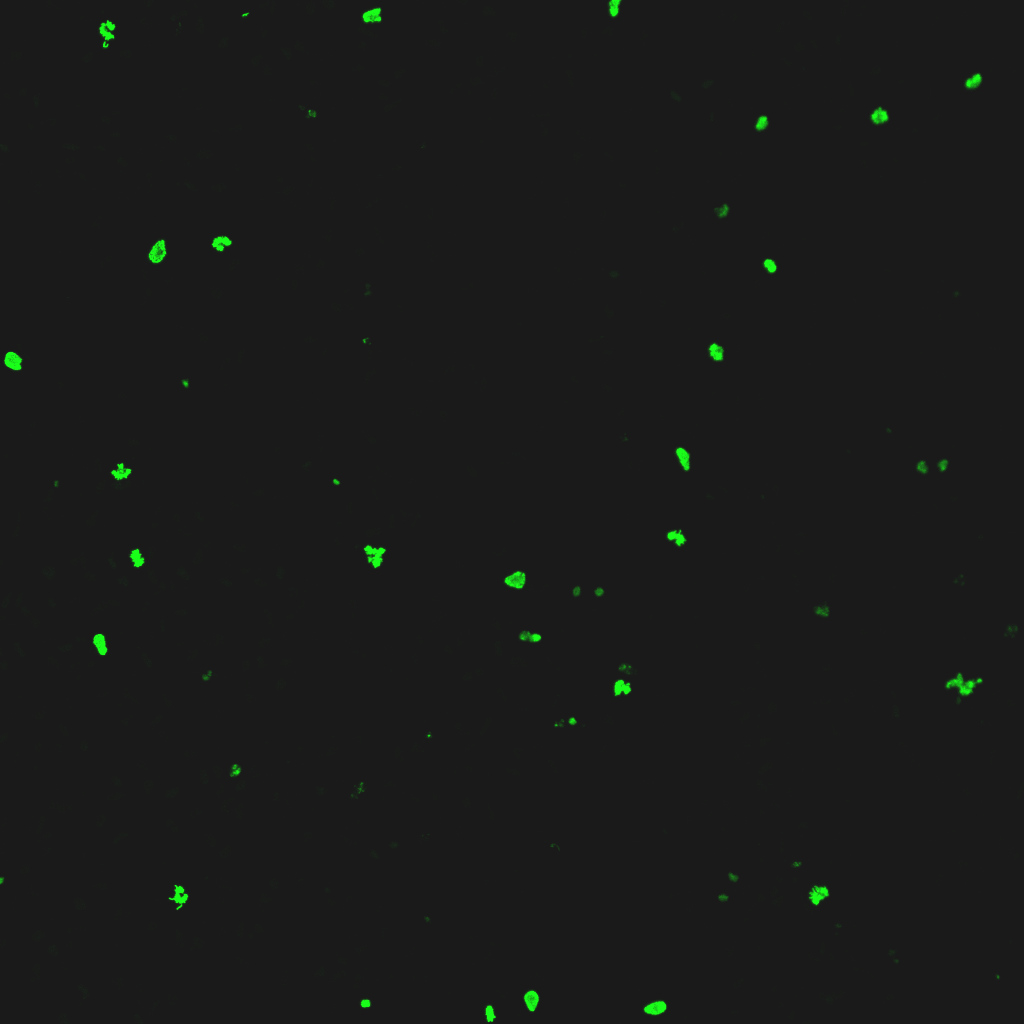

Supplement: Supplementary file 12 — Figure EV4 Source Data [file 44321_2025_302_MOESM12_ESM.zip › Figure EV4/EV4D/#12-3-PH3.tif]

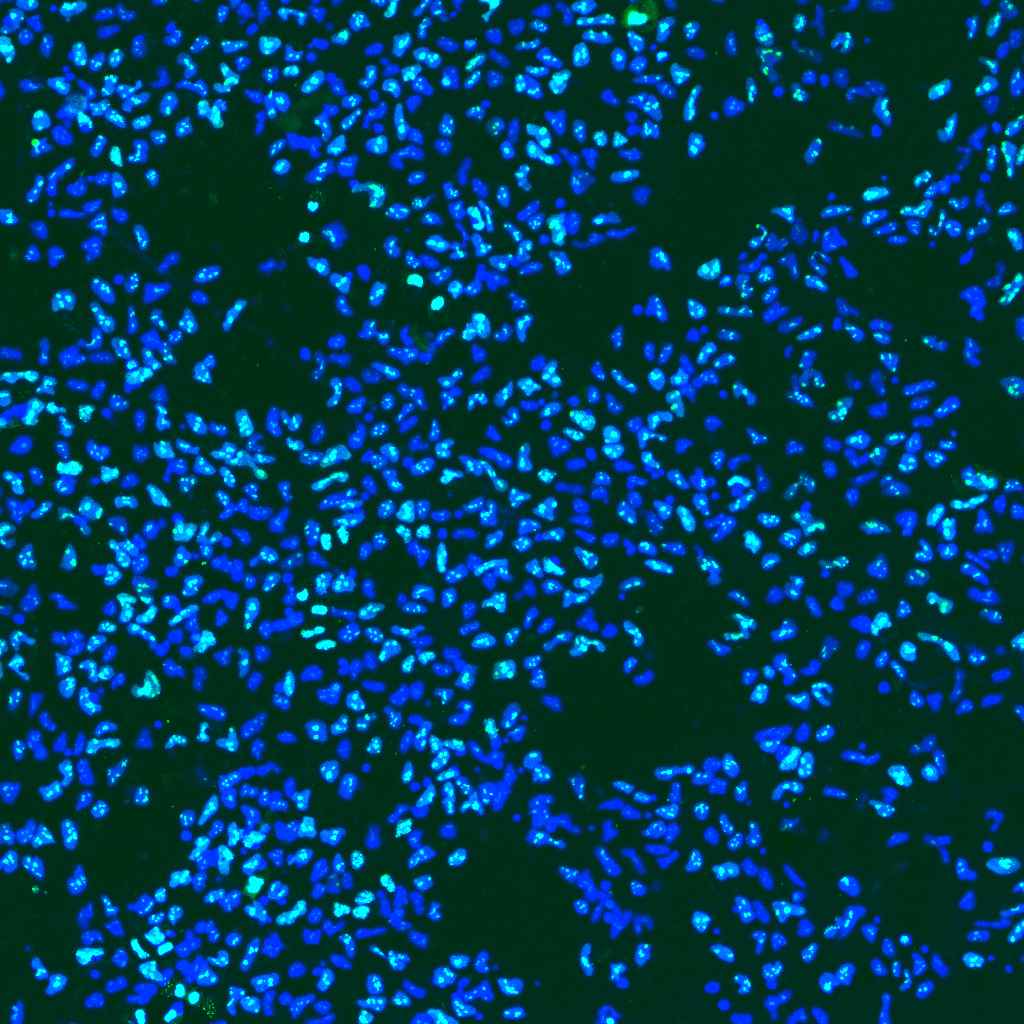

Supplement: Supplementary file 12 — Figure EV4 Source Data [file 44321_2025_302_MOESM12_ESM.zip › Figure EV4/EV4C/#12-3-merge.tif]

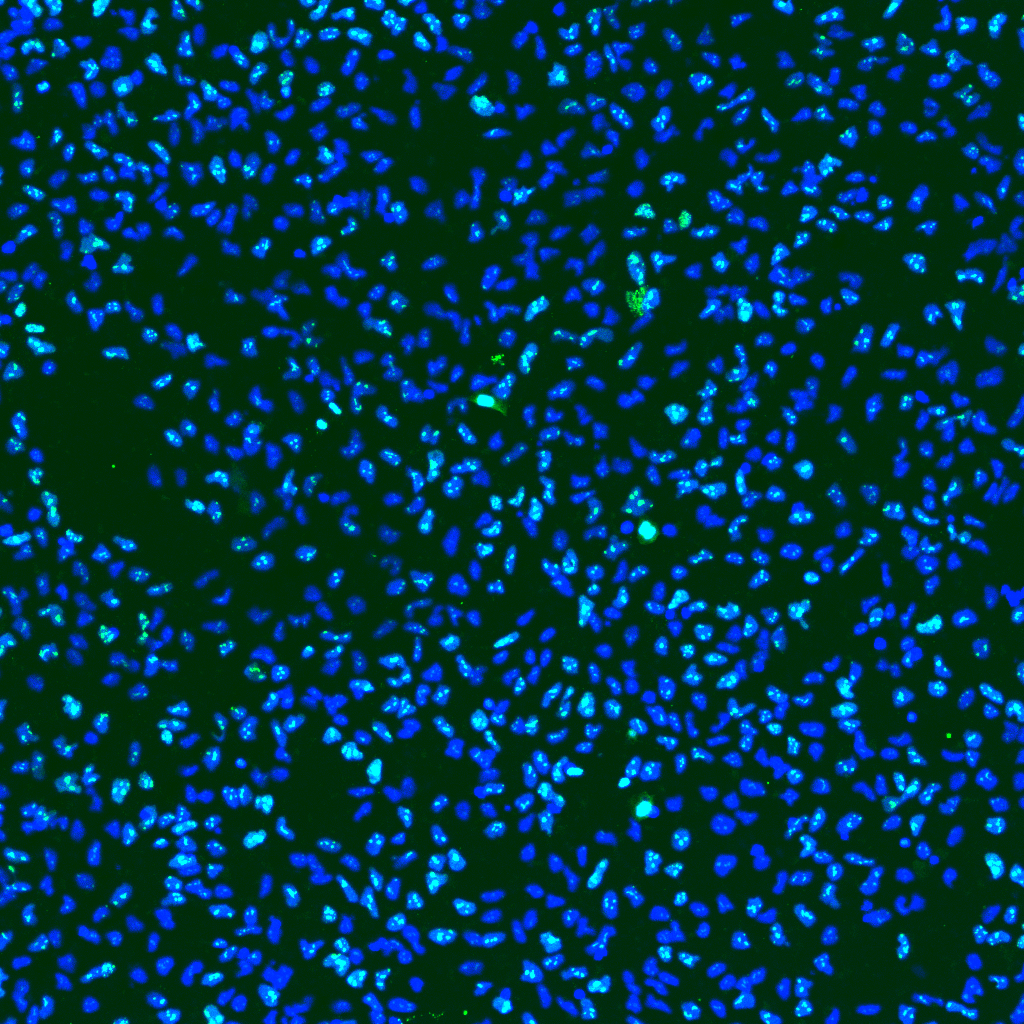

Supplement: Supplementary file 12 — Figure EV4 Source Data [file 44321_2025_302_MOESM12_ESM.zip › Figure EV4/EV4C/H9-merge.tif]

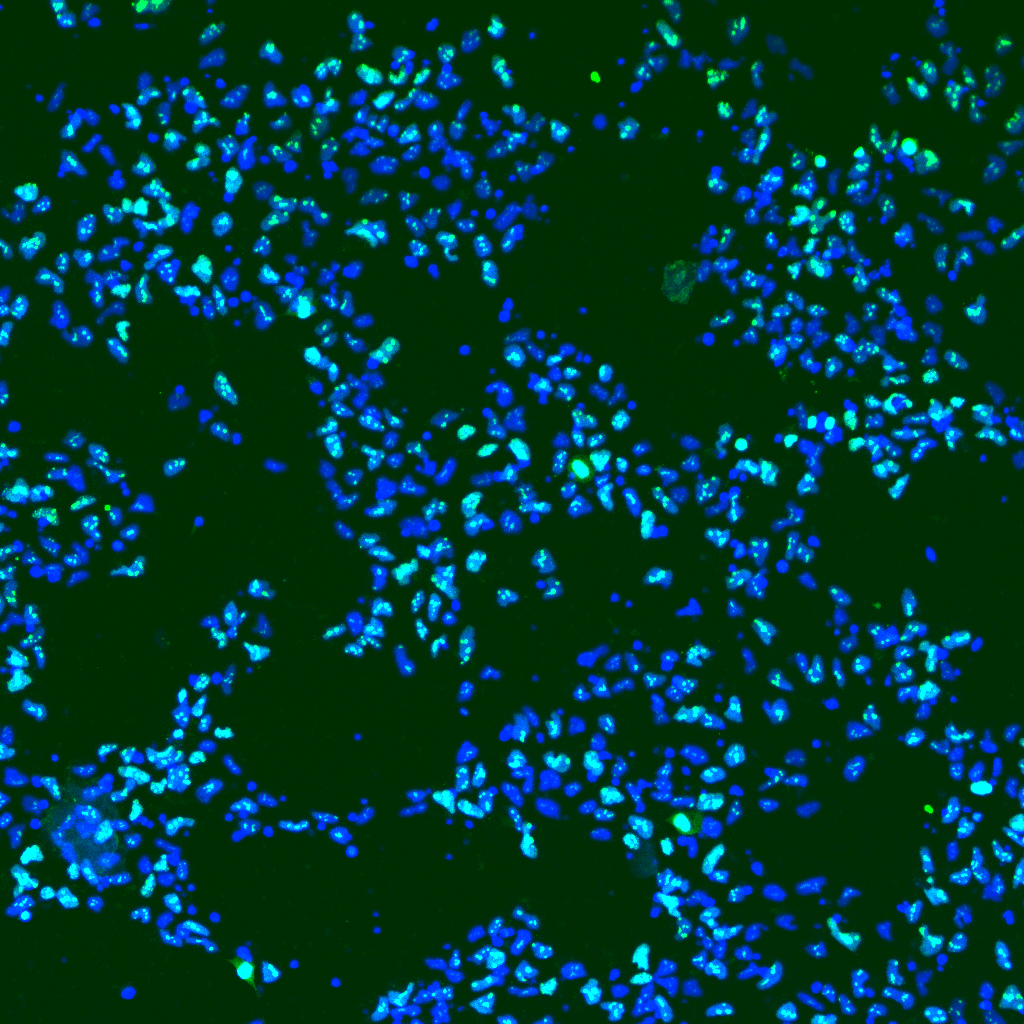

Supplement: Supplementary file 12 — Figure EV4 Source Data [file 44321_2025_302_MOESM12_ESM.zip › Figure EV4/EV4C/#7-5-merge.tif]

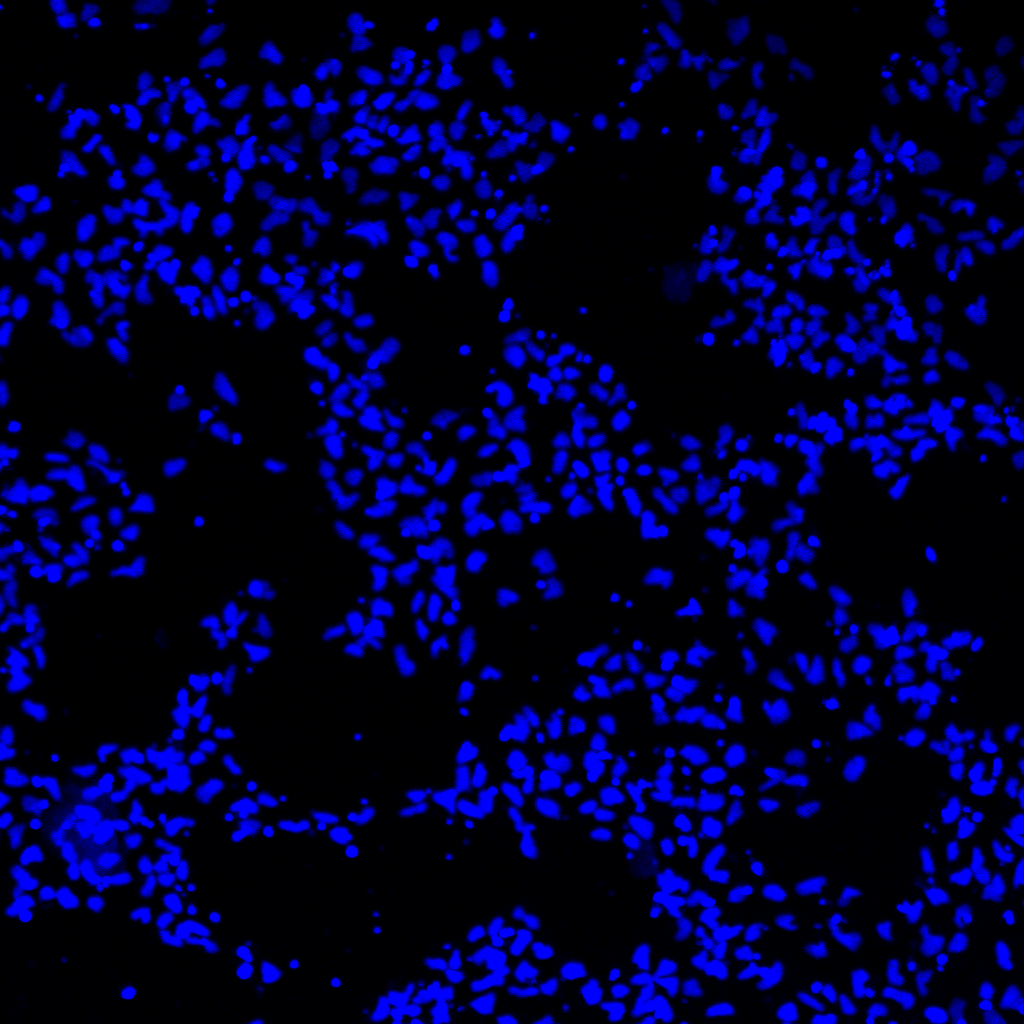

Supplement: Supplementary file 12 — Figure EV4 Source Data [file 44321_2025_302_MOESM12_ESM.zip › Figure EV4/EV4C/#7-5-DAPI.tif]

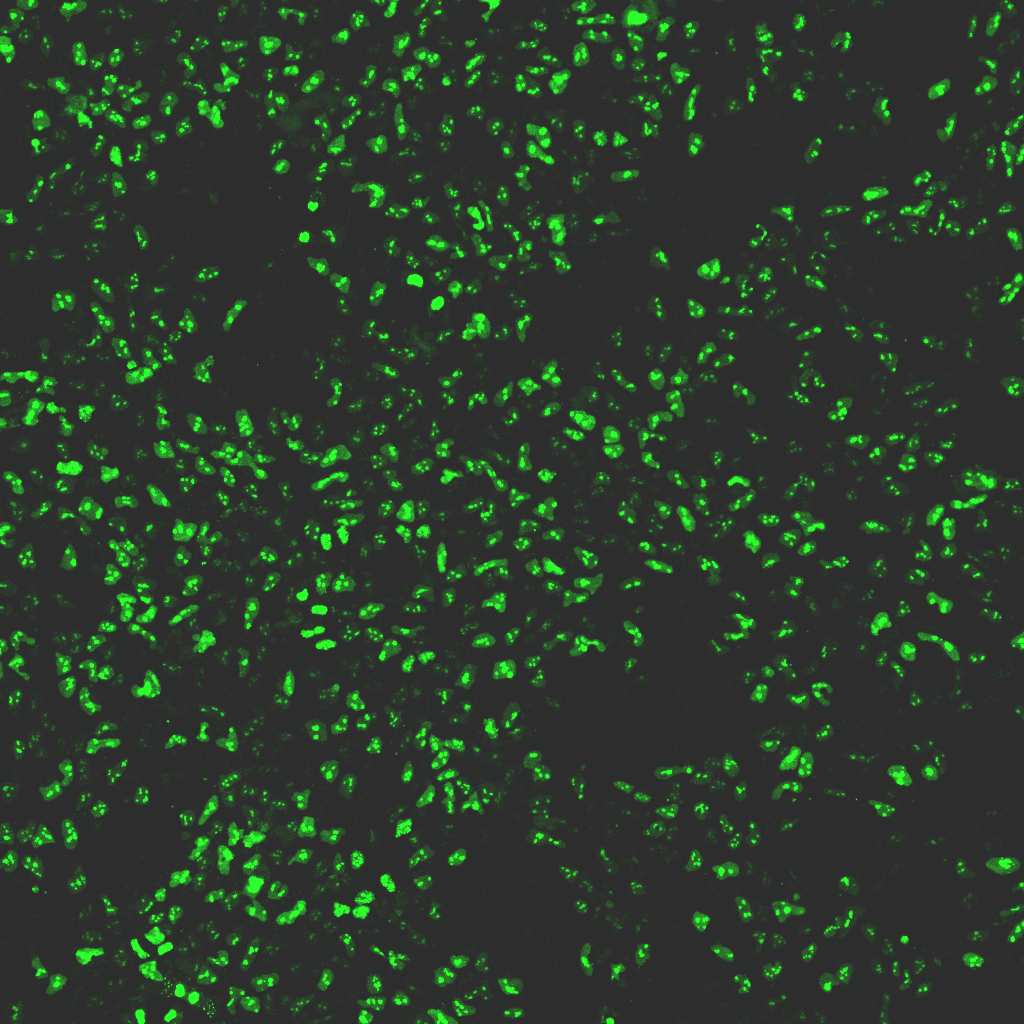

Supplement: Supplementary file 12 — Figure EV4 Source Data [file 44321_2025_302_MOESM12_ESM.zip › Figure EV4/EV4C/#12-3-Ki67.tif]

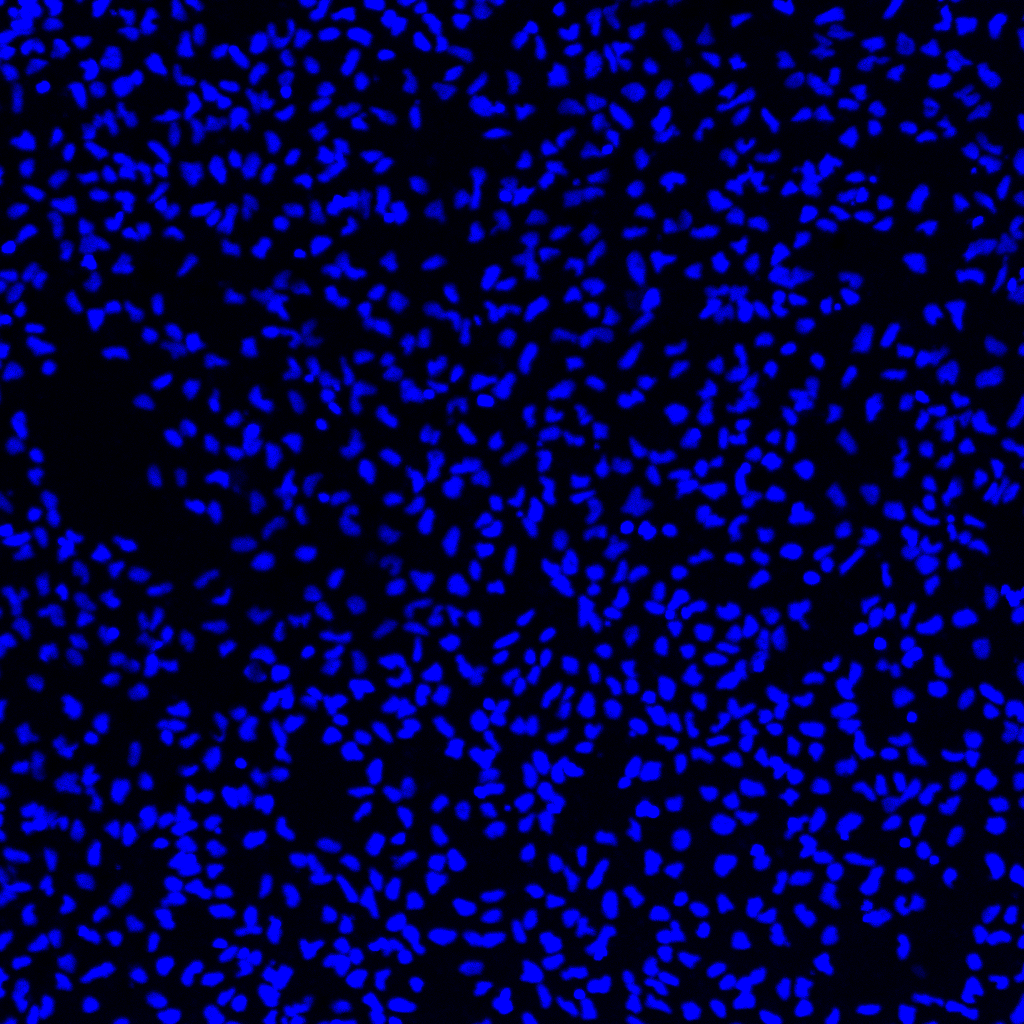

Supplement: Supplementary file 12 — Figure EV4 Source Data [file 44321_2025_302_MOESM12_ESM.zip › Figure EV4/EV4C/H9-DAPI.tif]

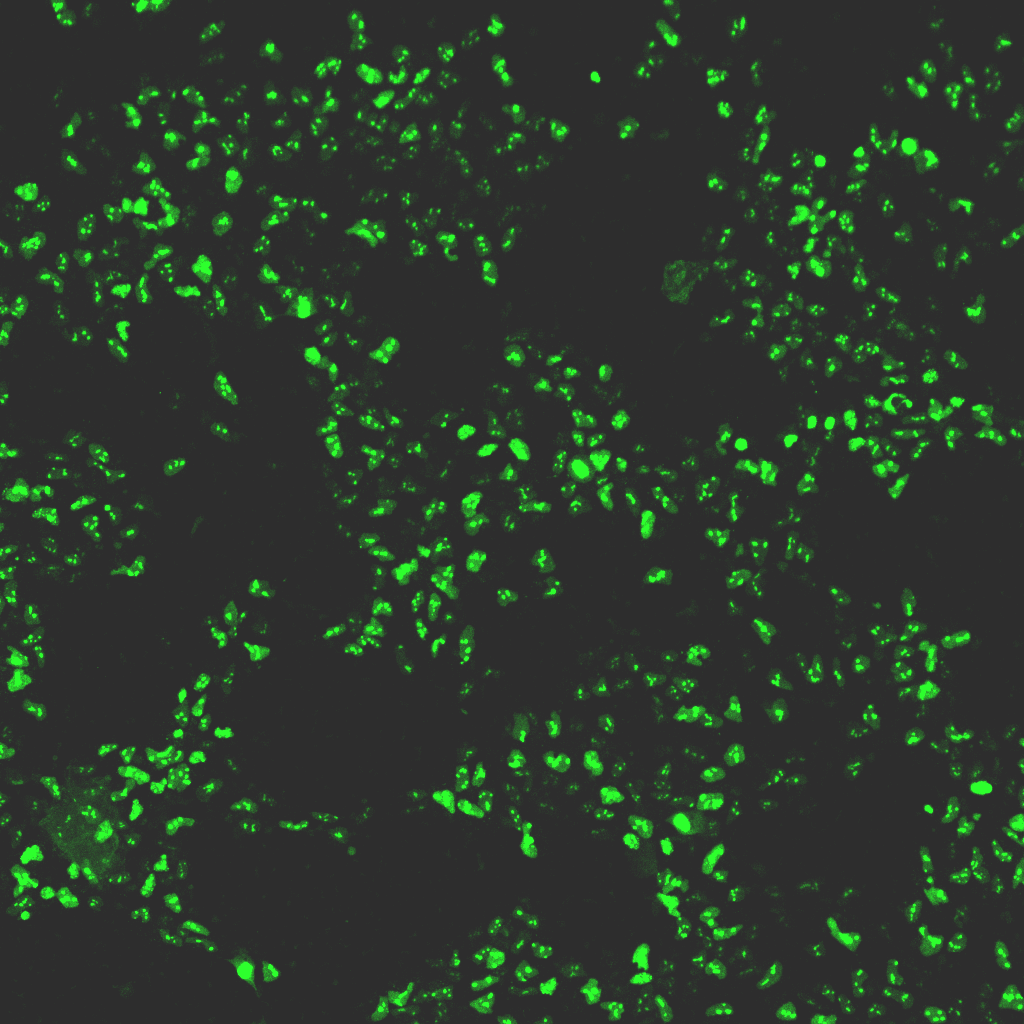

Supplement: Supplementary file 12 — Figure EV4 Source Data [file 44321_2025_302_MOESM12_ESM.zip › Figure EV4/EV4C/#7-5-Ki67.tif]

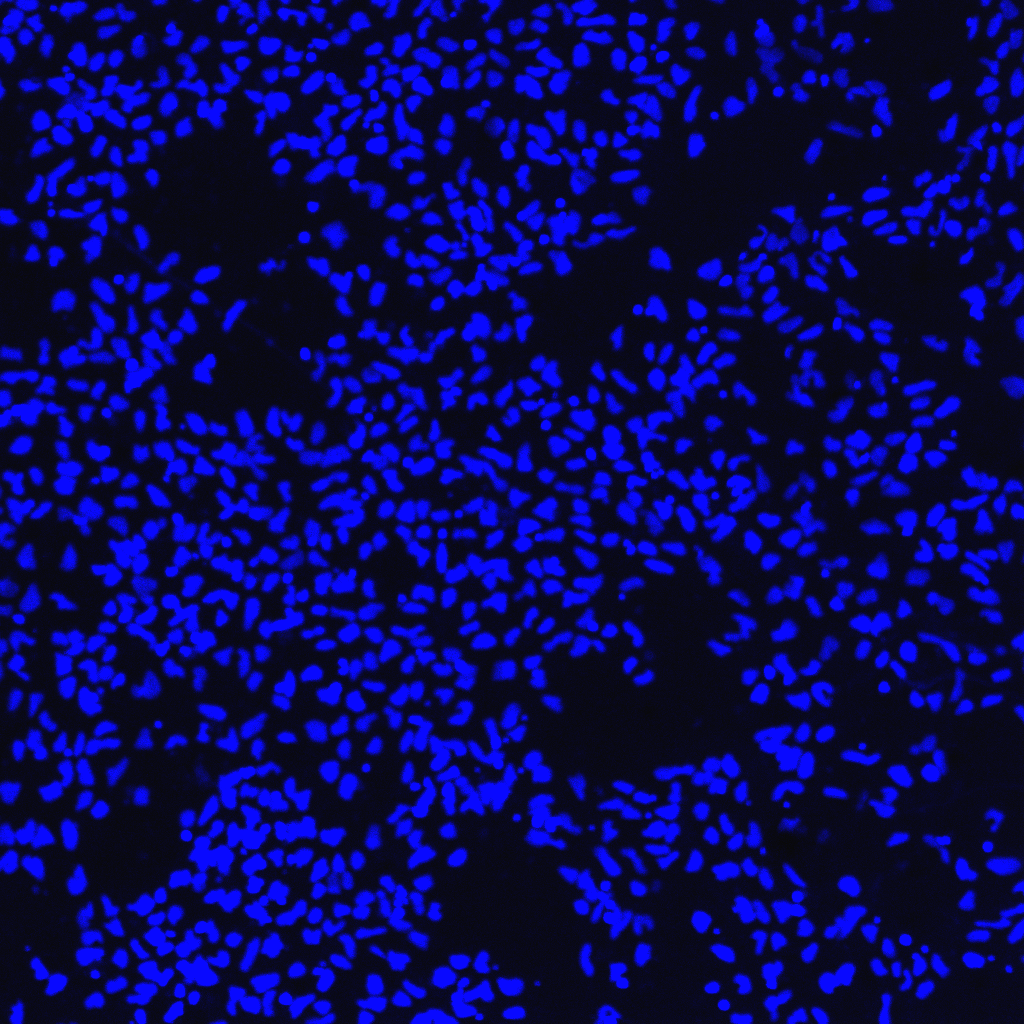

Supplement: Supplementary file 12 — Figure EV4 Source Data [file 44321_2025_302_MOESM12_ESM.zip › Figure EV4/EV4C/#12-3-DAPI.tif]

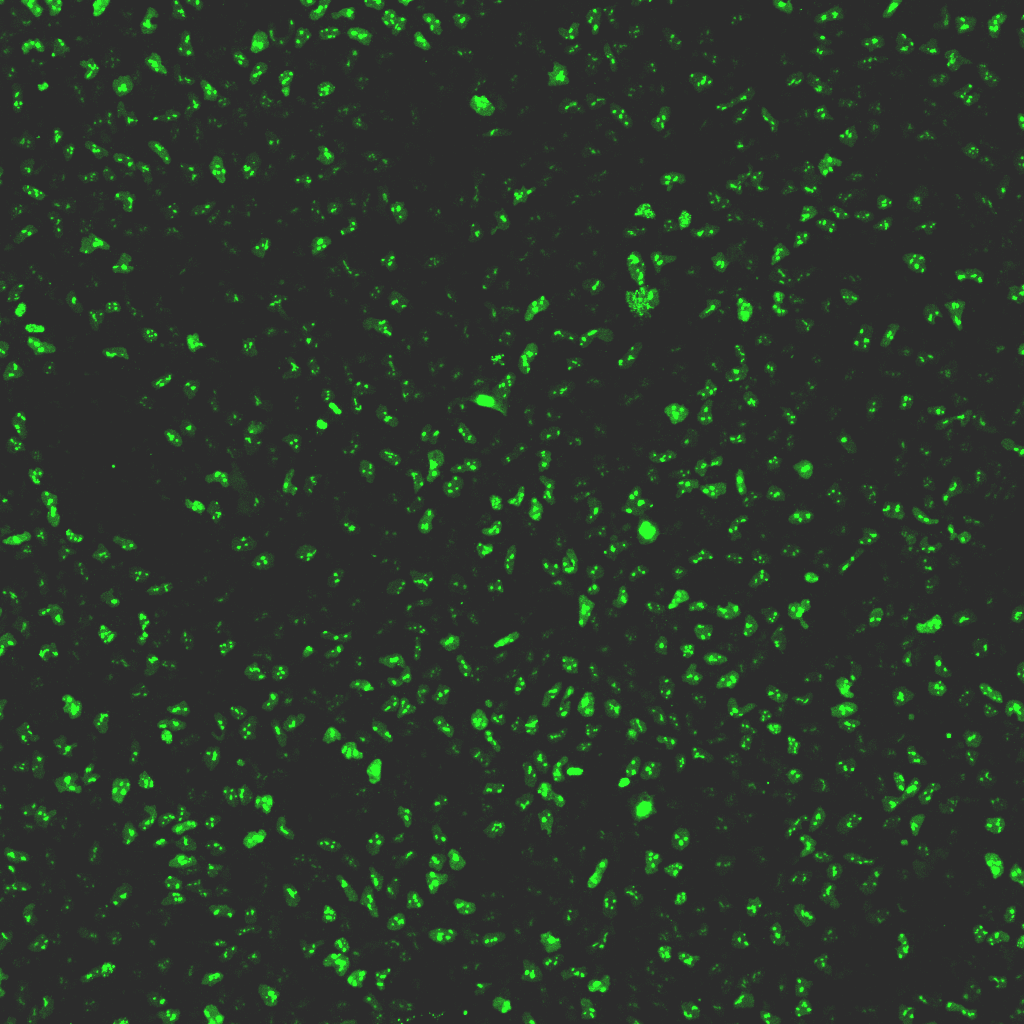

Supplement: Supplementary file 12 — Figure EV4 Source Data [file 44321_2025_302_MOESM12_ESM.zip › Figure EV4/EV4C/H9-Ki67.tif]

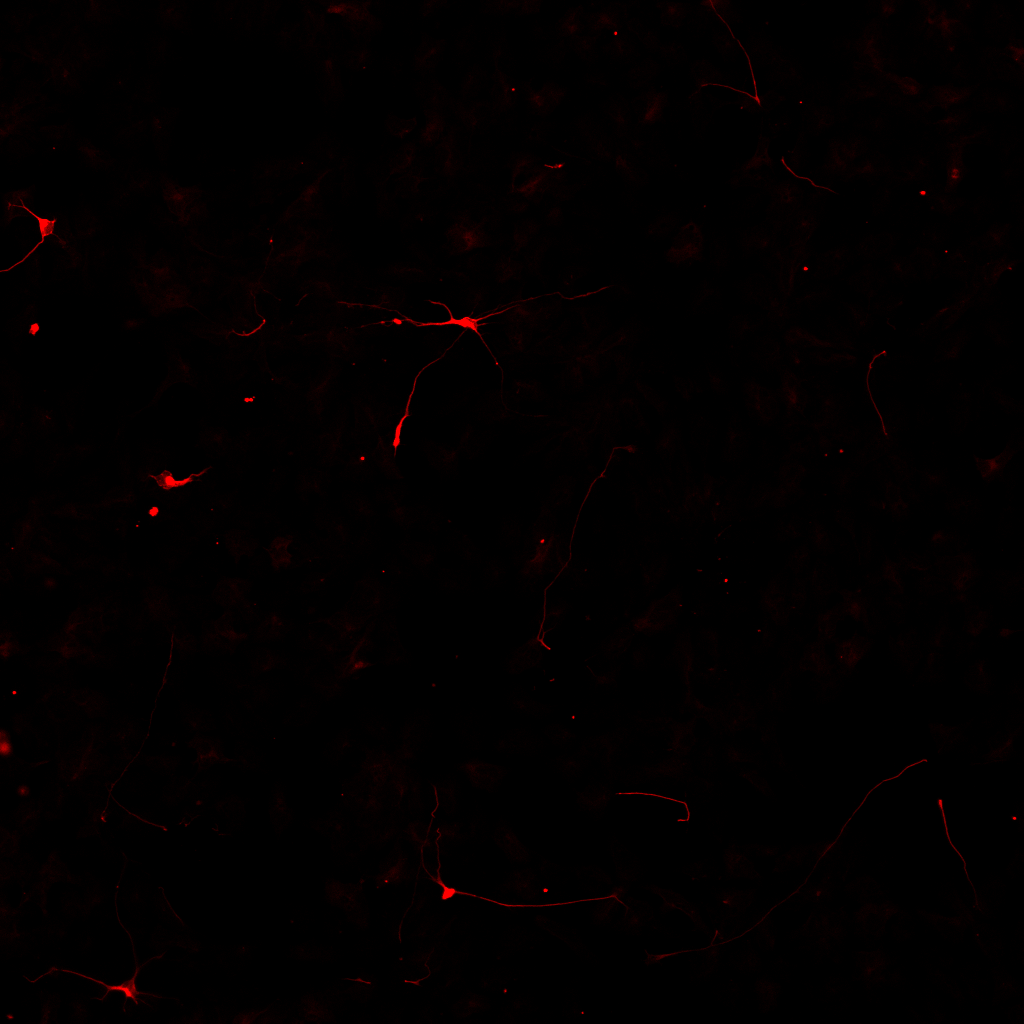

Supplement: Supplementary file 12 — Figure EV4 Source Data [file 44321_2025_302_MOESM12_ESM.zip › Figure EV4/EV4B/H9-TUJ1.tif]

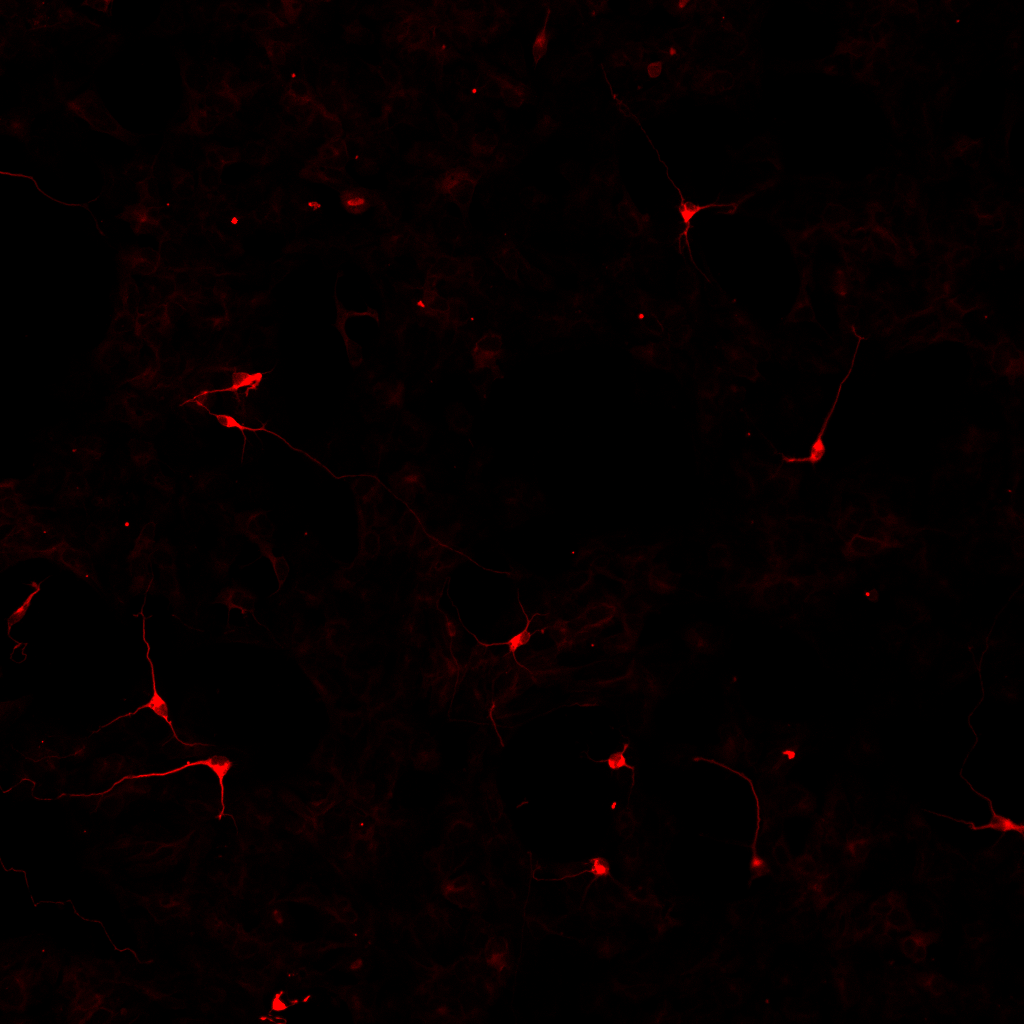

Supplement: Supplementary file 12 — Figure EV4 Source Data [file 44321_2025_302_MOESM12_ESM.zip › Figure EV4/EV4B/#7-5-TUJ1.tif]

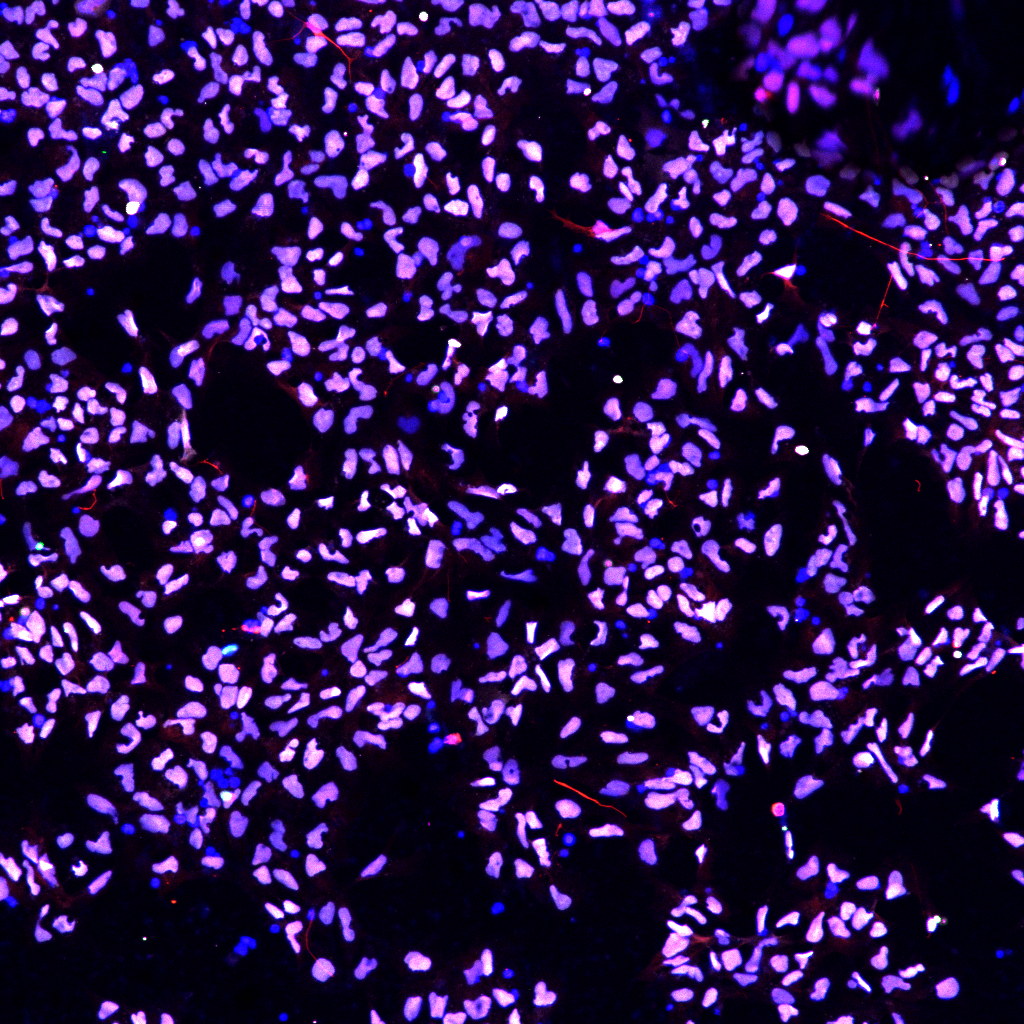

Supplement: Supplementary file 12 — Figure EV4 Source Data [file 44321_2025_302_MOESM12_ESM.zip › Figure EV4/EV4B/#12-3-merge.tif]

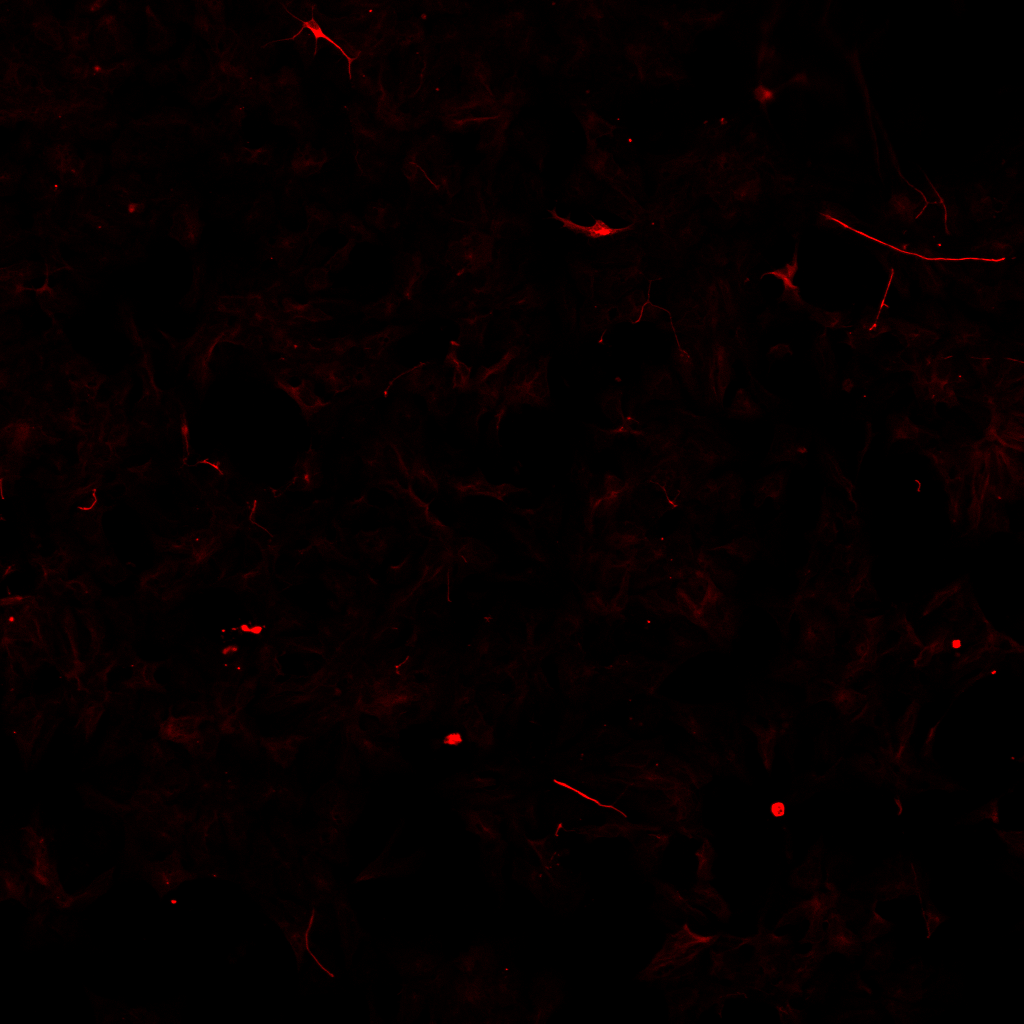

Supplement: Supplementary file 12 — Figure EV4 Source Data [file 44321_2025_302_MOESM12_ESM.zip › Figure EV4/EV4B/#12-3-TUJ1.tif]

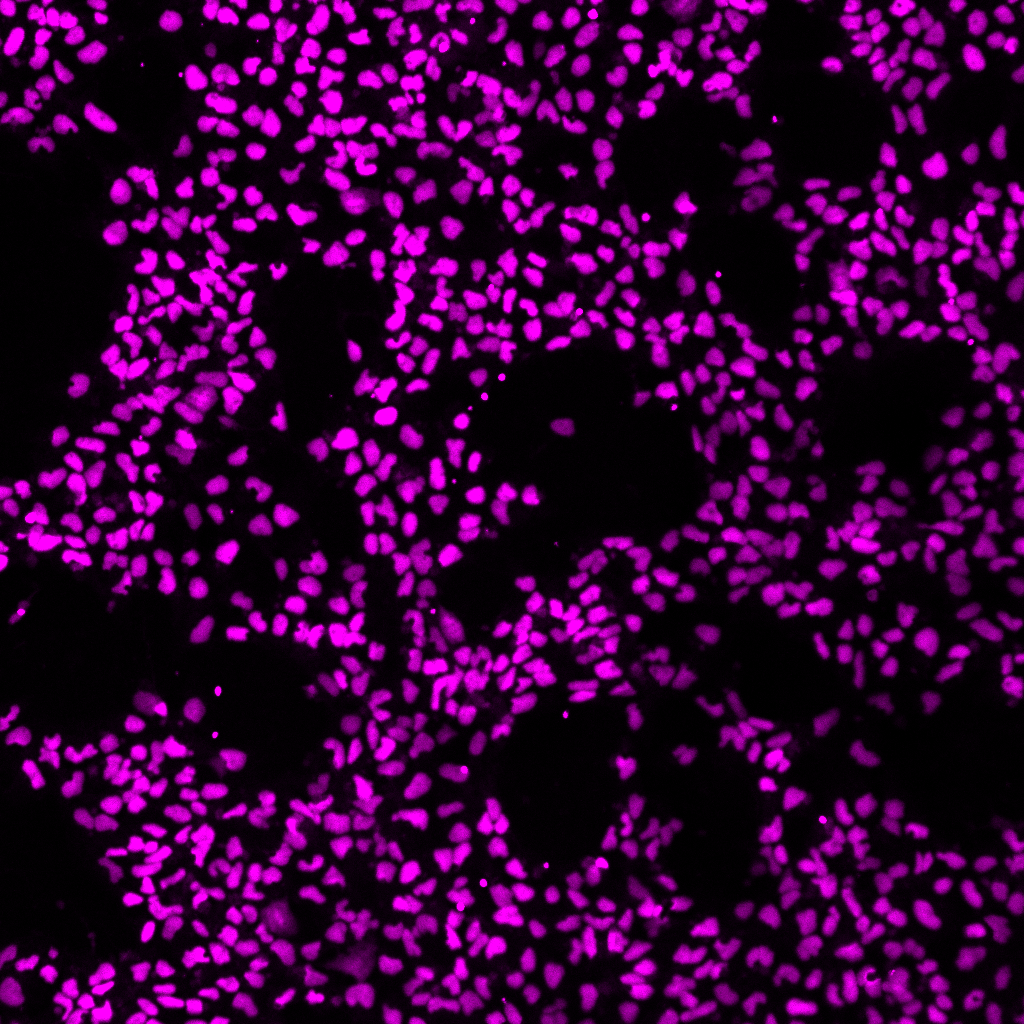

Supplement: Supplementary file 12 — Figure EV4 Source Data [file 44321_2025_302_MOESM12_ESM.zip › Figure EV4/EV4B/#7-5-PAX6.tif]

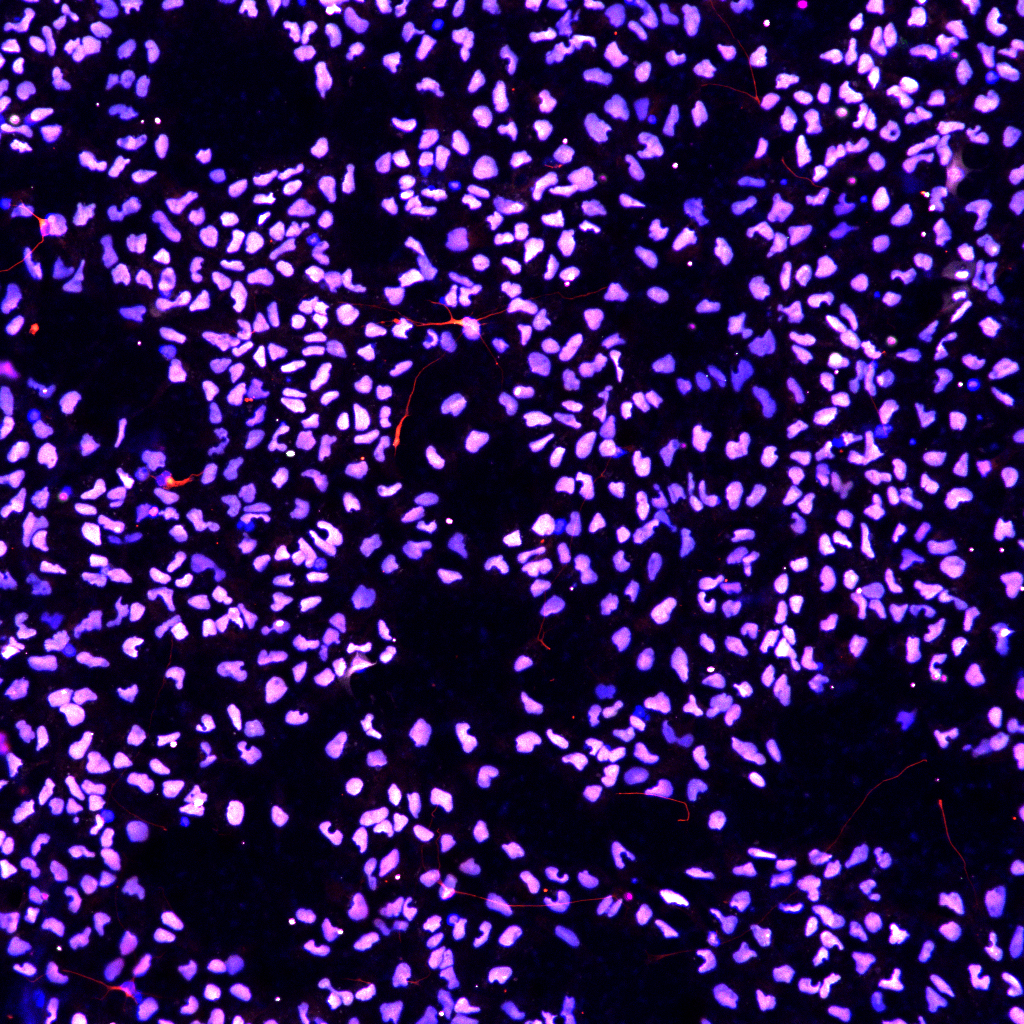

Supplement: Supplementary file 12 — Figure EV4 Source Data [file 44321_2025_302_MOESM12_ESM.zip › Figure EV4/EV4B/H9-merge.tif]

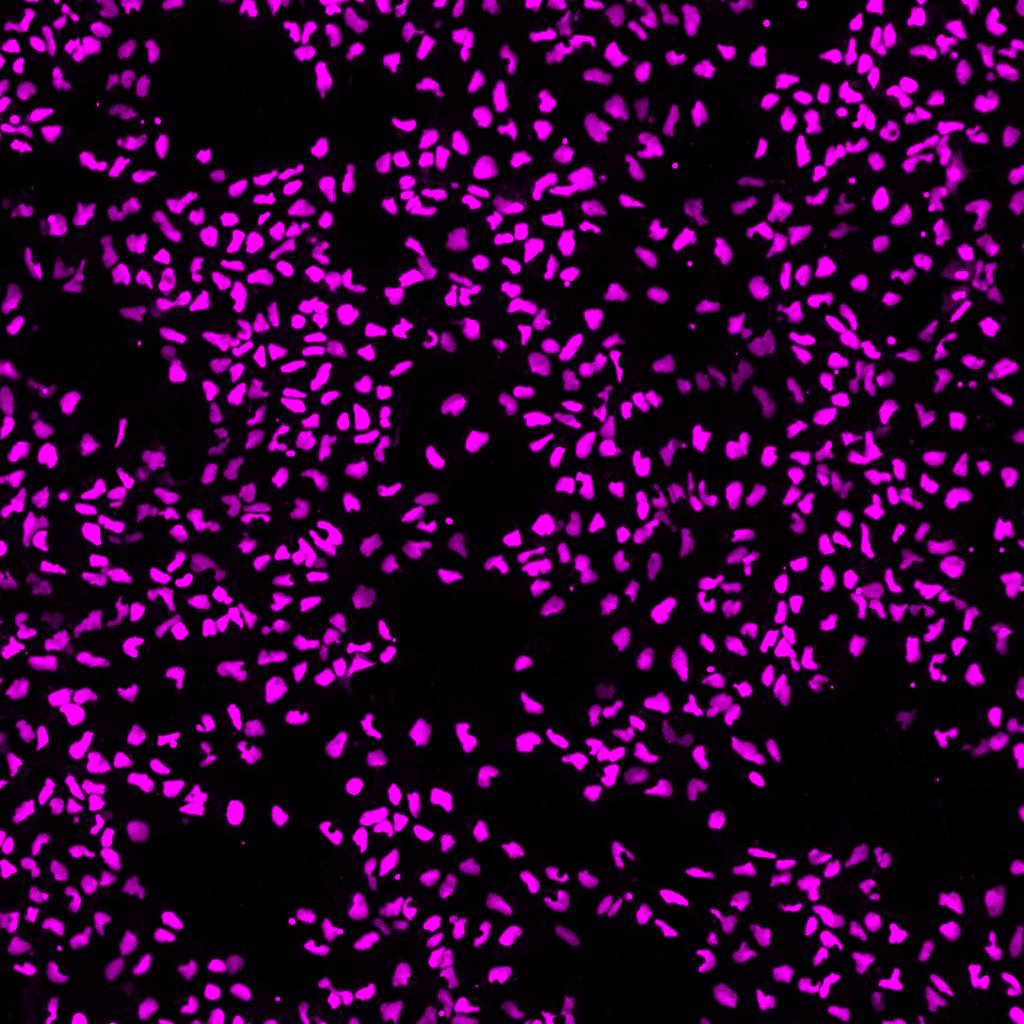

Supplement: Supplementary file 12 — Figure EV4 Source Data [file 44321_2025_302_MOESM12_ESM.zip › Figure EV4/EV4B/H9-PAX6.tif]

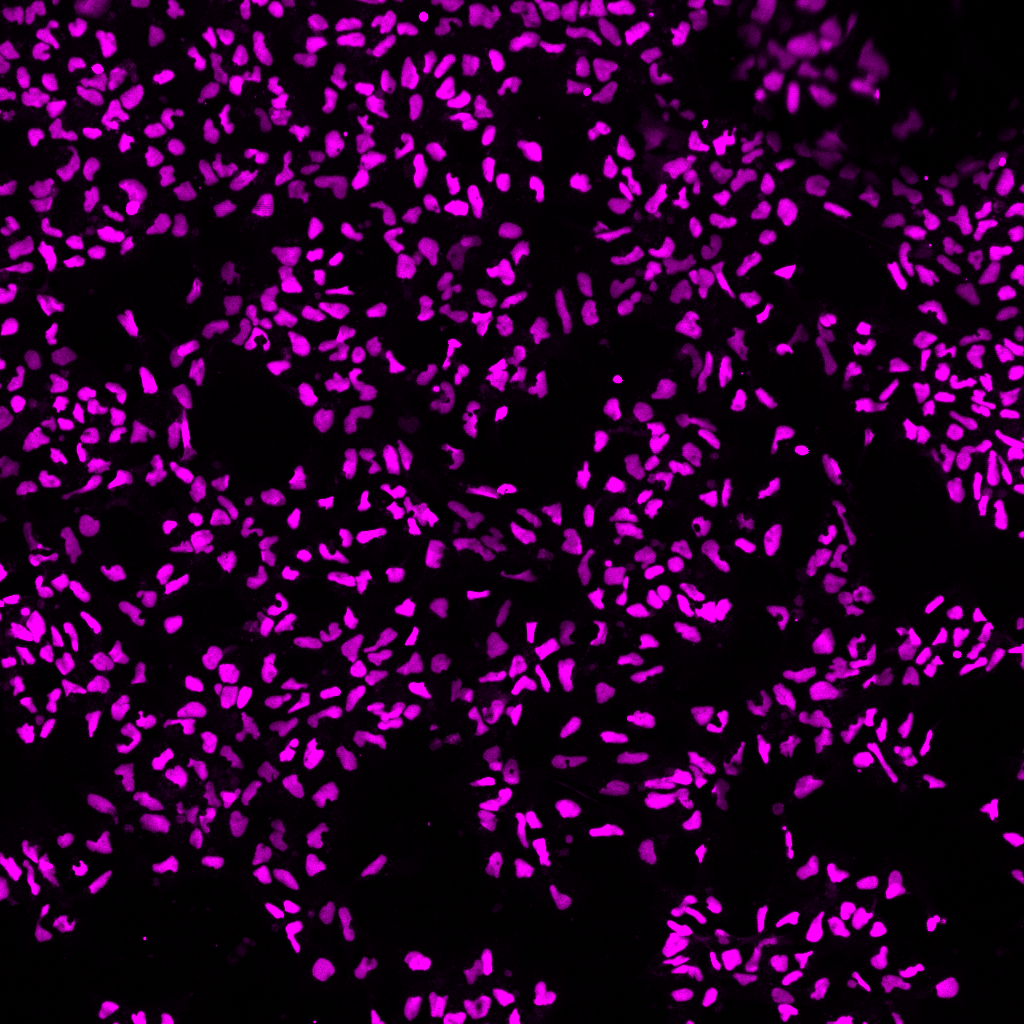

Supplement: Supplementary file 12 — Figure EV4 Source Data [file 44321_2025_302_MOESM12_ESM.zip › Figure EV4/EV4B/#12-3-PAX6.tif]

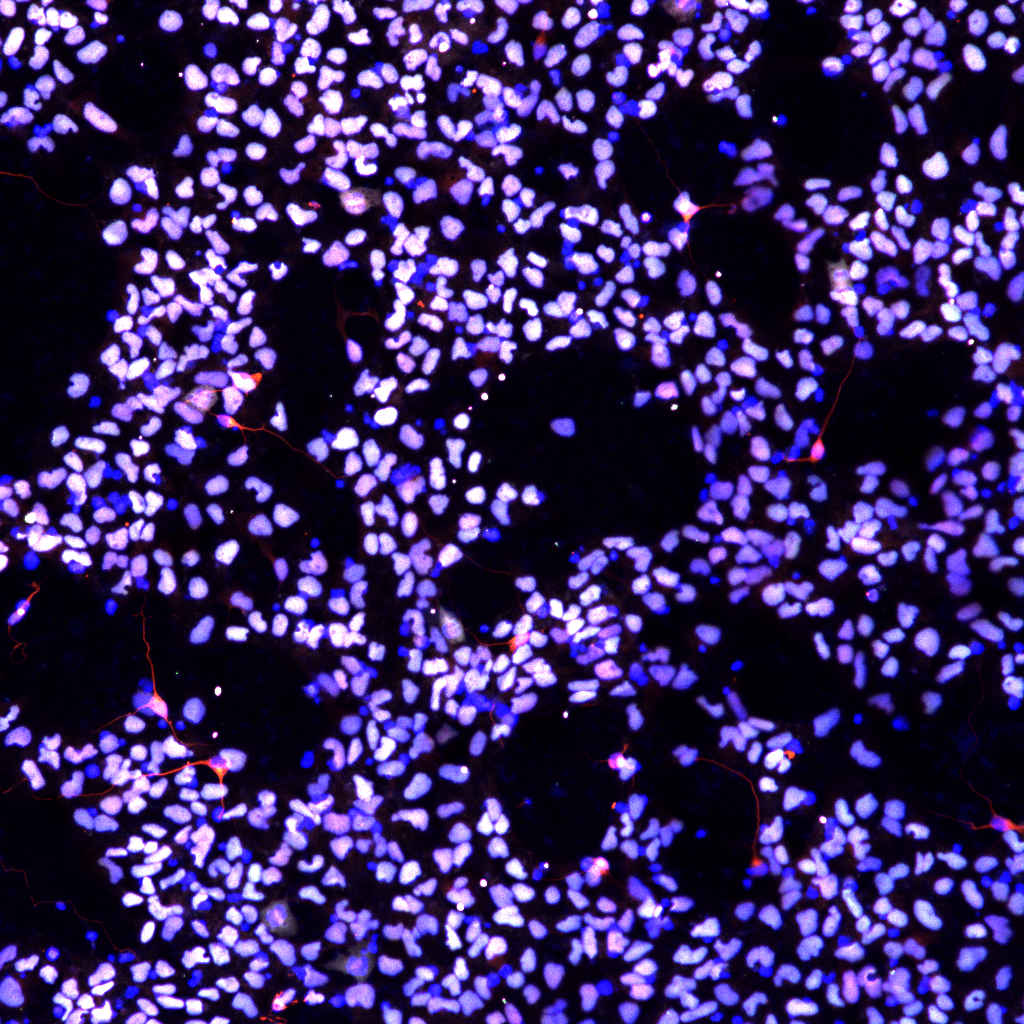

Supplement: Supplementary file 12 — Figure EV4 Source Data [file 44321_2025_302_MOESM12_ESM.zip › Figure EV4/EV4B/#7-5-merge.tif]

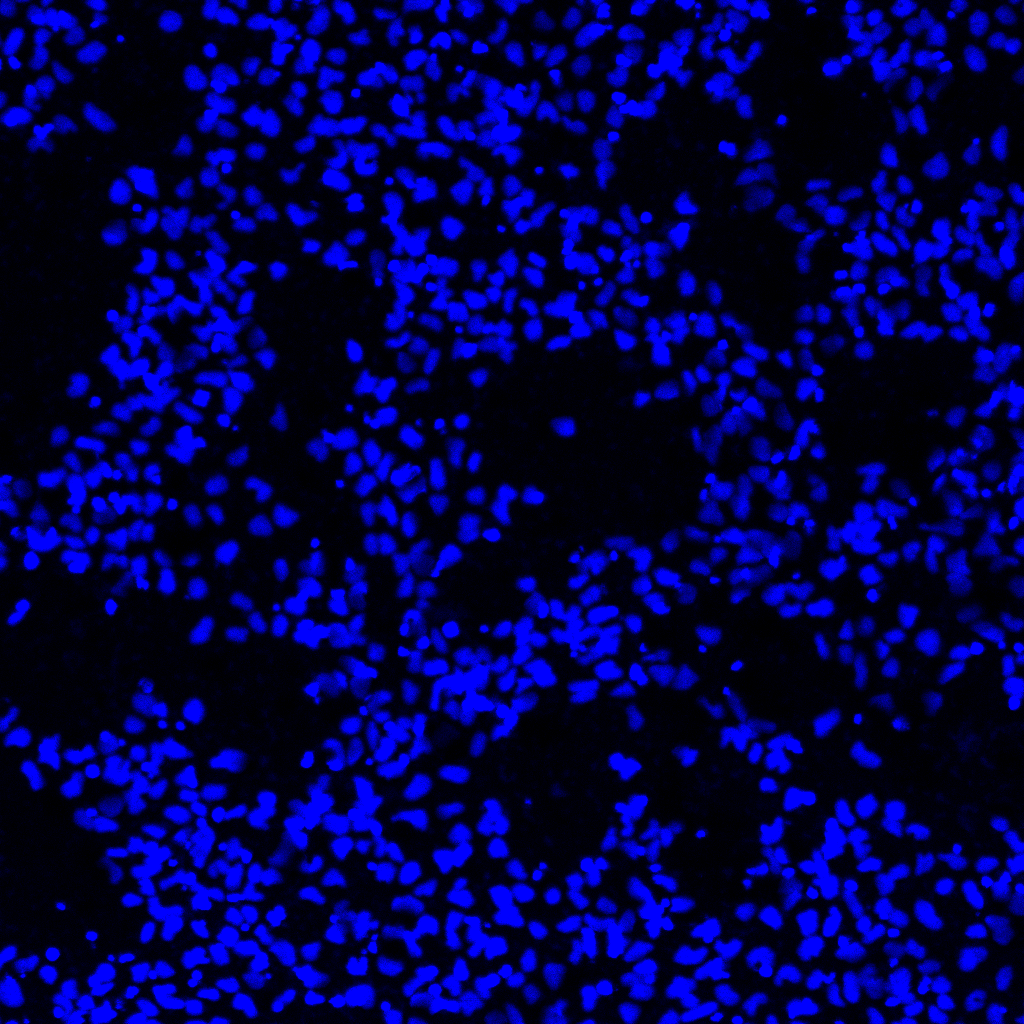

Supplement: Supplementary file 12 — Figure EV4 Source Data [file 44321_2025_302_MOESM12_ESM.zip › Figure EV4/EV4B/#7-5-DAPI.tif]

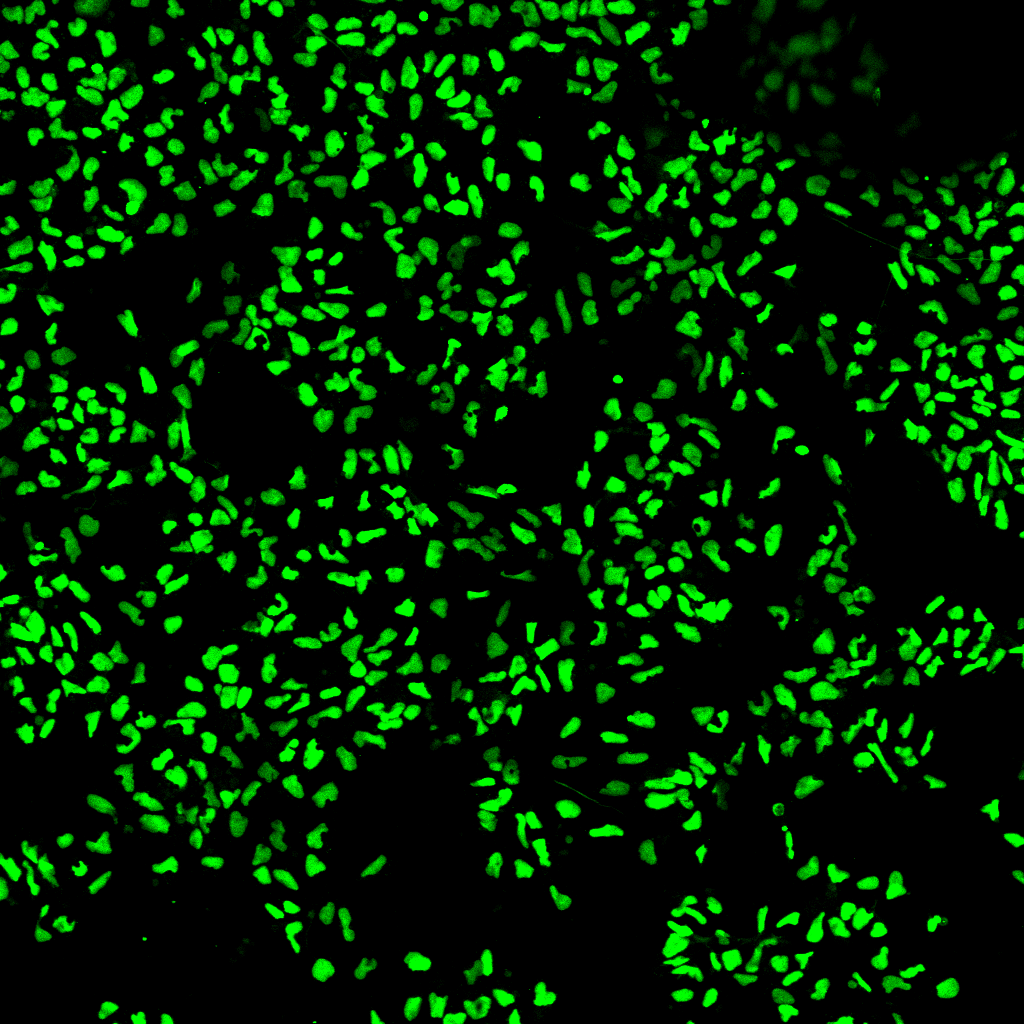

Supplement: Supplementary file 12 — Figure EV4 Source Data [file 44321_2025_302_MOESM12_ESM.zip › Figure EV4/EV4B/#12-3-SOX1.tif]

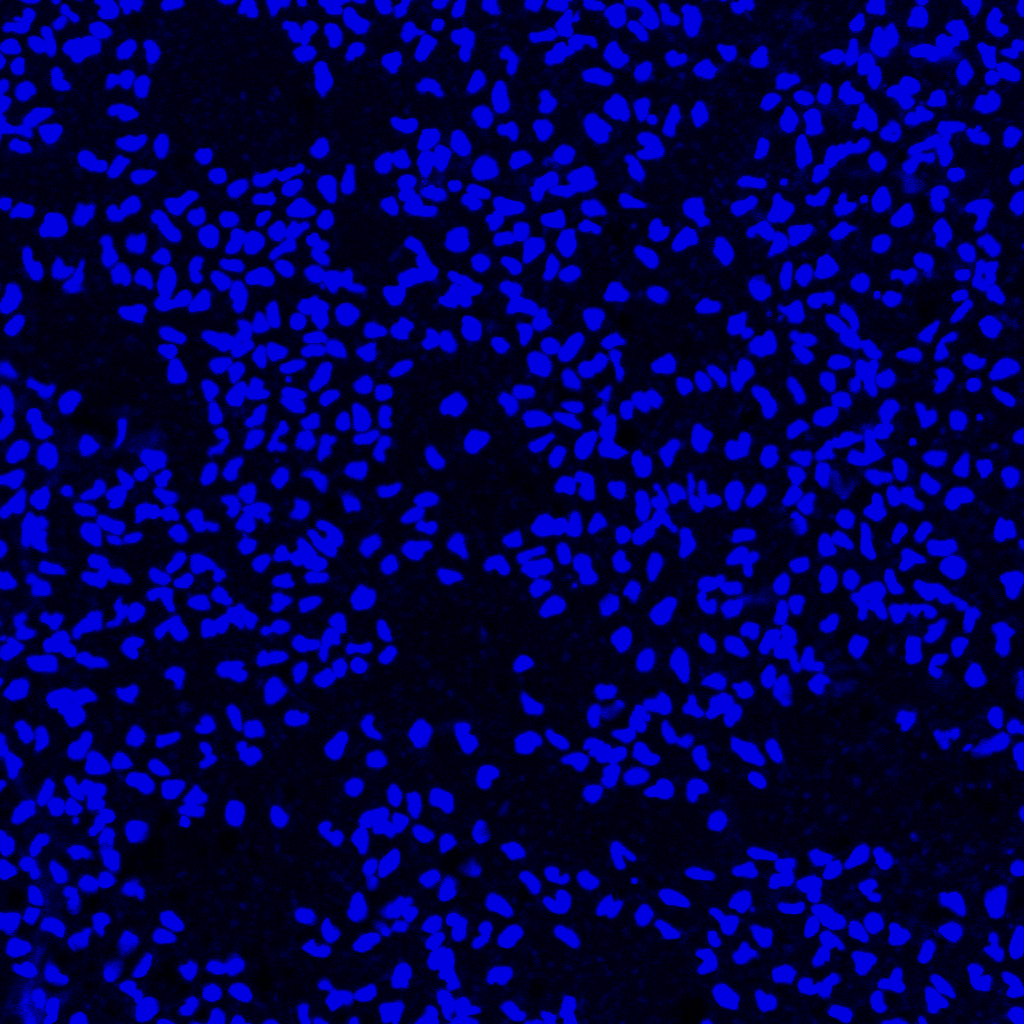

Supplement: Supplementary file 12 — Figure EV4 Source Data [file 44321_2025_302_MOESM12_ESM.zip › Figure EV4/EV4B/H9-DAPI.tif]

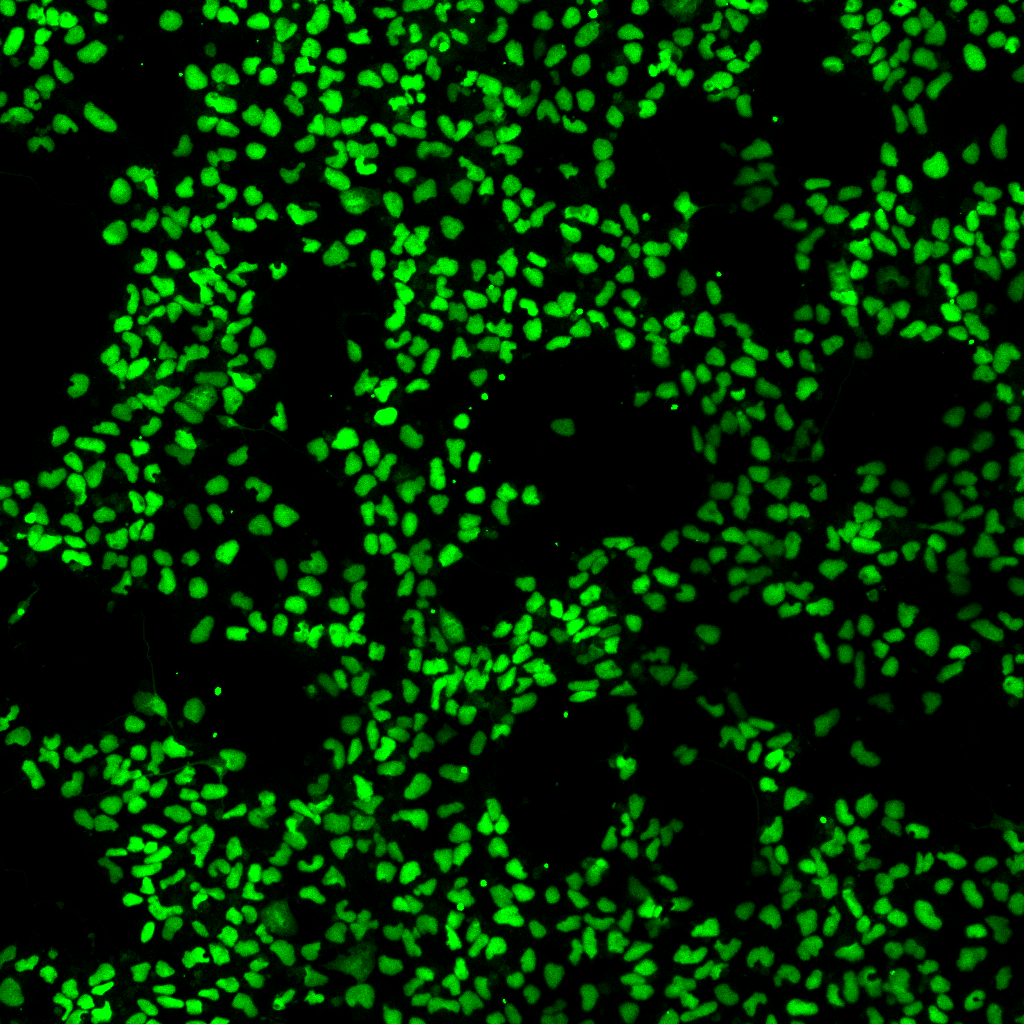

Supplement: Supplementary file 12 — Figure EV4 Source Data [file 44321_2025_302_MOESM12_ESM.zip › Figure EV4/EV4B/#7-5-SOX1.tif]

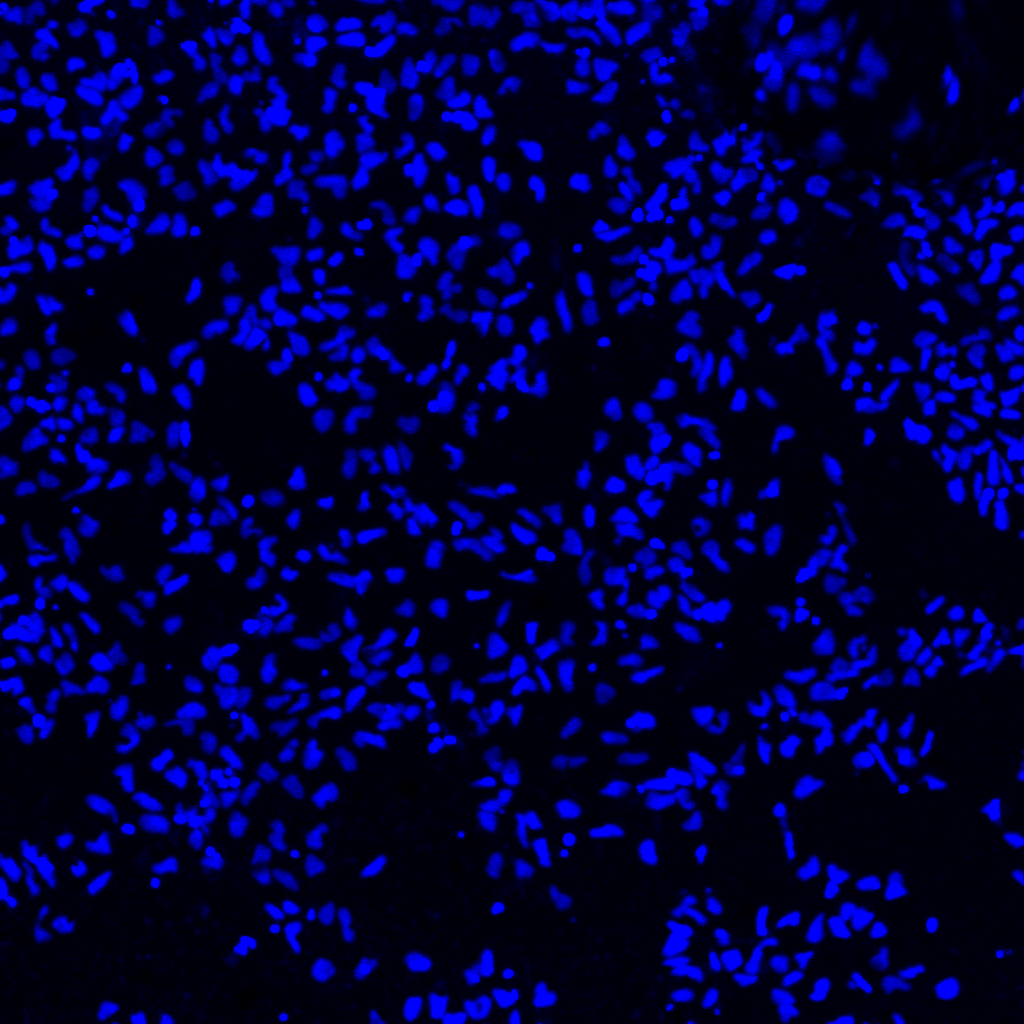

Supplement: Supplementary file 12 — Figure EV4 Source Data [file 44321_2025_302_MOESM12_ESM.zip › Figure EV4/EV4B/#12-3-DAPI.tif]

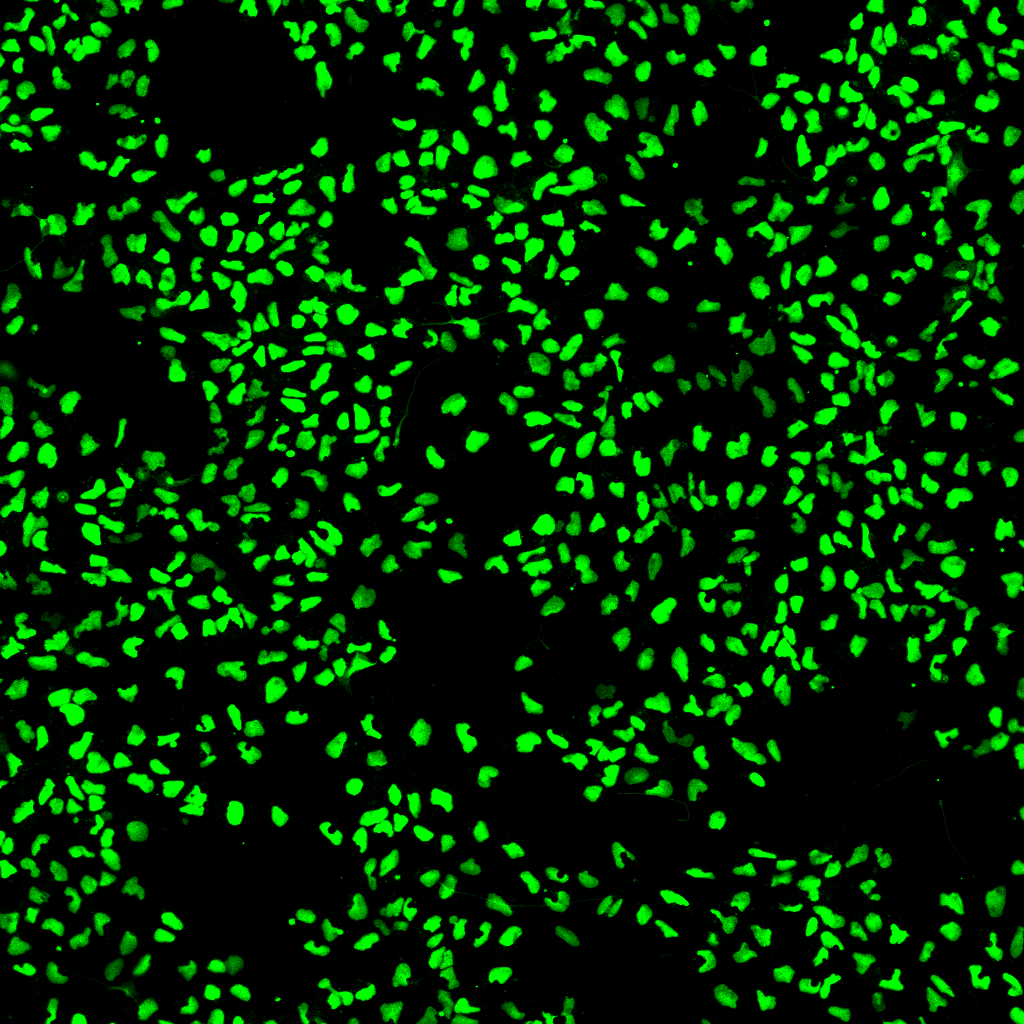

Supplement: Supplementary file 12 — Figure EV4 Source Data [file 44321_2025_302_MOESM12_ESM.zip › Figure EV4/EV4B/H9-SOX1.tif]

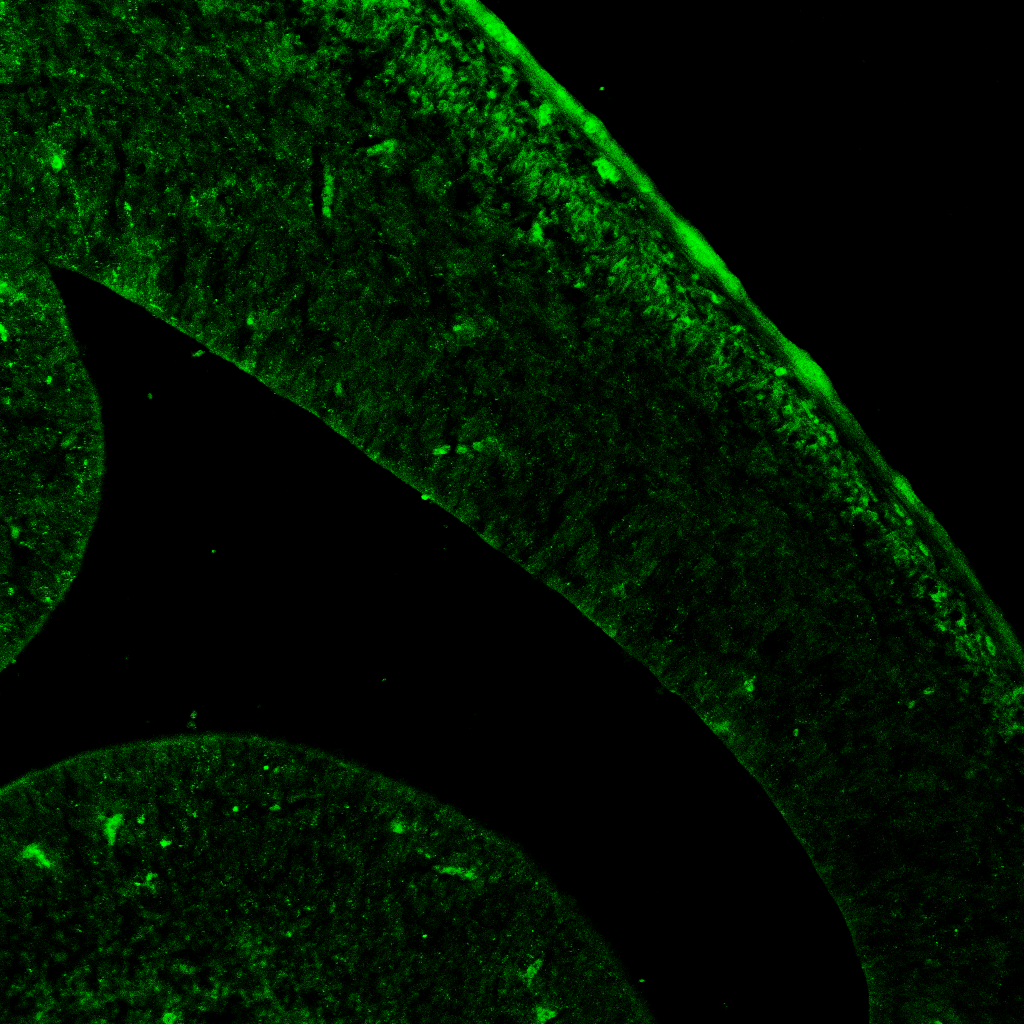

Supplement: Supplementary file 13 — Figure EV6 Source Data [file 44321_2025_302_MOESM13_ESM.zip › Figure EV6/EV6C/cKO_MAP2.tif]

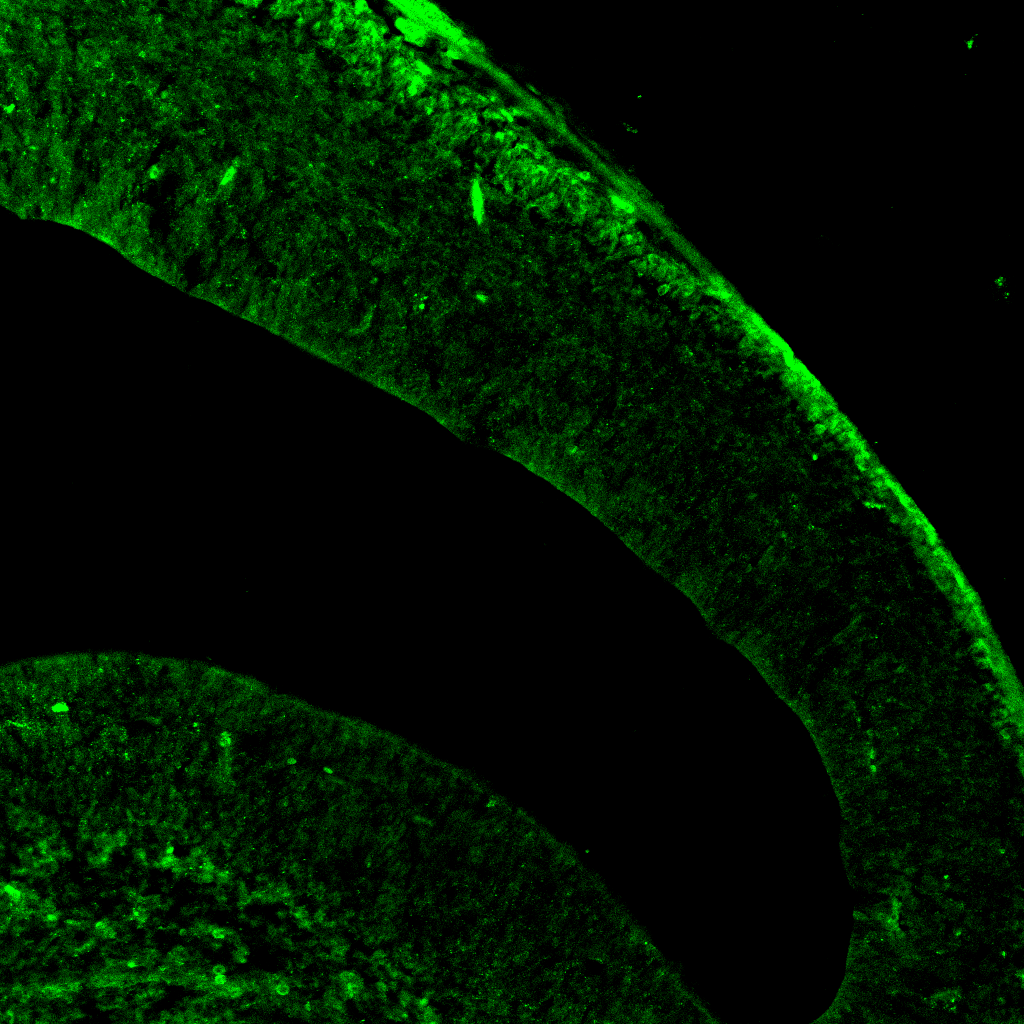

Supplement: Supplementary file 13 — Figure EV6 Source Data [file 44321_2025_302_MOESM13_ESM.zip › Figure EV6/EV6C/control_MAP2.tif]

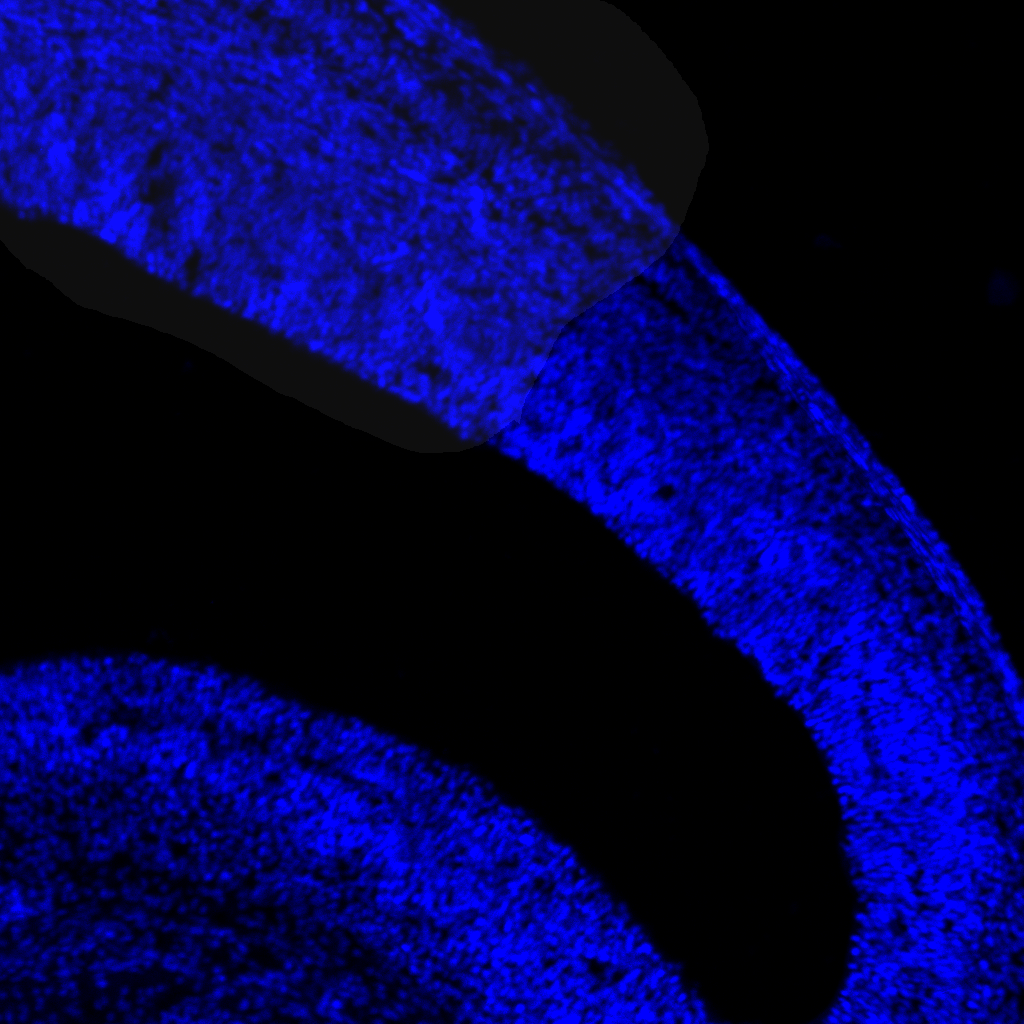

Supplement: Supplementary file 13 — Figure EV6 Source Data [file 44321_2025_302_MOESM13_ESM.zip › Figure EV6/EV6C/control_DAPI.tif]

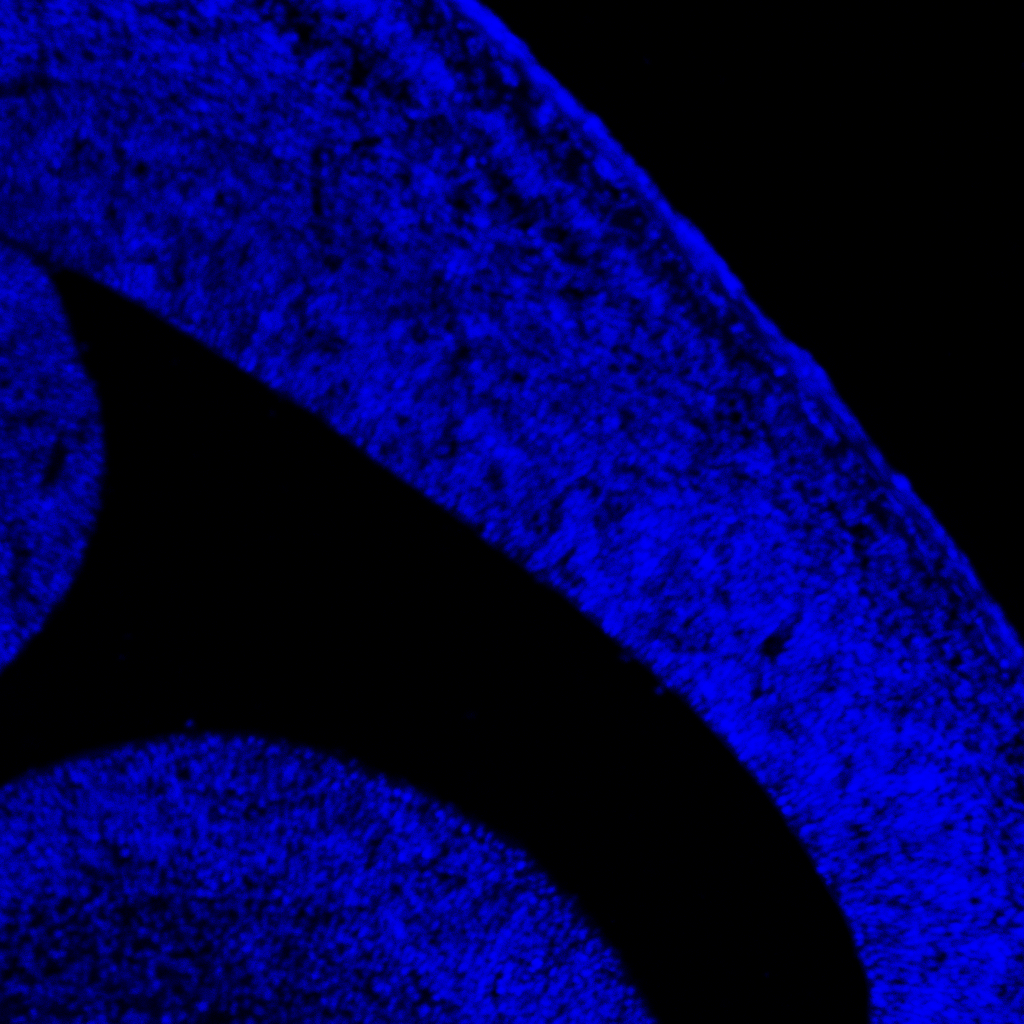

Supplement: Supplementary file 13 — Figure EV6 Source Data [file 44321_2025_302_MOESM13_ESM.zip › Figure EV6/EV6C/cKO_DAPI.tif]

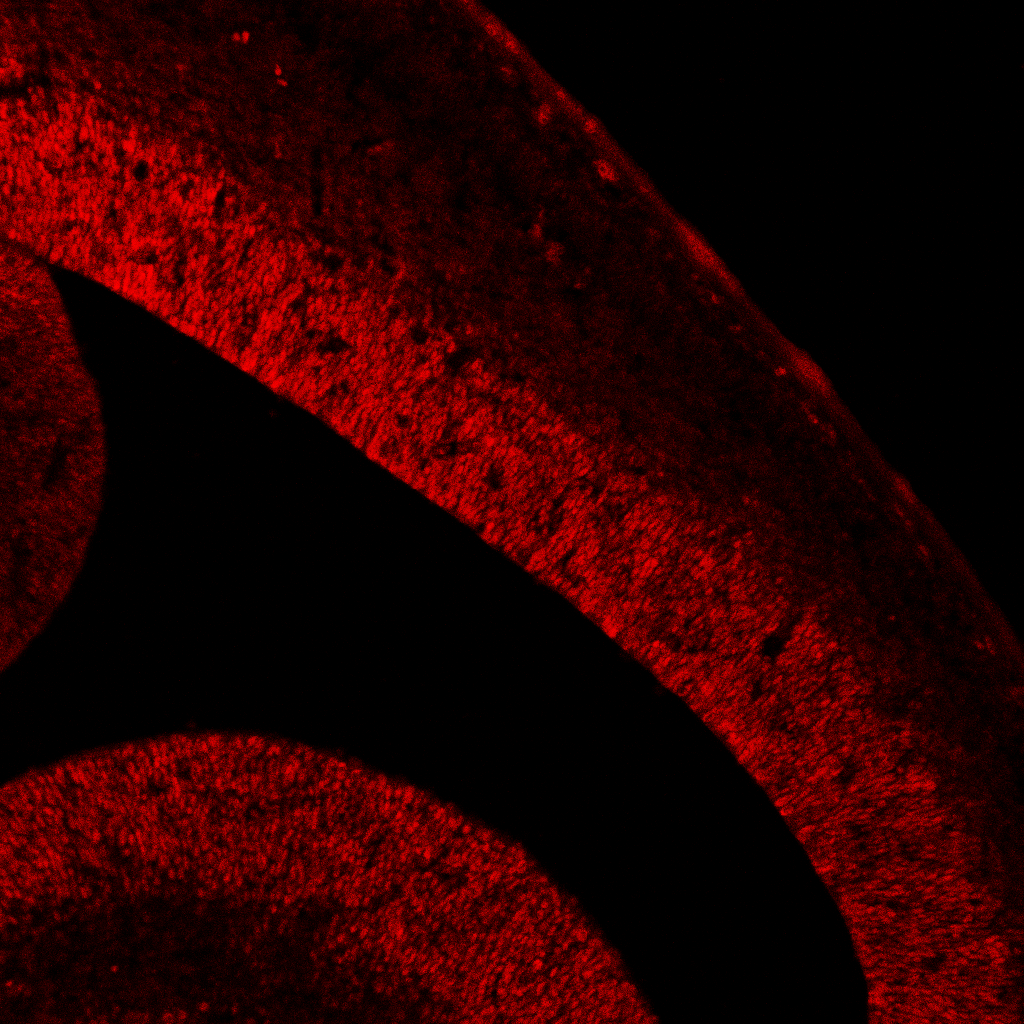

Supplement: Supplementary file 13 — Figure EV6 Source Data [file 44321_2025_302_MOESM13_ESM.zip › Figure EV6/EV6C/cKO_PAX6.tif]

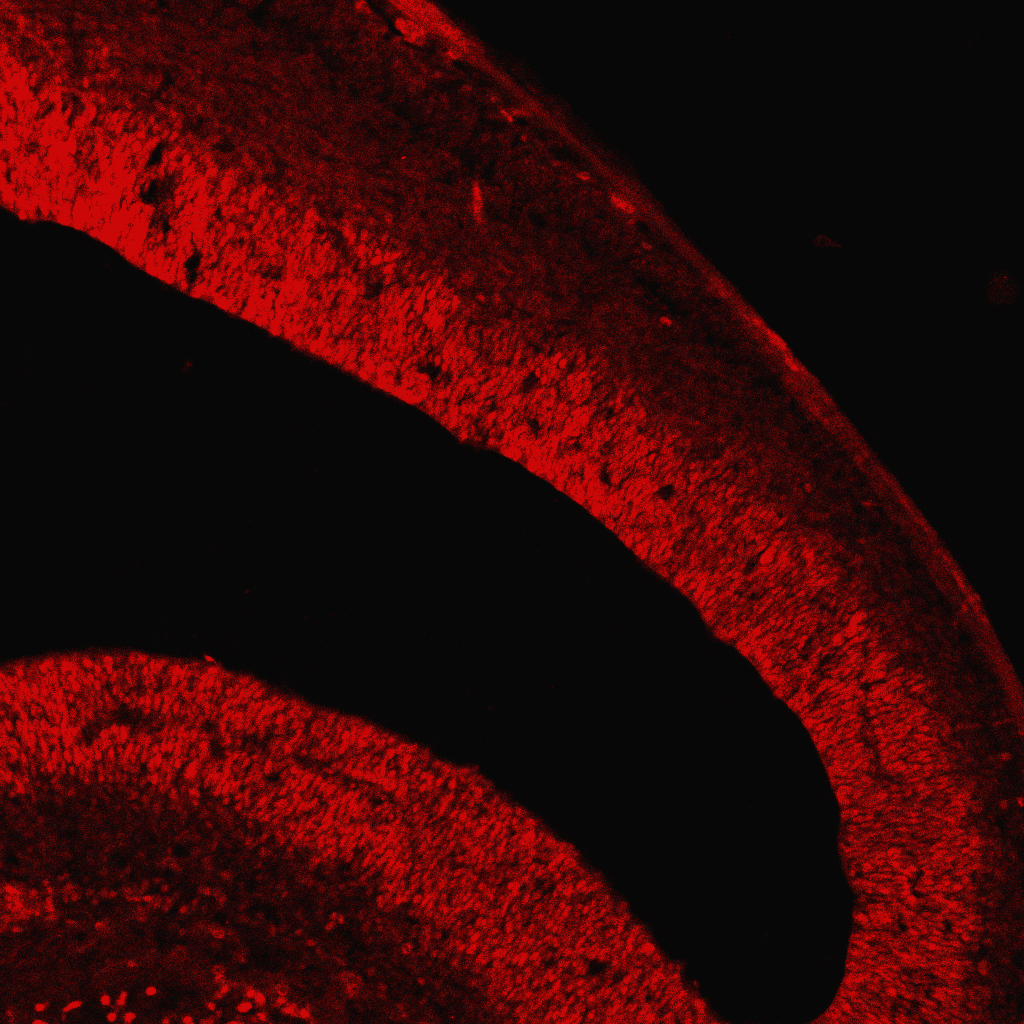

Supplement: Supplementary file 13 — Figure EV6 Source Data [file 44321_2025_302_MOESM13_ESM.zip › Figure EV6/EV6C/control_PAX6.tif]

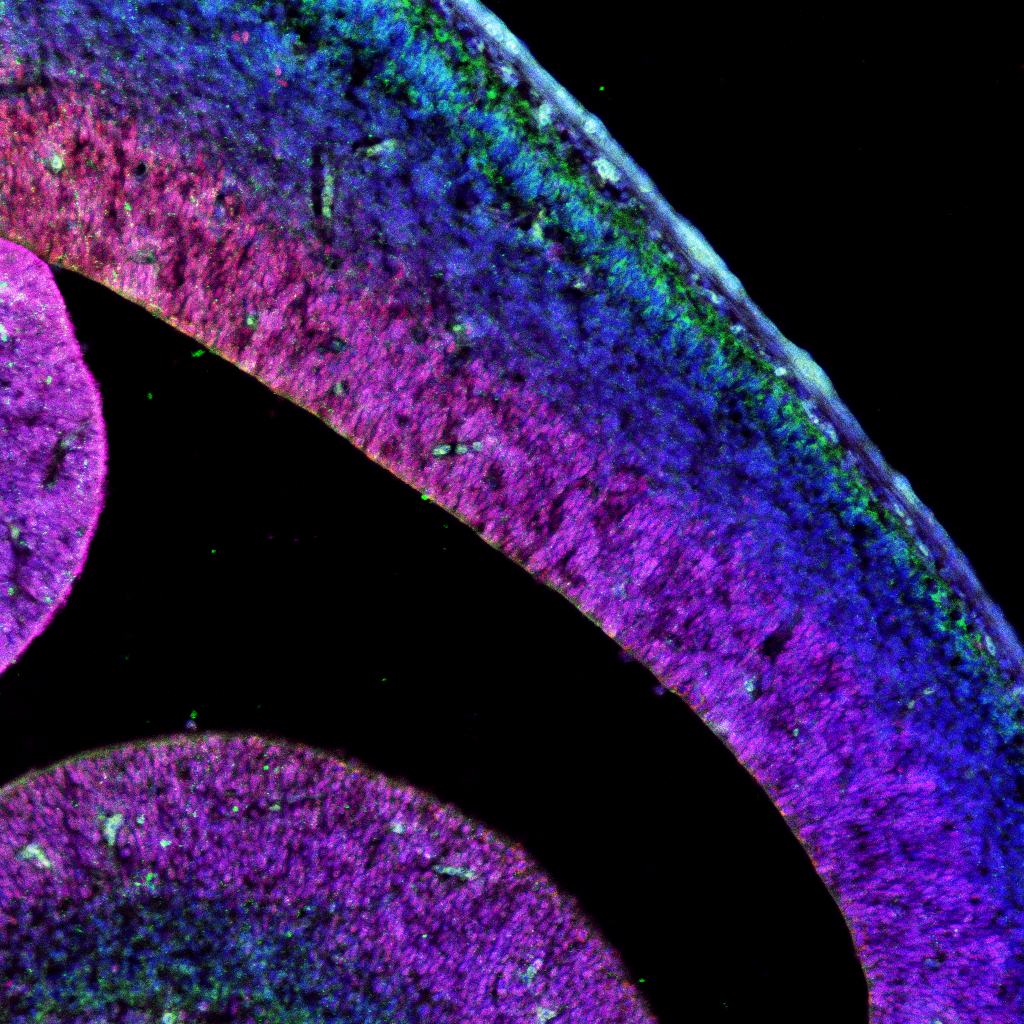

Supplement: Supplementary file 13 — Figure EV6 Source Data [file 44321_2025_302_MOESM13_ESM.zip › Figure EV6/EV6C/cKO_merge.tif]

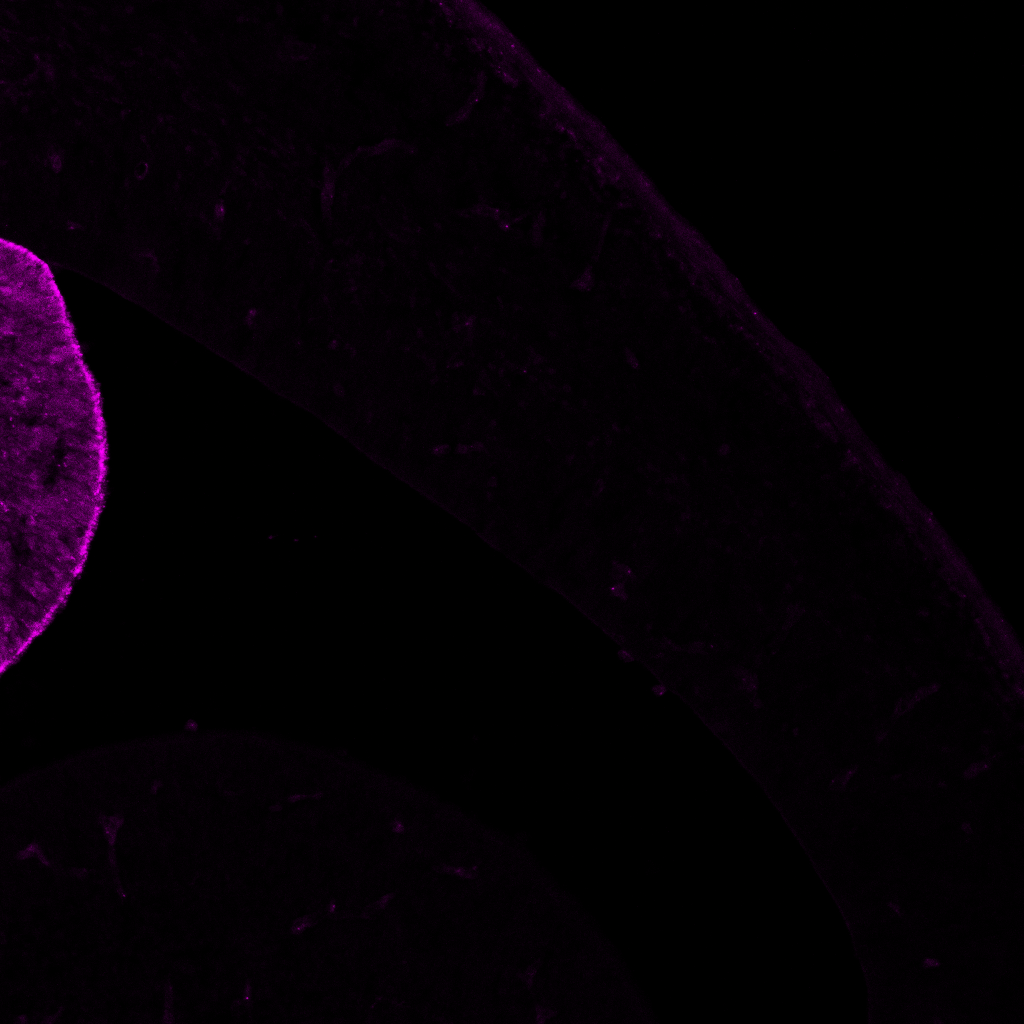

Supplement: Supplementary file 13 — Figure EV6 Source Data [file 44321_2025_302_MOESM13_ESM.zip › Figure EV6/EV6C/cKO_CETN3.tif]

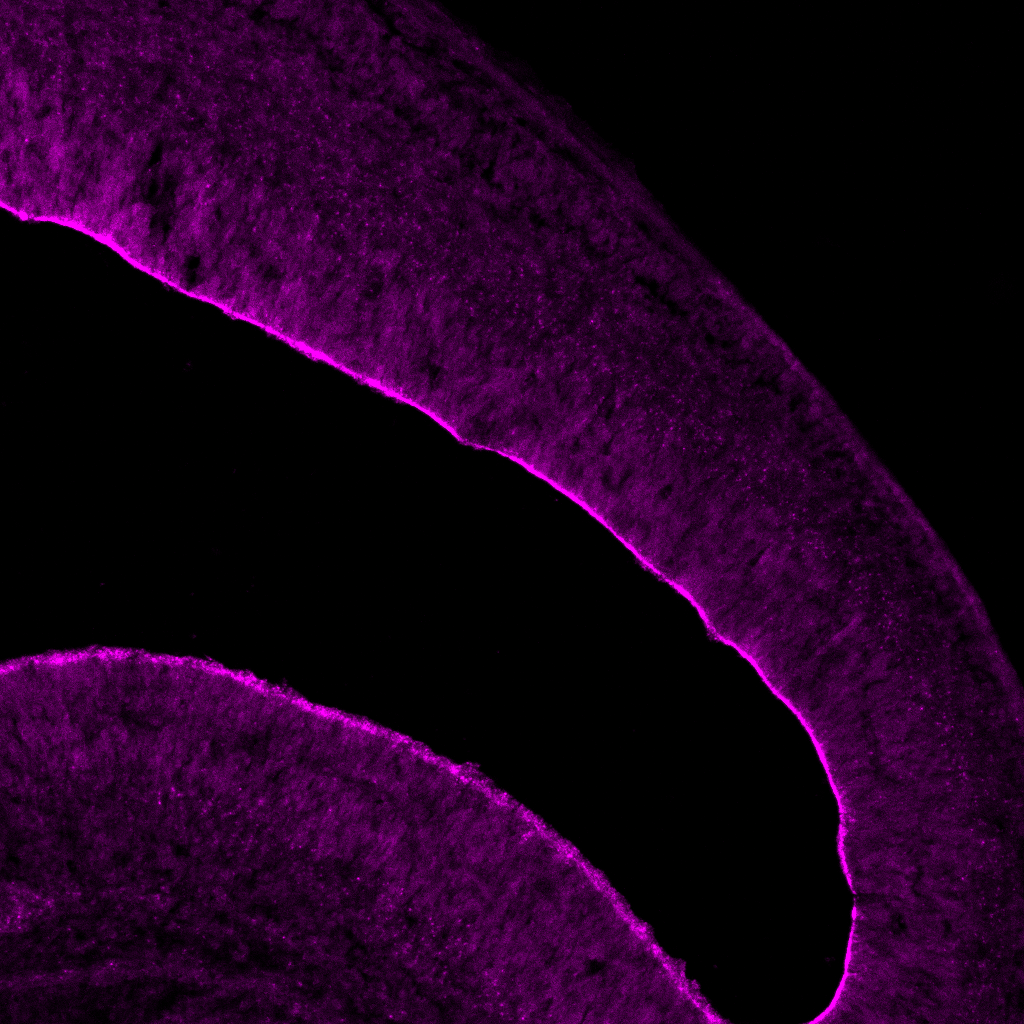

Supplement: Supplementary file 13 — Figure EV6 Source Data [file 44321_2025_302_MOESM13_ESM.zip › Figure EV6/EV6C/control_CETN3.tif]

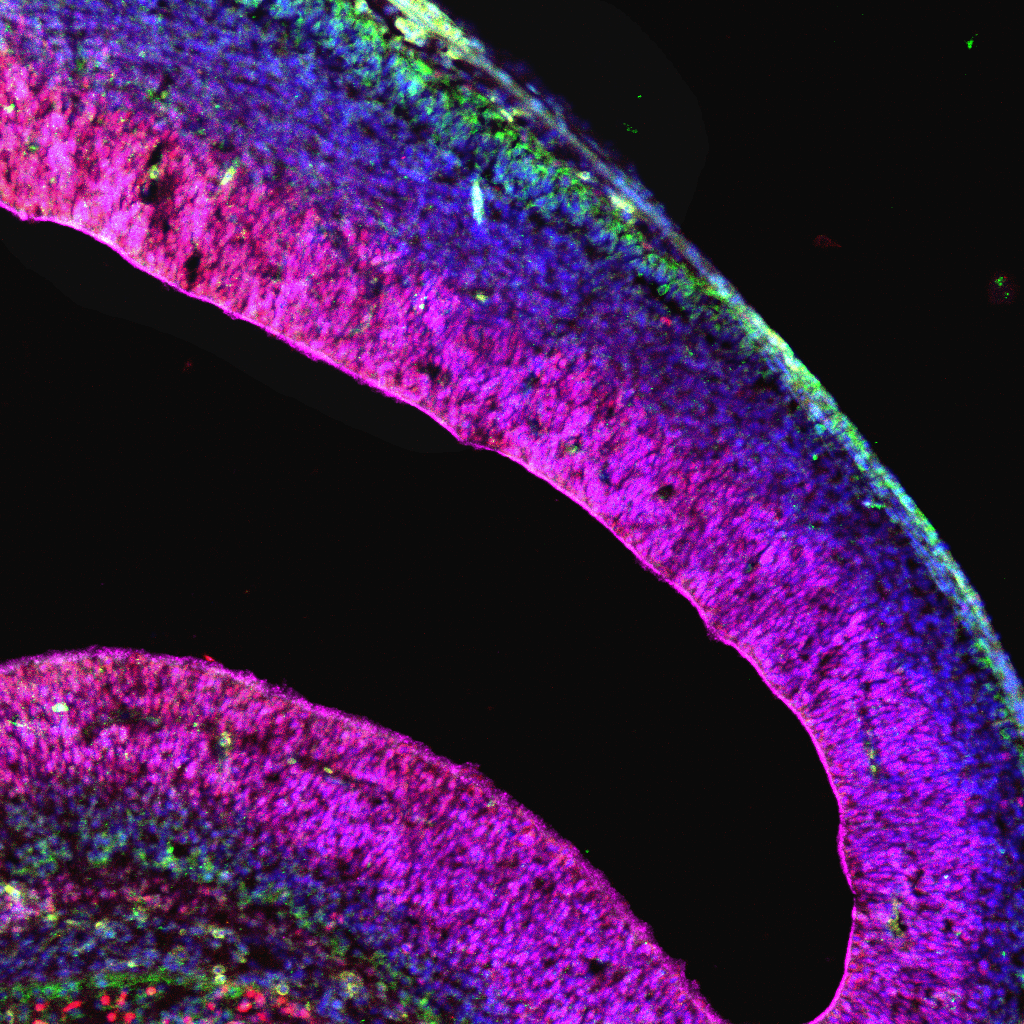

Supplement: Supplementary file 13 — Figure EV6 Source Data [file 44321_2025_302_MOESM13_ESM.zip › Figure EV6/EV6C/control_merge.tif]

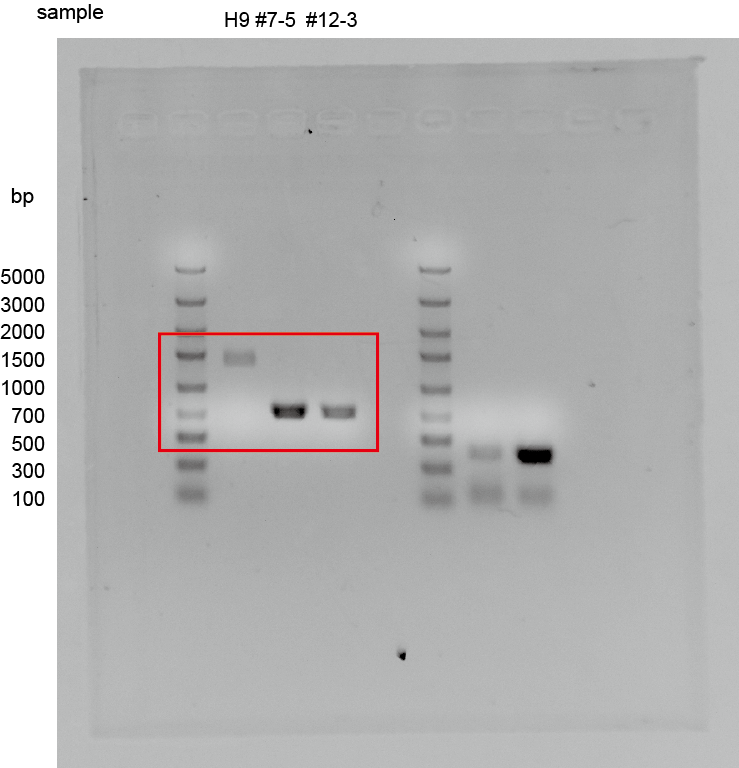

Supplement: Supplementary file 14 — Appendix Figure Source Data [file 44321_2025_302_MOESM14_ESM.zip › Appendix Figure S2/S2B/PCR.png]

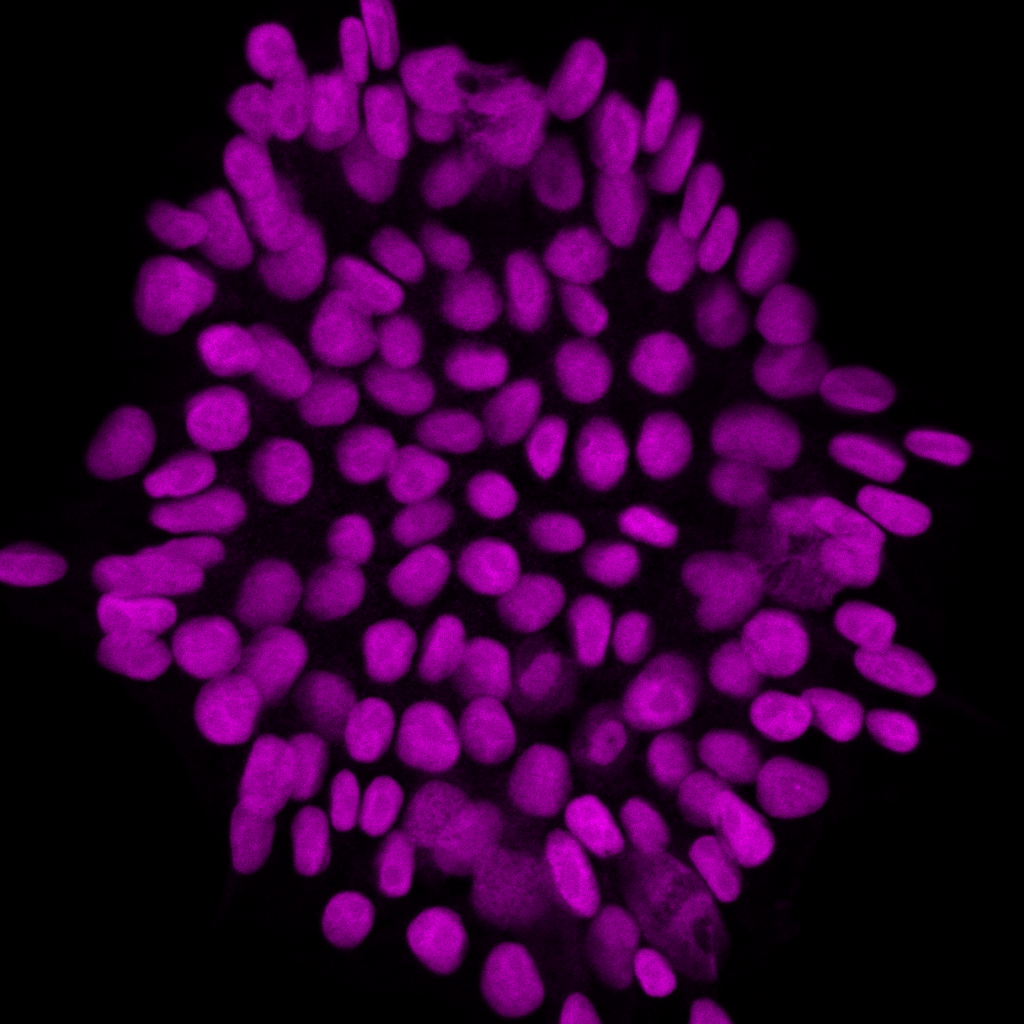

Supplement: Supplementary file 14 — Appendix Figure Source Data [file 44321_2025_302_MOESM14_ESM.zip › Appendix Figure S2/S2C/IF-#12-3-SOX2.tif]

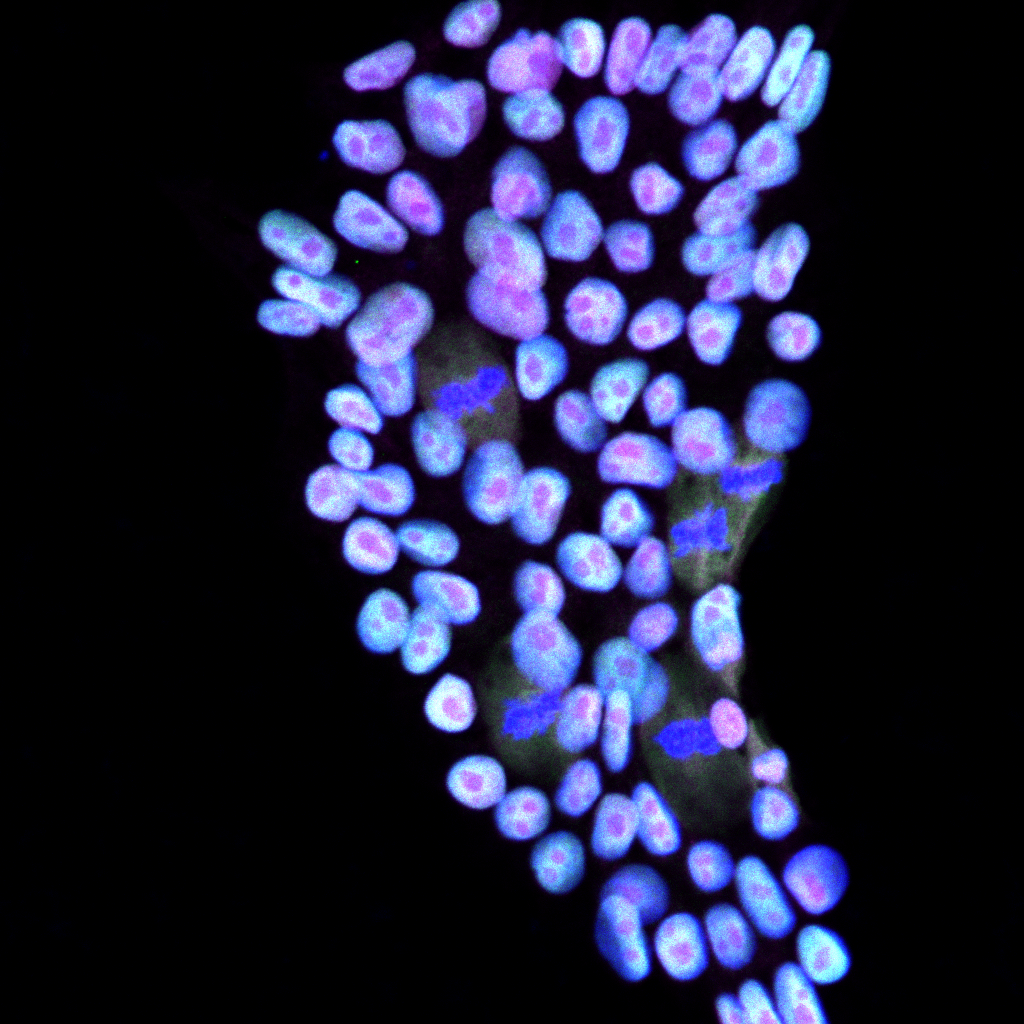

Supplement: Supplementary file 14 — Appendix Figure Source Data [file 44321_2025_302_MOESM14_ESM.zip › Appendix Figure S2/S2C/IF-H9-merge.tif]

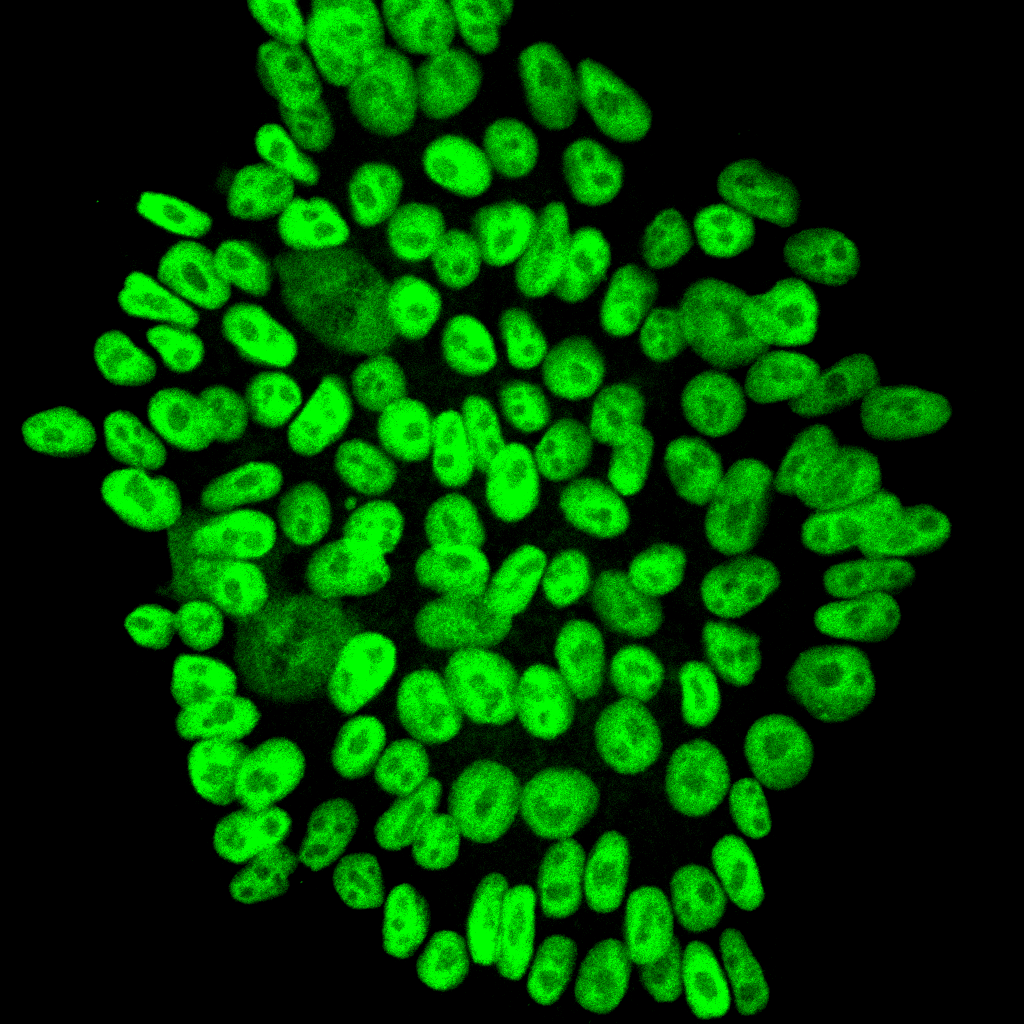

Supplement: Supplementary file 14 — Appendix Figure Source Data [file 44321_2025_302_MOESM14_ESM.zip › Appendix Figure S2/S2C/IF-#7-5-OCT4.tif]

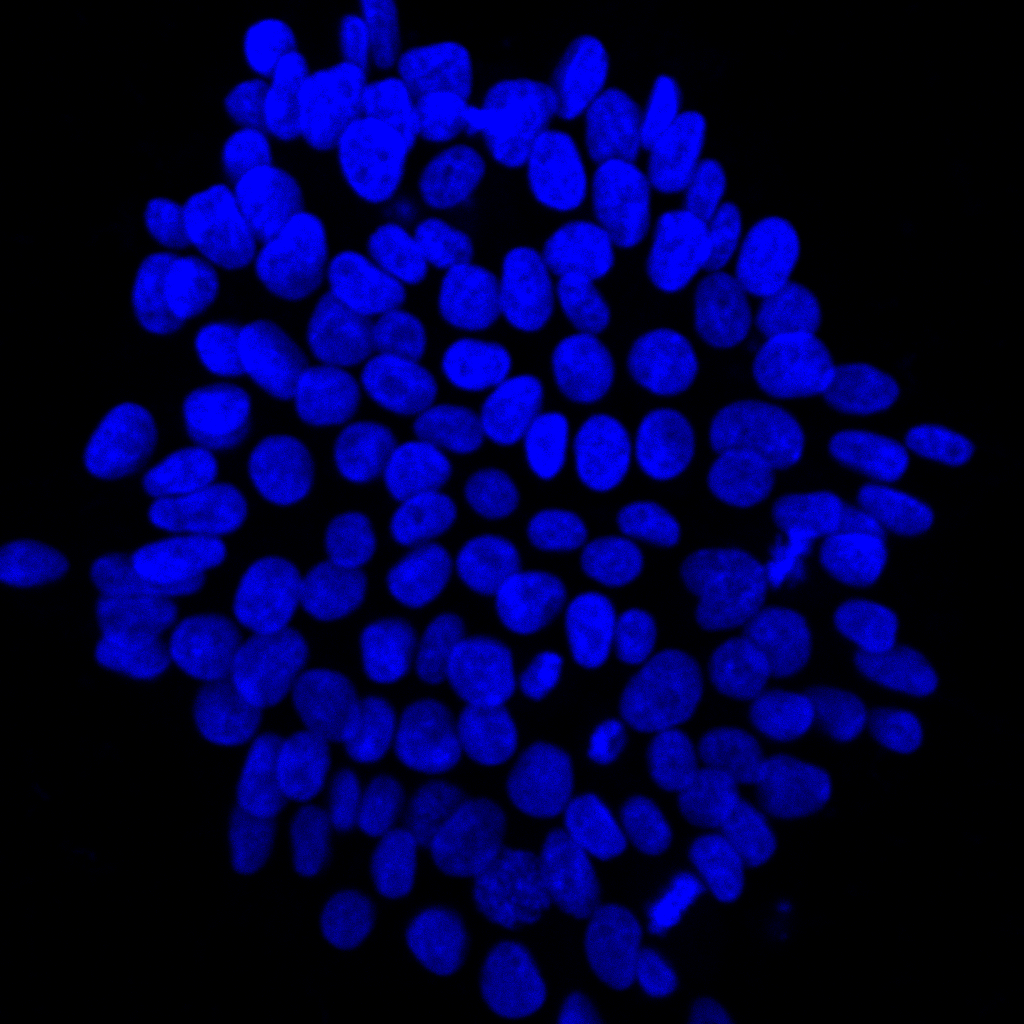

Supplement: Supplementary file 14 — Appendix Figure Source Data [file 44321_2025_302_MOESM14_ESM.zip › Appendix Figure S2/S2C/IF-#12-3-DAPI.tif]

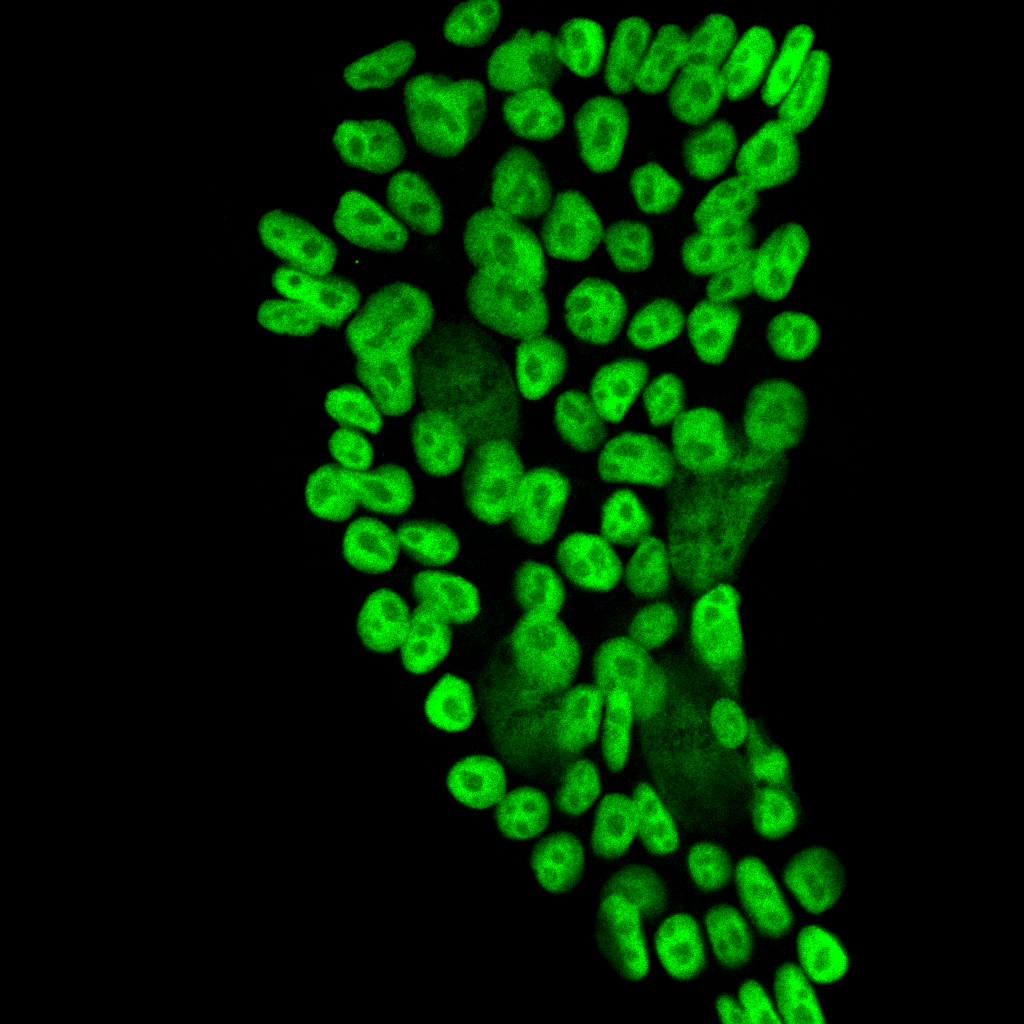

Supplement: Supplementary file 14 — Appendix Figure Source Data [file 44321_2025_302_MOESM14_ESM.zip › Appendix Figure S2/S2C/IF-H9-OCT4.tif]

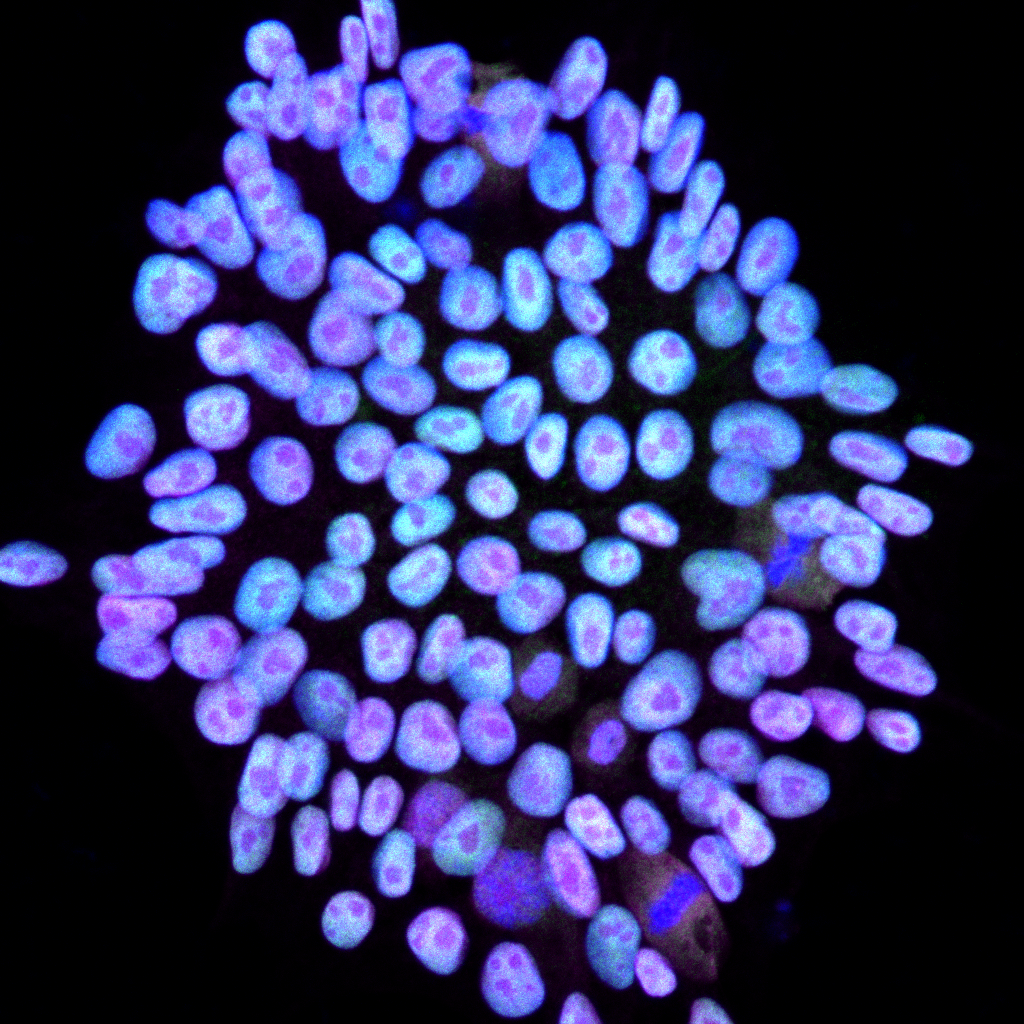

Supplement: Supplementary file 14 — Appendix Figure Source Data [file 44321_2025_302_MOESM14_ESM.zip › Appendix Figure S2/S2C/IF-#12-3-merge.tif]

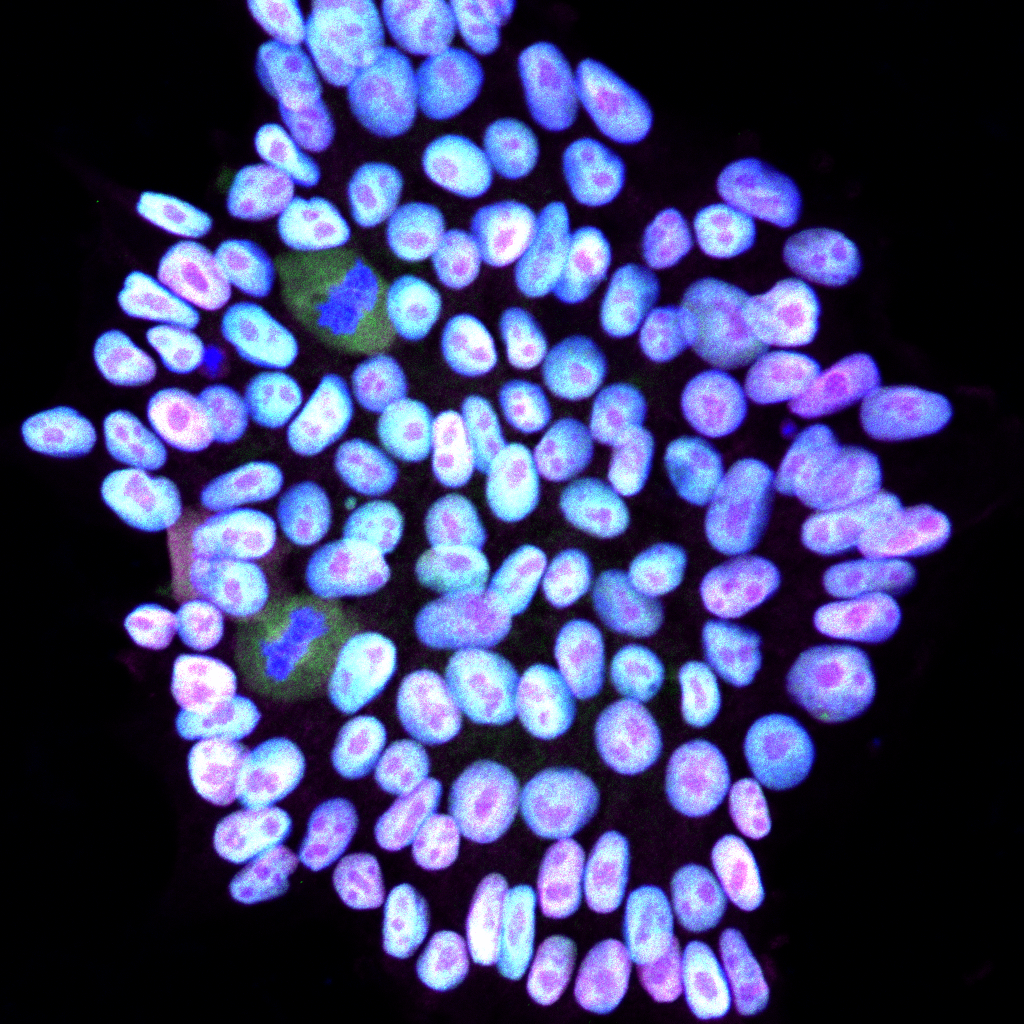

Supplement: Supplementary file 14 — Appendix Figure Source Data [file 44321_2025_302_MOESM14_ESM.zip › Appendix Figure S2/S2C/IF-#7-5-merge.tif]

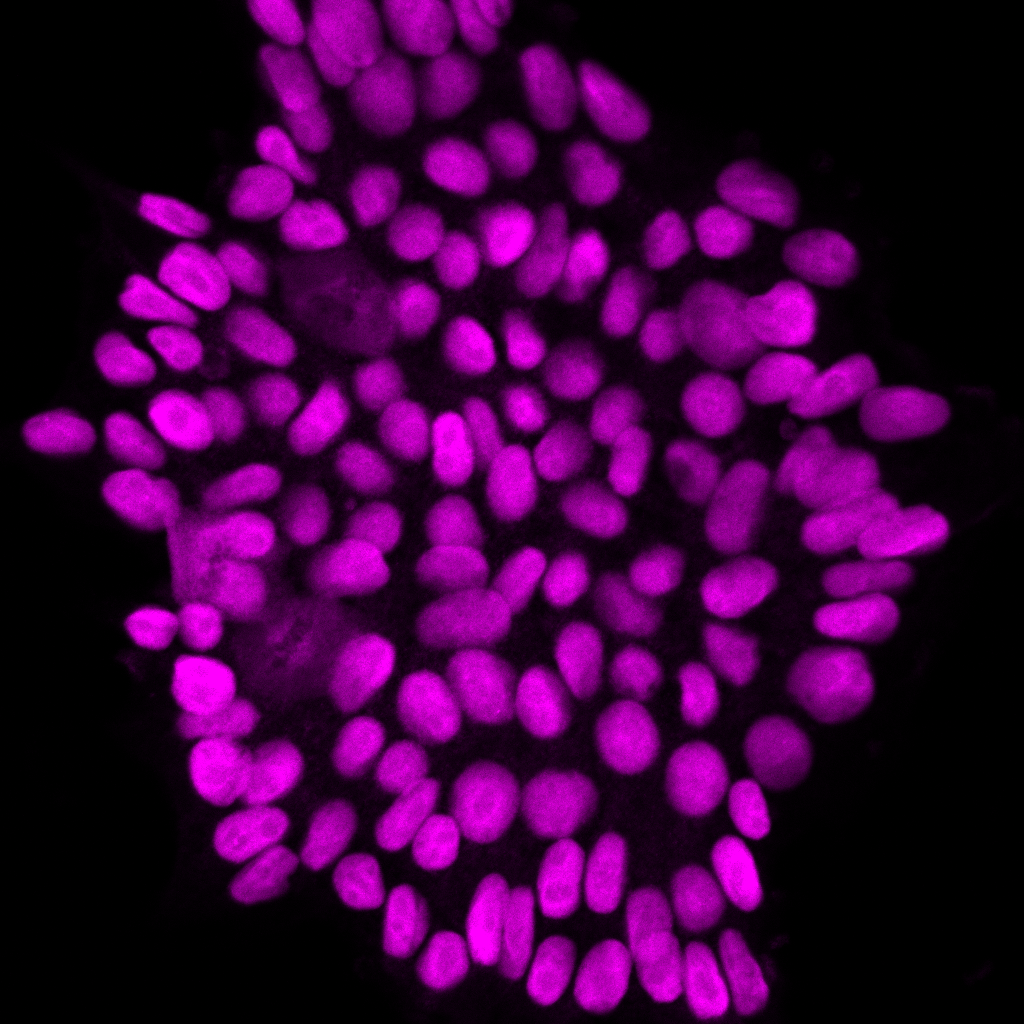

Supplement: Supplementary file 14 — Appendix Figure Source Data [file 44321_2025_302_MOESM14_ESM.zip › Appendix Figure S2/S2C/IF-#7-5-SOX2.tif]

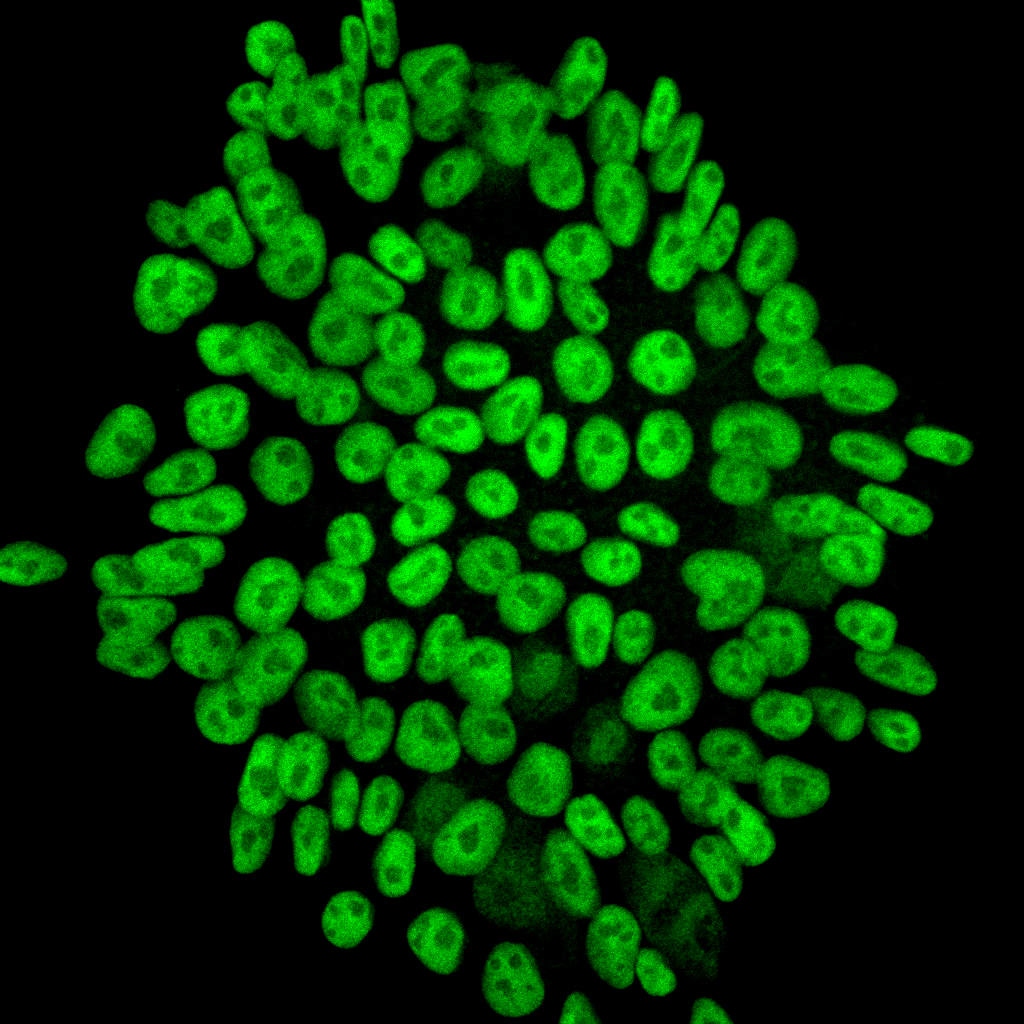

Supplement: Supplementary file 14 — Appendix Figure Source Data [file 44321_2025_302_MOESM14_ESM.zip › Appendix Figure S2/S2C/IF-#12-3-OCT4.tif]

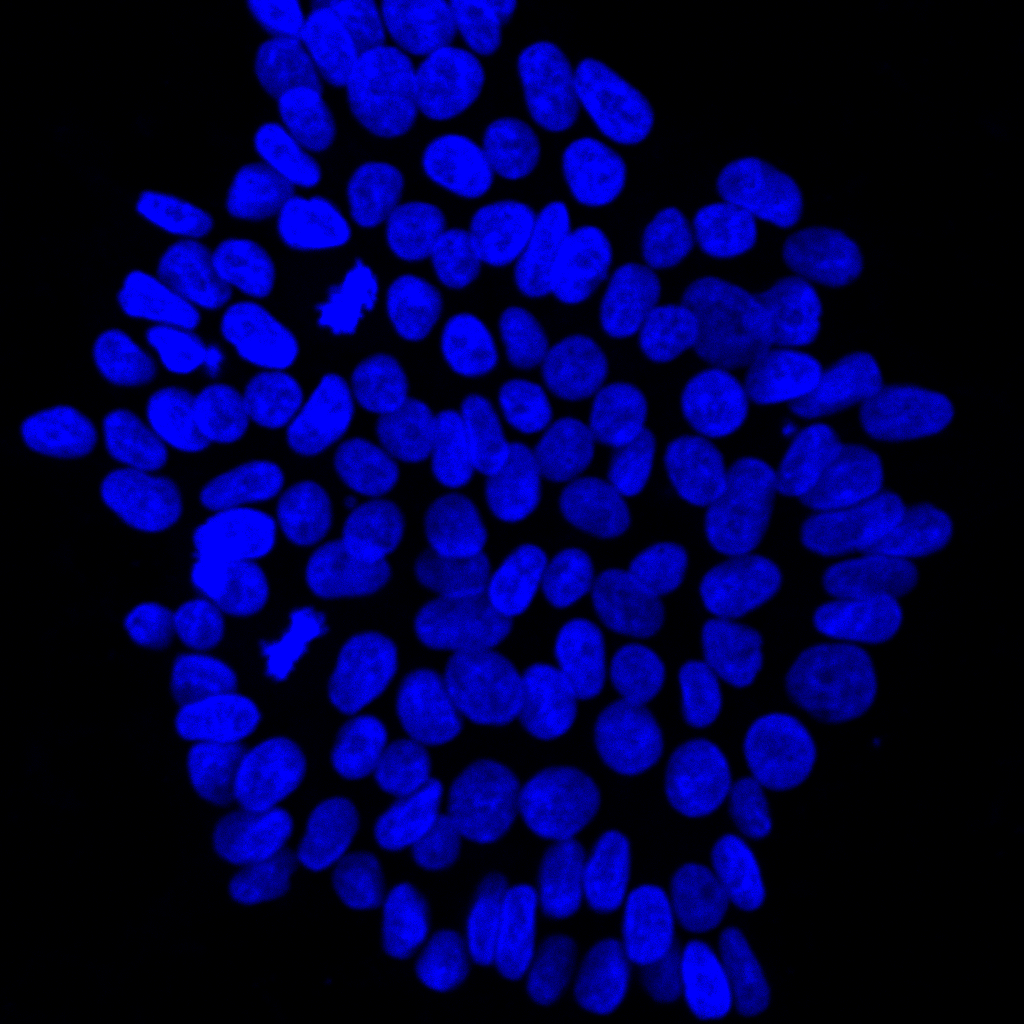

Supplement: Supplementary file 14 — Appendix Figure Source Data [file 44321_2025_302_MOESM14_ESM.zip › Appendix Figure S2/S2C/IF-#7-5-DAPI.tif]

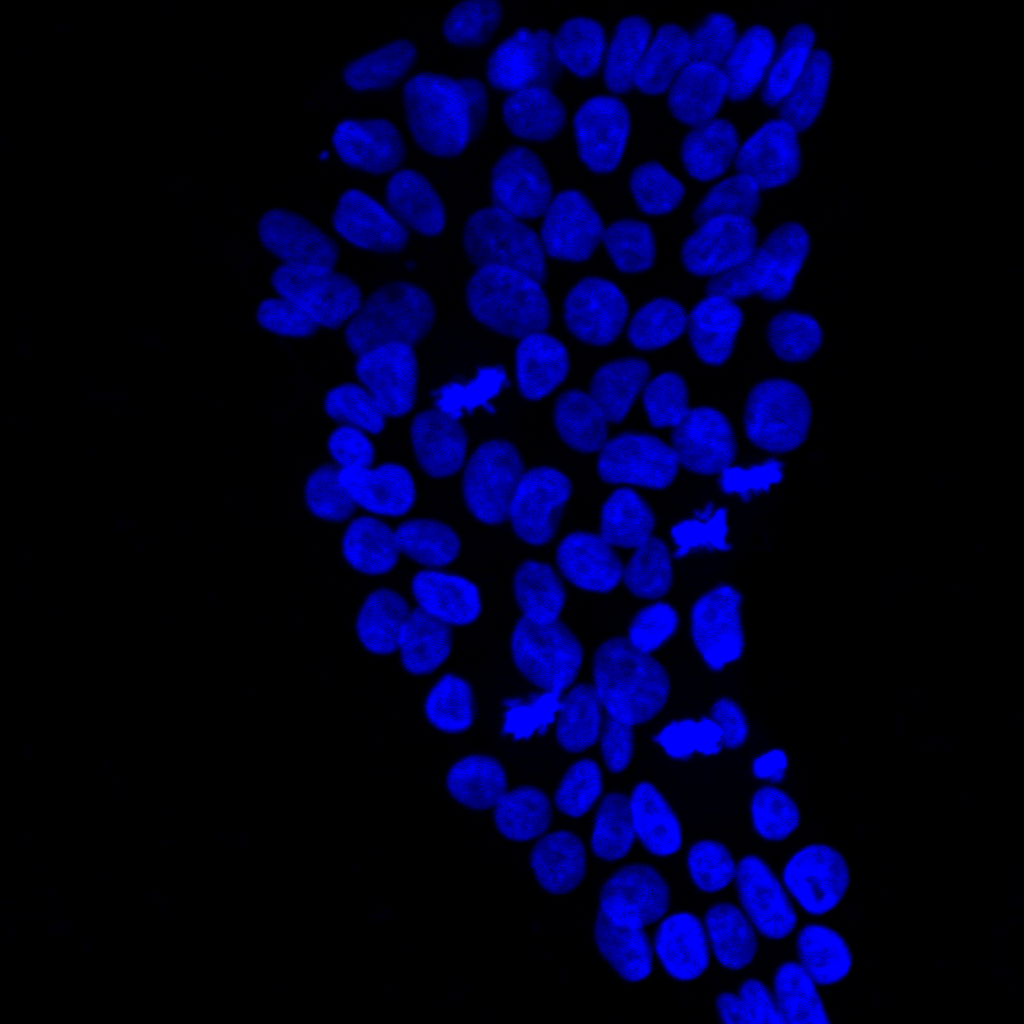

Supplement: Supplementary file 14 — Appendix Figure Source Data [file 44321_2025_302_MOESM14_ESM.zip › Appendix Figure S2/S2C/IF-H9-DAPI.tif]

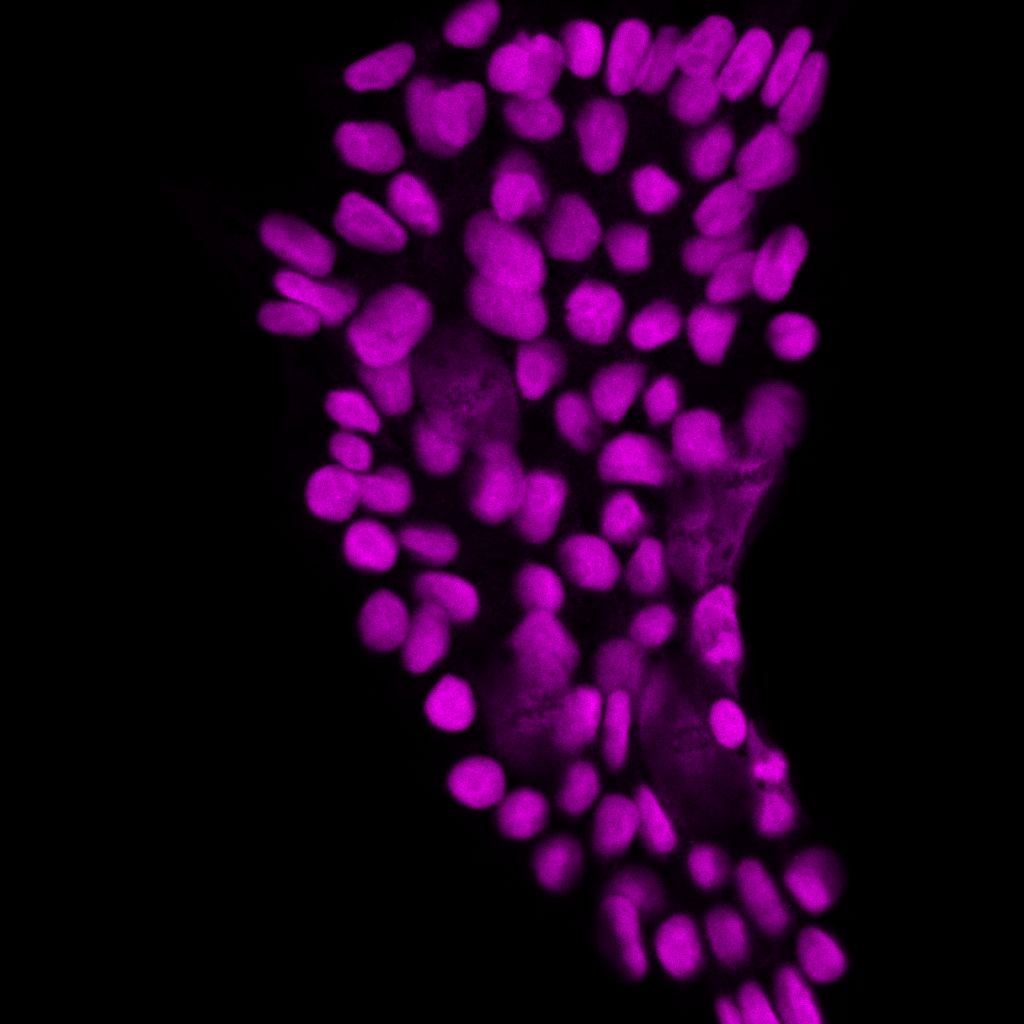

Supplement: Supplementary file 14 — Appendix Figure Source Data [file 44321_2025_302_MOESM14_ESM.zip › Appendix Figure S2/S2C/IF-H9-SOX2.tif]
