# Supplementary figures and images for: CETN3 deficiency induces microcephaly by disrupting neural stem/progenitor cell fate through impaired centrosome assembly and RNA splicing (part 5 of 5)
Source: EMBO Mol Med. 2025 Sep 8;17(10):2735–61. doi: 10.1038/s44321-025-00302-7 (PMC12514221; doi:10.1038/s44321-025-00302-7)

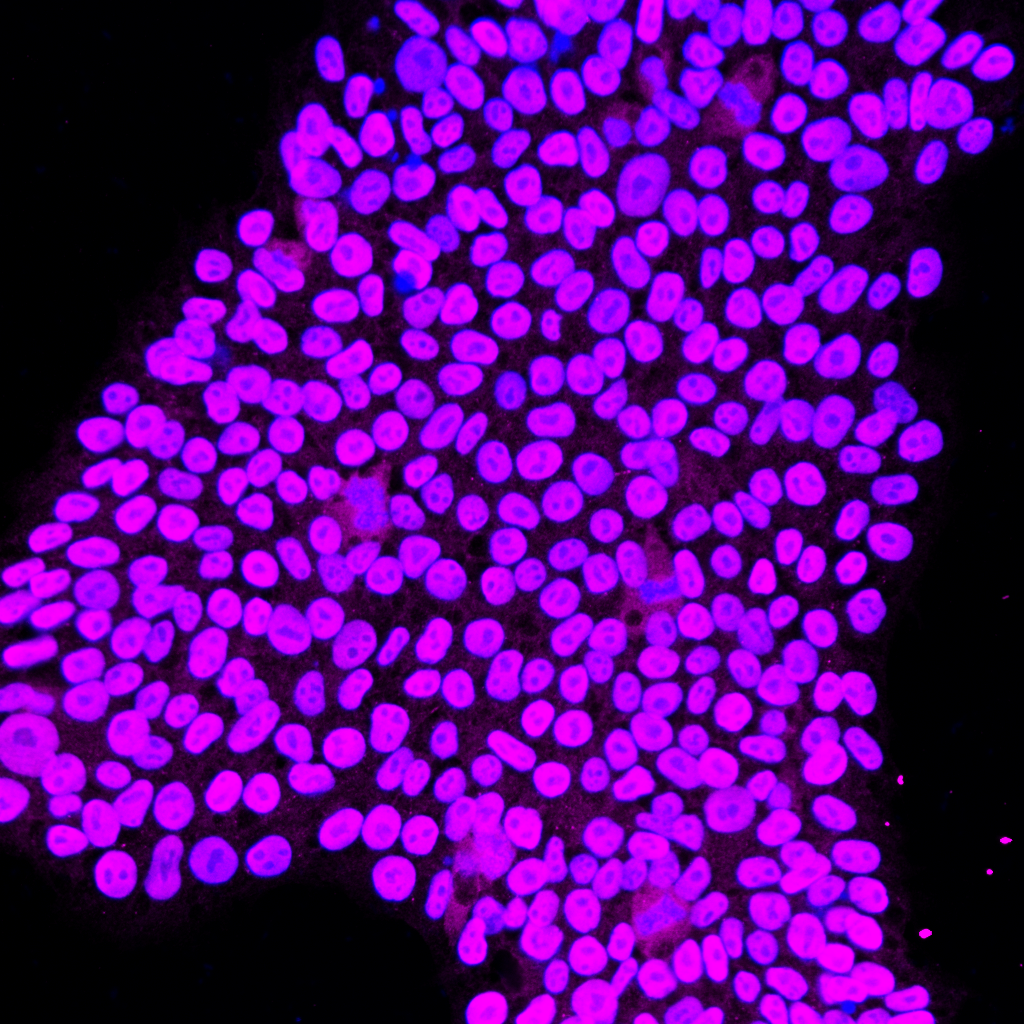

Supplement: Supplementary file 14 — Appendix Figure Source Data [file 44321_2025_302_MOESM14_ESM.zip › Appendix Figure S2/S2D/#12-3-merge.tif]

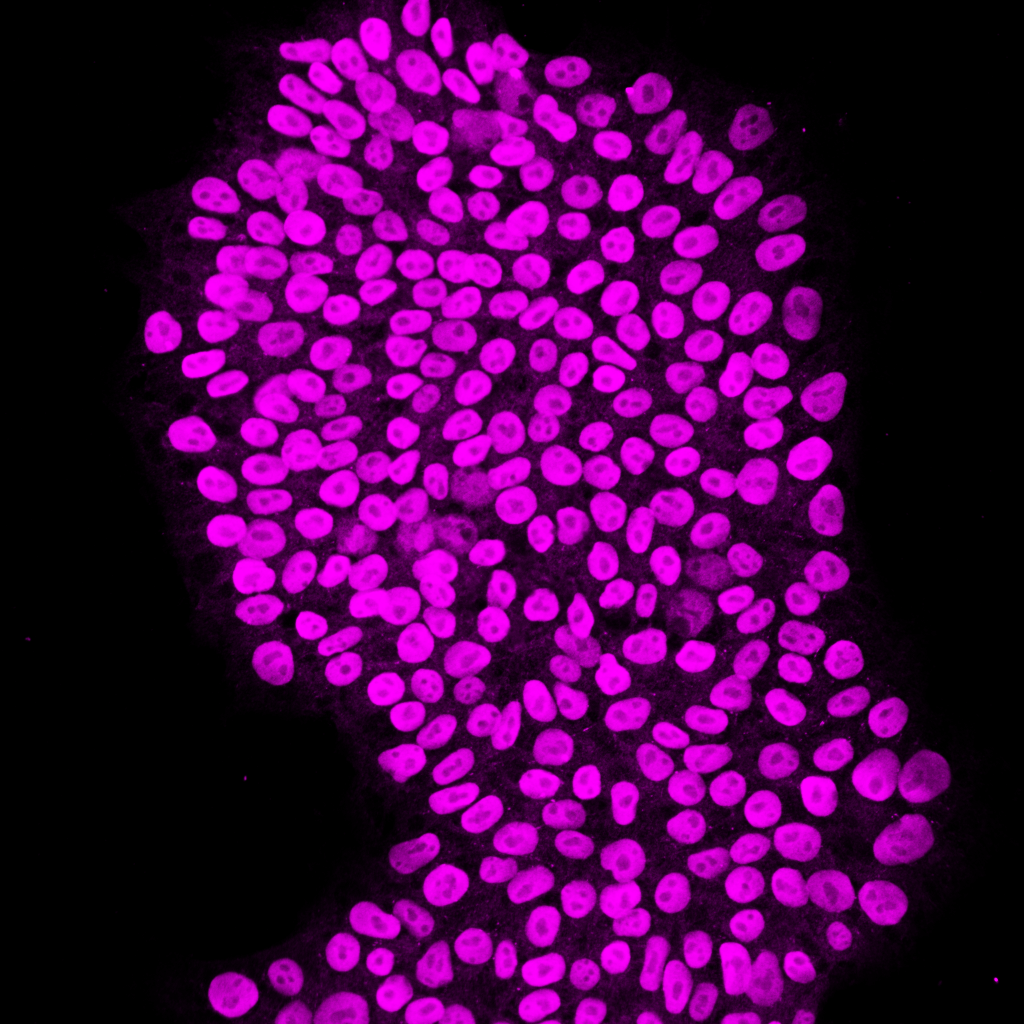

Supplement: Supplementary file 14 — Appendix Figure Source Data [file 44321_2025_302_MOESM14_ESM.zip › Appendix Figure S2/S2D/H9-NANOG.tif]

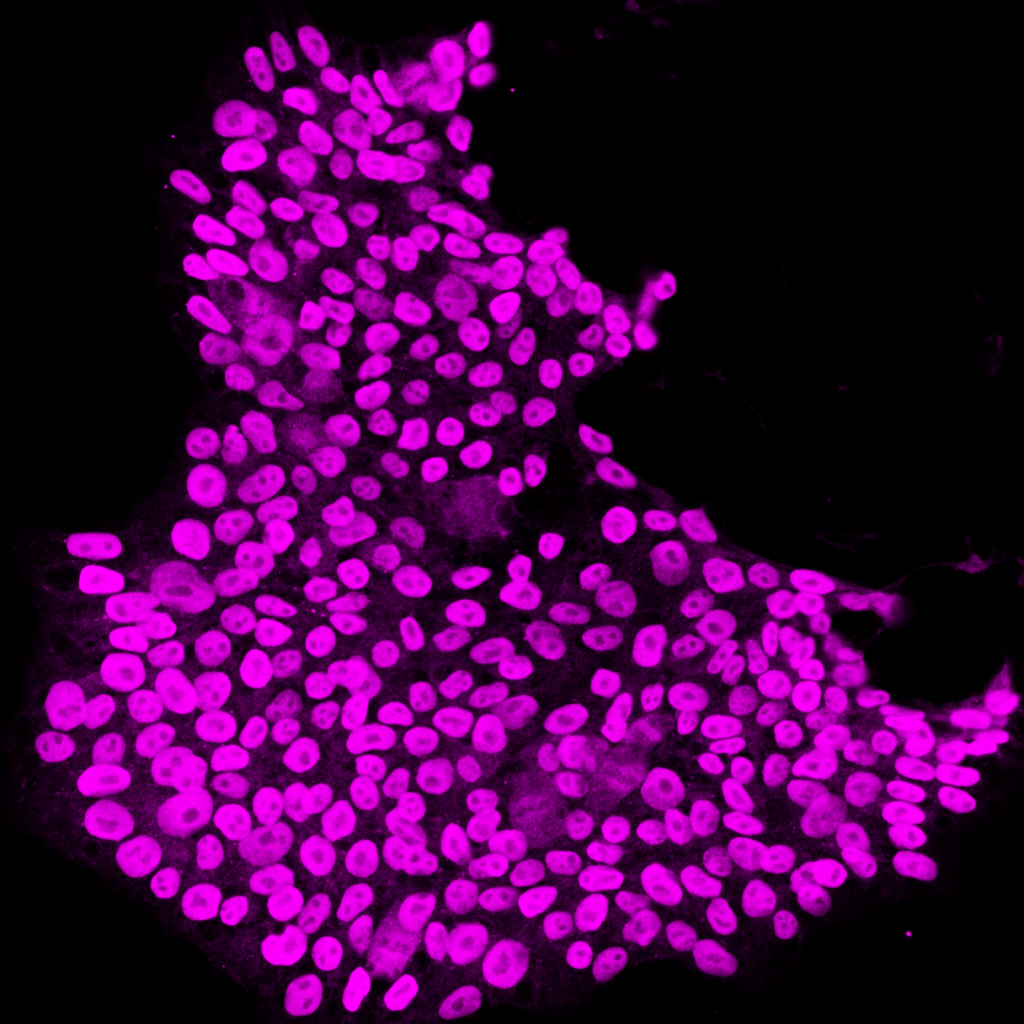

Supplement: Supplementary file 14 — Appendix Figure Source Data [file 44321_2025_302_MOESM14_ESM.zip › Appendix Figure S2/S2D/#7-5-NANOG.tif]

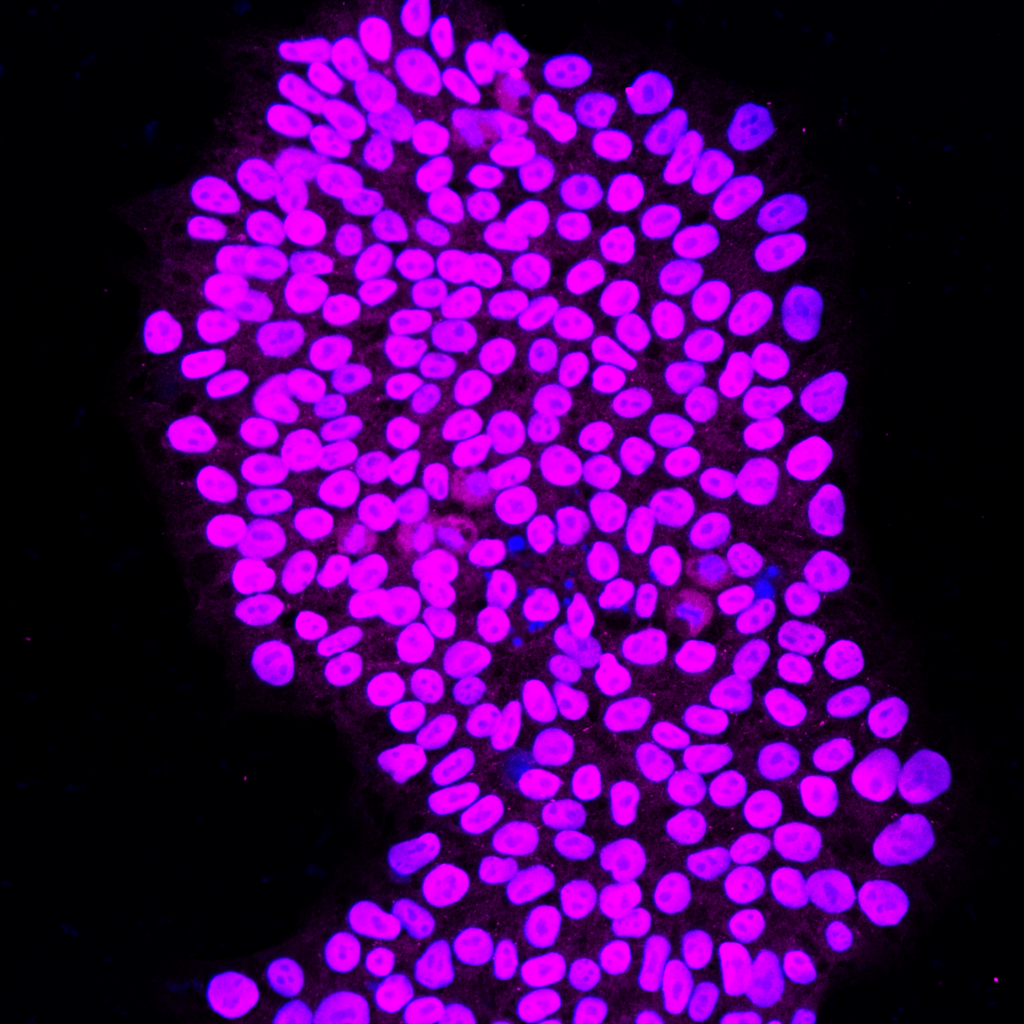

Supplement: Supplementary file 14 — Appendix Figure Source Data [file 44321_2025_302_MOESM14_ESM.zip › Appendix Figure S2/S2D/H9-merge.tif]

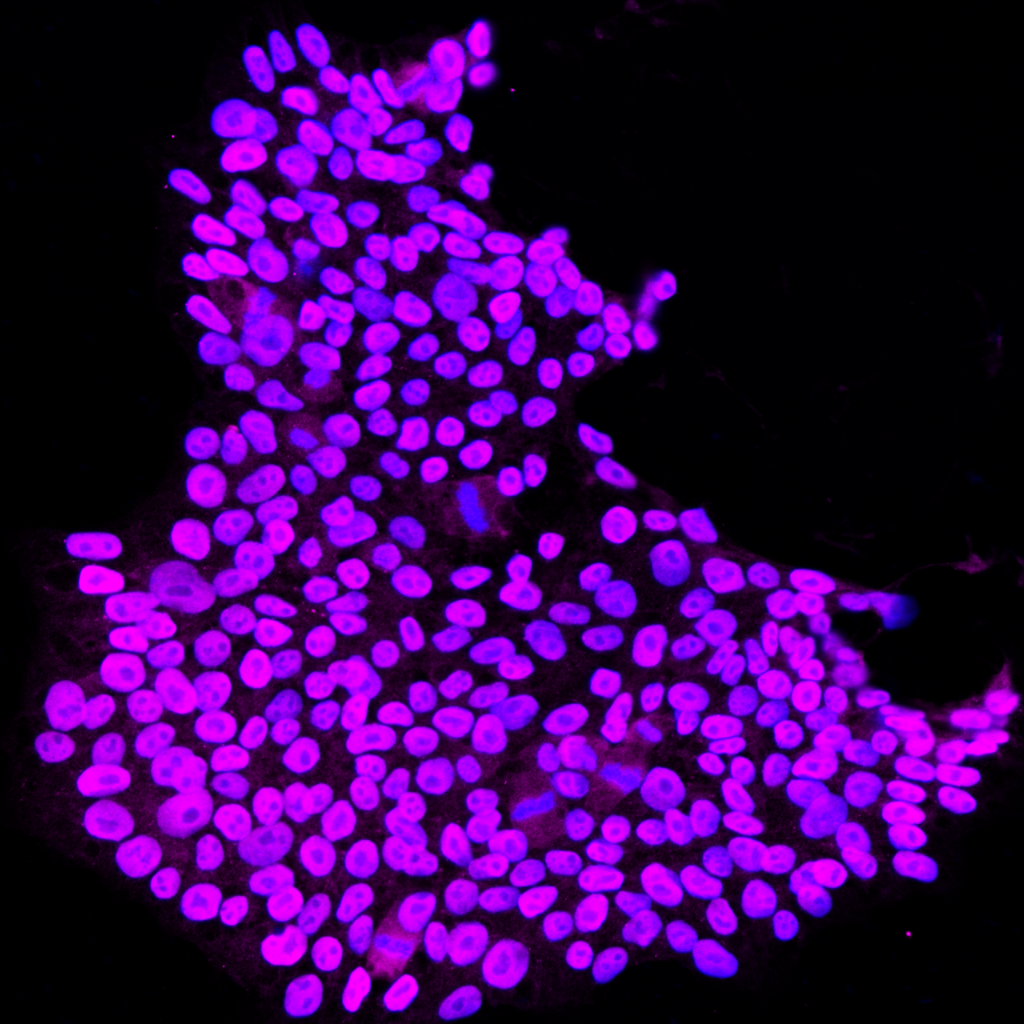

Supplement: Supplementary file 14 — Appendix Figure Source Data [file 44321_2025_302_MOESM14_ESM.zip › Appendix Figure S2/S2D/#7-5-merge.tif]

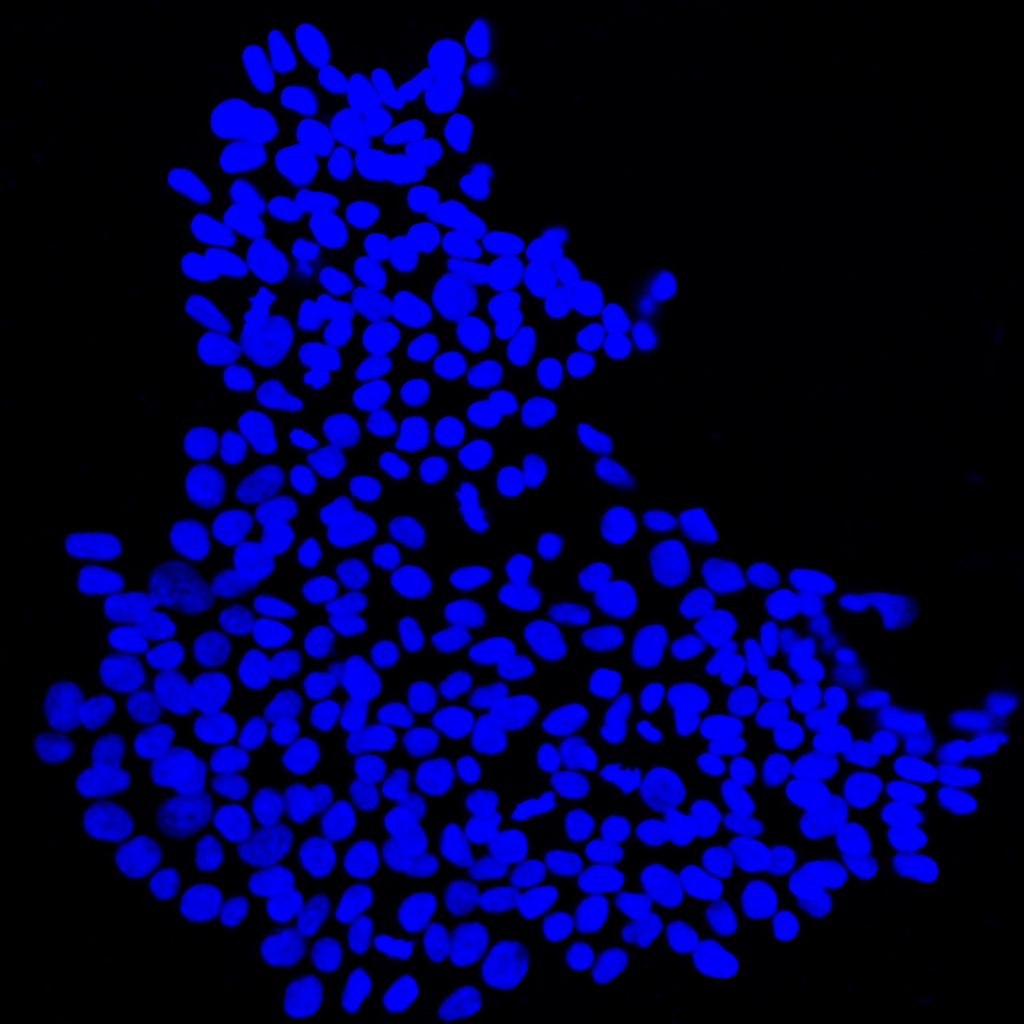

Supplement: Supplementary file 14 — Appendix Figure Source Data [file 44321_2025_302_MOESM14_ESM.zip › Appendix Figure S2/S2D/#7-5-DAPI.tif]

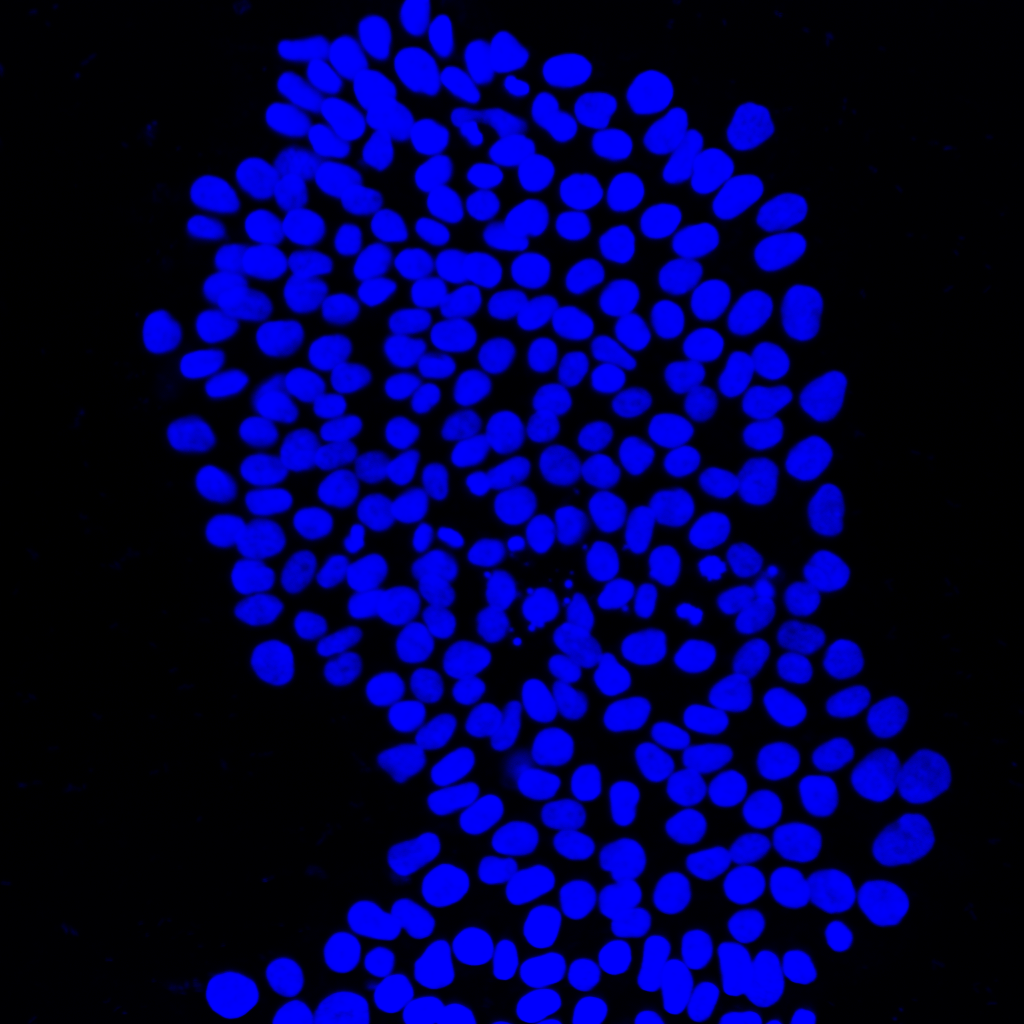

Supplement: Supplementary file 14 — Appendix Figure Source Data [file 44321_2025_302_MOESM14_ESM.zip › Appendix Figure S2/S2D/H9-DAPI.tif]

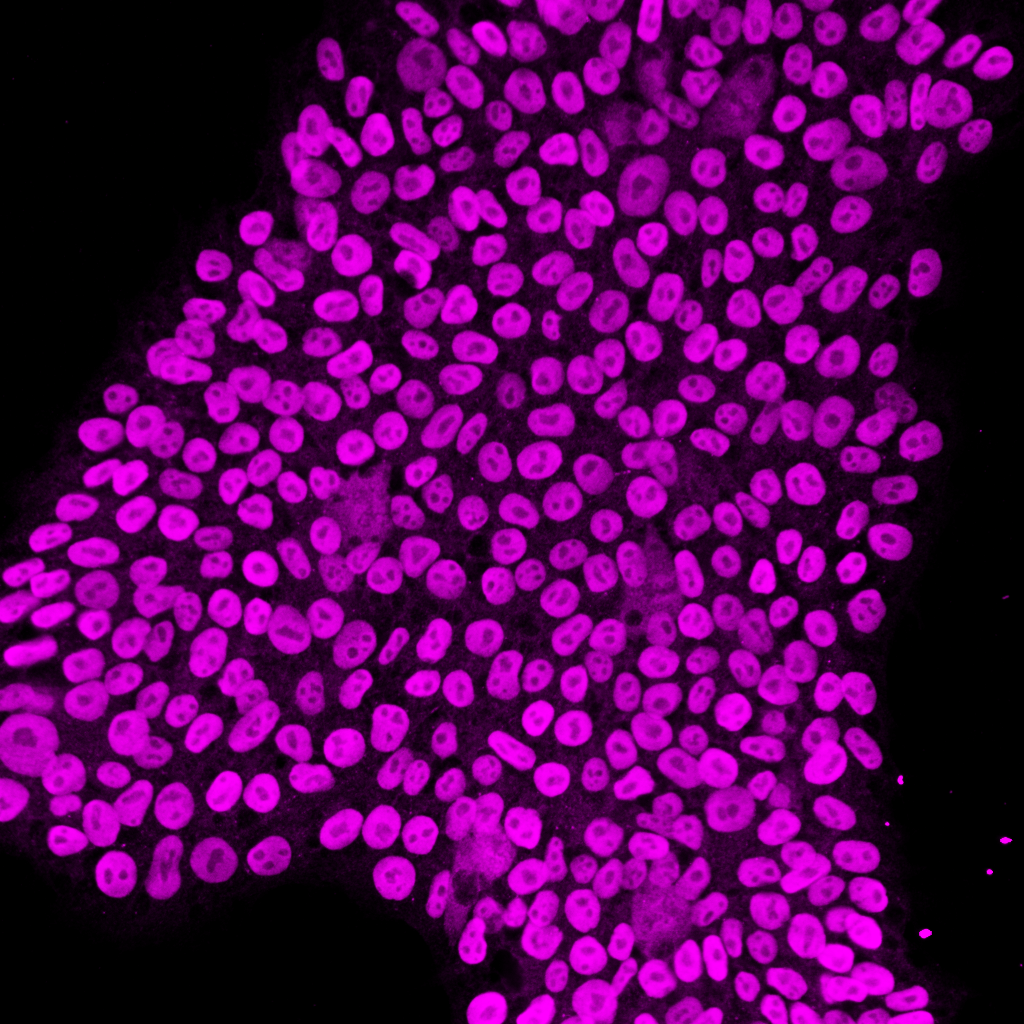

Supplement: Supplementary file 14 — Appendix Figure Source Data [file 44321_2025_302_MOESM14_ESM.zip › Appendix Figure S2/S2D/#12-3-NANOG.tif]

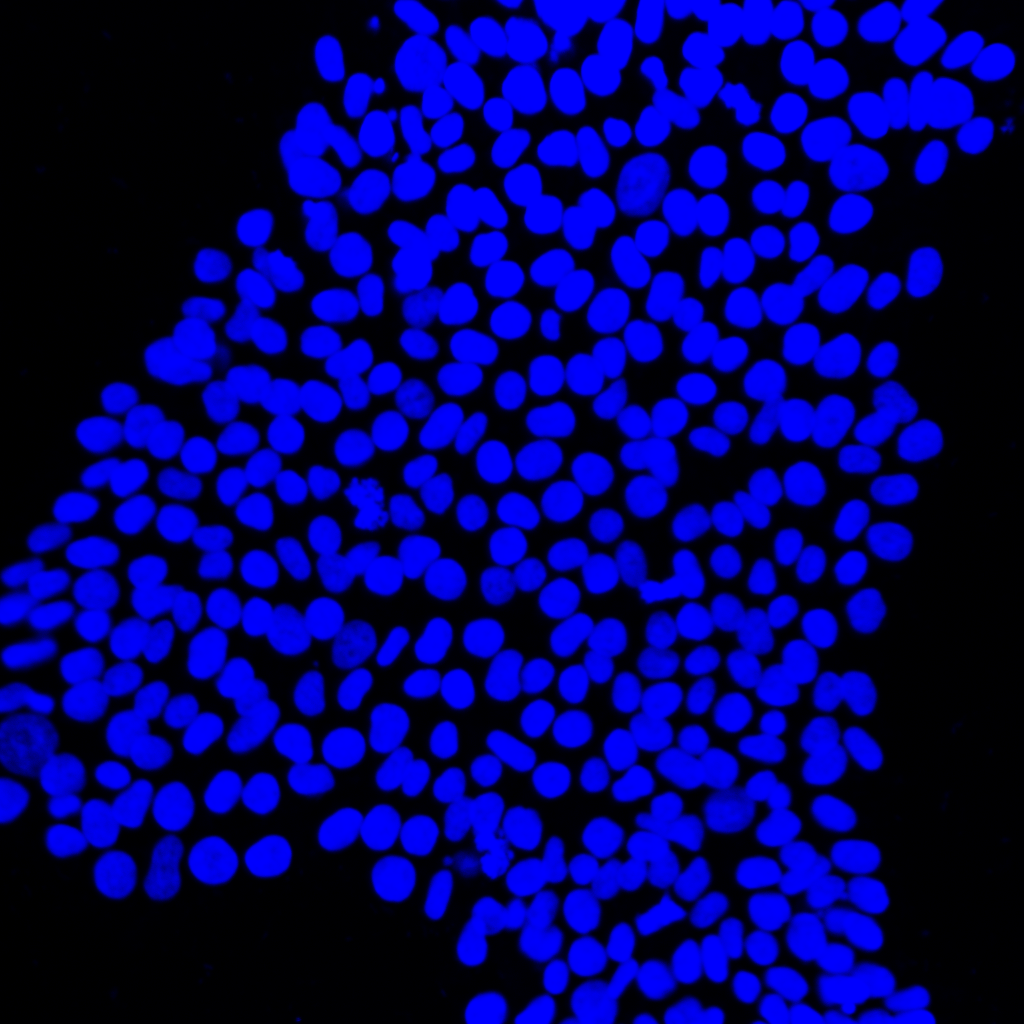

Supplement: Supplementary file 14 — Appendix Figure Source Data [file 44321_2025_302_MOESM14_ESM.zip › Appendix Figure S2/S2D/#12-3-DAPI.tif]

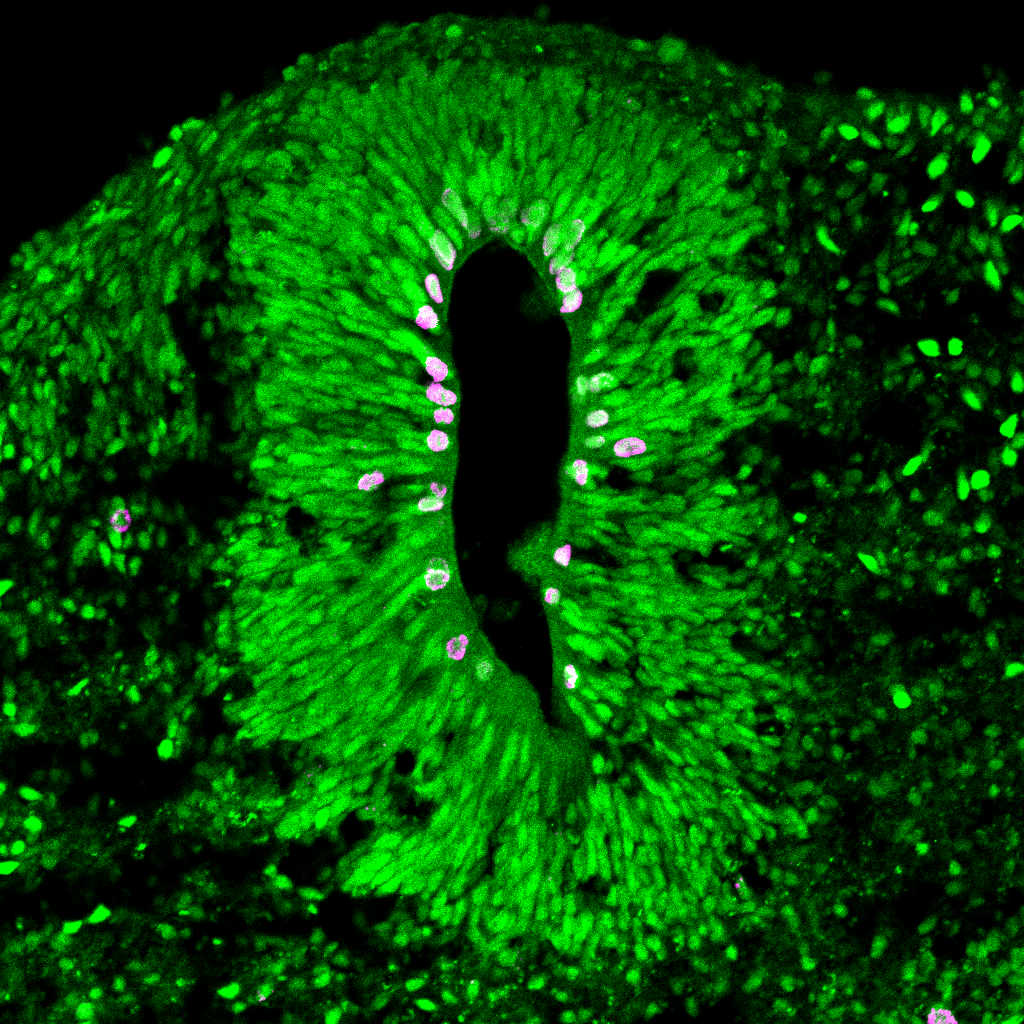

Supplement: Supplementary file 14 — Appendix Figure Source Data [file 44321_2025_302_MOESM14_ESM.zip › Appendix Figure S3/S3C/#12-3-merge.tif]

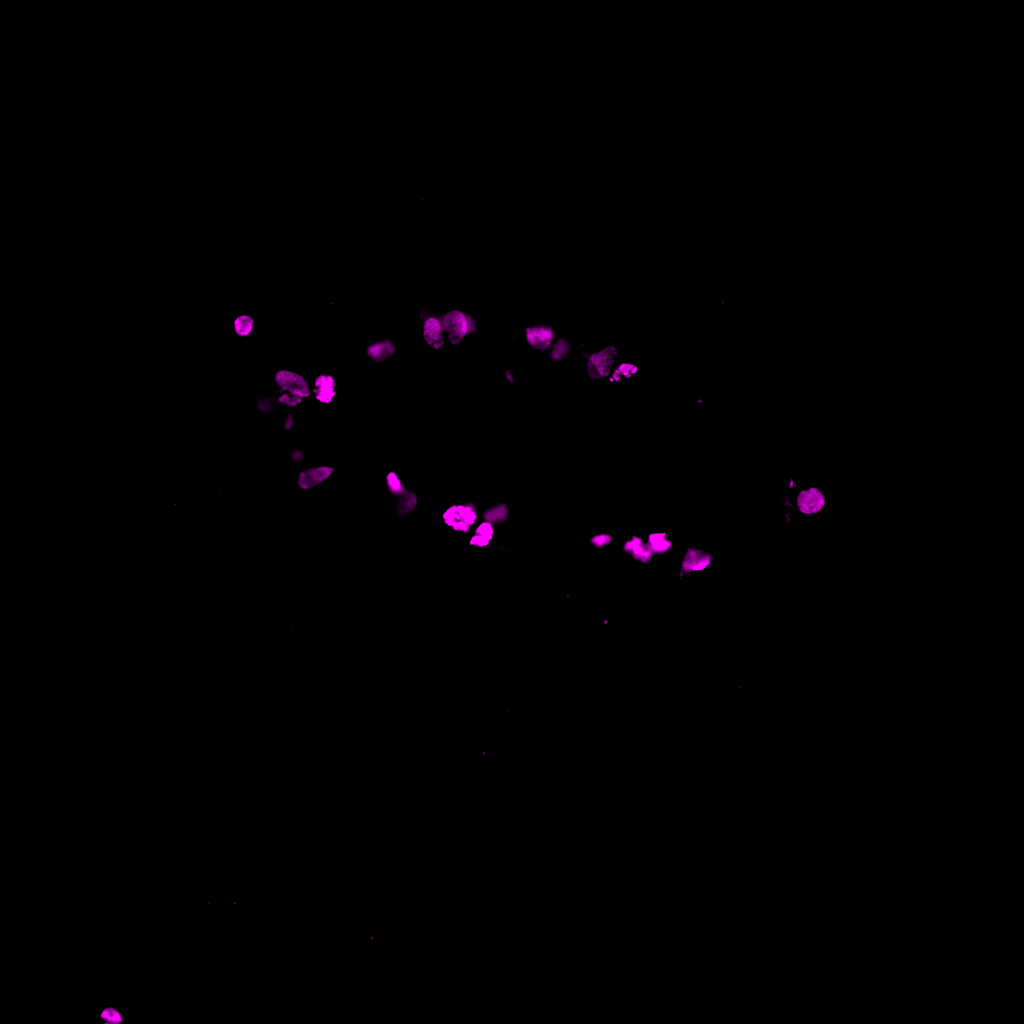

Supplement: Supplementary file 14 — Appendix Figure Source Data [file 44321_2025_302_MOESM14_ESM.zip › Appendix Figure S3/S3C/#7-5-PH3.tif]

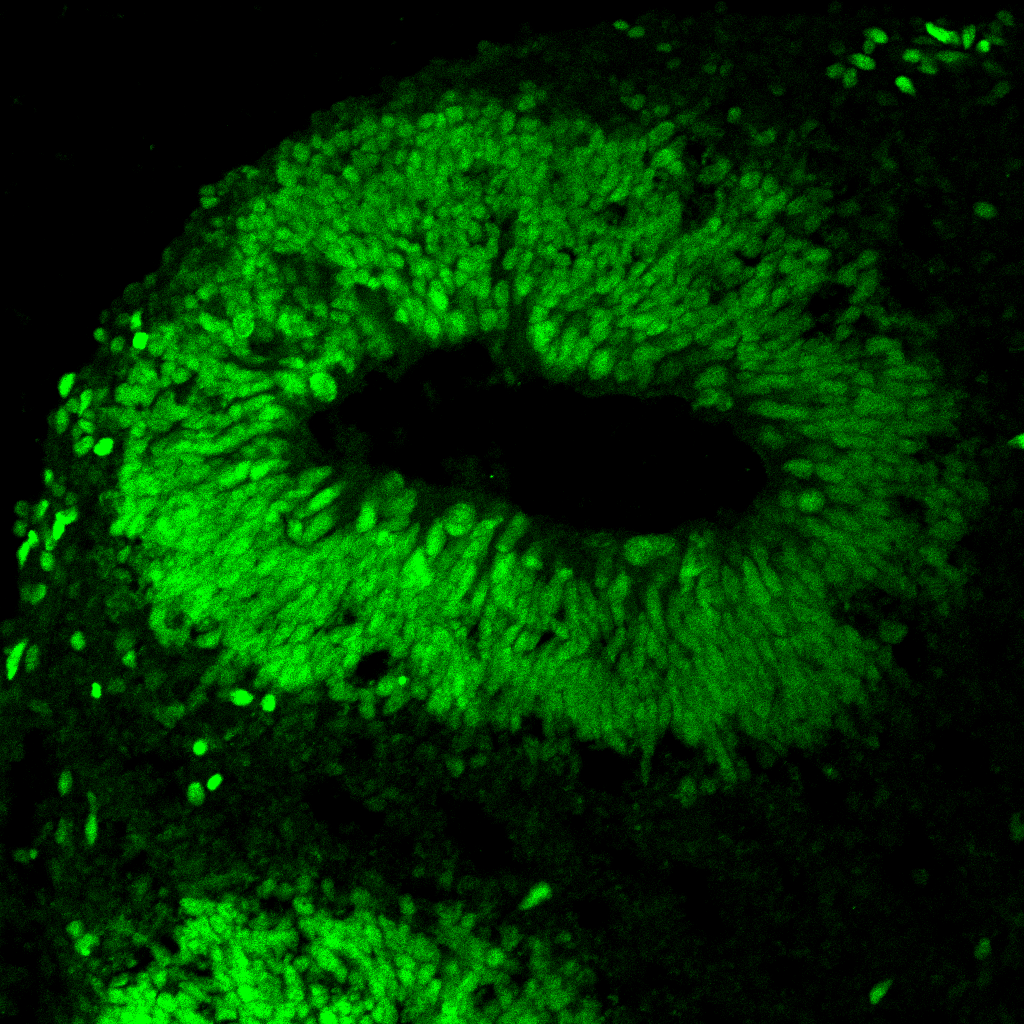

Supplement: Supplementary file 14 — Appendix Figure Source Data [file 44321_2025_302_MOESM14_ESM.zip › Appendix Figure S3/S3C/#7-5-PAX6.tif]

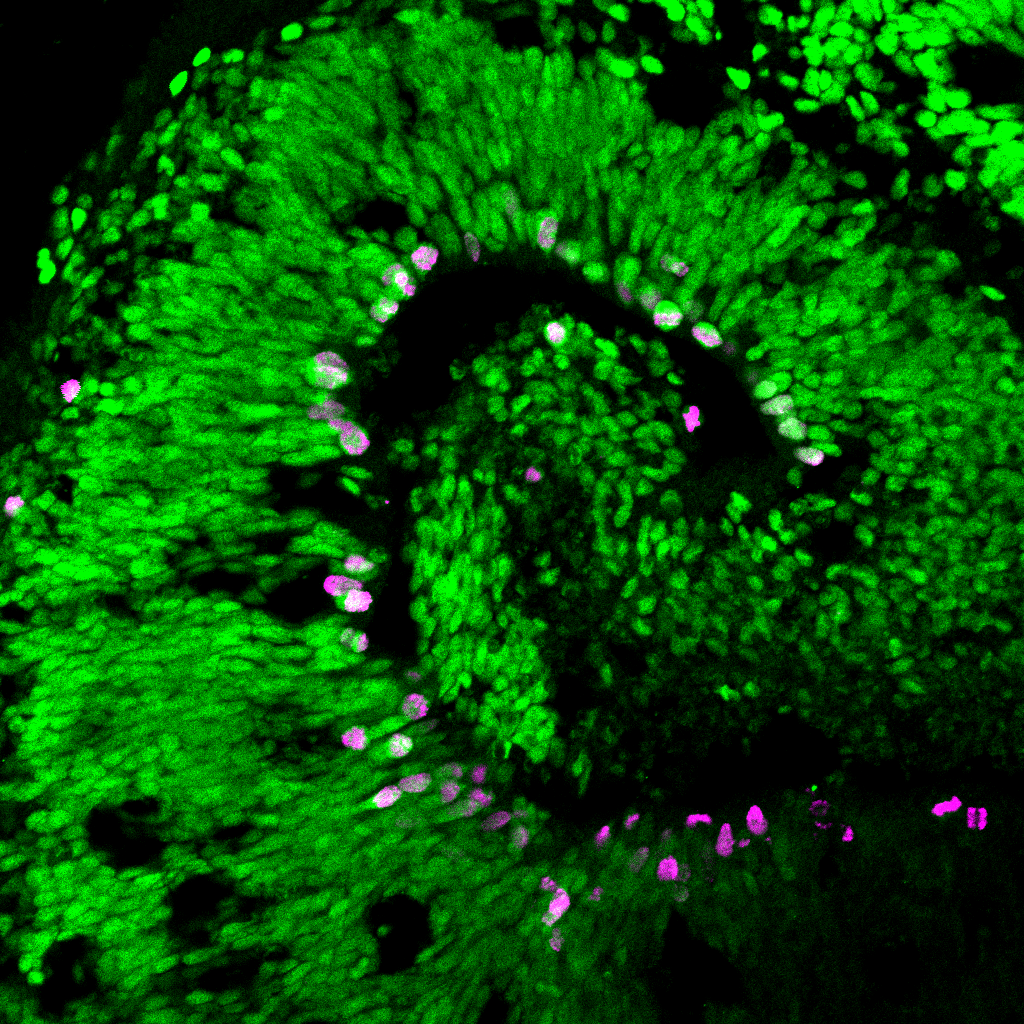

Supplement: Supplementary file 14 — Appendix Figure Source Data [file 44321_2025_302_MOESM14_ESM.zip › Appendix Figure S3/S3C/H9-merge.tif]

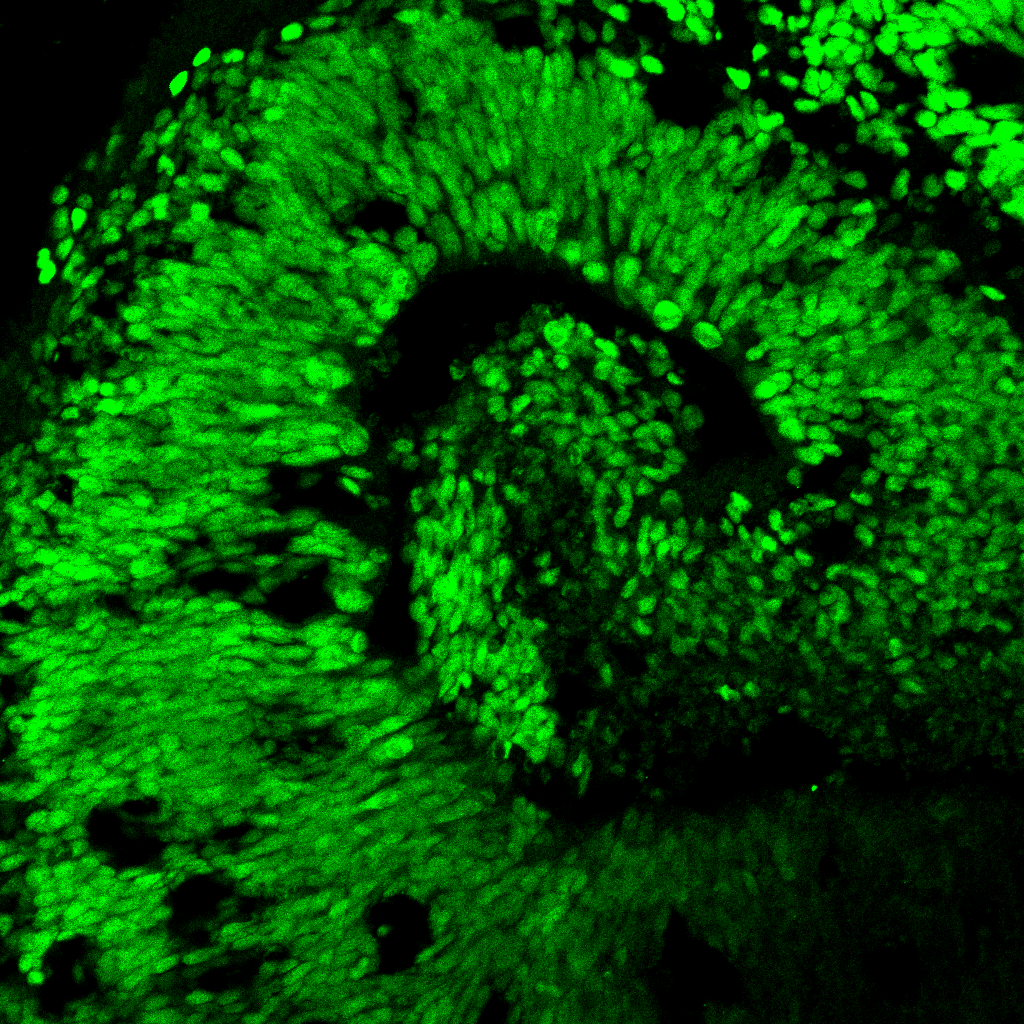

Supplement: Supplementary file 14 — Appendix Figure Source Data [file 44321_2025_302_MOESM14_ESM.zip › Appendix Figure S3/S3C/H9-PAX6.tif]

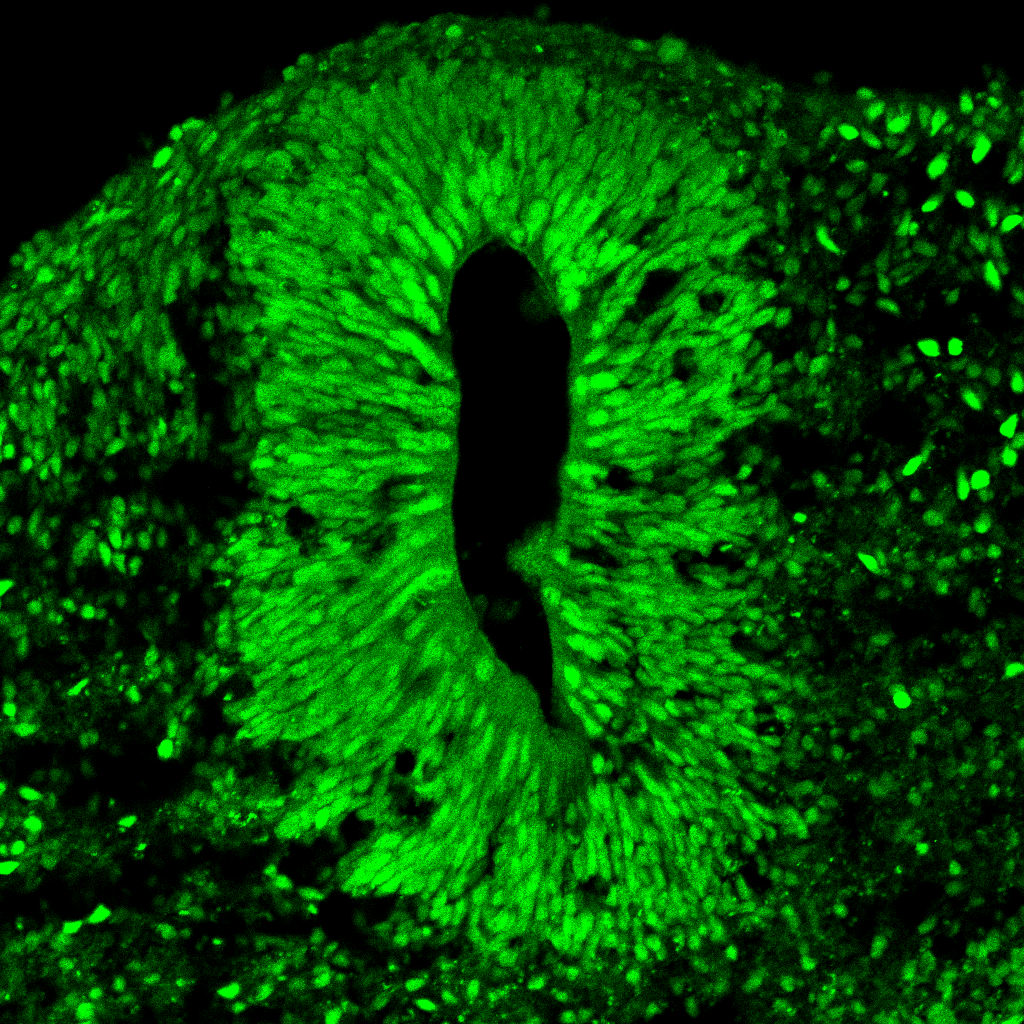

Supplement: Supplementary file 14 — Appendix Figure Source Data [file 44321_2025_302_MOESM14_ESM.zip › Appendix Figure S3/S3C/#12-3-PAX6.tif]

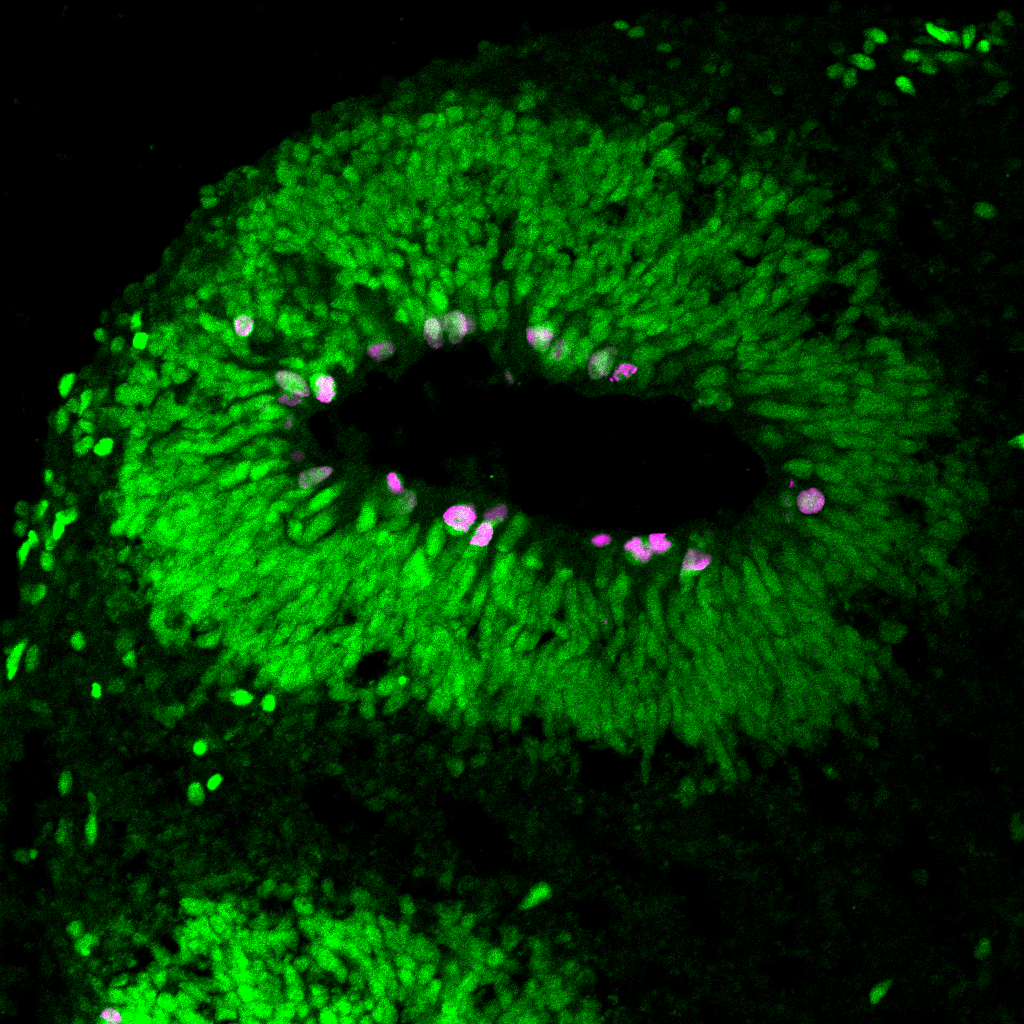

Supplement: Supplementary file 14 — Appendix Figure Source Data [file 44321_2025_302_MOESM14_ESM.zip › Appendix Figure S3/S3C/#7-5-merge.tif]

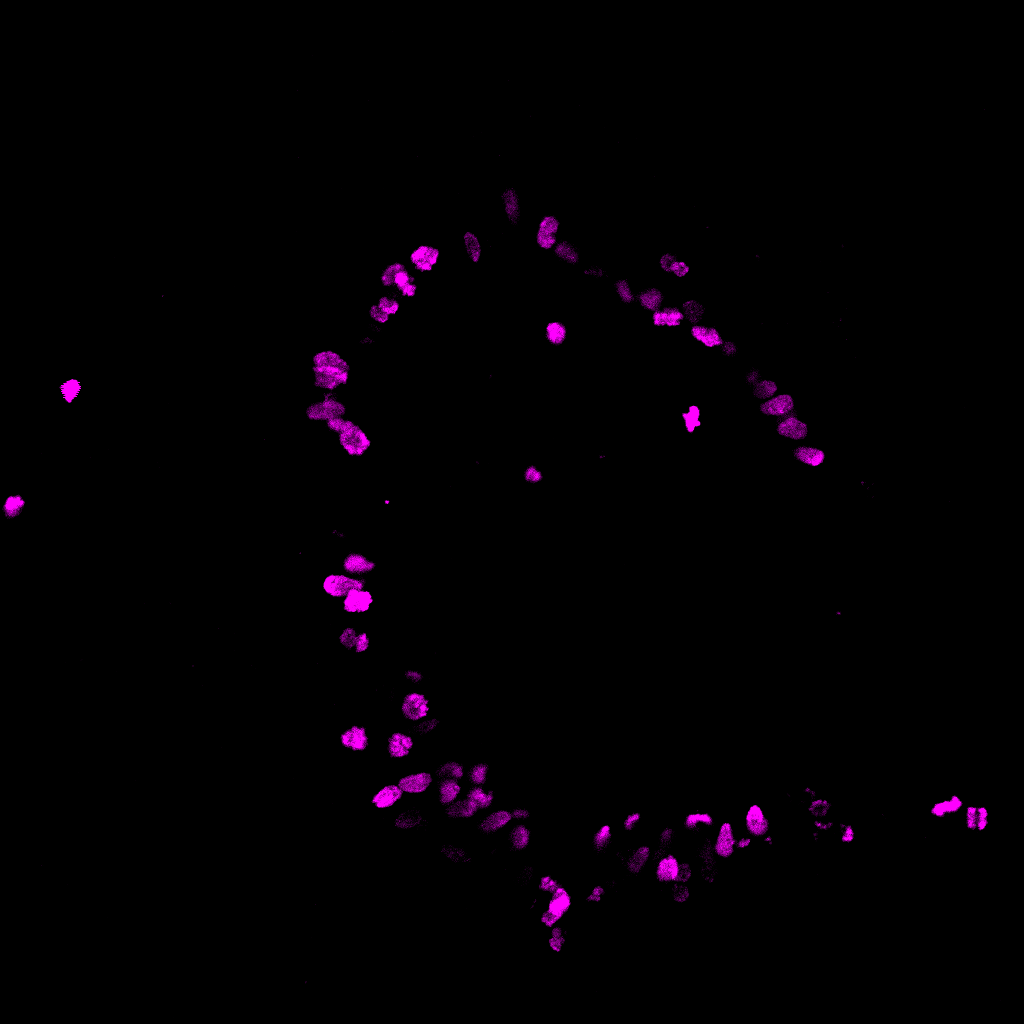

Supplement: Supplementary file 14 — Appendix Figure Source Data [file 44321_2025_302_MOESM14_ESM.zip › Appendix Figure S3/S3C/H9-PH3.tif]

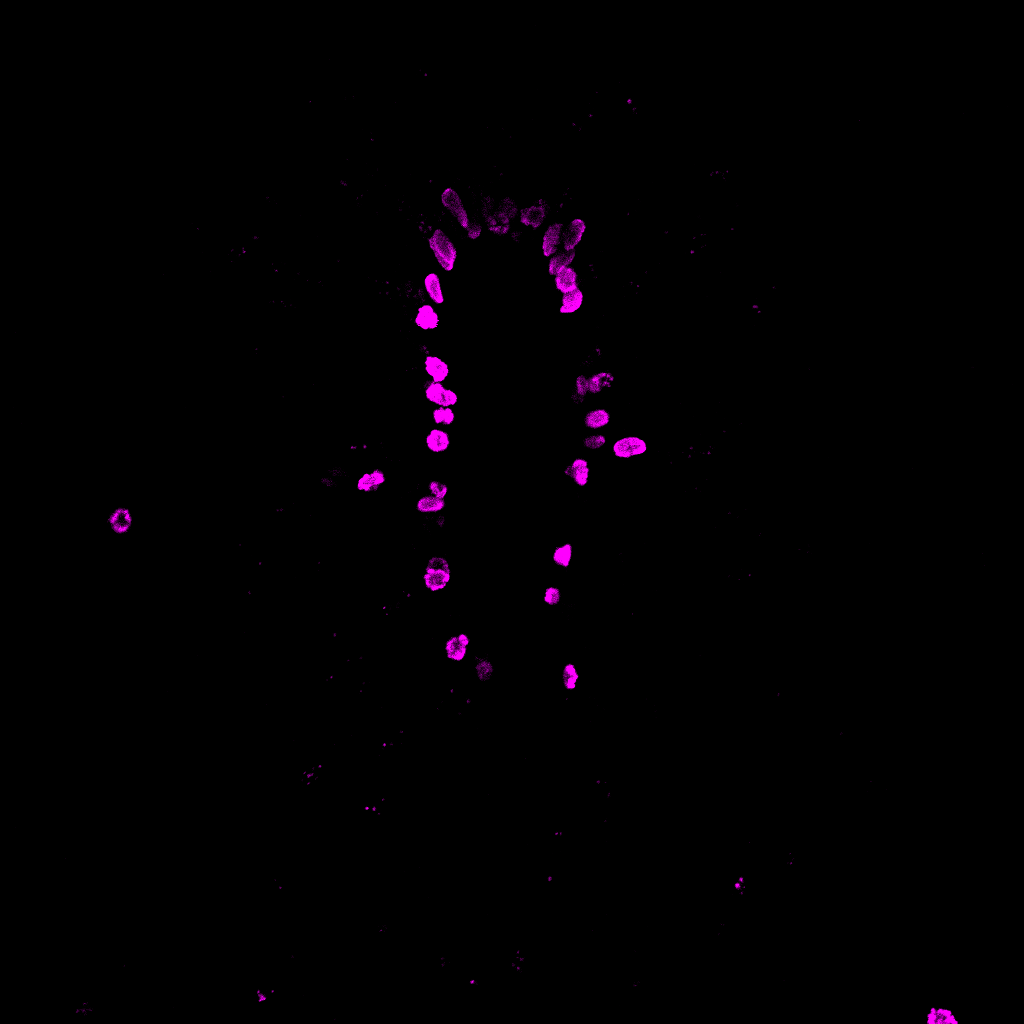

Supplement: Supplementary file 14 — Appendix Figure Source Data [file 44321_2025_302_MOESM14_ESM.zip › Appendix Figure S3/S3C/#12-3-PH3.tif]

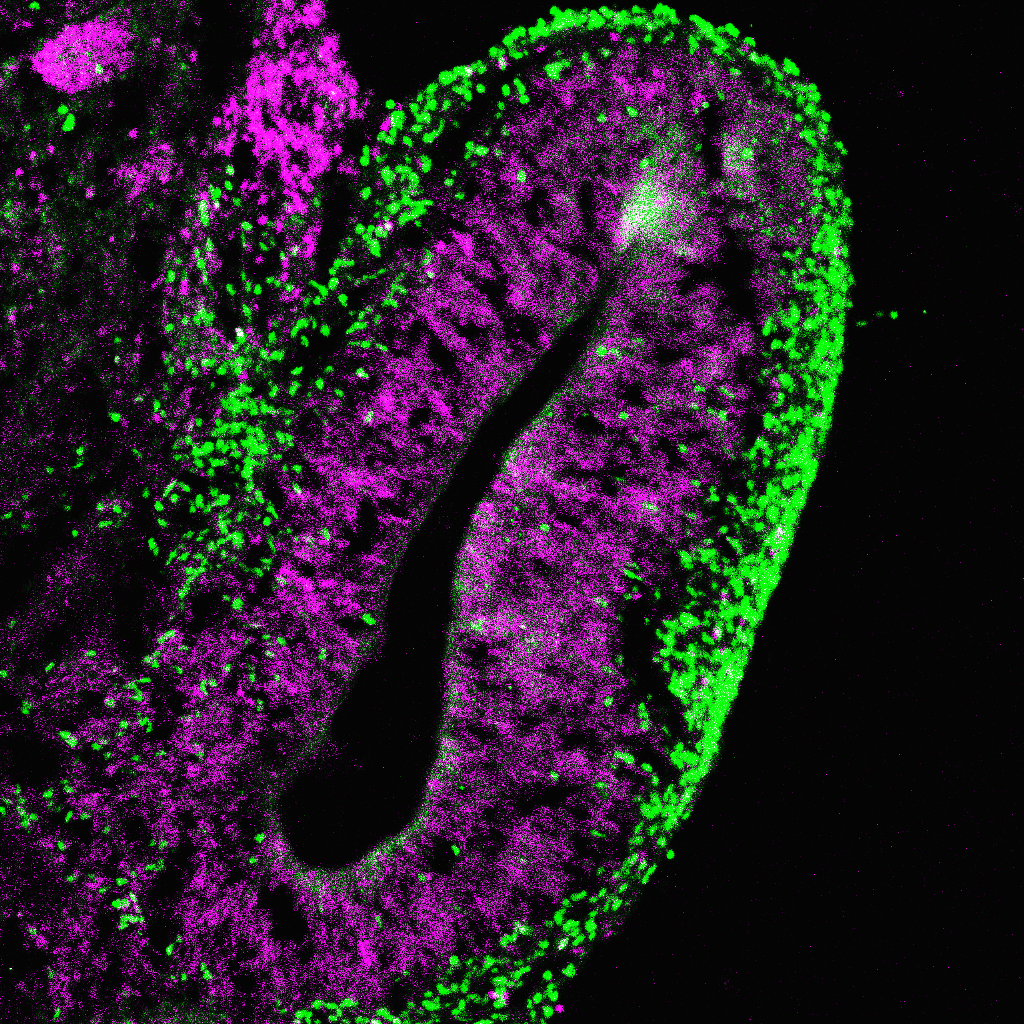

Supplement: Supplementary file 14 — Appendix Figure Source Data [file 44321_2025_302_MOESM14_ESM.zip › Appendix Figure S3/S3B/#12-3-merge.tif]

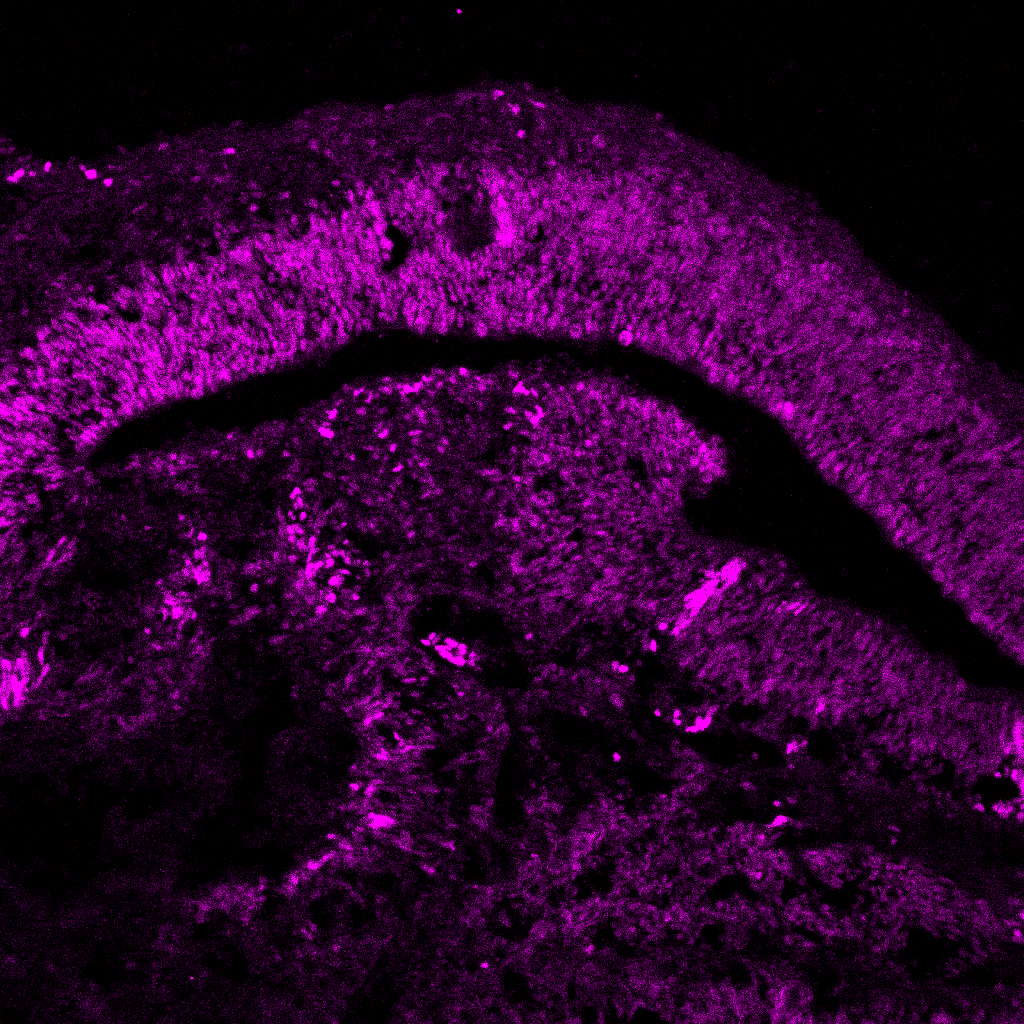

Supplement: Supplementary file 14 — Appendix Figure Source Data [file 44321_2025_302_MOESM14_ESM.zip › Appendix Figure S3/S3B/#7-5-PAX6.tif]

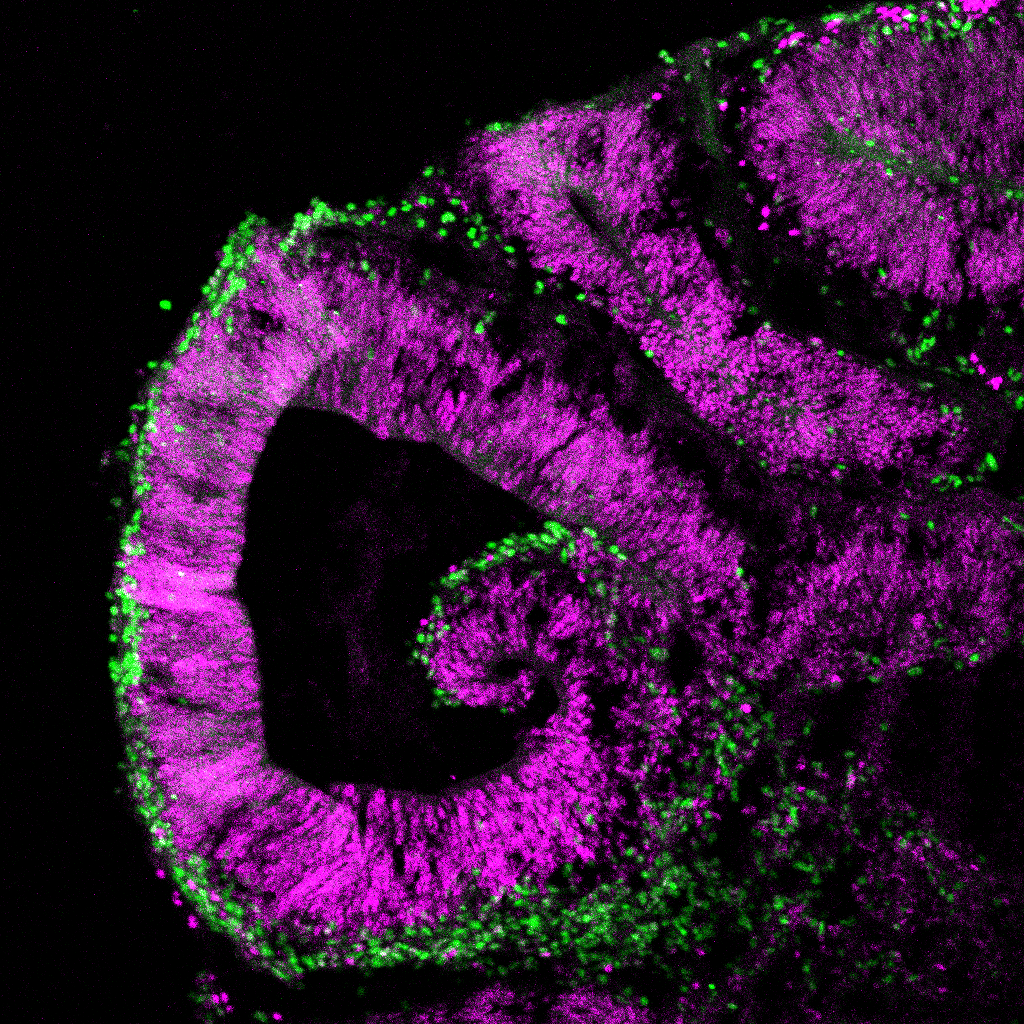

Supplement: Supplementary file 14 — Appendix Figure Source Data [file 44321_2025_302_MOESM14_ESM.zip › Appendix Figure S3/S3B/H9-merge.tif]

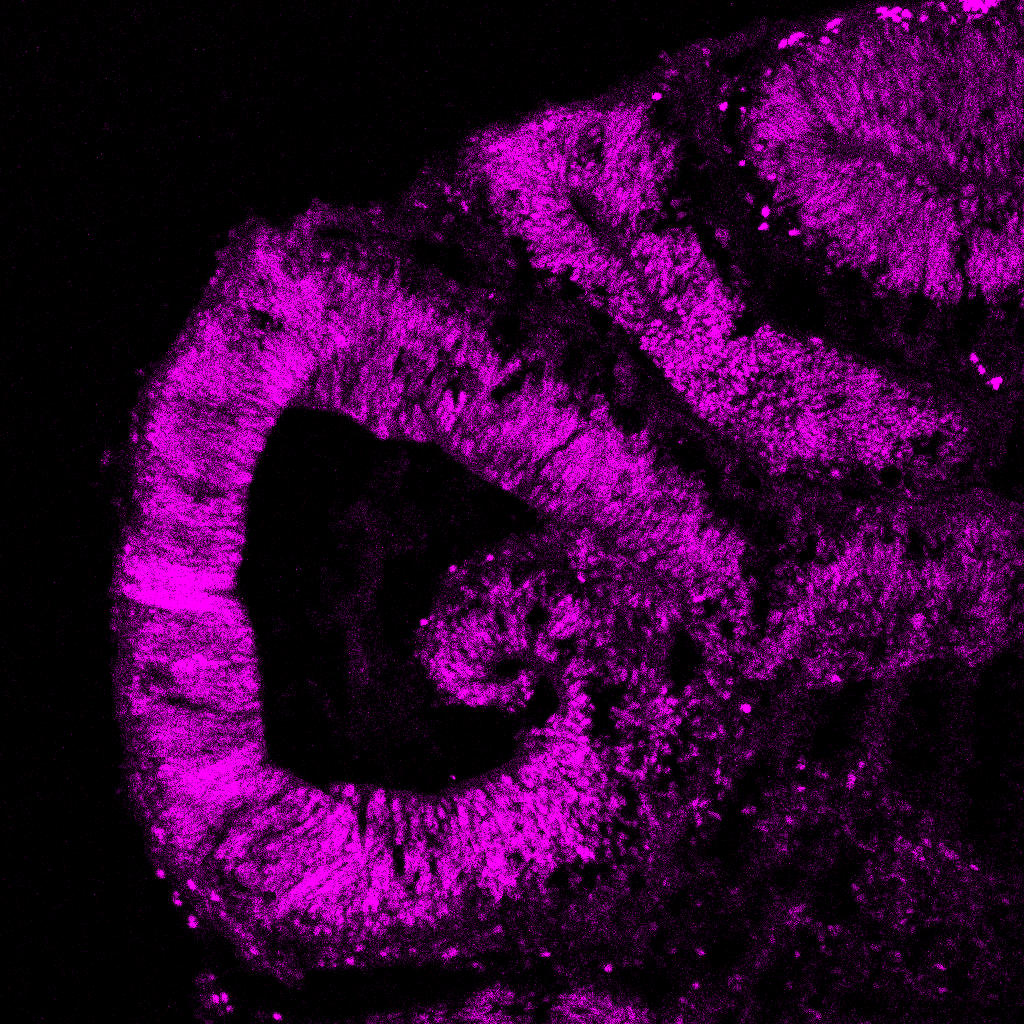

Supplement: Supplementary file 14 — Appendix Figure Source Data [file 44321_2025_302_MOESM14_ESM.zip › Appendix Figure S3/S3B/H9-PAX6.tif]

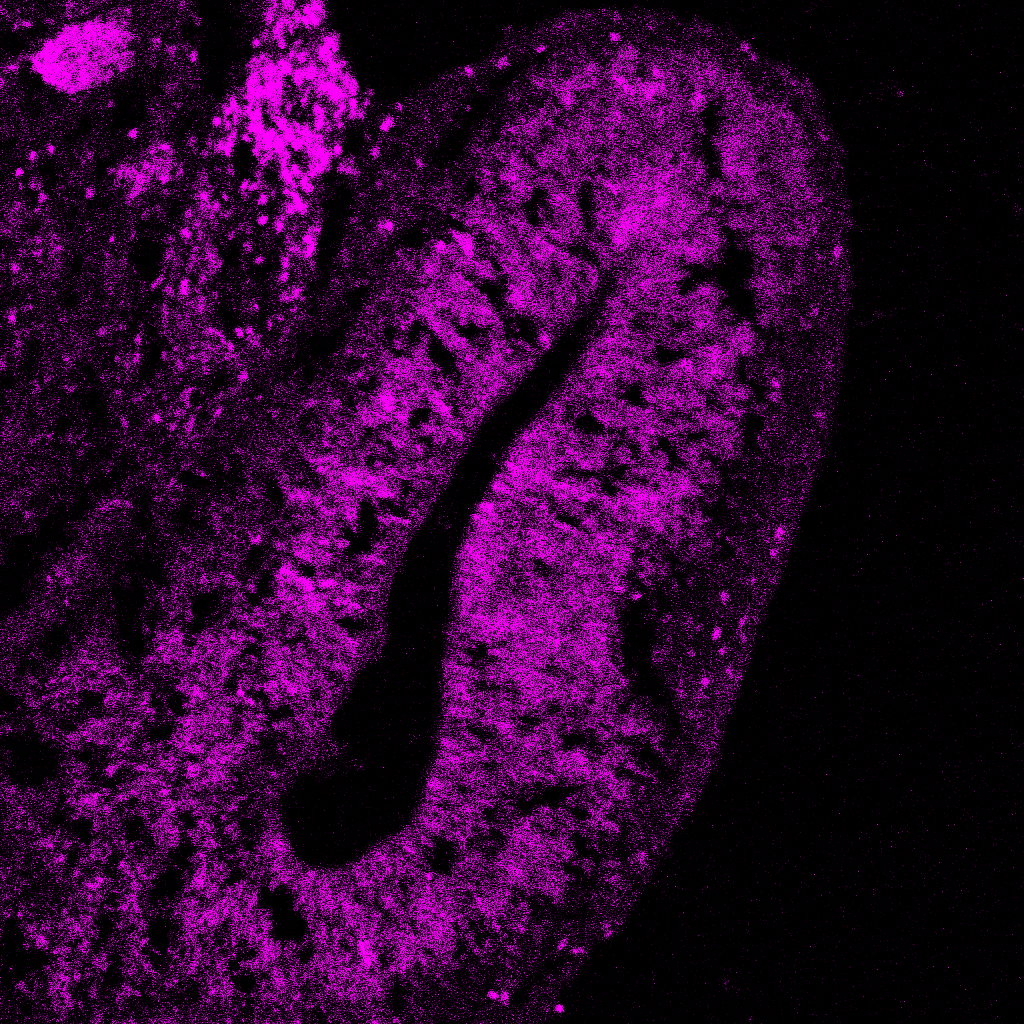

Supplement: Supplementary file 14 — Appendix Figure Source Data [file 44321_2025_302_MOESM14_ESM.zip › Appendix Figure S3/S3B/#12-3-PAX6.tif]

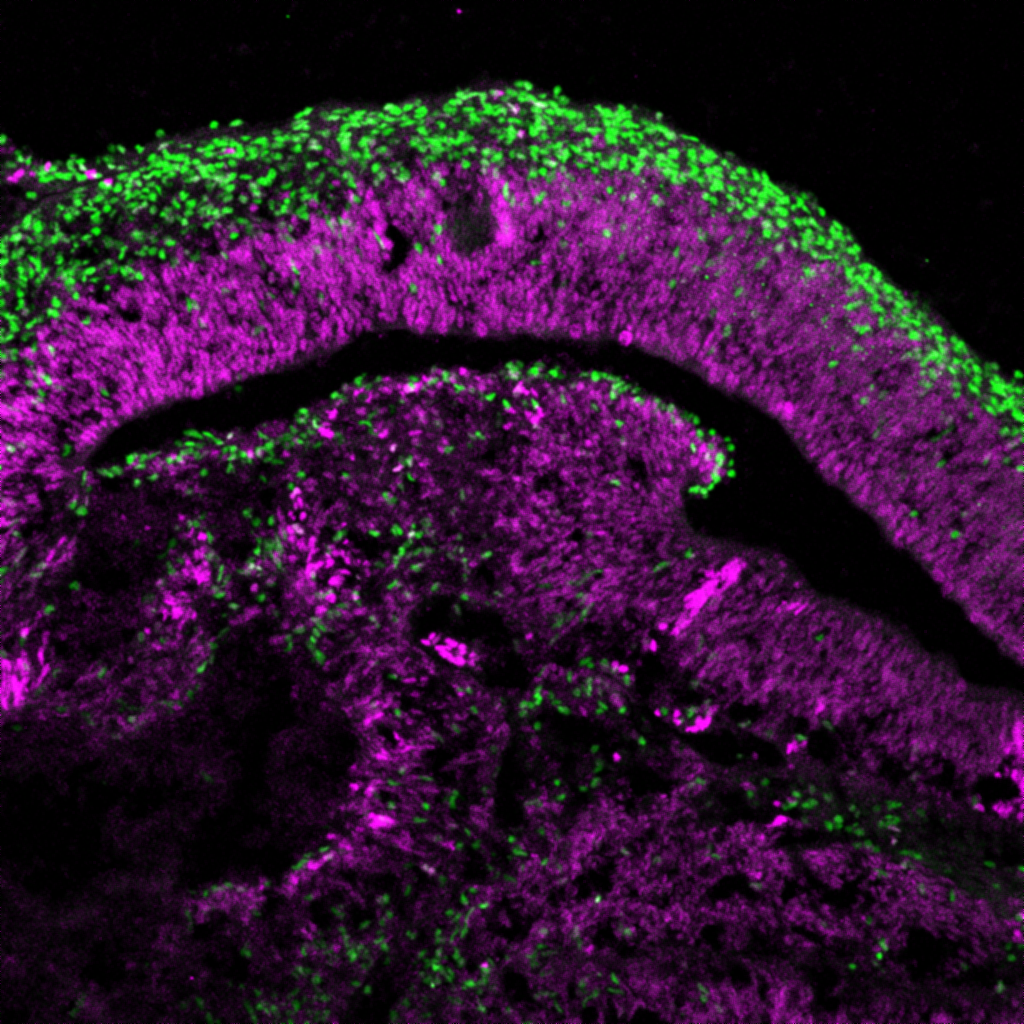

Supplement: Supplementary file 14 — Appendix Figure Source Data [file 44321_2025_302_MOESM14_ESM.zip › Appendix Figure S3/S3B/#7-5-merge.tif]

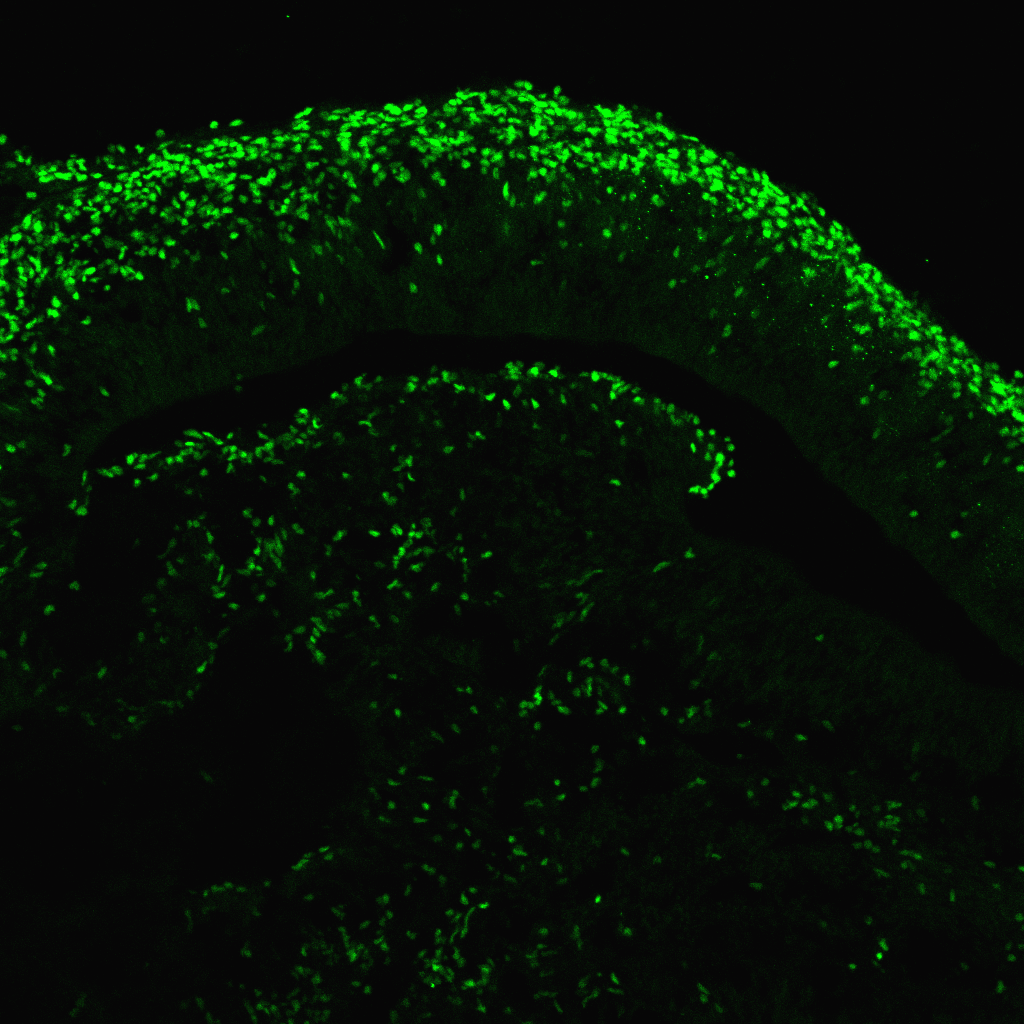

Supplement: Supplementary file 14 — Appendix Figure Source Data [file 44321_2025_302_MOESM14_ESM.zip › Appendix Figure S3/S3B/#7-5-TBR1.tif]

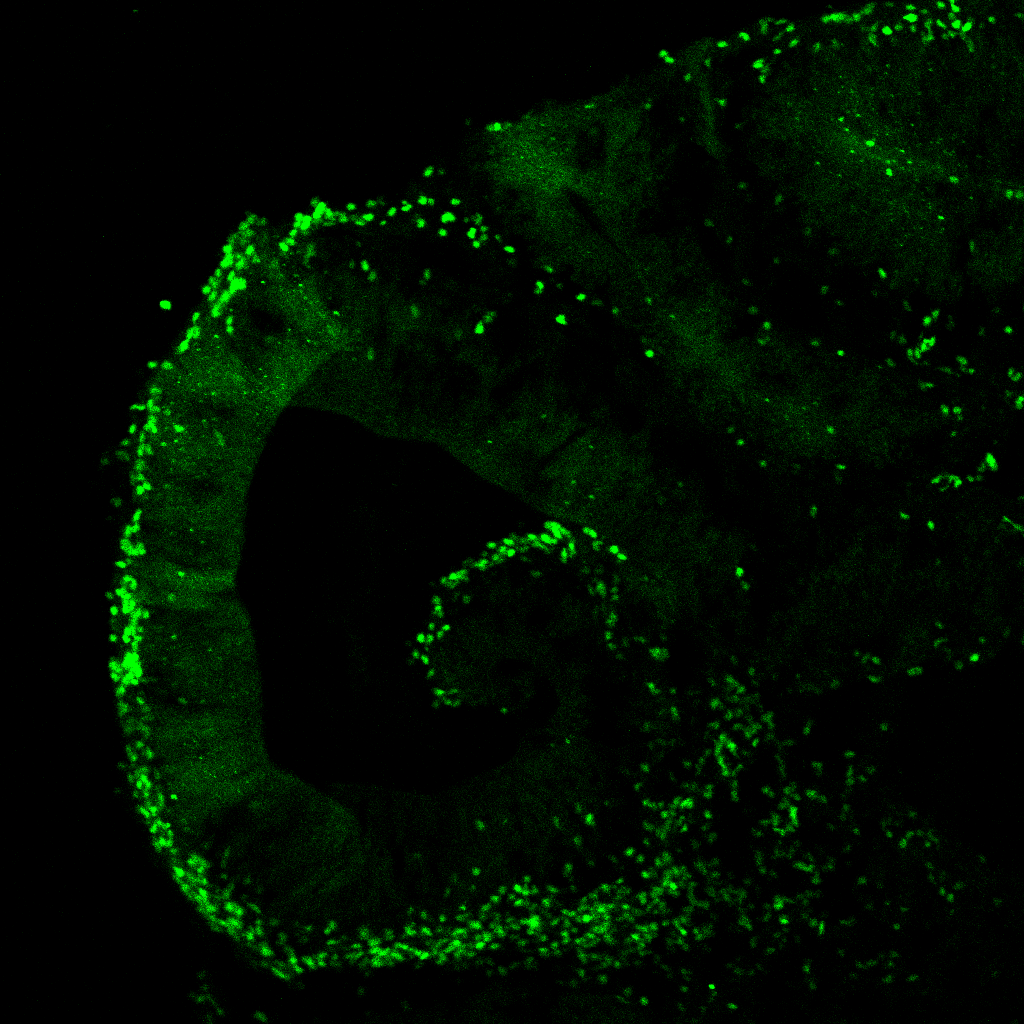

Supplement: Supplementary file 14 — Appendix Figure Source Data [file 44321_2025_302_MOESM14_ESM.zip › Appendix Figure S3/S3B/H9-TBR1.tif]

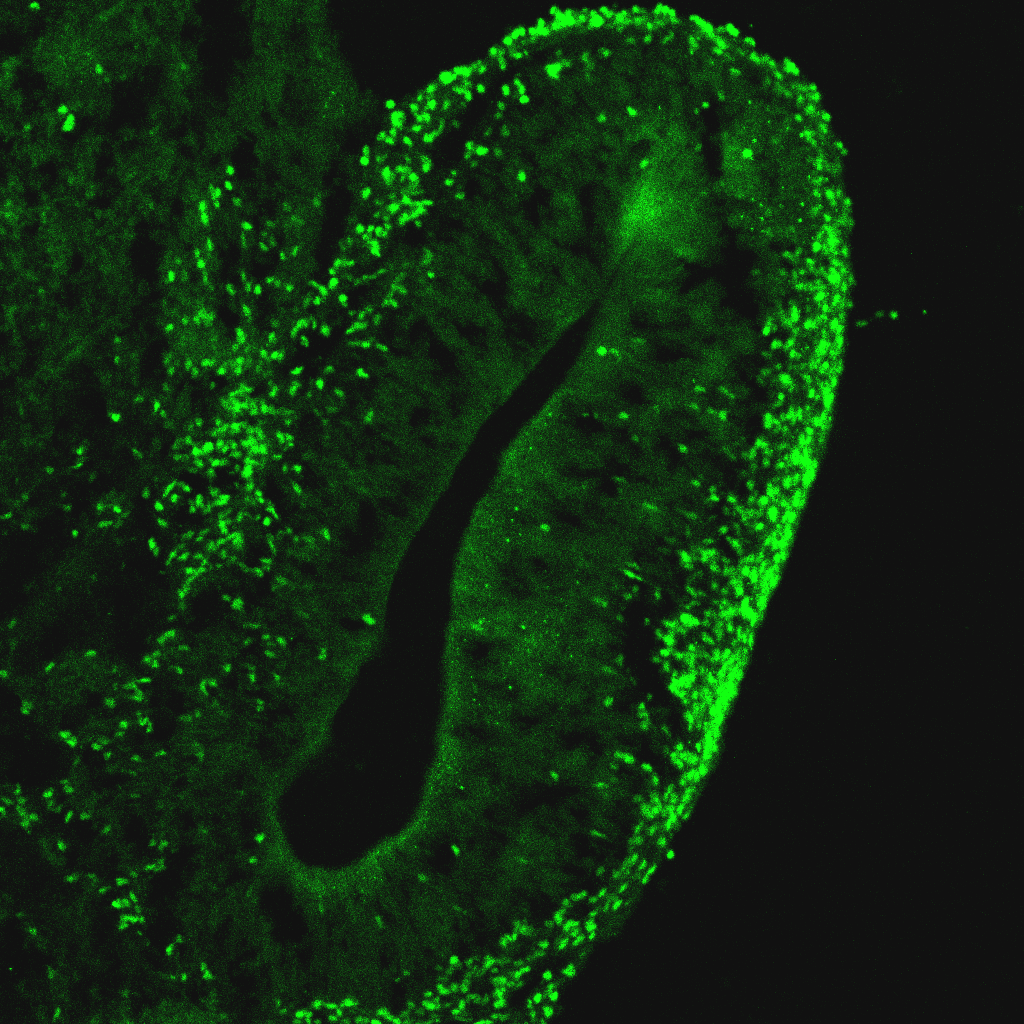

Supplement: Supplementary file 14 — Appendix Figure Source Data [file 44321_2025_302_MOESM14_ESM.zip › Appendix Figure S3/S3B/#12-3-TBR1.tif]

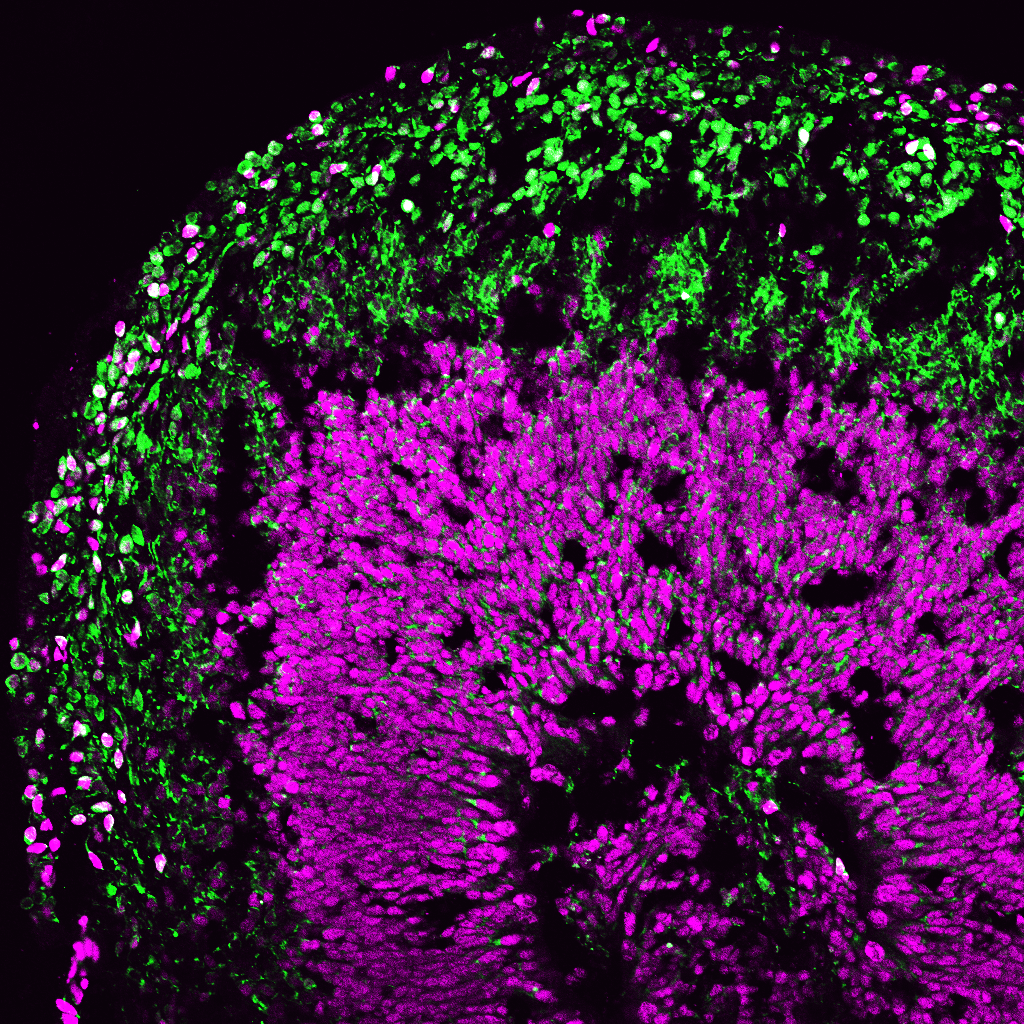

Supplement: Supplementary file 14 — Appendix Figure Source Data [file 44321_2025_302_MOESM14_ESM.zip › Appendix Figure S3/S3A/#12-3-merge.tif]

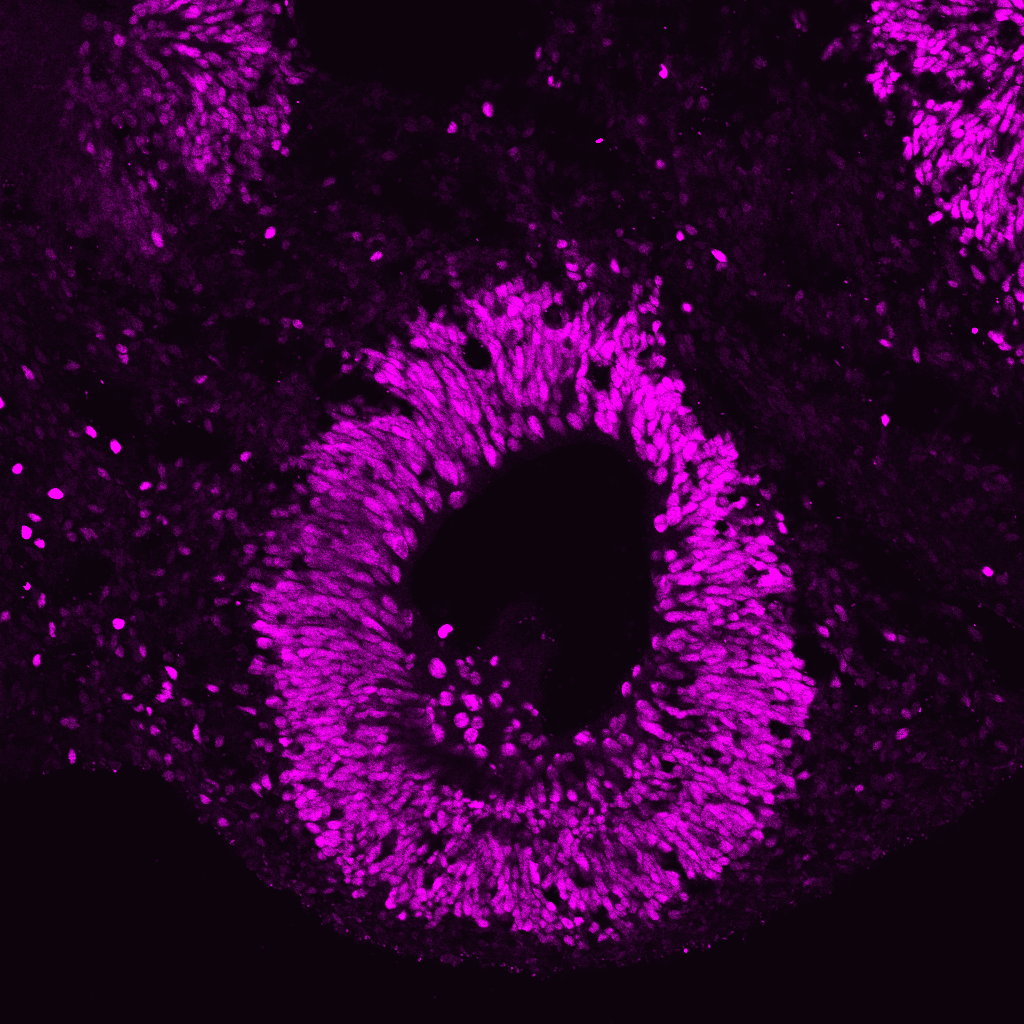

Supplement: Supplementary file 14 — Appendix Figure Source Data [file 44321_2025_302_MOESM14_ESM.zip › Appendix Figure S3/S3A/#7-5-PAX6.tif]

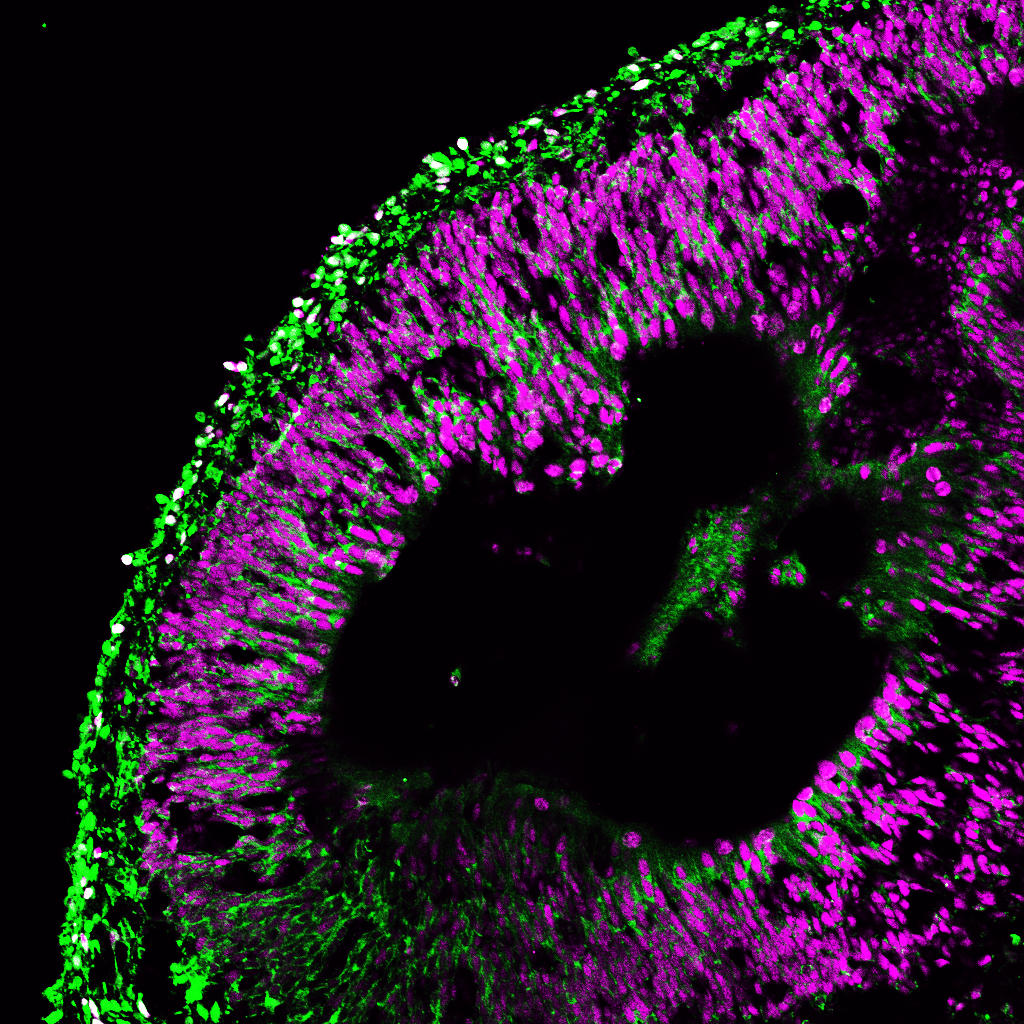

Supplement: Supplementary file 14 — Appendix Figure Source Data [file 44321_2025_302_MOESM14_ESM.zip › Appendix Figure S3/S3A/H9-merge.tif]

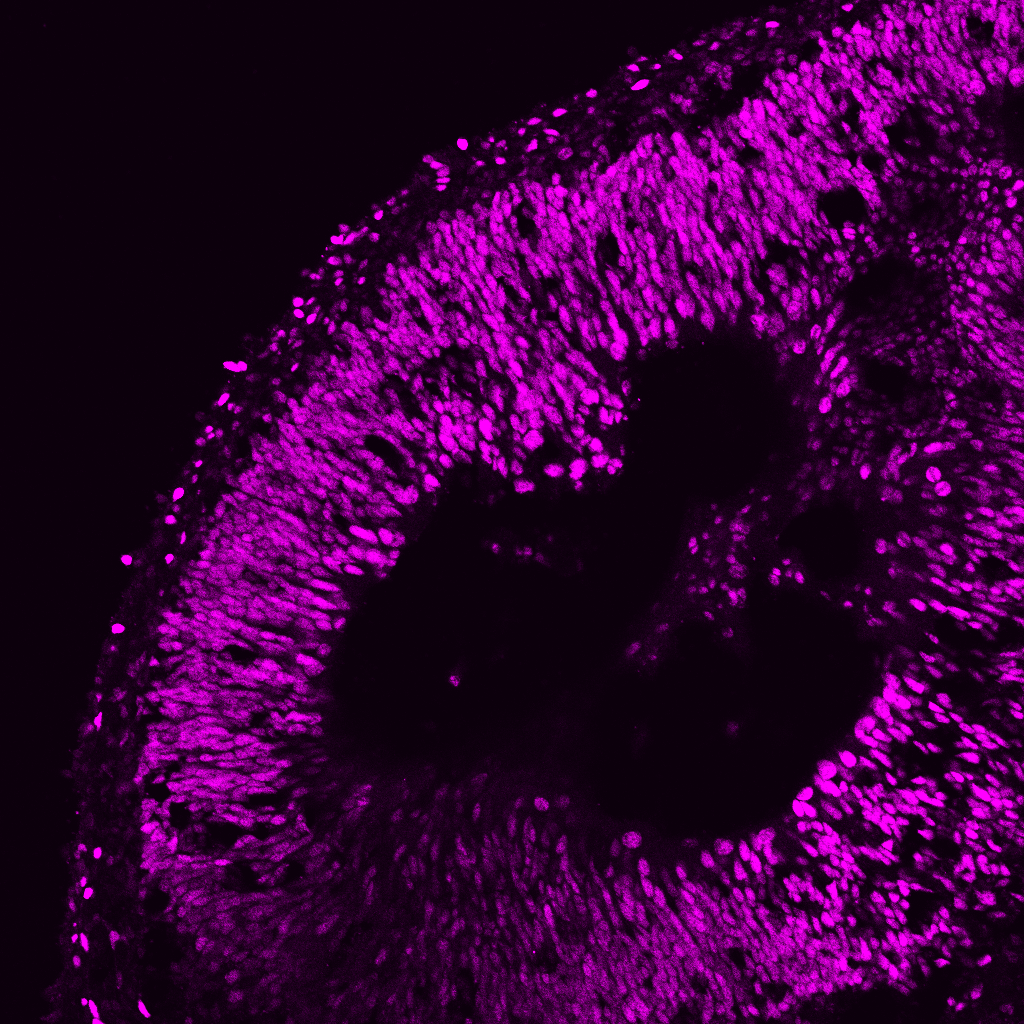

Supplement: Supplementary file 14 — Appendix Figure Source Data [file 44321_2025_302_MOESM14_ESM.zip › Appendix Figure S3/S3A/H9-PAX6.tif]

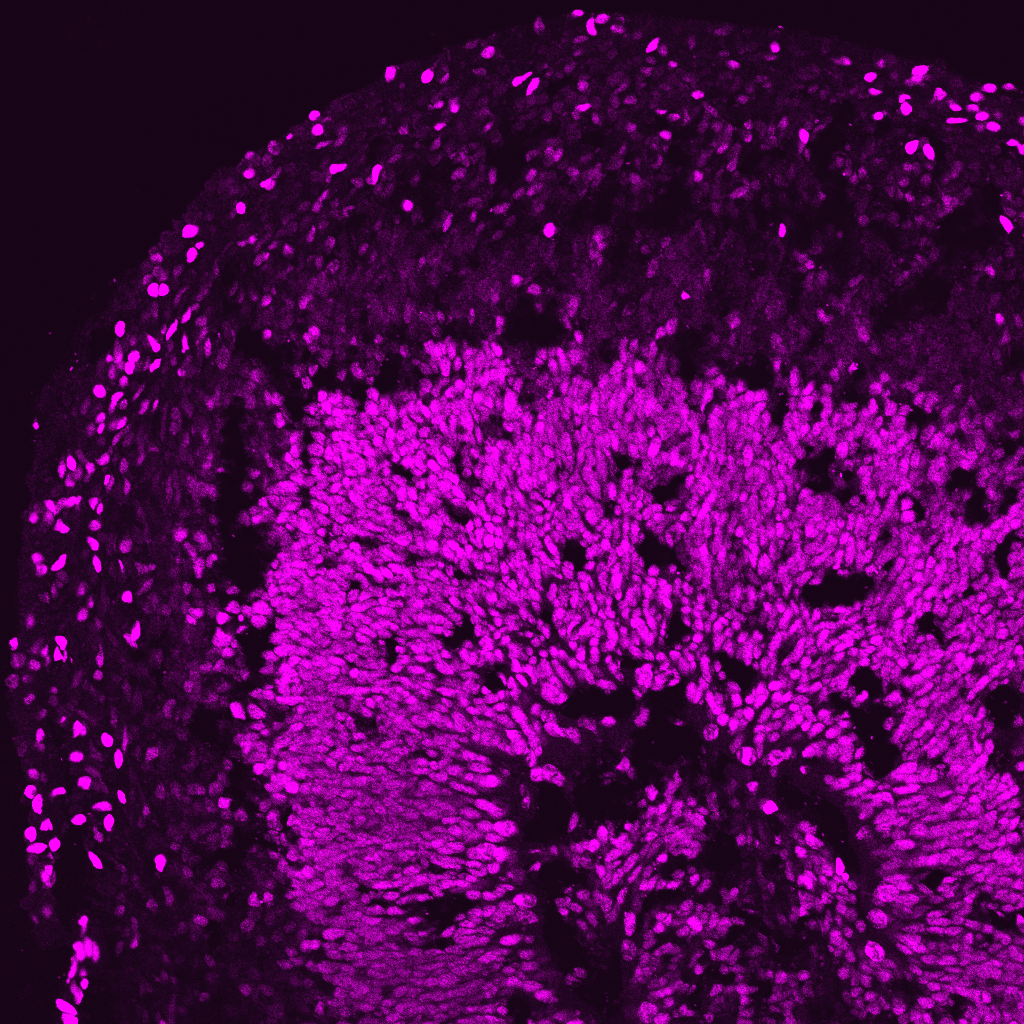

Supplement: Supplementary file 14 — Appendix Figure Source Data [file 44321_2025_302_MOESM14_ESM.zip › Appendix Figure S3/S3A/#12-3-PAX6.tif]

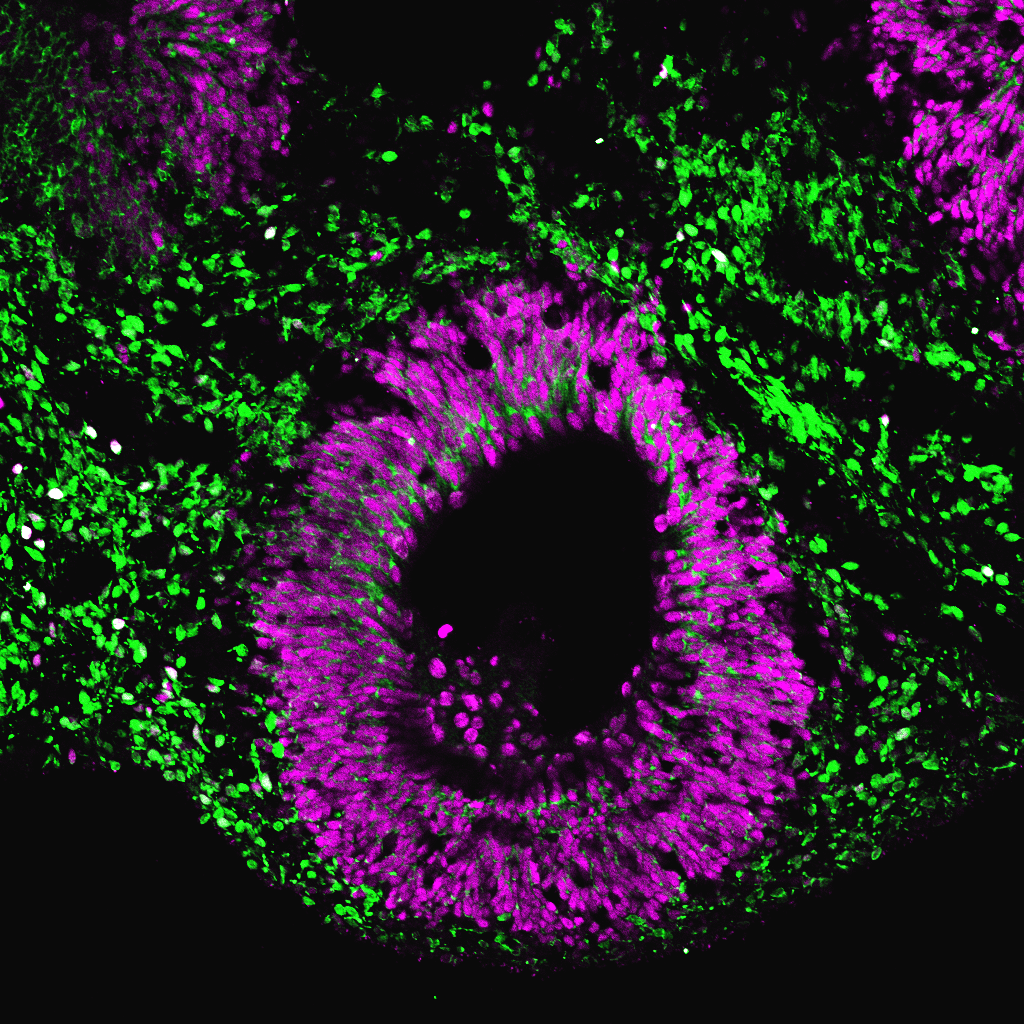

Supplement: Supplementary file 14 — Appendix Figure Source Data [file 44321_2025_302_MOESM14_ESM.zip › Appendix Figure S3/S3A/#7-5-merge.tif]

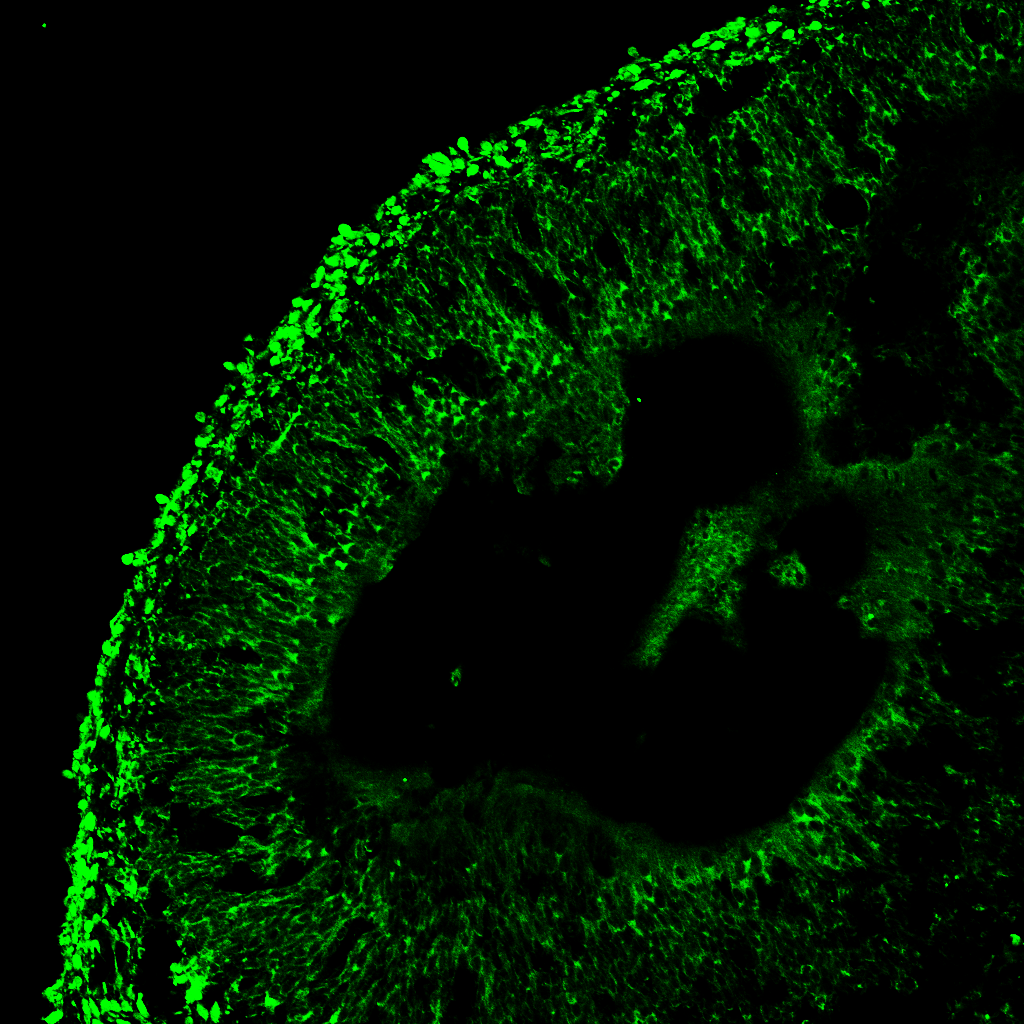

Supplement: Supplementary file 14 — Appendix Figure Source Data [file 44321_2025_302_MOESM14_ESM.zip › Appendix Figure S3/S3A/H9-HuCD.tif]

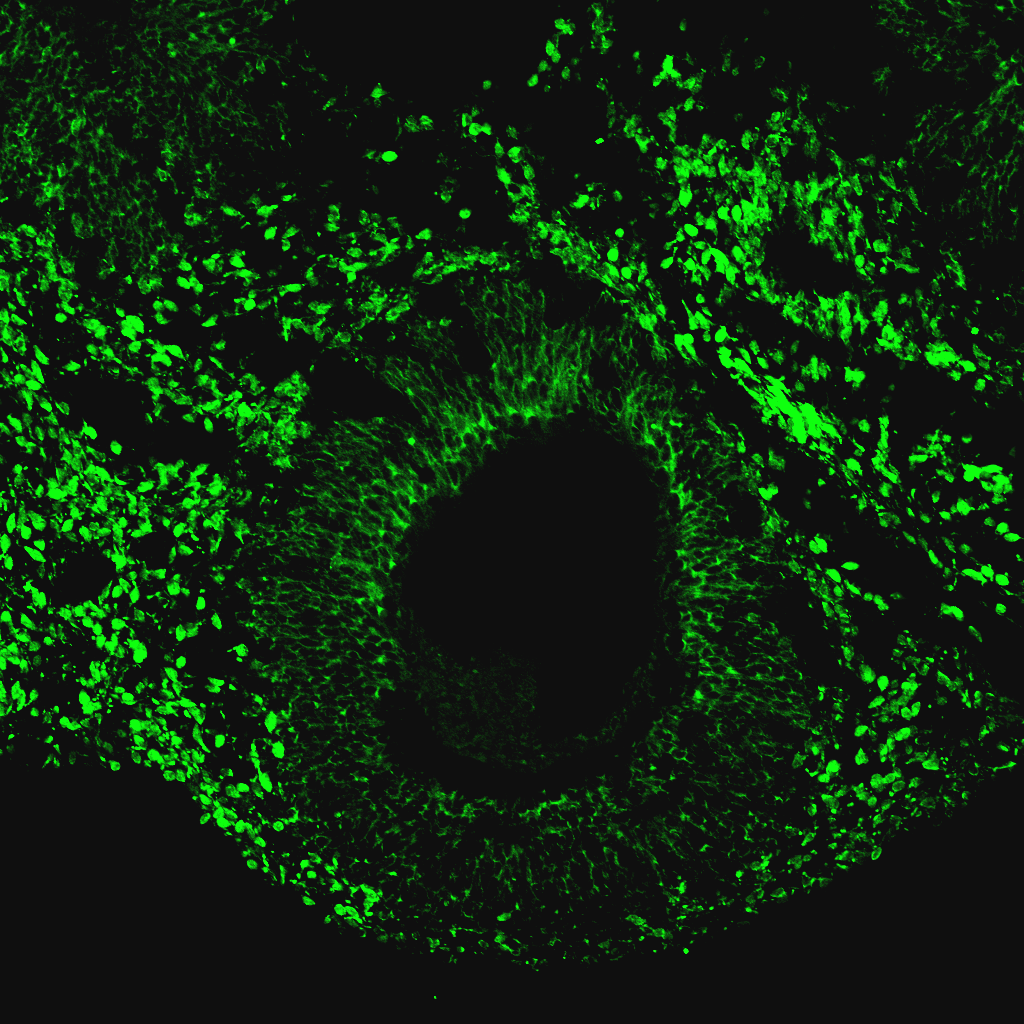

Supplement: Supplementary file 14 — Appendix Figure Source Data [file 44321_2025_302_MOESM14_ESM.zip › Appendix Figure S3/S3A/#7-5-HuCD.tif]

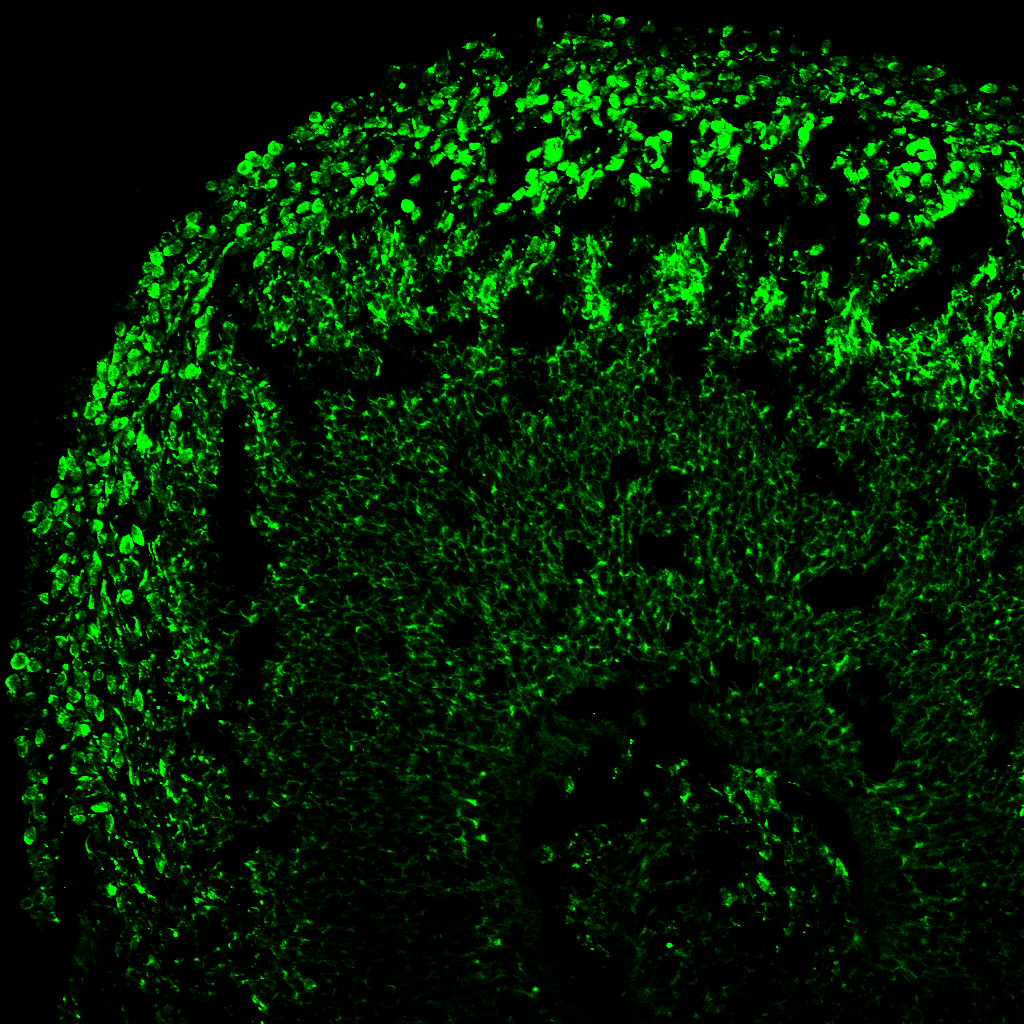

Supplement: Supplementary file 14 — Appendix Figure Source Data [file 44321_2025_302_MOESM14_ESM.zip › Appendix Figure S3/S3A/#12-3-HuCD.tif]

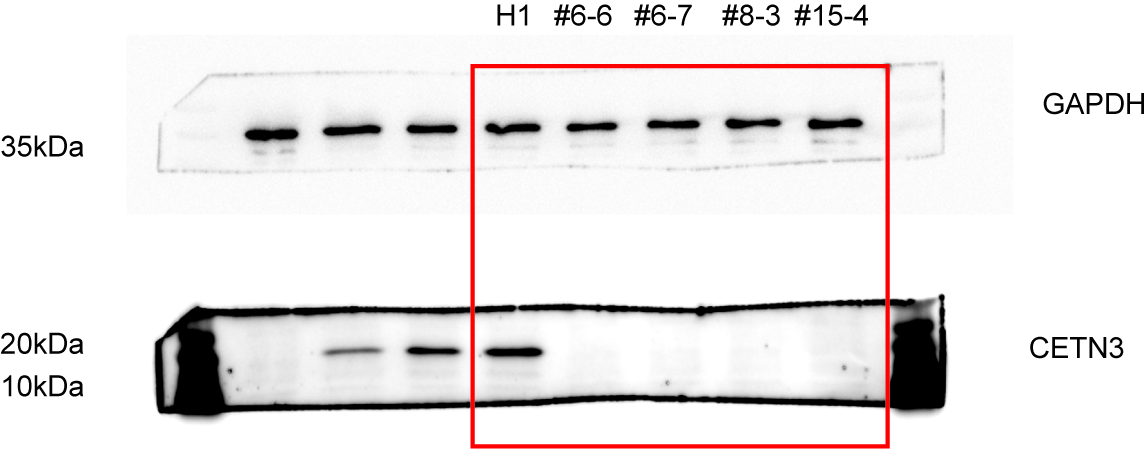

Supplement: Supplementary file 14 — Appendix Figure Source Data [file 44321_2025_302_MOESM14_ESM.zip › Appendix Figure S4/S4C/Western blot.tif]

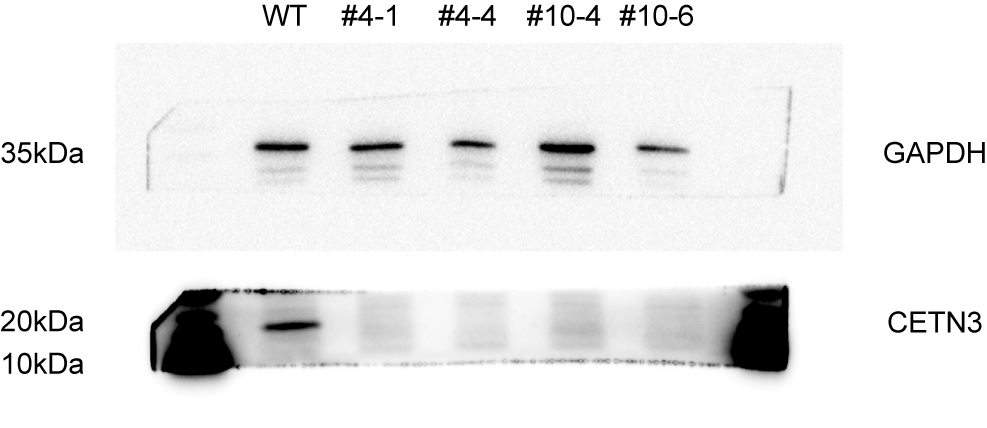

Supplement: Supplementary file 14 — Appendix Figure Source Data [file 44321_2025_302_MOESM14_ESM.zip › Appendix Figure S4/S4D/Western blot.tif]

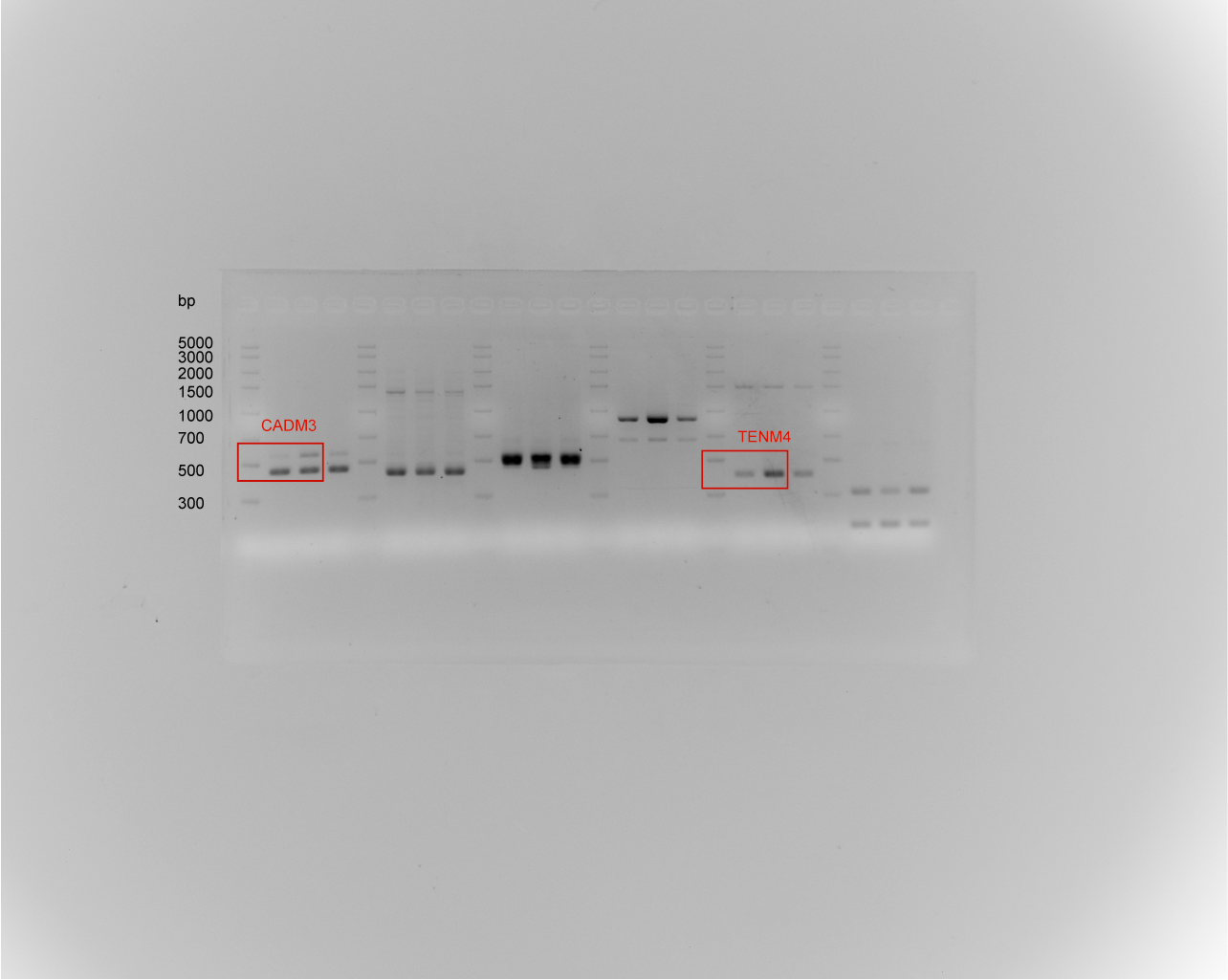

Supplement: Supplementary file 14 — Appendix Figure Source Data [file 44321_2025_302_MOESM14_ESM.zip › Appendix Figure S5/4A-B/RTPCR.tif]

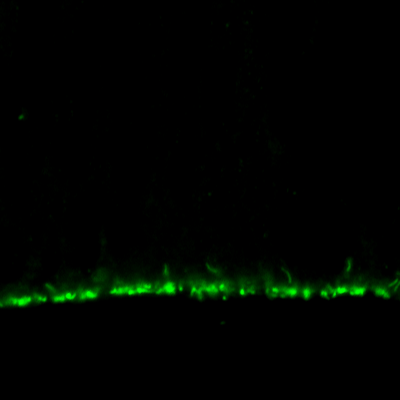

Supplement: Supplementary file 14 — Appendix Figure Source Data [file 44321_2025_302_MOESM14_ESM.zip › Appendix Figure S5/4C/H9-ARL3b.png]

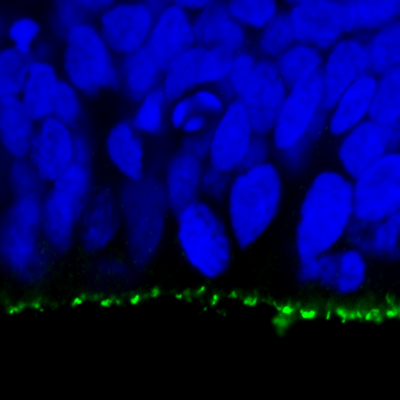

Supplement: Supplementary file 14 — Appendix Figure Source Data [file 44321_2025_302_MOESM14_ESM.zip › Appendix Figure S5/4C/#12-3-merge.tif]

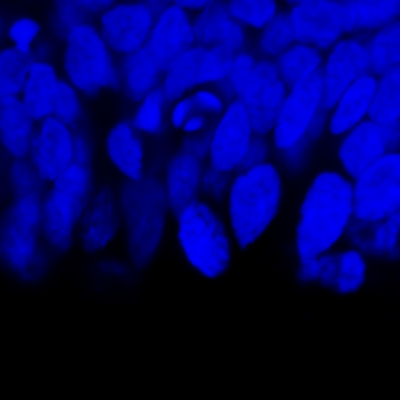

Supplement: Supplementary file 14 — Appendix Figure Source Data [file 44321_2025_302_MOESM14_ESM.zip › Appendix Figure S5/4C/#12-3-DAPI.png]

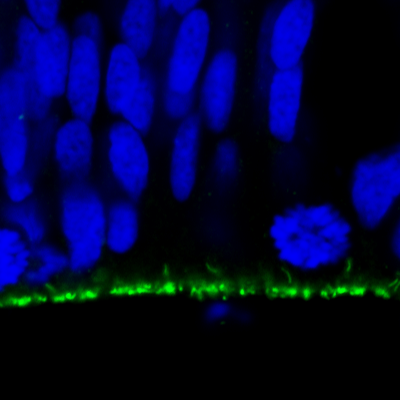

Supplement: Supplementary file 14 — Appendix Figure Source Data [file 44321_2025_302_MOESM14_ESM.zip › Appendix Figure S5/4C/H9-merge.tif]

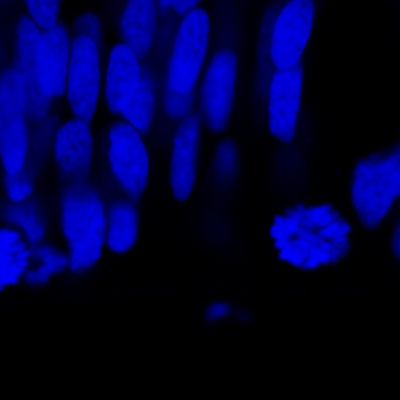

Supplement: Supplementary file 14 — Appendix Figure Source Data [file 44321_2025_302_MOESM14_ESM.zip › Appendix Figure S5/4C/H9-DAPI.png]

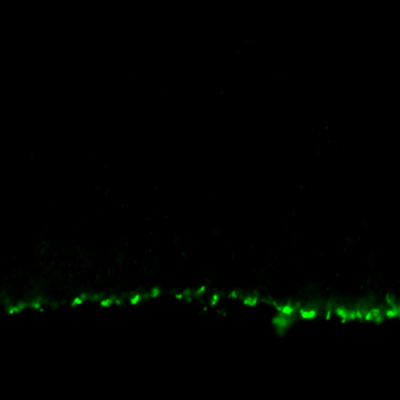

Supplement: Supplementary file 14 — Appendix Figure Source Data [file 44321_2025_302_MOESM14_ESM.zip › Appendix Figure S5/4C/#12-3-ARL13B.png]
