# Supplementary material for: CETN3 deficiency induces microcephaly by disrupting neural stem/progenitor cell fate through impaired centrosome assembly and RNA splicing
Source: EMBO Mol Med. 2025 Sep 8;17(10):2735–61. doi: 10.1038/s44321-025-00302-7 (PMC12514221; doi:10.1038/s44321-025-00302-7)
Supplement: Supplementary file 15 — Expanded View Figures [file 44321_2025_302_MOESM15_ESM.pdf]

Expanded View Figures

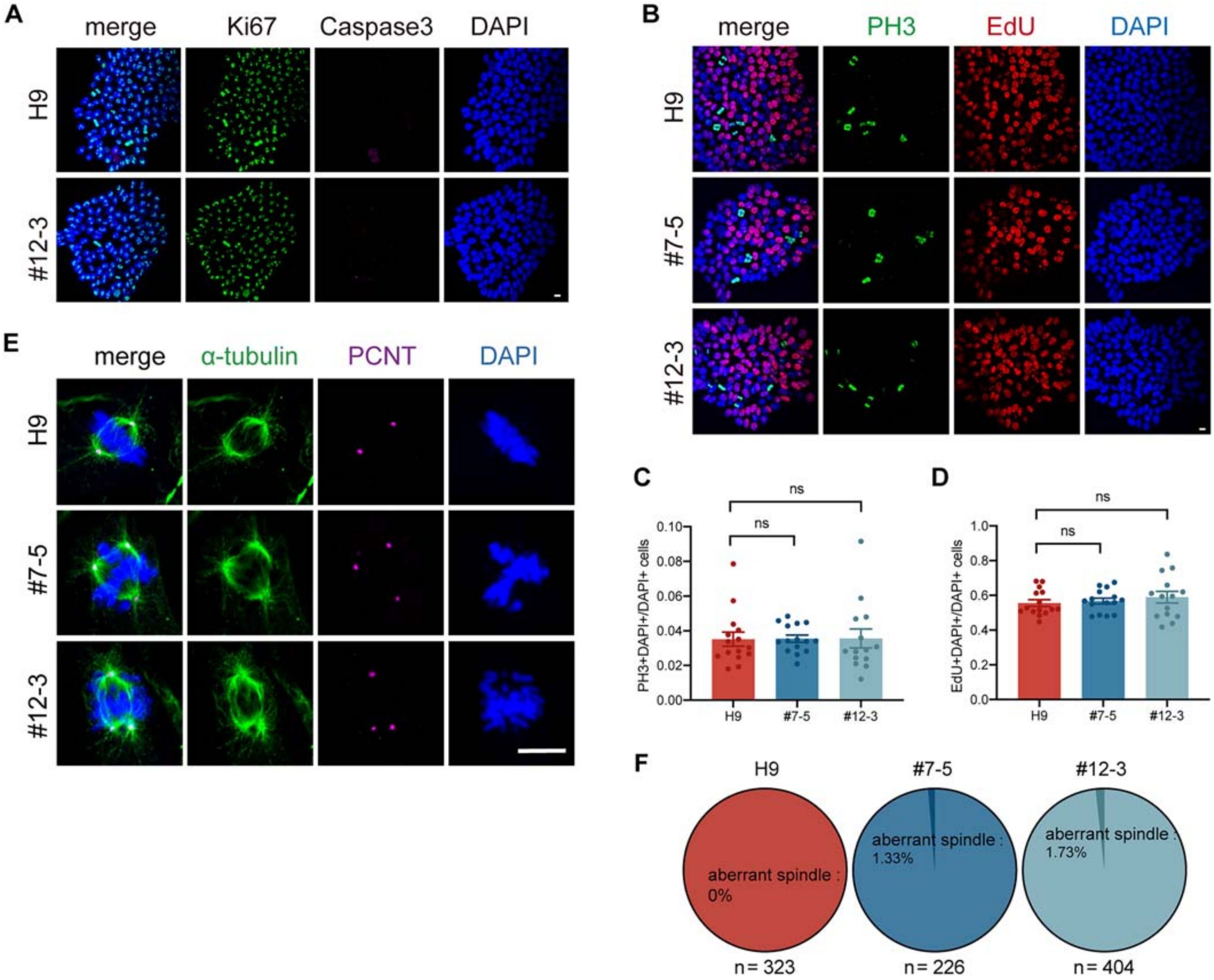

Figure EV1. Deletion of CETN3 doesn't affect proliferation and apoptosis in ESCs.

(**A**, **B**) Immunofluorescence of ESCs. Markers for proliferation, Ki67, EdU, PH3, and apoptosis, cleaved caspase-3, were stained. ESCs were labeled with EdU for 0.5 h. Scale bar: 10 μm. (**C**, **D**) Statistics of PH3 and EdU positive cells in (**B**). Each dot represented a clone (H9, $n = 15$; #7-5, $n = 15$; #12-3, $n = 14$). The cell during anaphase or telophase was considered a single cell. Data were shown as mean ± SEM. One-way ANOVA was used for differential analysis. ns: not significant. (**E**) Immunofluorescence of ESCs. Markers for microtubule, α-tubulin, and centrosome, PCNT, were stained. Representative cells undergoing mitosis were magnified for each cell line. Scale bar: 10 μm. (**F**) Statistics for the proportion of aberrant spindles in each cell line. Red: H9; blue: #7-5; green: #12-3. The darker sections in each pie chart indicated aberrant spindles, with the numbers representing their respective proportions. Data were collected from three independent experiments. Source data are available online for this figure.

 © The Author(s)

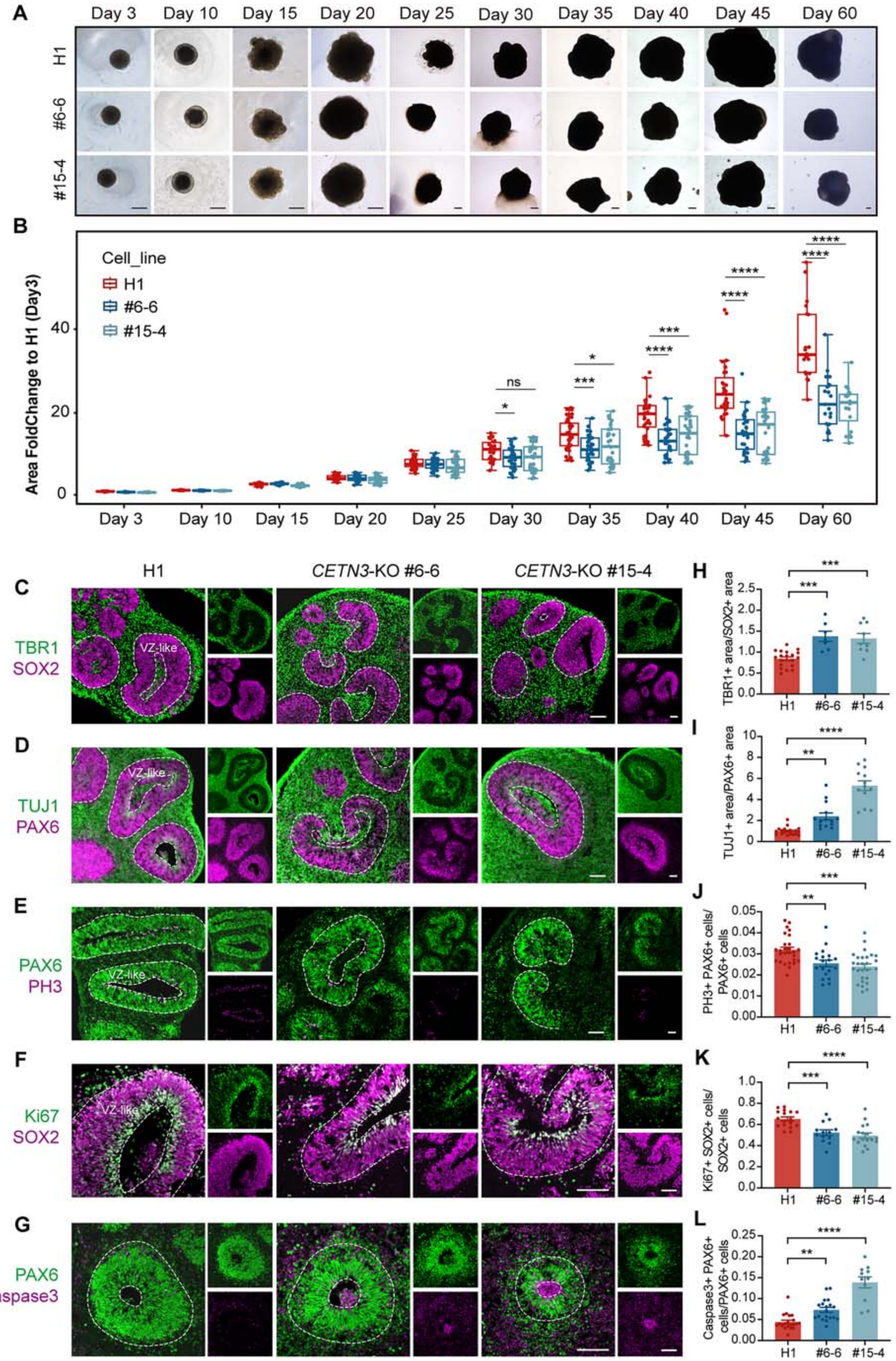
A
Day 3
Day 10
Day 15
Day 20
Day 25
Day 30
Day 35
Day 40
Day 45
Day 60
H1
#6-6
#15-4
B
Cell_line
H1
#6-6
#15-4
Area FoldChange to H1 (Day3)
C
H1
CETN3-KO #6-6
CETN3-KO #15-4
TBR1
SOX2
VZ-like
D
TUJ1
PAX6
E
PAX6
PH3
F
Ki67
SOX2
G
PAX6
Caspase3
H
TBR1+ area/SOX2+ area
I
TUJ1+ area/PAX6+ area
J
PH3+ PAX6+ cells/PAX6+ cells
K
Ki67+ SOX2+ cells/SOX2+ cells
L
Caspase3+ PAX6+ cells/PAX6+ cells

Figure EV2. Deficiency of CETN3 interferes with differentiation and proliferation of NS/PCs in hCOs derived from H1.

(**A**) Representative images showing the morphology and size of organoids at various stages of culture. Scale bar: 400 μm. (**B**) Quantification of organoid size at different culture time points. Data were derived from two independent batches and normalized to H1 (day 3). Each plot represents one organoid (day 3: $n = 16$; day 10: $n = 15$; day 15: H1, $n = 34$, #6-6, $n = 32$, #15-4, $n = 37$; day 20: H1, $n = 38$, #6-6, $n = 35$, #15-4, $n = 37$; day 25: H1, $n = 34$, #6-6, $n = 31$, #15-4, $n = 33$; day 30: H1, $n = 34$, #6-6, $n = 33$, #15-4, $n = 34$; day 35: H1, $n = 40$, #6-6, $n = 34$, #15-4, $n = 38$; day 40: H1, $n = 32$, #6-6, $n = 33$, #15-4, $n = 36$; day 45: H1, $n = 31$, #6-6, $n = 28$, #15-4, $n = 32$; day 60: H1, $n = 21$, #6-6, $n = 21$, #15-4, $n = 20$). Boxes represent the IQR from the first to third quartile, with the median shown as a horizontal line. Whiskers extend to the most extreme data points within 1.5× IQR; outliers beyond this range are shown as individual dots. Day 30: $P = 0.011$ (H1 vs. #6-6), $P = 0.059$ (H1 vs. #15-4); day 35: $P = 0.000164$ (H1 vs. #6-6), $P = 0.012$ (H1 vs. #15-4); day 40: $P < 0.0001$ (H1 vs. #6-6), $P = 0.000295$ (H1 vs. #15-4); day 45: $P < 0.0001$ (H1 vs. #6-6), $P < 0.0001$ (H1 vs. #15-4); day 60: $P < 0.0001$ (H1 vs. #6-6), $P < 0.0001$ (H1 vs. #15-4). Statistical analysis was conducted using one-way ANOVA. *$P < 0.05$; ***$P < 0.001$; ****$P < 0.0001$; ns: not significant. (**C**, **D**) Immunofluorescence staining of cryosections from day 45 hCOs. NS/PCs were labeled with SOX2 or PAX6, while neurons were identified using TBR1 or TUJ1. Scale bar: 100 μm. VZ: ventricular zone. (**E**, **F**) Immunofluorescence staining of cryosections from day 45 hCOs. Markers used included PAX6 or SOX2 for NS/PCs, PH3 and Ki67 for cell proliferation. Scale bar: 100 μm. (**G**) Immunofluorescence staining of cryosections from day 45 hCOs. Markers used included PAX6 for NS/PCs, cleaved caspase-3 for cell apoptosis. Scale bar: 100 μm. (**H–L**) Quantification of cell number or staining area for each marker. Each dot represented an individual rosette or several adjacent rosettes (**H**: H1, $n = 19$, #6-6, $n = 7$, #15-4, $n = 11$; **I**: H1, $n = 19$, #6-6, $n = 14$, #15-4, $n = 13$; **J**: H1, $n = 27$, #6-6, $n = 19$, #15-4, $n = 27$; **K**: H1, $n = 17$, #6-6, $n = 13$, #15-4, $n = 17$; **L**: H1, $n = 20$, #6-6, $n = 19$, #15-4, $n = 11$). Data were collected from organoids across two independent experiments, with results presented as mean ± SEM. (**H**) $P = 0.0002$ (H1 vs. #6-6), $P = 0.0002$ (H1 vs. #15-4); (**I**) $P = 0.0021$ (H1 vs. #6-6), $P < 0.0001$ (H1 vs. #15-4); (**J**) $P = 0.0051$ (H1 vs. #6-6), $P = 0.0001$ (H1 vs. #15-4); (**K**) $P = 0.0009$ (H1 vs. #6-6), $P < 0.0001$ (H1 vs. #15-4); (**L**) $P = 0.0048$ (H1 vs. #6-6), $P < 0.0001$ (H1 vs. #15-4). Differential analysis was performed using one-way ANOVA. **$P < 0.01$; ***$P < 0.001$; ****$P < 0.0001$. Source data are available online for this figure.

© The Author(s)

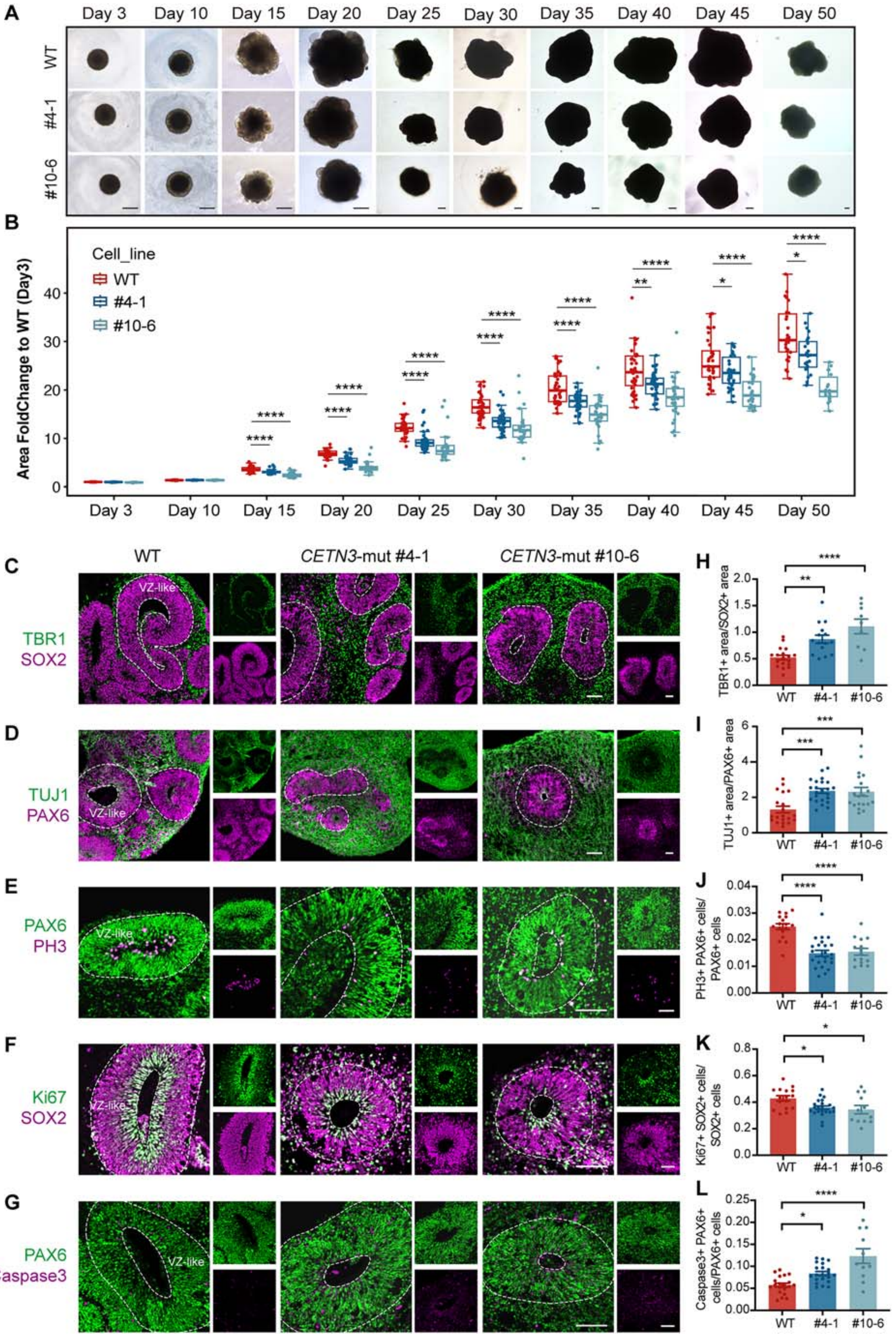
A
Day 3
Day 10
Day 15
Day 20
Day 25
Day 30
Day 35
Day 40
Day 45
Day 50
WT
#4-1
#10-6
B
Cell_line
WT
#4-1
#10-6
Area FoldChange to WT (Day3)
C
WT
CETN3-mut #4-1
CETN3-mut #10-6
TBR1
SOX2
VZ-like
D
TUJ1
PAX6
E
PAX6
PH3
F
Ki67
SOX2
G
PAX6
Caspase3
H
TBR1+ area/SOX2+ area
I
TUJ1+ area/PAX6+ area
J
PH3+ PAX6+ cells/PAX6+ cells
K
Ki67+ SOX2+ cells/SOX2+ cells
L
Caspase3+ PAX6+ cells/PAX6+ cells

Figure EV3. Loss-of-function mutations in *CETN3* impair NS/PC differentiation and proliferation in iPSC-derived hCOs.

(**A**) Representative images showing the morphology and size of organoids at various stages of culture. Scale bar: 400 µm. (**B**) Quantification of organoid size at different culture time points. Data were derived from two independent batches and normalized to WT group (day 3). Each plot represents one organoid (day 3: $n = 16$; day 10: $n = 16$; day 15: WT, $n = 39$, #4-1, $n = 39$, #10-6, $n = 37$; day 20: WT, $n = 35$, #4-1, $n = 41$, #10-6, $n = 25$; day 25: WT, $n = 37$, #4-1, $n = 41$, #10-6, $n = 29$; day 30: WT, $n = 40$, #4-1, $n = 41$, #10-6, $n = 32$; day 35: WT, $n = 40$, #4-1, $n = 40$, #10-6, $n = 35$; day 40: WT, $n = 40$, #4-1, $n = 42$, #10-6, $n = 36$; day 45: WT, $n = 36$, #4-1, $n = 34$, #10-6, $n = 32$; day 50: WT, $n = 26$, #4-1, $n = 23$, #10-6, $n = 18$). Boxes represent the IQR from the first to third quartile, with the median shown as a horizontal line. Whiskers extend to the most extreme data points within 1.5× IQR; outliers beyond this range are shown as individual dots. Day 15: $P < 0.0001$ (WT vs. #4-1), $P < 0.0001$ (WT vs. #10-6); day 20: $P < 0.0001$ (WT vs. #4-1), $P < 0.0001$ (WT vs. #10-6); day 25: $P < 0.0001$ (WT vs. #4-1), $P < 0.0001$ (WT vs. #10-6); day 30: $P < 0.0001$ (WT vs. #4-1), $P < 0.0001$ (WT vs. #10-6); day 35: $P < 0.0001$ (WT vs. #4-1), $P < 0.0001$ (WT vs. #10-6); day 40: $P = 0.002$ (WT vs. #4-1), $P < 0.0001$ (WT vs. #10-6); day 45: $P = 0.032$ (WT vs. #4-1), $P < 0.0001$ (WT vs. #10-6); day 50: $P = 0.012$ (WT vs. #4-1), $P < 0.0001$ (WT vs. #10-6). Statistical analysis was conducted using one-way ANOVA. *$P < 0.05$; **$P < 0.01$; ****$P < 0.0001$; ns not significant. (**C**, **D**) Immunofluorescence staining of cryosections from day 45 hCOs. NS/PCs were labeled with SOX2 or PAX6, while neurons were identified using TBR1 or TUJ1. Scale bar: 100 µm. VZ: ventricular zone. (**E**, **F**) Immunofluorescence staining of cryosections from day 45 hCOs. Markers used included PAX6 or SOX2 for NS/PCs, PH3 and Ki67 for cell proliferation. Scale bar: 100 µm. (**G**) Immunofluorescence staining of cryosections from day 45 hCOs. Markers used included PAX6 for NS/PCs, cleaved caspase-3 for cell apoptosis. Scale bar: 100 µm. (**H–L**) Quantification of cell number or staining area for each marker. Each dot represented an individual rosette or several adjacent rosettes (**H**: WT, $n = 18$, #4-1, $n = 15$, #10-6, $n = 9$; **I**: WT, $n = 18$, #4-1, $n = 21$, #10-6, $n = 17$; **J**: WT, $n = 17$, #4-1, $n = 23$, #10-6, $n = 15$; **K**: WT, $n = 16$, #4-1, $n = 22$, #10-6, $n = 12$; **L**: WT, $n = 20$, #4-1, $n = 21$, #10-6, $n = 11$). Data were collected from organoids across two independent experiments, with results presented as mean ± SEM. (**H**) $P = 0.0026$ (WT vs. #4-1), $P < 0.0001$ (WT vs. #10-6); (**I**) $P = 0.0003$ (WT vs. #4-1), $P = 0.0009$ (WT vs. #10-6); (**J**) $P < 0.0001$ (WT vs. #4-1), $P < 0.0001$ (WT vs. #10-6); (**K**) $P = 0.0199$ (WT vs. #4-1), $P = 0.0153$ (WT vs. #10-6); (**L**) $P = 0.0172$ (WT vs. #4-1), $P < 0.0001$ (WT vs. #10-6). Differential analysis was performed using one-way ANOVA. *$P < 0.05$; **$P < 0.01$; ***$P < 0.001$; ****$P < 0.0001$. Source data are available online for this figure.

© The Author(s)

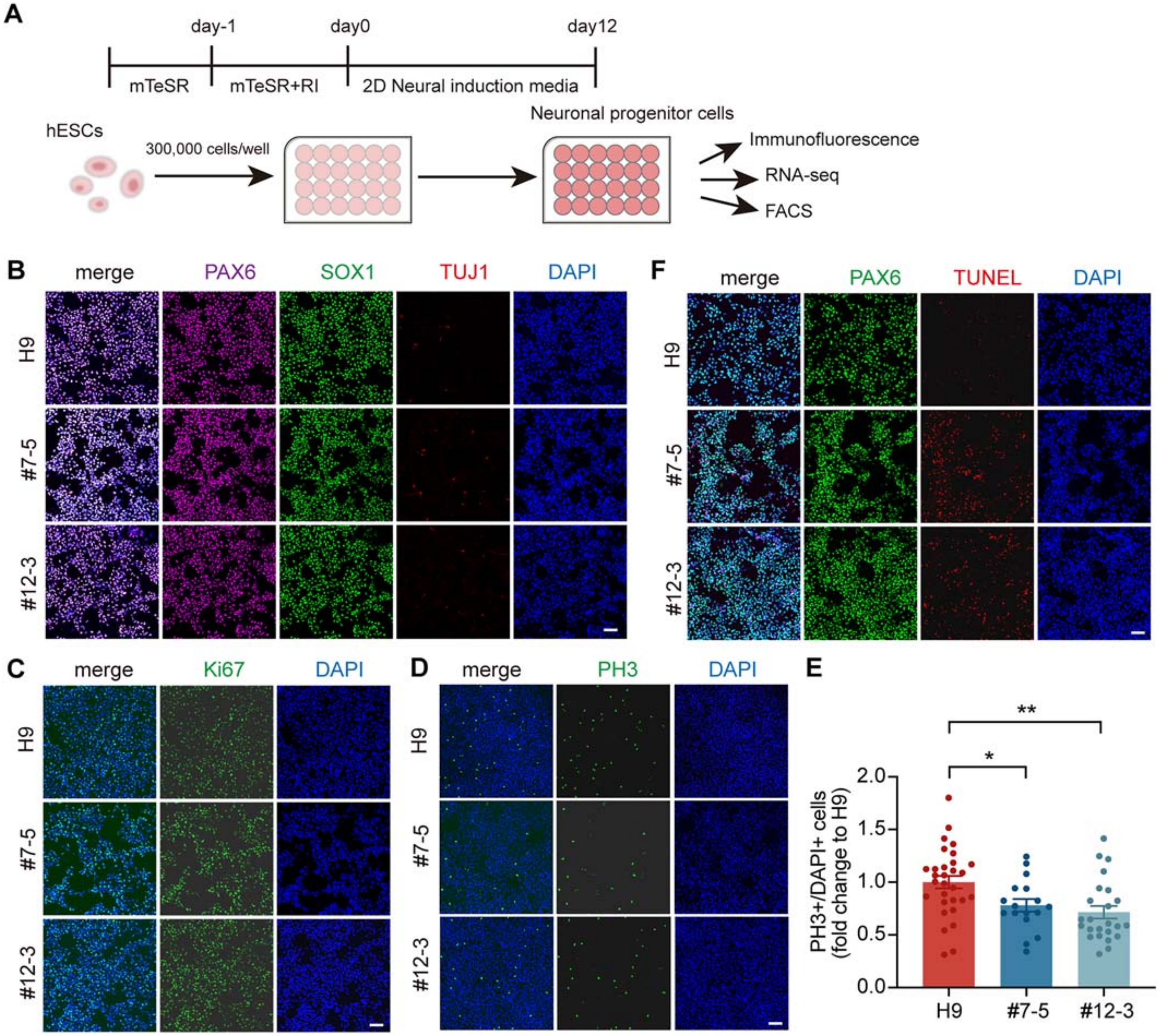

Figure EV4. Deletion of CETN3 affects proliferation and apoptosis in NS/PCs.

(**A**) Technological process for 2D differentiation. On day −1, 300,000 cells were plated in a Matrigel-coated 24-well plate, and the ROCK inhibitor was added. On day 0, the media were switched from mTesR to 2D neural induction media. Fresh 2D neural induction media were replaced daily until day 12 to obtain NS/PCs. The NS/PCs were then used for immunofluorescence, RNA-seq, and flow cytometry analysis. (**B**) Immunofluorescence of NS/PCs. Markers for NS/PCs, PAX6 and SOX1, and neurons, TUJ1, were stained. Scale bar: 100 µm. (**C**, **D**) Immunofluorescence of NS/PCs. Markers for proliferation, Ki67 and PH3, were stained. Scale bar: 100 µm. (**E**) Statistics of PH3-positive cells in (**D**). Each dot indicated the statistical result of an individual image (H9, $n = 30$; #7-5, $n = 17$; #12-3, $n = 24$). The cell during anaphase or telophase was considered a single cell. Data were collected from three independent experiments, with results presented as mean ± SEM. $P = 0.0347$ (H9 vs. #7-5), $P = 0.0017$ (H9 vs. #12-3). One-way ANOVA was used for differential analysis. $^*P < 0.05$; $^{**}P < 0.01$. (**F**) TUNEL technique to observe apoptosis in NS/PCs, PAX6 was co-stained. Scale bar: 100 µm. Source data are available online for this figure.

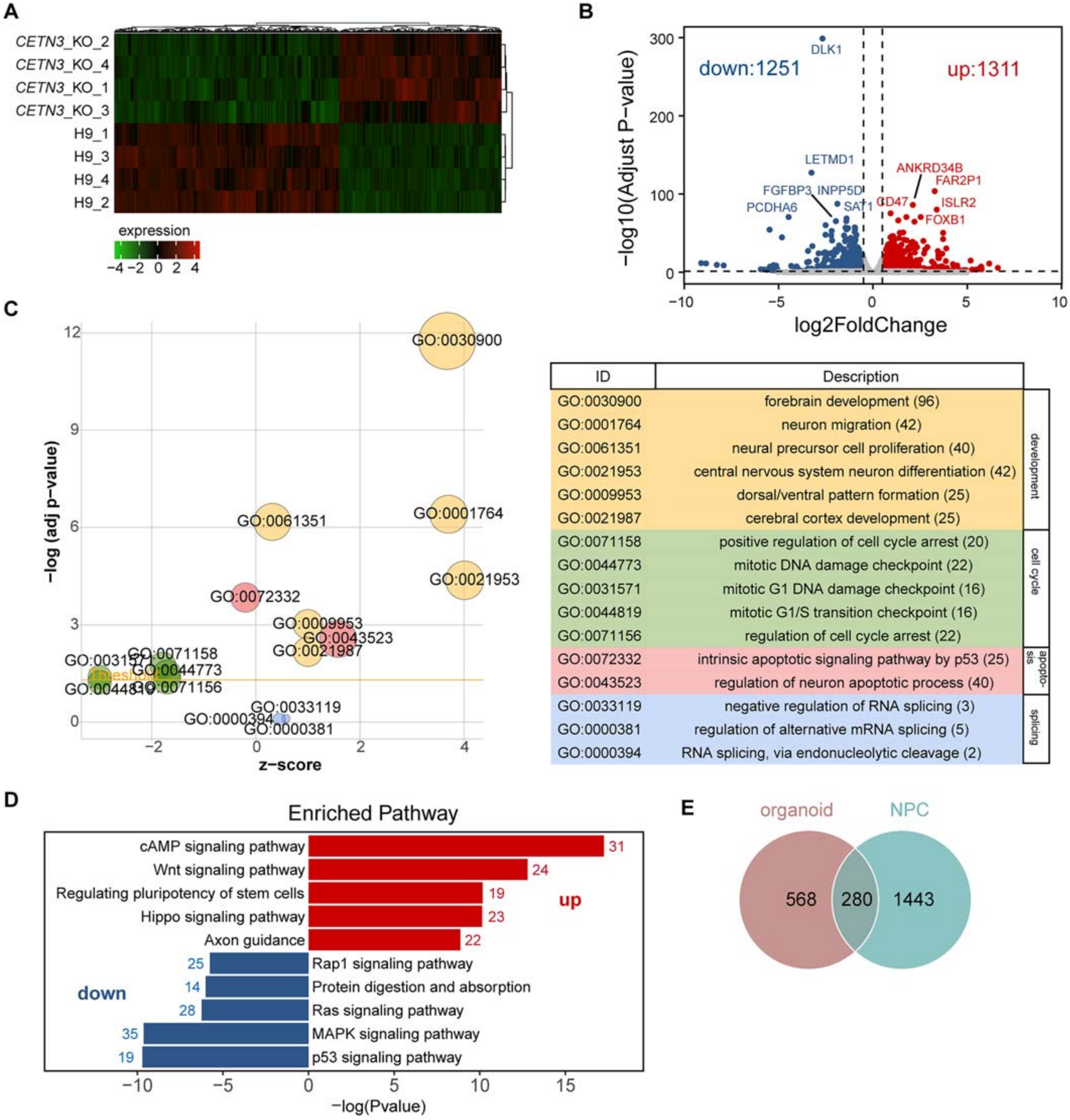

Figure EV5. Deletion of CETN3 disrupts the expression of genes related to neuronal proliferation, differentiation, apoptosis, and splicing in NS/PCs.

(**A**) Heatmap showed DEGs (*p*adj <0.05, |log2FC| >0.5) between H9 and *CETN3*-KO NS/PCs at day 12 from 2D differentiation. Four replicates for each group. (**B**) Volcano plot showed DEGs between H9 and *CETN3*-KO NS/PCs. The top five genes were labeled in the plot. Statistical significance of differential gene expression was determined in DESeq2 using a Wald test, with *p* values adjusted for multiple comparisons by the Benjamini–Hochberg procedure. (**C**) GO_MF enrichment bubble plot (left), and description for the GO terms (right). The GO terms were manually classified into four categories according to their biological functions, and gene counts for each term were labeled behind. (**D**) KEGG enrichment results. Pathways related to brain development were selected. The numbers beside each bar were gene counts for each pathway. Red, upregulated pathway; blue, downregulated pathway. Enrichment analysis was performed using clusterProfiler to identify GO terms and KEGG pathways enriched among differentially expressed genes. Gene set *P* values were calculated using a hypergeometric test and adjusted for multiple comparisons using the Benjamini–Hochberg method. (**E**) Intersection of differentially spliced genes in organoids (day 45) and NS/PCs from 2D differentiation.

 © The Author(s)

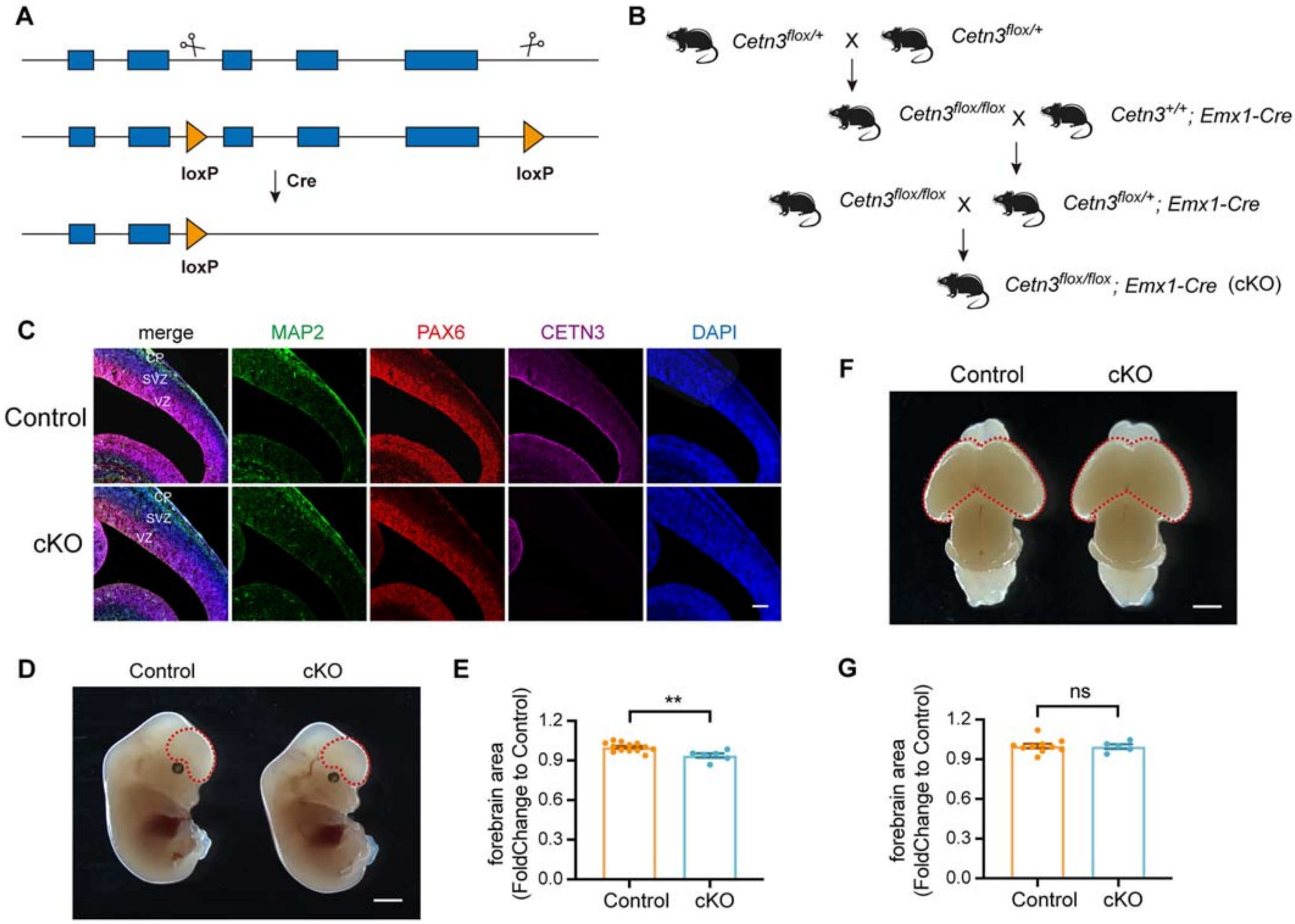

Figure EV6. Conditional deletion of *Cetn3* in the mouse forebrain affects brain development exclusively during the embryonic stage.

(**A**) Schematic of the *Cetn3* knockout strategy in mice. Two loxP elements were inserted into intron2-3 and 3′ UTR, respectively, enabling Cre-mediated excision of exons 3–5. (**B**) Breeding strategy used to generate *Cetn3*-cKO mice. (**C**) Immunofluorescence staining of coronal cryosections from E13.5 mouse brains. Markers for neurons (MAP2), NS/PCs (PAX6), and CETN3 were co-stained. VZ: ventricular zone, SVZ: subventricular zone, CP: cortical plate. Scale bar: 100 μm. (**D**) Representative images of E13.5 embryos from control and *Cetn3*-cKO mice. Forebrains were outlined with red dashed lines. Scale bar: 1 mm. (**E**) Quantification of forebrain area in E13.5 mice. Data were normalized to control. Control, $n = 13$; cKO, $n = 6$ (from three litters). Data were shown as mean ± SEM. $P = 0.0025$ (Control vs. cKO). An unpaired *t*-test was used for differential analysis. $^{**}P < 0.01$. (**F**) Representative images of P0 brains from control and *Cetn3*-cKO mice. Forebrains were outlined with red dashed lines. Scale bar: 1 mm. (**G**) Quantification of forebrain area in P0 mice. Data were normalized to control. Control, $n = 10$; cKO, $n = 5$ (from three litters). Data were shown as mean ± SEM. $P = 0.8759$ (Control vs. cKO). An unpaired *t*-test was used for differential analysis. ns: not significant. Source data are available online for this figure.

© The Author(s)
